# Supplementary material for: Expanding Training in Quality Improvement and Patient Safety Through a Multispecialty Graduate Medical Education Curriculum Designed for Fellows
Source: MedEdPORTAL. 2020 Dec 30;16:11064. doi: 10.15766/mep_2374-8265.11064 (PMC7780740; doi:10.15766/mep_2374-8265.11064)
Supplement: Supplementary file 1 — Foundations in Patient Safety Teaching Slides.pptxFoundations in Patient Safety Playbook and Small-Group Activities.docxAdverse Events Into QI Teaching Slides.pptxAdverse Events Into QI Playbook and Small-Group Activities.docxQuality in Academics Teaching Slides.pptxQuality in Academics Playbook and Small-Group Activities.docxFoundations in Patient Safety Assessment Survey.docxAdverse Events Into QI Assessment Survey.docxQuality in Academics Assessment Survey.docx [file mep_2374-8265.11064-s001.zip › A. Foundations in Patient Safety Teaching Slides.pptx]

## Slide 1
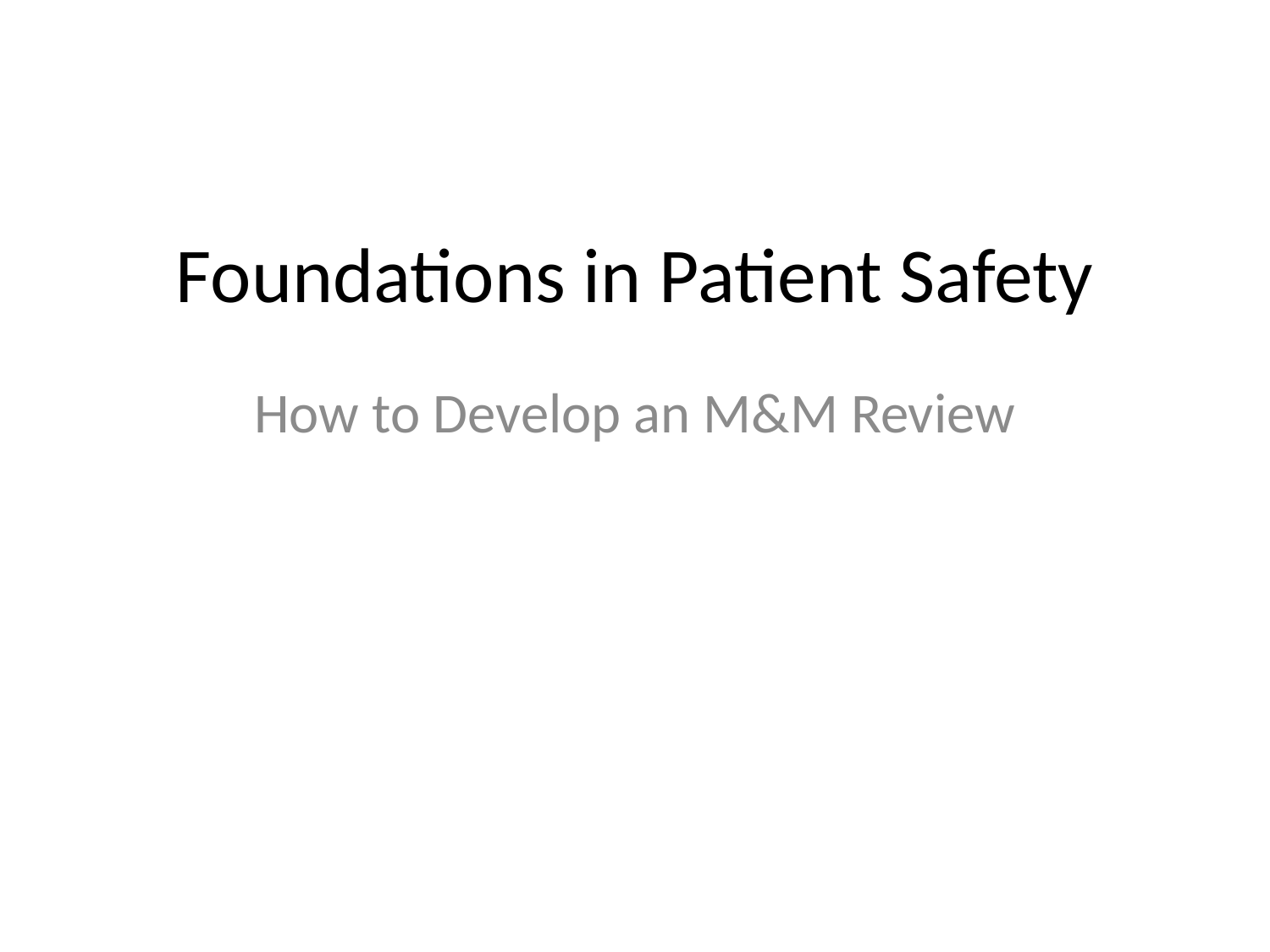

# Foundations in Patient Safety
How to Develop an M&M Review

## Slide 2
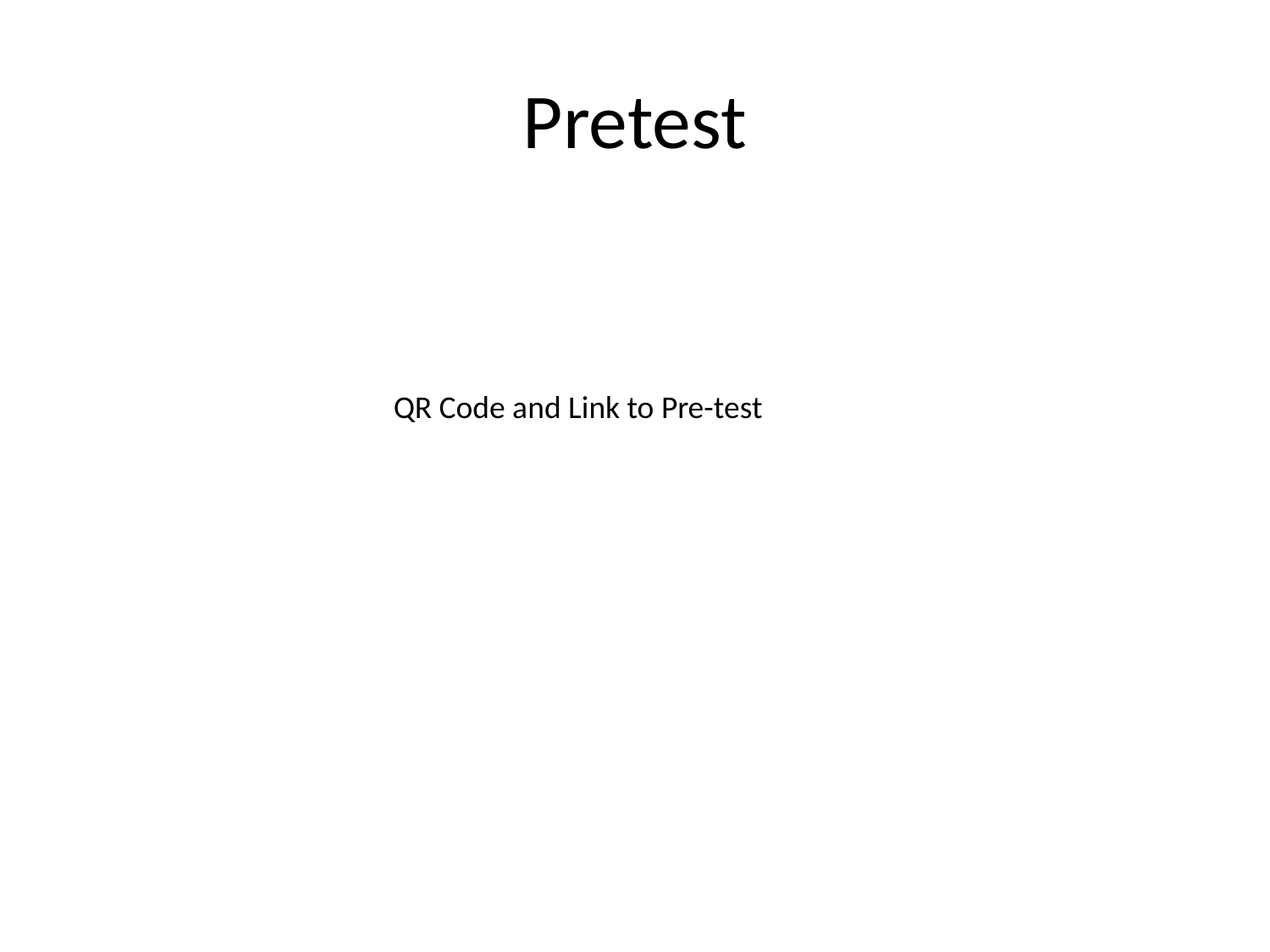

Pretest
QR Code and Link to Pre-test

## Slide 3
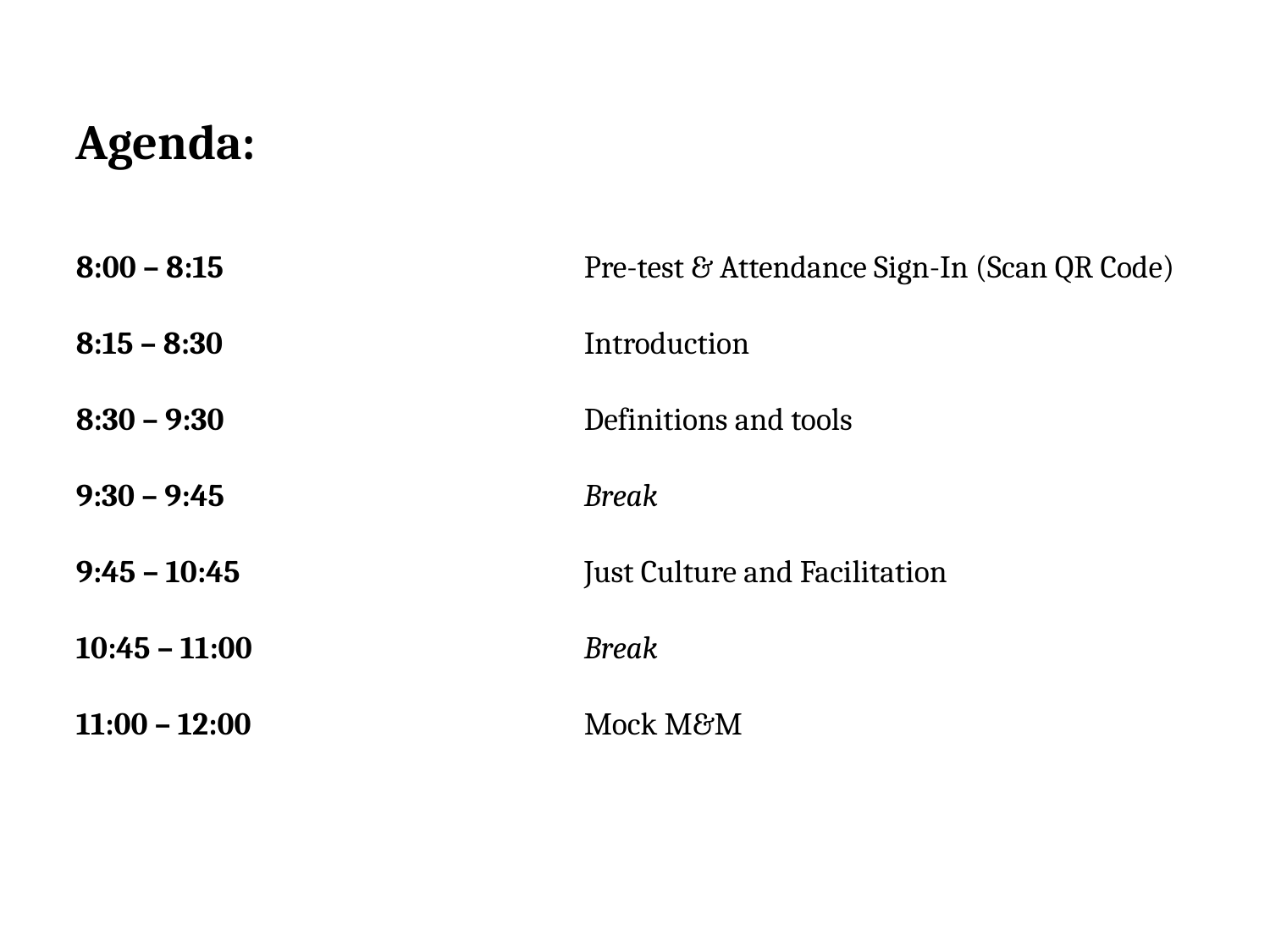

Agenda:
8:00 – 8:15			Pre-test & Attendance Sign-In (Scan QR Code)
8:15 – 8:30 			Introduction
8:30 – 9:30 			Definitions and tools
9:30 – 9:45 			Break
9:45 – 10:45		 	Just Culture and Facilitation
10:45 – 11:00 			Break
11:00 – 12:00 			Mock M&M

## Slide 4
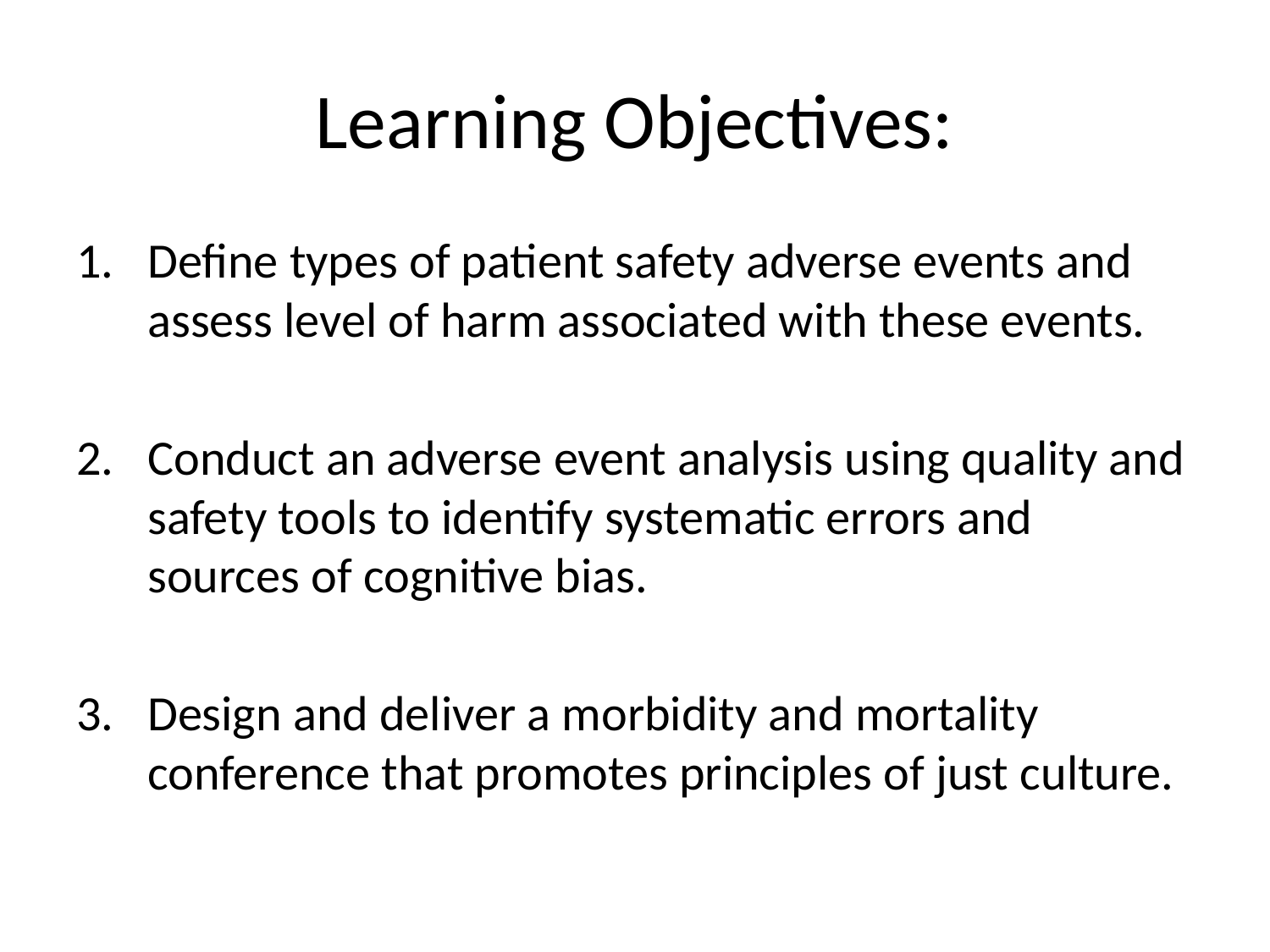

# Learning Objectives:
Define types of patient safety adverse events and assess level of harm associated with these events.
Conduct an adverse event analysis using quality and safety tools to identify systematic errors and sources of cognitive bias.
Design and deliver a morbidity and mortality conference that promotes principles of just culture.

## Slide 5
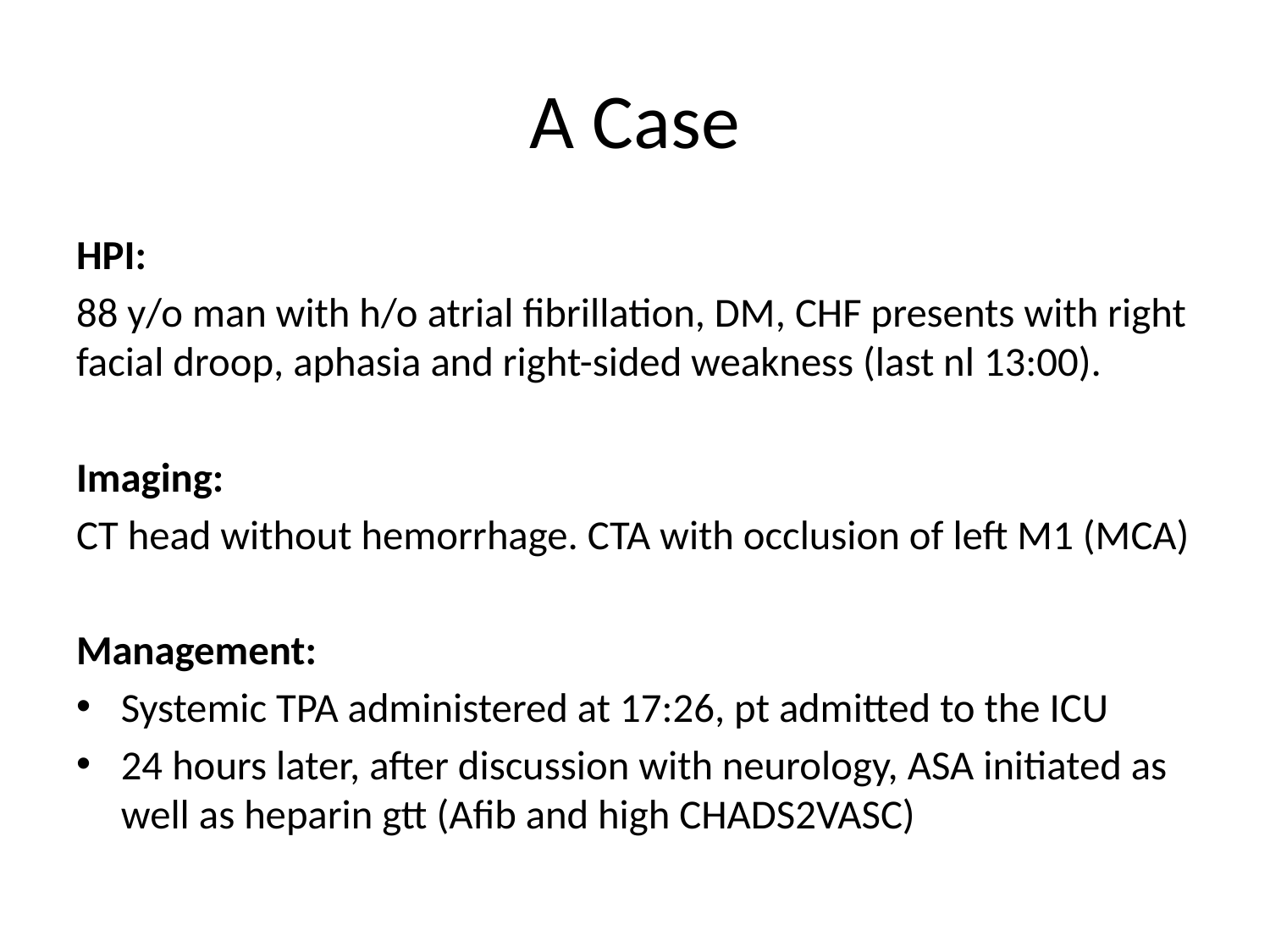

# A Case
HPI:
88 y/o man with h/o atrial fibrillation, DM, CHF presents with right facial droop, aphasia and right-sided weakness (last nl 13:00).
Imaging:
CT head without hemorrhage. CTA with occlusion of left M1 (MCA)
Management:
Systemic TPA administered at 17:26, pt admitted to the ICU
24 hours later, after discussion with neurology, ASA initiated as well as heparin gtt (Afib and high CHADS2VASC)

## Slide 6
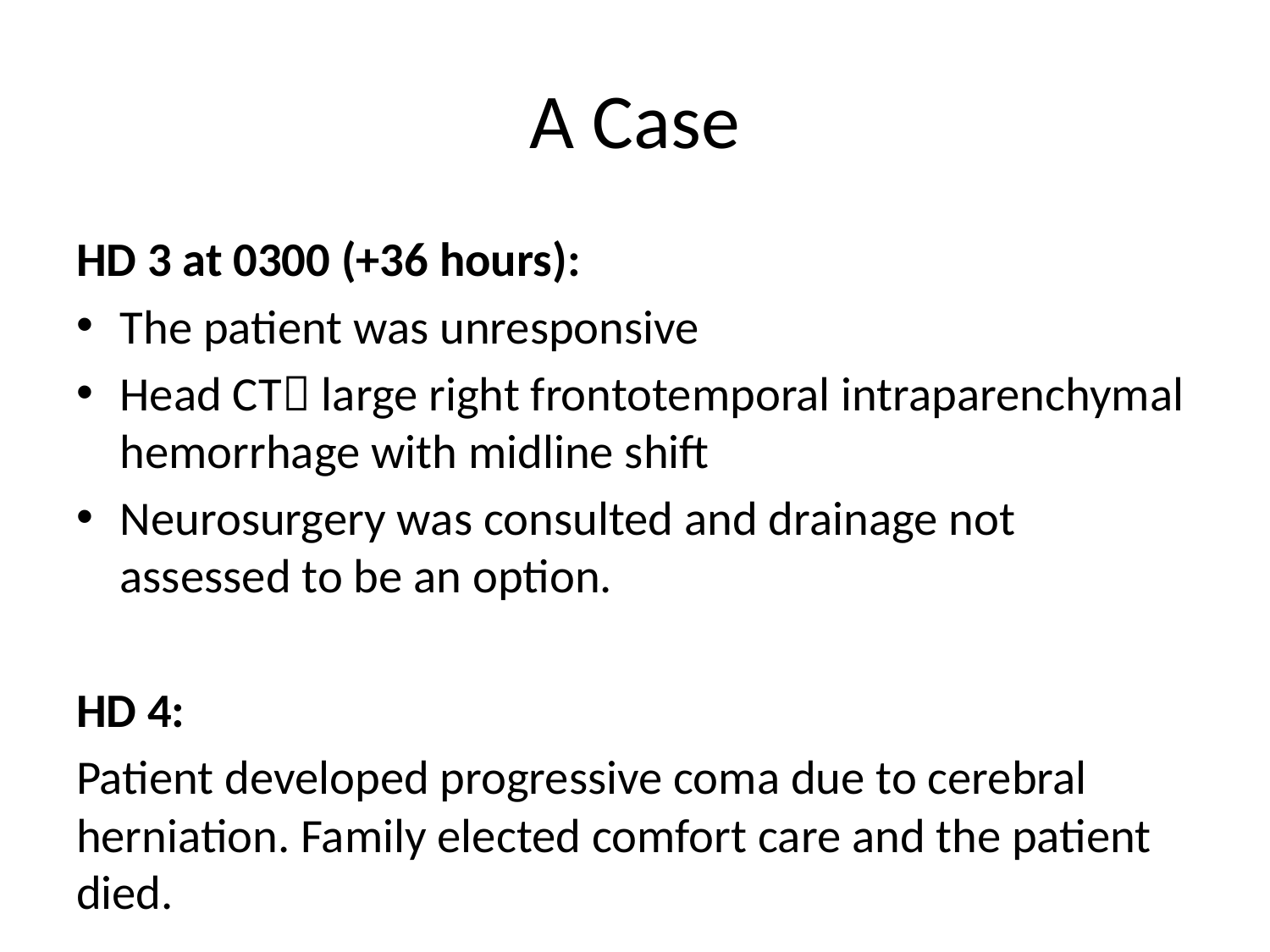

# A Case
HD 3 at 0300 (+36 hours):
The patient was unresponsive
Head CT large right frontotemporal intraparenchymal hemorrhage with midline shift
Neurosurgery was consulted and drainage not assessed to be an option.
HD 4:
Patient developed progressive coma due to cerebral herniation. Family elected comfort care and the patient died.

## Slide 7
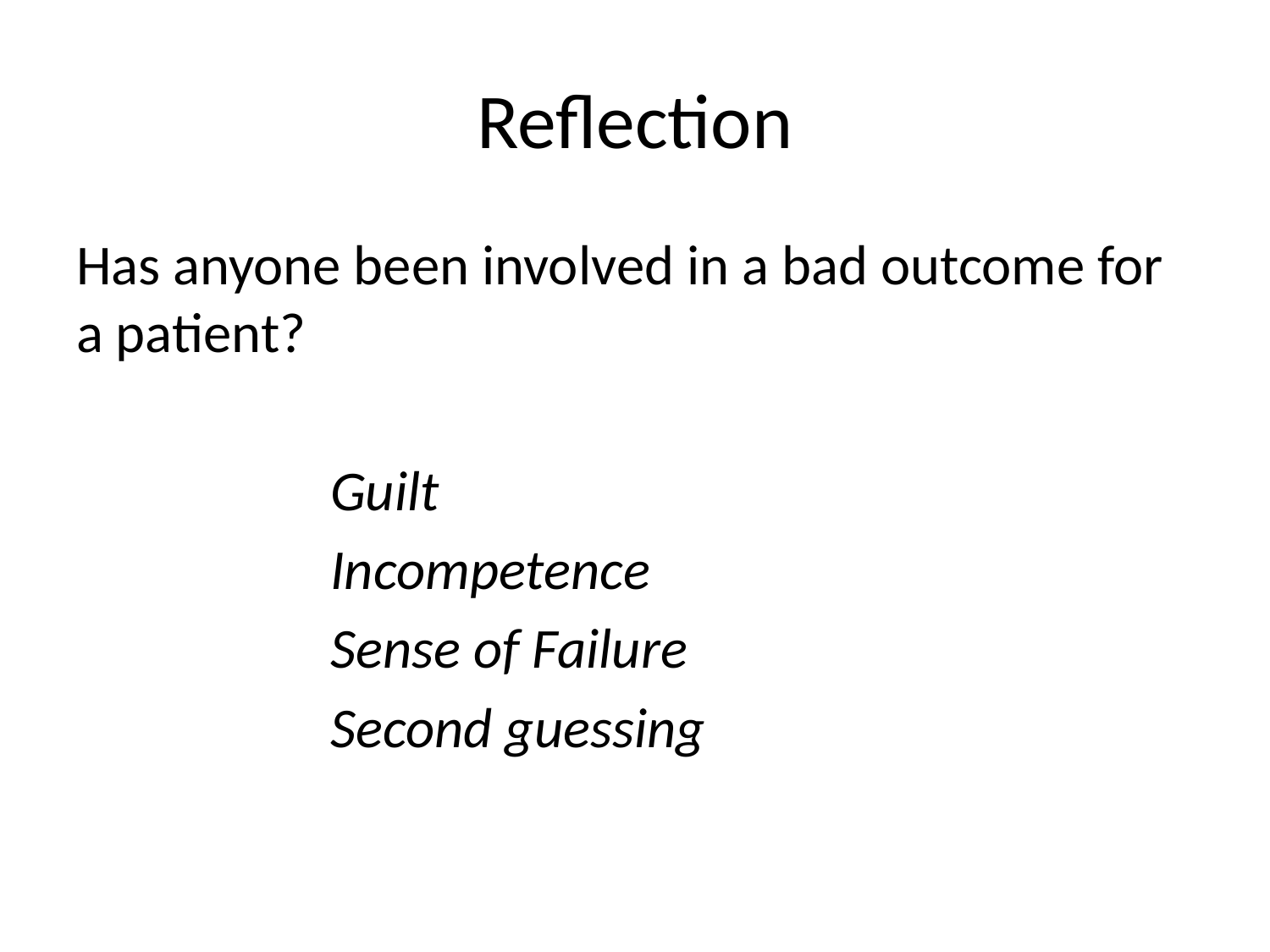

# Reflection
Has anyone been involved in a bad outcome for a patient?
		Guilt
		Incompetence
		Sense of Failure
		Second guessing

## Slide 8
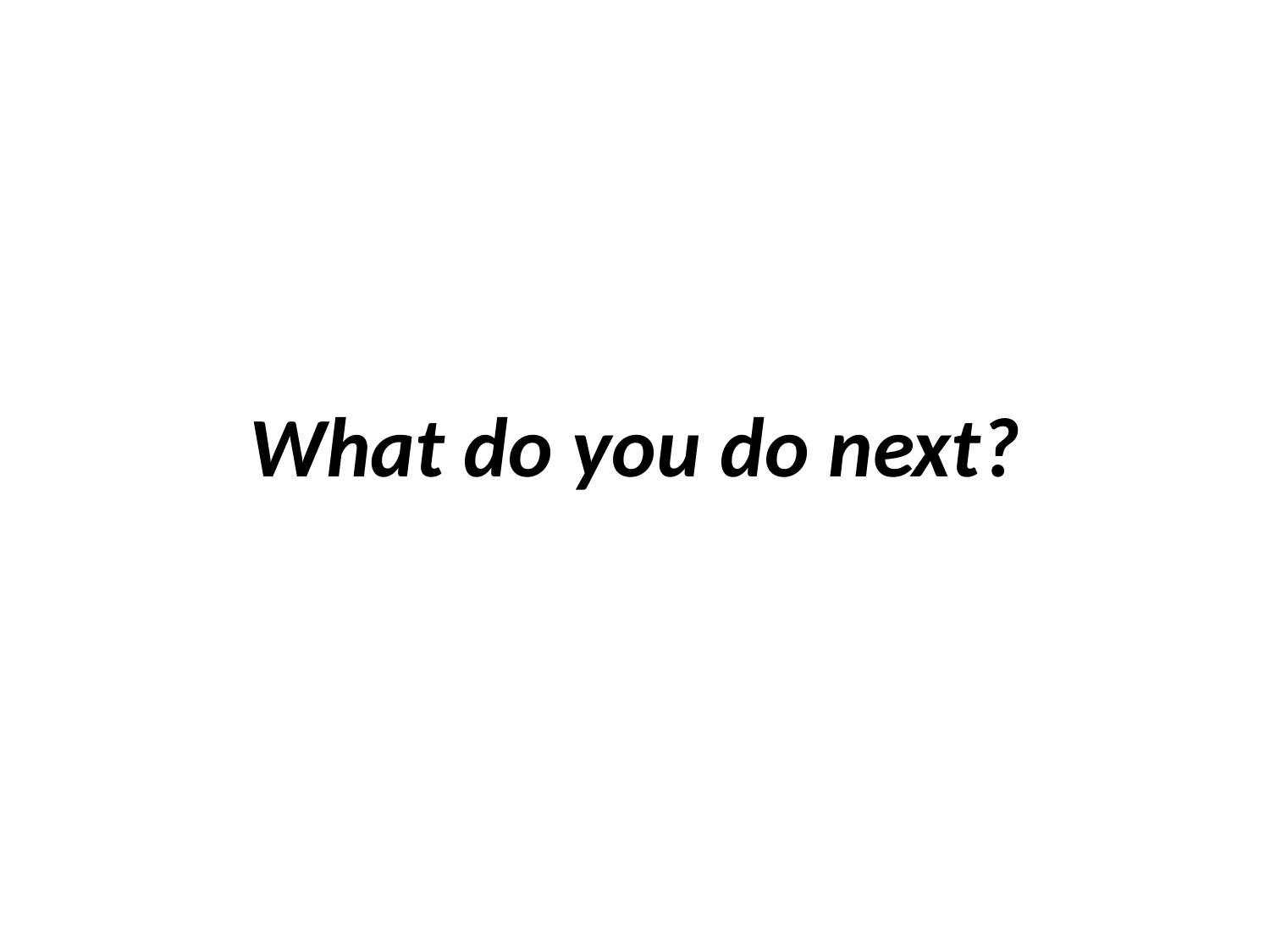

#
What do you do next?

## Slide 9
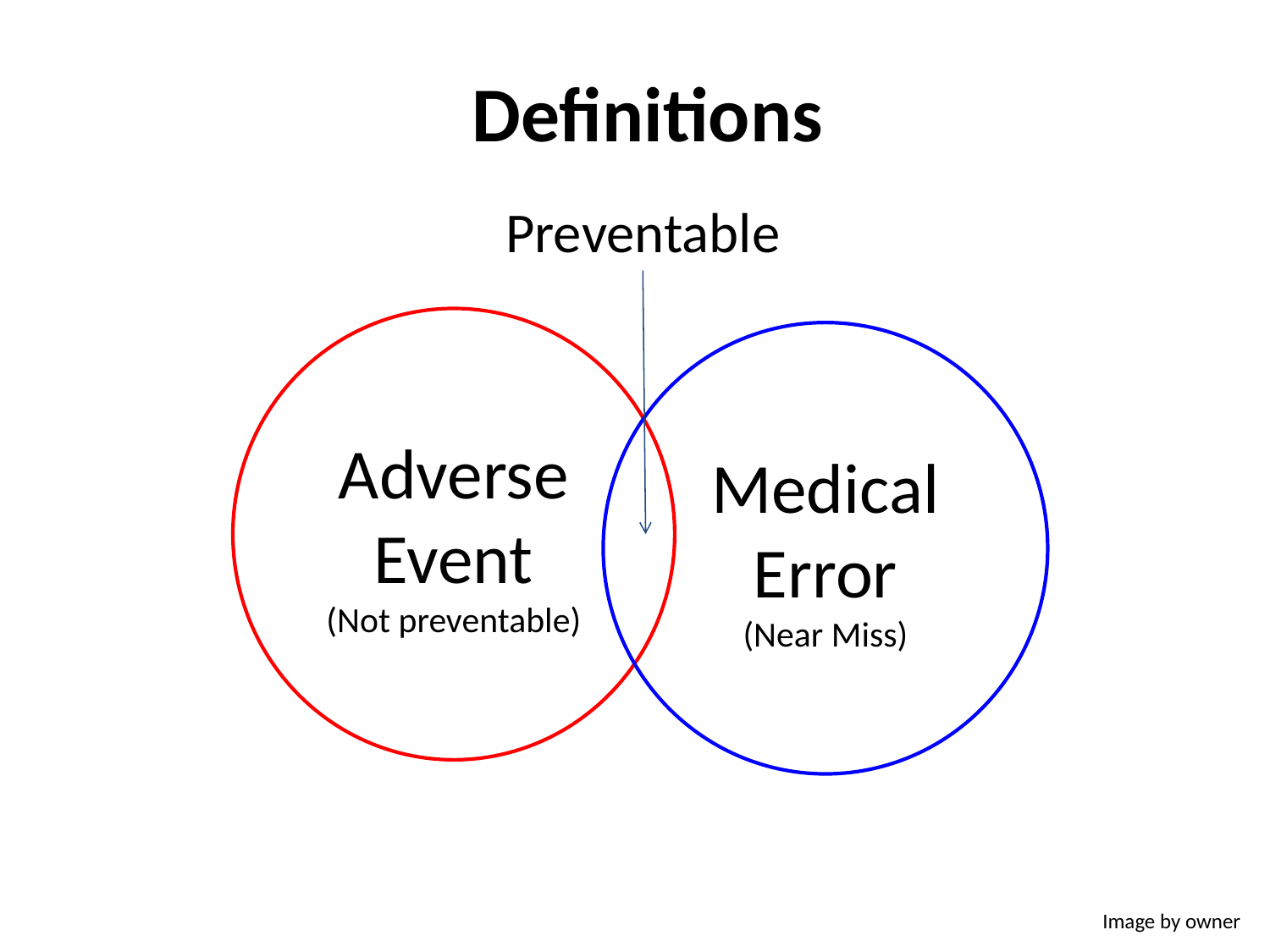

Definitions
Preventable
Adverse Event
(Not preventable)
Medical Error
(Near Miss)
Image by owner

## Slide 10
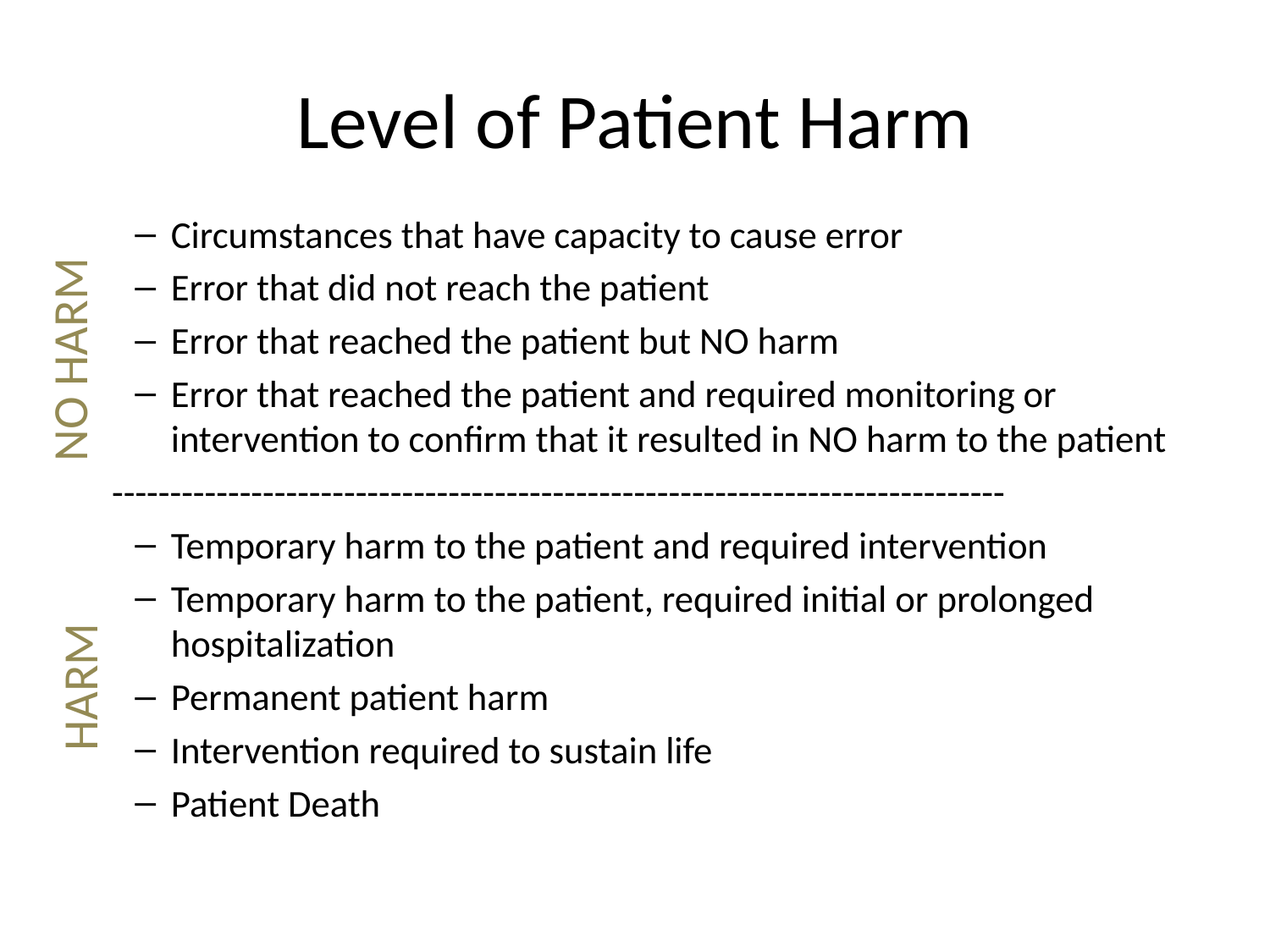

# Level of Patient Harm
Circumstances that have capacity to cause error
Error that did not reach the patient
Error that reached the patient but NO harm
Error that reached the patient and required monitoring or intervention to confirm that it resulted in NO harm to the patient
-----------------------------------------------------------------------------
Temporary harm to the patient and required intervention
Temporary harm to the patient, required initial or prolonged hospitalization
Permanent patient harm
Intervention required to sustain life
Patient Death
NO HARM
 HARM

## Slide 11
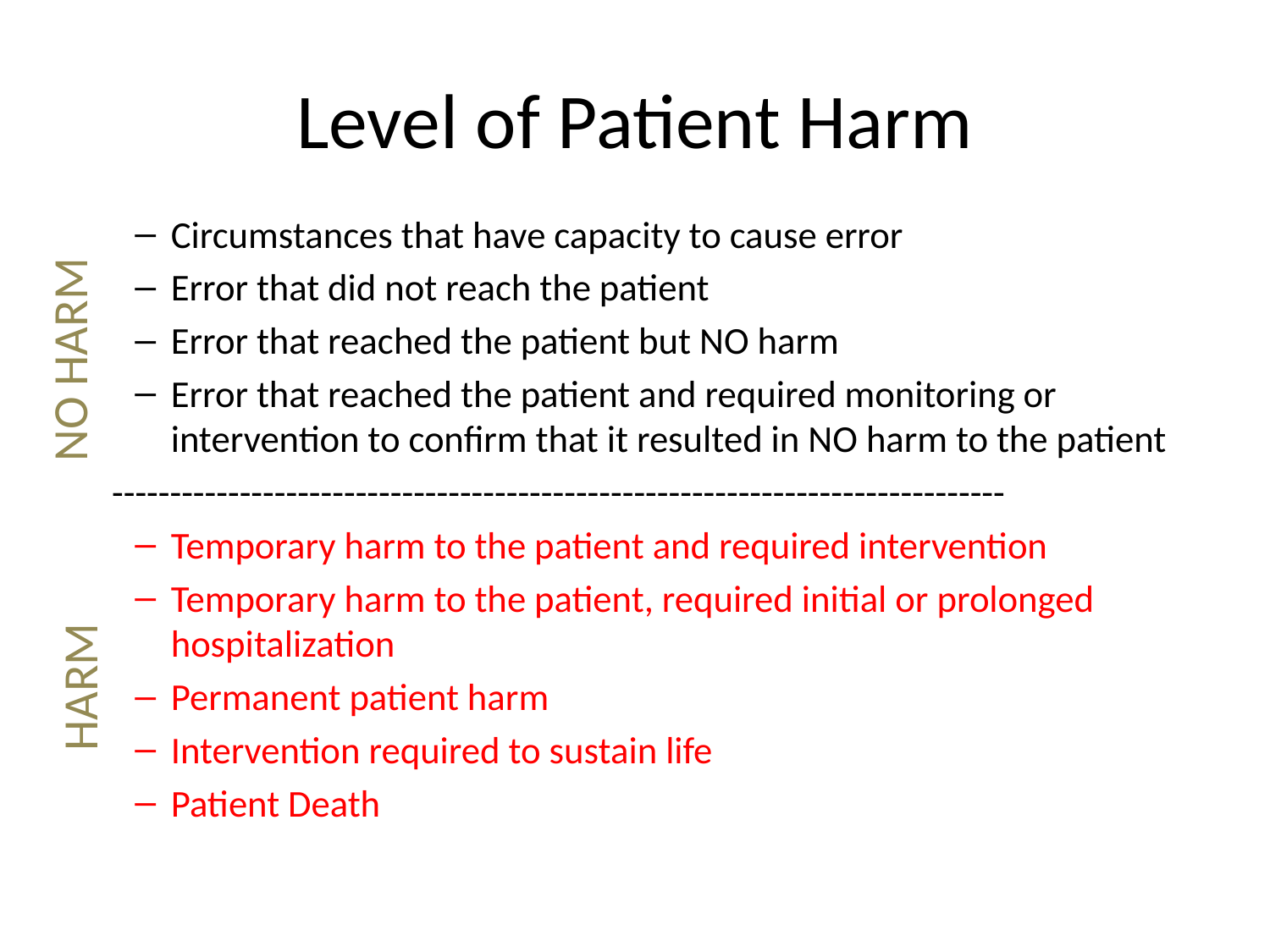

# Level of Patient Harm
Circumstances that have capacity to cause error
Error that did not reach the patient
Error that reached the patient but NO harm
Error that reached the patient and required monitoring or intervention to confirm that it resulted in NO harm to the patient
-----------------------------------------------------------------------------
Temporary harm to the patient and required intervention
Temporary harm to the patient, required initial or prolonged hospitalization
Permanent patient harm
Intervention required to sustain life
Patient Death
NO HARM
 HARM

## Slide 12
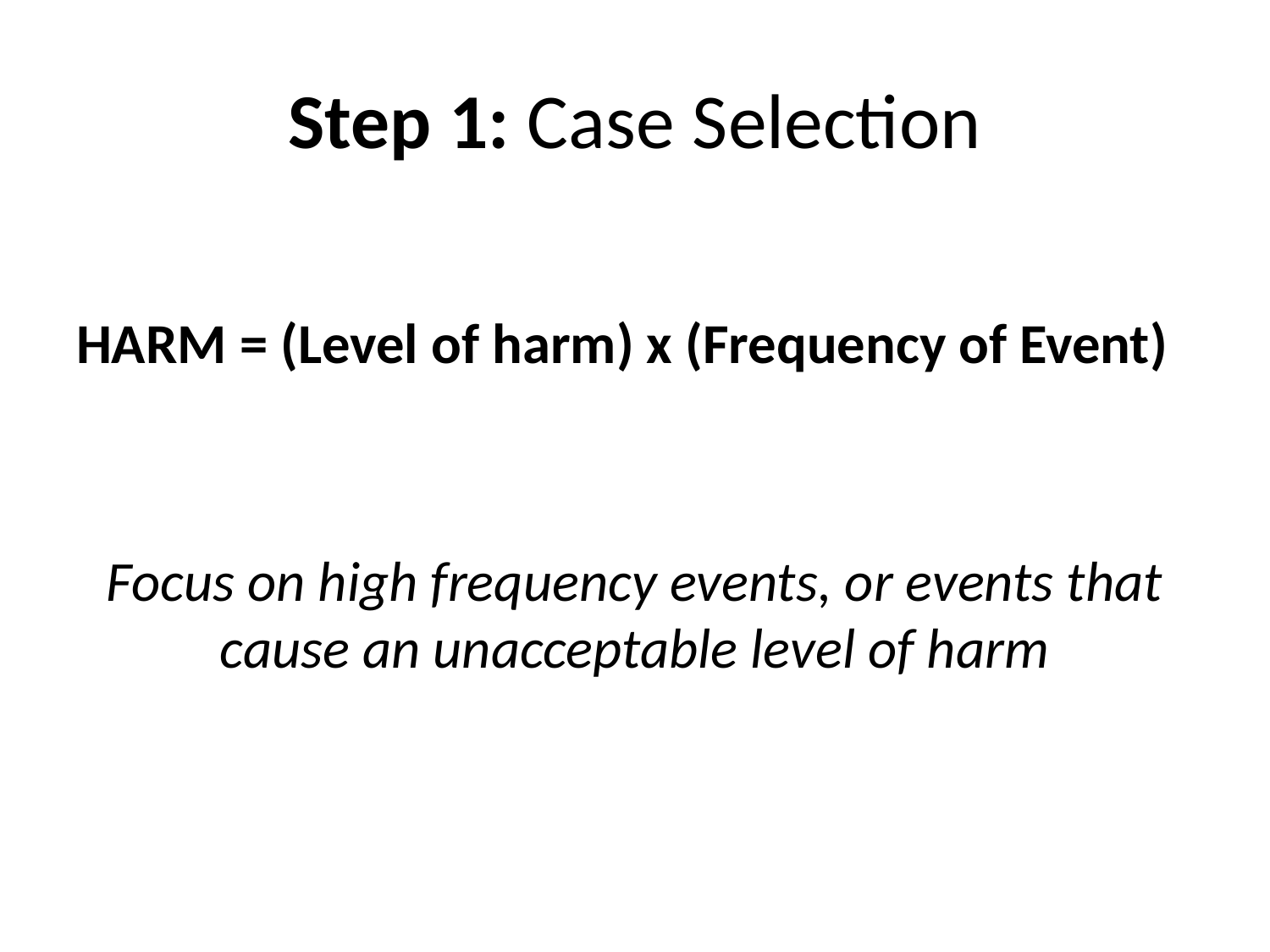

# Step 1: Case Selection
HARM = (Level of harm) x (Frequency of Event)
Focus on high frequency events, or events that cause an unacceptable level of harm

## Slide 13
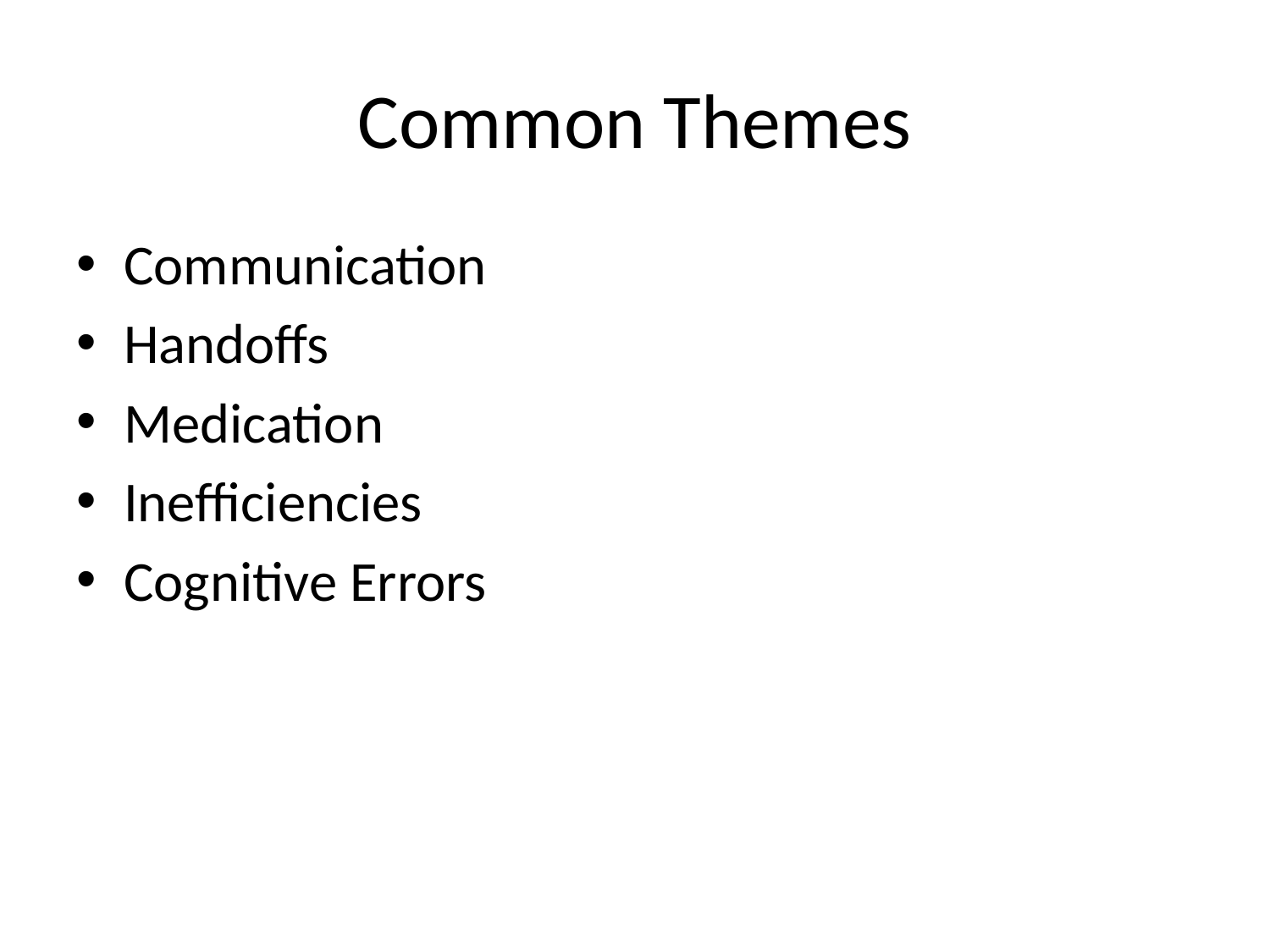

# Common Themes
Communication
Handoffs
Medication
Inefficiencies
Cognitive Errors

## Slide 14
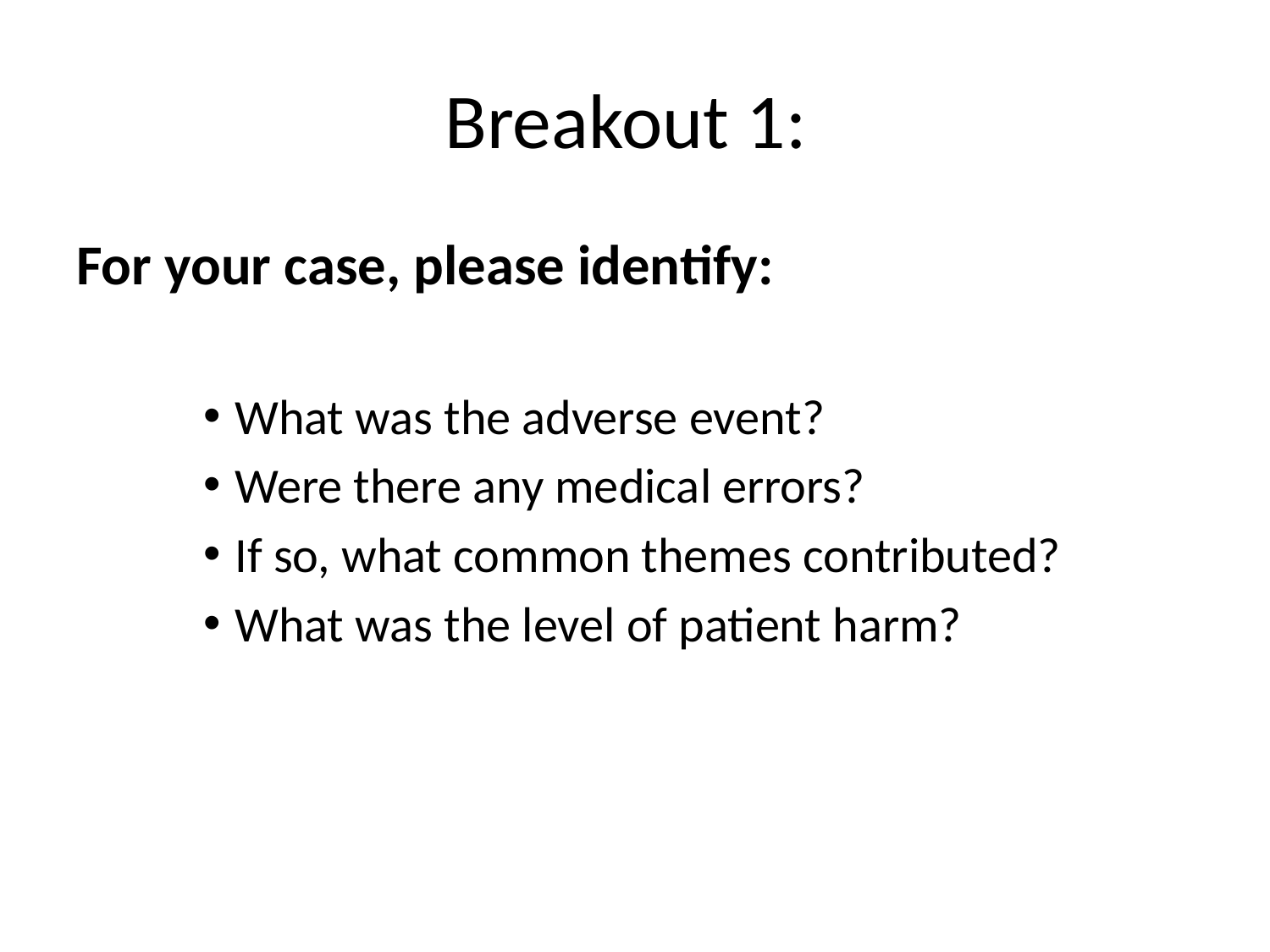

# Breakout 1:
For your case, please identify:
What was the adverse event?
Were there any medical errors?
If so, what common themes contributed?
What was the level of patient harm?

## Slide 15
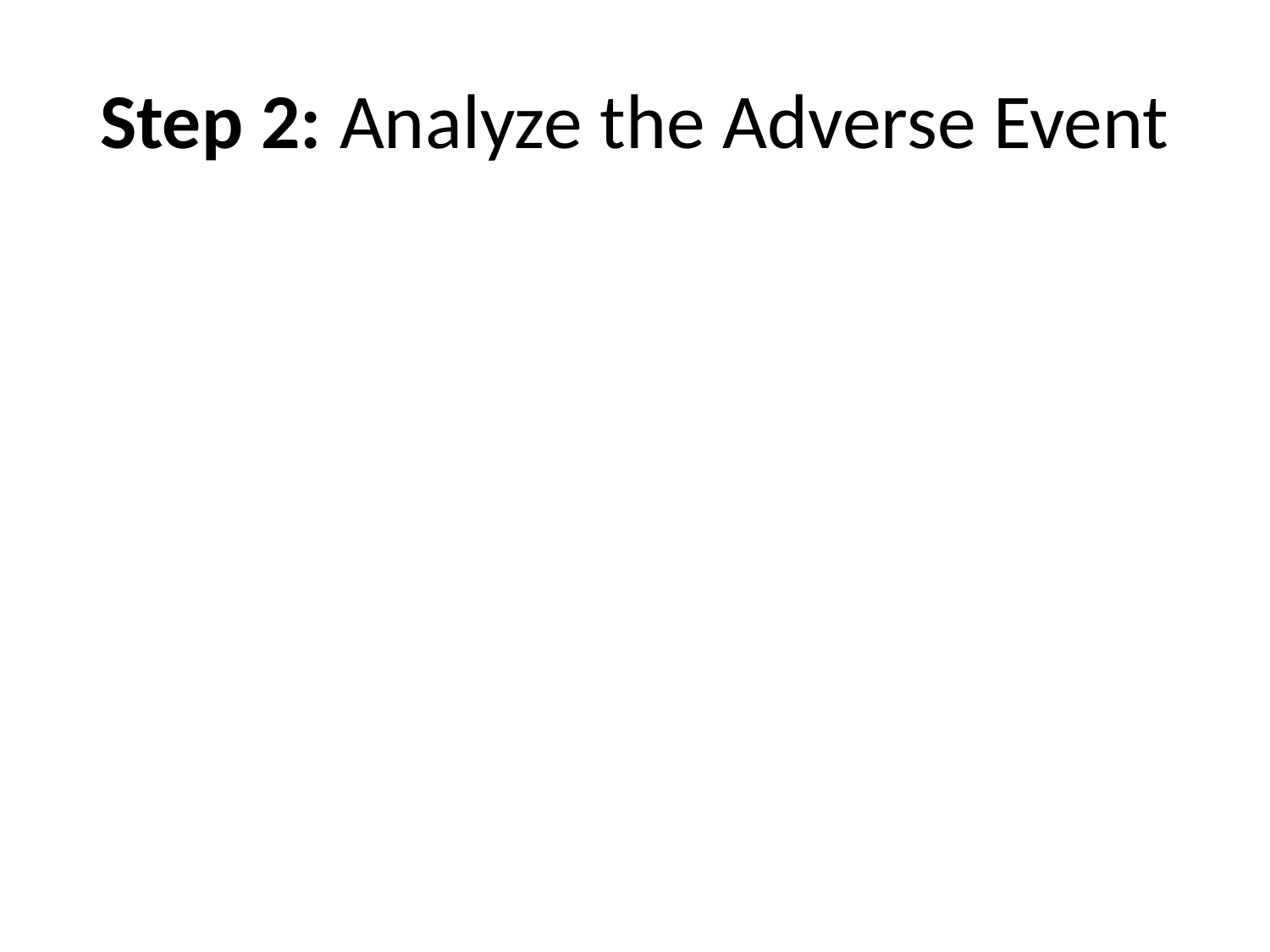

# Step 2: Analyze the Adverse Event

## Slide 16
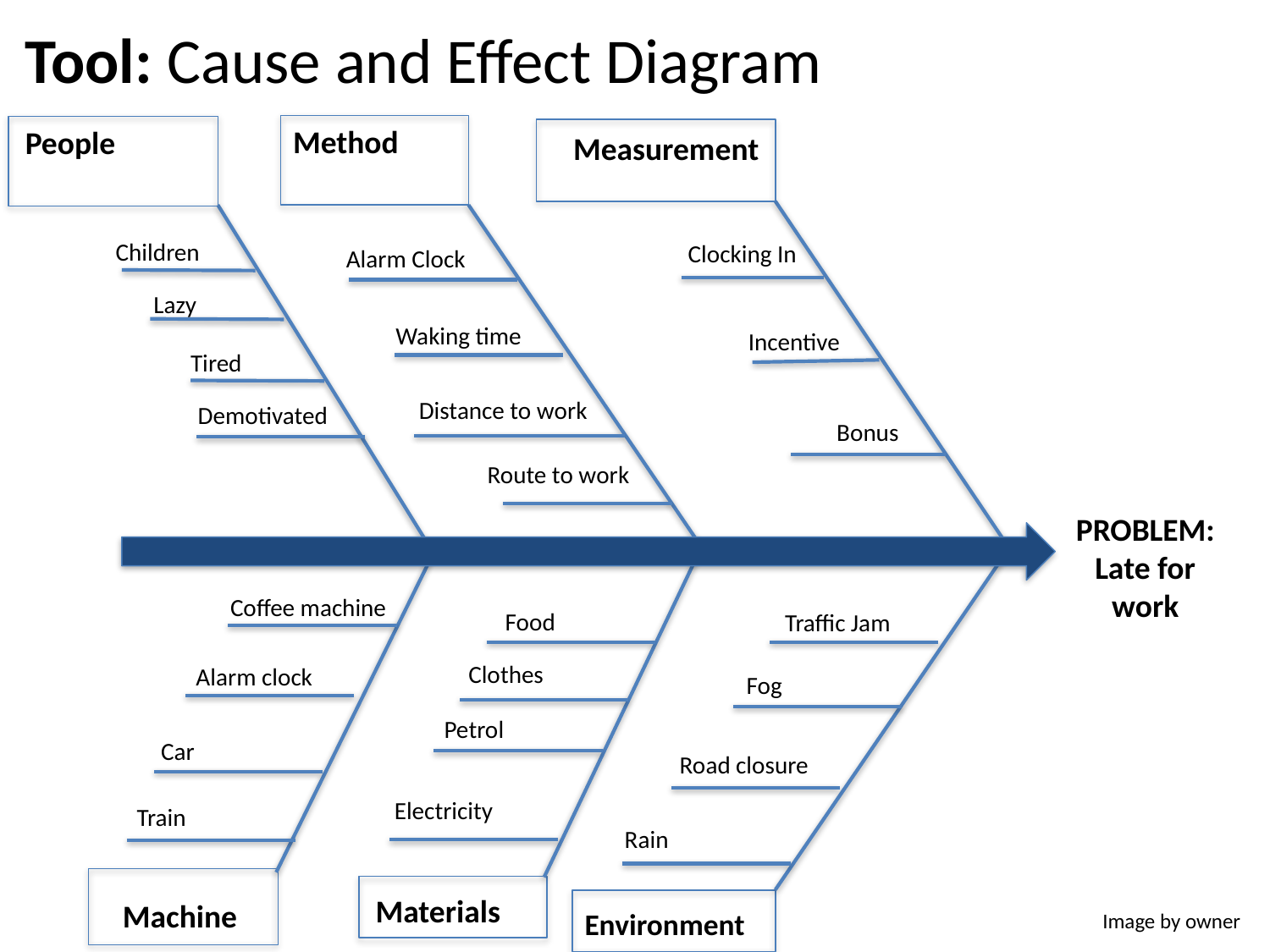

Tool: Cause and Effect Diagram
Method
People
Measurement
Children
Clocking In
Alarm Clock
Lazy
Waking time
Incentive
Tired
Distance to work
Demotivated
Bonus
Route to work
PROBLEM:
Late for work
Coffee machine
Food
Traffic Jam
Clothes
Alarm clock
Fog
Petrol
Car
Road closure
Electricity
Train
Rain
Materials
Machine
Environment
Image by owner

## Slide 17
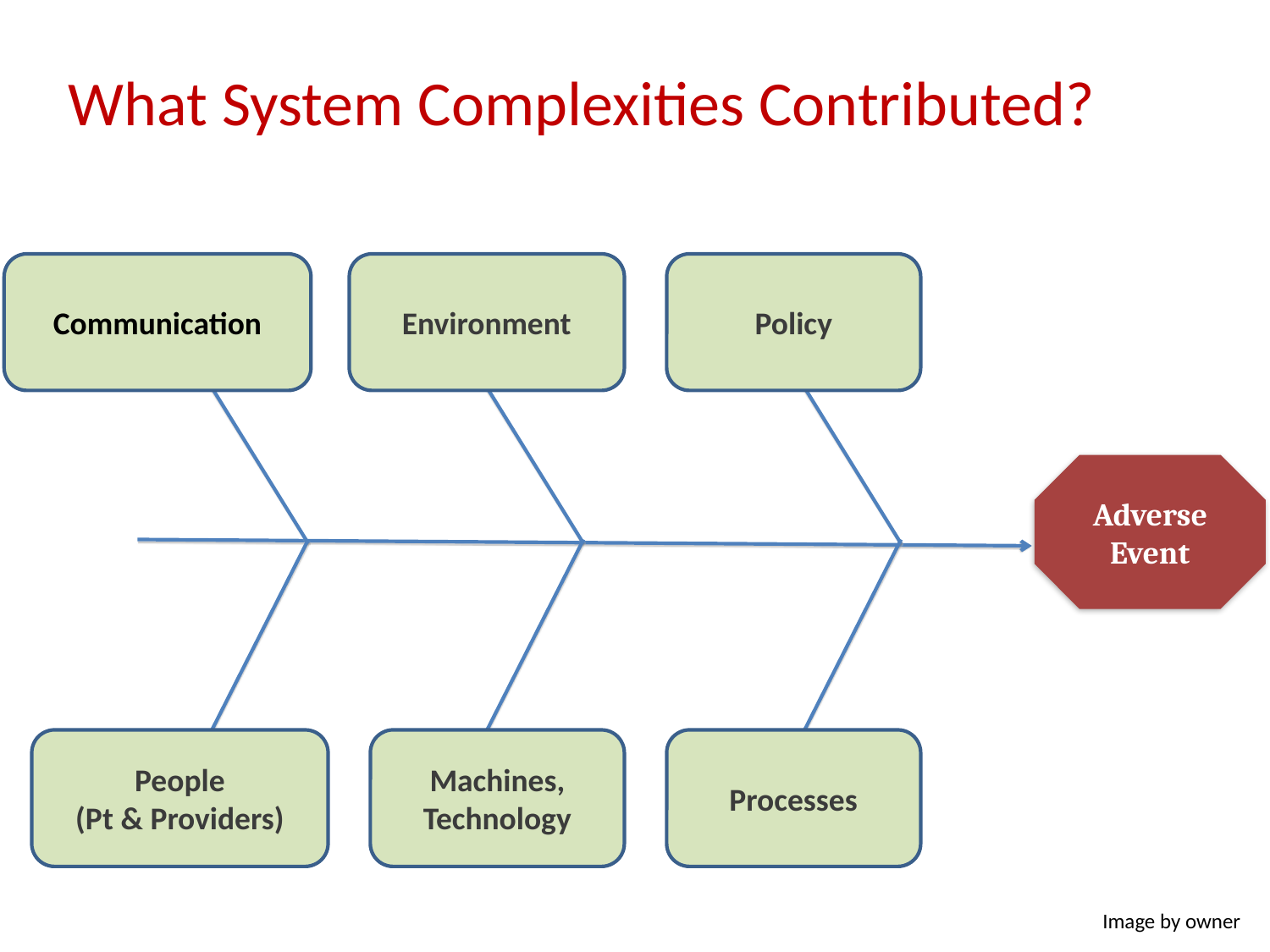

# What System Complexities Contributed?
Communication
Environment
Policy
Adverse Event
People
(Pt & Providers)
Machines, Technology
Processes
Image by owner

## Slide 18
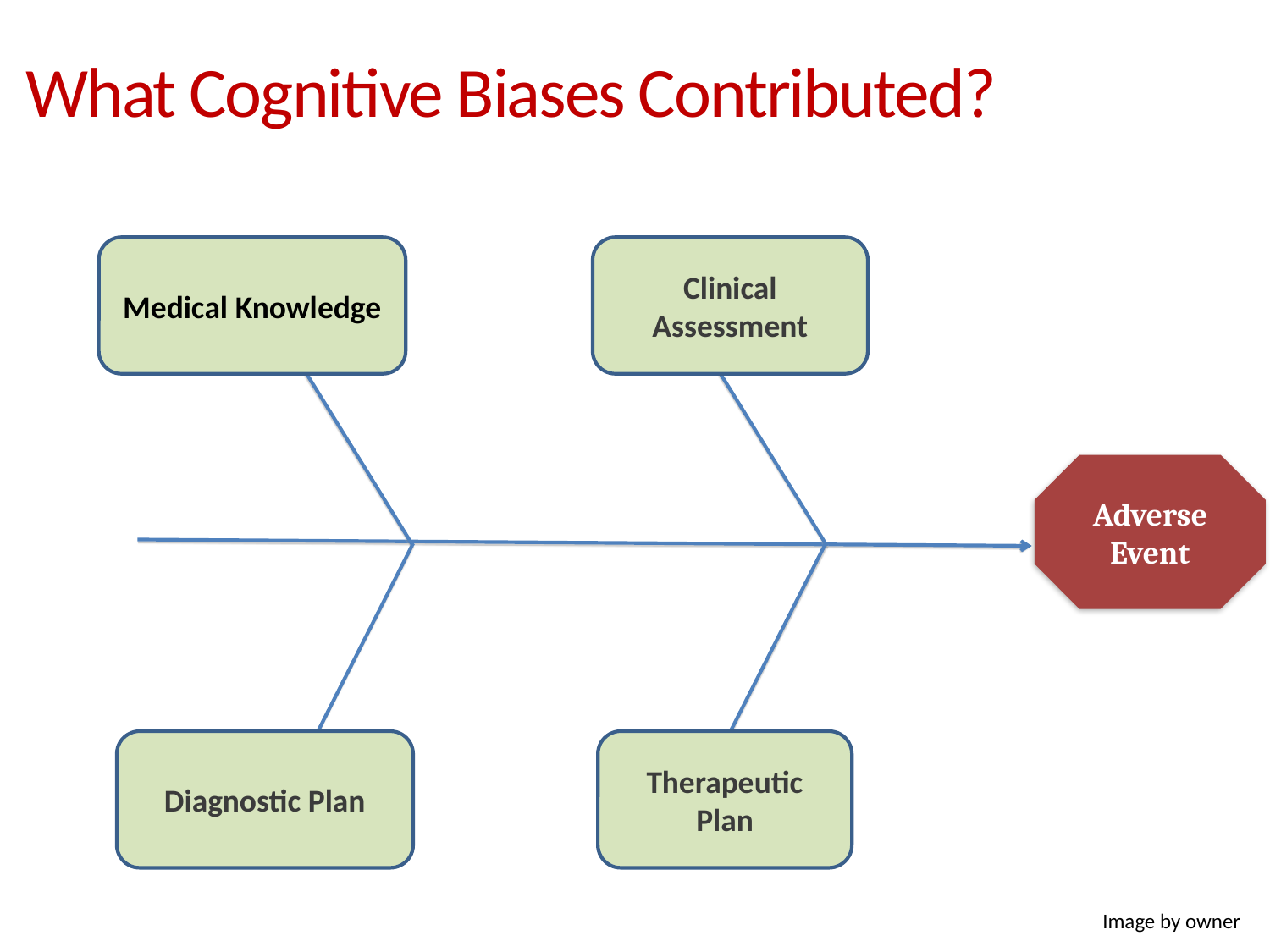

What Cognitive Biases Contributed?
Medical Knowledge
Clinical Assessment
Adverse Event
Diagnostic Plan
Therapeutic Plan
Image by owner

## Slide 19
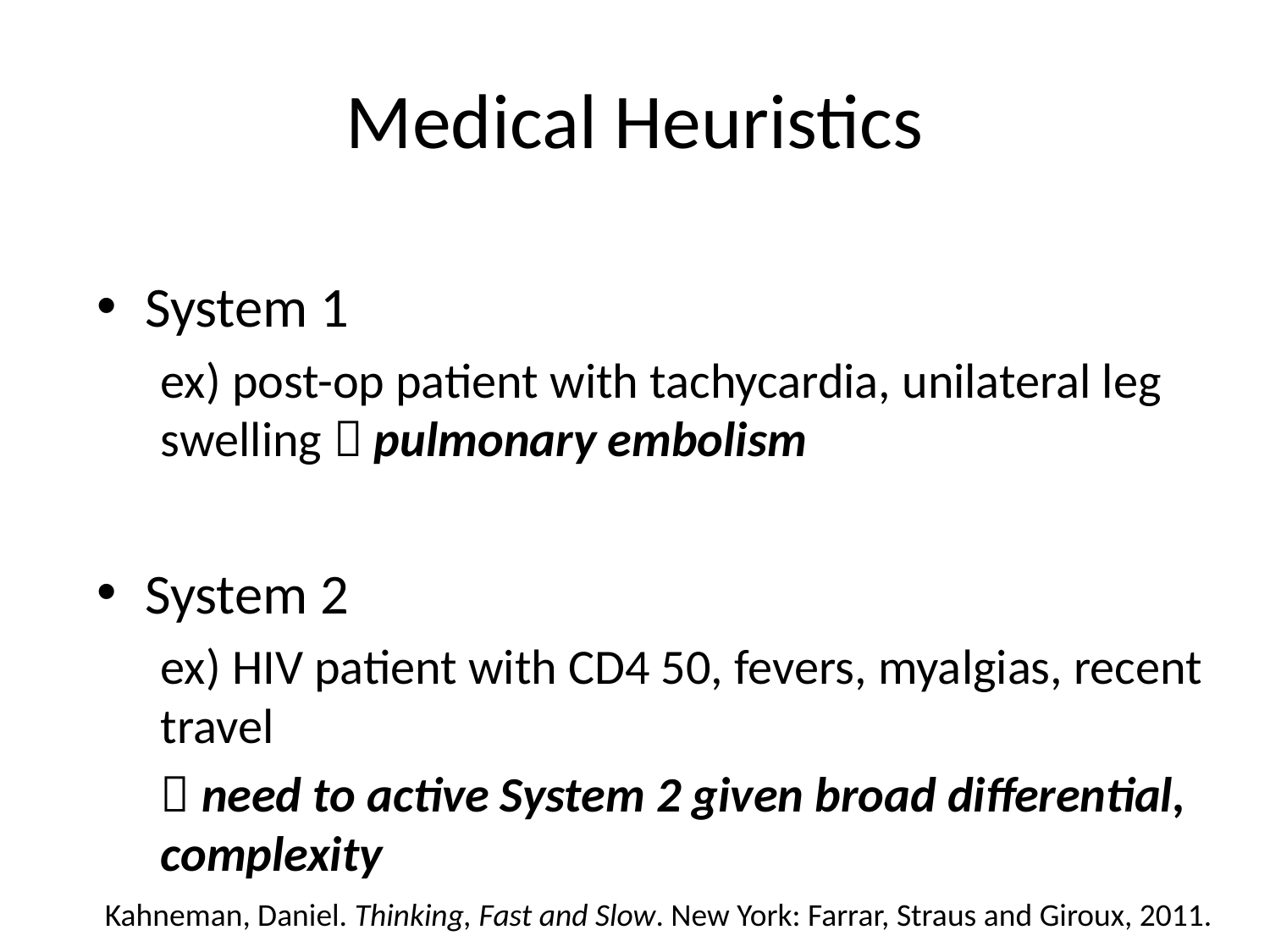

# Medical Heuristics
System 1
ex) post-op patient with tachycardia, unilateral leg swelling  pulmonary embolism
System 2
ex) HIV patient with CD4 50, fevers, myalgias, recent travel
 need to active System 2 given broad differential, complexity
 Kahneman, Daniel. Thinking, Fast and Slow. New York: Farrar, Straus and Giroux, 2011.

## Slide 20
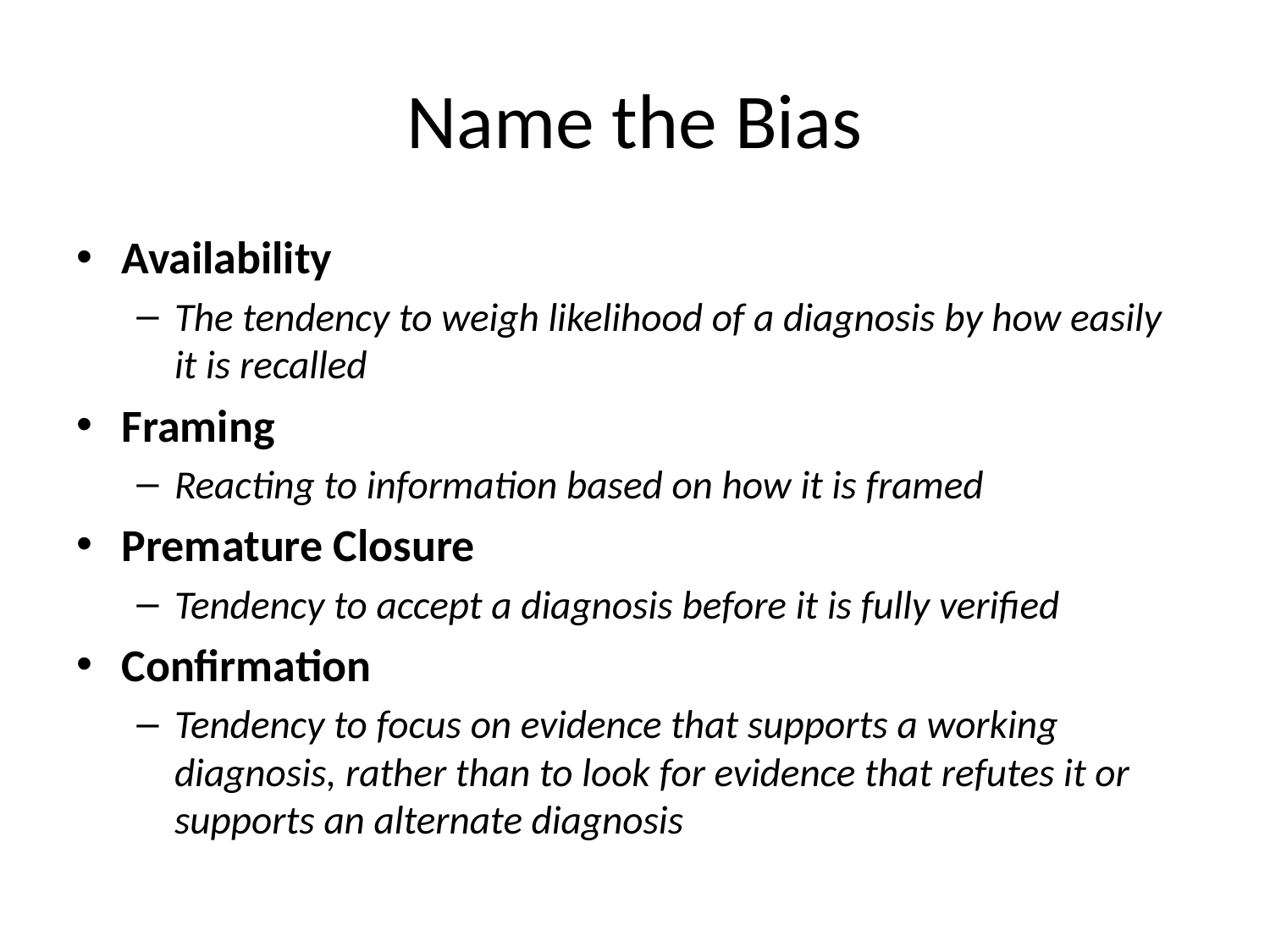

# Name the Bias
Availability
The tendency to weigh likelihood of a diagnosis by how easily it is recalled
Framing
Reacting to information based on how it is framed
Premature Closure
Tendency to accept a diagnosis before it is fully verified
Confirmation
Tendency to focus on evidence that supports a working diagnosis, rather than to look for evidence that refutes it or supports an alternate diagnosis

## Slide 21
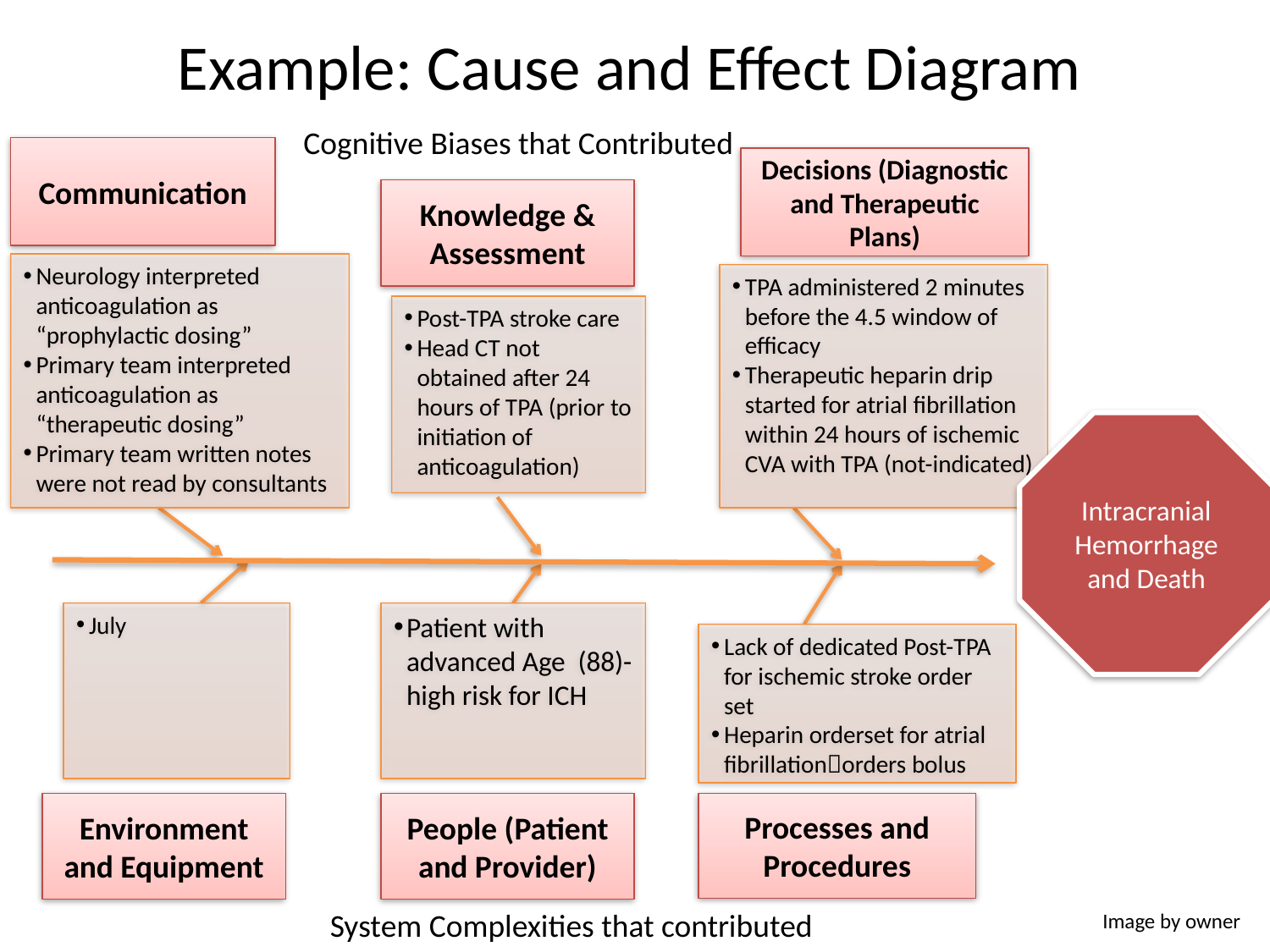

# Example: Cause and Effect Diagram
Cognitive Biases that Contributed
Communication
Decisions (Diagnostic and Therapeutic Plans)
Knowledge & Assessment
Neurology interpreted anticoagulation as “prophylactic dosing”
Primary team interpreted anticoagulation as “therapeutic dosing”
Primary team written notes were not read by consultants
TPA administered 2 minutes before the 4.5 window of efficacy
Therapeutic heparin drip started for atrial fibrillation within 24 hours of ischemic CVA with TPA (not-indicated)
Post-TPA stroke care
Head CT not obtained after 24 hours of TPA (prior to initiation of anticoagulation)
Intracranial Hemorrhage and Death
July
Patient with advanced Age (88)- high risk for ICH
Lack of dedicated Post-TPA for ischemic stroke order set
Heparin orderset for atrial fibrillationorders bolus
Environment and Equipment
People (Patient and Provider)
Processes and Procedures
System Complexities that contributed
Image by owner

## Slide 22
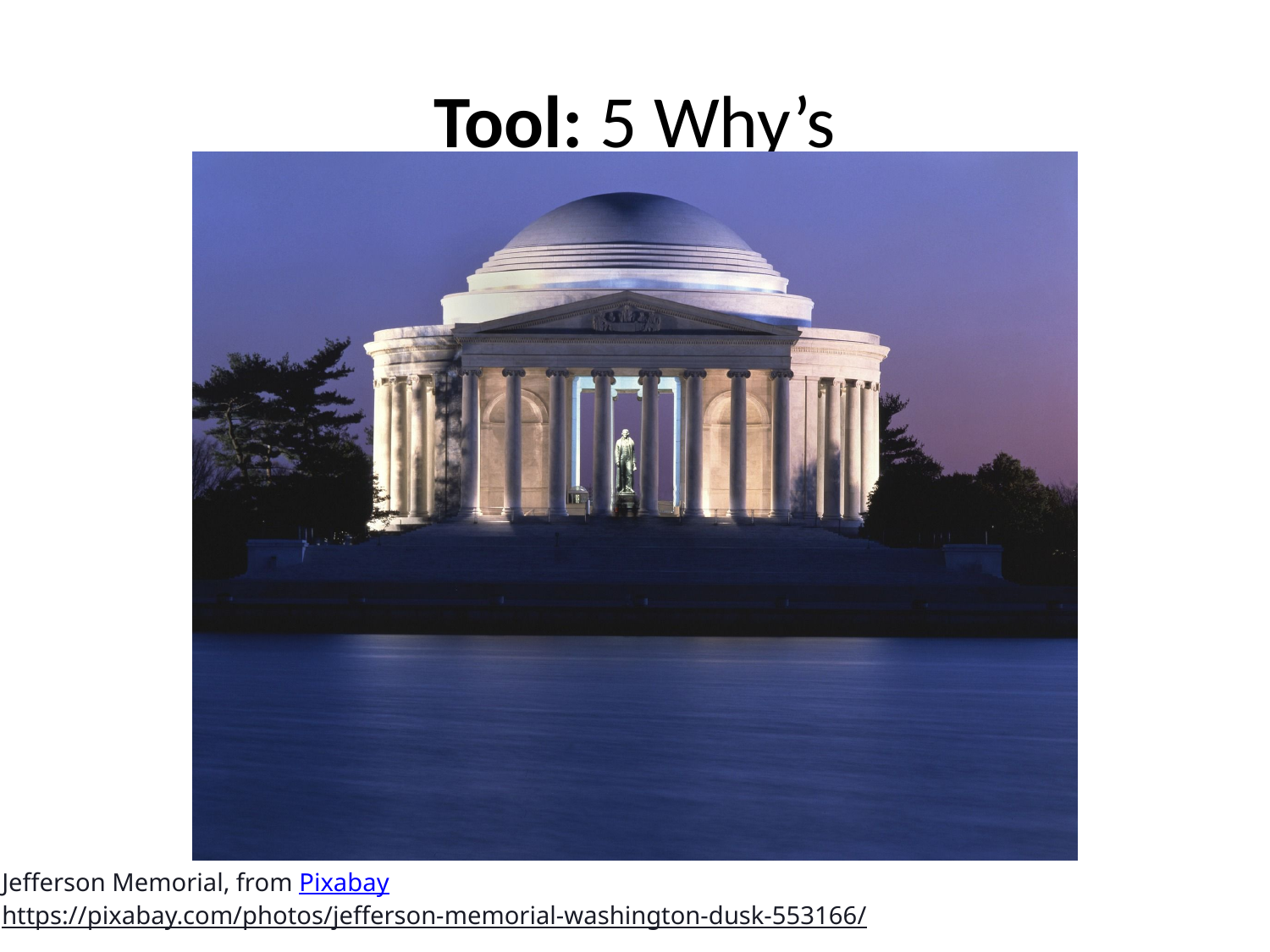

Tool: 5 Why’s
Jefferson Memorial, from Pixabay
https://pixabay.com/photos/jefferson-memorial-washington-dusk-553166/

## Slide 23
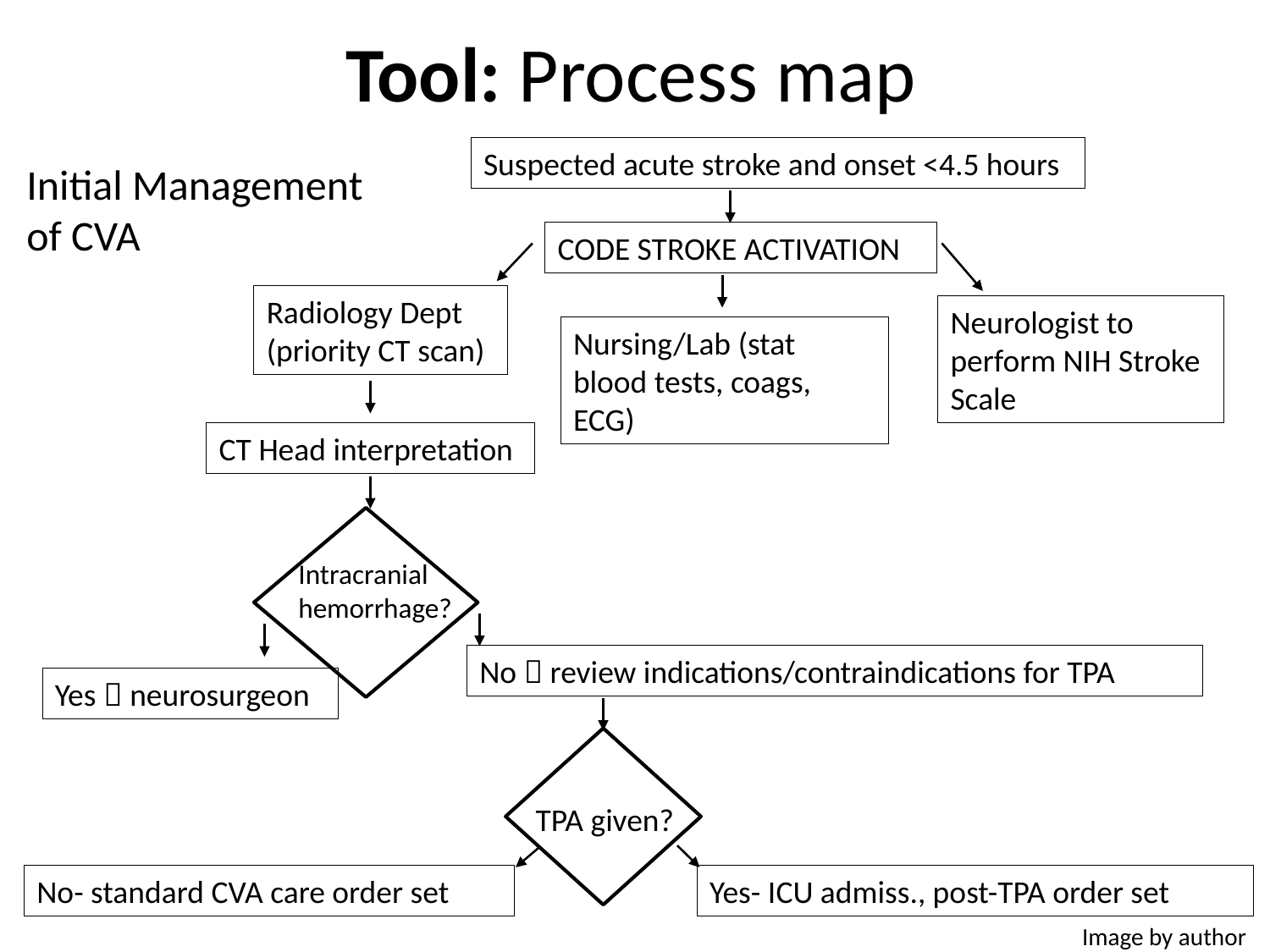

# Tool: Process map
Suspected acute stroke and onset <4.5 hours
Initial Management of CVA
CODE STROKE ACTIVATION
Radiology Dept (priority CT scan)
Neurologist to perform NIH Stroke Scale
Nursing/Lab (stat blood tests, coags, ECG)
CT Head interpretation
Intracranial hemorrhage?
No  review indications/contraindications for TPA
Yes  neurosurgeon
TPA given?
No- standard CVA care order set
Yes- ICU admiss., post-TPA order set
Image by author

## Slide 24
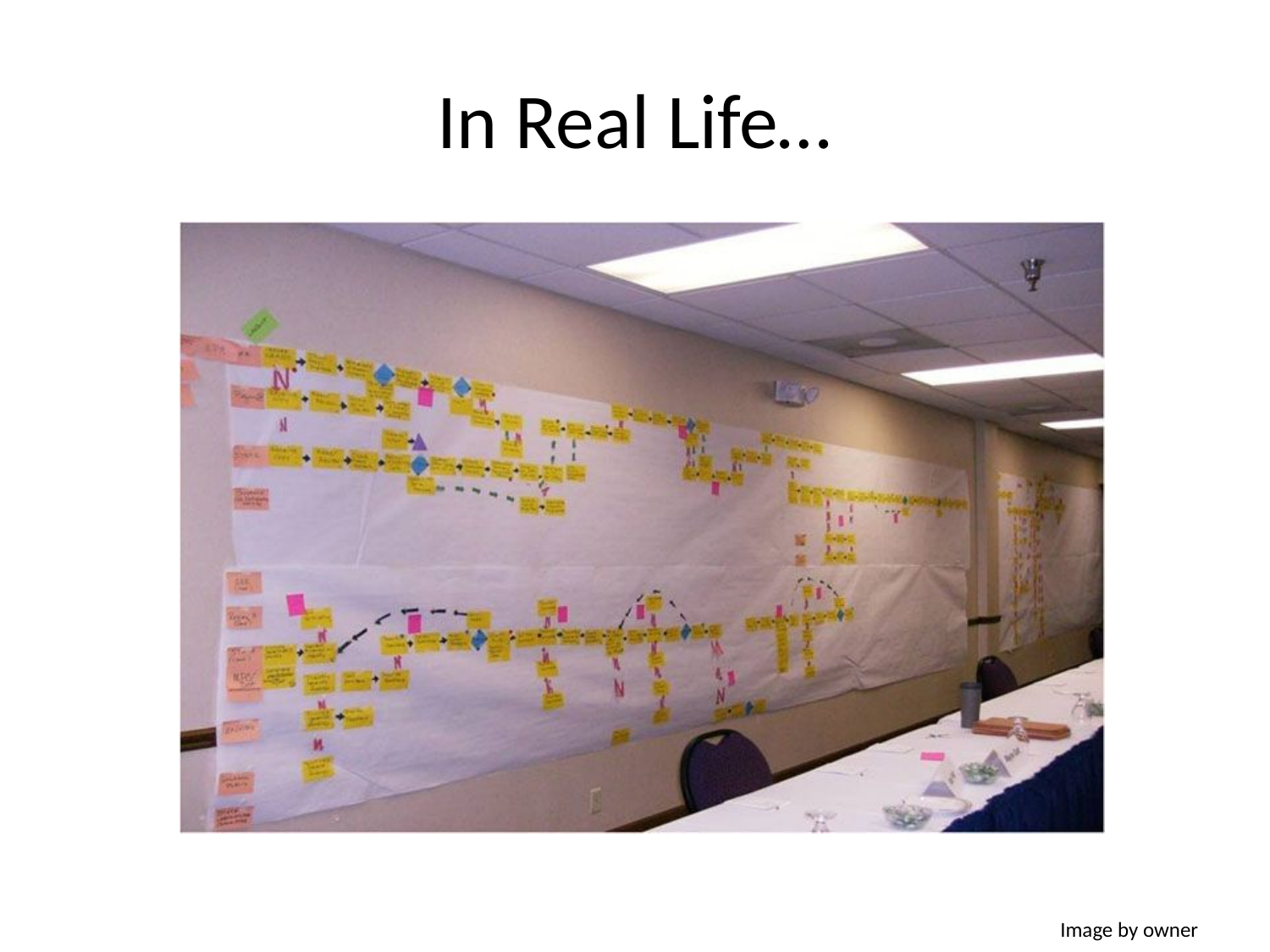

# In Real Life…
Image by owner

## Slide 25
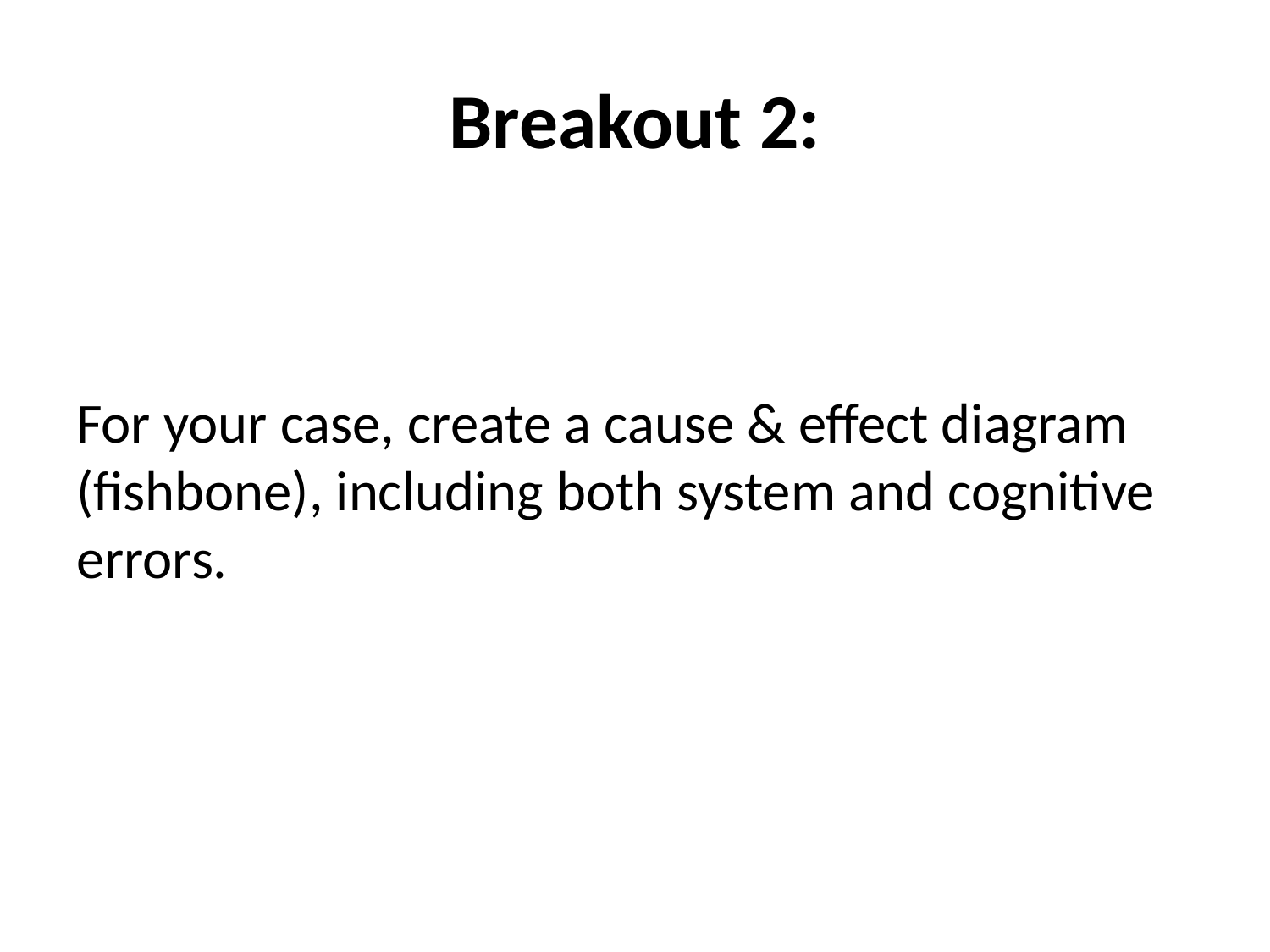

# Breakout 2:
For your case, create a cause & effect diagram (fishbone), including both system and cognitive errors.

## Slide 26
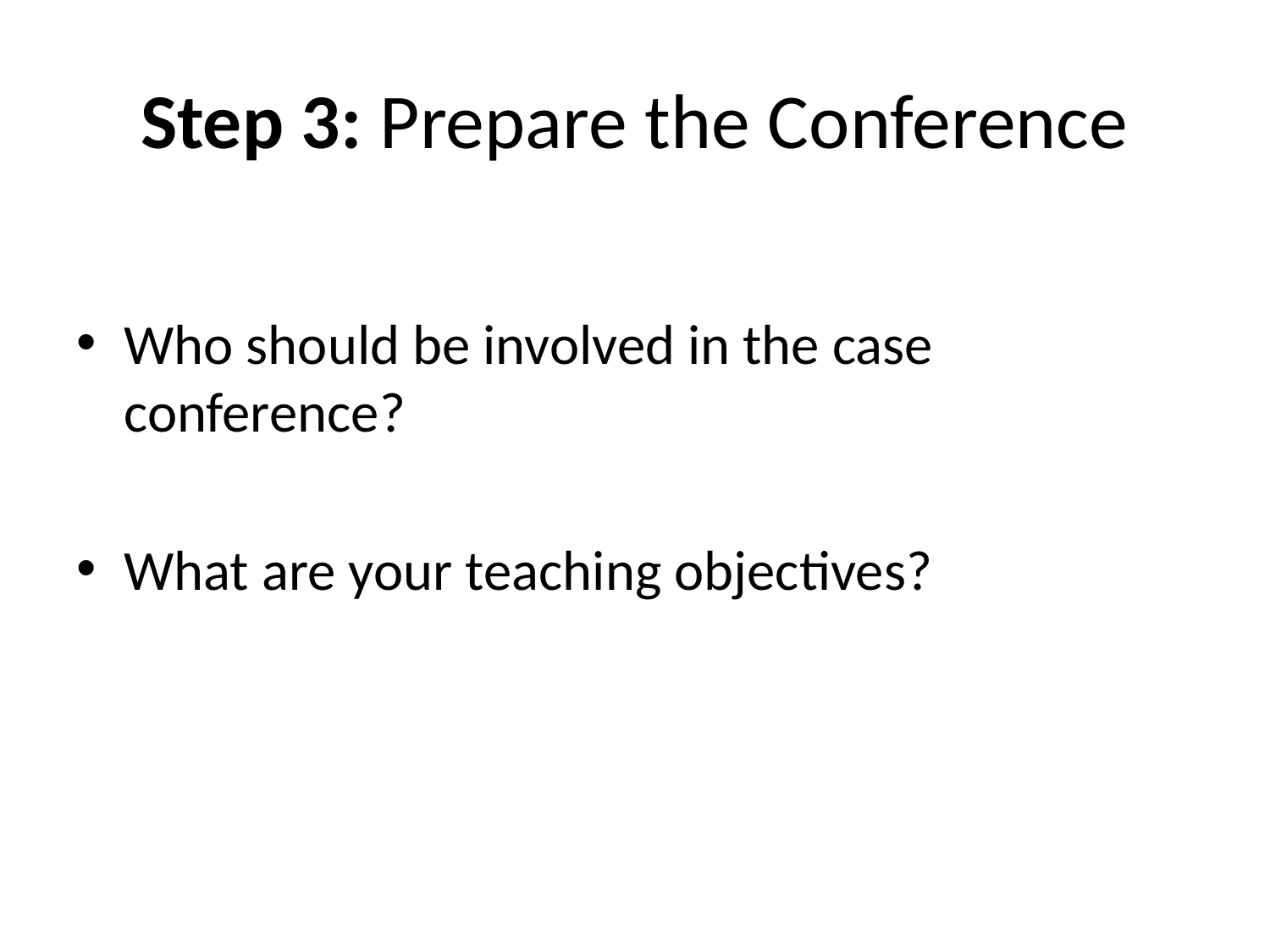

# Step 3: Prepare the Conference
Who should be involved in the case conference?
What are your teaching objectives?

## Slide 27
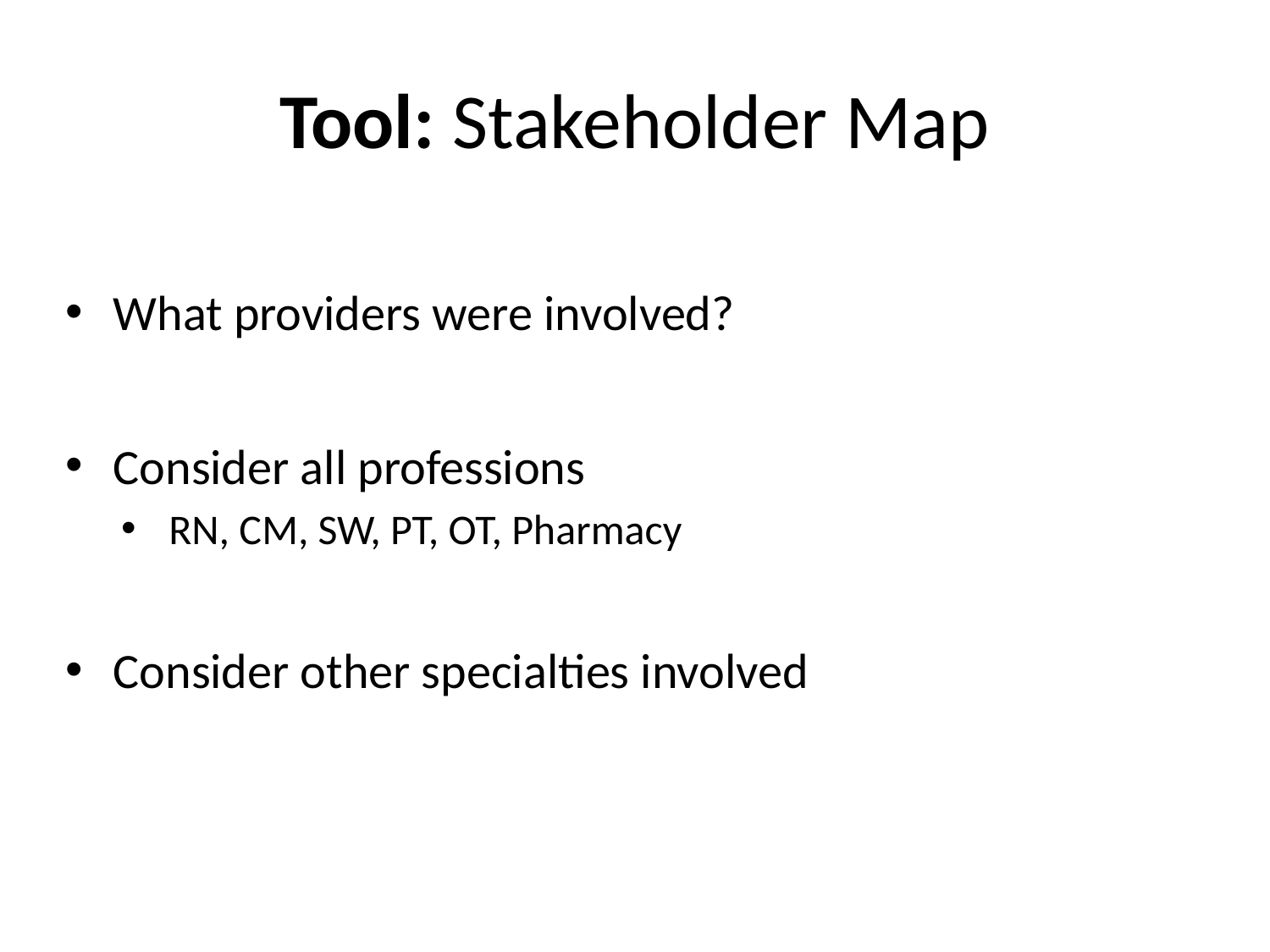

# Tool: Stakeholder Map
What providers were involved?
Consider all professions
RN, CM, SW, PT, OT, Pharmacy
Consider other specialties involved

## Slide 28
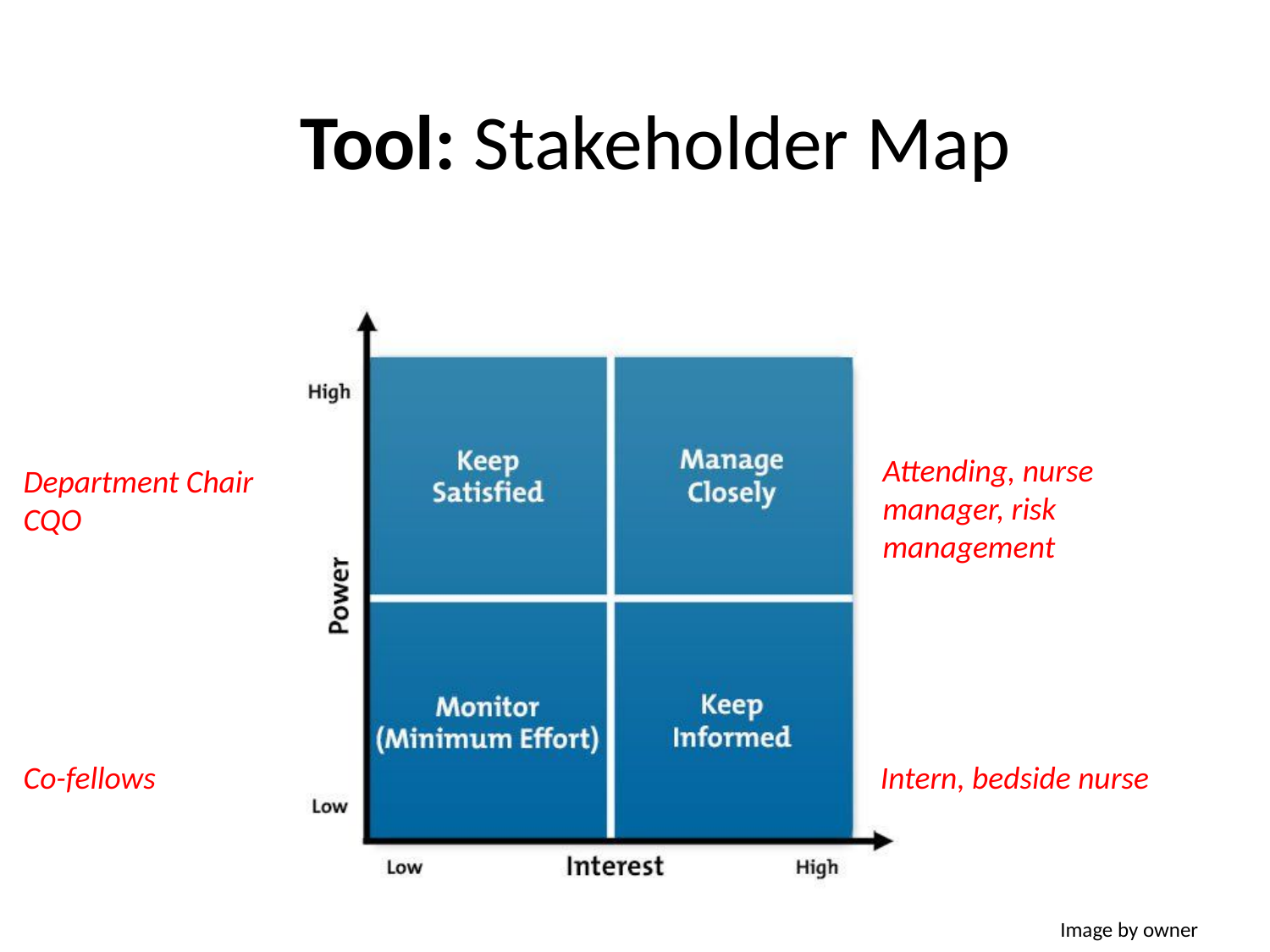

Tool: Stakeholder Map
Attending, nurse manager, risk management
Department Chair
CQO
Co-fellows
Intern, bedside nurse
Image by owner

## Slide 29
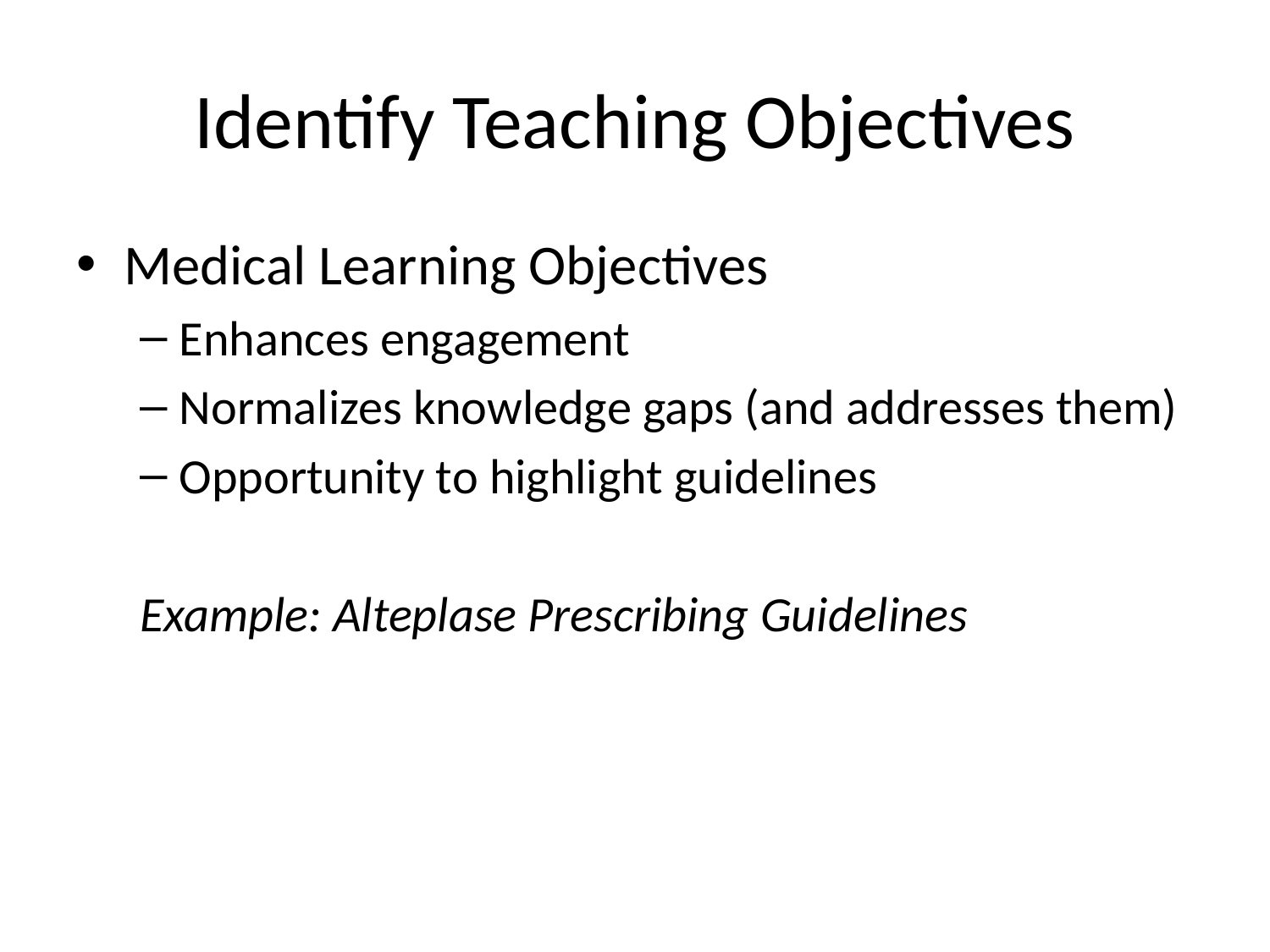

# Identify Teaching Objectives
Medical Learning Objectives
Enhances engagement
Normalizes knowledge gaps (and addresses them)
Opportunity to highlight guidelines
Example: Alteplase Prescribing Guidelines

## Slide 30
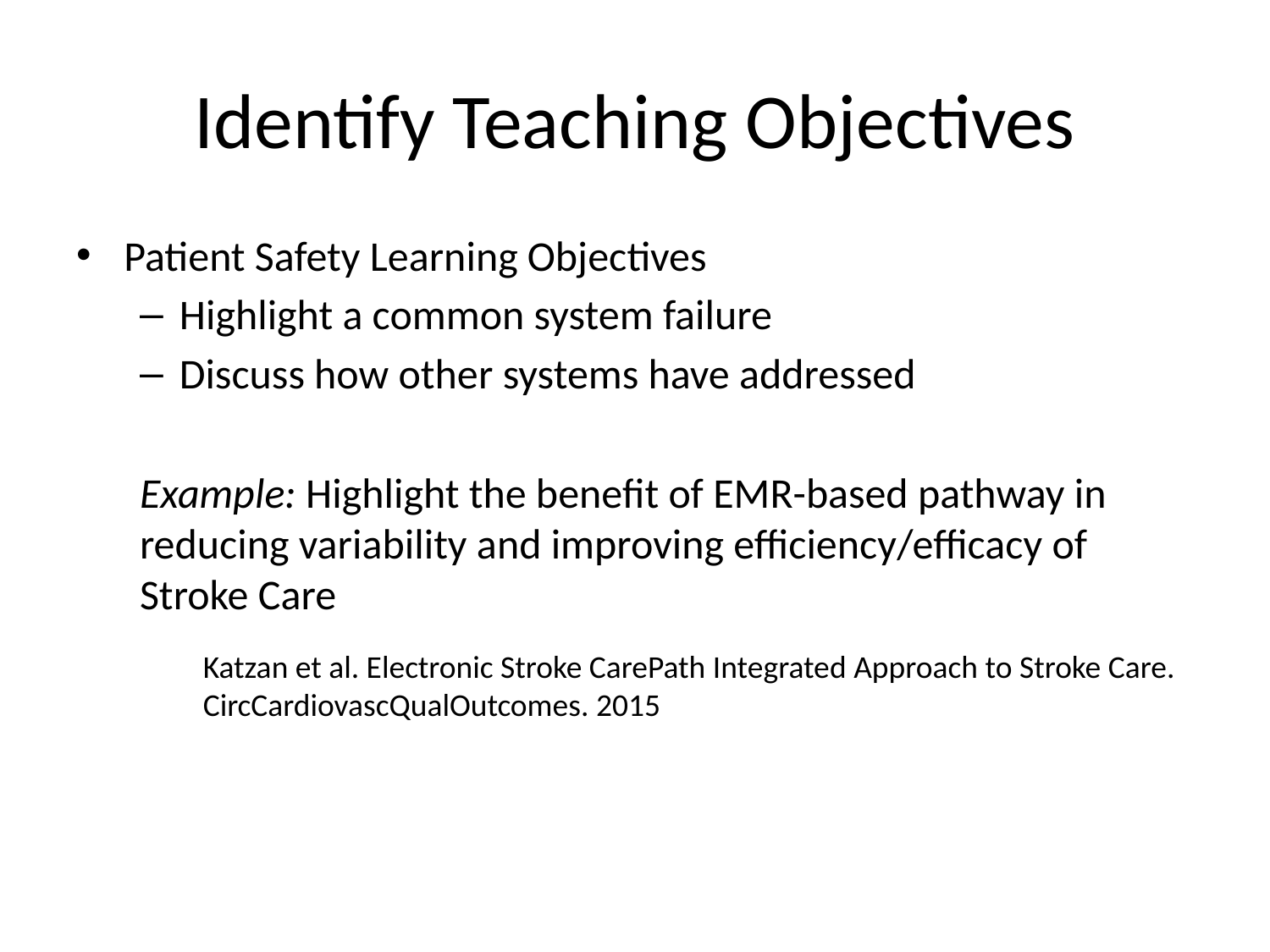

# Identify Teaching Objectives
Patient Safety Learning Objectives
Highlight a common system failure
Discuss how other systems have addressed
Example: Highlight the benefit of EMR-based pathway in reducing variability and improving efficiency/efficacy of Stroke Care
Katzan et al. Electronic Stroke CarePath Integrated Approach to Stroke Care. CircCardiovascQualOutcomes. 2015

## Slide 31
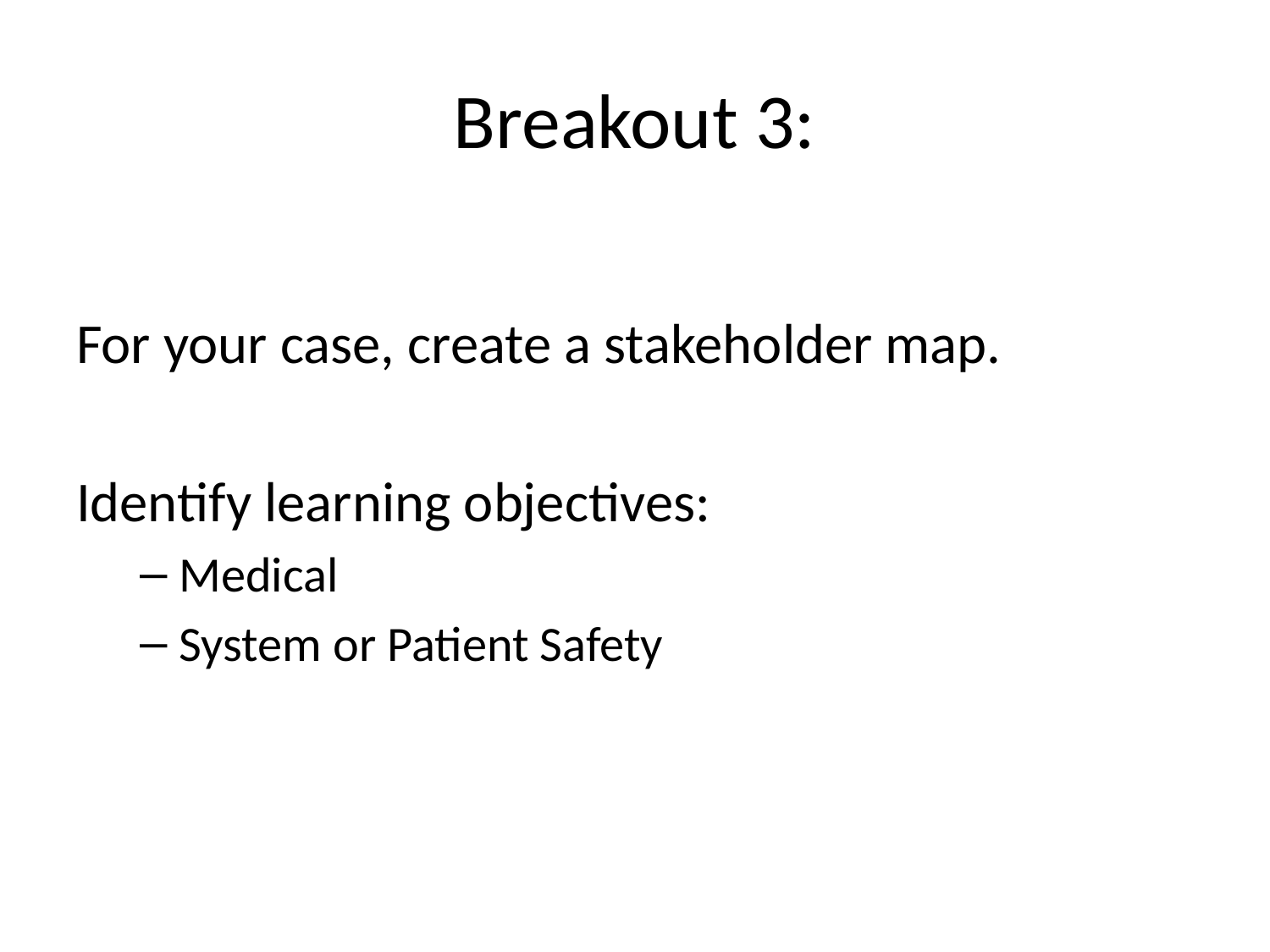

# Breakout 3:
For your case, create a stakeholder map.
Identify learning objectives:
Medical
System or Patient Safety

## Slide 32
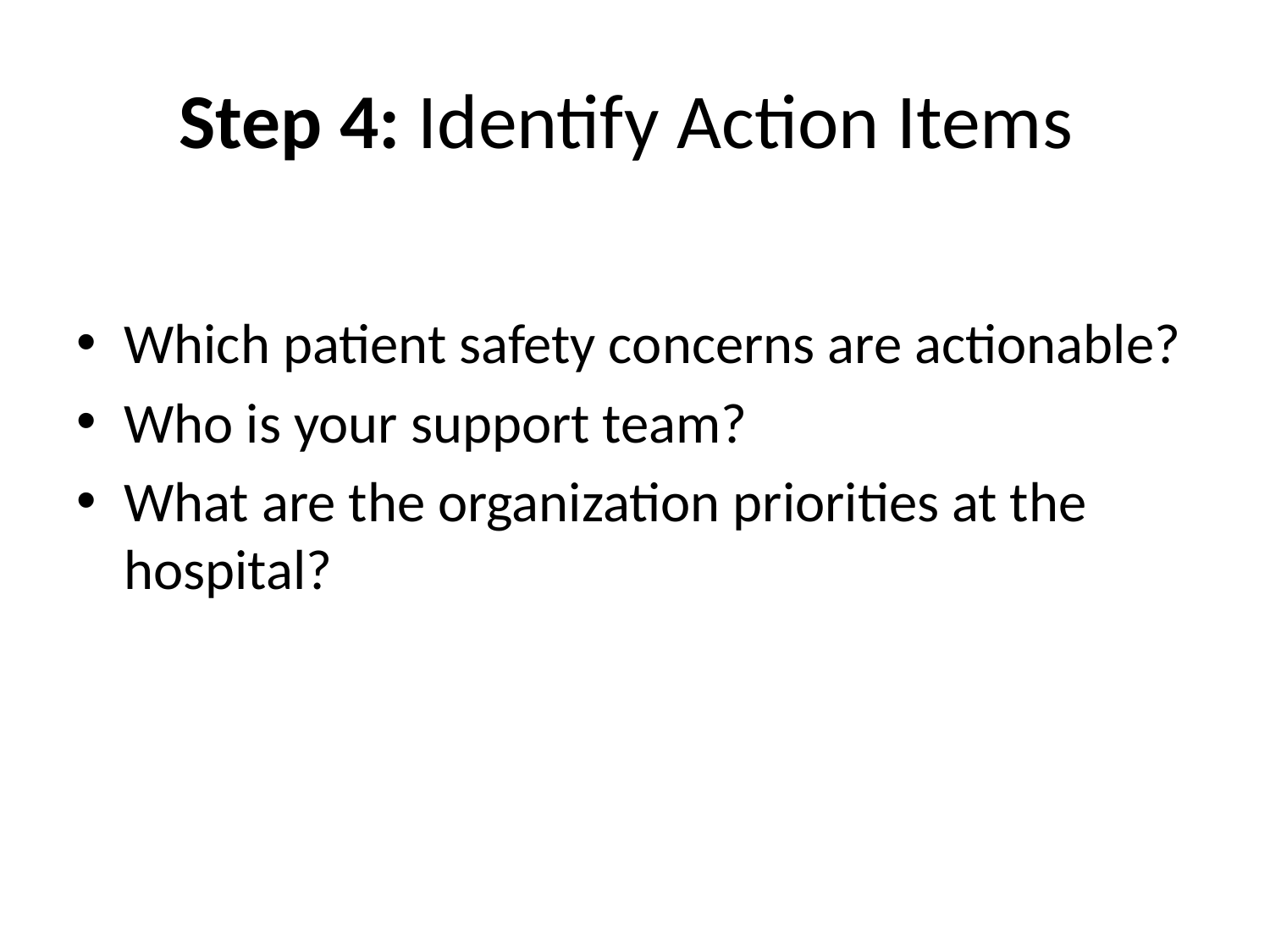

# Step 4: Identify Action Items
Which patient safety concerns are actionable?
Who is your support team?
What are the organization priorities at the hospital?

## Slide 33
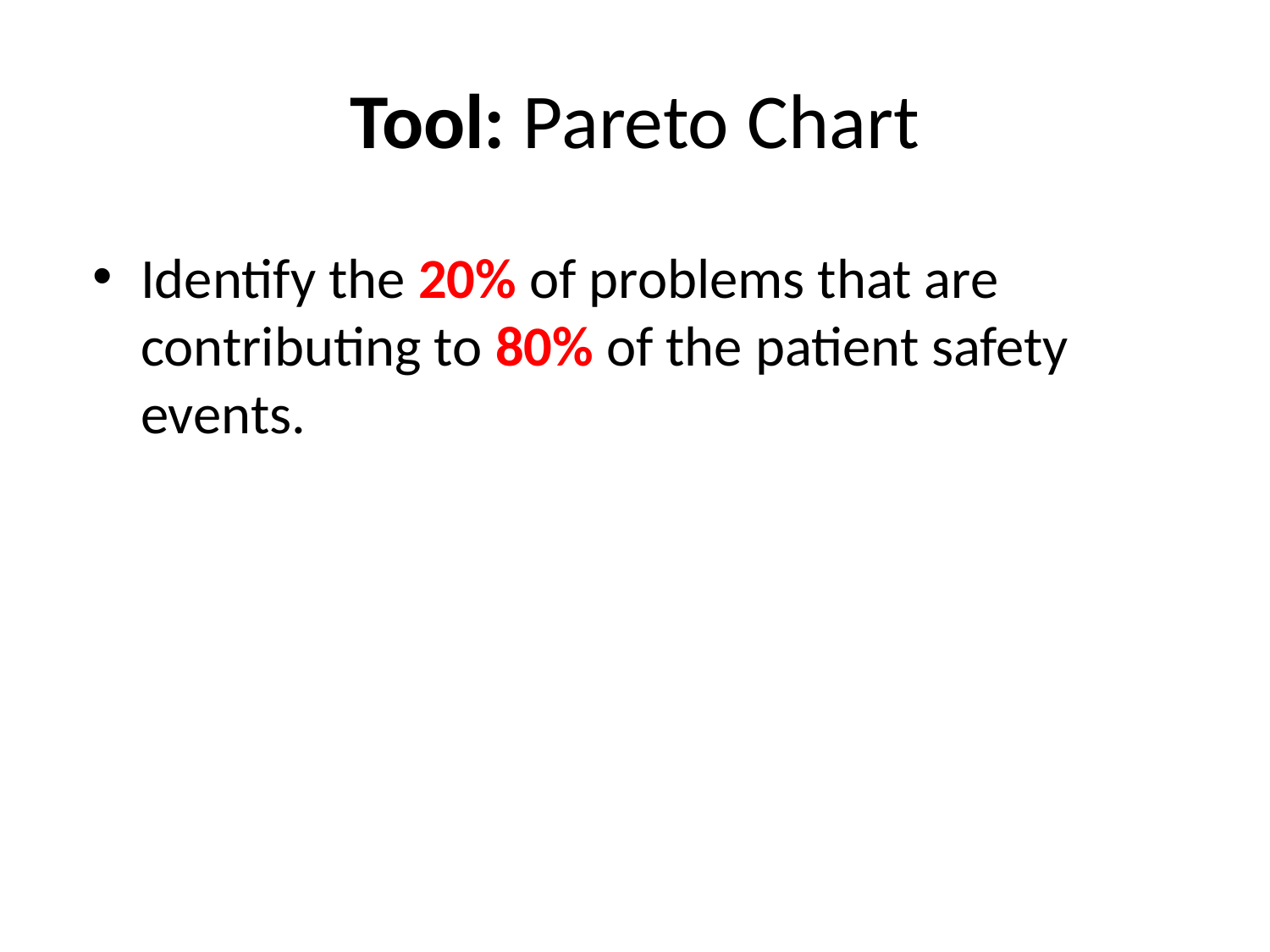

# Tool: Pareto Chart
Identify the 20% of problems that are contributing to 80% of the patient safety events.

## Slide 34
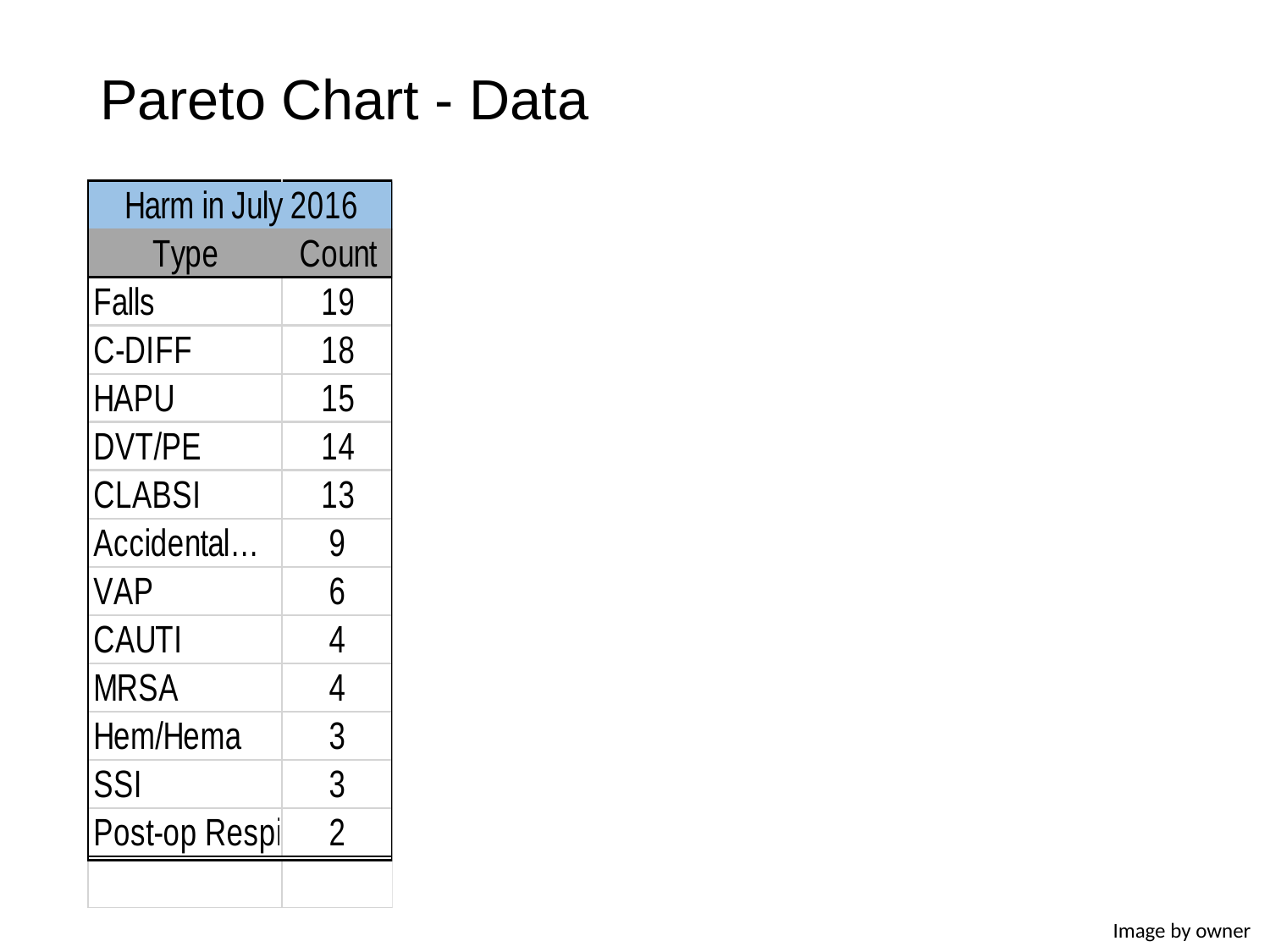

# Pareto Chart - Data
Image by owner

## Slide 35
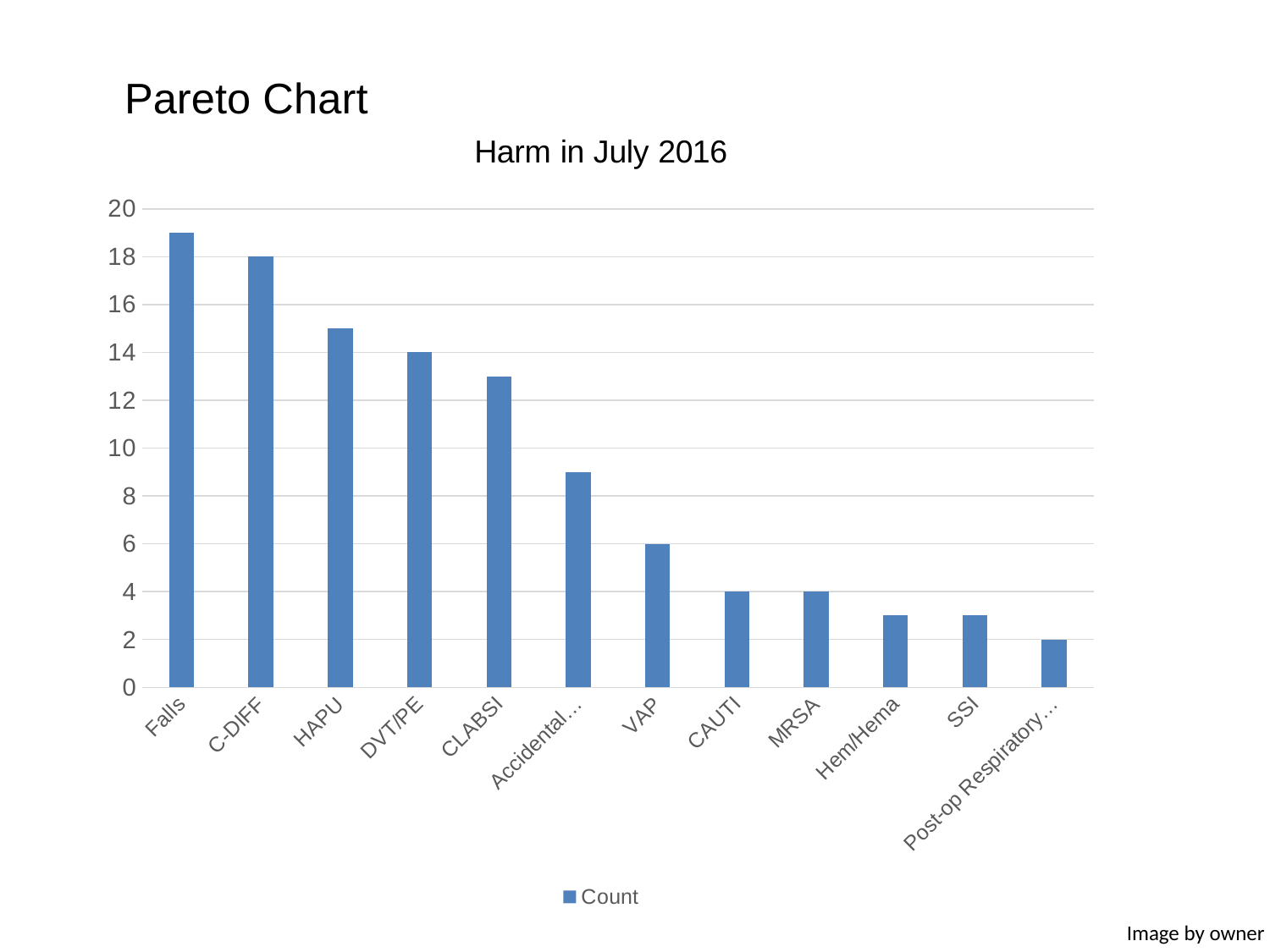

# Pareto Chart
### Chart: Harm in July 2016
| Category | Count |
|---|---|
| Falls | 19.0 |
| C-DIFF | 18.0 |
| HAPU | 15.0 |
| DVT/PE | 14.0 |
| CLABSI | 13.0 |
| Accidental… | 9.0 |
| VAP | 6.0 |
| CAUTI | 4.0 |
| MRSA | 4.0 |
| Hem/Hema | 3.0 |
| SSI | 3.0 |
| Post-op Respiratory… | 2.0 |Image by owner

## Slide 36
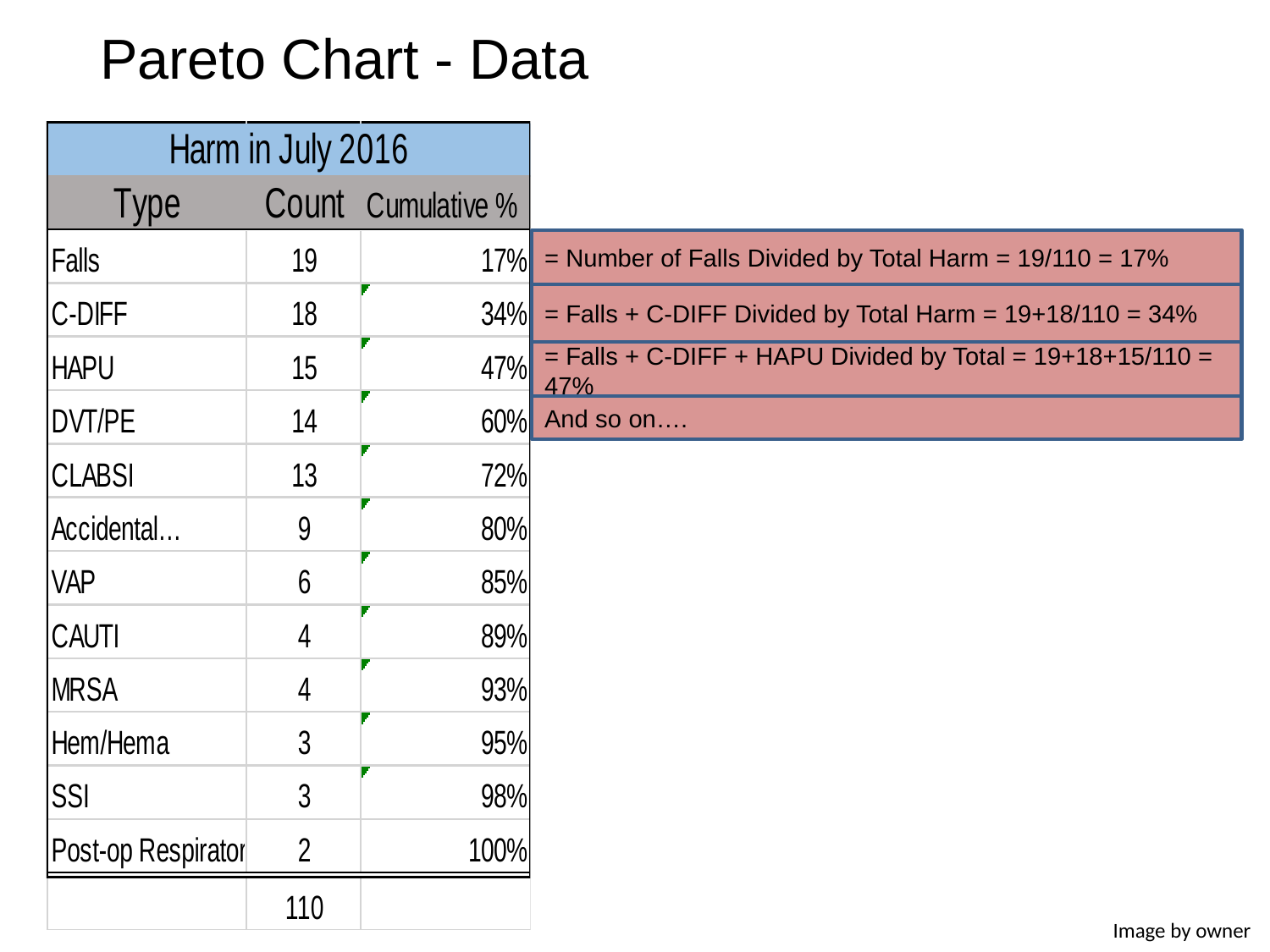

# Pareto Chart - Data
= Number of Falls Divided by Total Harm = 19/110 = 17%
= Falls + C-DIFF Divided by Total Harm = 19+18/110 = 34%
= Falls + C-DIFF + HAPU Divided by Total = 19+18+15/110 = 47%
And so on….
Image by owner

## Slide 37
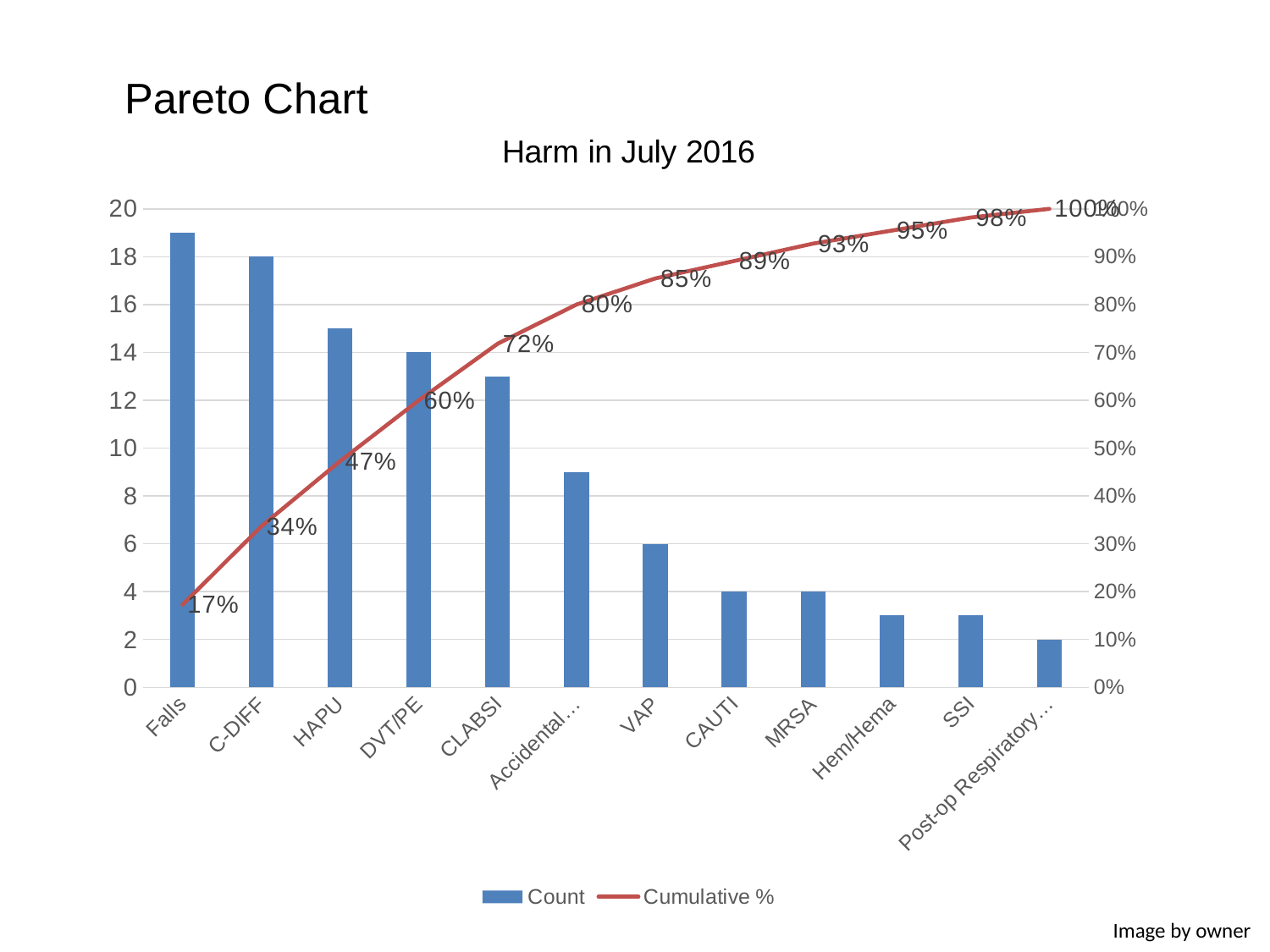

# Pareto Chart
### Chart: Harm in July 2016
| Category | Count | Cumulative % |
|---|---|---|
| Falls | 19.0 | 0.172727272727273 |
| C-DIFF | 18.0 | 0.336363636363636 |
| HAPU | 15.0 | 0.472727272727273 |
| DVT/PE | 14.0 | 0.6 |
| CLABSI | 13.0 | 0.718181818181818 |
| Accidental… | 9.0 | 0.8 |
| VAP | 6.0 | 0.854545454545455 |
| CAUTI | 4.0 | 0.890909090909091 |
| MRSA | 4.0 | 0.927272727272727 |
| Hem/Hema | 3.0 | 0.954545454545455 |
| SSI | 3.0 | 0.981818181818182 |
| Post-op Respiratory… | 2.0 | 1.0 |Image by owner

## Slide 38
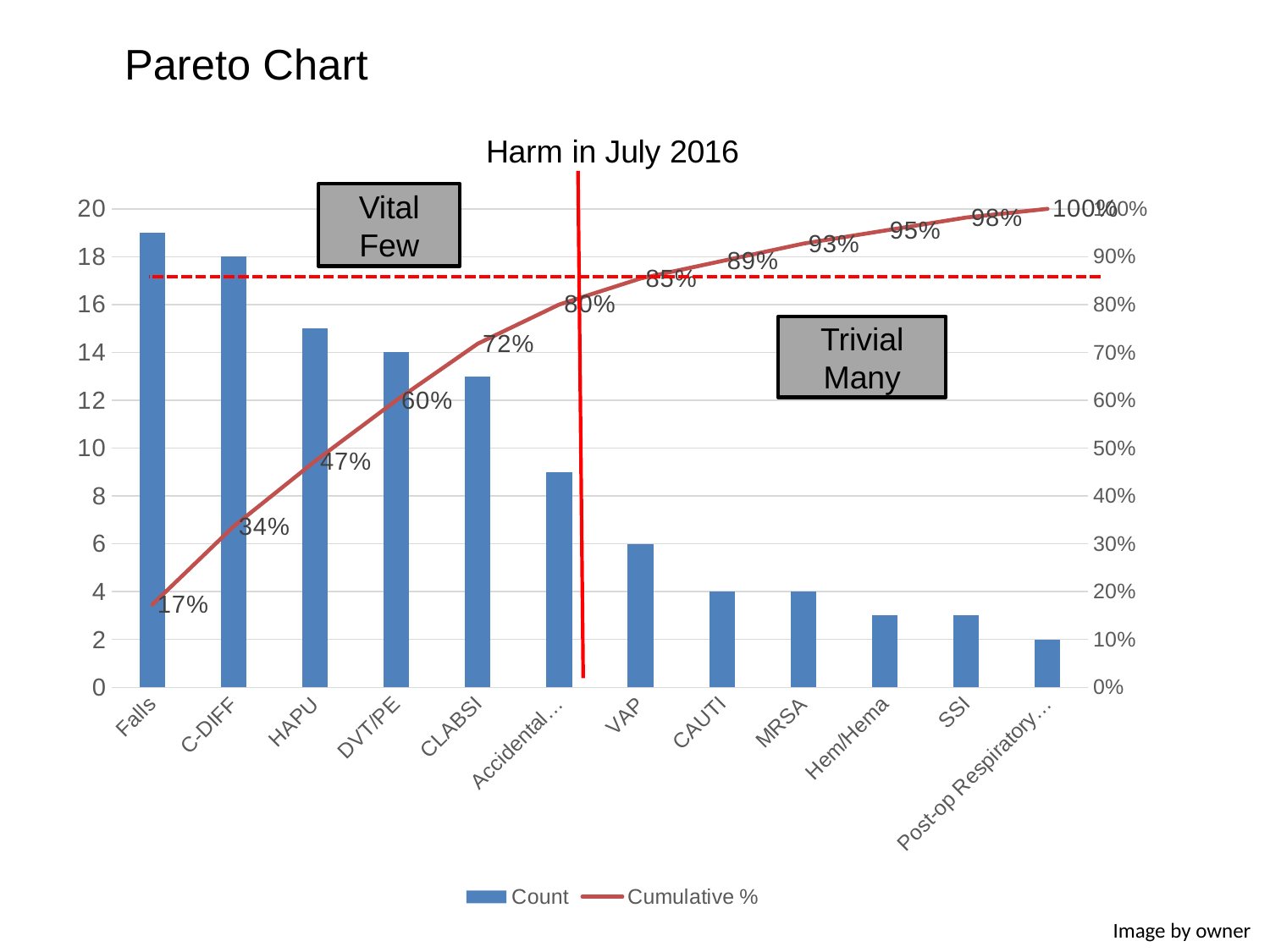

# Pareto Chart
### Chart: Harm in July 2016
| Category | Count | Cumulative % |
|---|---|---|
| Falls | 19.0 | 0.172727272727273 |
| C-DIFF | 18.0 | 0.336363636363636 |
| HAPU | 15.0 | 0.472727272727273 |
| DVT/PE | 14.0 | 0.6 |
| CLABSI | 13.0 | 0.718181818181818 |
| Accidental… | 9.0 | 0.8 |
| VAP | 6.0 | 0.854545454545455 |
| CAUTI | 4.0 | 0.890909090909091 |
| MRSA | 4.0 | 0.927272727272727 |
| Hem/Hema | 3.0 | 0.954545454545455 |
| SSI | 3.0 | 0.981818181818182 |
| Post-op Respiratory… | 2.0 | 1.0 |Vital Few
Trivial Many
Image by owner

## Slide 39
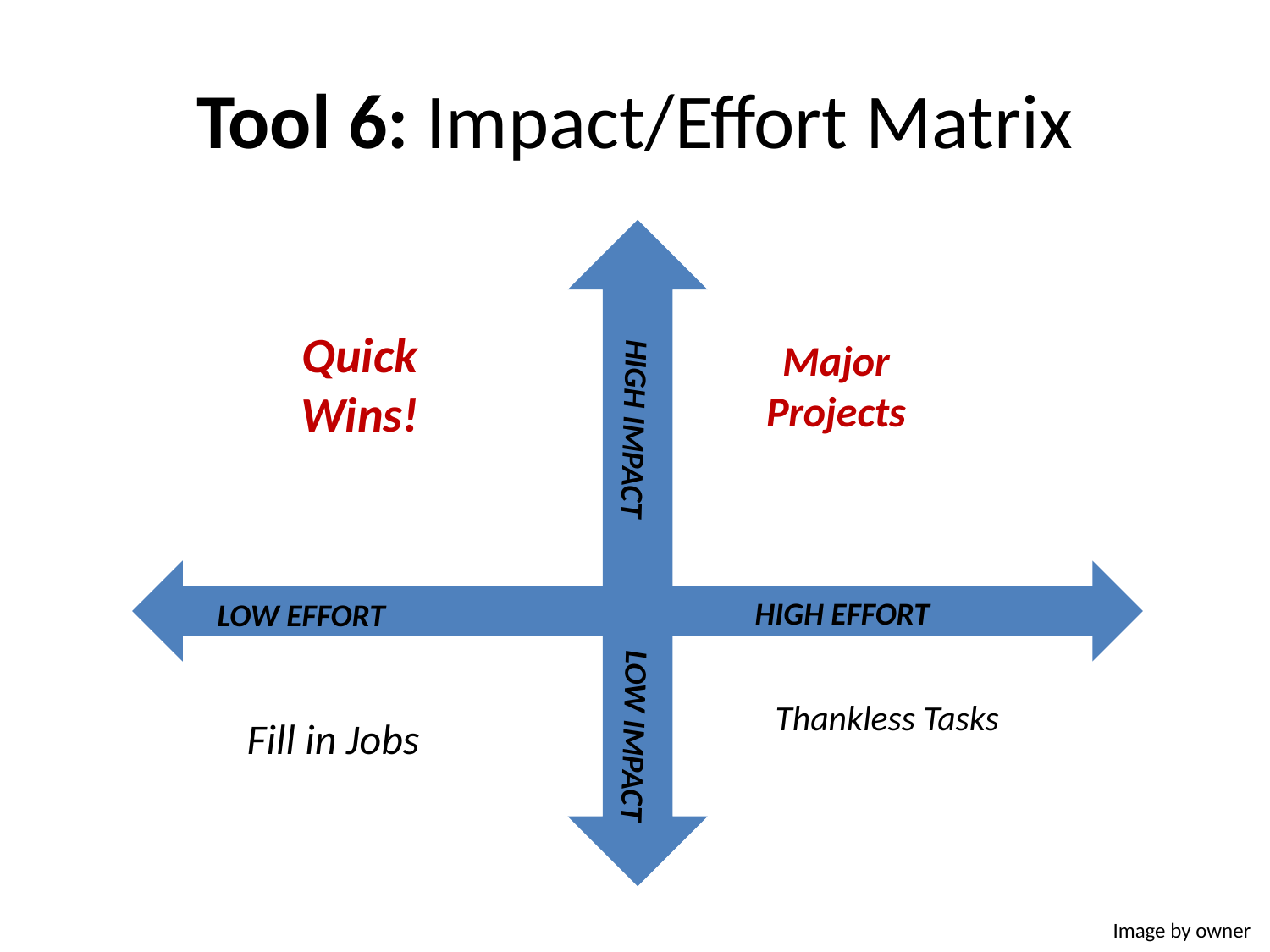

# Tool 6: Impact/Effort Matrix
Quick Wins!
Major Projects
HIGH IMPACT
HIGH EFFORT
LOW EFFORT
Thankless Tasks
LOW IMPACT
Fill in Jobs
Image by owner

## Slide 40
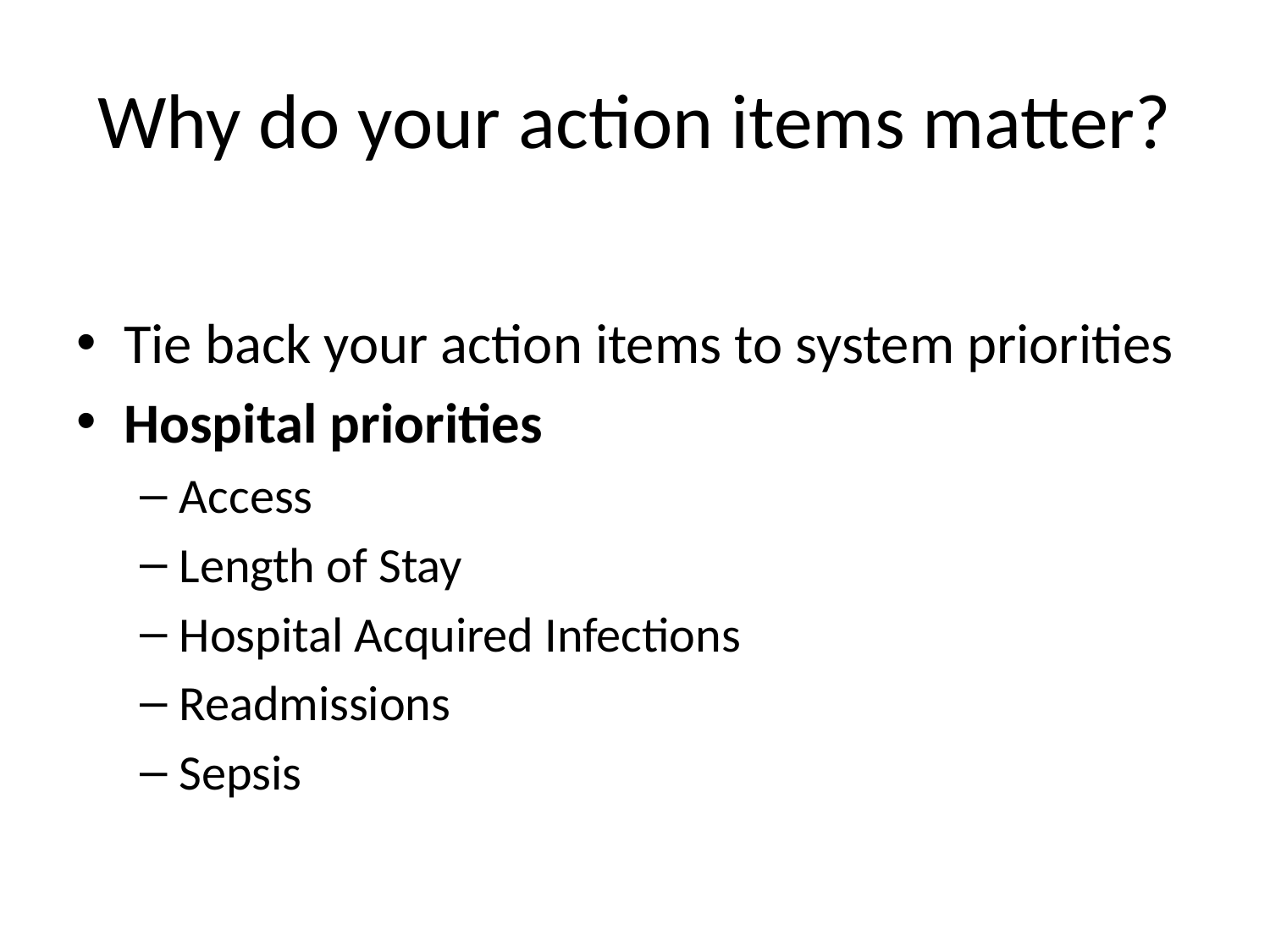

# Why do your action items matter?
Tie back your action items to system priorities
Hospital priorities
Access
Length of Stay
Hospital Acquired Infections
Readmissions
Sepsis

## Slide 41
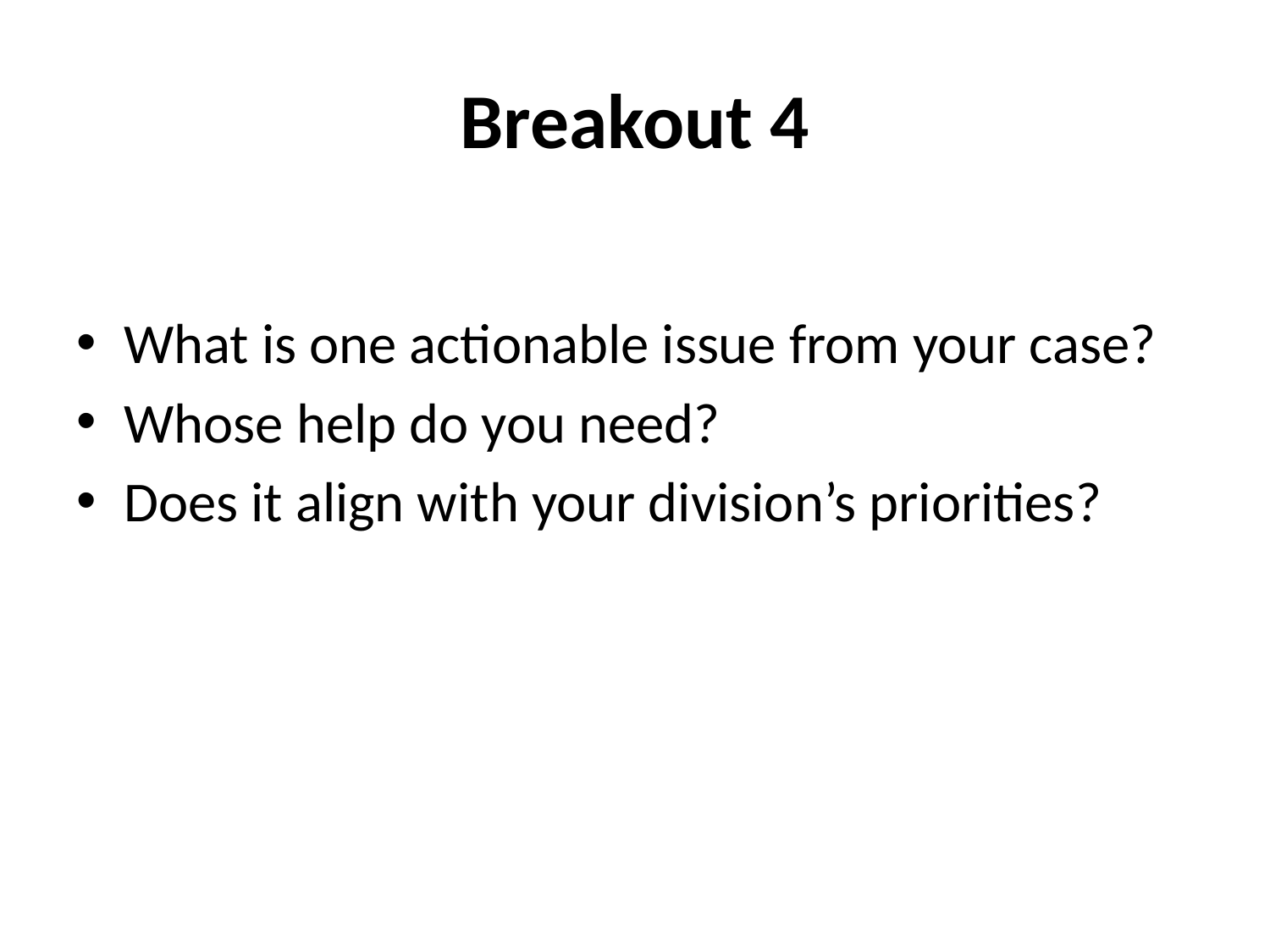

# Breakout 4
What is one actionable issue from your case?
Whose help do you need?
Does it align with your division’s priorities?

## Slide 42
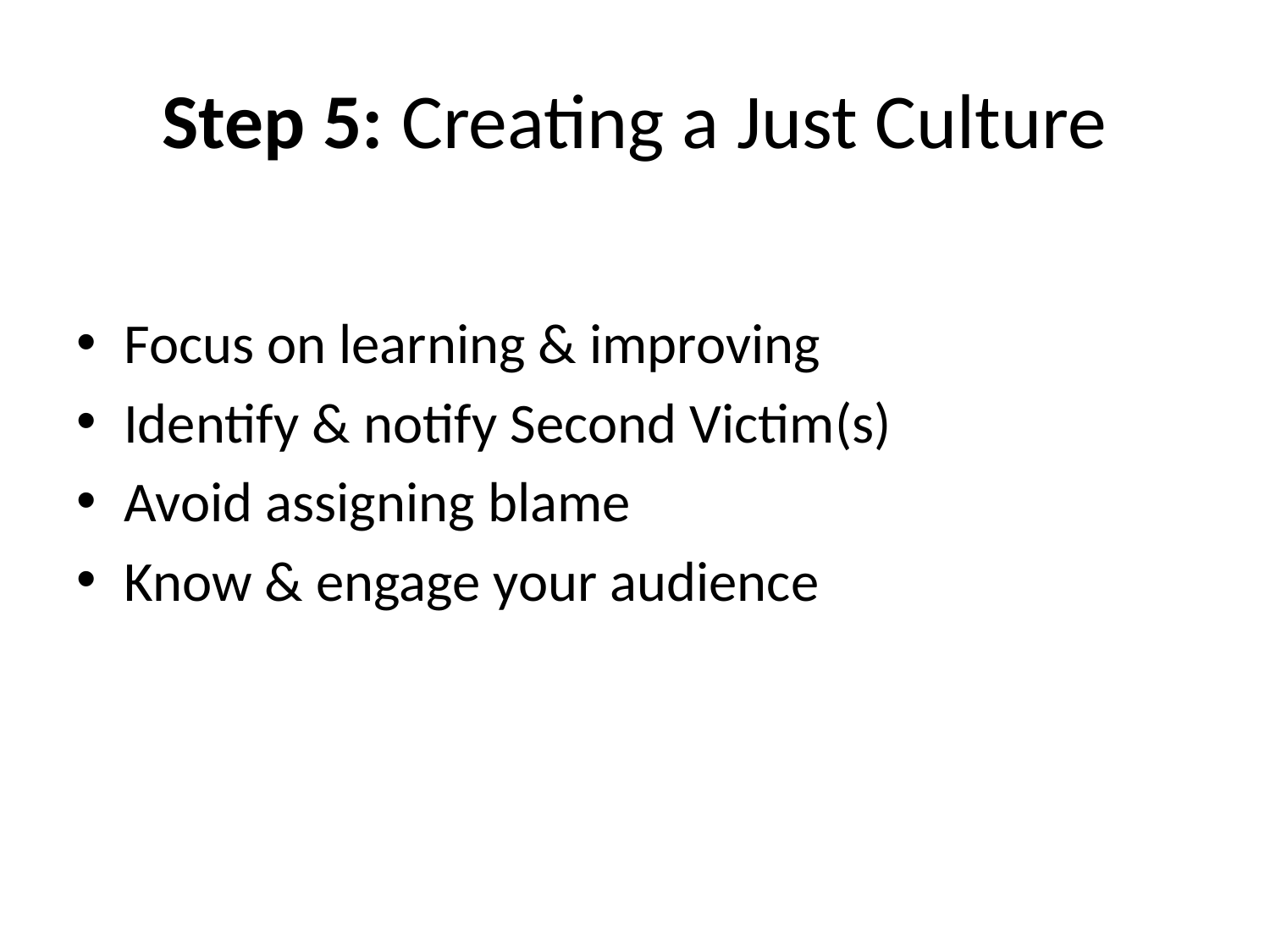

# Step 5: Creating a Just Culture
Focus on learning & improving
Identify & notify Second Victim(s)
Avoid assigning blame
Know & engage your audience

## Slide 43
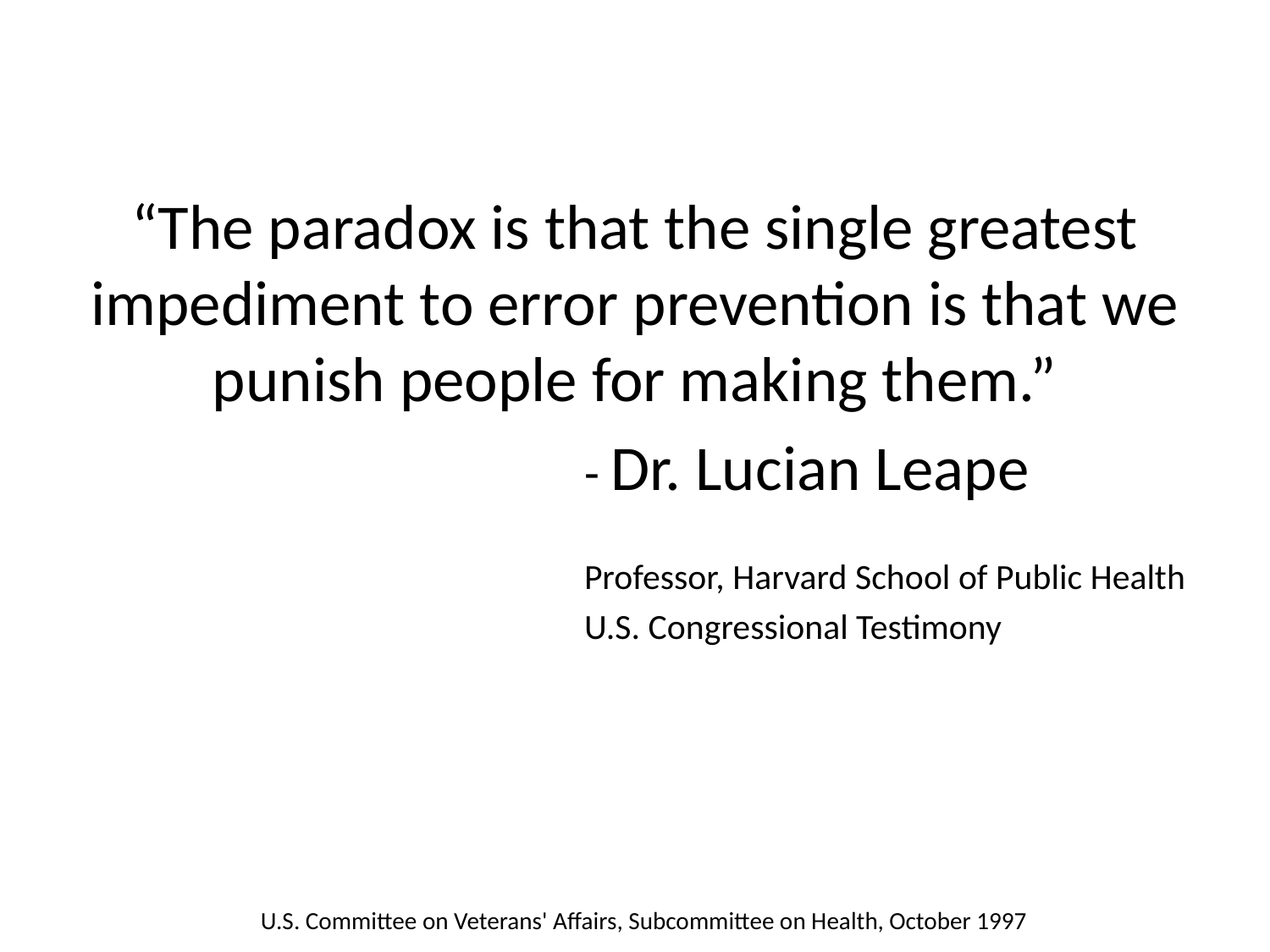

“The paradox is that the single greatest impediment to error prevention is that we punish people for making them.”
				- Dr. Lucian Leape			 						 				Professor, Harvard School of Public Health
				U.S. Congressional Testimony
U.S. Committee on Veterans' Affairs, Subcommittee on Health, October 1997

## Slide 44
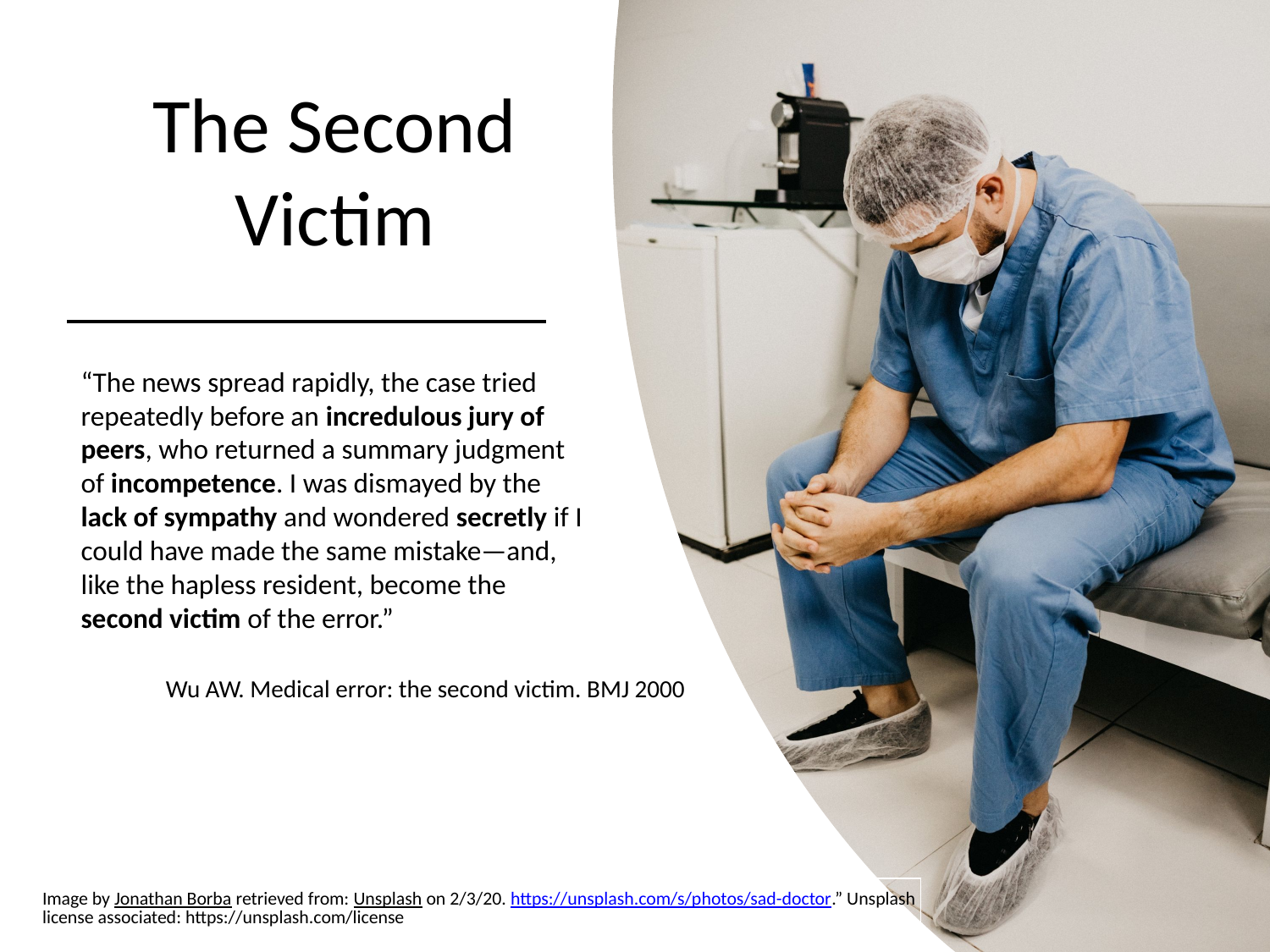

# The Second Victim
“The news spread rapidly, the case tried repeatedly before an incredulous jury of peers, who returned a summary judgment of incompetence. I was dismayed by the lack of sympathy and wondered secretly if I could have made the same mistake—and, like the hapless resident, become the second victim of the error.”
Wu AW. Medical error: the second victim. BMJ 2000
| Image by Jonathan Borba retrieved from: Unsplash on 2/3/20. https://unsplash.com/s/photos/sad-doctor.” Unsplash license associated: https://unsplash.com/license |
| --- |

## Slide 45
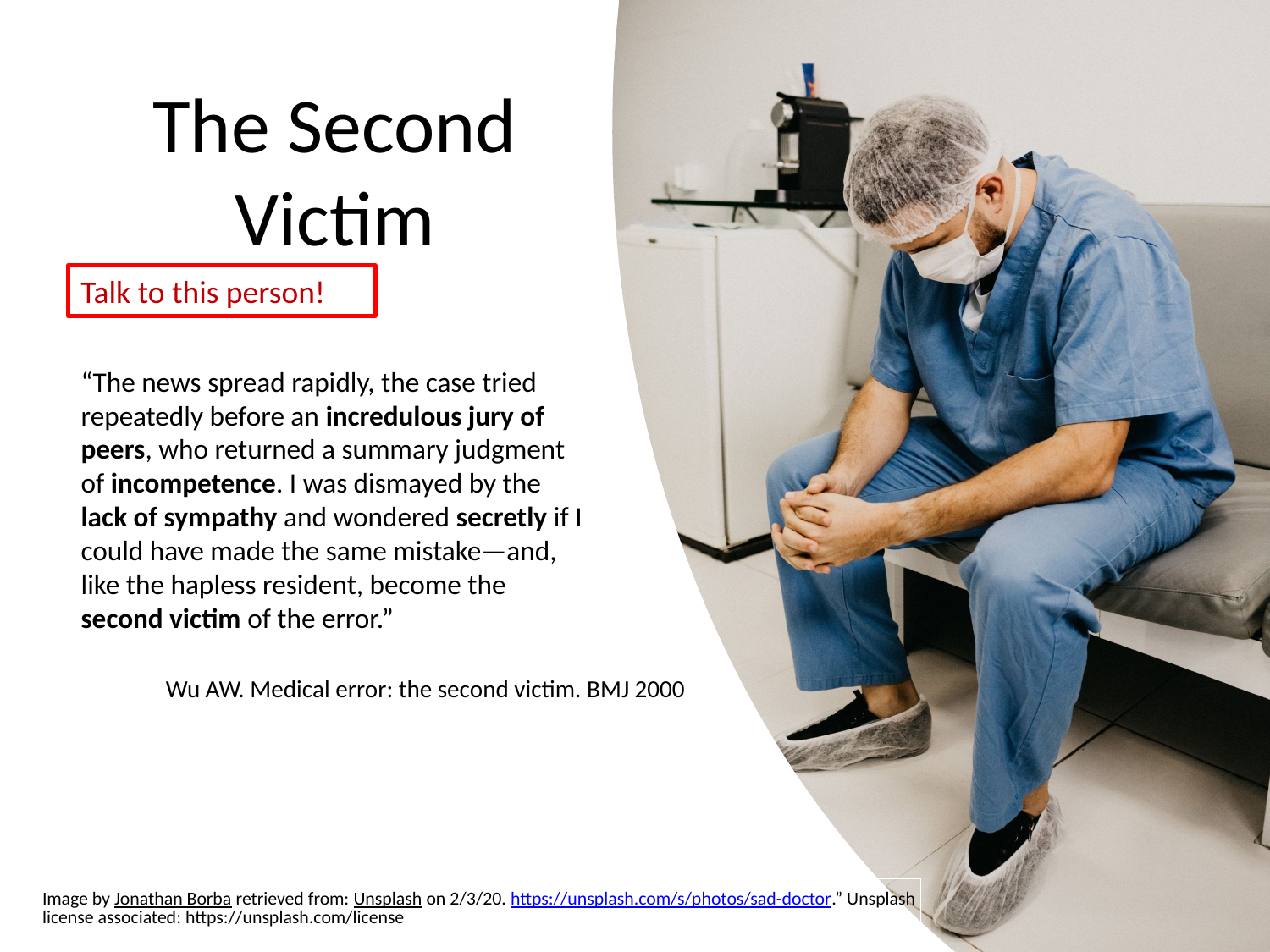

# The Second Victim
Talk to this person!
“The news spread rapidly, the case tried repeatedly before an incredulous jury of peers, who returned a summary judgment of incompetence. I was dismayed by the lack of sympathy and wondered secretly if I could have made the same mistake—and, like the hapless resident, become the second victim of the error.”
Wu AW. Medical error: the second victim. BMJ 2000
| Image by Jonathan Borba retrieved from: Unsplash on 2/3/20. https://unsplash.com/s/photos/sad-doctor.” Unsplash license associated: https://unsplash.com/license |
| --- |

## Slide 46
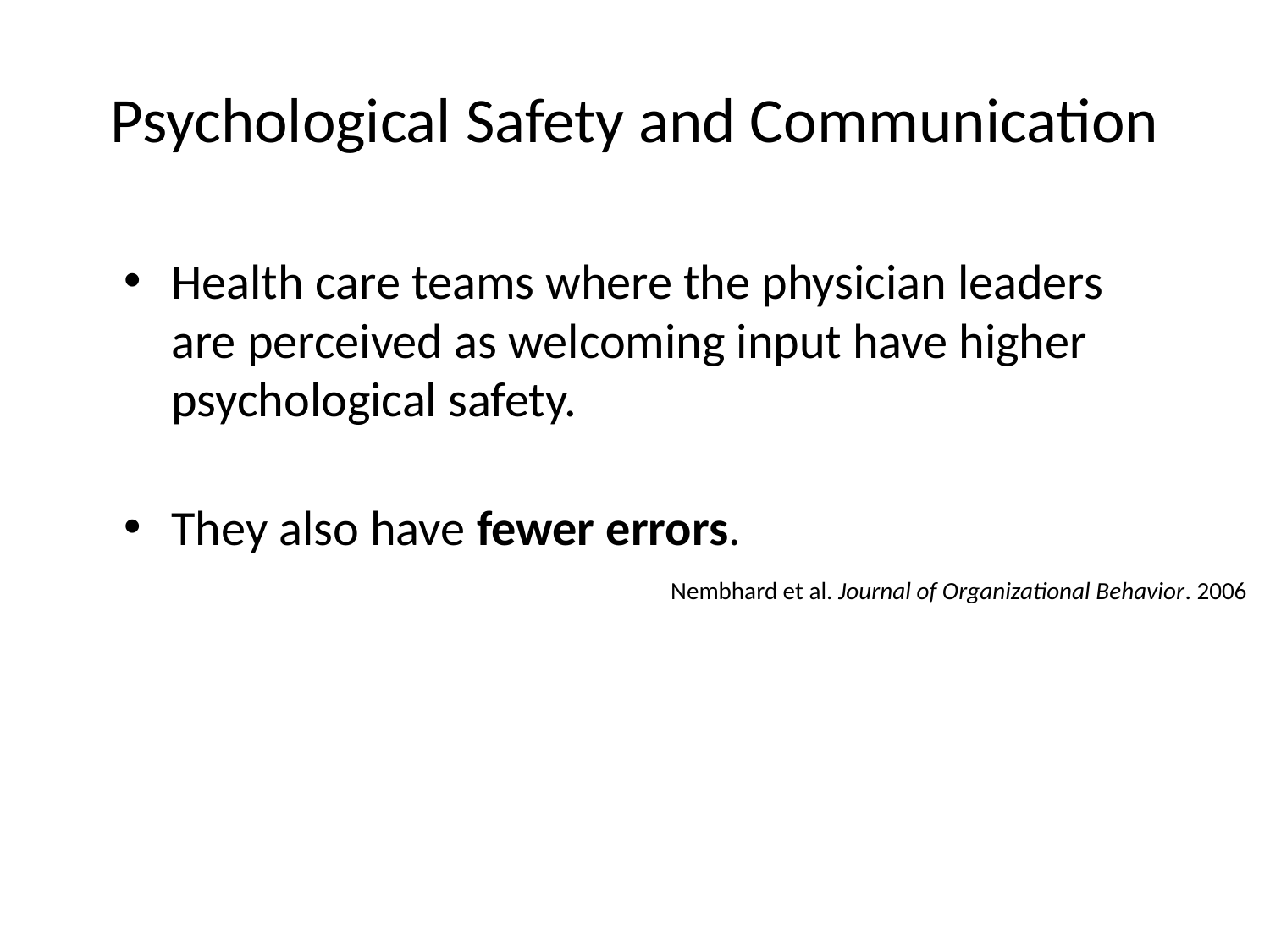

# Psychological Safety and Communication
Health care teams where the physician leaders are perceived as welcoming input have higher psychological safety.
They also have fewer errors.
Nembhard et al. Journal of Organizational Behavior. 2006

## Slide 47
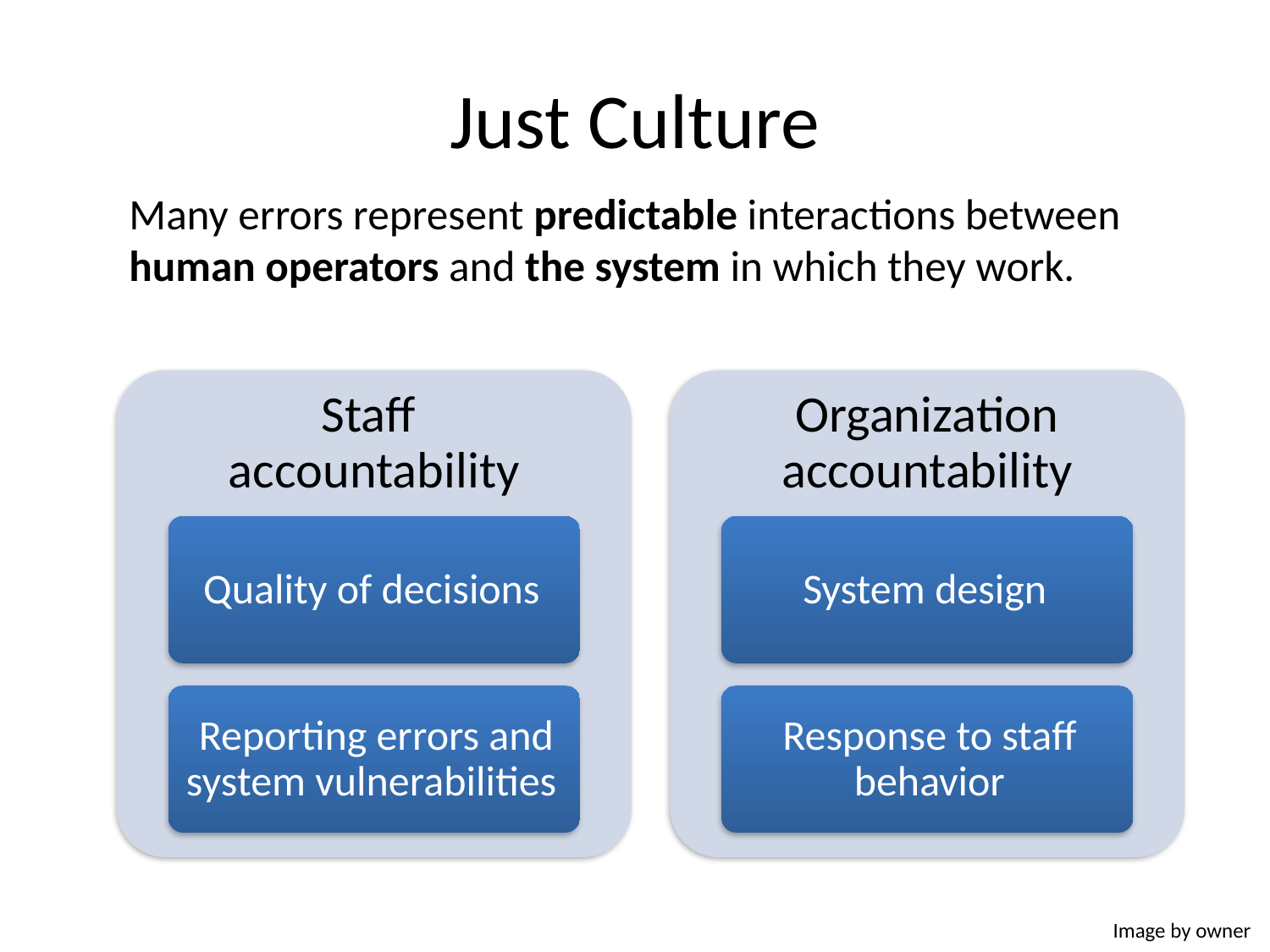

# Just Culture
Many errors represent predictable interactions between human operators and the system in which they work.
Image by owner

## Slide 48
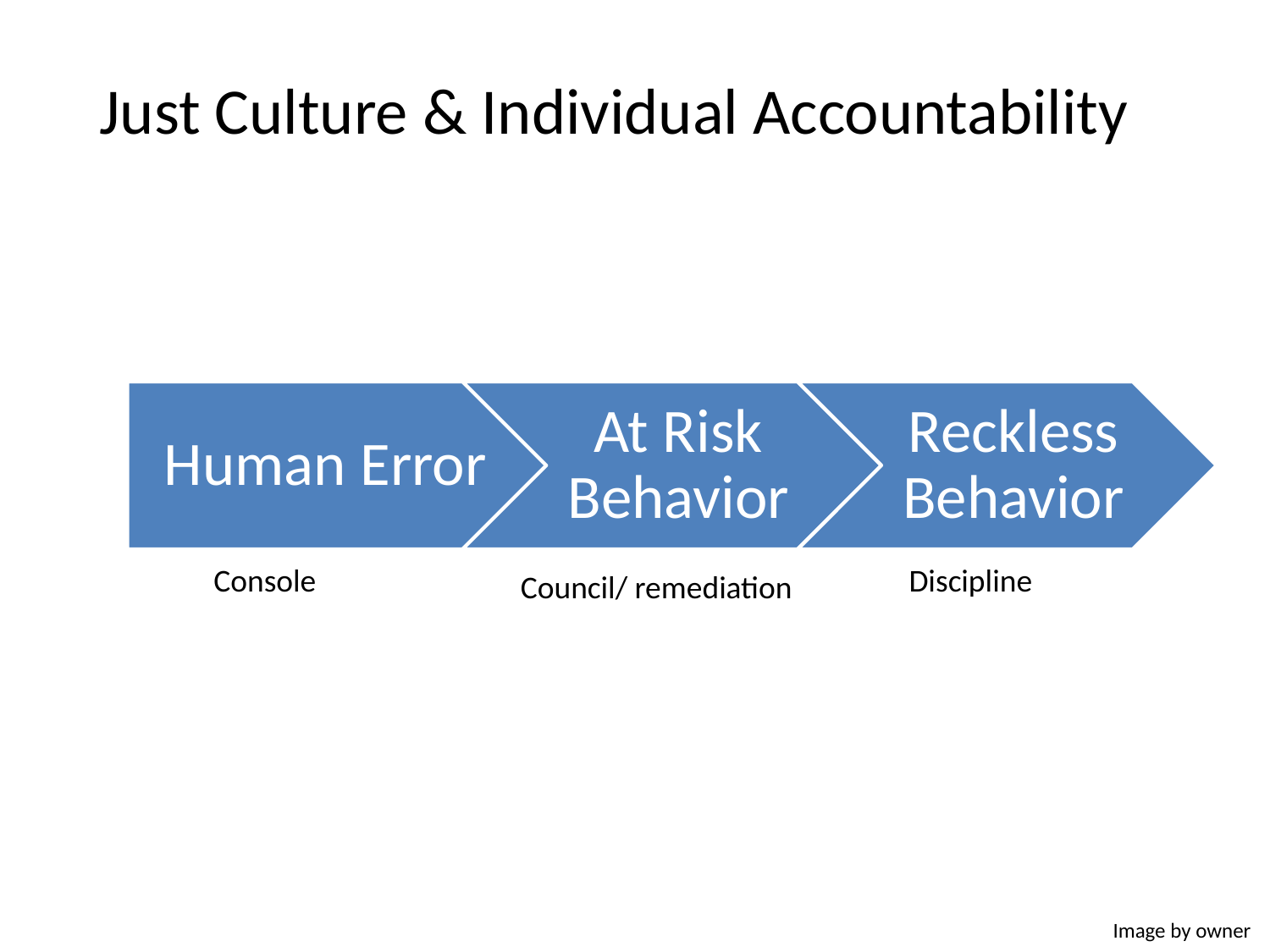

# Just Culture & Individual Accountability
Console
Discipline
Council/ remediation
Image by owner

## Slide 49
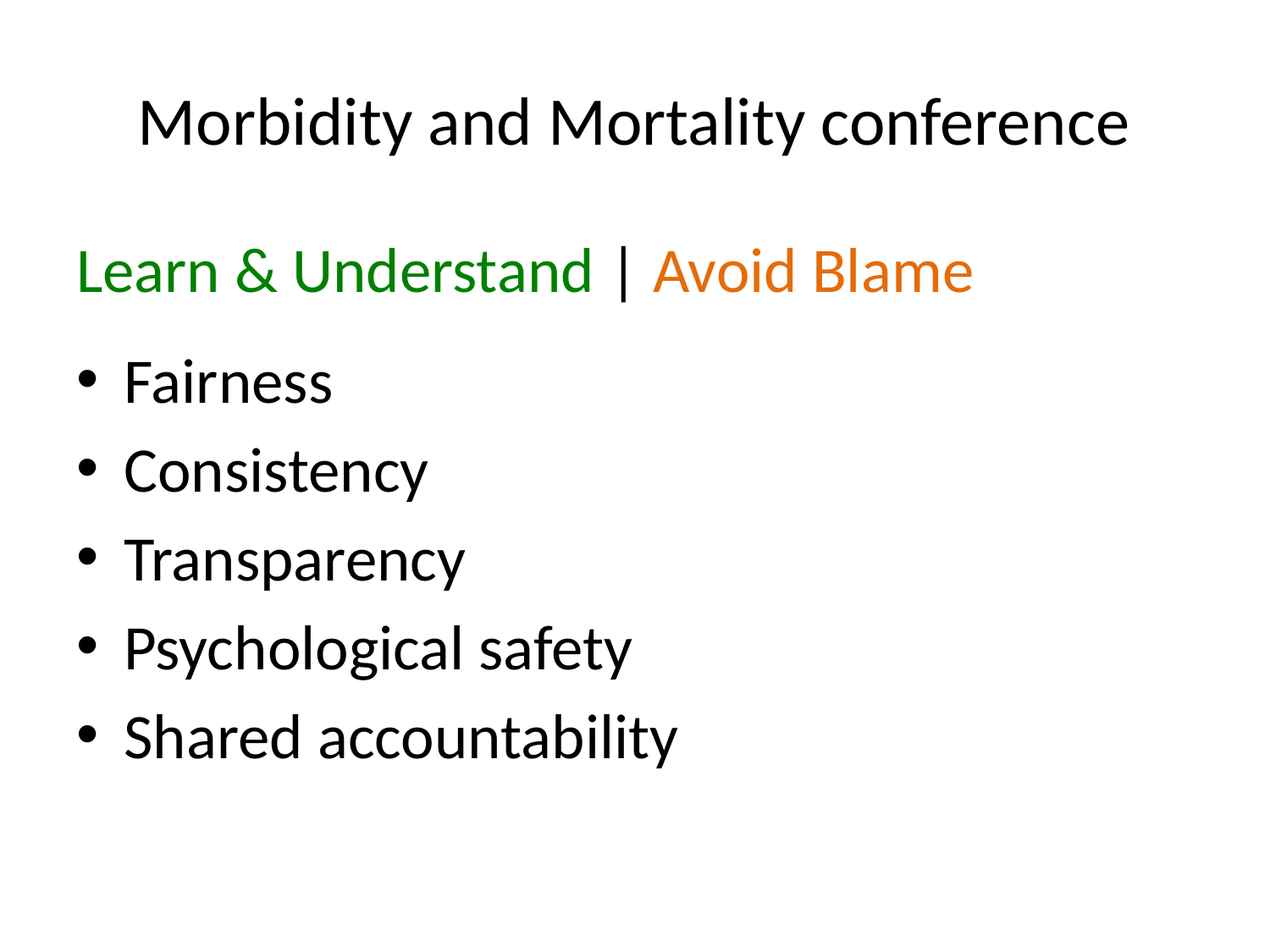

# Morbidity and Mortality conference
Learn & Understand | Avoid Blame
Fairness
Consistency
Transparency
Psychological safety
Shared accountability

## Slide 50
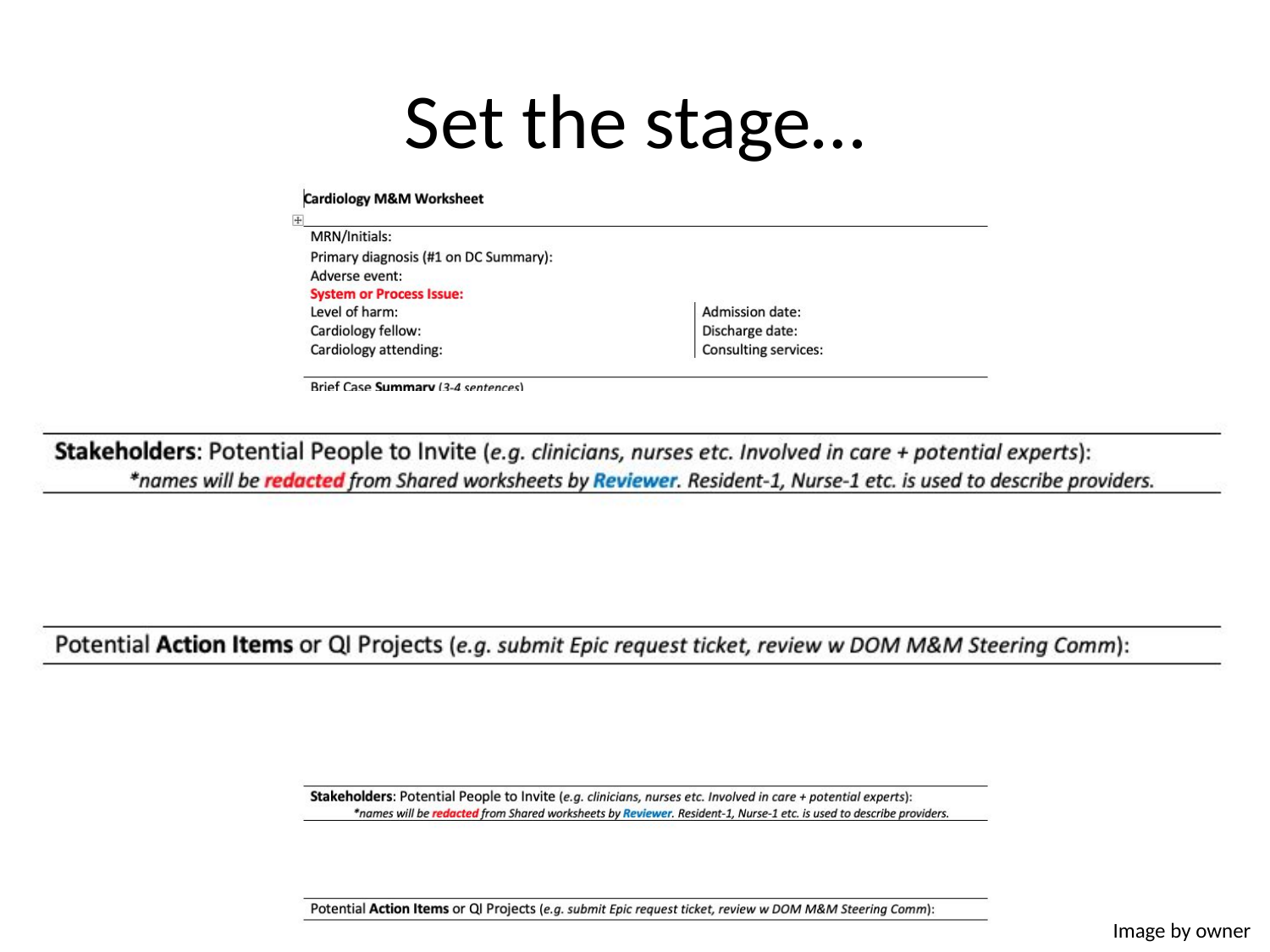

# Set the stage…
Image by owner

## Slide 51
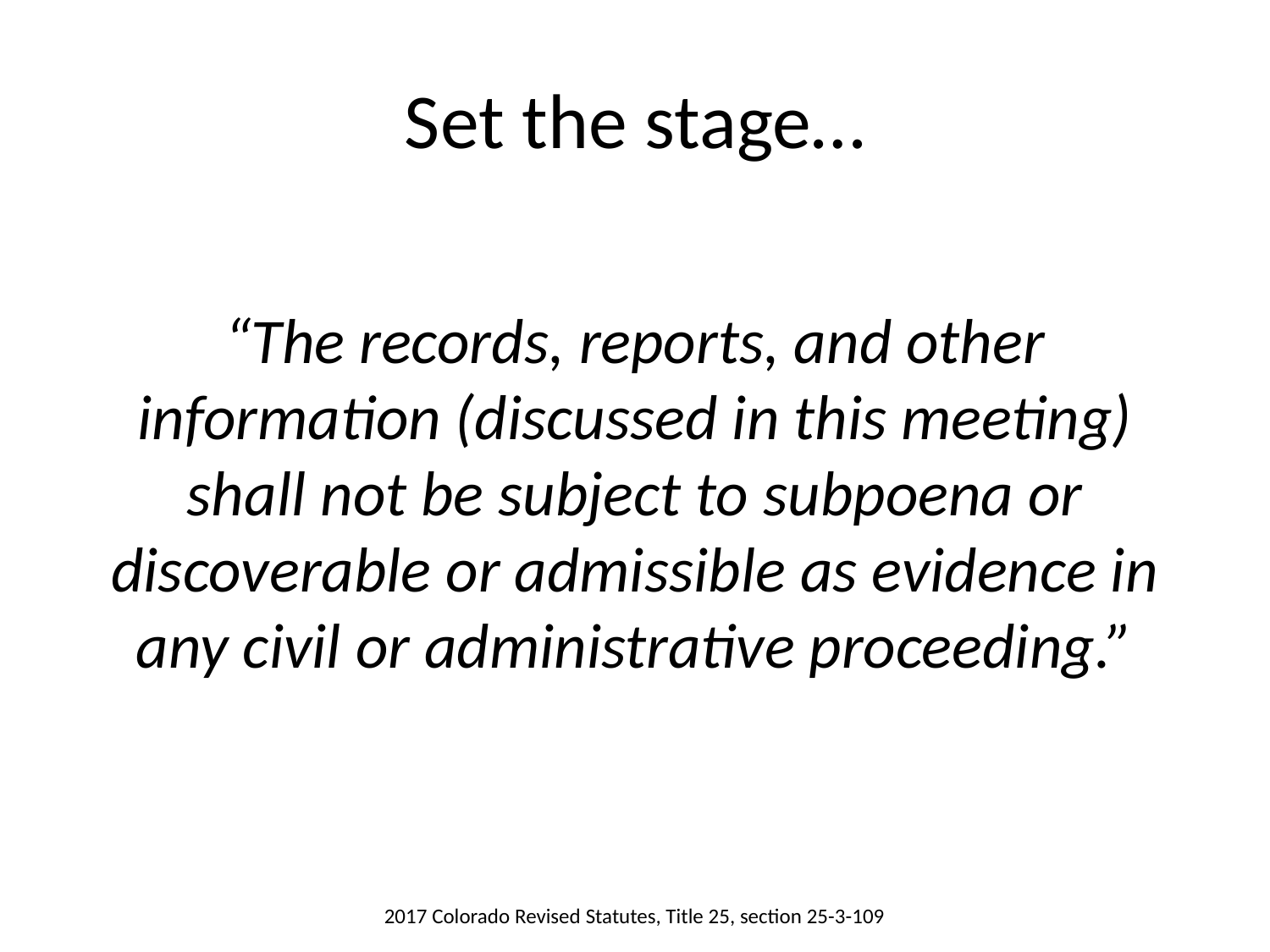

# Set the stage…
“The records, reports, and other information (discussed in this meeting) shall not be subject to subpoena or discoverable or admissible as evidence in any civil or administrative proceeding.”
2017 Colorado Revised Statutes, Title 25, section 25-3-109

## Slide 52
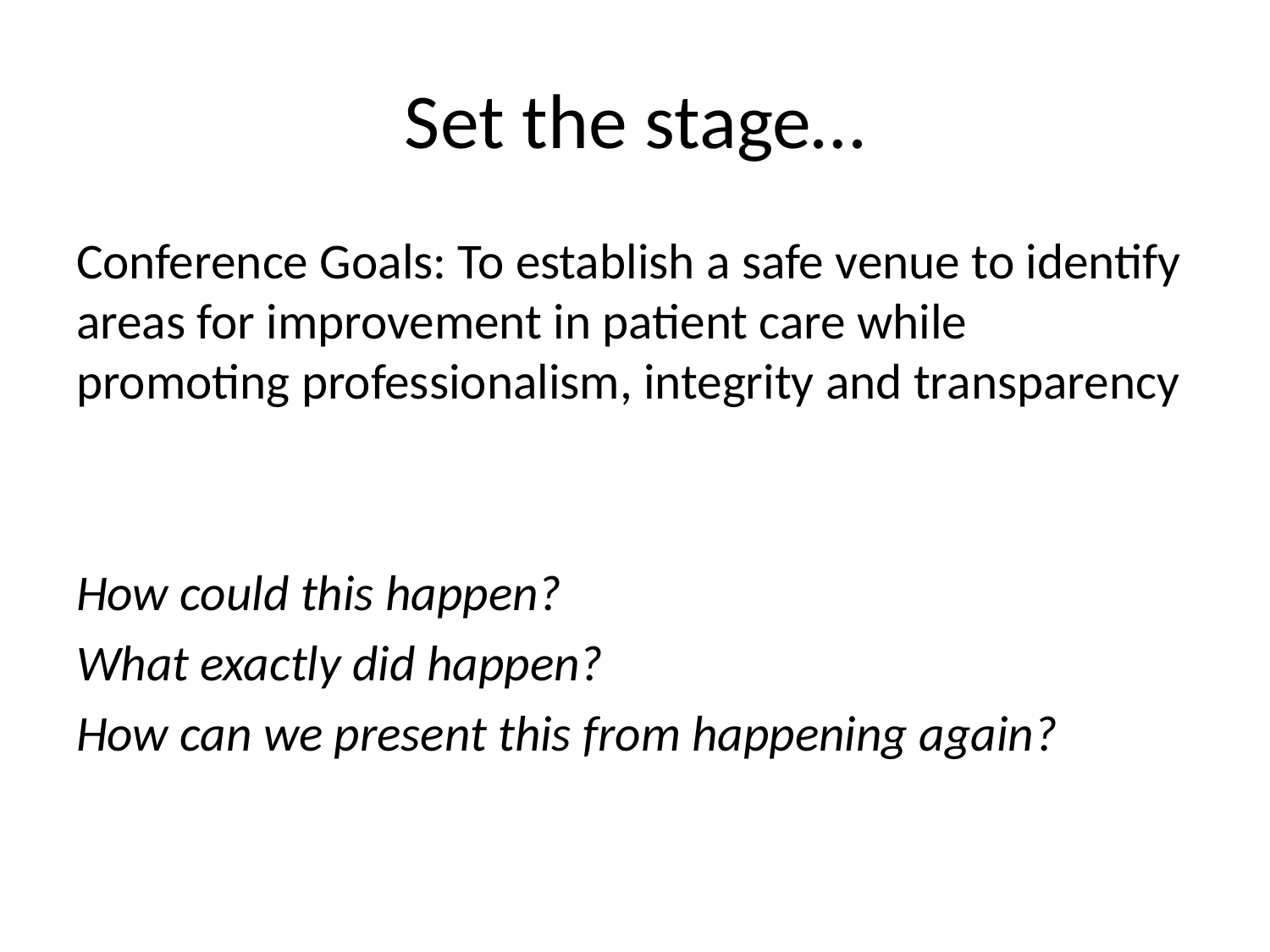

# Set the stage…
Conference Goals: To establish a safe venue to identify areas for improvement in patient care while promoting professionalism, integrity and transparency
How could this happen?
What exactly did happen?
How can we present this from happening again?

## Slide 53
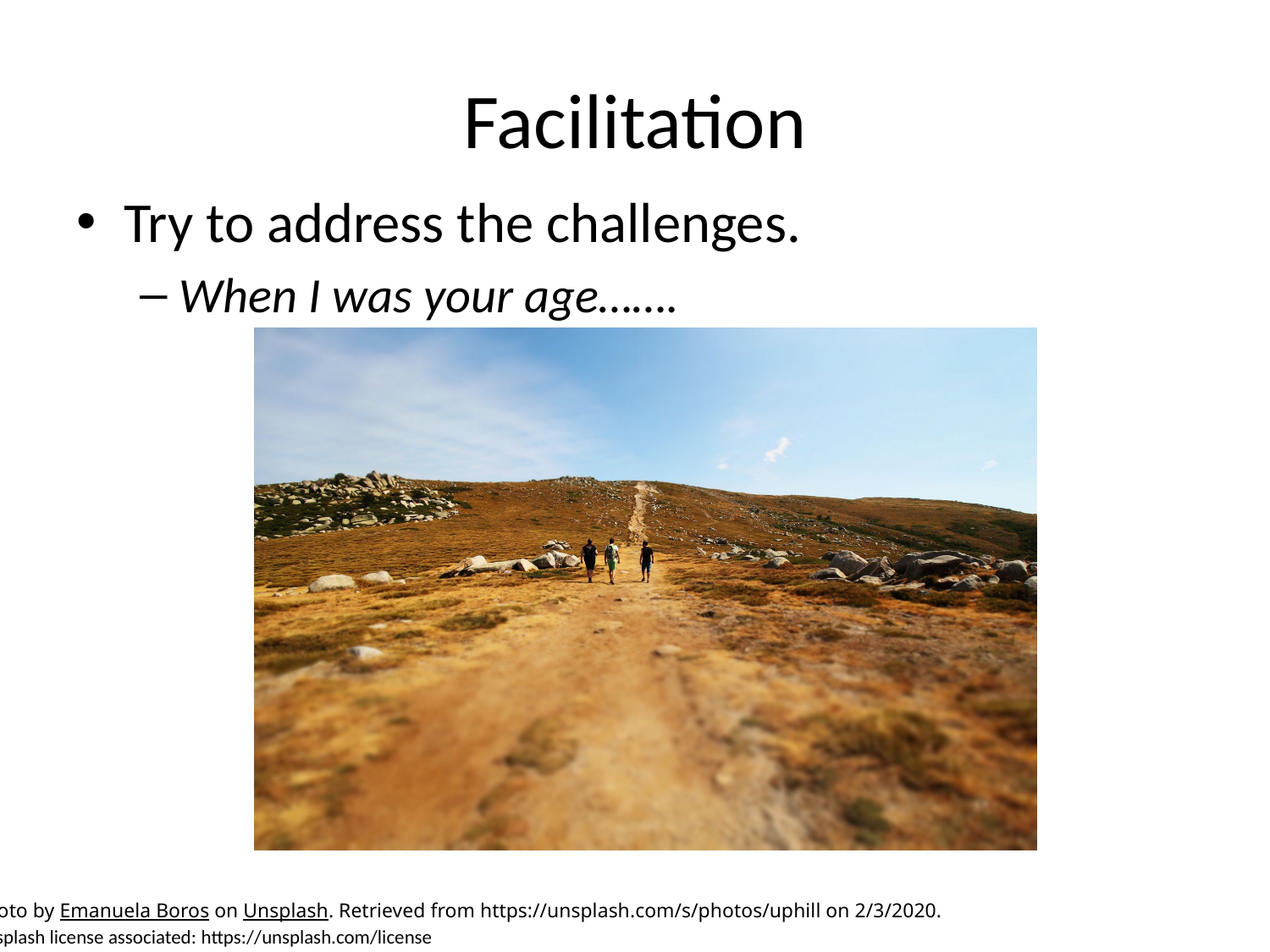

# Facilitation
Try to address the challenges.
When I was your age…….
Photo by Emanuela Boros on Unsplash. Retrieved from https://unsplash.com/s/photos/uphill on 2/3/2020.
Unsplash license associated: https://unsplash.com/license

## Slide 54
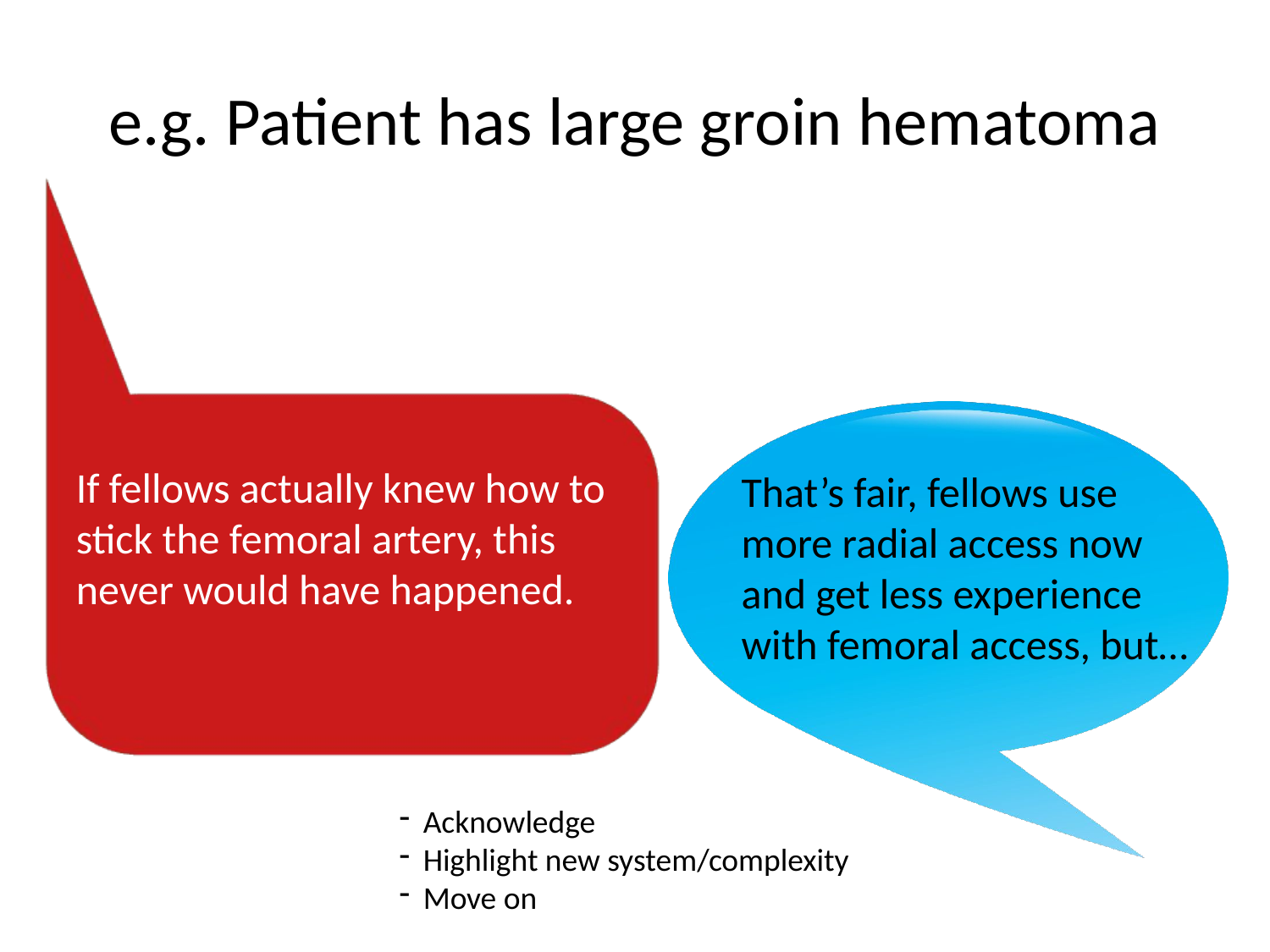

# e.g. Patient has large groin hematoma
That’s fair, fellows use more radial access now and get less experience with femoral access, but…
If fellows actually knew how to stick the femoral artery, this never would have happened.
Acknowledge
Highlight new system/complexity
Move on

## Slide 55
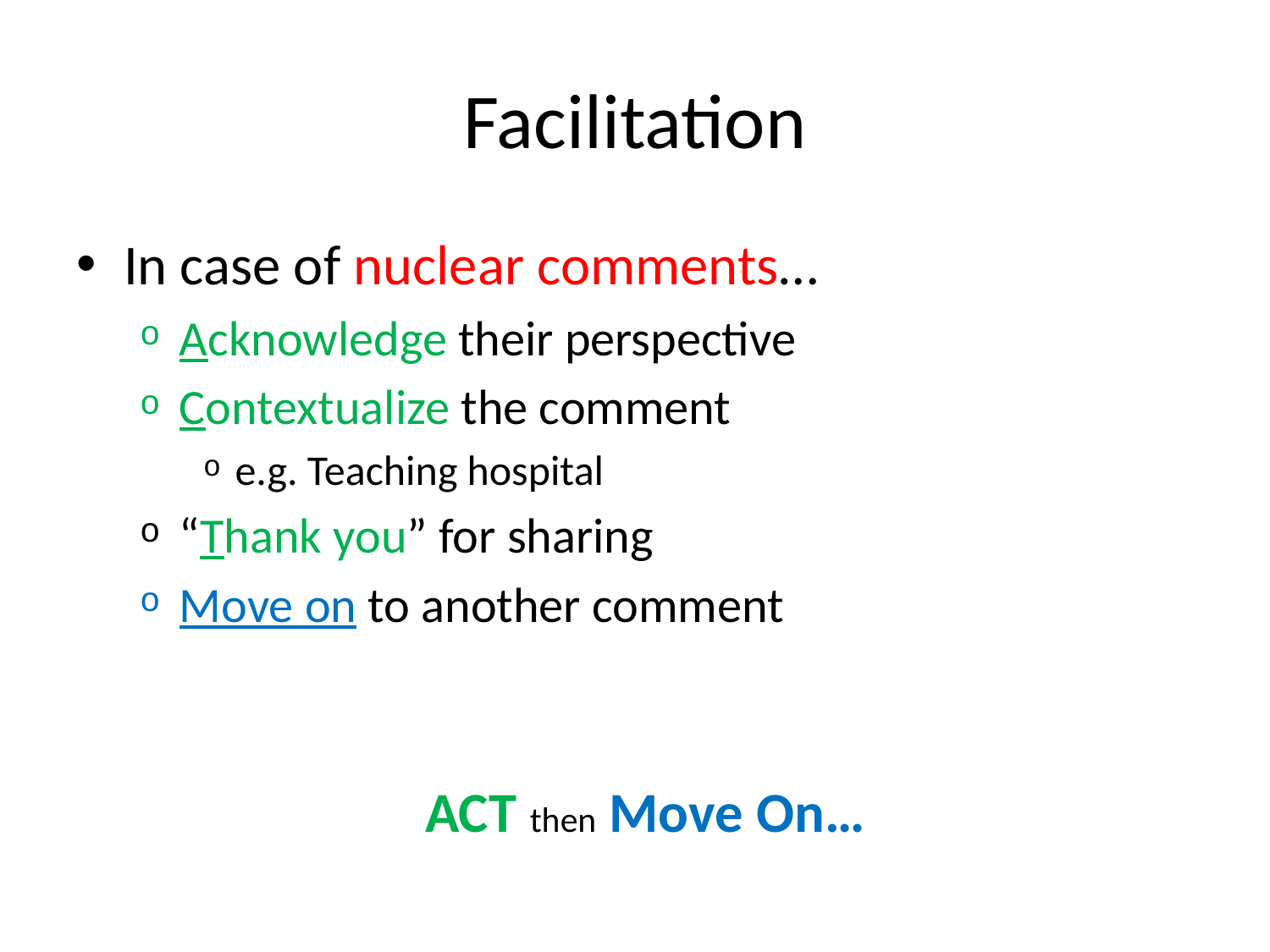

# Facilitation
In case of nuclear comments…
Acknowledge their perspective
Contextualize the comment
e.g. Teaching hospital
“Thank you” for sharing
Move on to another comment
ACT then Move On…

## Slide 56
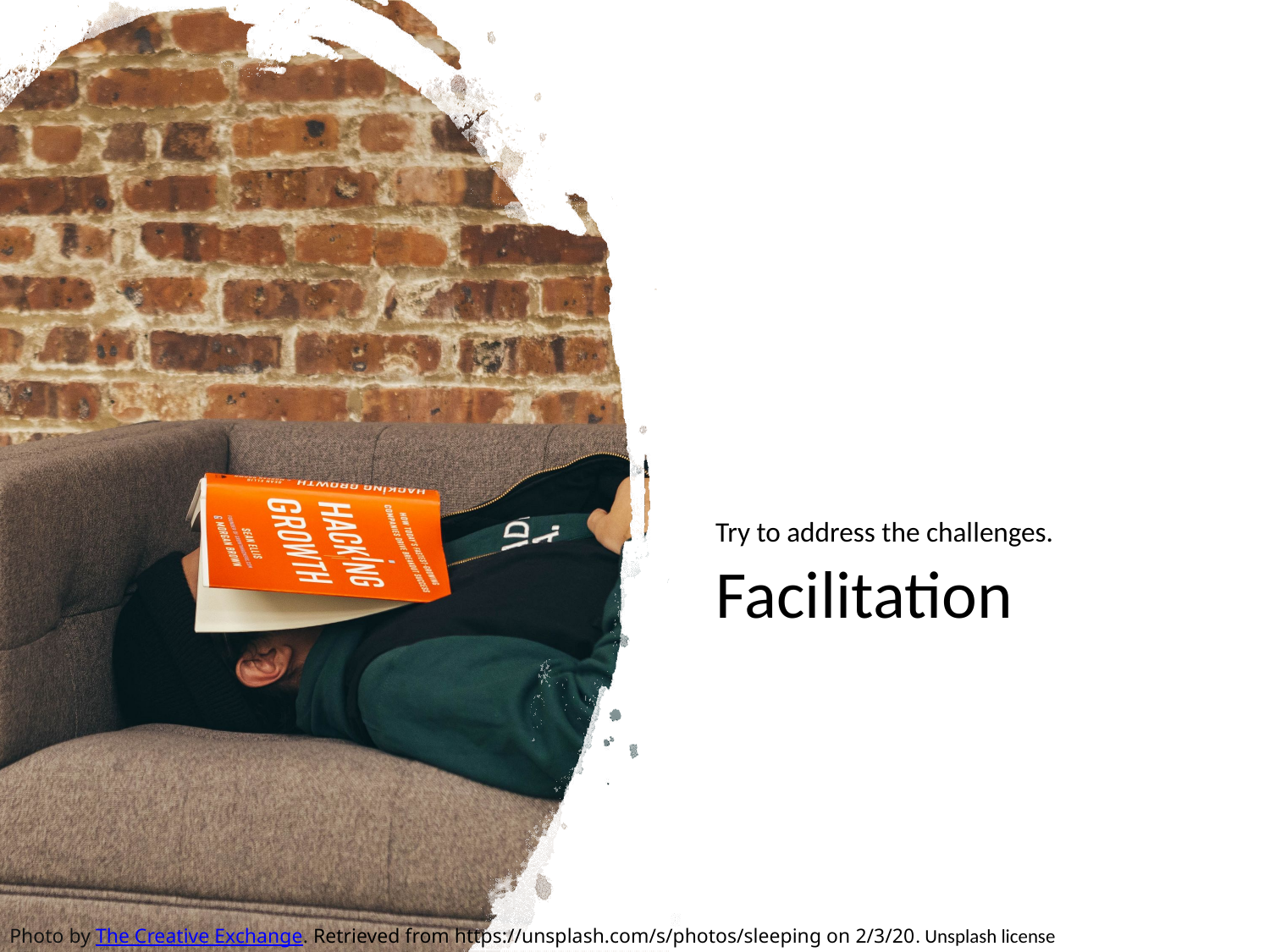

Try to address the challenges.
# Facilitation
Photo by The Creative Exchange. Retrieved from https://unsplash.com/s/photos/sleeping on 2/3/20. Unsplash license associated: https://unsplash.com/license

## Slide 57
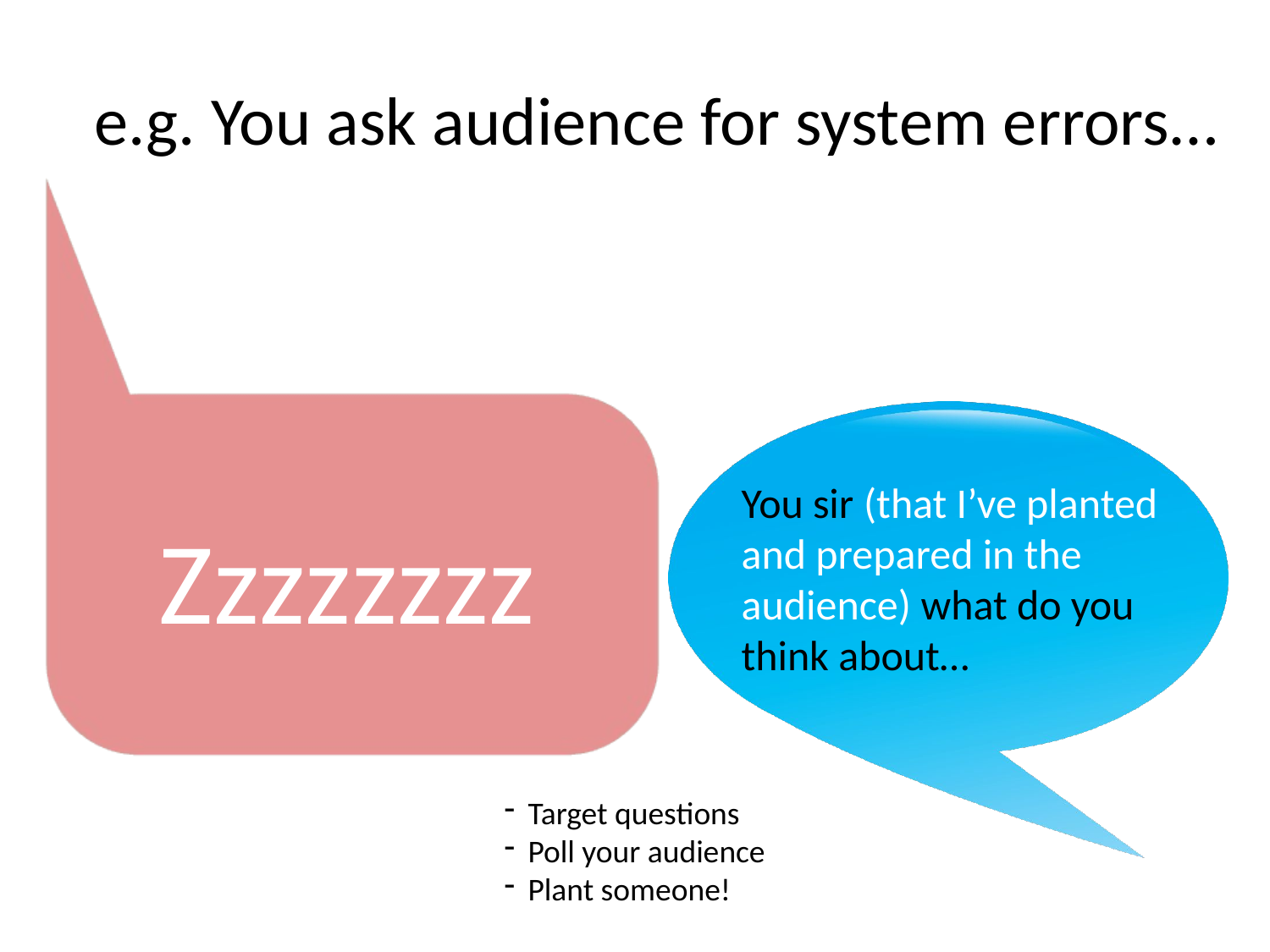

# e.g. You ask audience for system errors…
You sir (that I’ve planted and prepared in the audience) what do you think about…
Zzzzzzzz
Target questions
Poll your audience
Plant someone!

## Slide 58
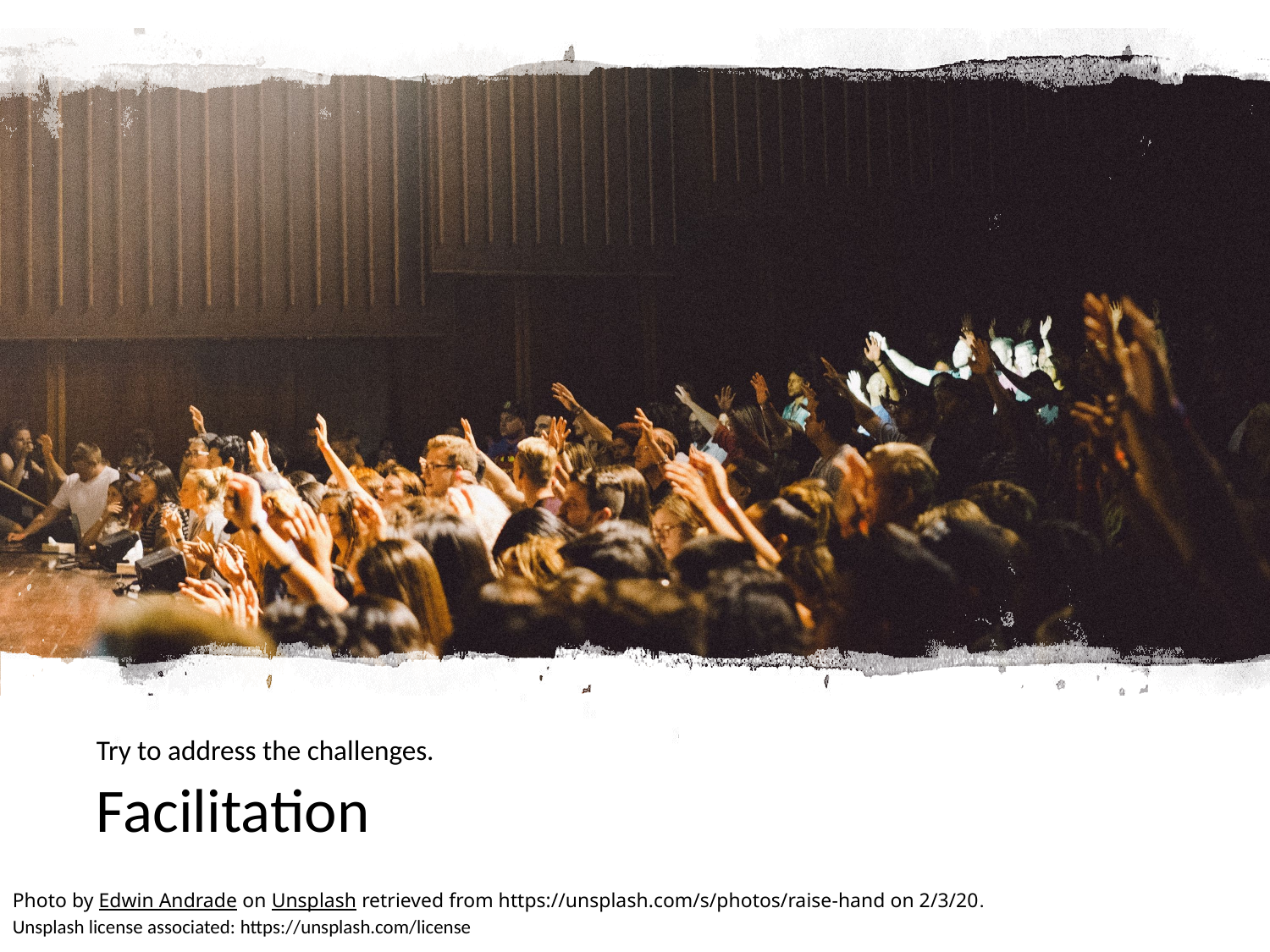

Try to address the challenges.
# Facilitation
Photo by Edwin Andrade on Unsplash retrieved from https://unsplash.com/s/photos/raise-hand on 2/3/20.
Unsplash license associated: https://unsplash.com/license

## Slide 59
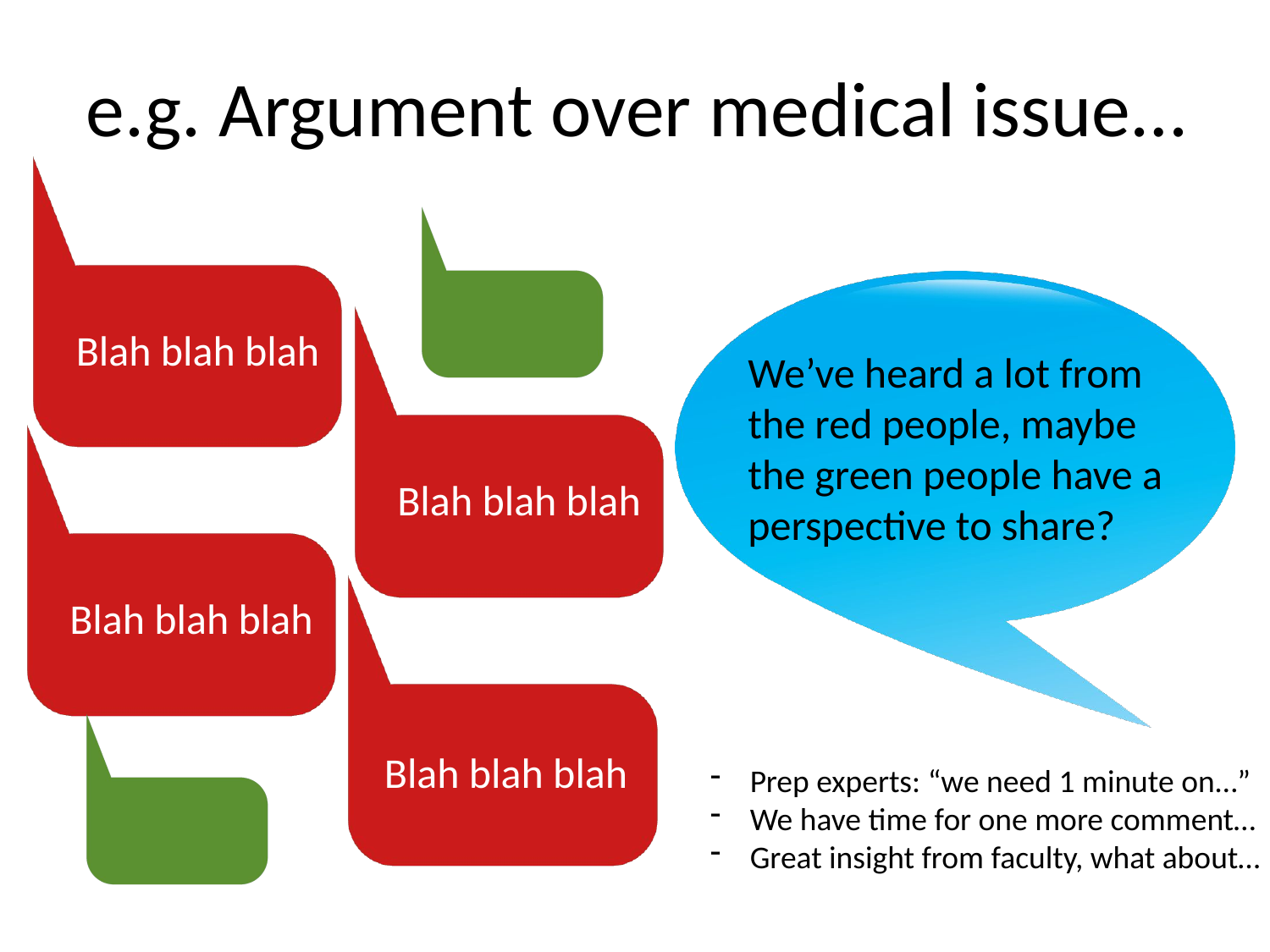

# e.g. Argument over medical issue…
We’ve heard a lot from the red people, maybe the green people have a perspective to share?
Blah blah blah
Blah blah blah
Blah blah blah
Blah blah blah
Prep experts: “we need 1 minute on…”
We have time for one more comment…
Great insight from faculty, what about…
Blah blah blah

## Slide 60
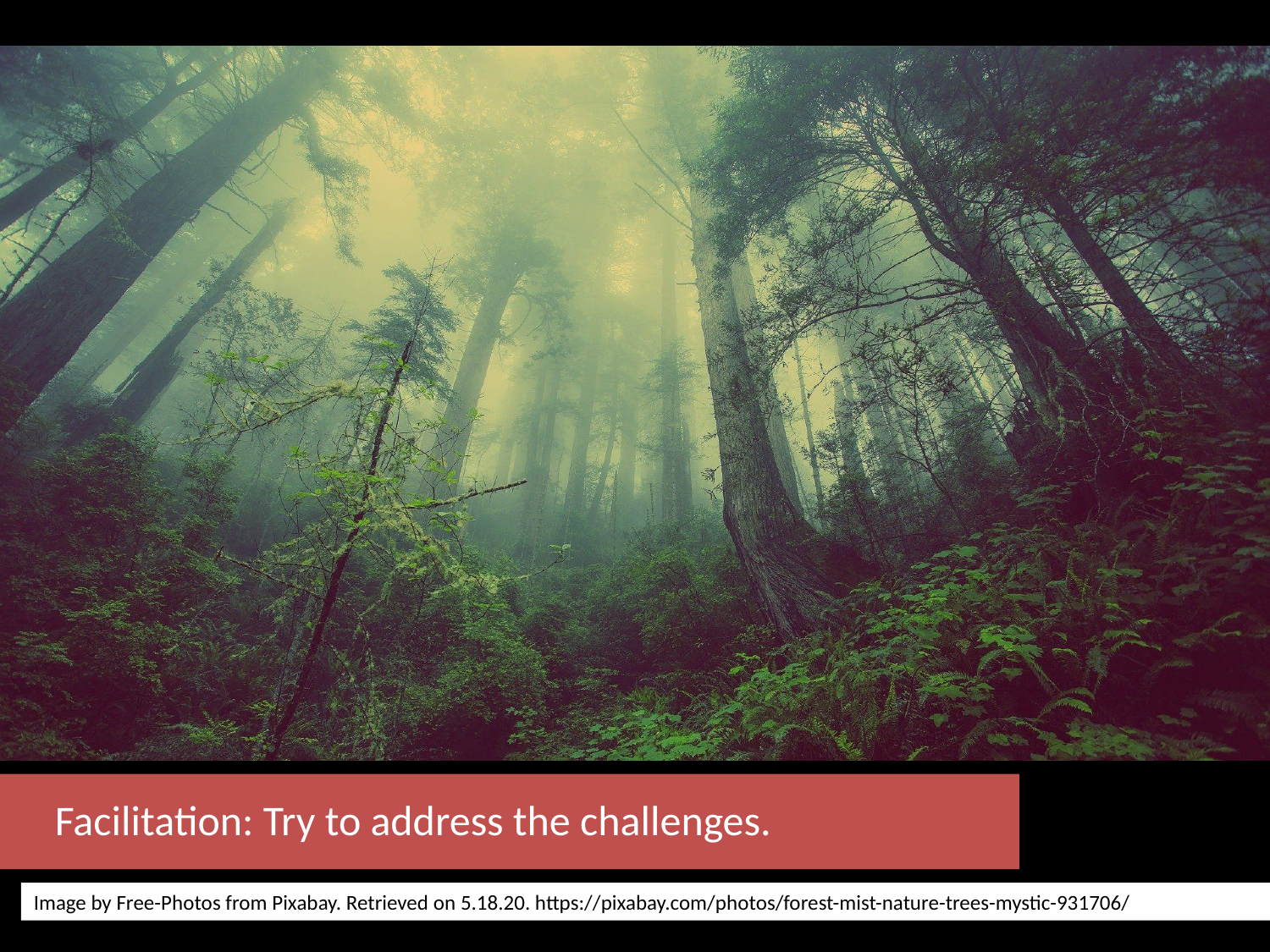

Facilitation: Try to address the challenges.
Image by Free-Photos from Pixabay. Retrieved on 5.18.20. https://pixabay.com/photos/forest-mist-nature-trees-mystic-931706/

## Slide 61
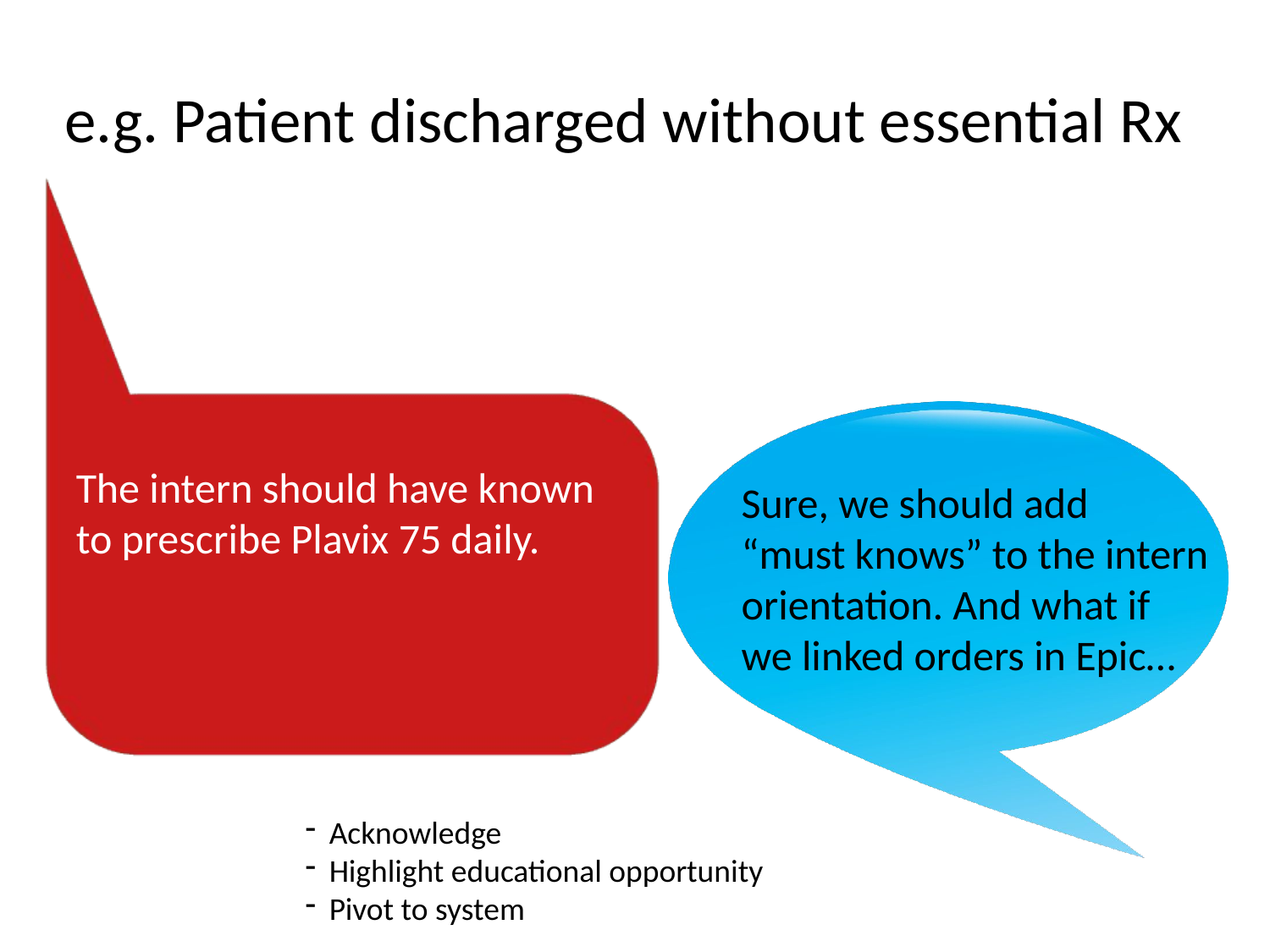

# e.g. Patient discharged without essential Rx
Sure, we should add
“must knows” to the intern orientation. And what if
we linked orders in Epic…
The intern should have known to prescribe Plavix 75 daily.
Acknowledge
Highlight educational opportunity
Pivot to system

## Slide 62
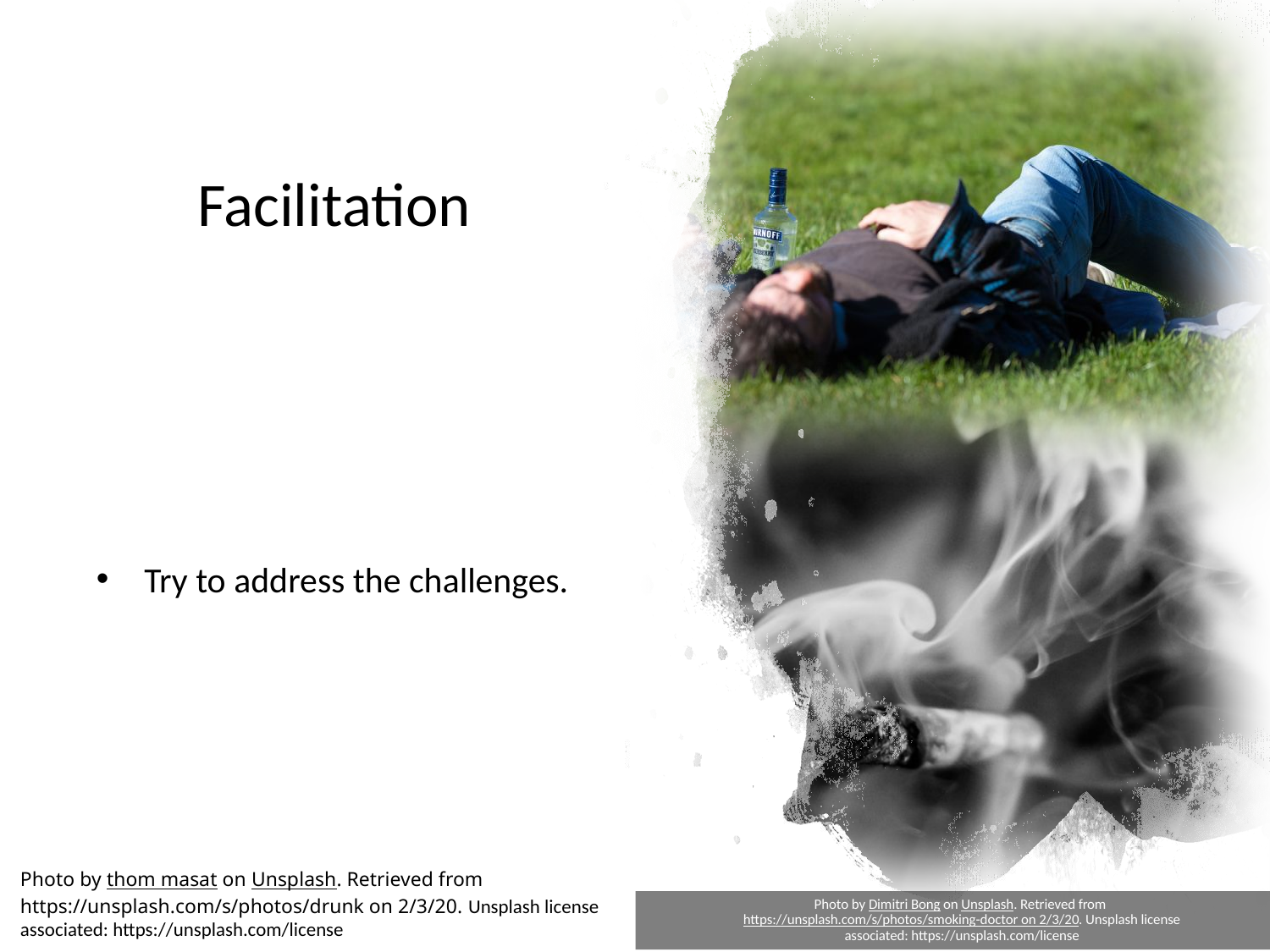

# Facilitation
Try to address the challenges.
Photo by thom masat on Unsplash. Retrieved from https://unsplash.com/s/photos/drunk on 2/3/20. Unsplash license associated: https://unsplash.com/license
Photo by Dimitri Bong on Unsplash. Retrieved from https://unsplash.com/s/photos/smoking-doctor on 2/3/20. Unsplash license associated: https://unsplash.com/license

## Slide 63
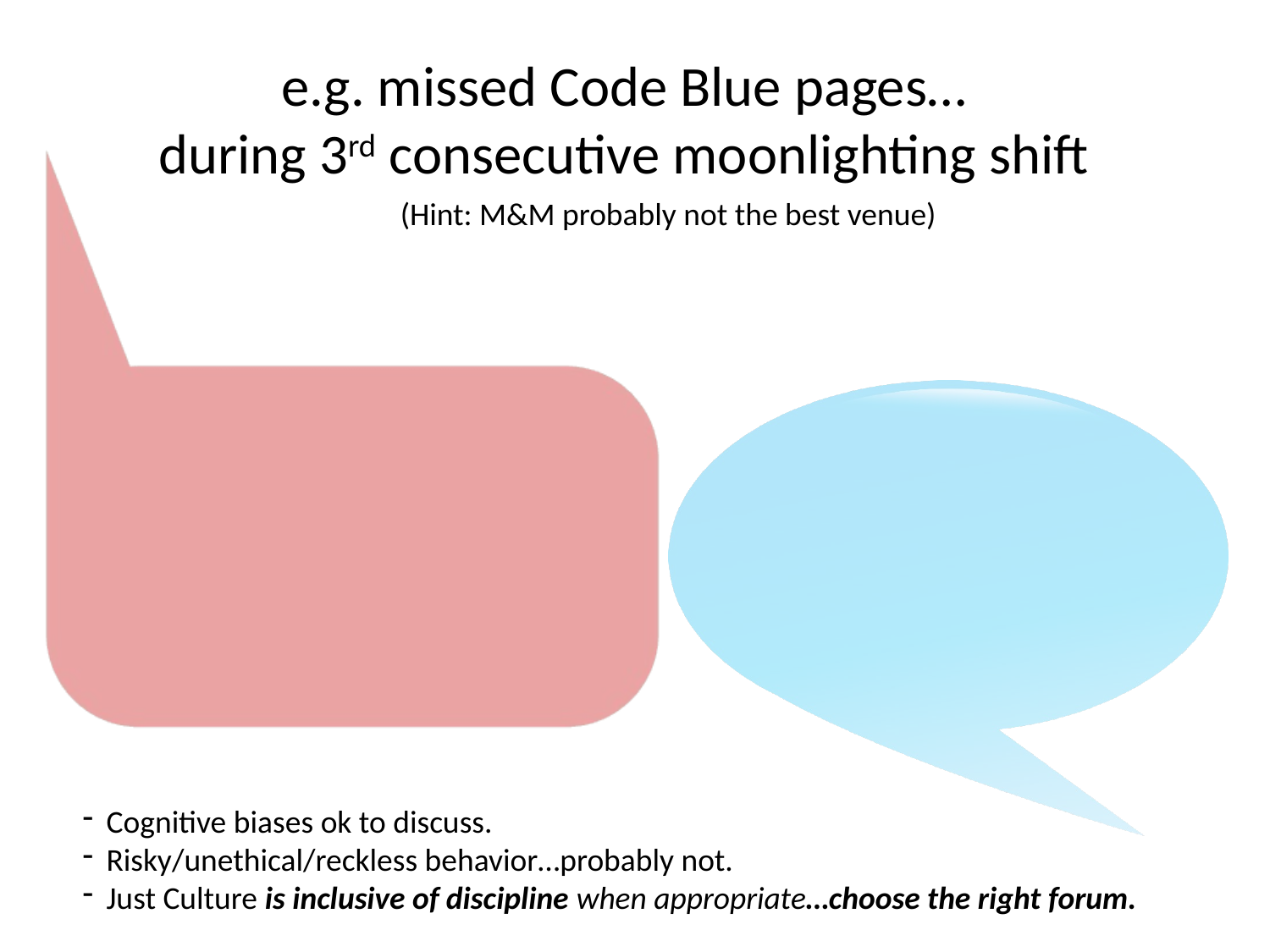

# e.g. missed Code Blue pages… during 3rd consecutive moonlighting shift
(Hint: M&M probably not the best venue)
Cognitive biases ok to discuss.
Risky/unethical/reckless behavior…probably not.
Just Culture is inclusive of discipline when appropriate…choose the right forum.

## Slide 64
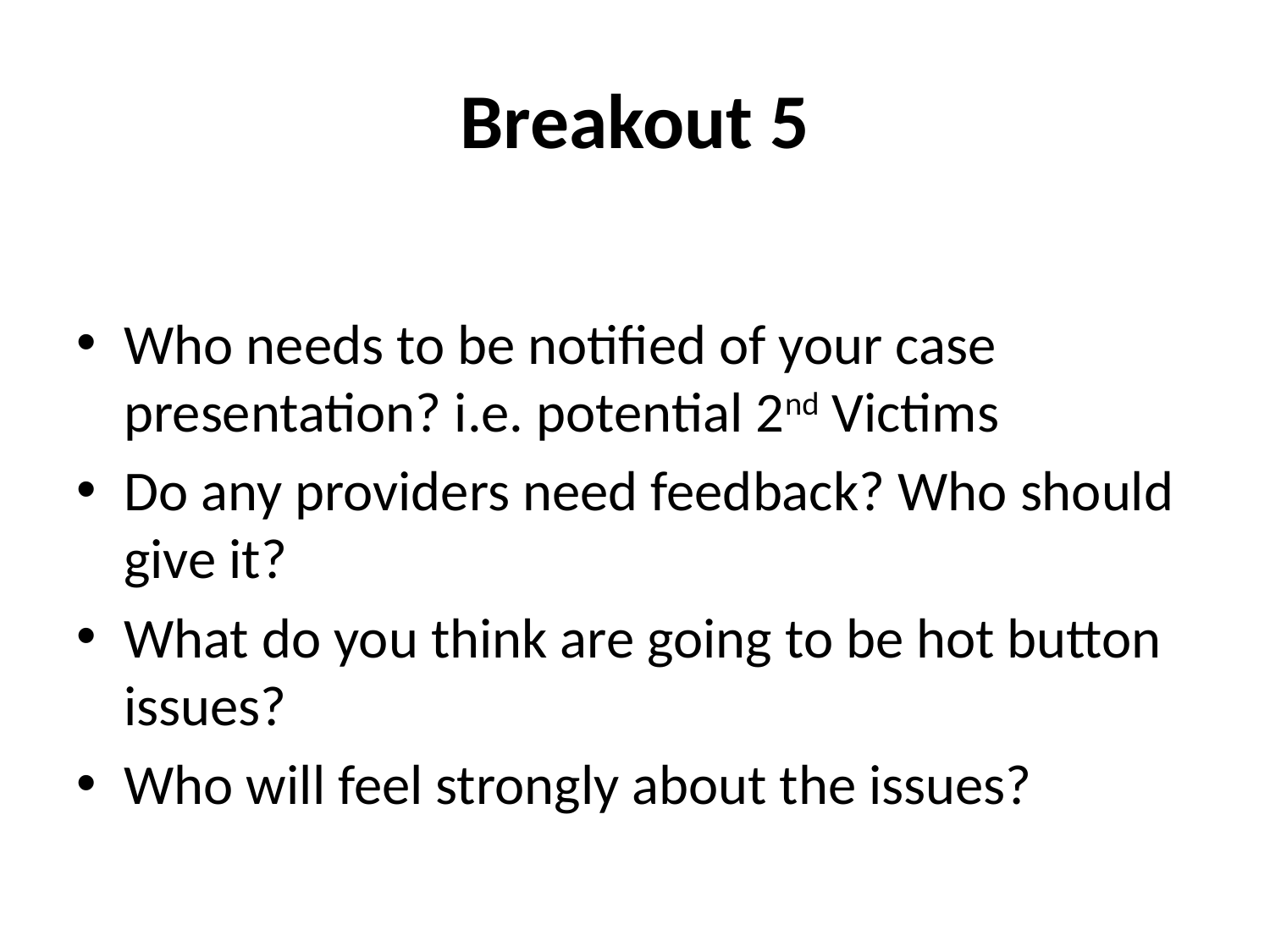

# Breakout 5
Who needs to be notified of your case presentation? i.e. potential 2nd Victims
Do any providers need feedback? Who should give it?
What do you think are going to be hot button issues?
Who will feel strongly about the issues?

## Slide 65
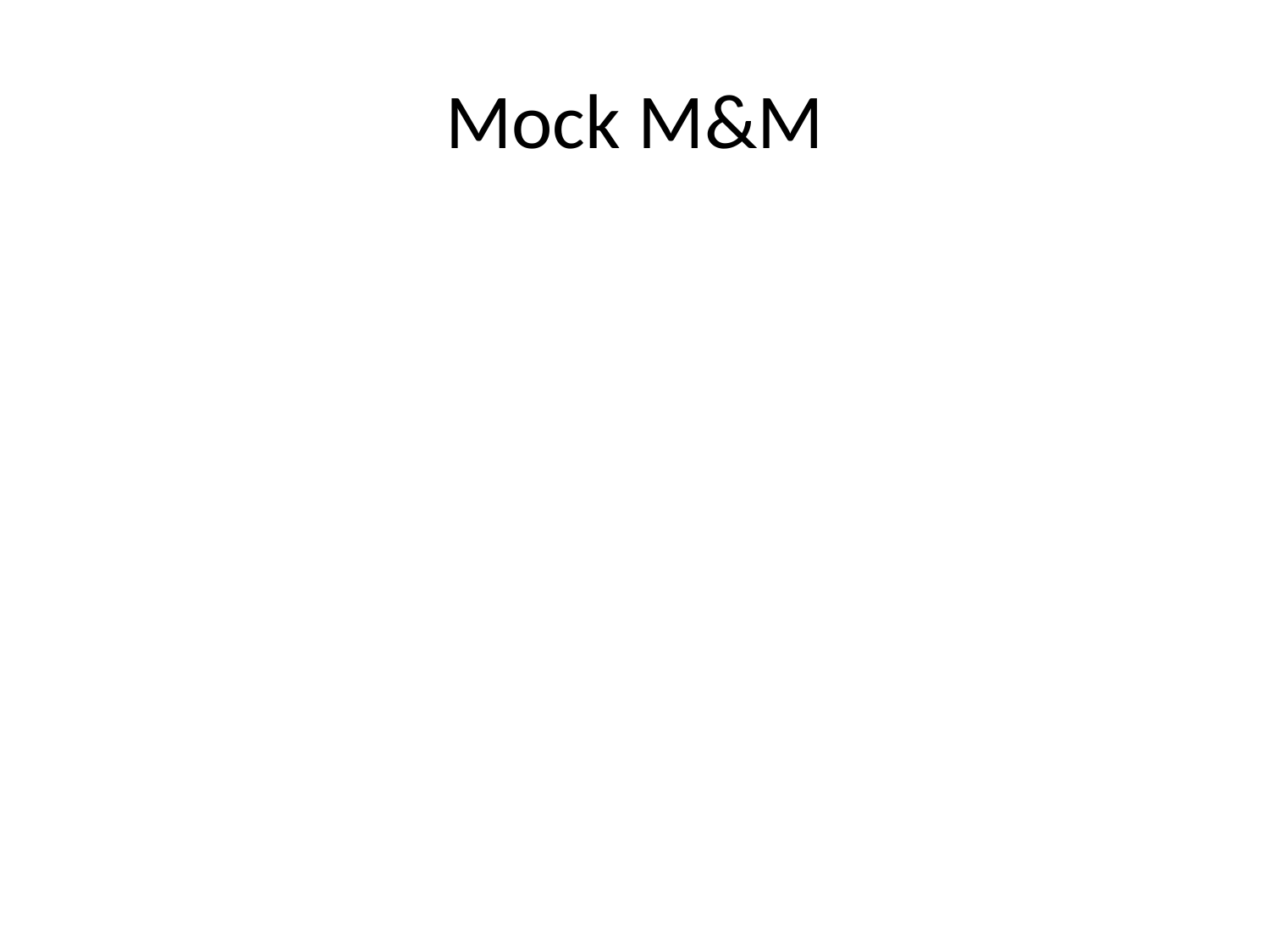

# Mock M&M

## Slide 66
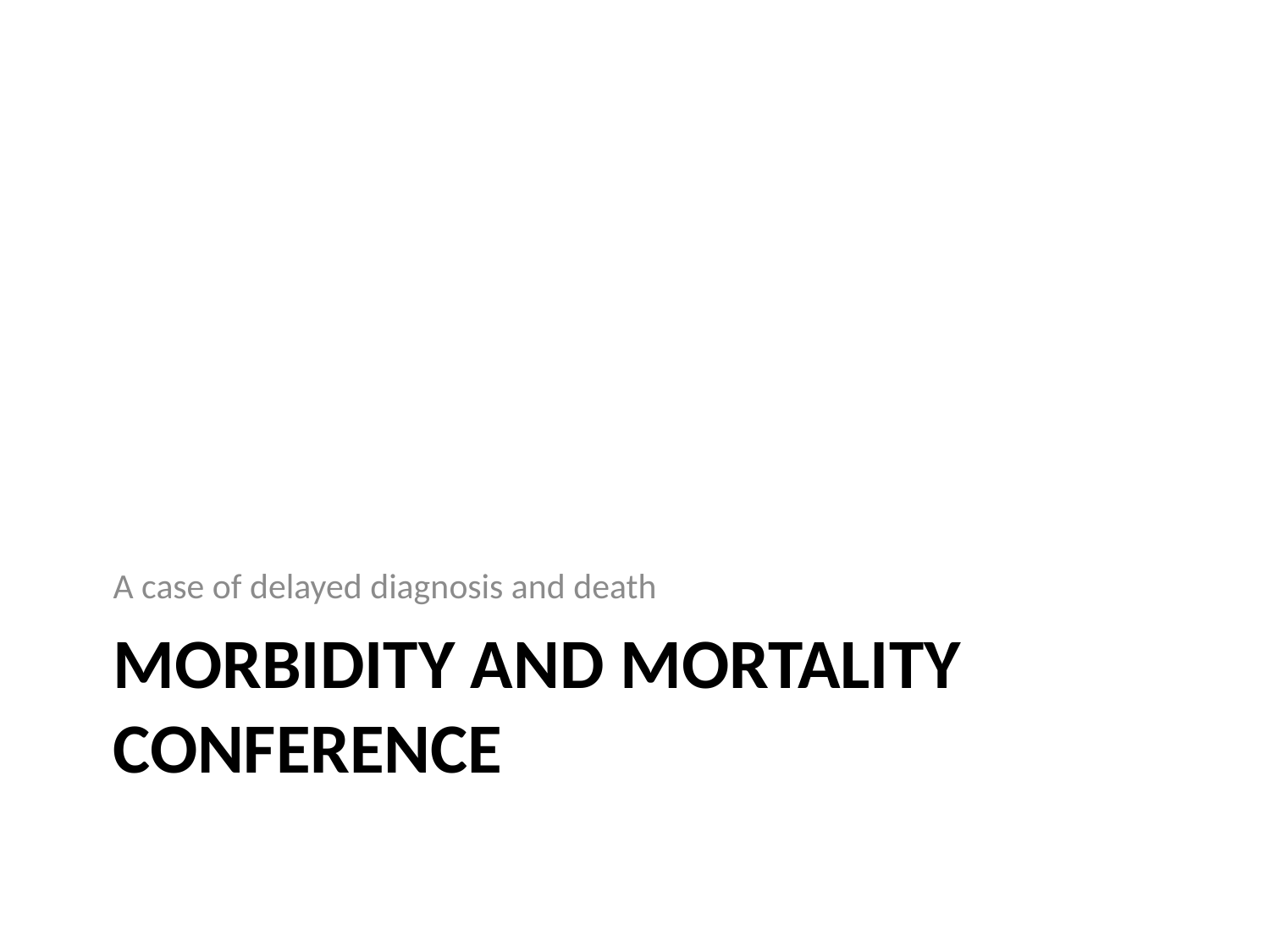

A case of delayed diagnosis and death
# Morbidity and mortality conference

## Slide 67
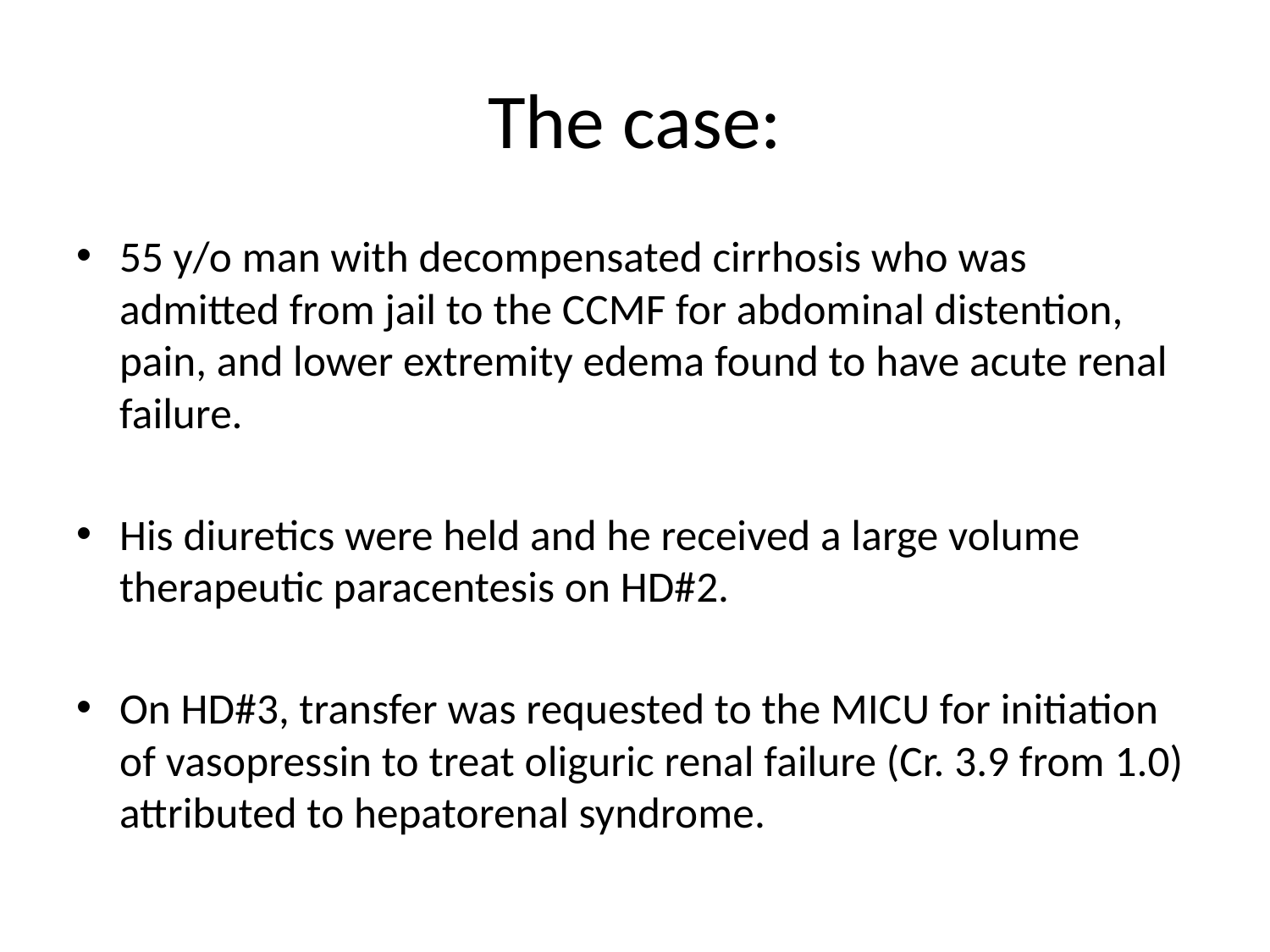

# The case:
55 y/o man with decompensated cirrhosis who was admitted from jail to the CCMF for abdominal distention, pain, and lower extremity edema found to have acute renal failure.
His diuretics were held and he received a large volume therapeutic paracentesis on HD#2.
On HD#3, transfer was requested to the MICU for initiation of vasopressin to treat oliguric renal failure (Cr. 3.9 from 1.0) attributed to hepatorenal syndrome.

## Slide 68
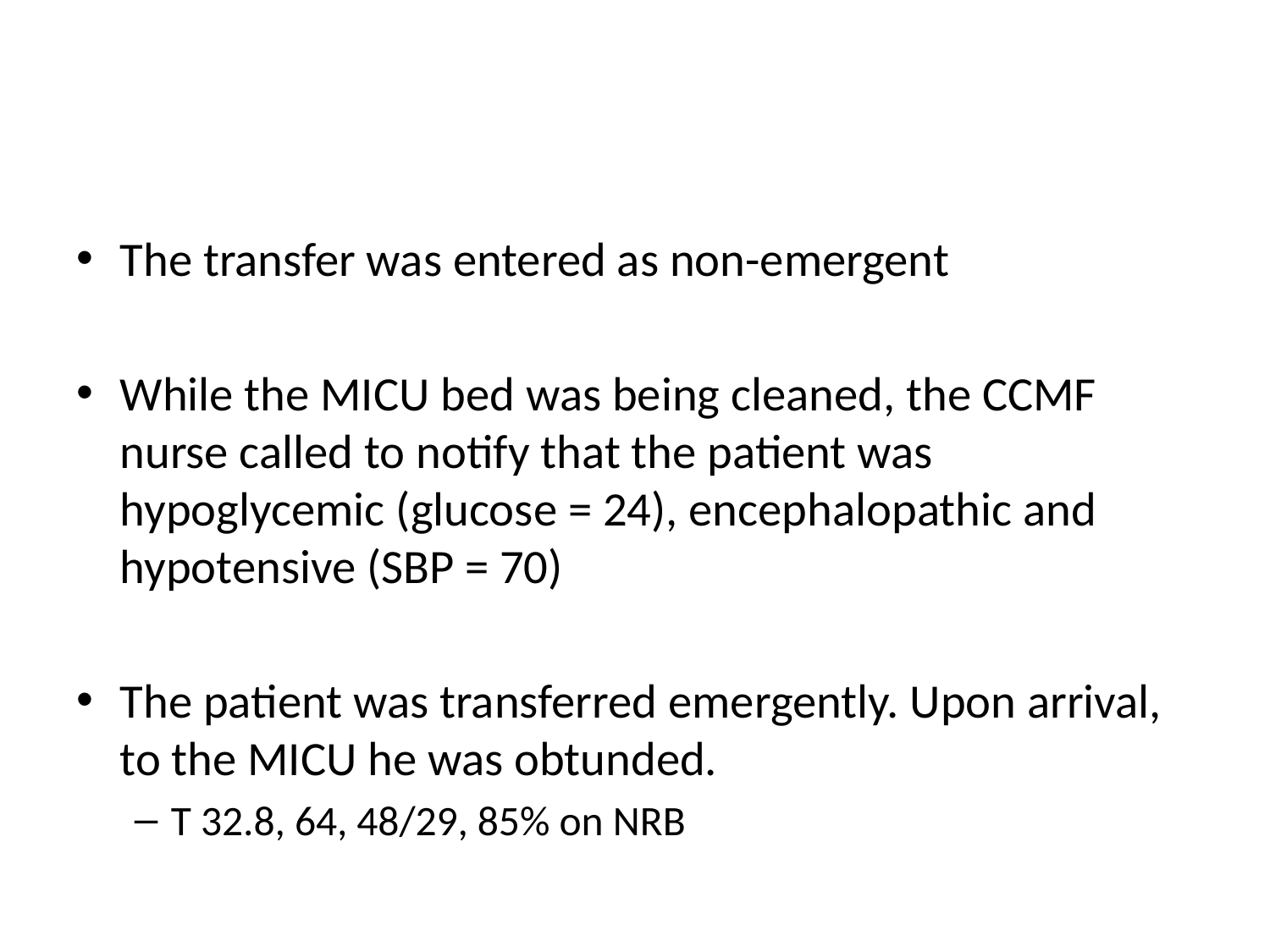

#
The transfer was entered as non-emergent
While the MICU bed was being cleaned, the CCMF nurse called to notify that the patient was hypoglycemic (glucose = 24), encephalopathic and hypotensive (SBP = 70)
The patient was transferred emergently. Upon arrival, to the MICU he was obtunded.
T 32.8, 64, 48/29, 85% on NRB

## Slide 69
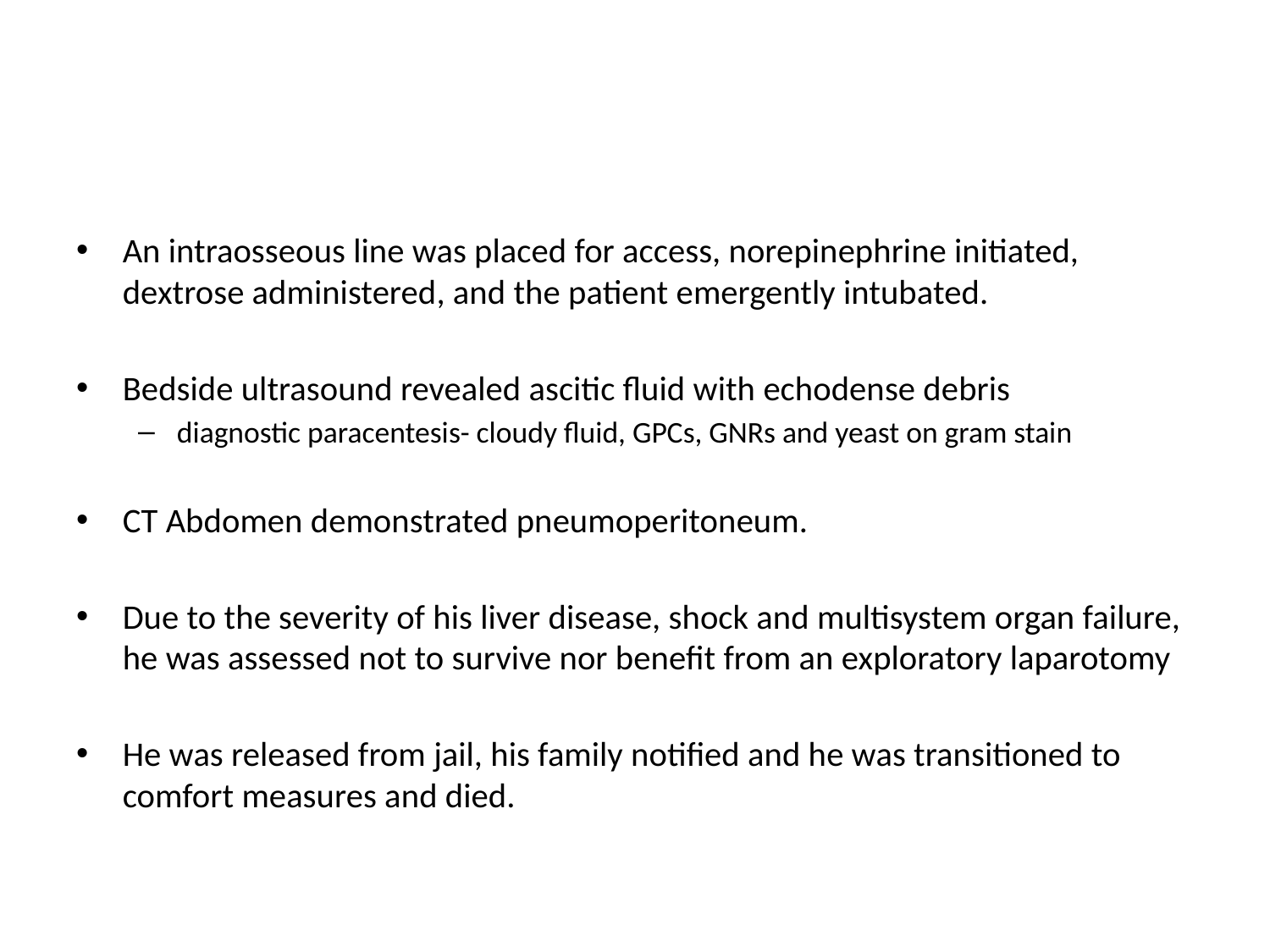

#
An intraosseous line was placed for access, norepinephrine initiated, dextrose administered, and the patient emergently intubated.
Bedside ultrasound revealed ascitic fluid with echodense debris
diagnostic paracentesis- cloudy fluid, GPCs, GNRs and yeast on gram stain
CT Abdomen demonstrated pneumoperitoneum.
Due to the severity of his liver disease, shock and multisystem organ failure, he was assessed not to survive nor benefit from an exploratory laparotomy
He was released from jail, his family notified and he was transitioned to comfort measures and died.

## Slide 70
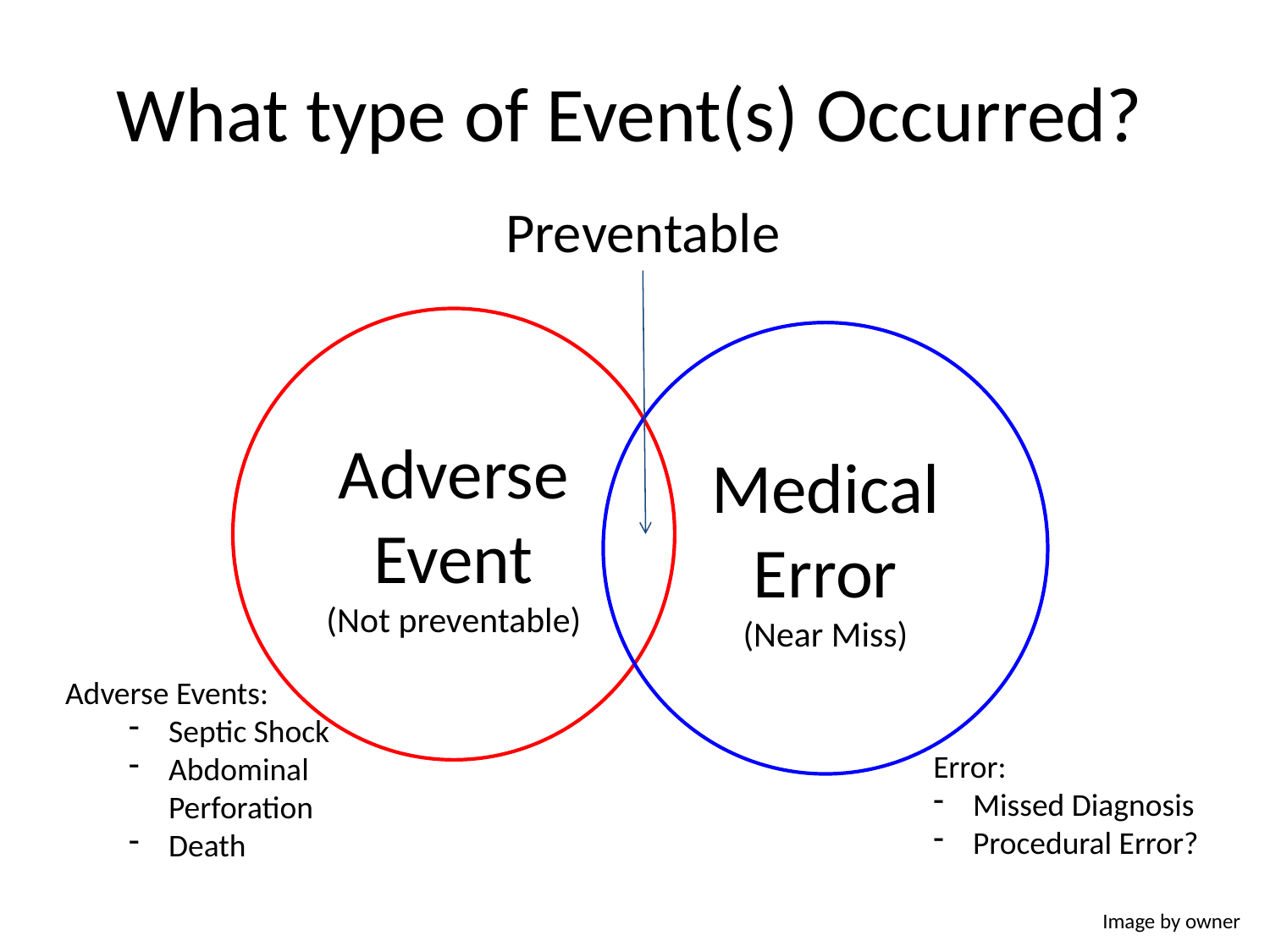

What type of Event(s) Occurred?
Preventable
Adverse Event
(Not preventable)
Medical Error
(Near Miss)
Adverse Events:
Septic Shock
Abdominal Perforation
Death
Error:
Missed Diagnosis
Procedural Error?
Image by owner

## Slide 71
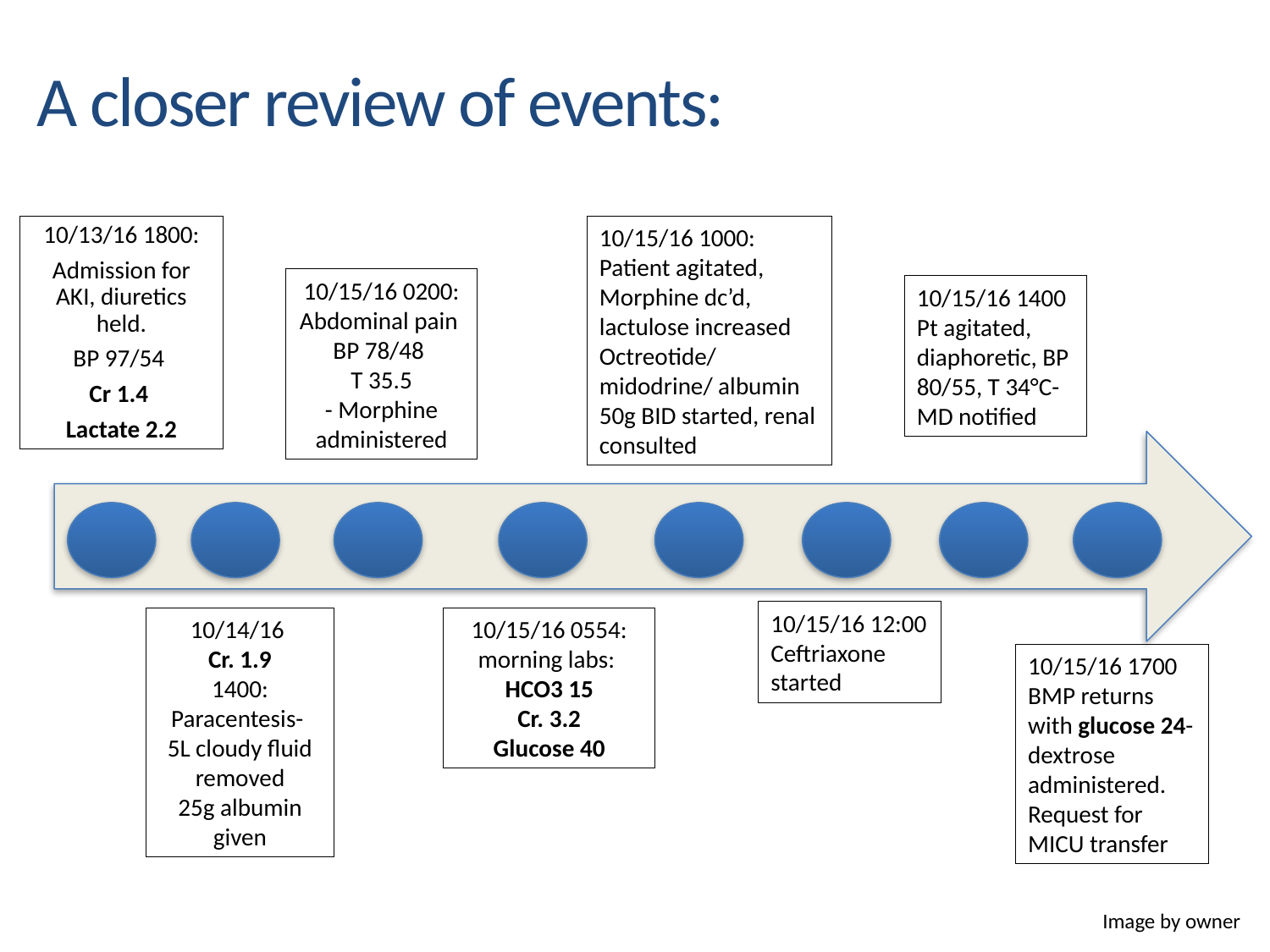

A closer review of events:
10/13/16 1800:
Admission for AKI, diuretics held.
BP 97/54
Cr 1.4
Lactate 2.2
10/15/16 1000:
Patient agitated, Morphine dc’d,
lactulose increased
Octreotide/midodrine/ albumin 50g BID started, renal consulted
10/15/16 0200:
Abdominal pain
BP 78/48
T 35.5
- Morphine administered
10/15/16 1400
Pt agitated, diaphoretic, BP 80/55, T 34°C- MD notified
10/15/16 12:00 Ceftriaxone started
10/14/16
Cr. 1.9
1400:
Paracentesis-
5L cloudy fluid removed
25g albumin given
10/15/16 0554: morning labs:
HCO3 15
Cr. 3.2
Glucose 40
10/15/16 1700
BMP returns with glucose 24- dextrose administered. Request for MICU transfer
Image by owner

## Slide 72
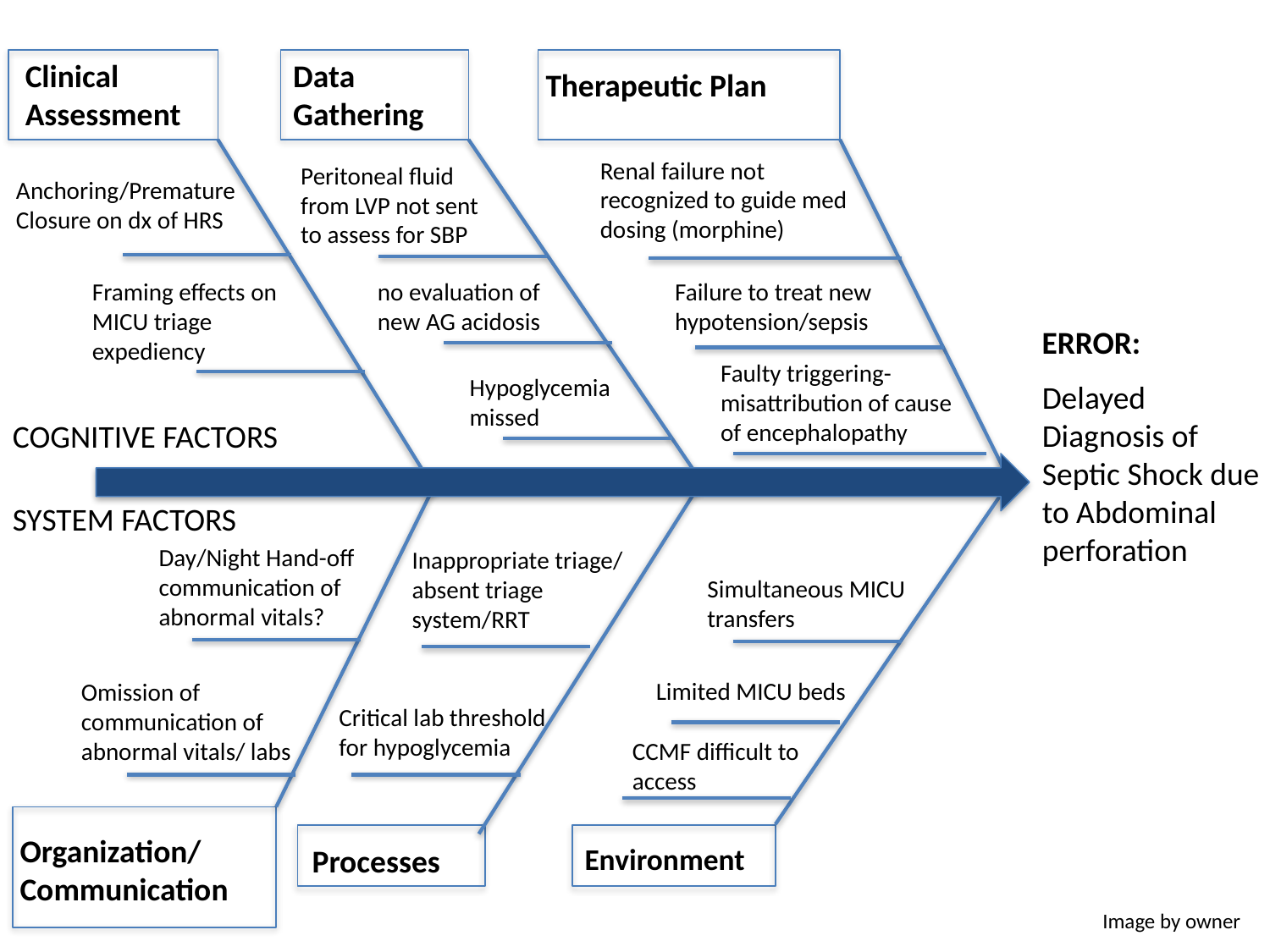

Clinical Assessment
Data Gathering
Therapeutic Plan
Renal failure not recognized to guide med dosing (morphine)
Peritoneal fluid from LVP not sent to assess for SBP
Anchoring/Premature Closure on dx of HRS
Framing effects on MICU triage expediency
no evaluation of new AG acidosis
Failure to treat new hypotension/sepsis
ERROR:
Faulty triggering- misattribution of cause of encephalopathy
Hypoglycemia missed
Delayed Diagnosis of Septic Shock due to Abdominal perforation
COGNITIVE FACTORS
SYSTEM FACTORS
Day/Night Hand-off communication of abnormal vitals?
Inappropriate triage/ absent triage system/RRT
Simultaneous MICU transfers
Limited MICU beds
Omission of communication of abnormal vitals/ labs
Critical lab threshold for hypoglycemia
CCMF difficult to access
Organization/Communication
Environment
Processes
Image by owner

## Slide 73
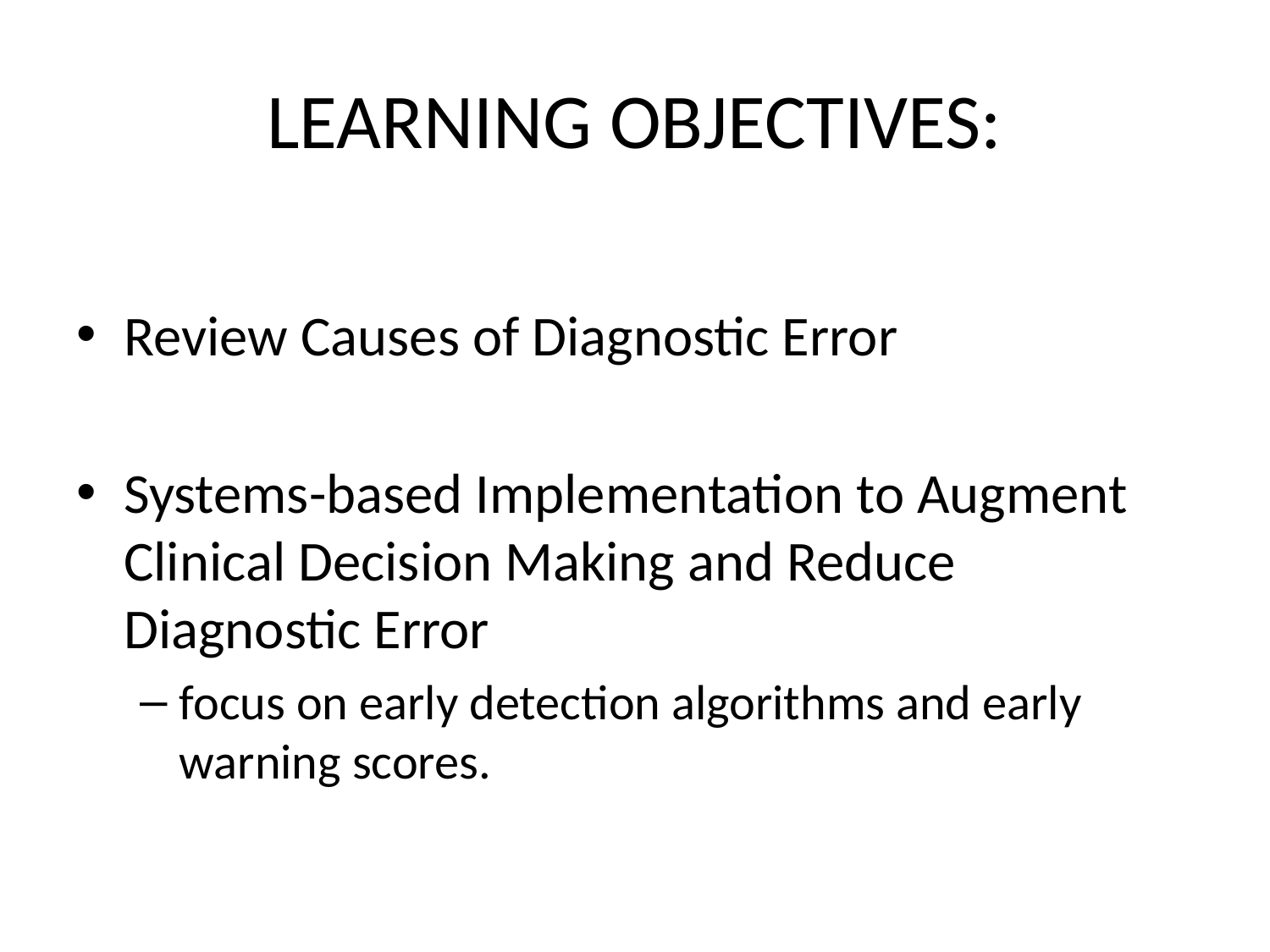

# LEARNING OBJECTIVES:
Review Causes of Diagnostic Error
Systems-based Implementation to Augment Clinical Decision Making and Reduce Diagnostic Error
focus on early detection algorithms and early warning scores.

## Slide 74
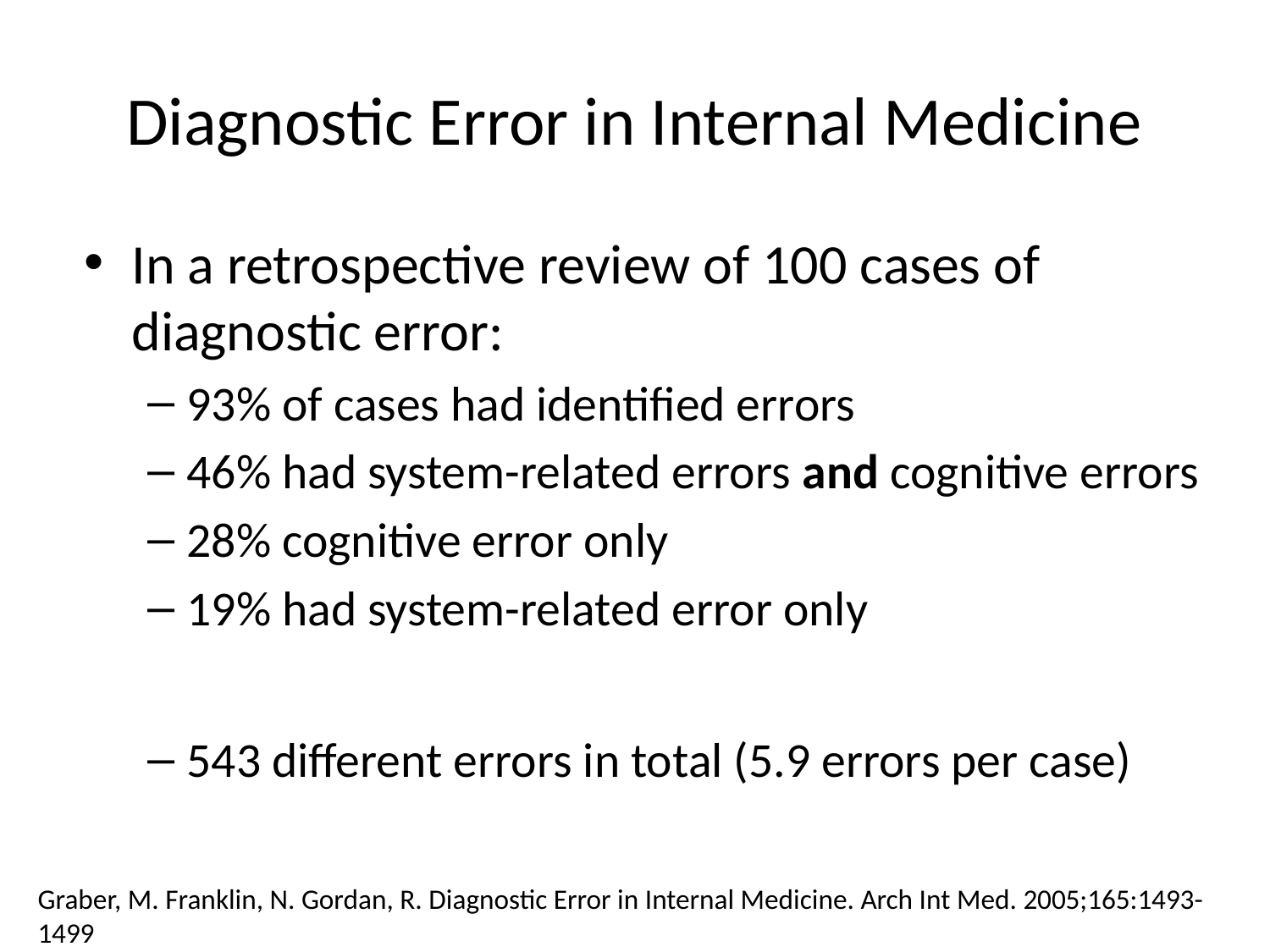

# Diagnostic Error in Internal Medicine
In a retrospective review of 100 cases of diagnostic error:
93% of cases had identified errors
46% had system-related errors and cognitive errors
28% cognitive error only
19% had system-related error only
543 different errors in total (5.9 errors per case)
Graber, M. Franklin, N. Gordan, R. Diagnostic Error in Internal Medicine. Arch Int Med. 2005;165:1493-1499

## Slide 75
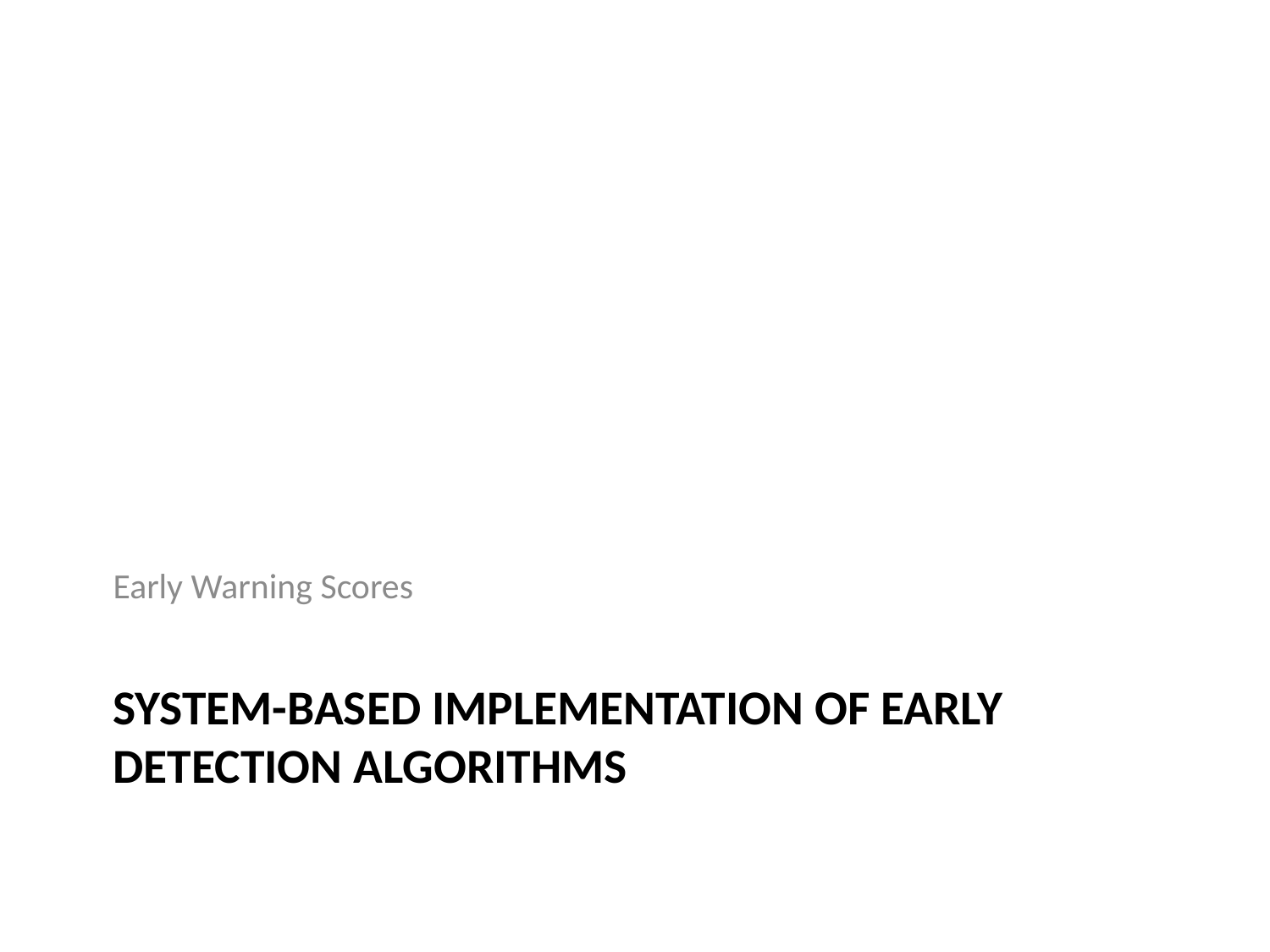

Early Warning Scores
# System-based implementation of early detection algorithms

## Slide 76
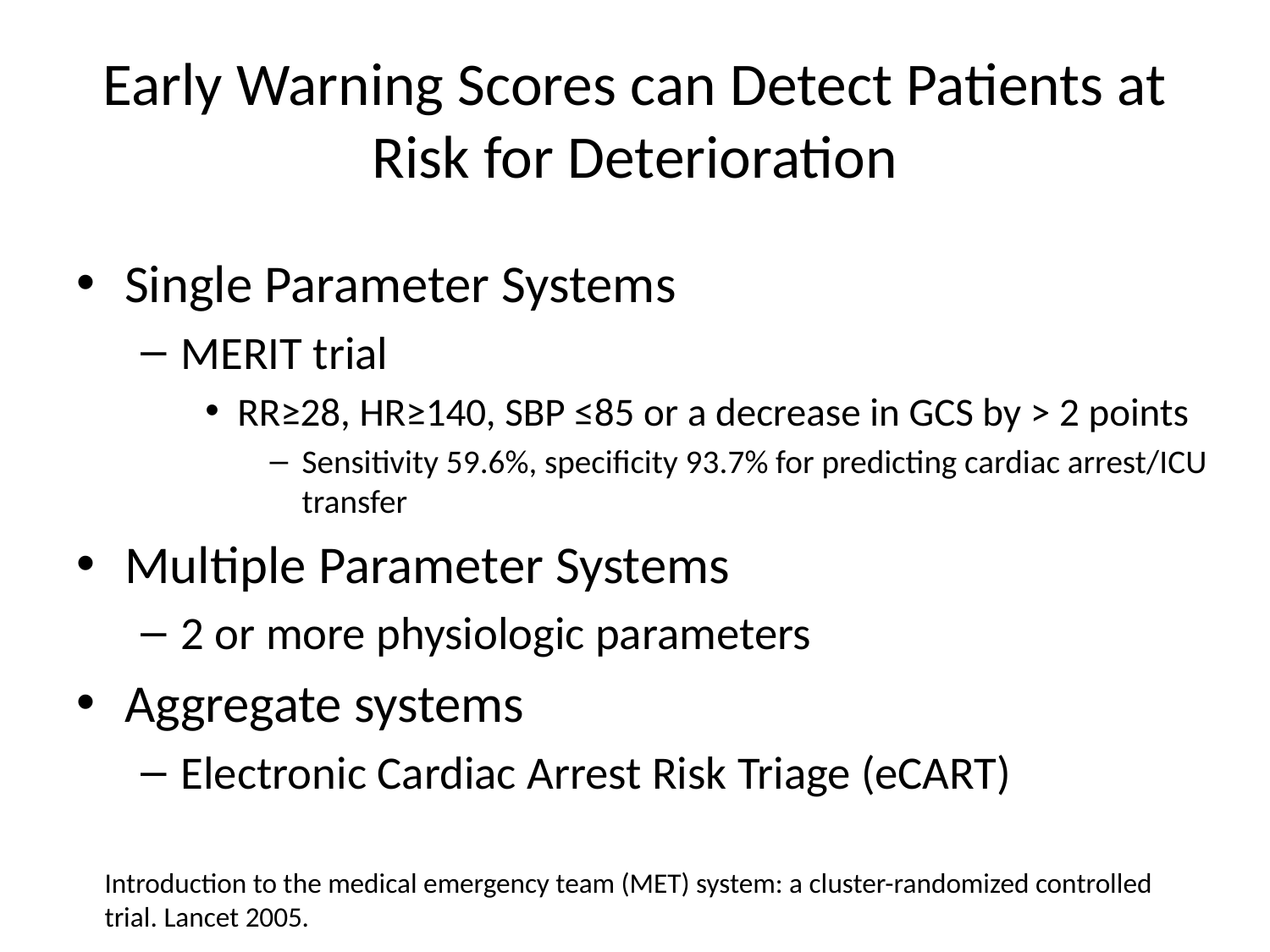

# Early Warning Scores can Detect Patients at Risk for Deterioration
Single Parameter Systems
MERIT trial
RR≥28, HR≥140, SBP ≤85 or a decrease in GCS by > 2 points
Sensitivity 59.6%, specificity 93.7% for predicting cardiac arrest/ICU transfer
Multiple Parameter Systems
2 or more physiologic parameters
Aggregate systems
Electronic Cardiac Arrest Risk Triage (eCART)
Introduction to the medical emergency team (MET) system: a cluster-randomized controlled trial. Lancet 2005.

## Slide 77
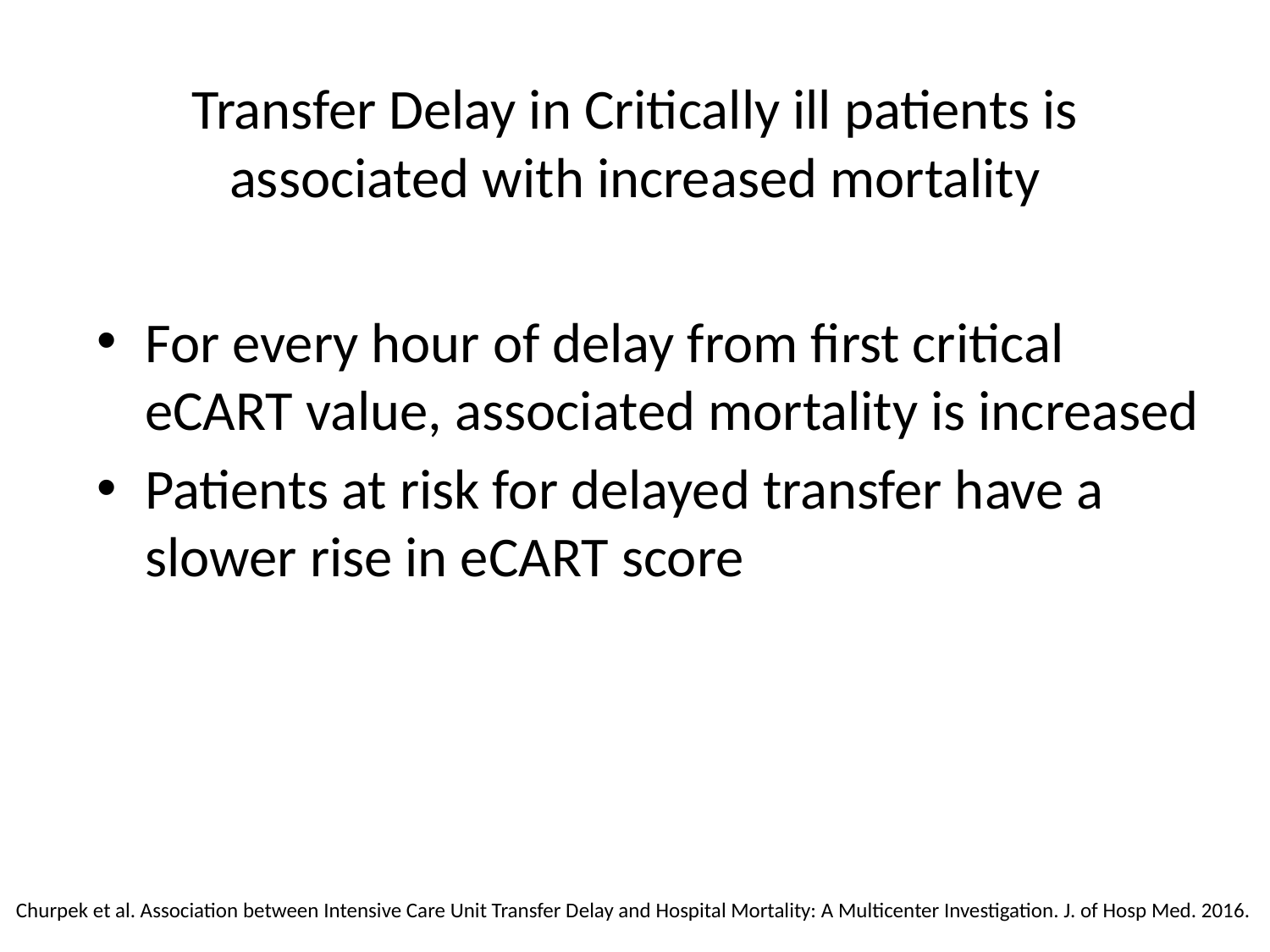

# Transfer Delay in Critically ill patients is associated with increased mortality
For every hour of delay from first critical eCART value, associated mortality is increased
Patients at risk for delayed transfer have a slower rise in eCART score
Churpek et al. Association between Intensive Care Unit Transfer Delay and Hospital Mortality: A Multicenter Investigation. J. of Hosp Med. 2016.

## Slide 78
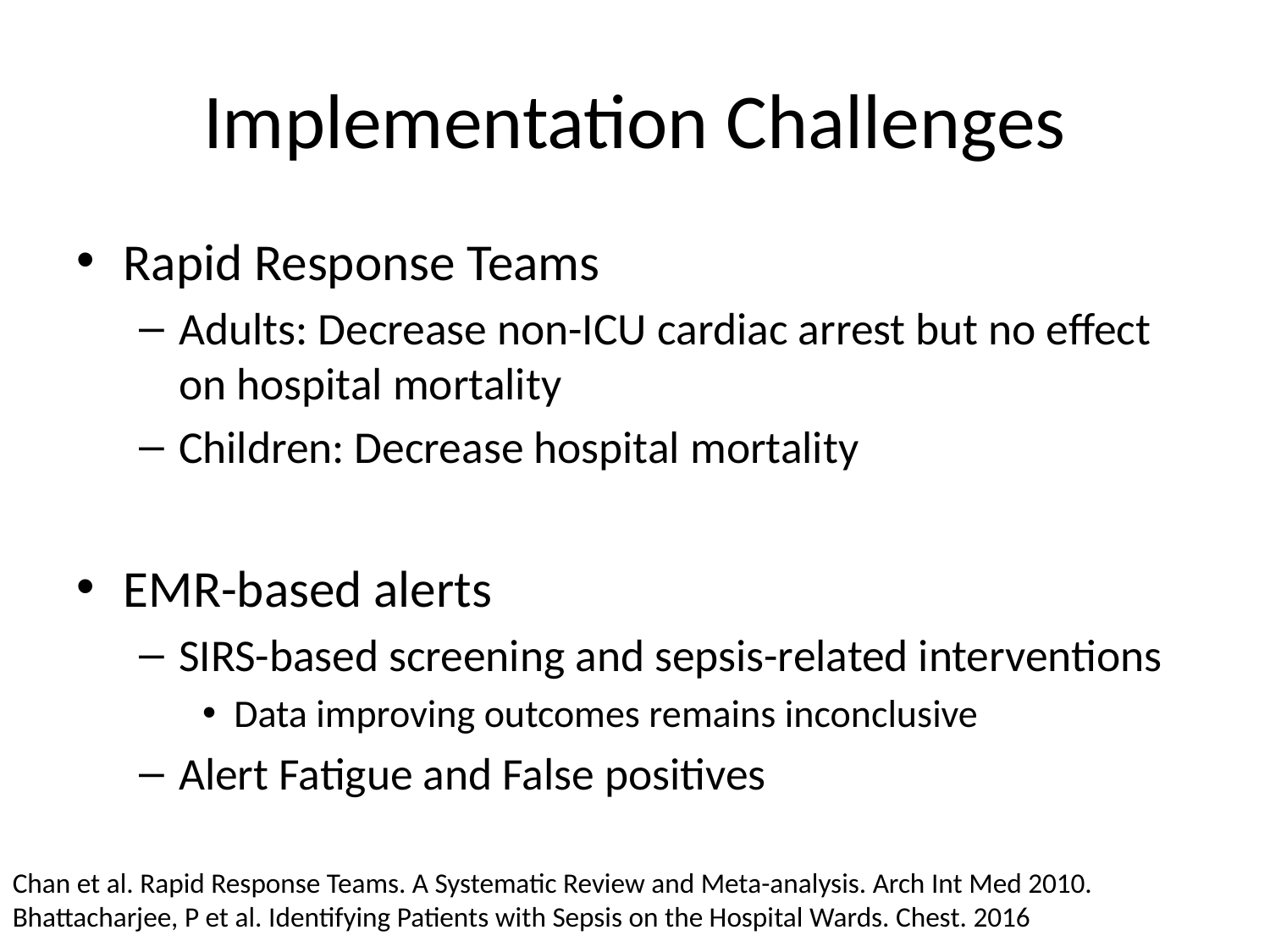

# Implementation Challenges
Rapid Response Teams
Adults: Decrease non-ICU cardiac arrest but no effect on hospital mortality
Children: Decrease hospital mortality
EMR-based alerts
SIRS-based screening and sepsis-related interventions
Data improving outcomes remains inconclusive
Alert Fatigue and False positives
Chan et al. Rapid Response Teams. A Systematic Review and Meta-analysis. Arch Int Med 2010.
Bhattacharjee, P et al. Identifying Patients with Sepsis on the Hospital Wards. Chest. 2016

## Slide 79
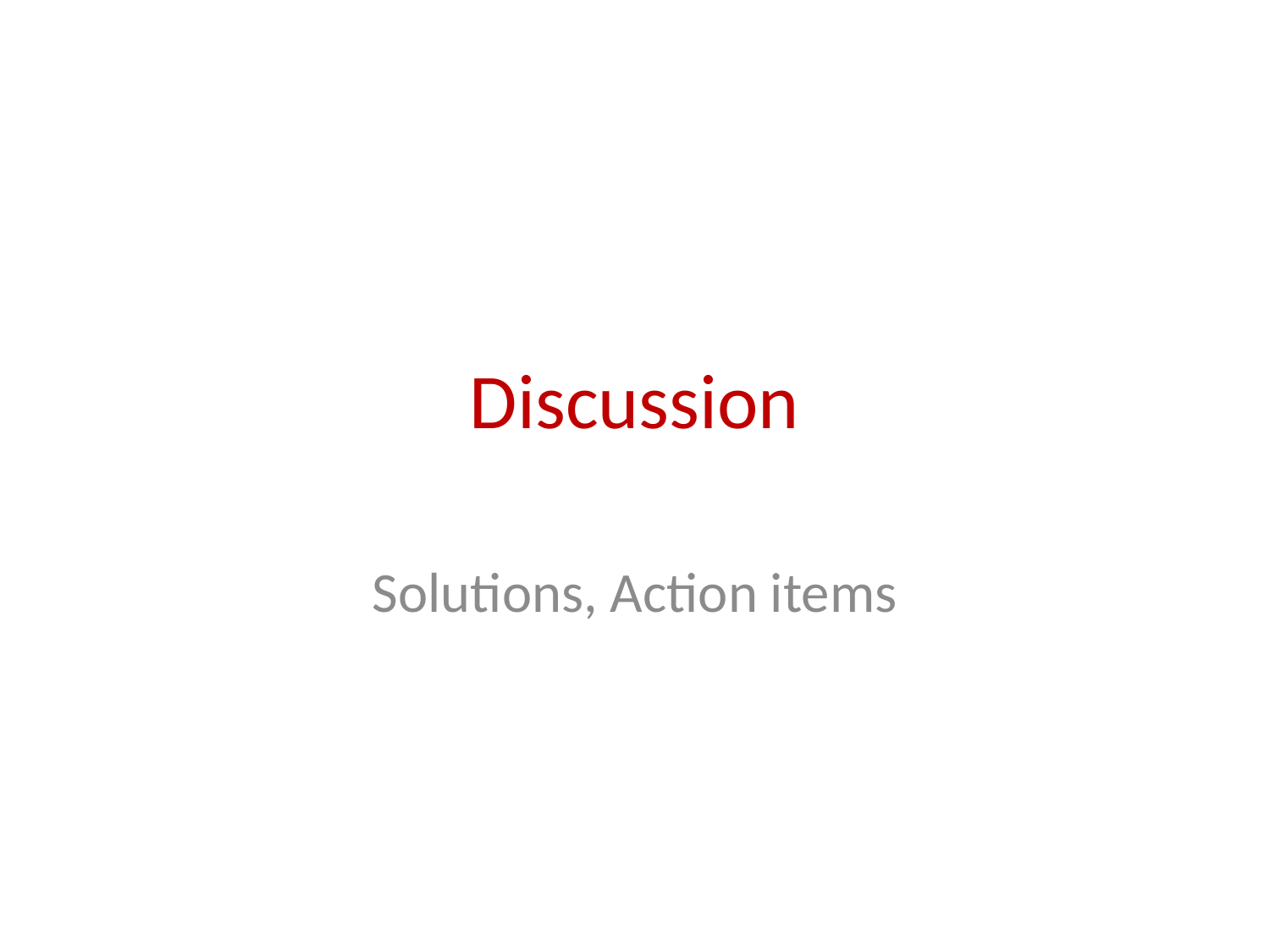

# Discussion
Solutions, Action items

## Slide 80
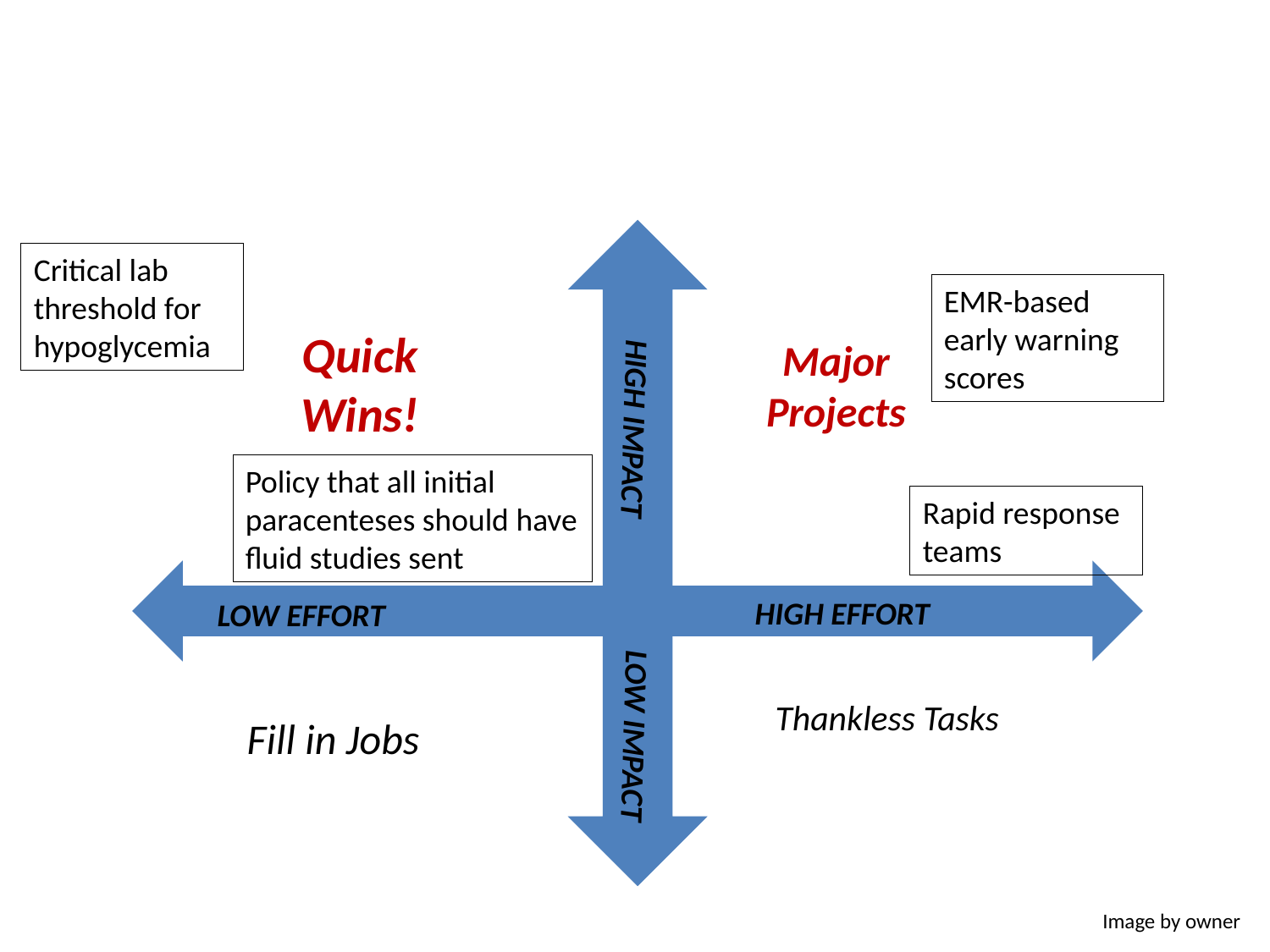

#
Critical lab threshold for hypoglycemia
EMR-based early warning scores
Quick Wins!
Major Projects
HIGH IMPACT
Policy that all initial paracenteses should have fluid studies sent
Rapid response teams
HIGH EFFORT
LOW EFFORT
Thankless Tasks
LOW IMPACT
Fill in Jobs
Image by owner

## Slide 81
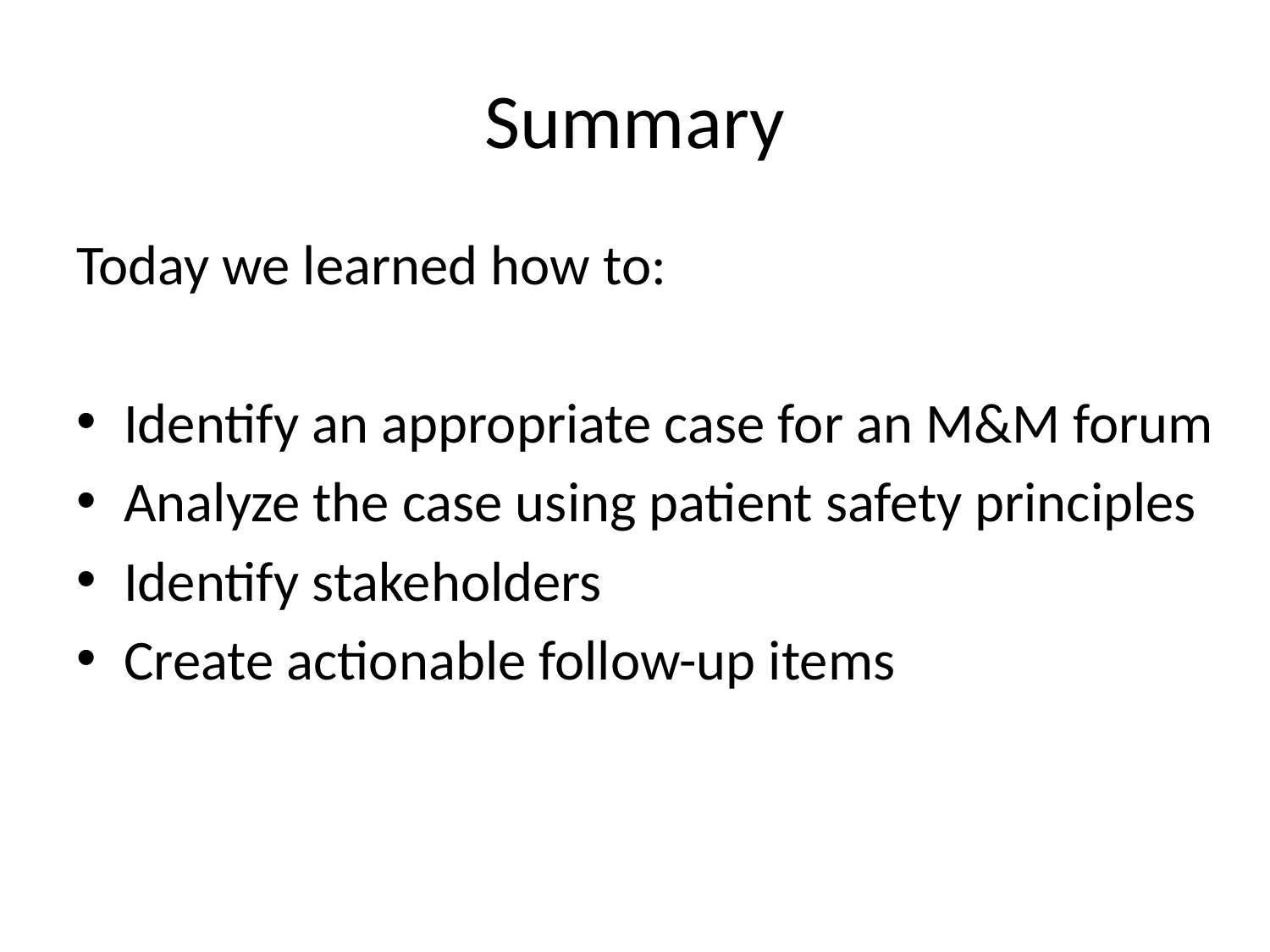

# Summary
Today we learned how to:
Identify an appropriate case for an M&M forum
Analyze the case using patient safety principles
Identify stakeholders
Create actionable follow-up items

## Slide 82
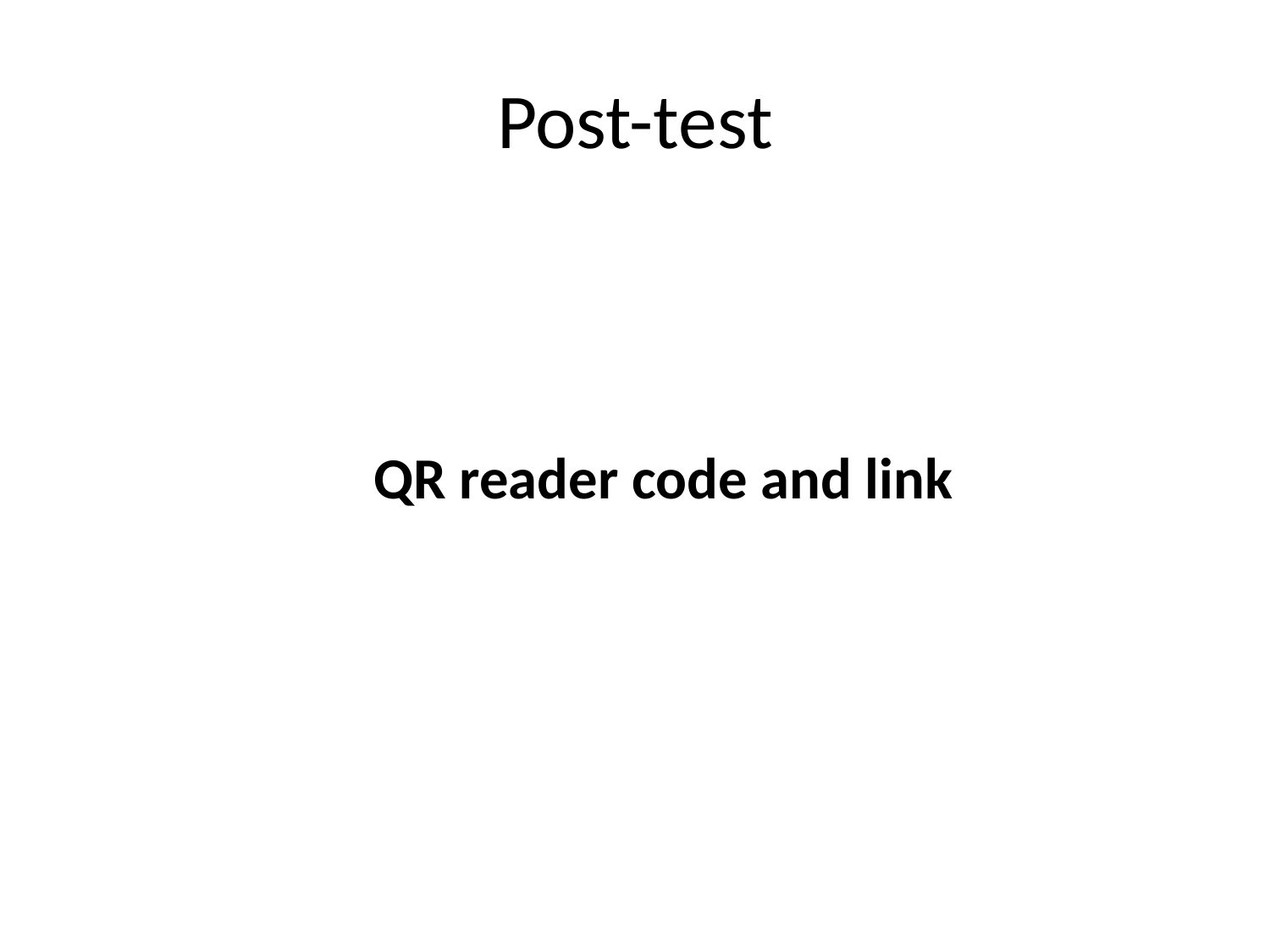

Post-test
QR reader code and link
