# Supplementary material for: Expanding Training in Quality Improvement and Patient Safety Through a Multispecialty Graduate Medical Education Curriculum Designed for Fellows
Source: MedEdPORTAL. 2020 Dec 30;16:11064. doi: 10.15766/mep_2374-8265.11064 (PMC7780740; doi:10.15766/mep_2374-8265.11064)
Supplement: Supplementary file 1 — Foundations in Patient Safety Teaching Slides.pptxFoundations in Patient Safety Playbook and Small-Group Activities.docxAdverse Events Into QI Teaching Slides.pptxAdverse Events Into QI Playbook and Small-Group Activities.docxQuality in Academics Teaching Slides.pptxQuality in Academics Playbook and Small-Group Activities.docxFoundations in Patient Safety Assessment Survey.docxAdverse Events Into QI Assessment Survey.docxQuality in Academics Assessment Survey.docx [file mep_2374-8265.11064-s001.zip › B. Foundations in Patient Safety Playbook and Small-Group Activities.docx]

# Foundations in Patient Safety Playbook

**Agenda**

**8:00 – 8:15** Pre-test & Attendance Sign-In (Scan QR Code)

**8:15 – 8:30** Introduction

**8:30 – 9:30** Definitions and tools

**9:30 – 9:45** *Break*

**9:45 – 10:45**  Just Culture and Facilitation

**10:45 – 11:00** *Break*

**11:00 – 12:00** Mock M&M

**Step 1: Case Selection**

Please provide a one-liner of your case:

What was the adverse event?

What was the level of harm to the patient?

What potential errors contributed?

**Step 2: Analyze the Case**

Create a Cause & Effect Analysis (Fishbone Diagram)

**
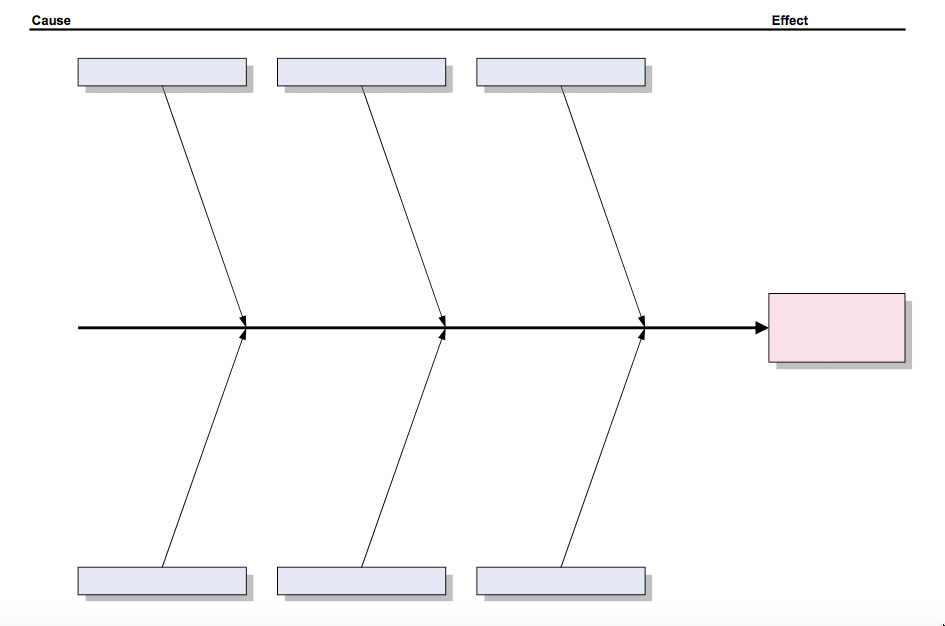
**

**Step 3: Prepare the Conference**

Identify the Stakeholders:


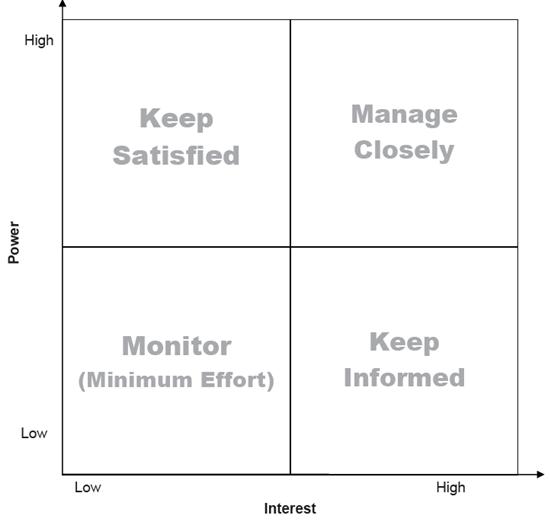


**Step 3: Prepare the Conference**

Identify Learning Objectives

- Medical Objectives  *i.e.* *Guidelines or Best Practices*
- Quality Improvement Objectives *i.e. Quality and Safety Practices*

**Step 4: Identify Action Items**


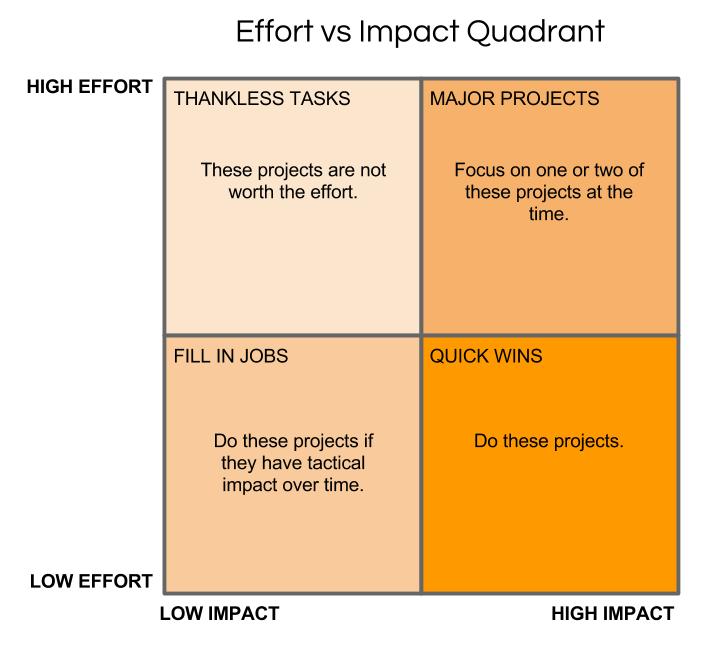


Which patient safety concerns are actionable?

Who is your support team?

How can you align these action items with organization priorities at your hospital?
