# Supplementary material for: Expanding Training in Quality Improvement and Patient Safety Through a Multispecialty Graduate Medical Education Curriculum Designed for Fellows
Source: MedEdPORTAL. 2020 Dec 30;16:11064. doi: 10.15766/mep_2374-8265.11064 (PMC7780740; doi:10.15766/mep_2374-8265.11064)
Supplement: Supplementary file 1 — Foundations in Patient Safety Teaching Slides.pptxFoundations in Patient Safety Playbook and Small-Group Activities.docxAdverse Events Into QI Teaching Slides.pptxAdverse Events Into QI Playbook and Small-Group Activities.docxQuality in Academics Teaching Slides.pptxQuality in Academics Playbook and Small-Group Activities.docxFoundations in Patient Safety Assessment Survey.docxAdverse Events Into QI Assessment Survey.docxQuality in Academics Assessment Survey.docx [file mep_2374-8265.11064-s001.zip › C. Adverse Events Into QI Teaching Slides.pptx]

## Slide 1
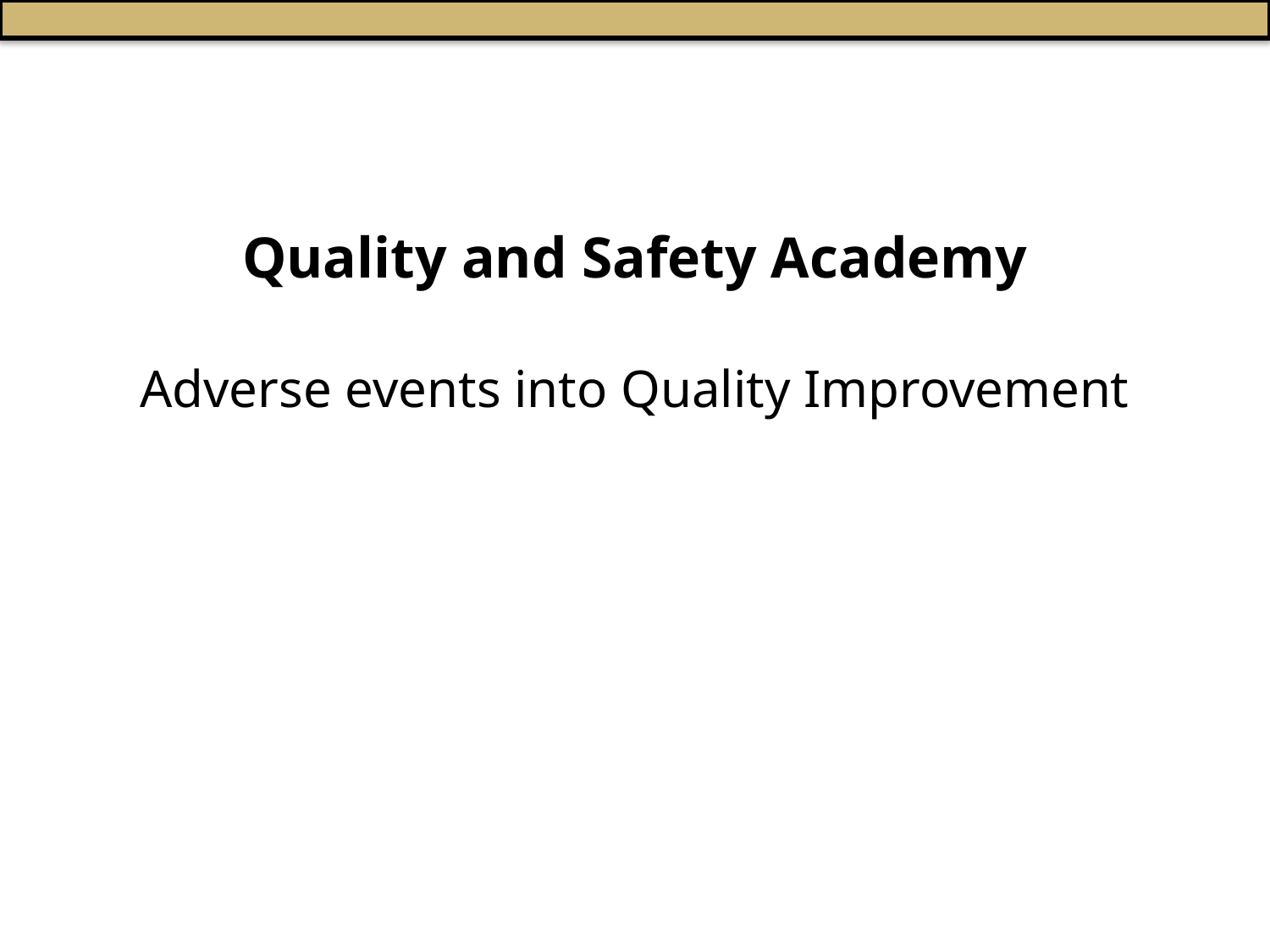

# Quality and Safety Academy Adverse events into Quality Improvement

## Slide 2
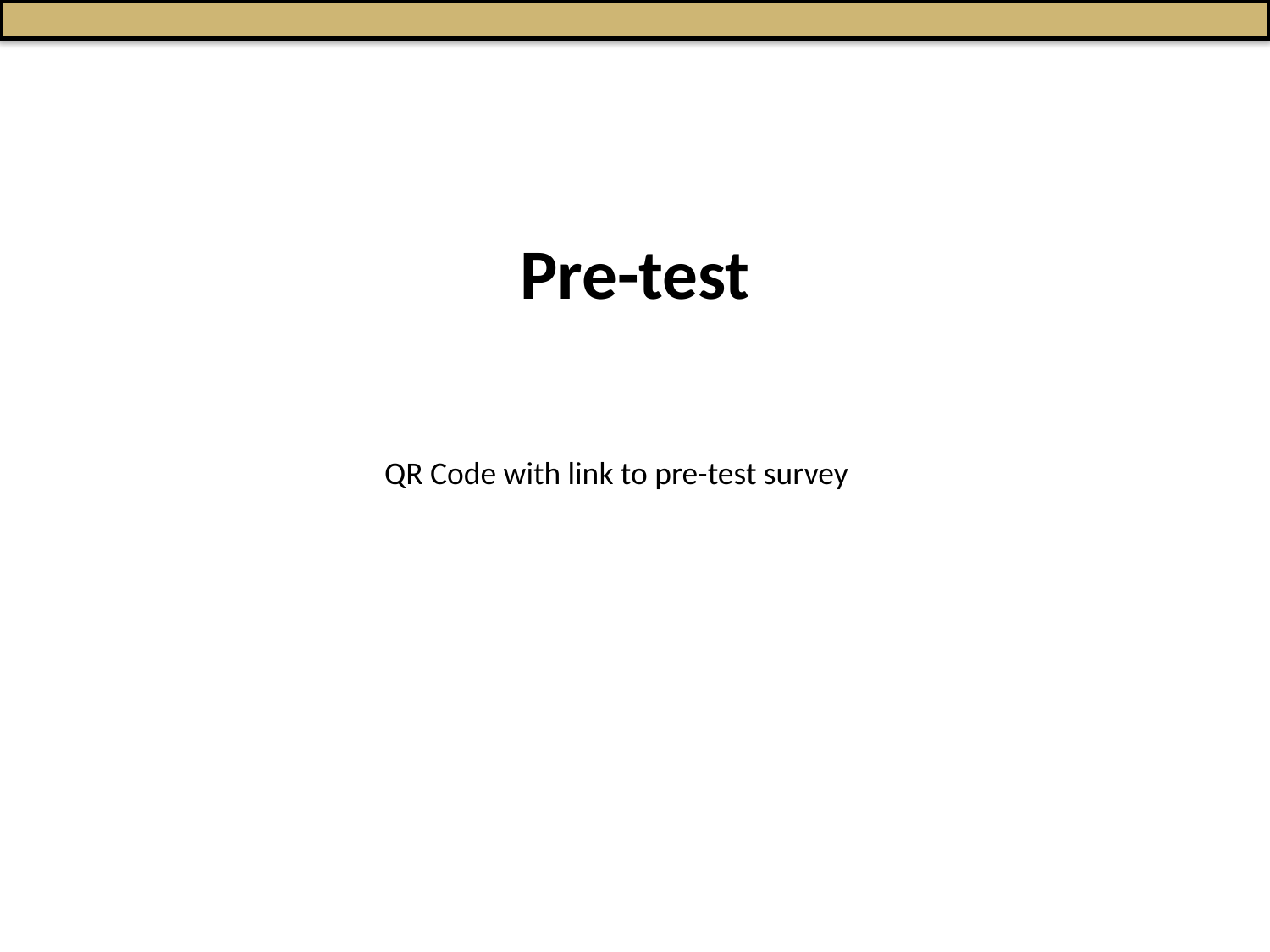

Pre-test
QR Code with link to pre-test survey

## Slide 3
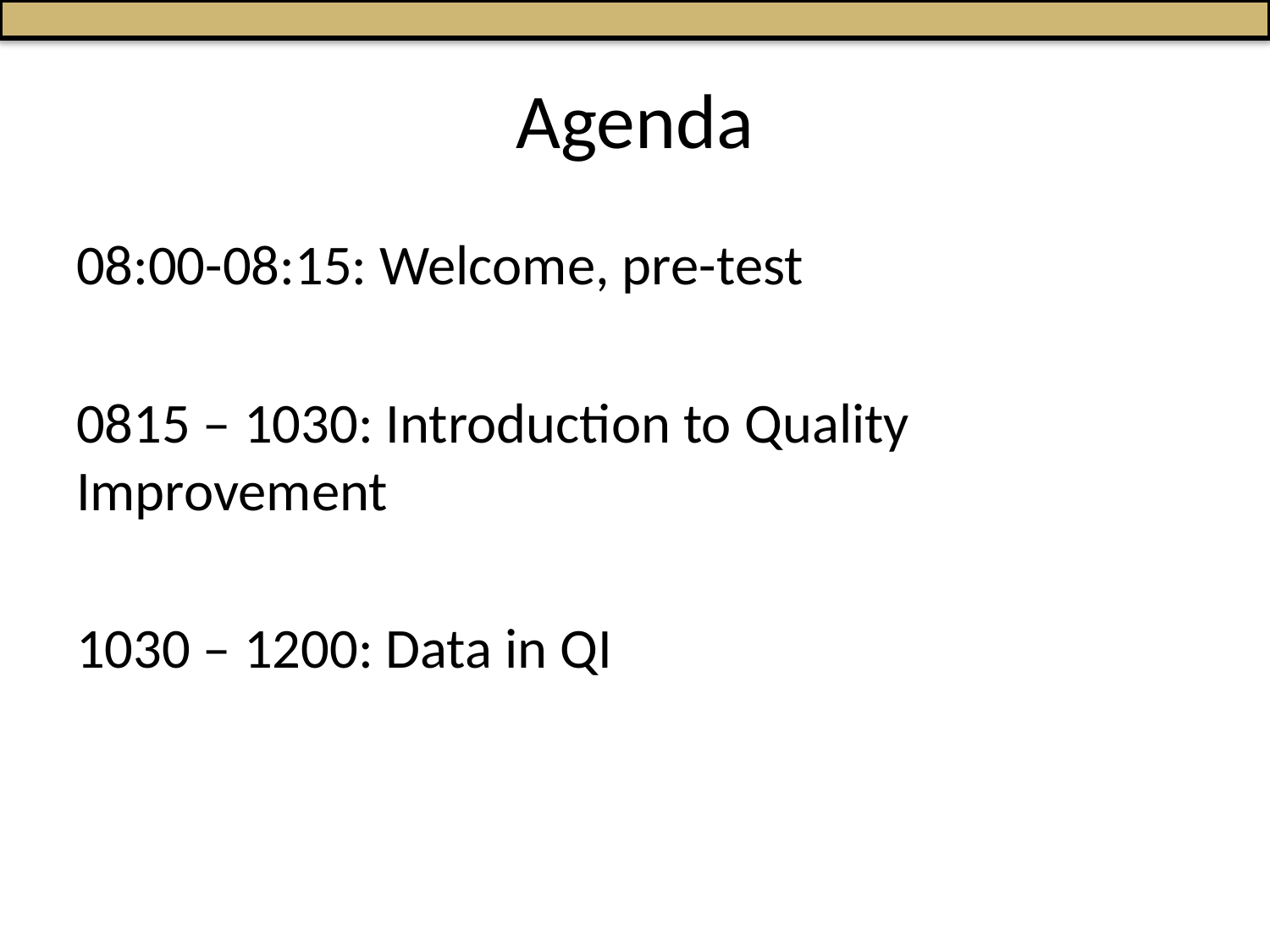

# Agenda
08:00-08:15: Welcome, pre-test
0815 – 1030: Introduction to Quality Improvement
1030 – 1200: Data in QI

## Slide 4
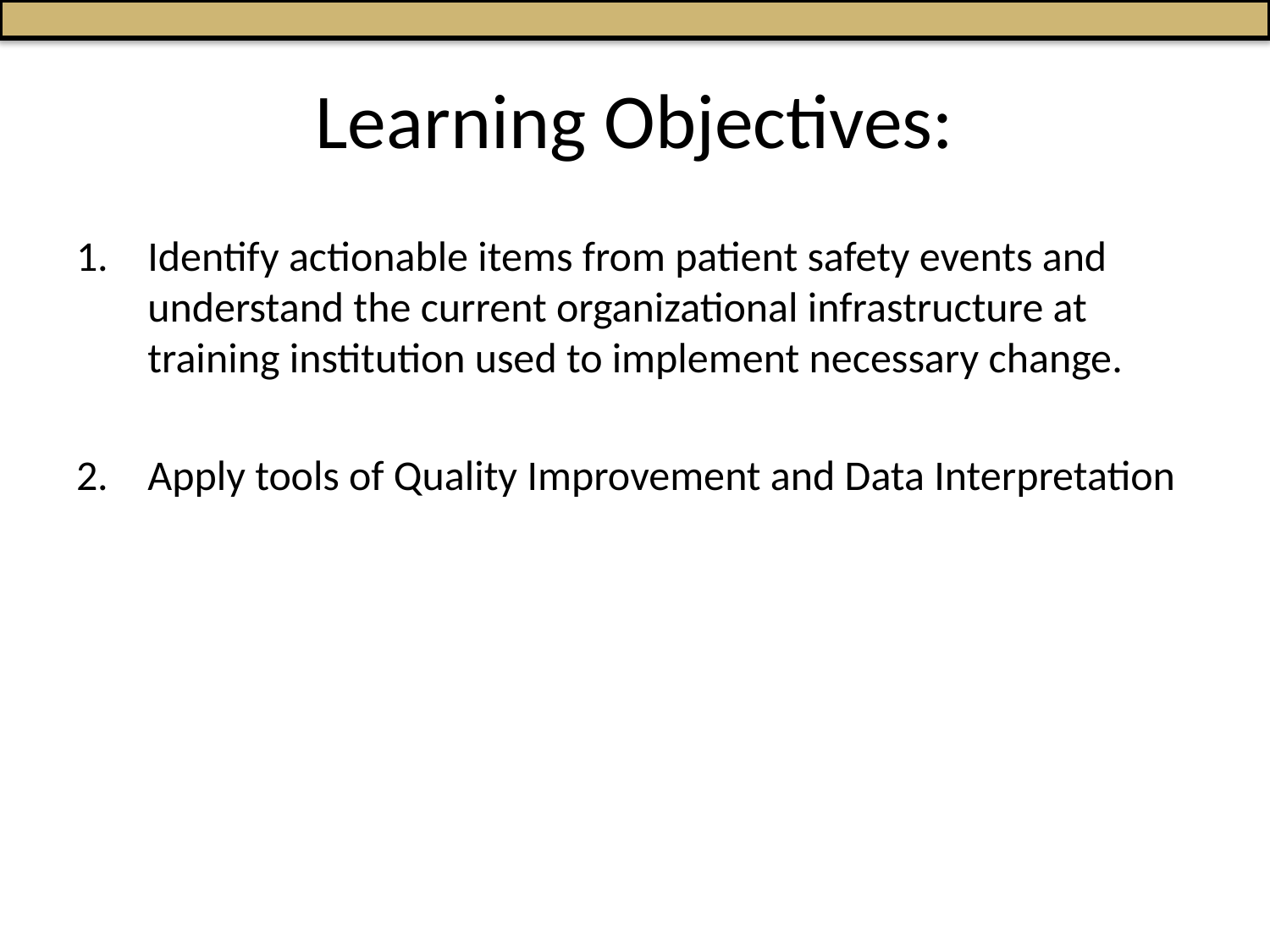

# Learning Objectives:
Identify actionable items from patient safety events and understand the current organizational infrastructure at training institution used to implement necessary change.
Apply tools of Quality Improvement and Data Interpretation

## Slide 5
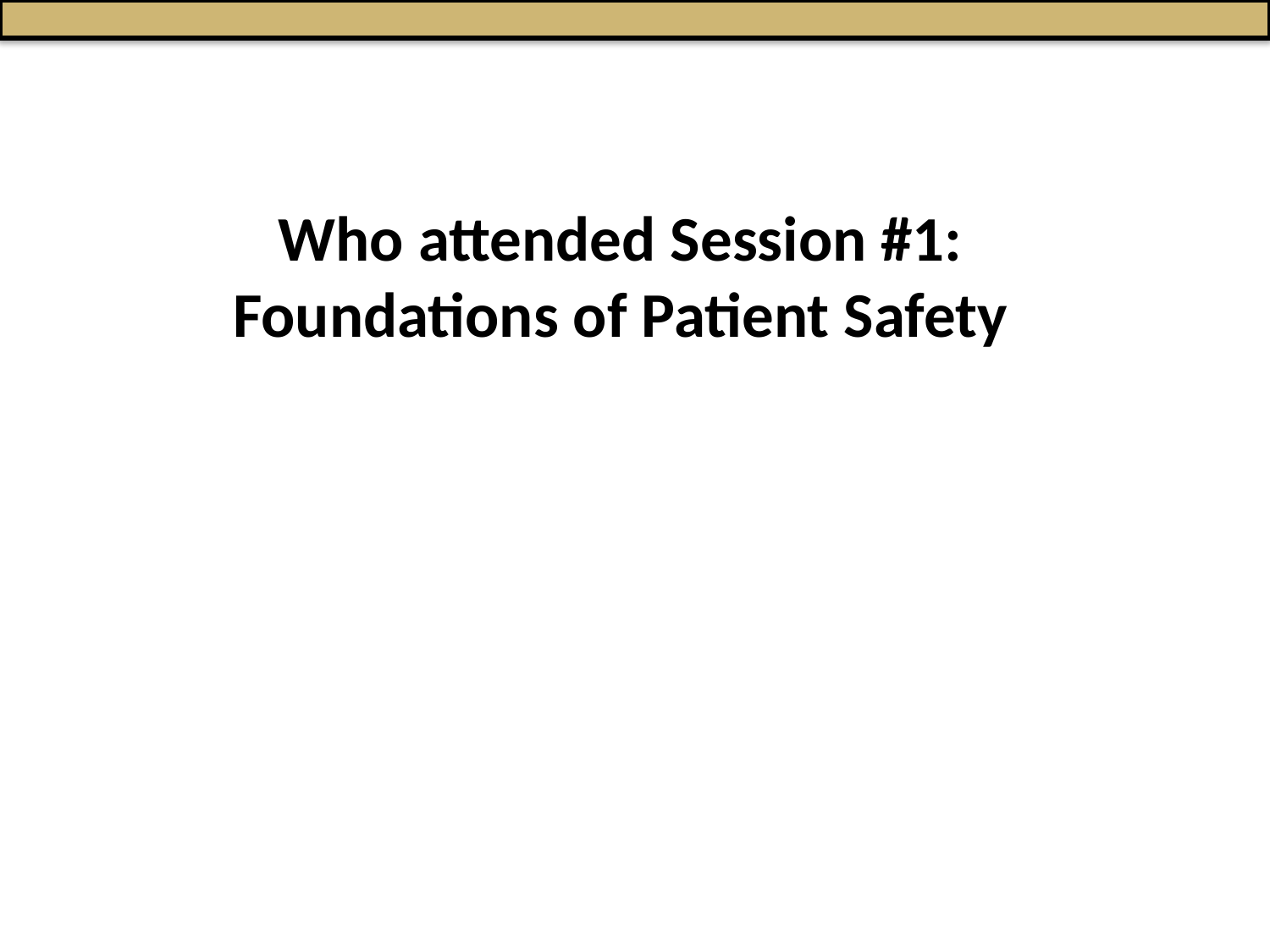

Who attended Session #1: Foundations of Patient Safety

## Slide 6
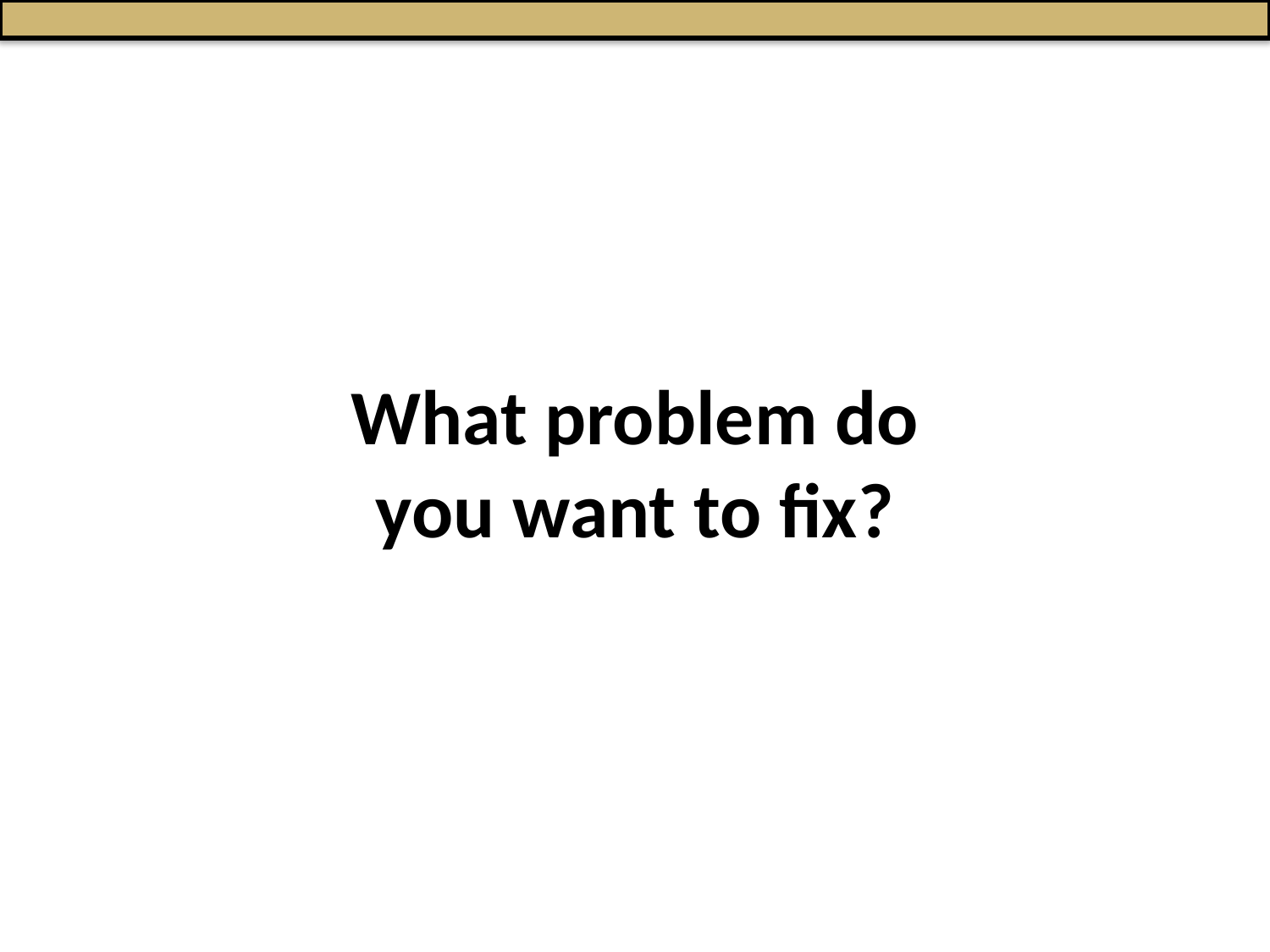

What problem do you want to fix?

## Slide 7
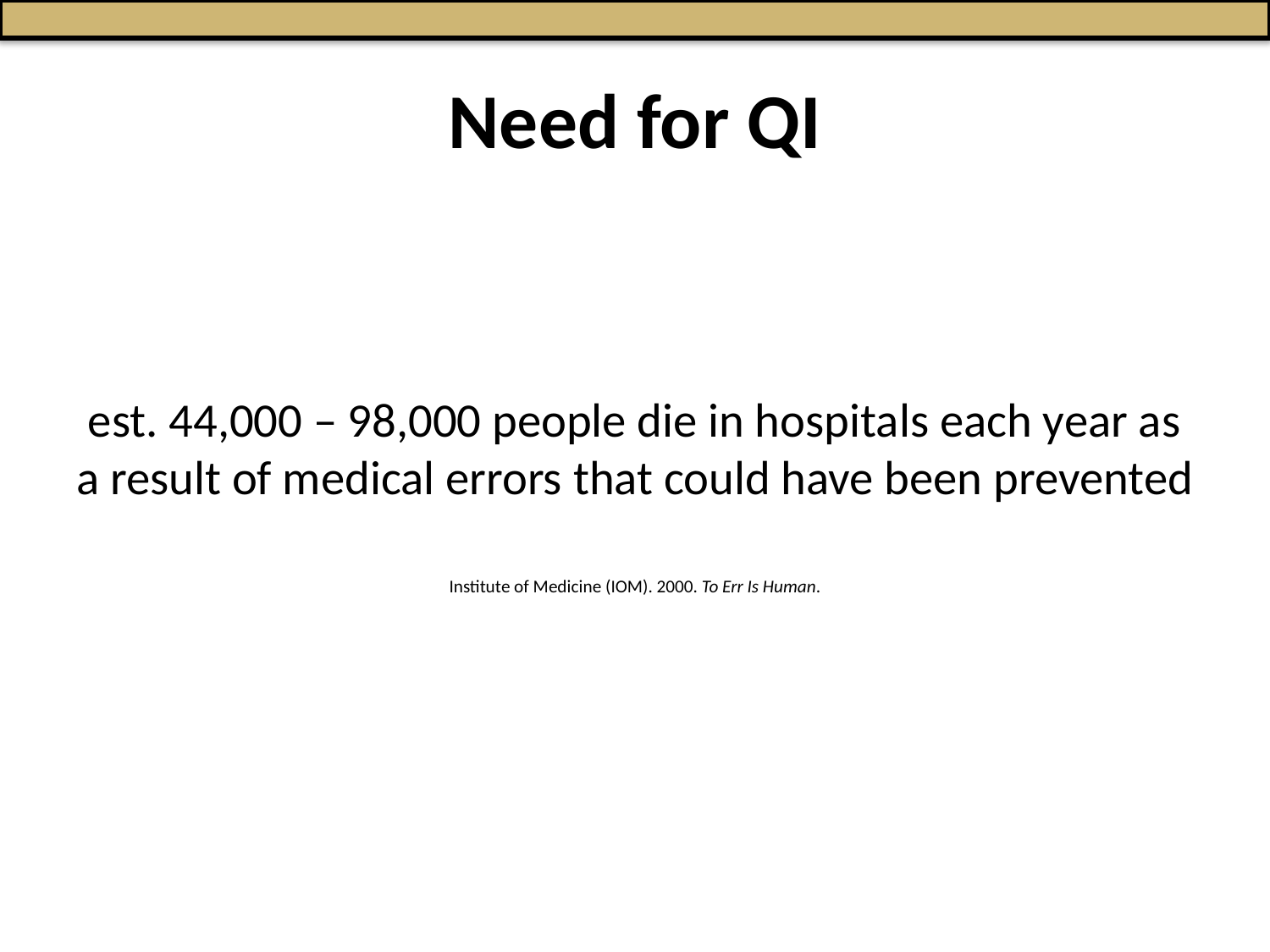

# Need for QI
est. 44,000 – 98,000 people die in hospitals each year as a result of medical errors that could have been prevented
Institute of Medicine (IOM). 2000. To Err Is Human.

## Slide 8
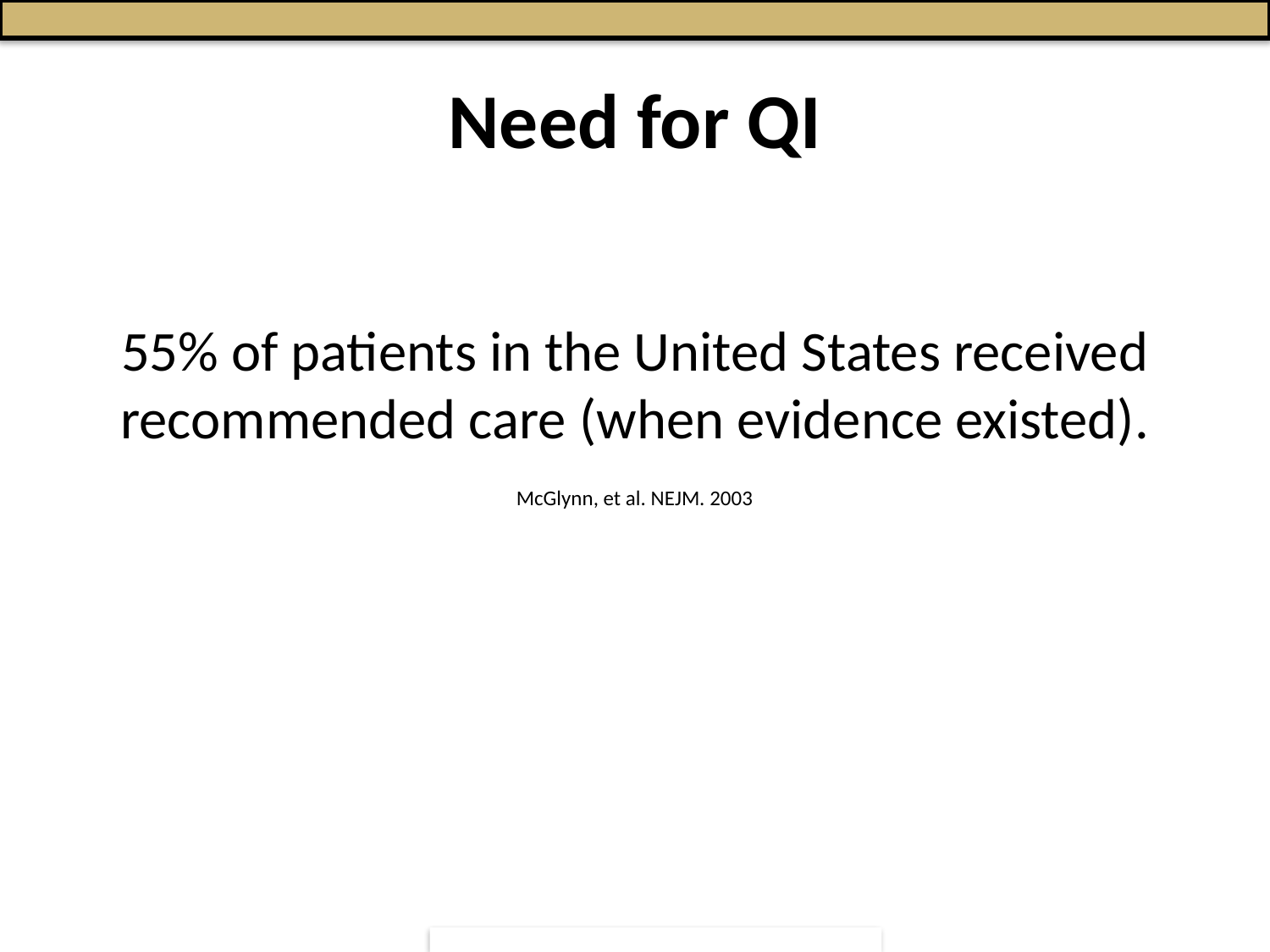

# Need for QI
55% of patients in the United States received recommended care (when evidence existed).
McGlynn, et al. NEJM. 2003

## Slide 9
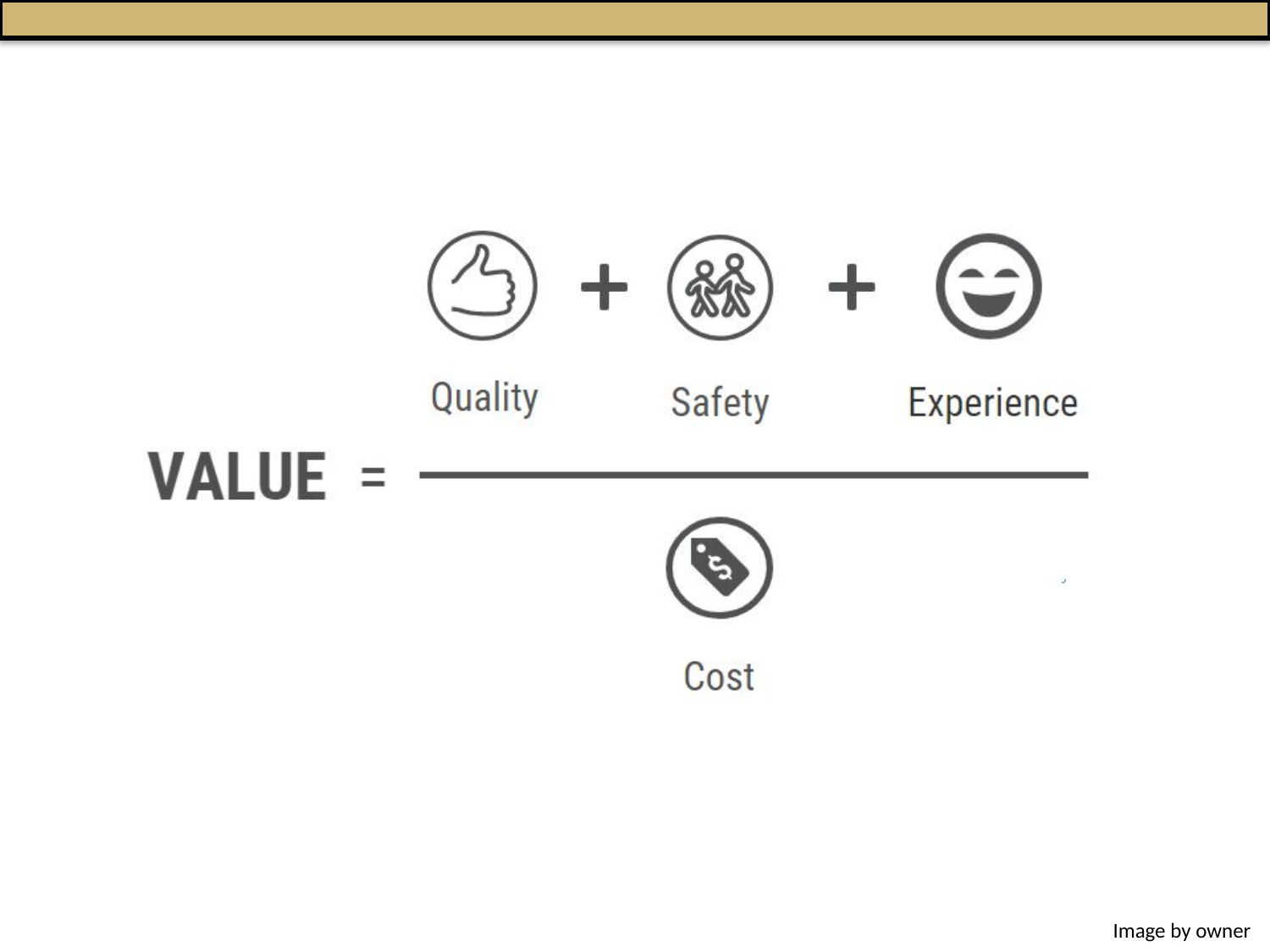

Image by owner

## Slide 10
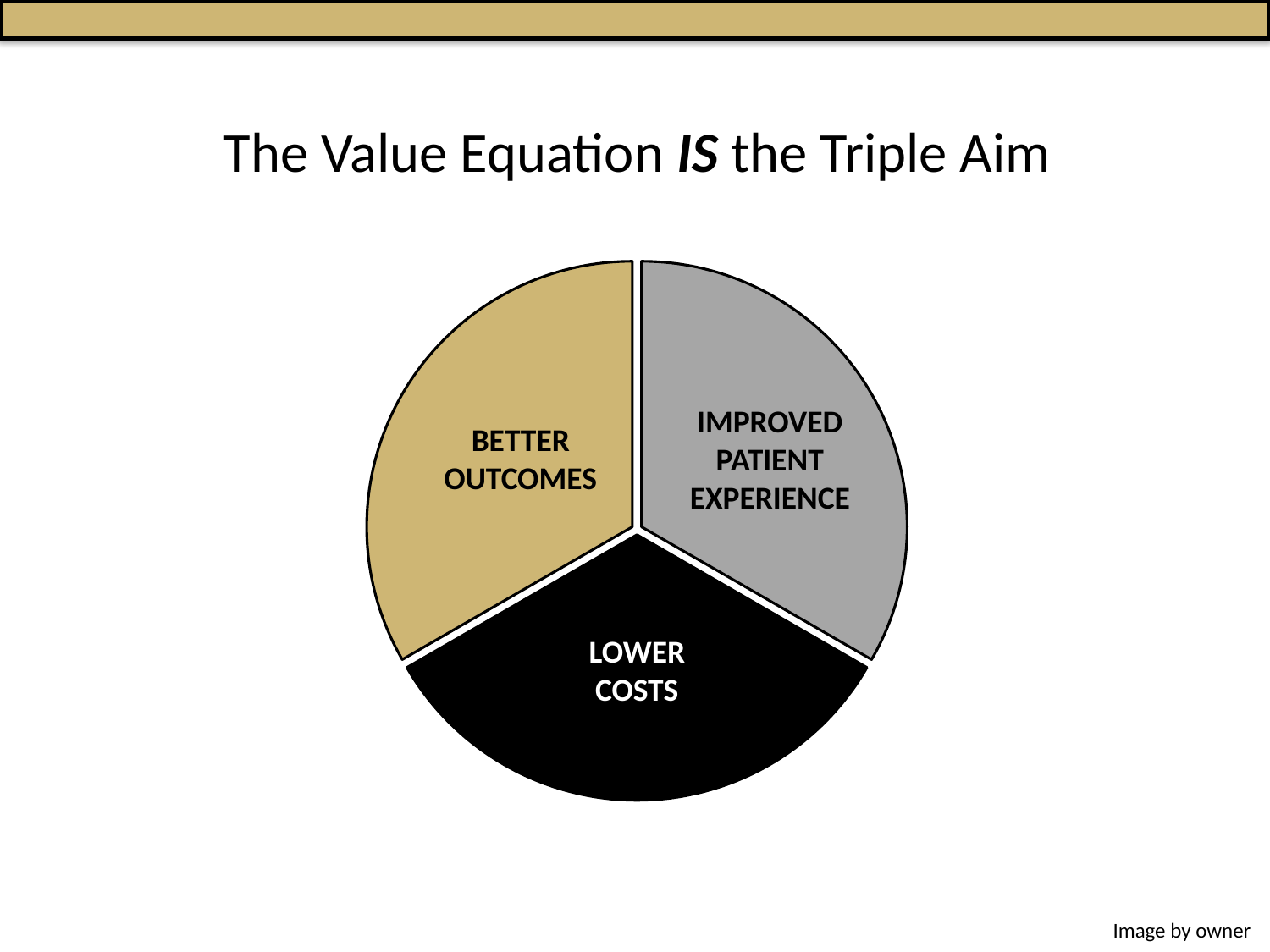

The Value Equation IS the Triple Aim
### Chart
| Category | Column1 |
|---|---|
| | 0.33 |
| | 0.33 |
| | 0.33 |IMPROVED PATIENT EXPERIENCE
BETTER OUTCOMES
LOWER COSTS
Image by owner

## Slide 11
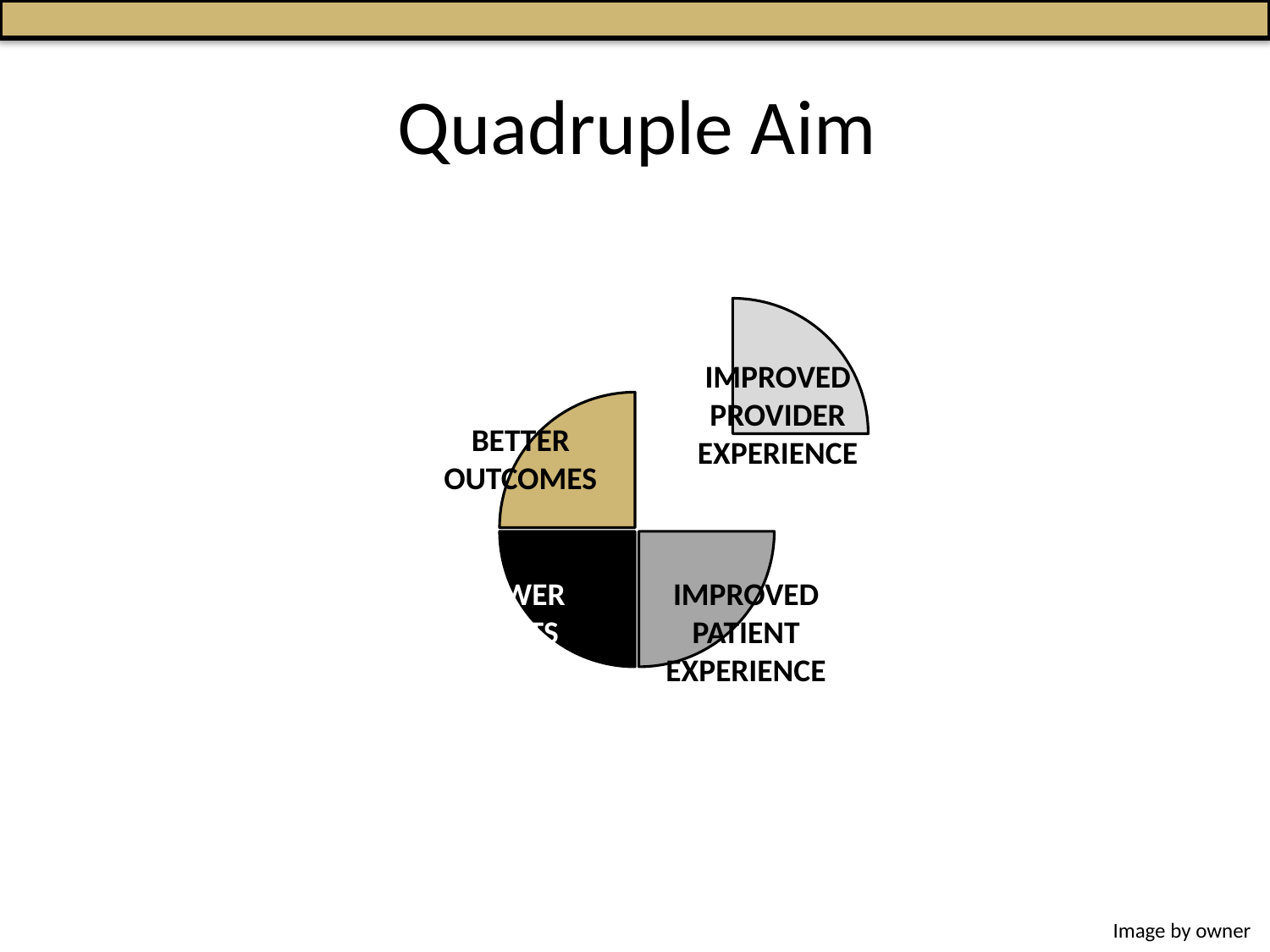

# Quadruple Aim
### Chart
| Category | Column1 |
|---|---|
| | 0.25 |
| | 0.25 |
| | 0.25 |
| | 0.25 |IMPROVED PROVIDER EXPERIENCE
BETTER OUTCOMES
LOWER COSTS
IMPROVED PATIENT EXPERIENCE
Image by owner

## Slide 12
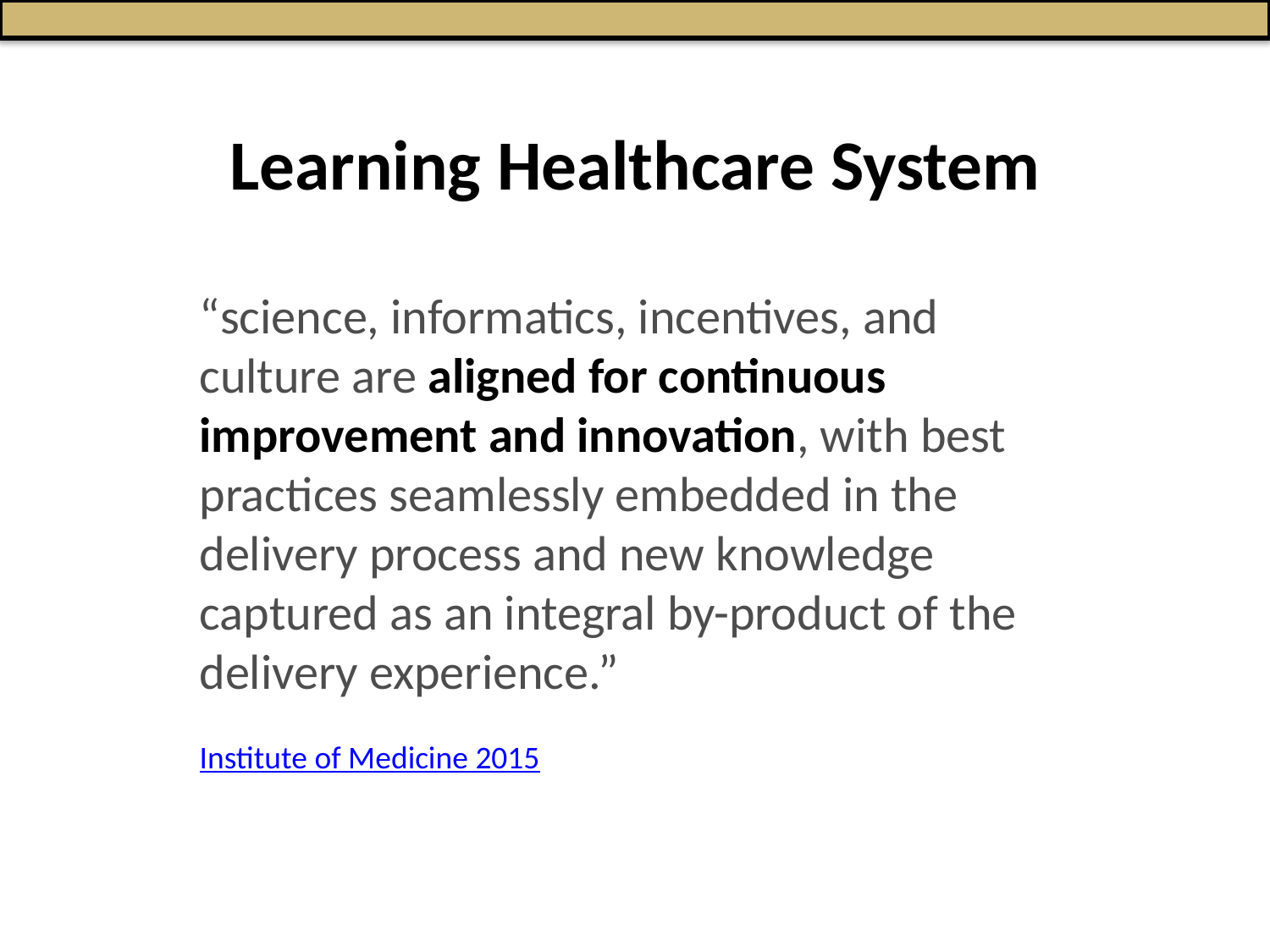

Learning Healthcare System
“science, informatics, incentives, and culture are aligned for continuous improvement and innovation, with best practices seamlessly embedded in the delivery process and new knowledge captured as an integral by-product of the delivery experience.”
Institute of Medicine 2015

## Slide 13
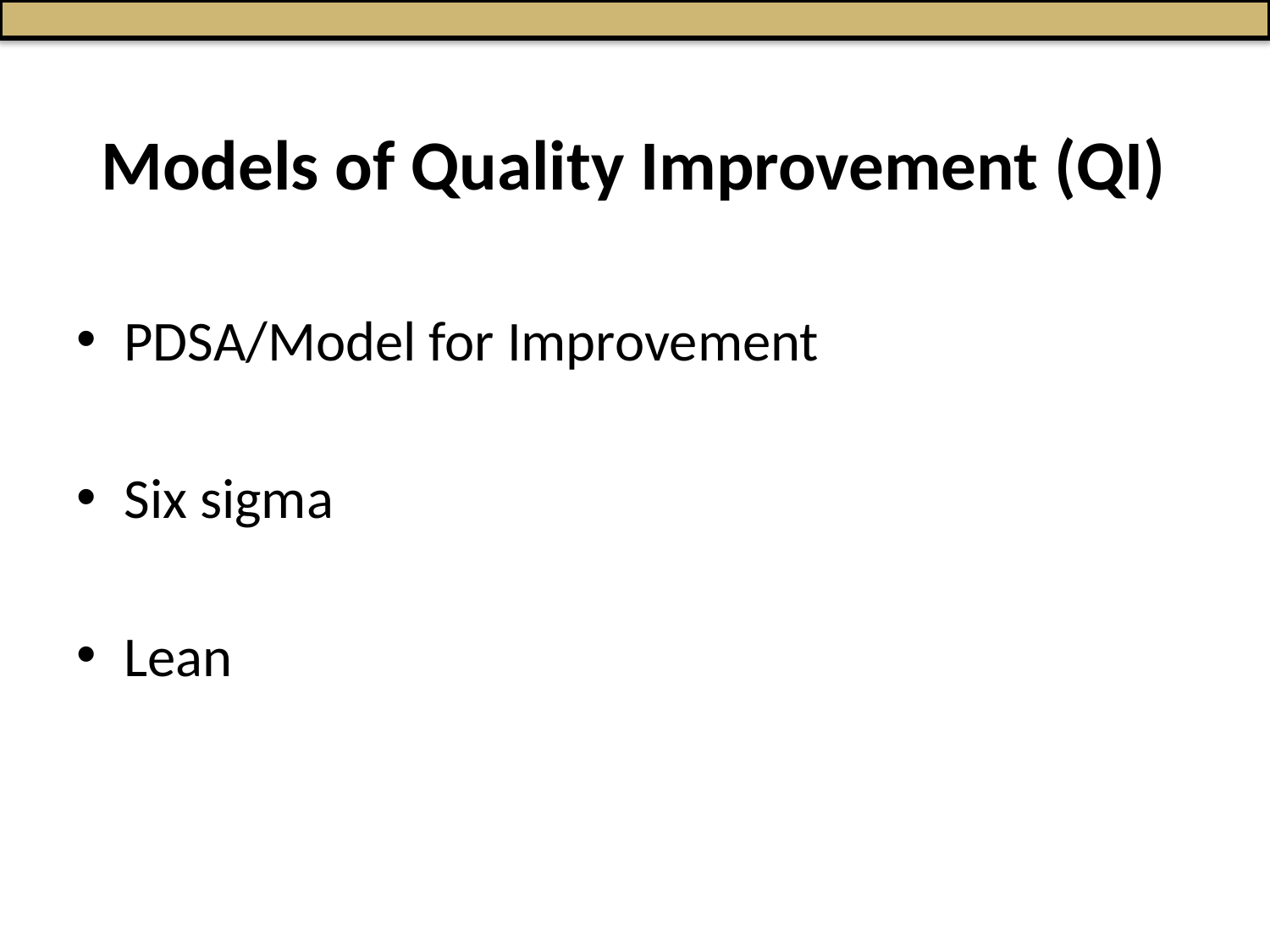

Models of Quality Improvement (QI)
PDSA/Model for Improvement
Six sigma
Lean

## Slide 14
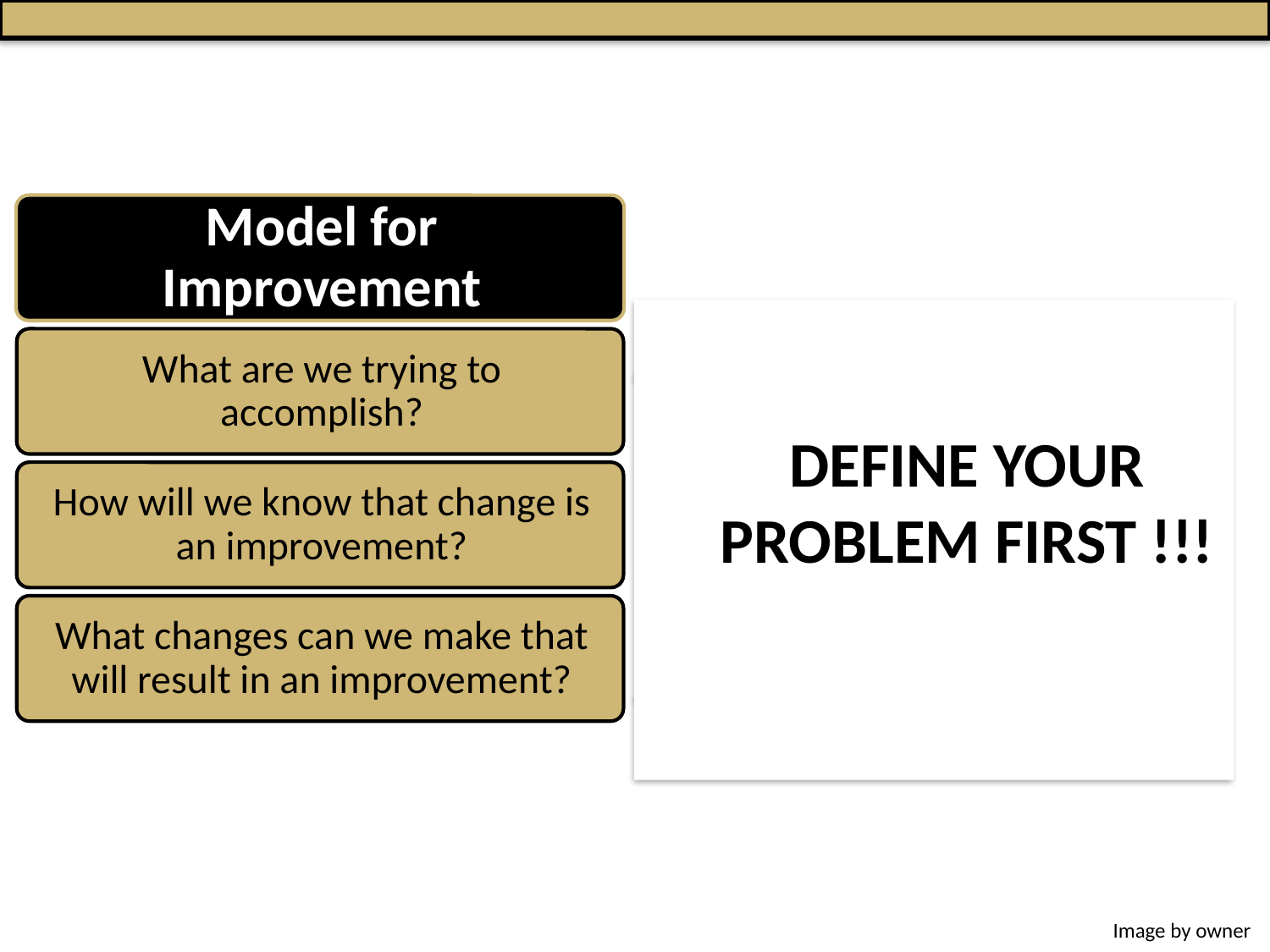

### Chart
| Category | Column1 |
|---|---|
| | 0.25 |
| | 0.25 |
| | 0.25 |
| | 0.25 |
DEFINE YOUR PROBLEM FIRST !!!
ACT
PLAN
STUDY
DO
Image by owner

## Slide 15
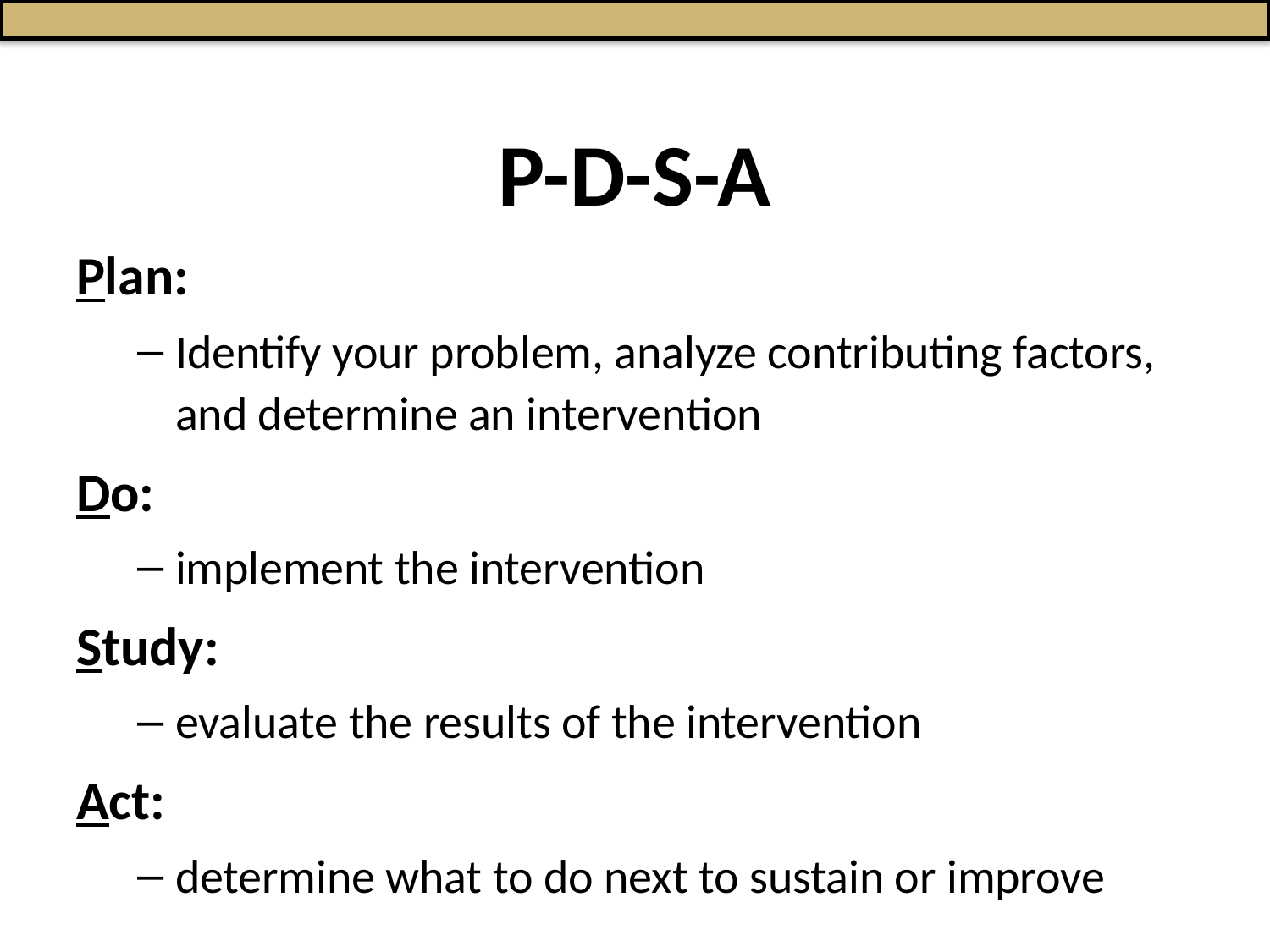

P-D-S-A
Plan:
Identify your problem, analyze contributing factors, and determine an intervention
Do:
implement the intervention
Study:
evaluate the results of the intervention
Act:
determine what to do next to sustain or improve

## Slide 16
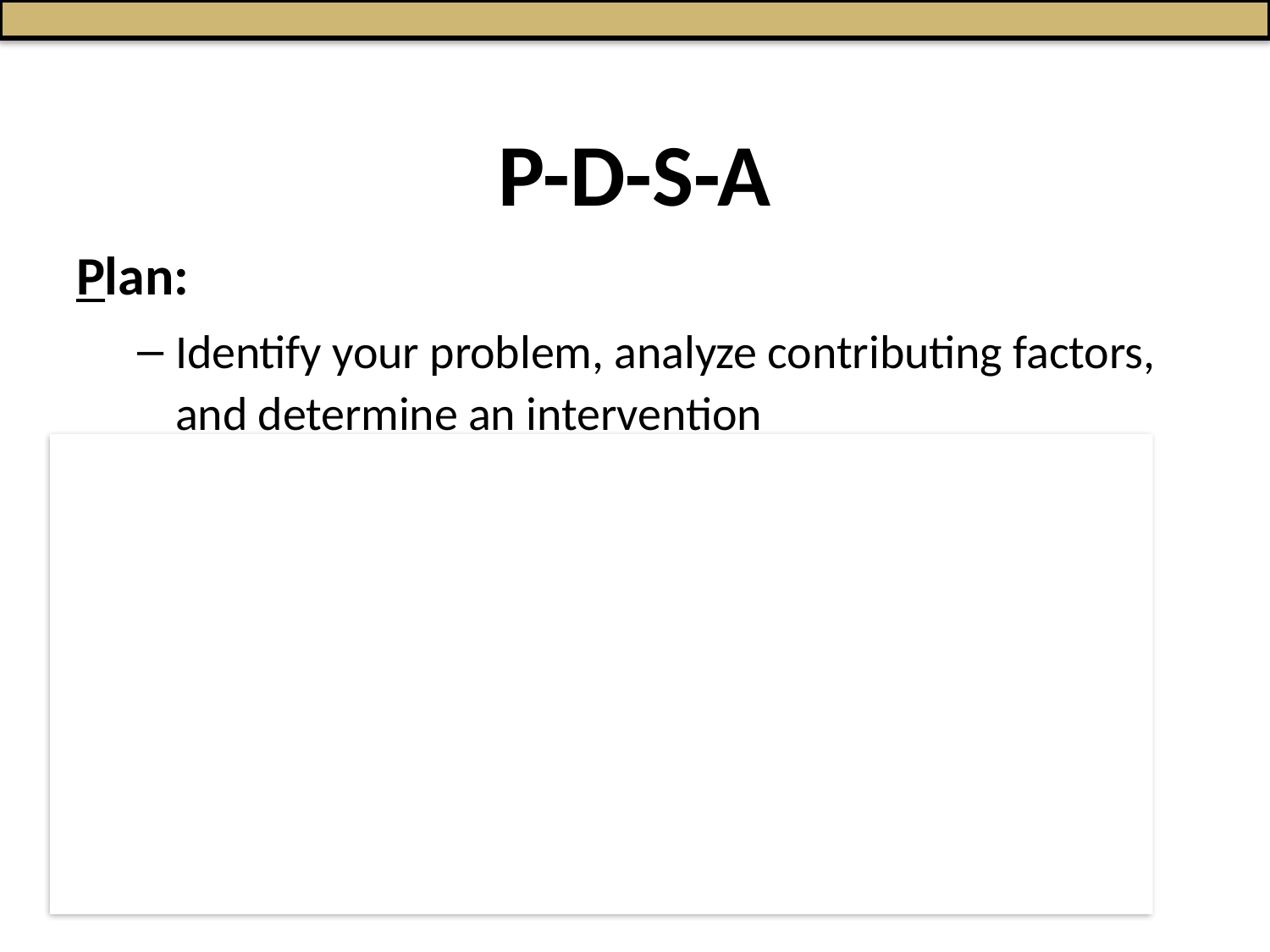

P-D-S-A
Plan:
Identify your problem, analyze contributing factors, and determine an intervention
Do:
implement the intervention
Study:
evaluate the results of the intervention
Act:
determine what to do next to sustain or improve

## Slide 17
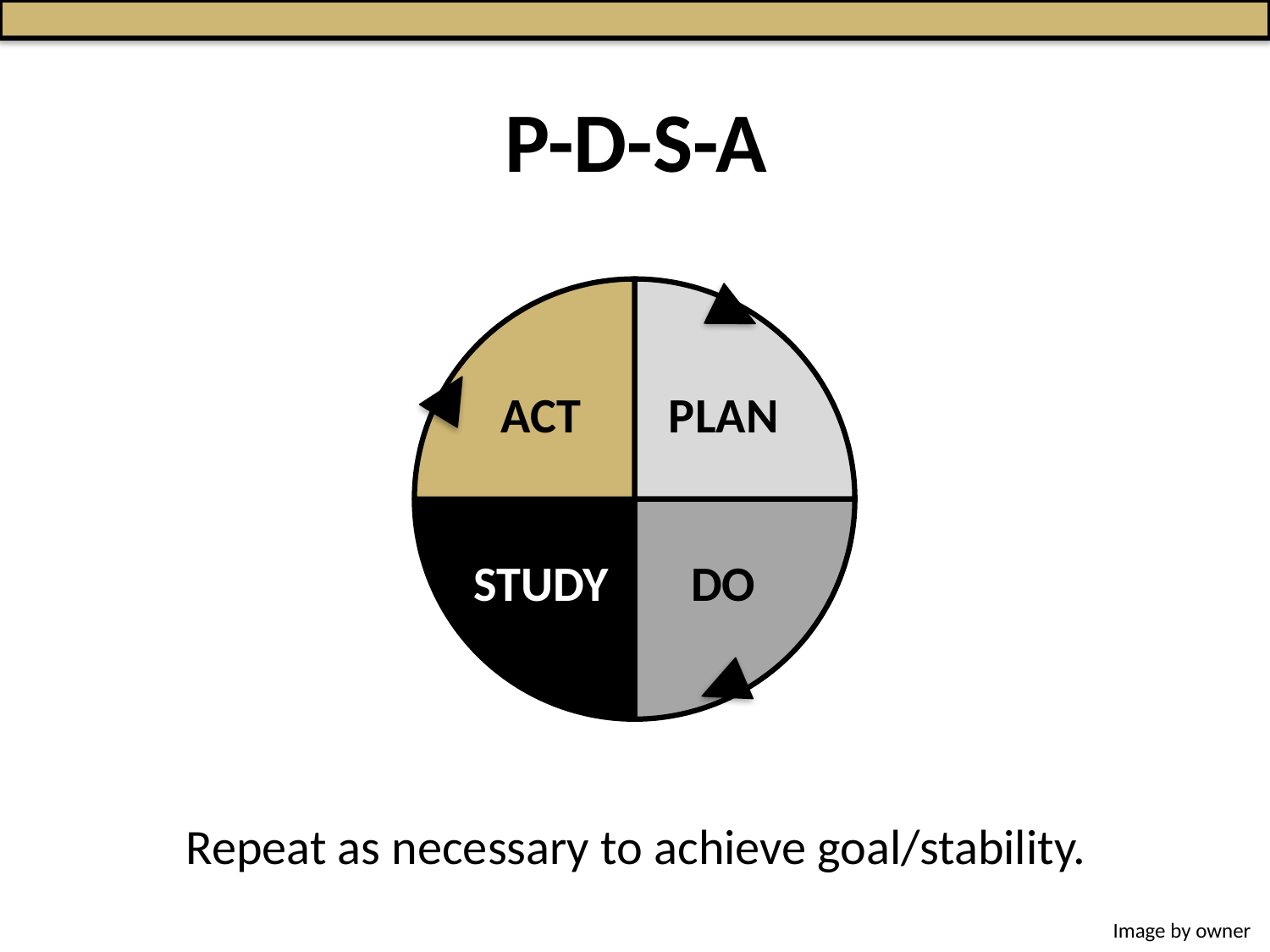

P-D-S-A
### Chart
| Category | Column1 |
|---|---|
| | 0.25 |
| | 0.25 |
| | 0.25 |
| | 0.25 |
ACT
PLAN
STUDY
DO
Repeat as necessary to achieve goal/stability.
Image by owner

## Slide 18
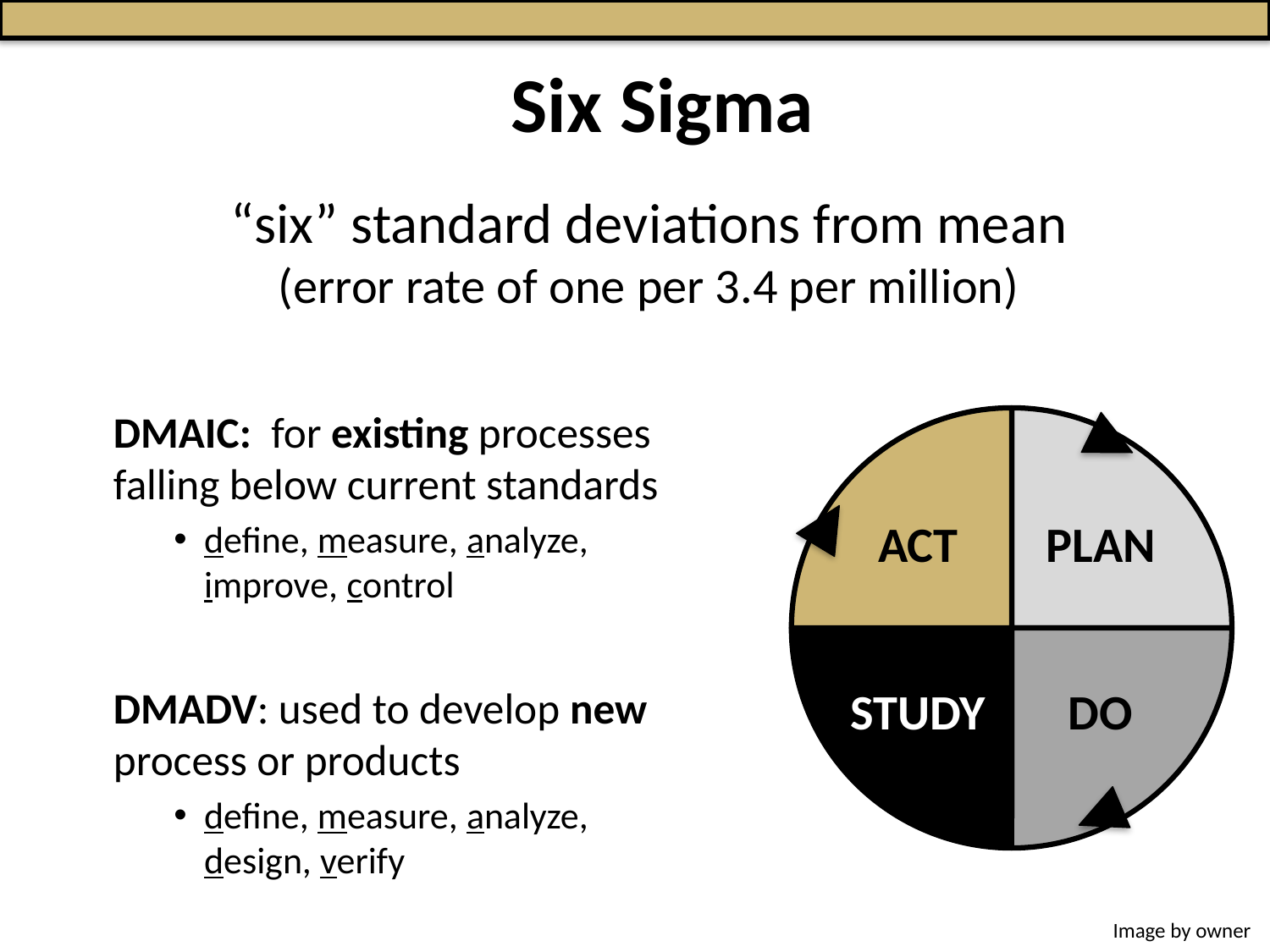

# Six Sigma
“six” standard deviations from mean
(error rate of one per 3.4 per million)
DMAIC: for existing processes falling below current standards
define, measure, analyze, improve, control
DMADV: used to develop new process or products
define, measure, analyze, design, verify
### Chart
| Category | Column1 |
|---|---|
| | 0.25 |
| | 0.25 |
| | 0.25 |
| | 0.25 |
ACT
PLAN
STUDY
DO
Image by owner

## Slide 19
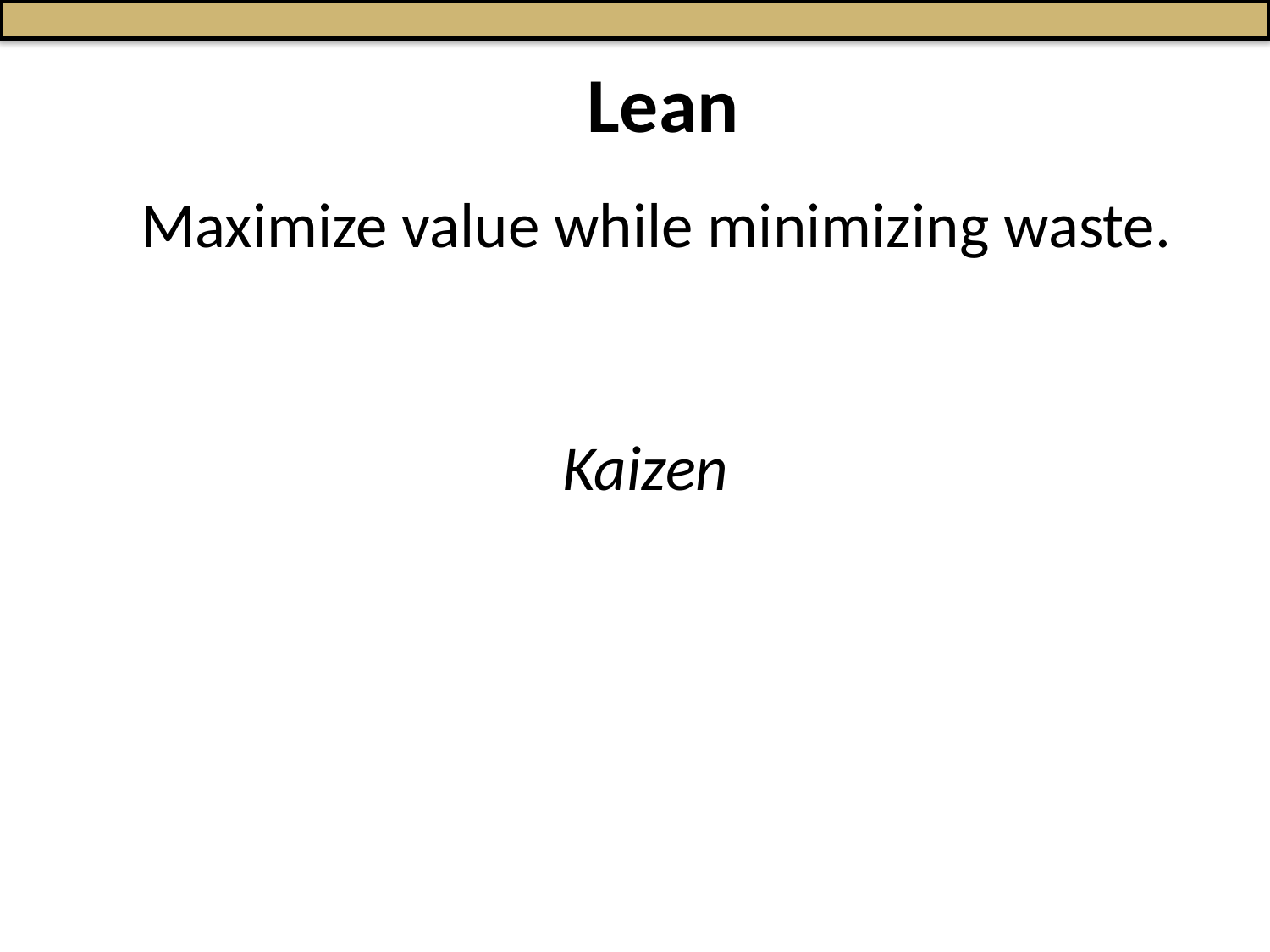

# Lean
Maximize value while minimizing waste.
Kaizen

## Slide 20
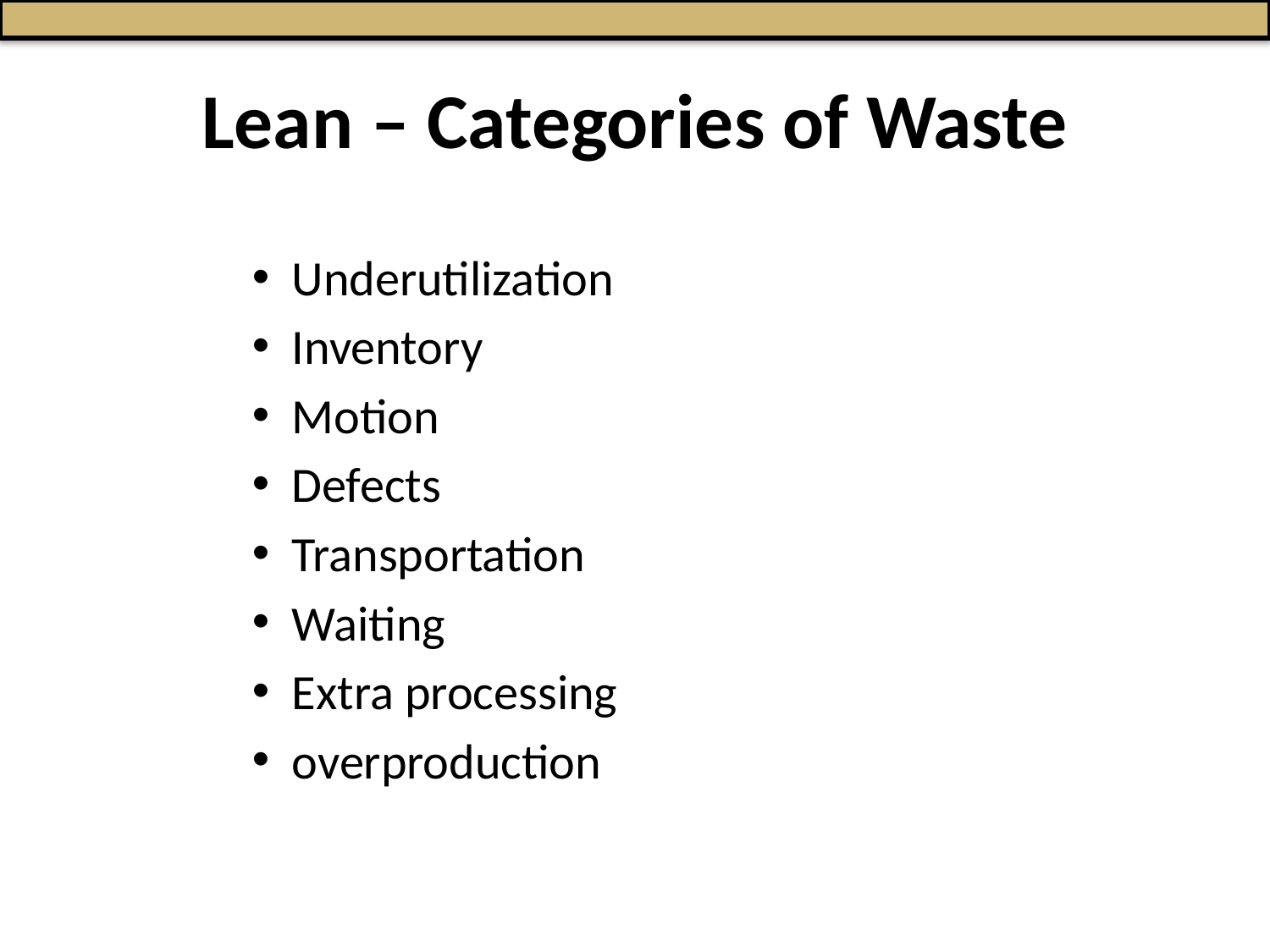

# Lean – Categories of Waste
Underutilization
Inventory
Motion
Defects
Transportation
Waiting
Extra processing
overproduction

## Slide 21
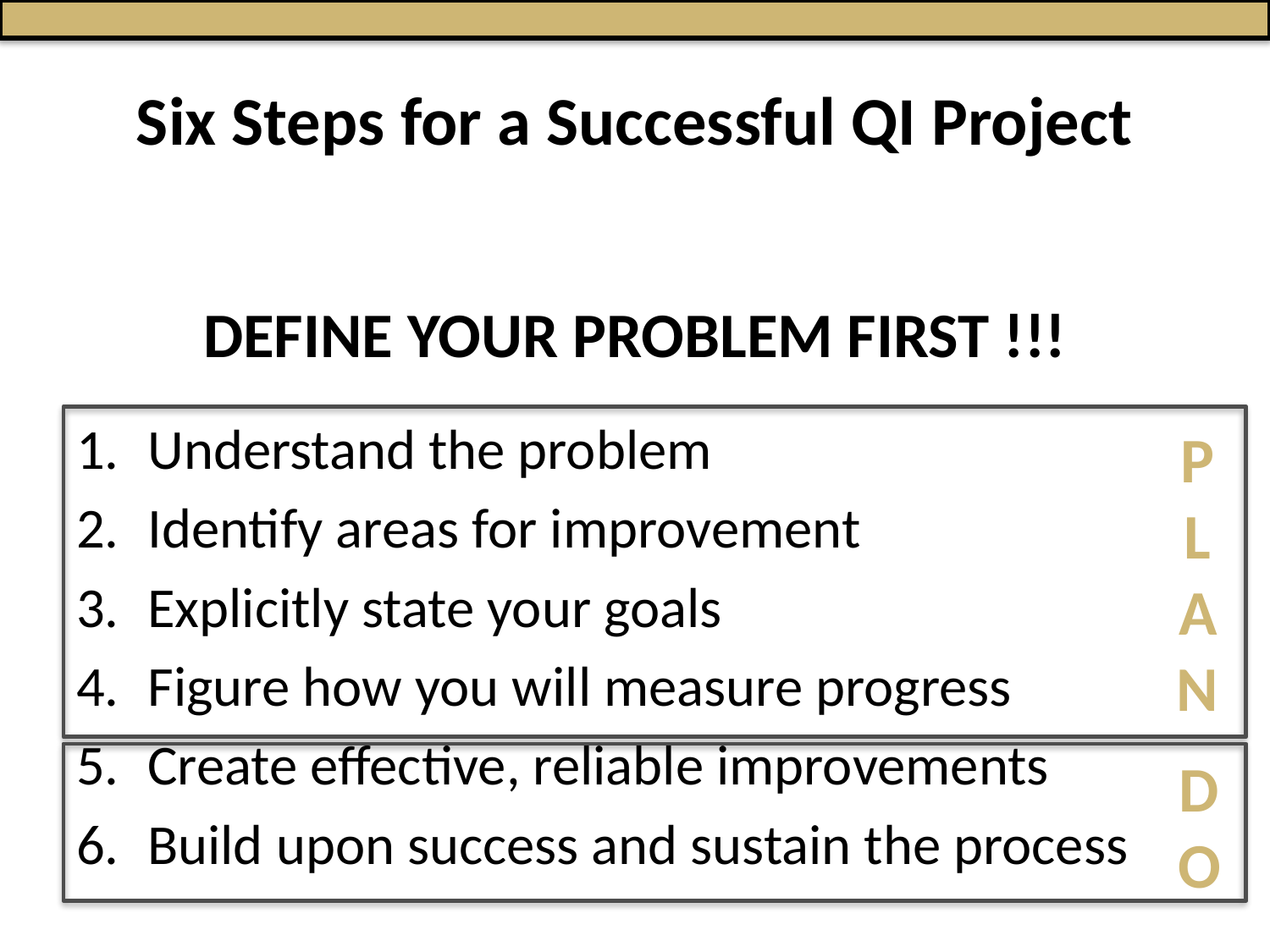

# Six Steps for a Successful QI Project
DEFINE YOUR PROBLEM FIRST !!!
Understand the problem
Identify areas for improvement
Explicitly state your goals
Figure how you will measure progress
Create effective, reliable improvements
Build upon success and sustain the process
PLAN
DO

## Slide 22
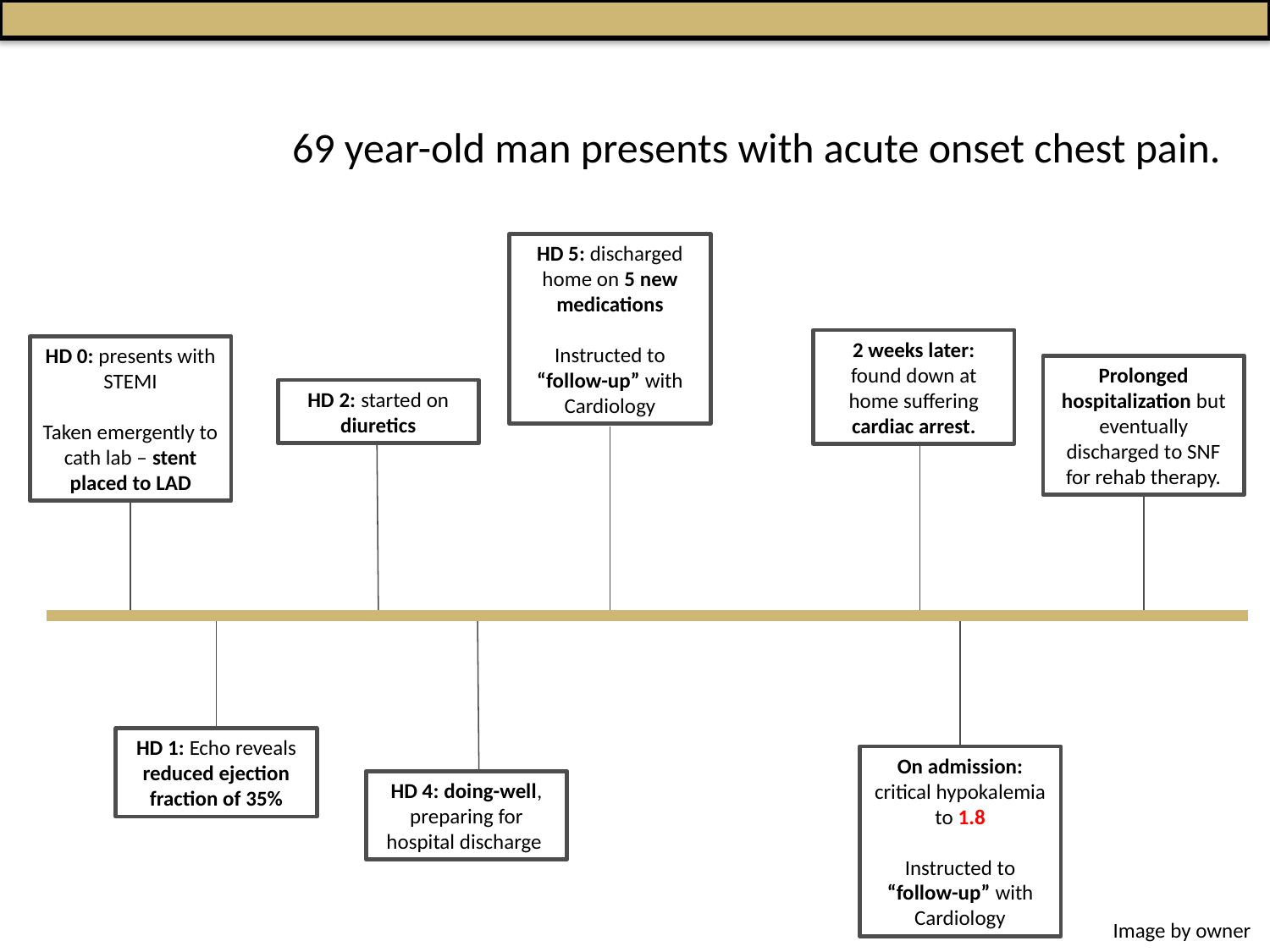

69 year-old man presents with acute onset chest pain.
HD 5: discharged home on 5 new medications
Instructed to “follow-up” with Cardiology
2 weeks later: found down at home suffering cardiac arrest.
HD 0: presents with STEMI
Taken emergently to cath lab – stent placed to LAD
Prolonged hospitalization but eventually discharged to SNF for rehab therapy.
HD 2: started on diuretics
HD 1: Echo reveals reduced ejection fraction of 35%
On admission: critical hypokalemia to 1.8
Instructed to “follow-up” with Cardiology
HD 4: doing-well, preparing for hospital discharge
Image by owner

## Slide 23
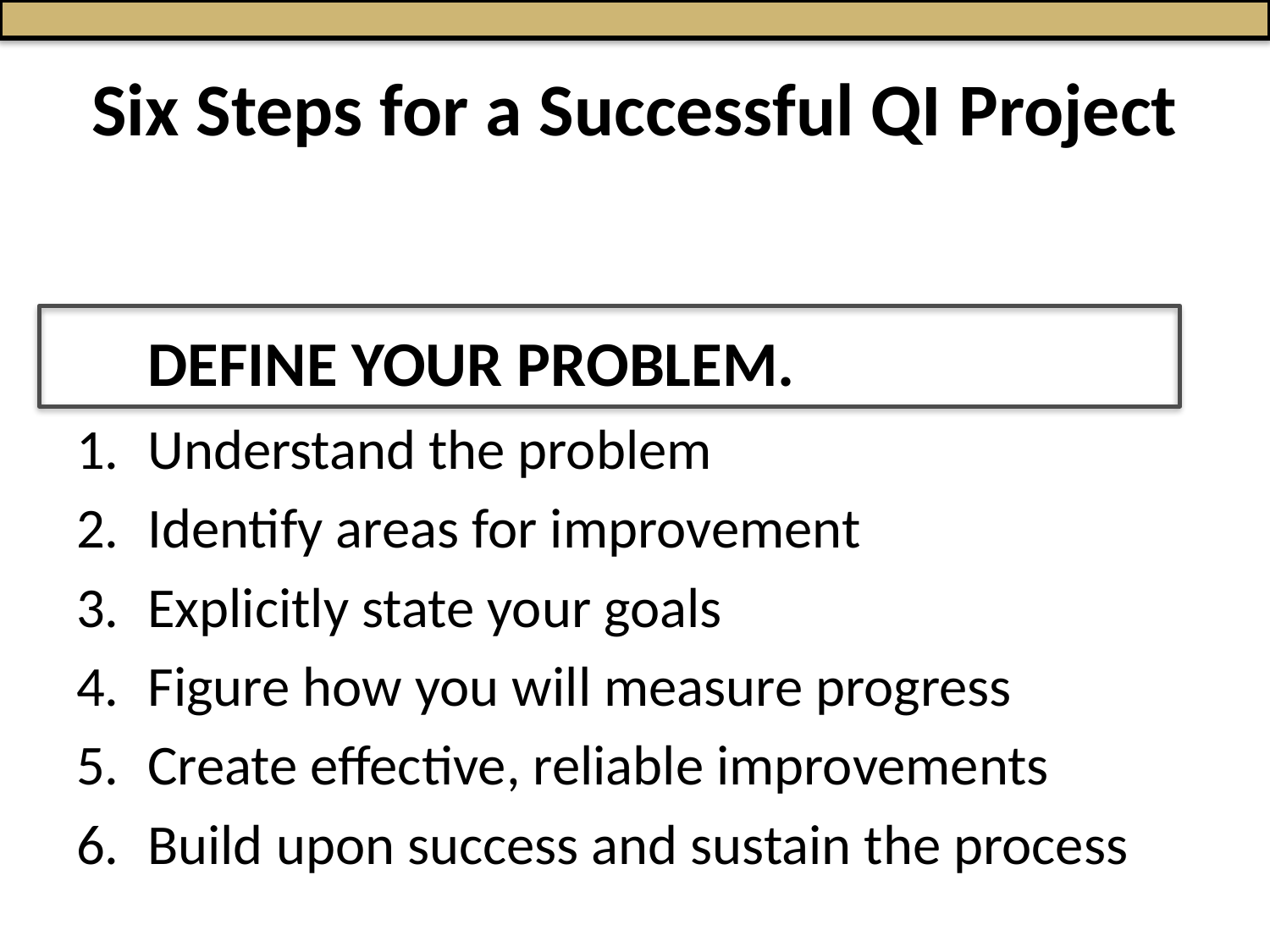

# Six Steps for a Successful QI Project
DEFINE YOUR PROBLEM.
Understand the problem
Identify areas for improvement
Explicitly state your goals
Figure how you will measure progress
Create effective, reliable improvements
Build upon success and sustain the process

## Slide 24
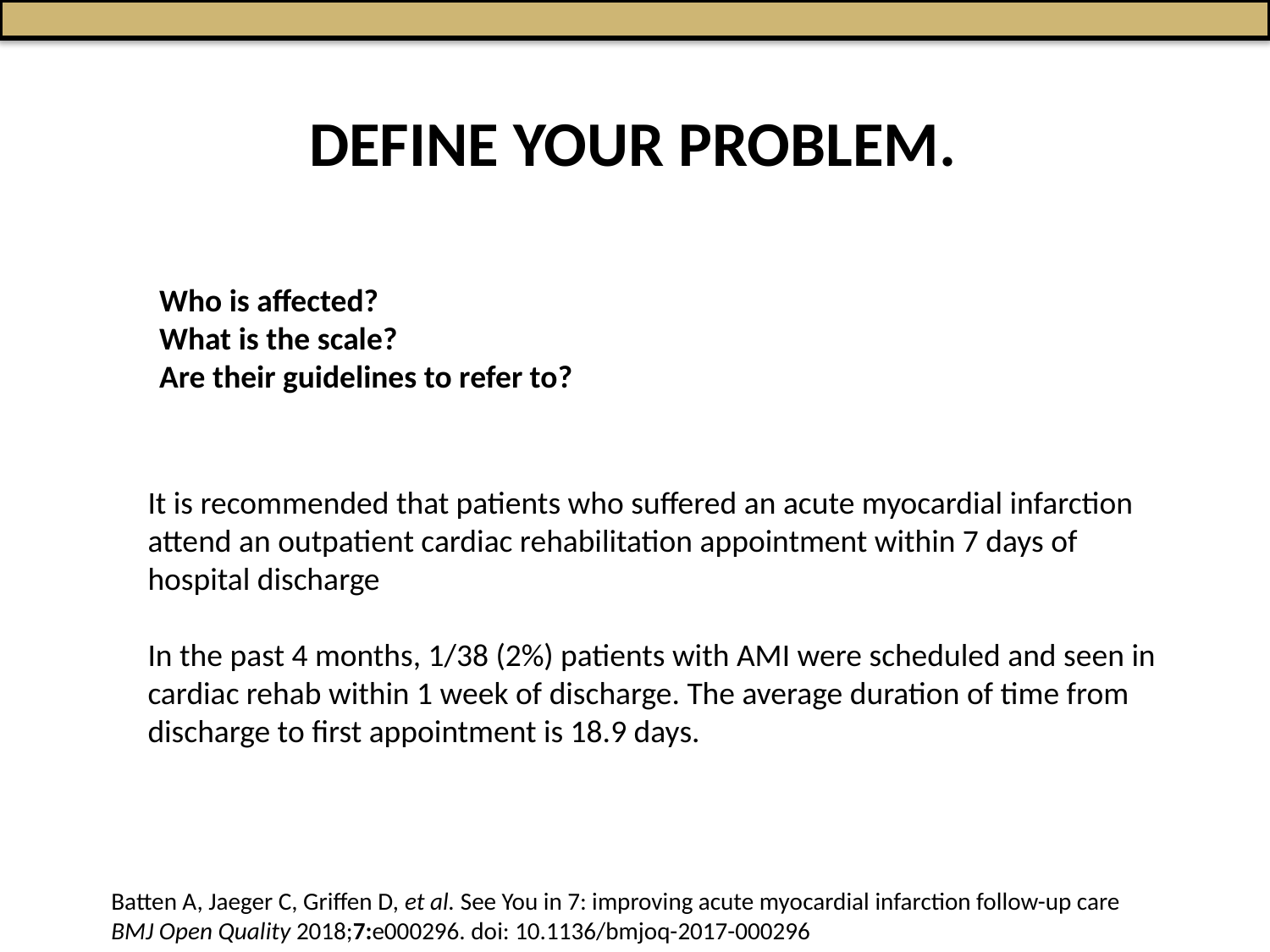

DEFINE YOUR PROBLEM.
Who is affected?
What is the scale?
Are their guidelines to refer to?
It is recommended that patients who suffered an acute myocardial infarction attend an outpatient cardiac rehabilitation appointment within 7 days of hospital discharge
In the past 4 months, 1/38 (2%) patients with AMI were scheduled and seen in cardiac rehab within 1 week of discharge. The average duration of time from discharge to first appointment is 18.9 days.
Batten A, Jaeger C, Griffen D, et al. See You in 7: improving acute myocardial infarction follow-up care
BMJ Open Quality 2018;7:e000296. doi: 10.1136/bmjoq-2017-000296

## Slide 25
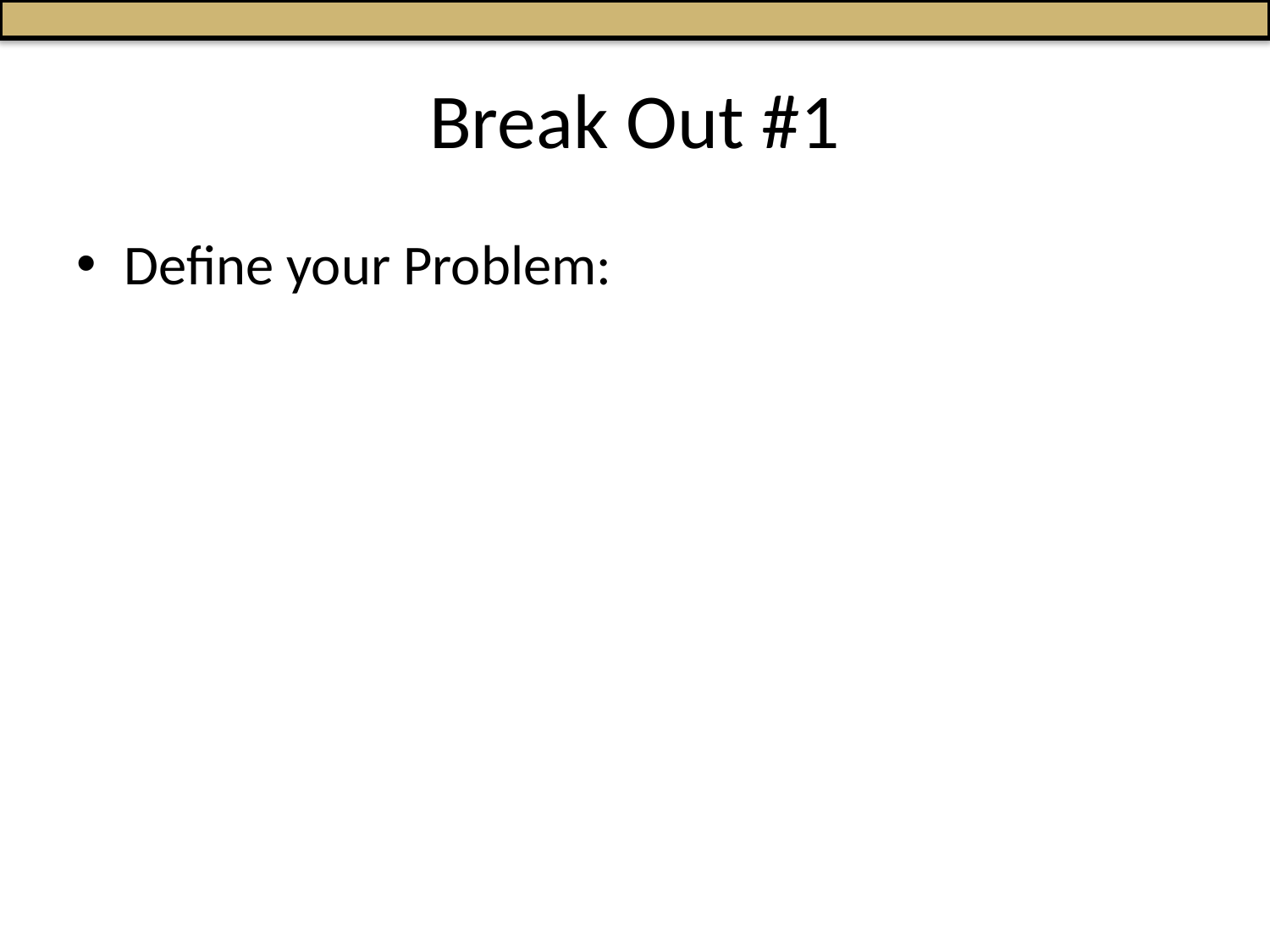

# Break Out #1
Define your Problem:

## Slide 26
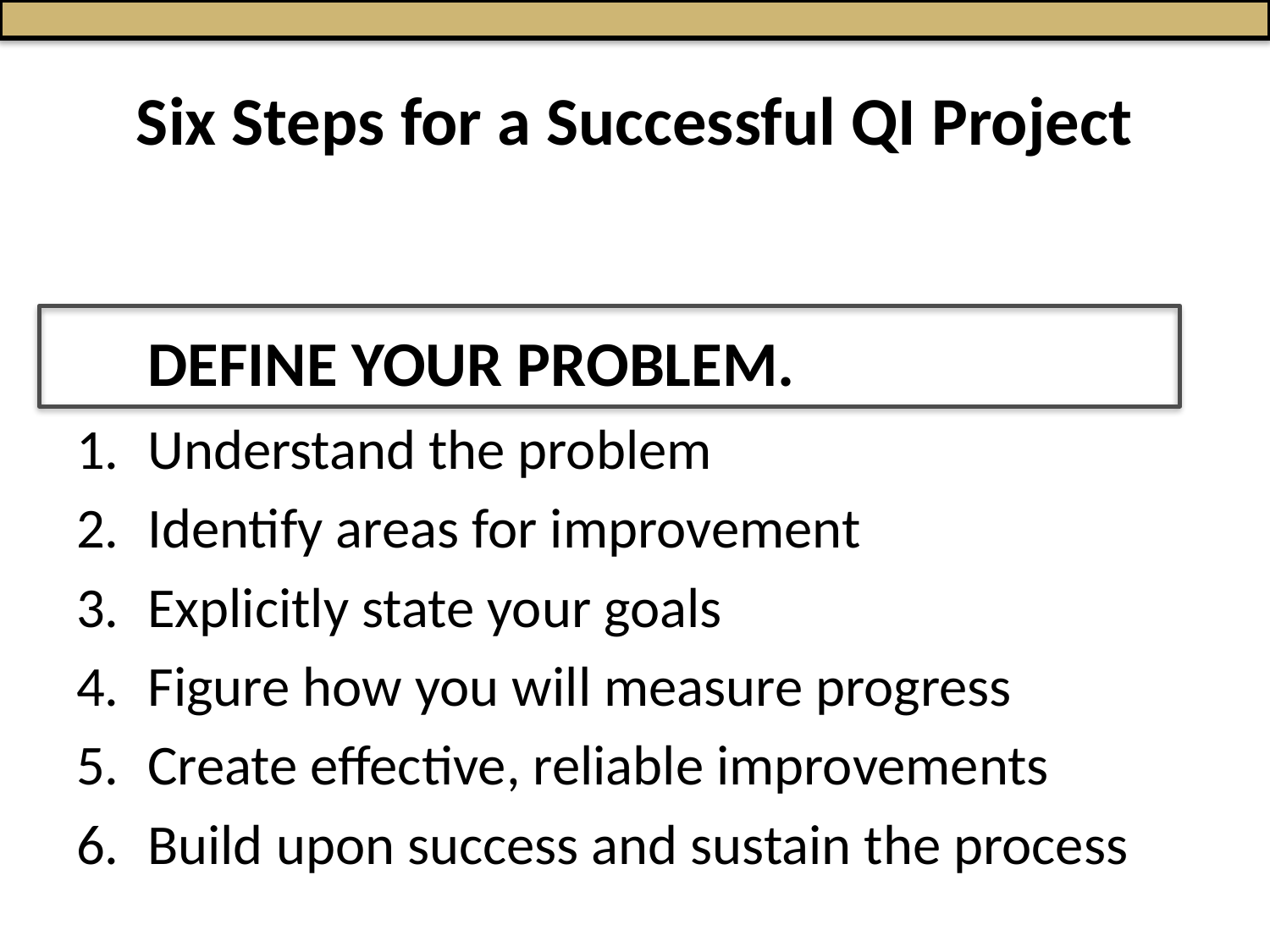

# Six Steps for a Successful QI Project
DEFINE YOUR PROBLEM.
Understand the problem
Identify areas for improvement
Explicitly state your goals
Figure how you will measure progress
Create effective, reliable improvements
Build upon success and sustain the process

## Slide 27
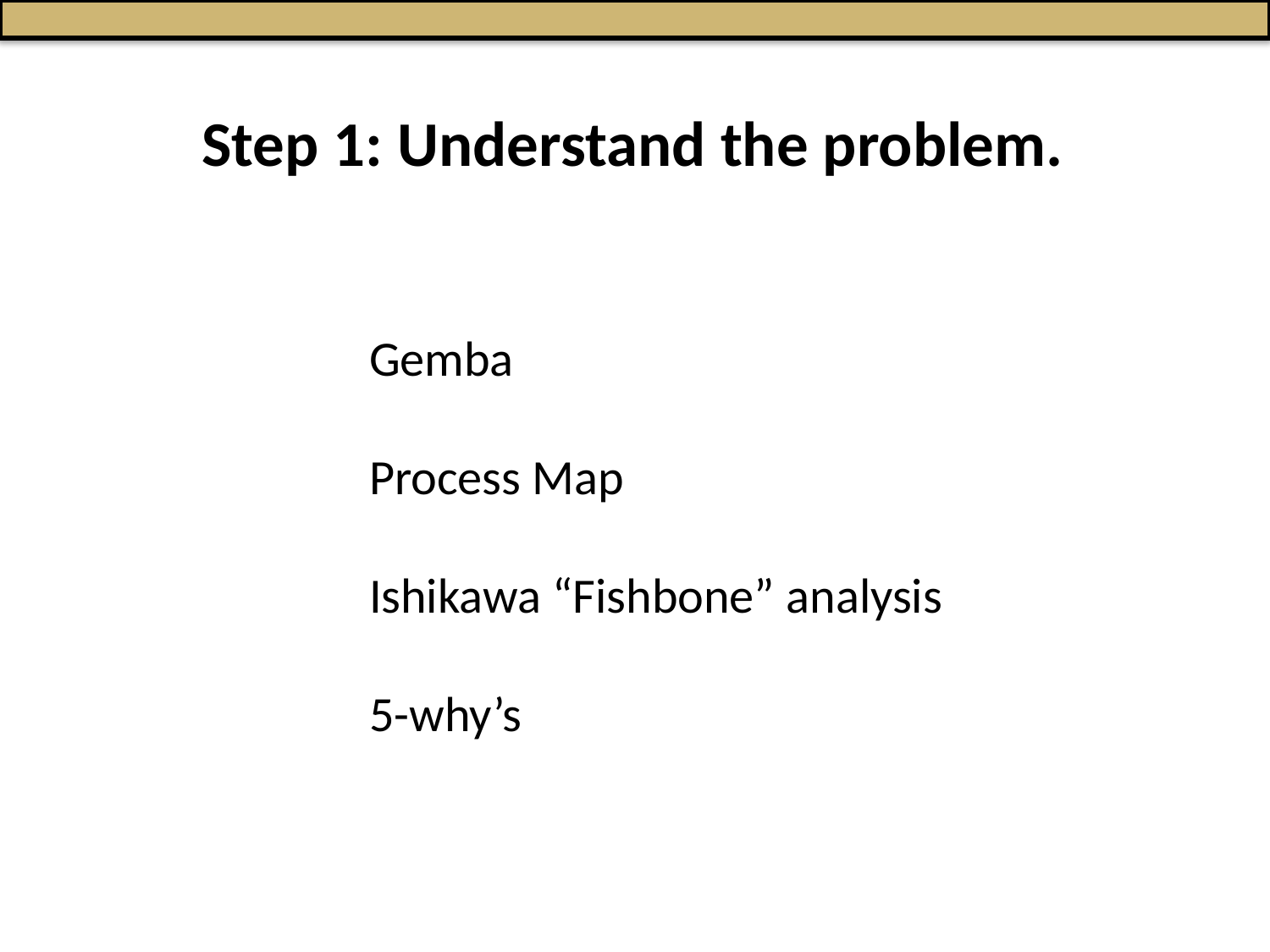

Step 1: Understand the problem.
Gemba
Process Map
Ishikawa “Fishbone” analysis
5-why’s

## Slide 28
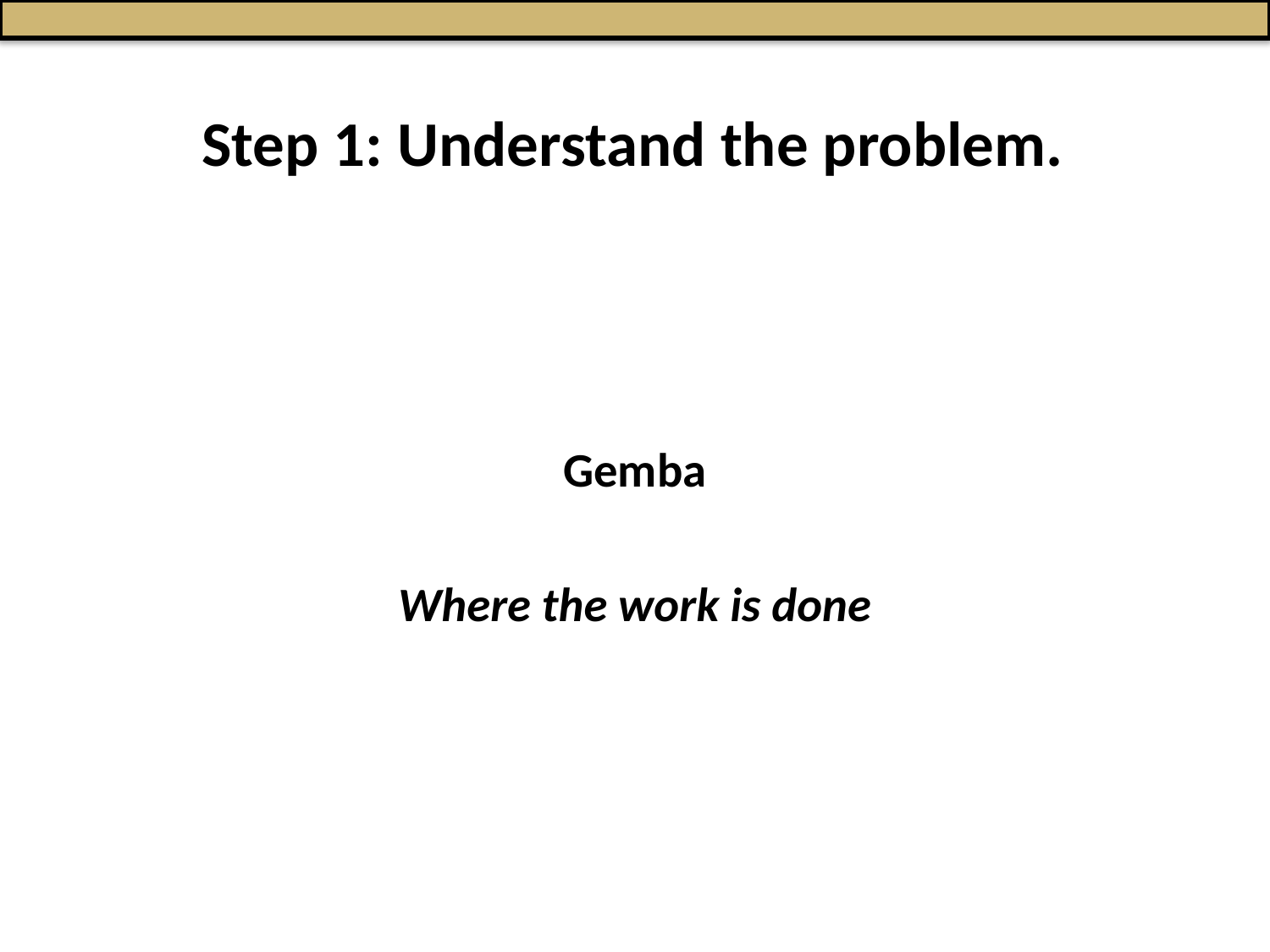

Step 1: Understand the problem.
Gemba
Where the work is done

## Slide 29
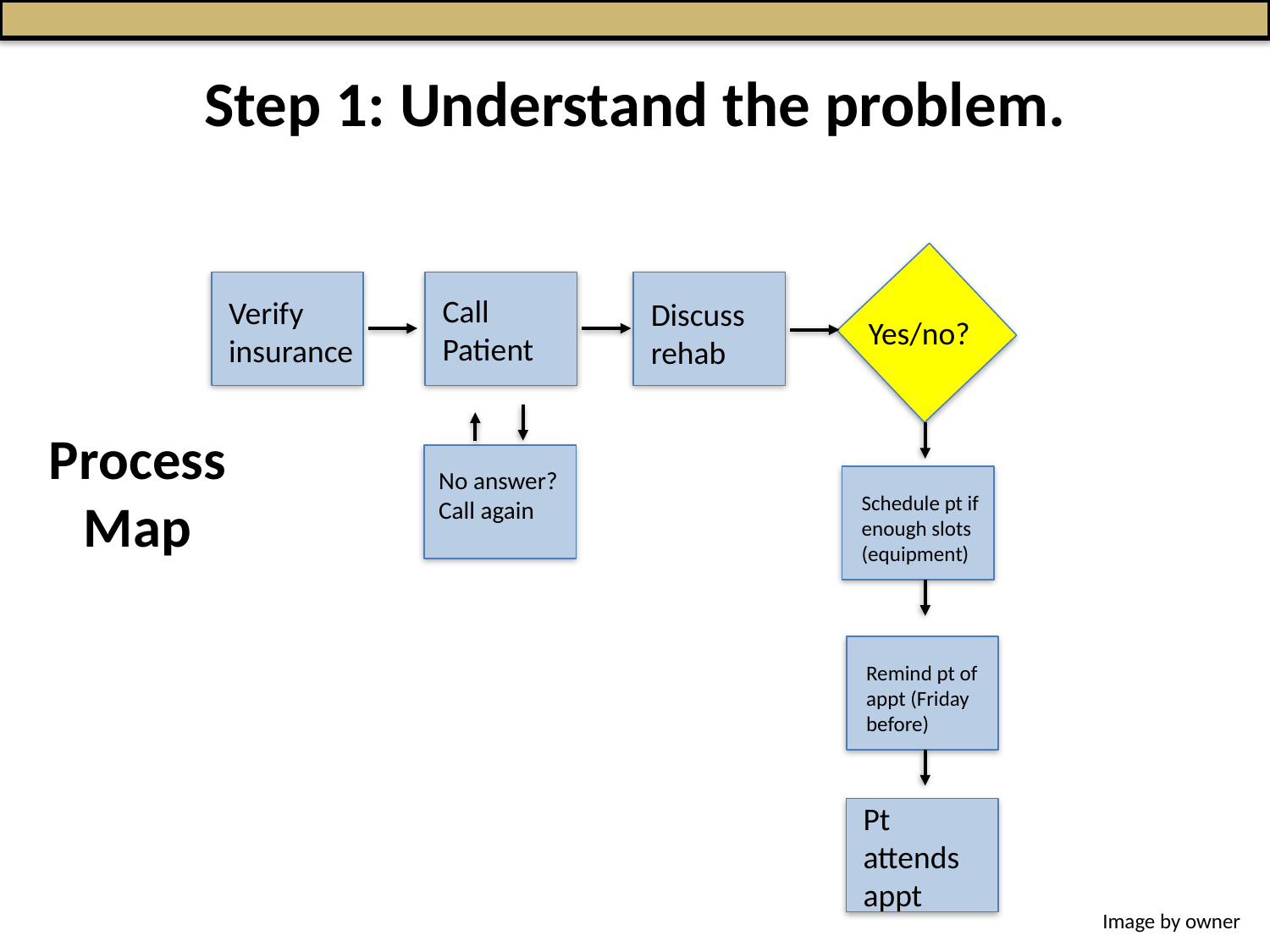

Step 1: Understand the problem.
Call Patient
Verify insurance
Discuss rehab
Yes/no?
Process Map
No answer? Call again
Schedule pt if enough slots (equipment)
Remind pt of appt (Friday before)
Pt attends appt
Image by owner

## Slide 30
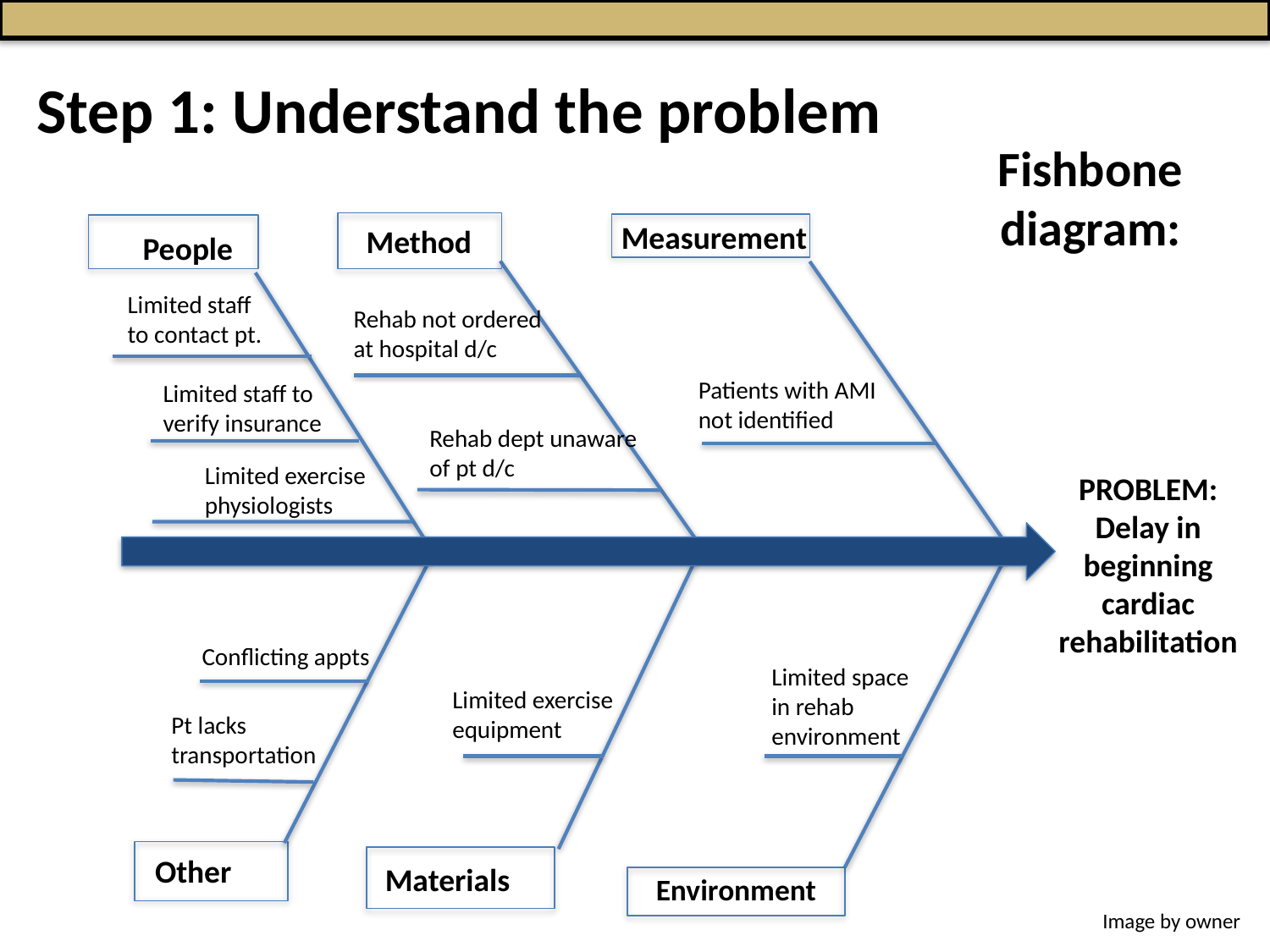

Step 1: Understand the problem
Fishbone diagram:
Measurement
Method
People
Limited staff to contact pt.
Rehab not ordered at hospital d/c
Patients with AMI not identified
Limited staff to verify insurance
Rehab dept unaware of pt d/c
Limited exercise physiologists
PROBLEM:
Delay in beginning cardiac rehabilitation
Conflicting appts
Limited space in rehab environment
Limited exercise equipment
Pt lacks transportation
Other
Materials
Environment
Image by owner

## Slide 31
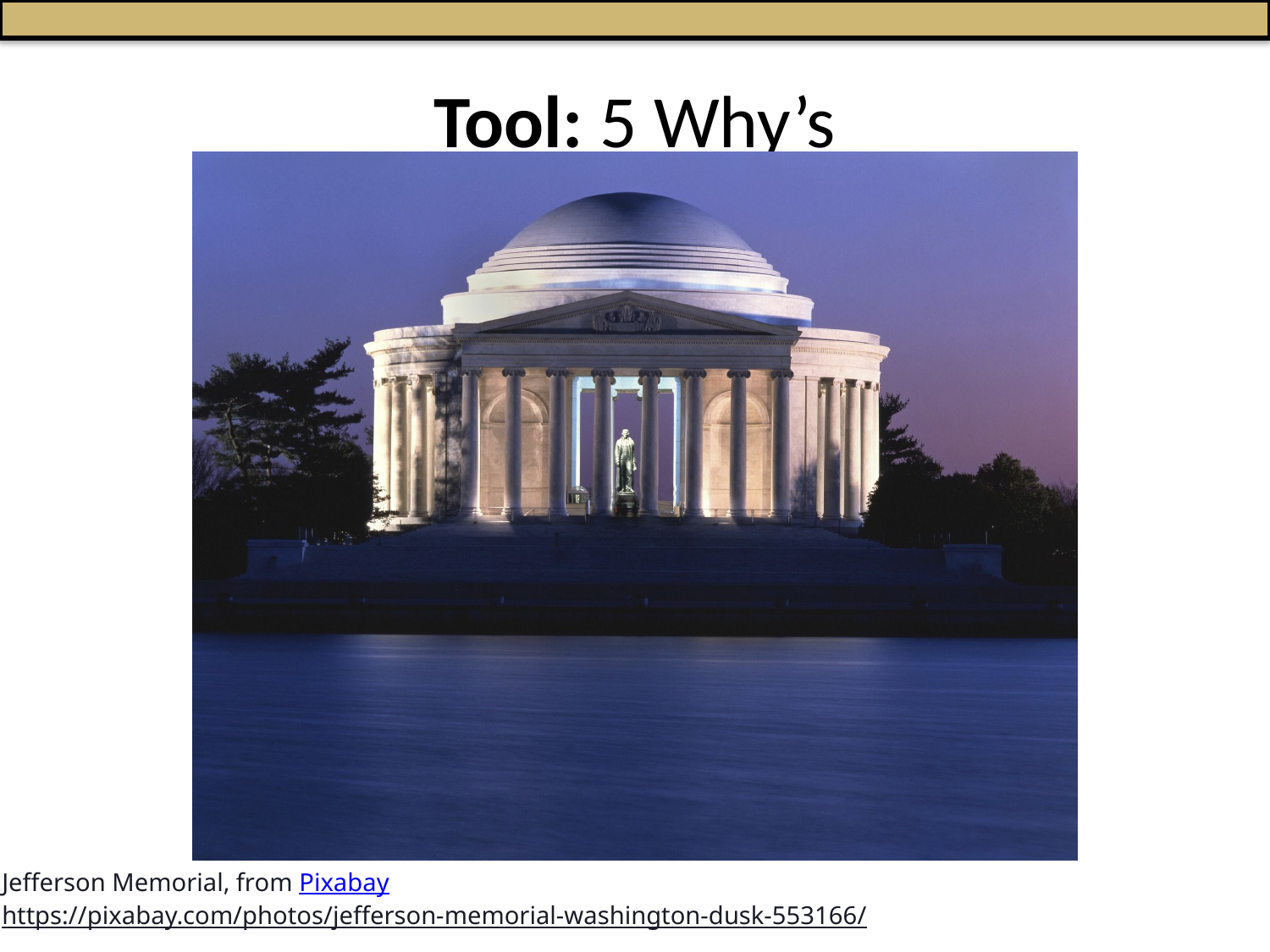

Tool: 5 Why’s
Jefferson Memorial, from Pixabay
https://pixabay.com/photos/jefferson-memorial-washington-dusk-553166/

## Slide 32
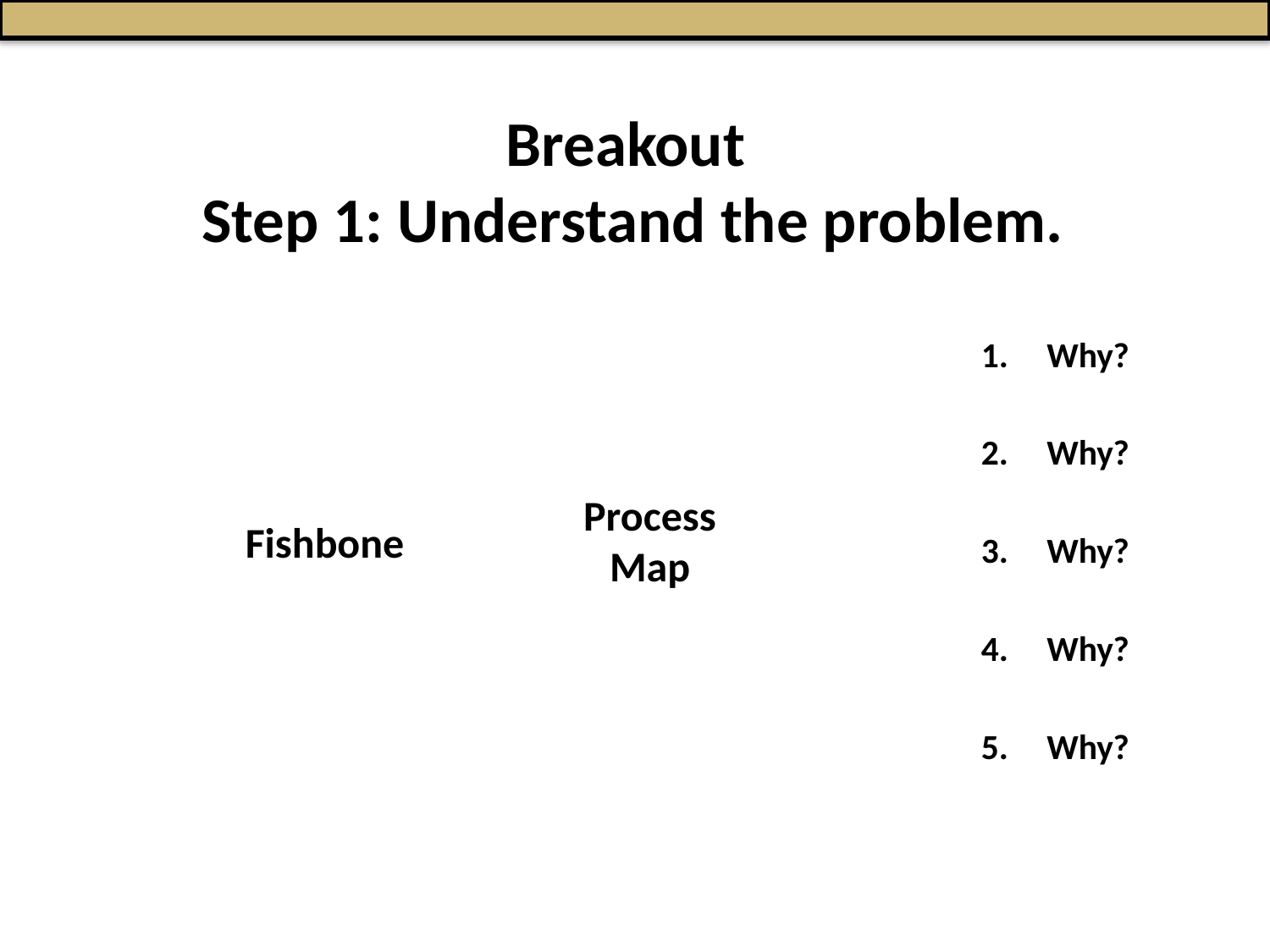

Breakout
Step 1: Understand the problem.
Why?
Why?
Why?
Why?
Why?
Process Map
Fishbone

## Slide 33
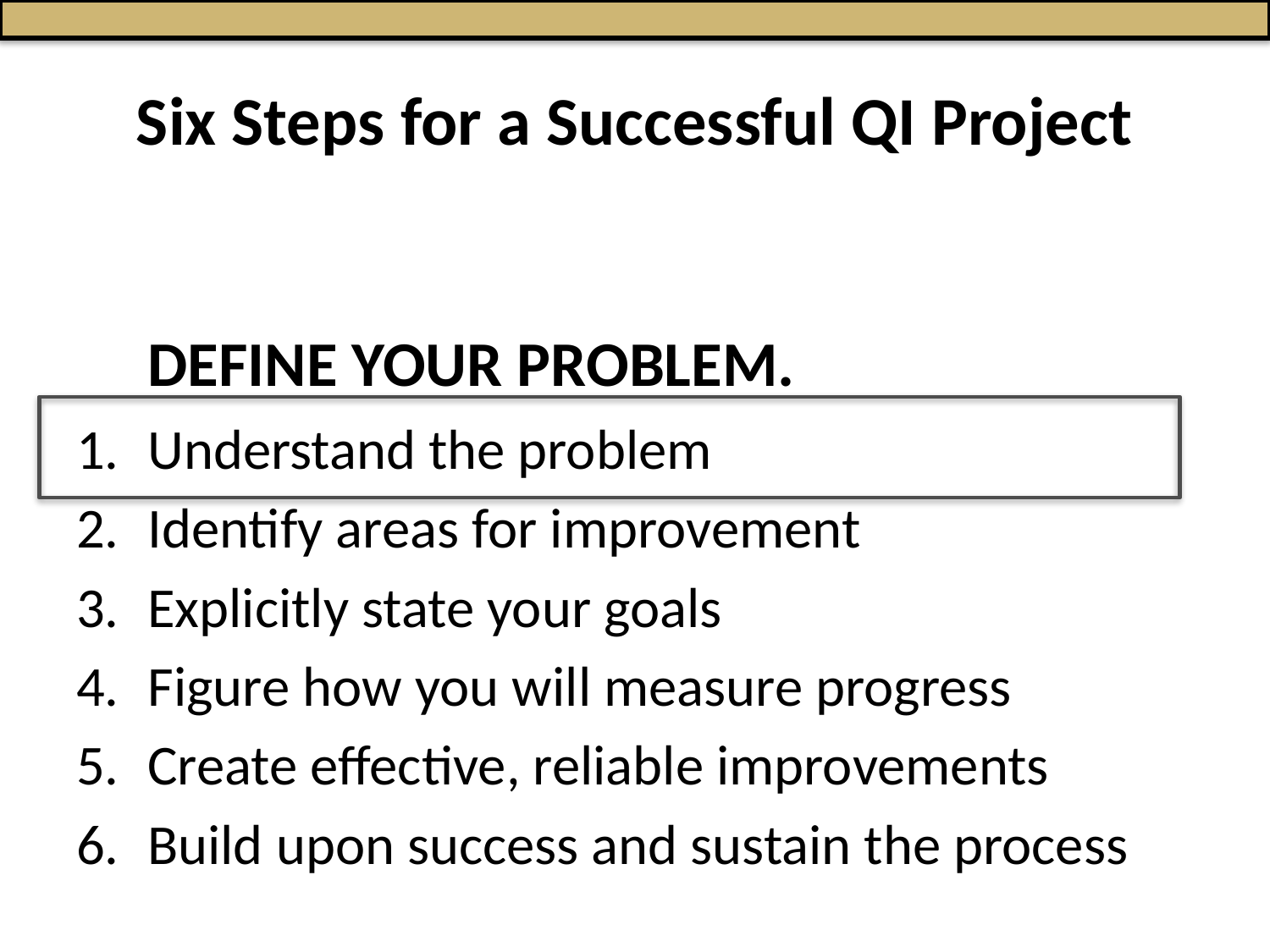

# Six Steps for a Successful QI Project
DEFINE YOUR PROBLEM.
Understand the problem
Identify areas for improvement
Explicitly state your goals
Figure how you will measure progress
Create effective, reliable improvements
Build upon success and sustain the process

## Slide 34
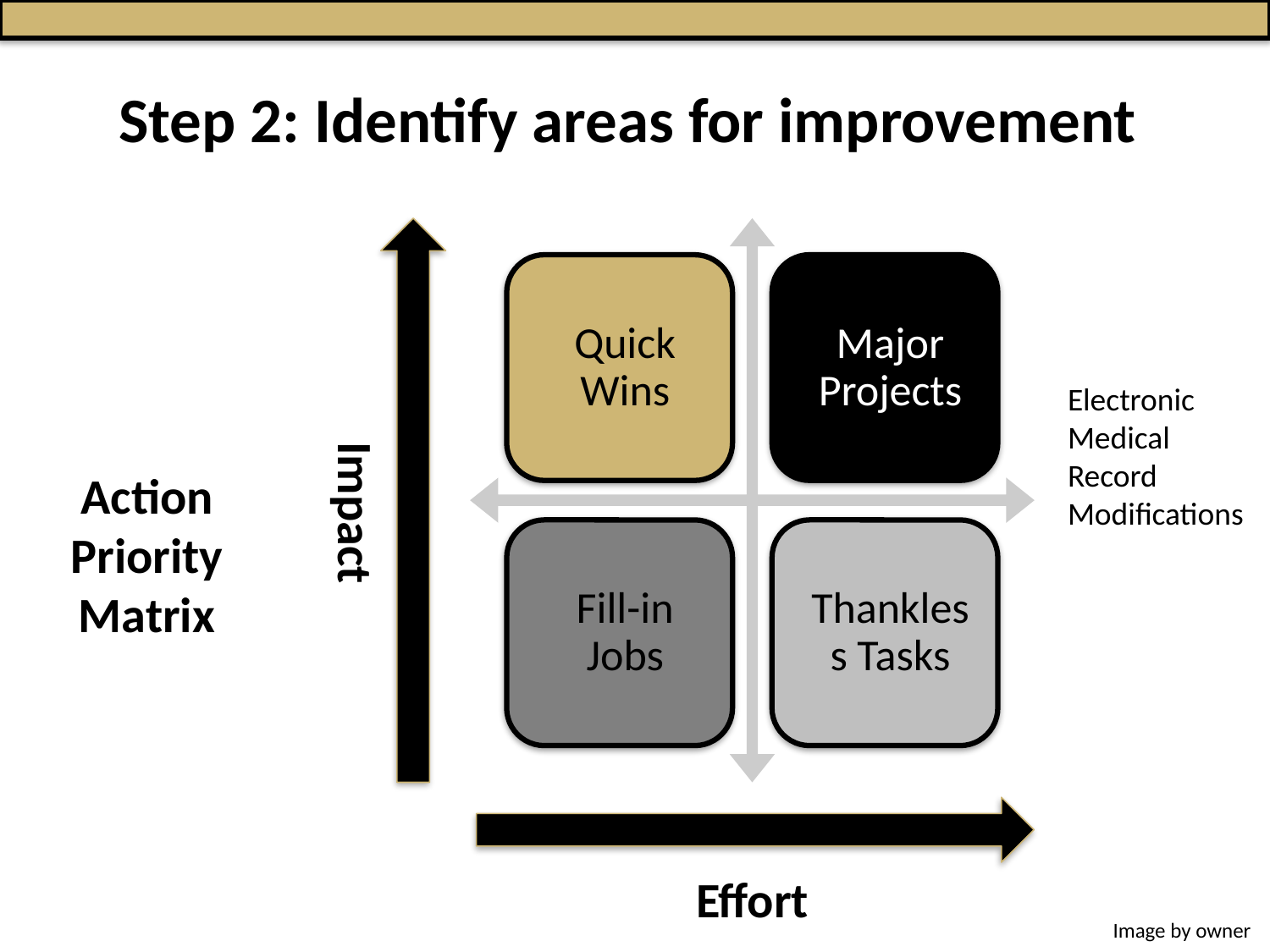

# Step 2: Identify areas for improvement
Impact
Electronic Medical Record Modifications
Action Priority Matrix
Effort
Image by owner

## Slide 35
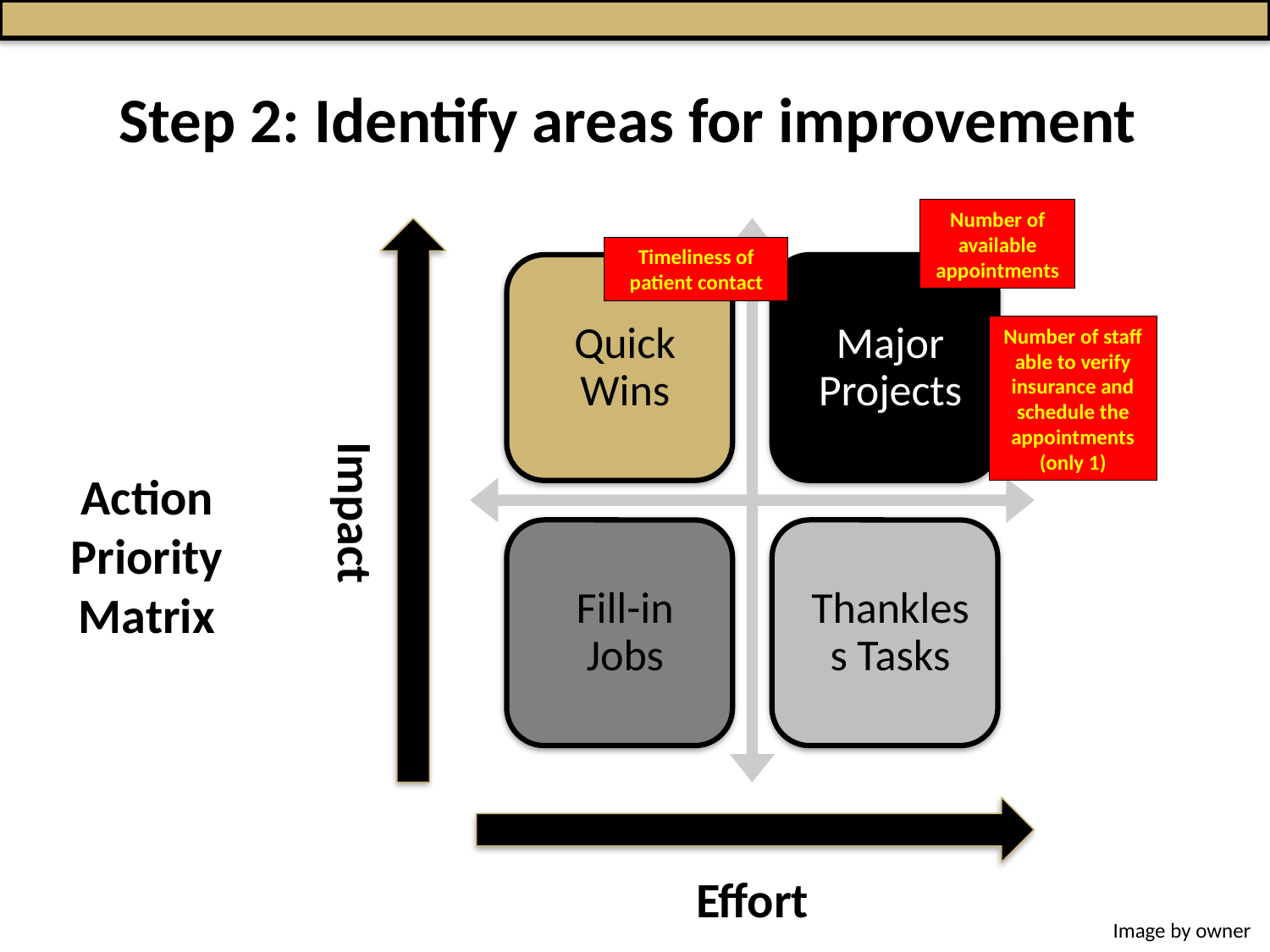

# Step 2: Identify areas for improvement
Number of available appointments
Timeliness of patient contact
Number of staff able to verify insurance and schedule the appointments (only 1)
Impact
Action Priority Matrix
Effort
Image by owner

## Slide 36
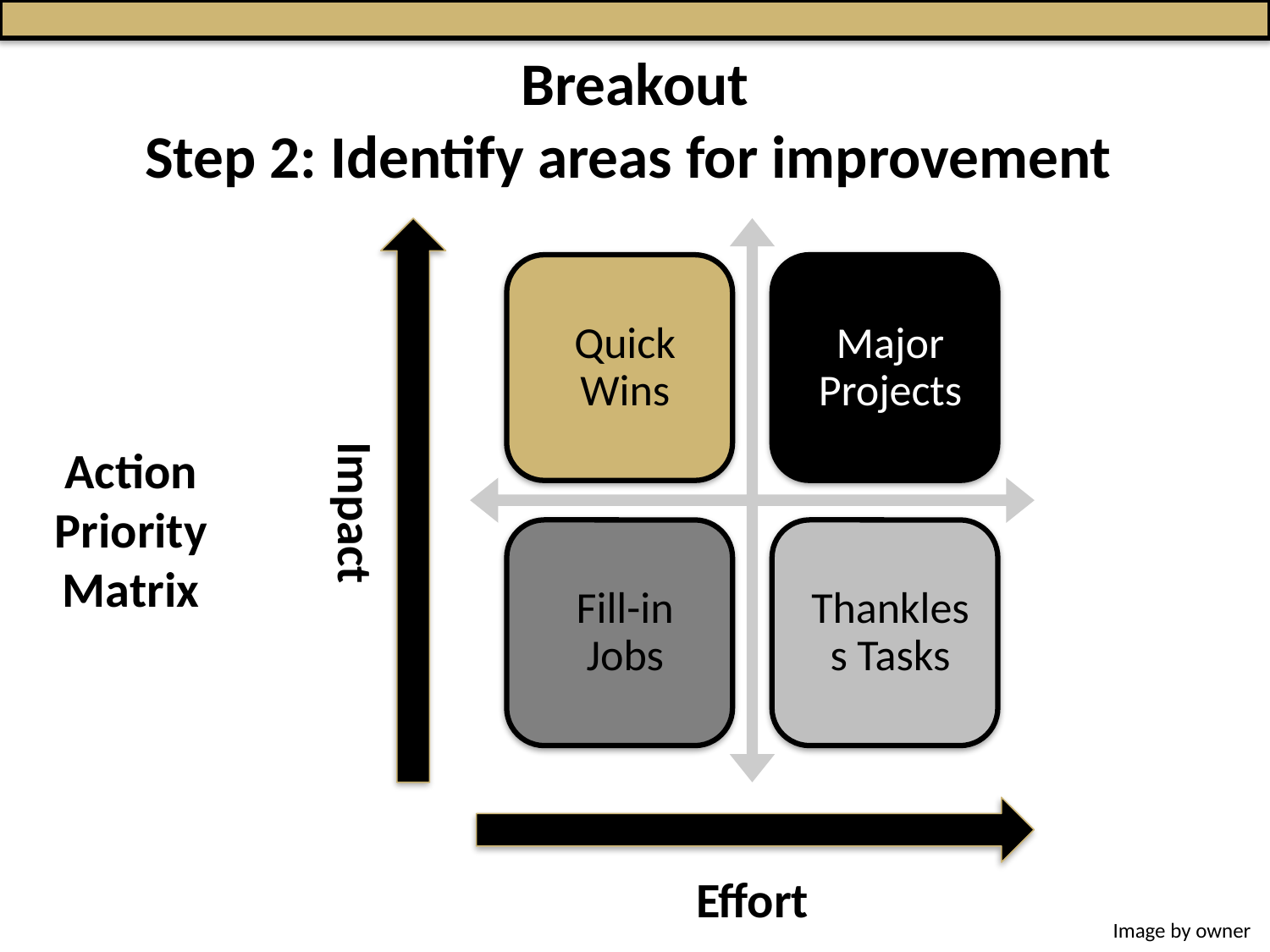

# Breakout Step 2: Identify areas for improvement
Impact
Action Priority Matrix
Effort
Image by owner

## Slide 37
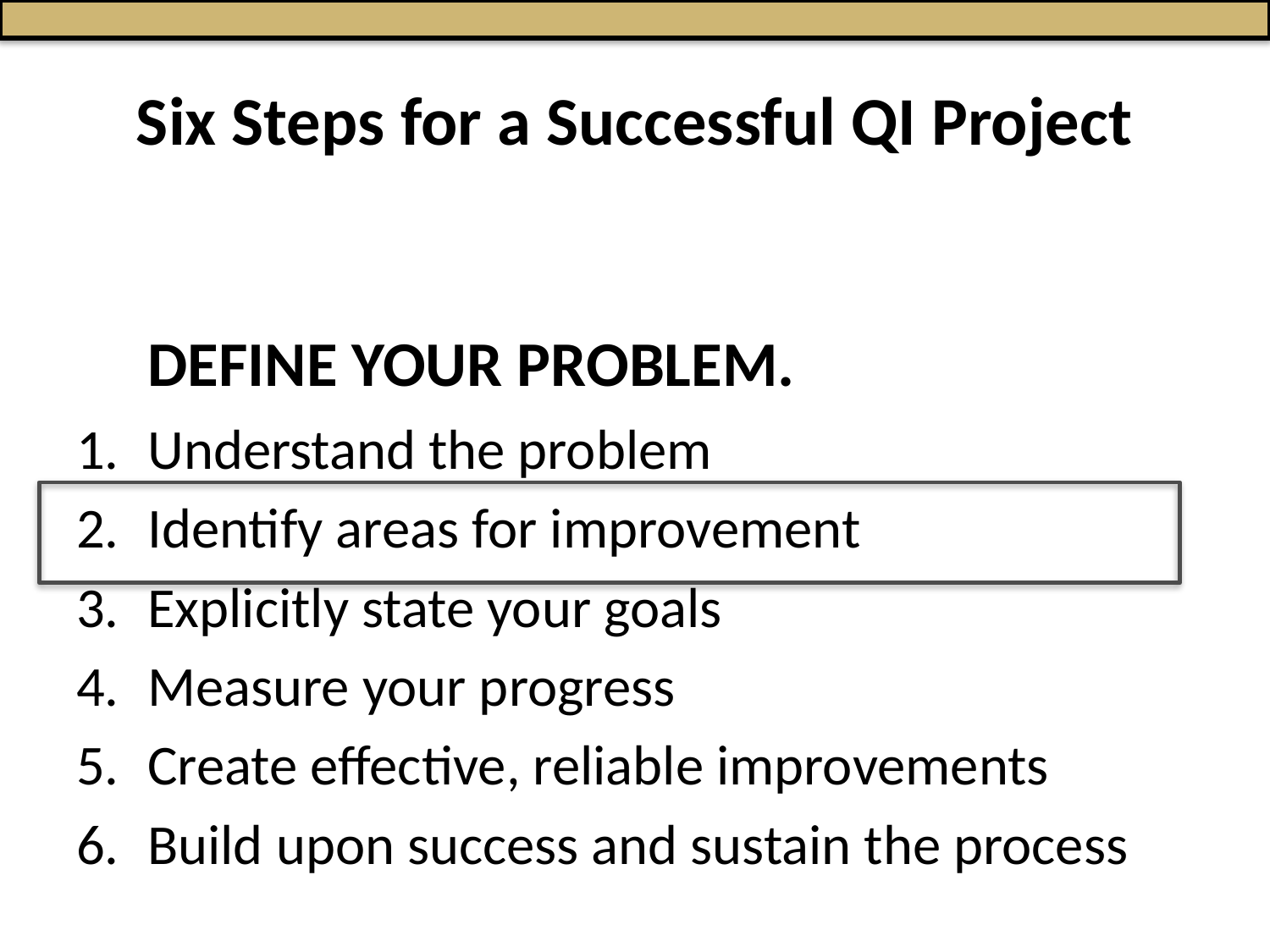

# Six Steps for a Successful QI Project
DEFINE YOUR PROBLEM.
Understand the problem
Identify areas for improvement
Explicitly state your goals
Measure your progress
Create effective, reliable improvements
Build upon success and sustain the process

## Slide 38
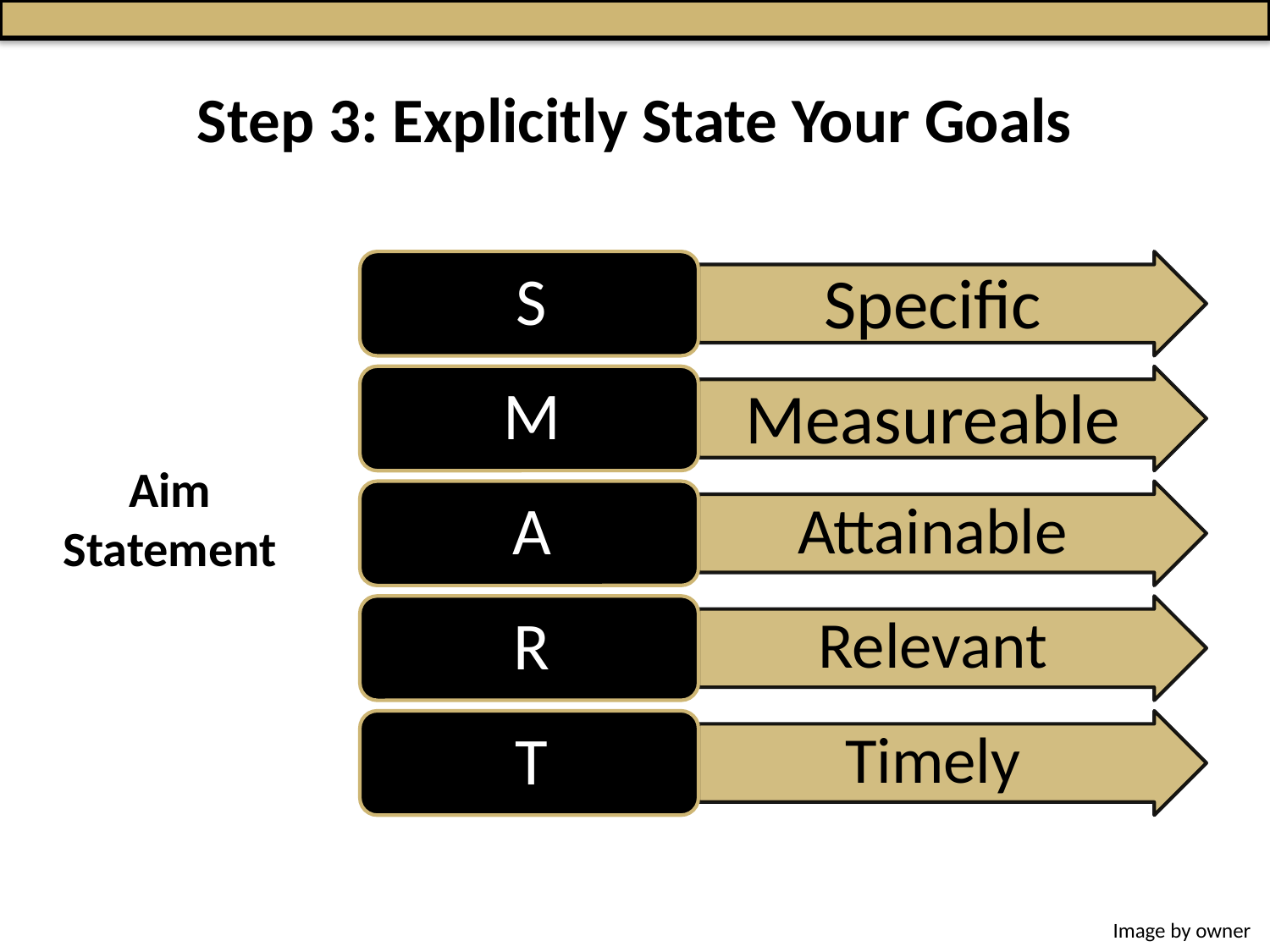

# Step 3: Explicitly State Your Goals
Aim Statement
Image by owner

## Slide 39
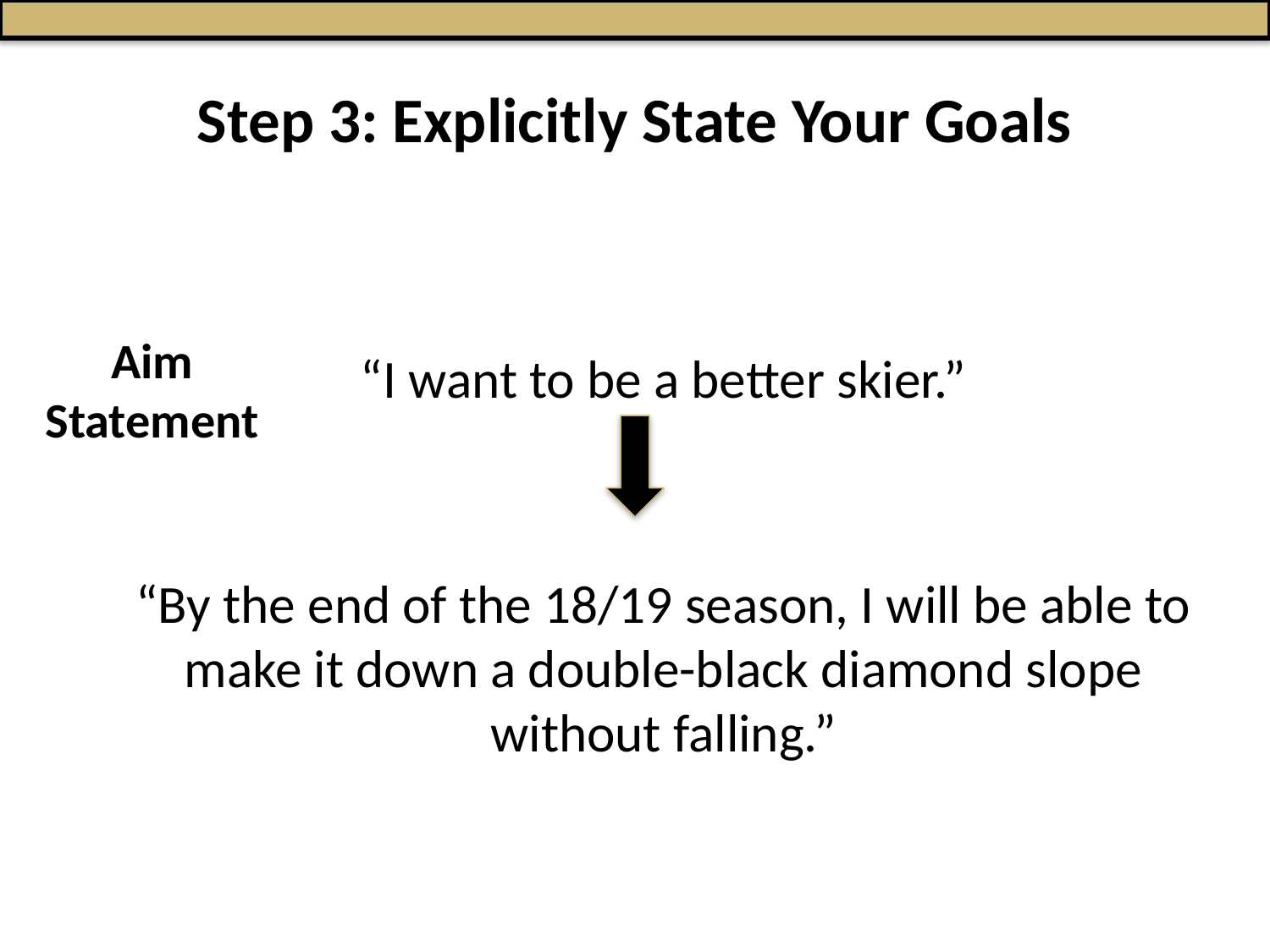

# Step 3: Explicitly State Your Goals
“I want to be a better skier.”
“By the end of the 18/19 season, I will be able to make it down a double-black diamond slope without falling.”
Aim Statement

## Slide 40
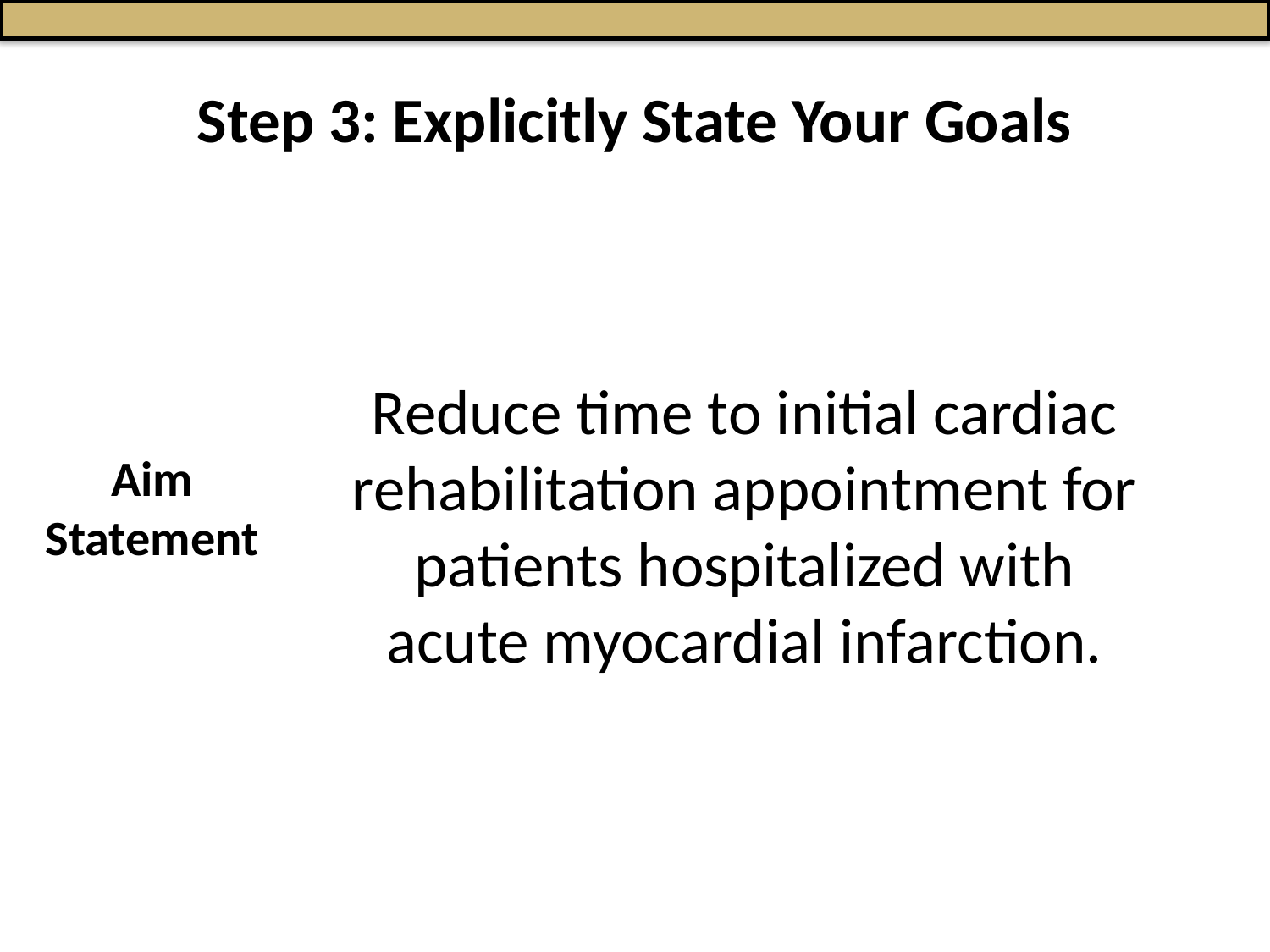

# Step 3: Explicitly State Your Goals
Reduce time to initial cardiac rehabilitation appointment for patients hospitalized with acute myocardial infarction.
Aim Statement

## Slide 41
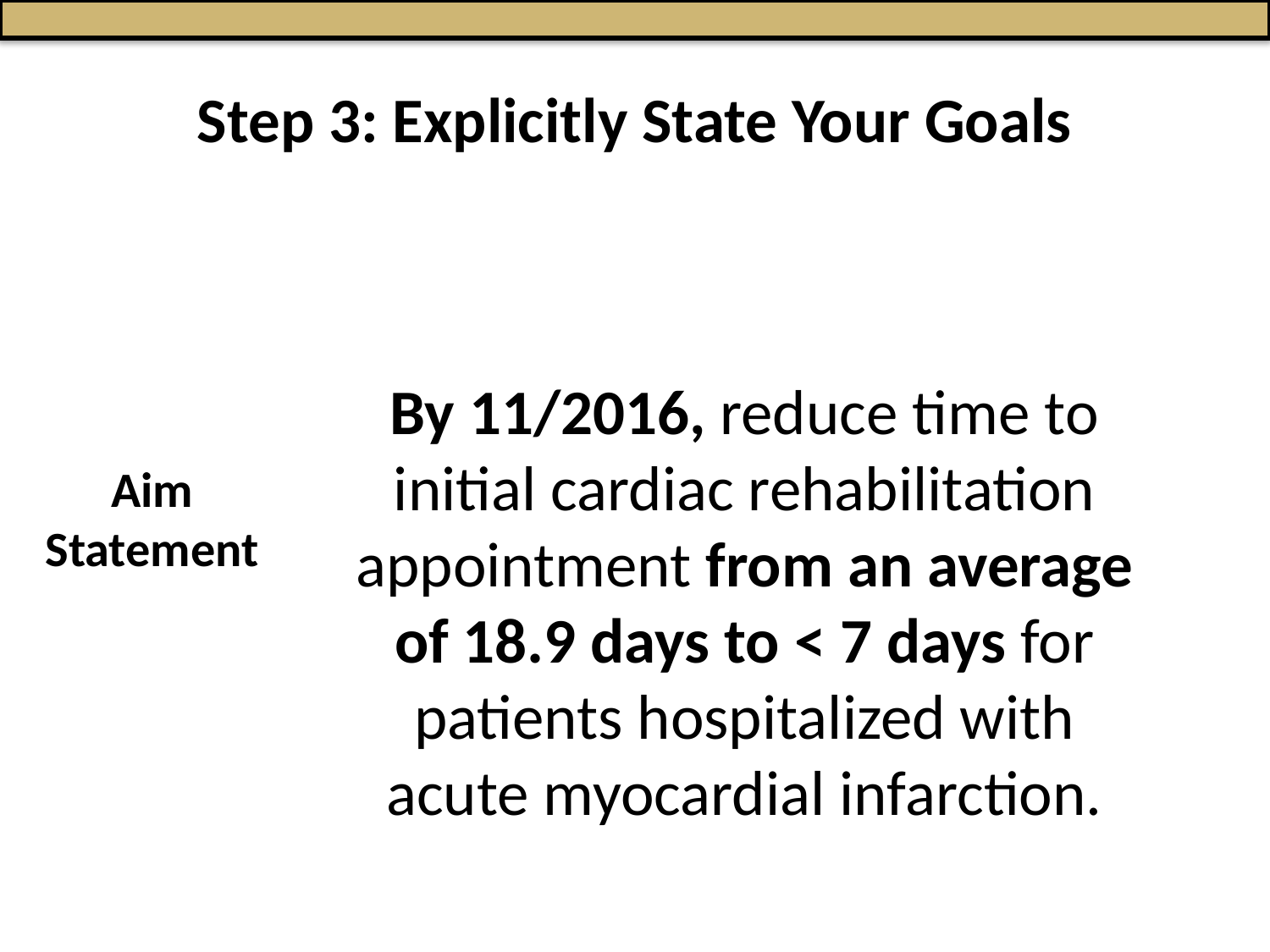

# Step 3: Explicitly State Your Goals
By 11/2016, reduce time to initial cardiac rehabilitation appointment from an average of 18.9 days to < 7 days for patients hospitalized with acute myocardial infarction.
Aim Statement

## Slide 42
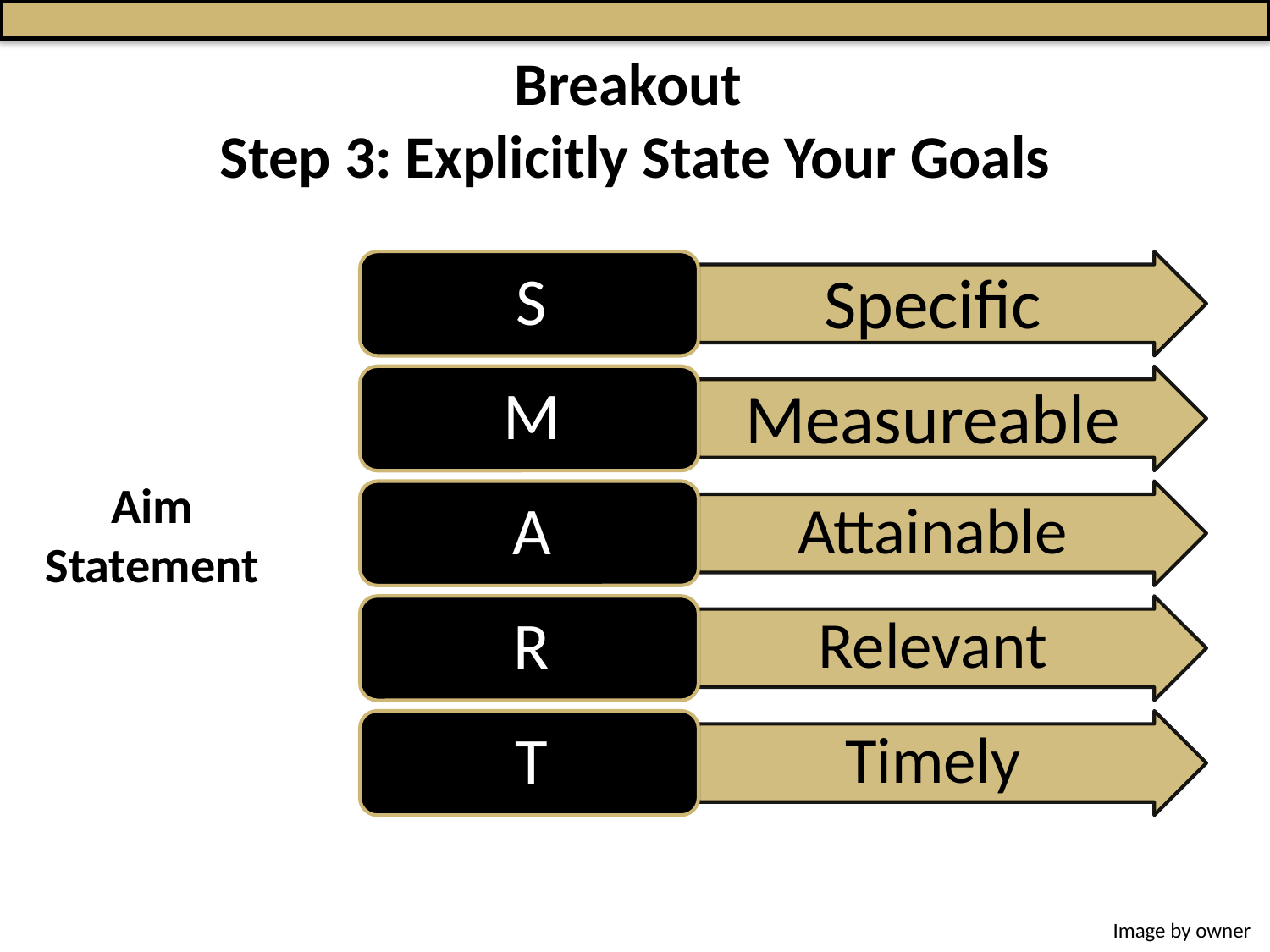

# Breakout Step 3: Explicitly State Your Goals
Aim Statement
Image by owner

## Slide 43
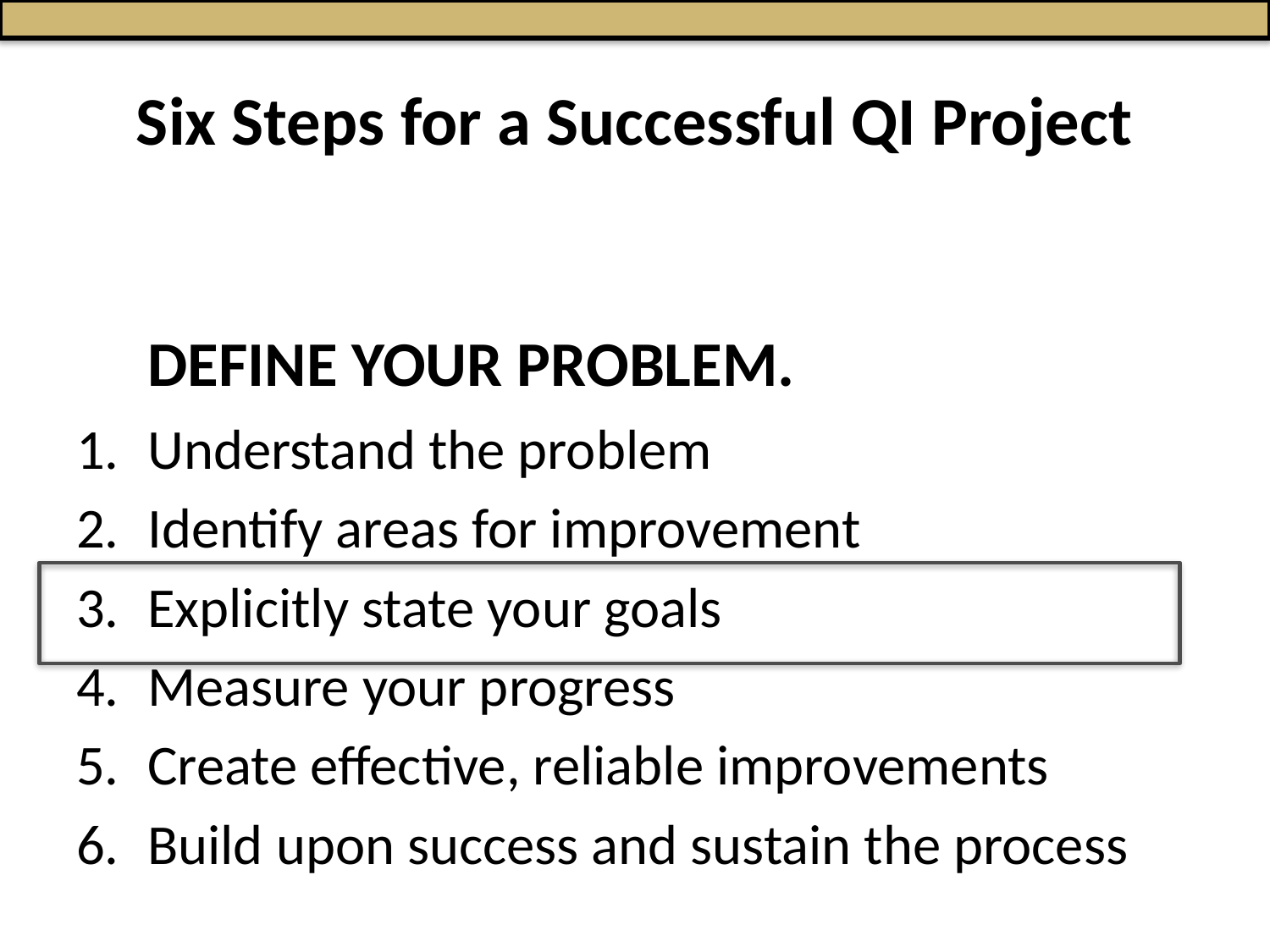

# Six Steps for a Successful QI Project
DEFINE YOUR PROBLEM.
Understand the problem
Identify areas for improvement
Explicitly state your goals
Measure your progress
Create effective, reliable improvements
Build upon success and sustain the process

## Slide 44
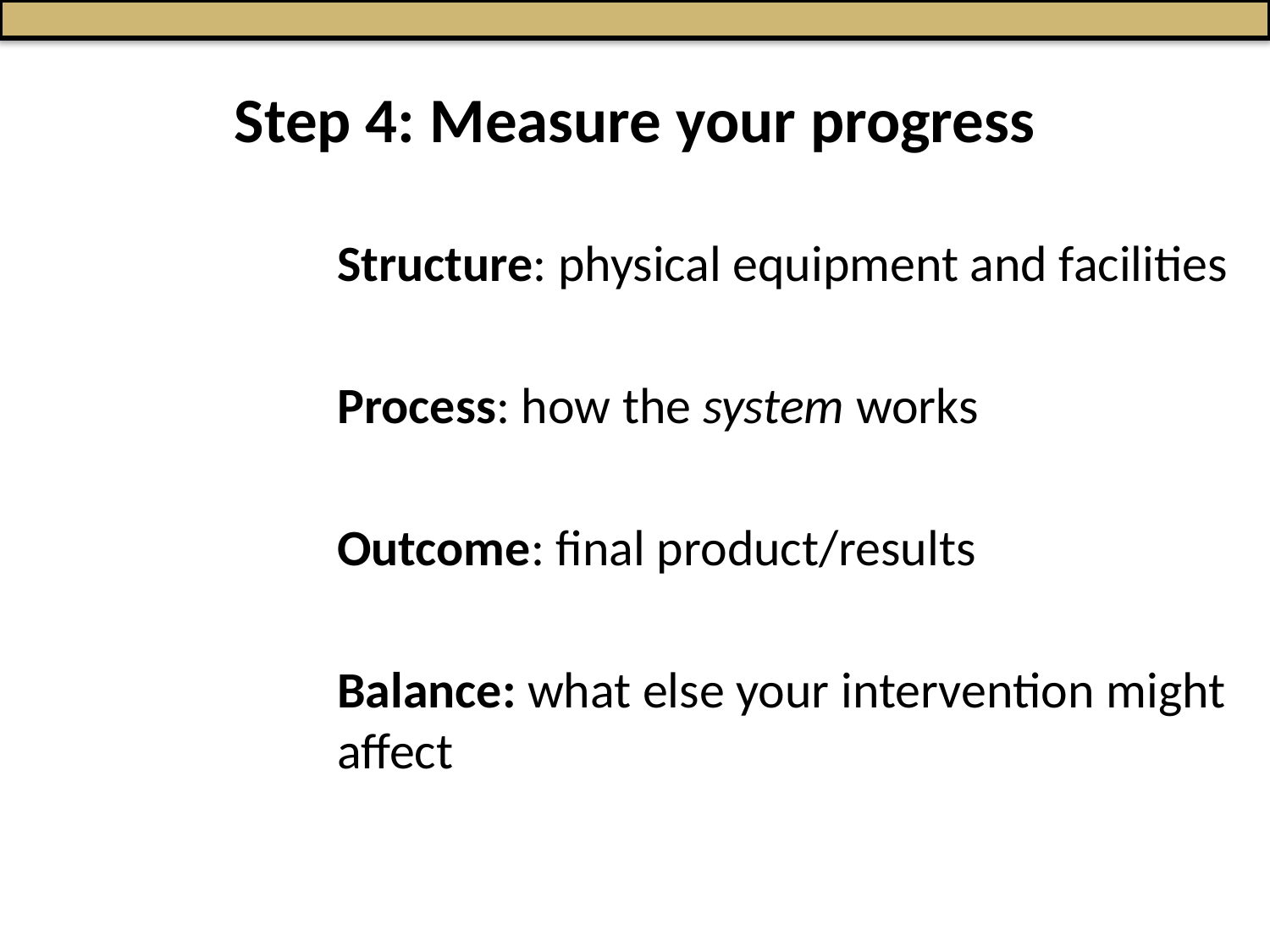

# Step 4: Measure your progress
Structure: physical equipment and facilities
Process: how the system works
Outcome: final product/results
Balance: what else your intervention might affect

## Slide 45
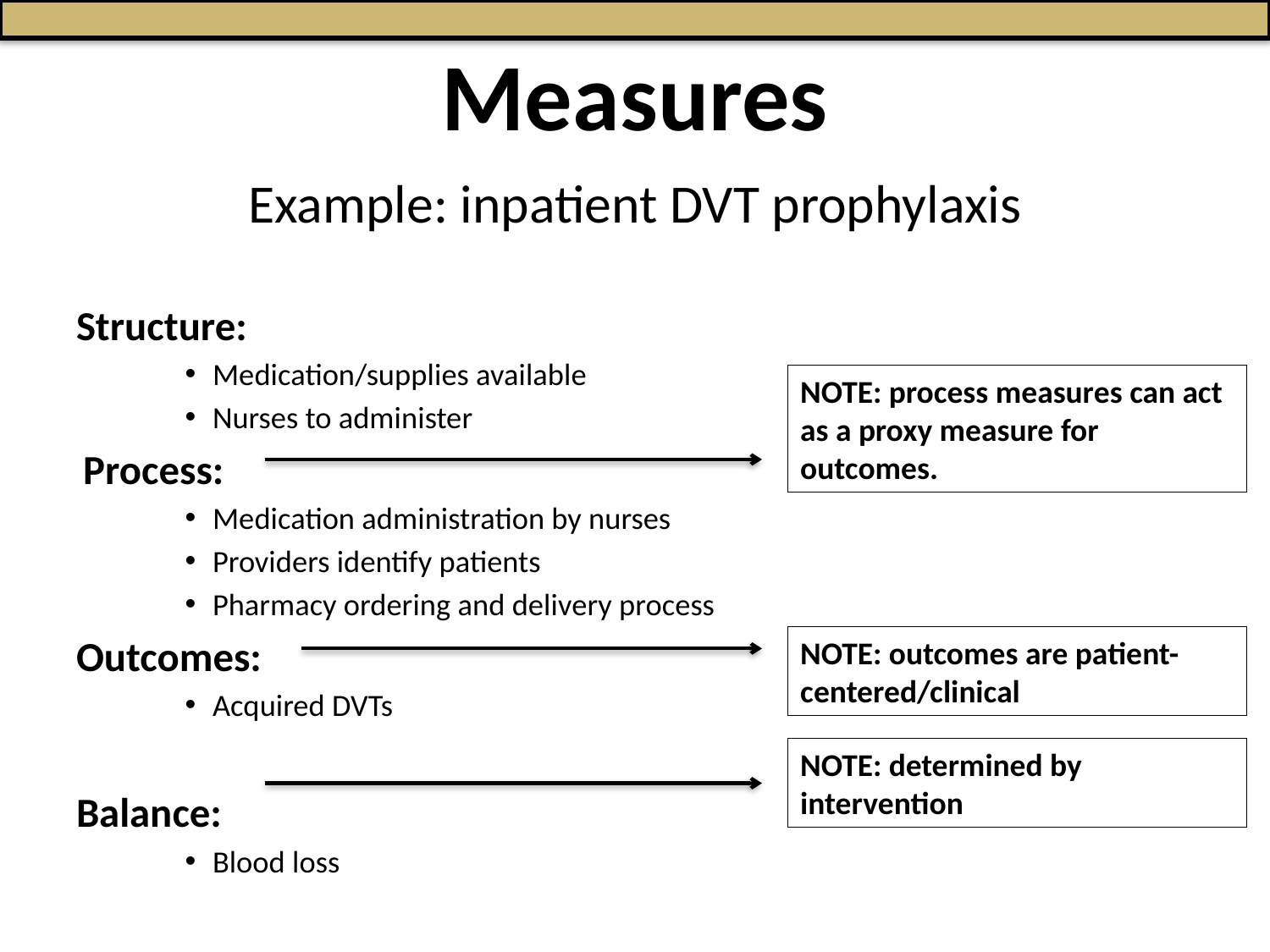

# Measures
Example: inpatient DVT prophylaxis
Structure:
Medication/supplies available
Nurses to administer
Process:
Medication administration by nurses
Providers identify patients
Pharmacy ordering and delivery process
Outcomes:
Acquired DVTs
Balance:
Blood loss
NOTE: process measures can act as a proxy measure for outcomes.
NOTE: outcomes are patient-centered/clinical
NOTE: determined by intervention

## Slide 46
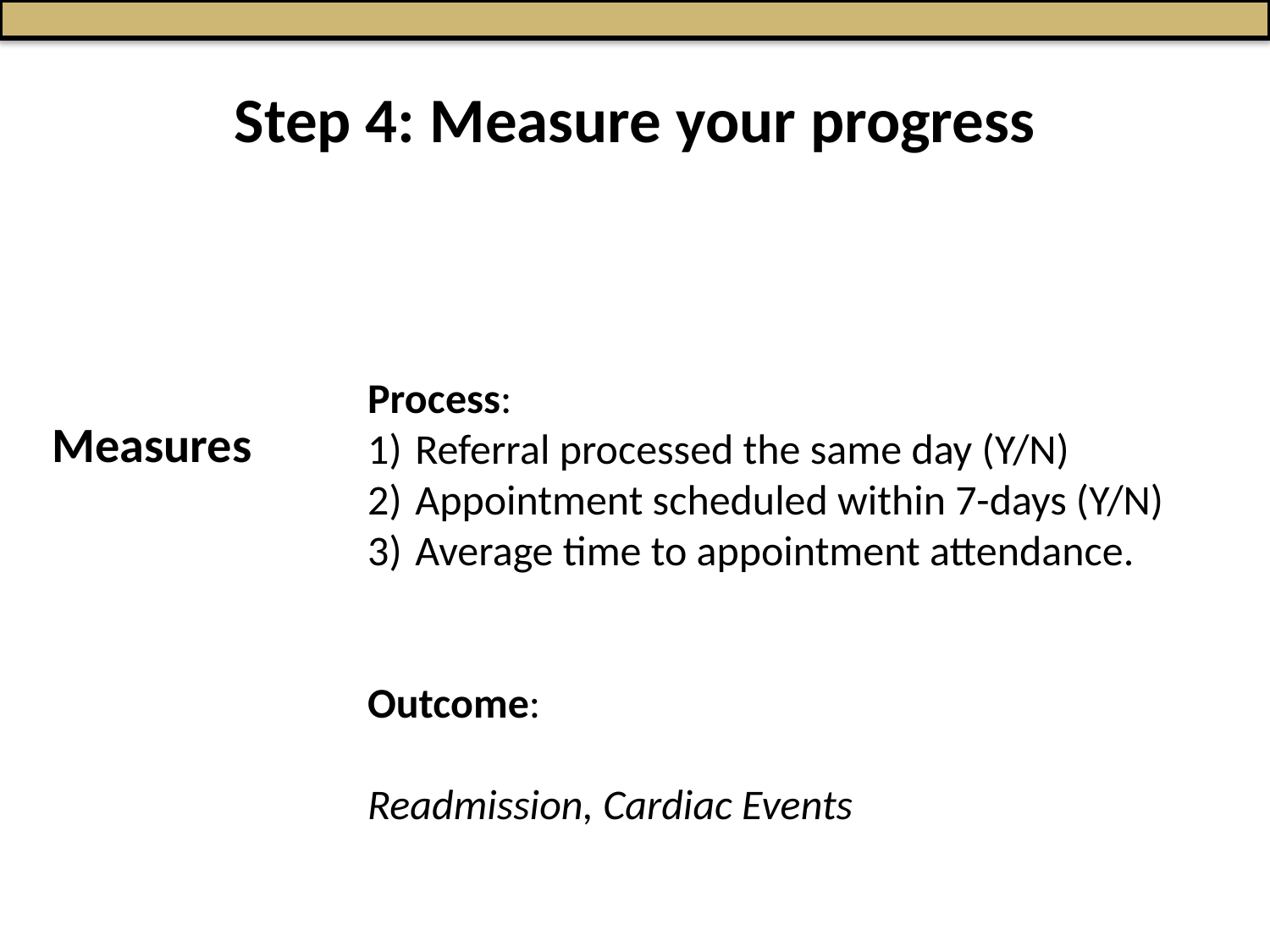

# Step 4: Measure your progress
Measures
Process:
Referral processed the same day (Y/N)
Appointment scheduled within 7-days (Y/N)
Average time to appointment attendance.
Outcome:
Readmission, Cardiac Events

## Slide 47
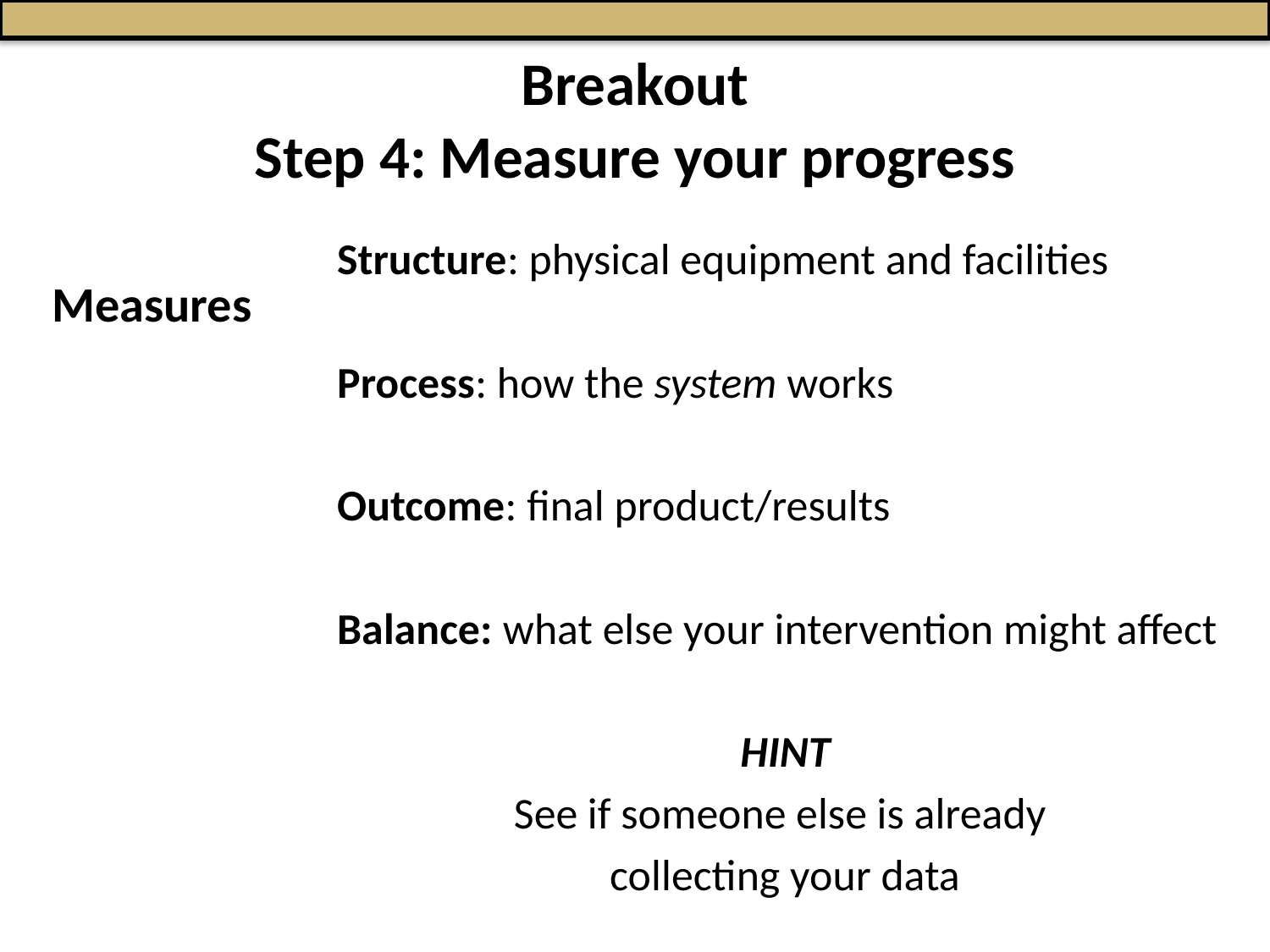

# Breakout Step 4: Measure your progress
Measures
Structure: physical equipment and facilities
Process: how the system works
Outcome: final product/results
Balance: what else your intervention might affect
HINT
See if someone else is already
collecting your data

## Slide 48
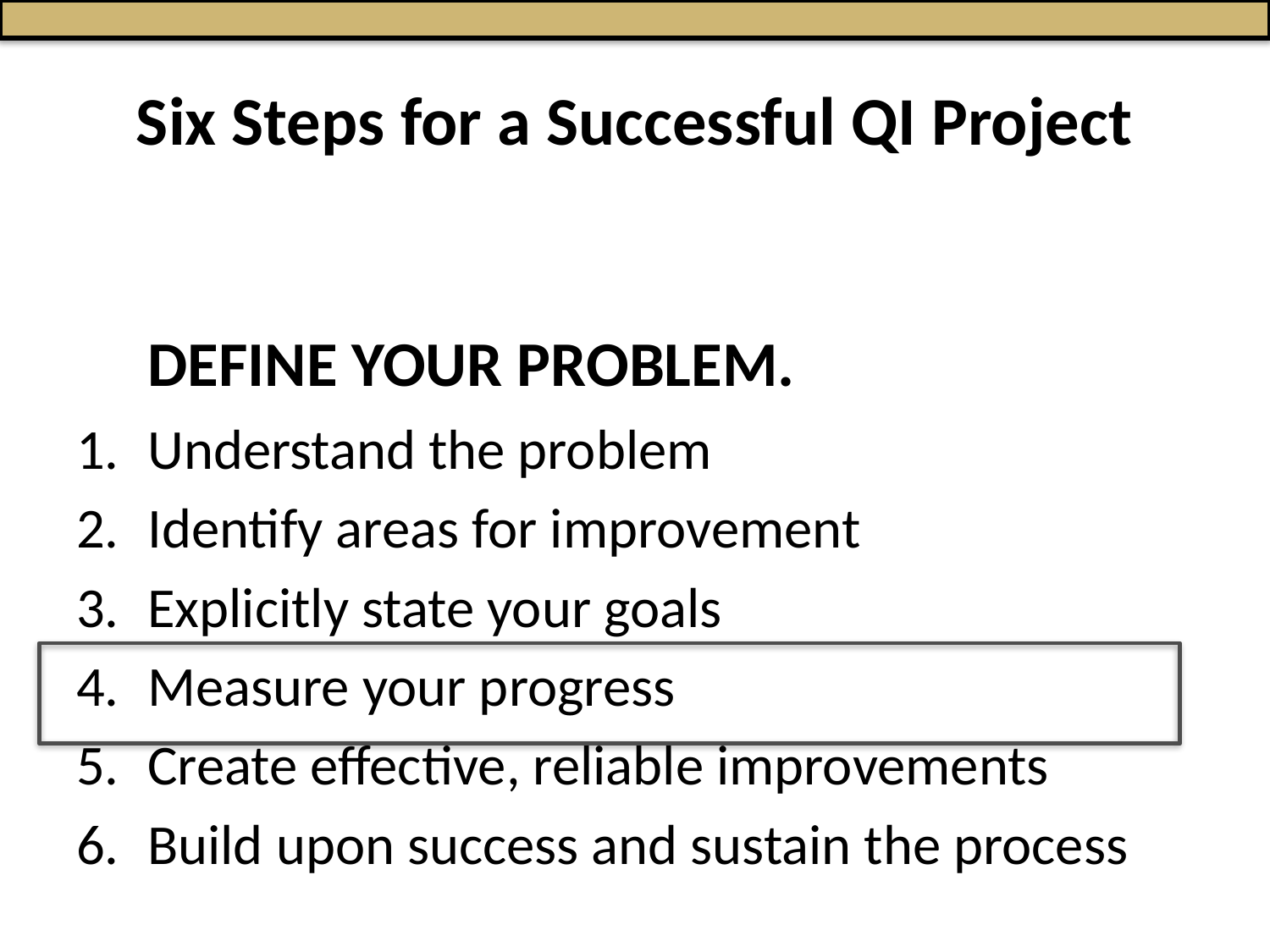

# Six Steps for a Successful QI Project
DEFINE YOUR PROBLEM.
Understand the problem
Identify areas for improvement
Explicitly state your goals
Measure your progress
Create effective, reliable improvements
Build upon success and sustain the process

## Slide 49
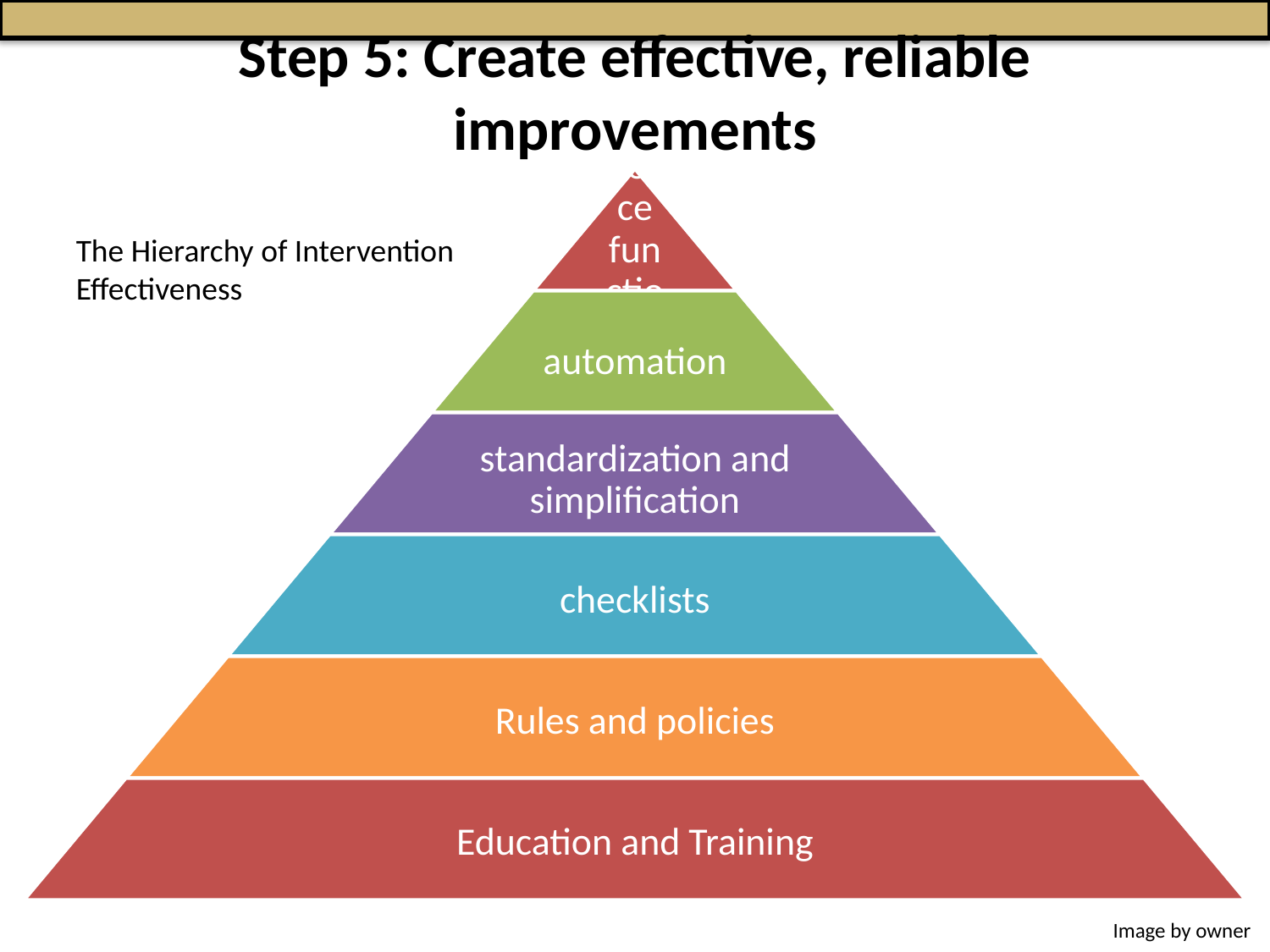

# Step 5: Create effective, reliable improvements
The Hierarchy of Intervention Effectiveness
Image by owner

## Slide 50
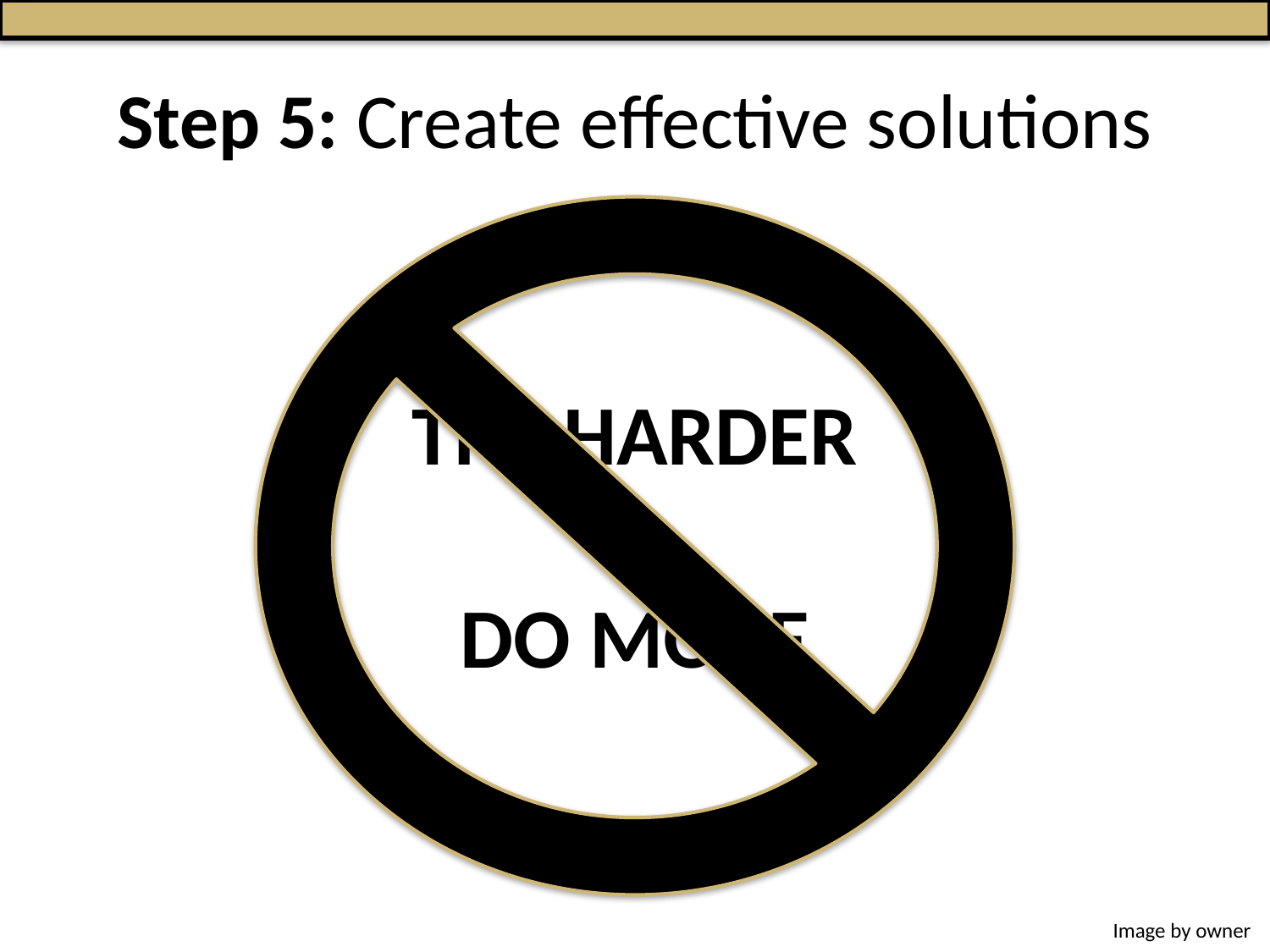

# Step 5: Create effective solutions
TRY HARDER
DO MORE
Image by owner

## Slide 51
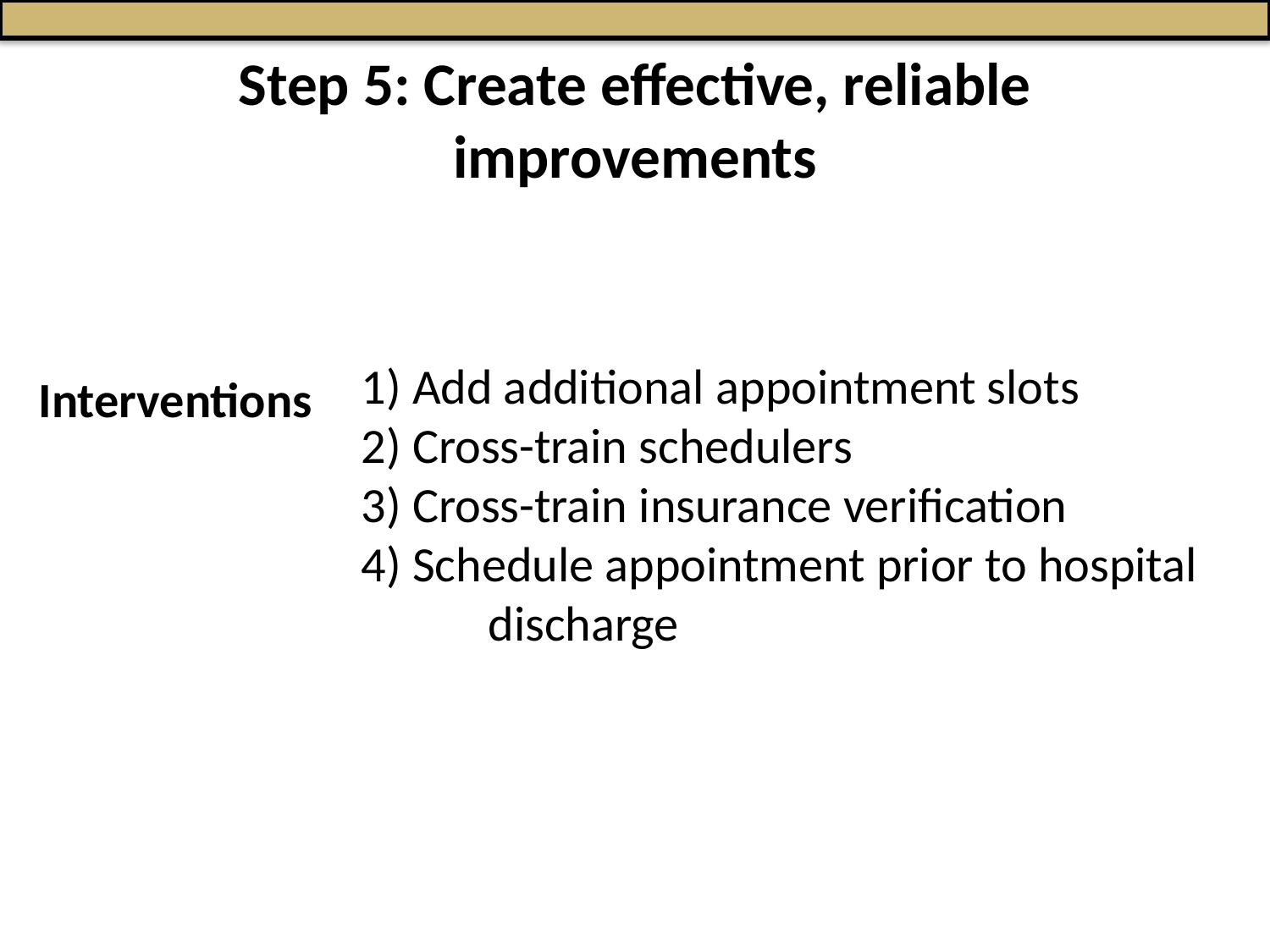

# Step 5: Create effective, reliable improvements
Interventions
1) Add additional appointment slots
2) Cross-train schedulers
3) Cross-train insurance verification
4) Schedule appointment prior to hospital 	discharge

## Slide 52
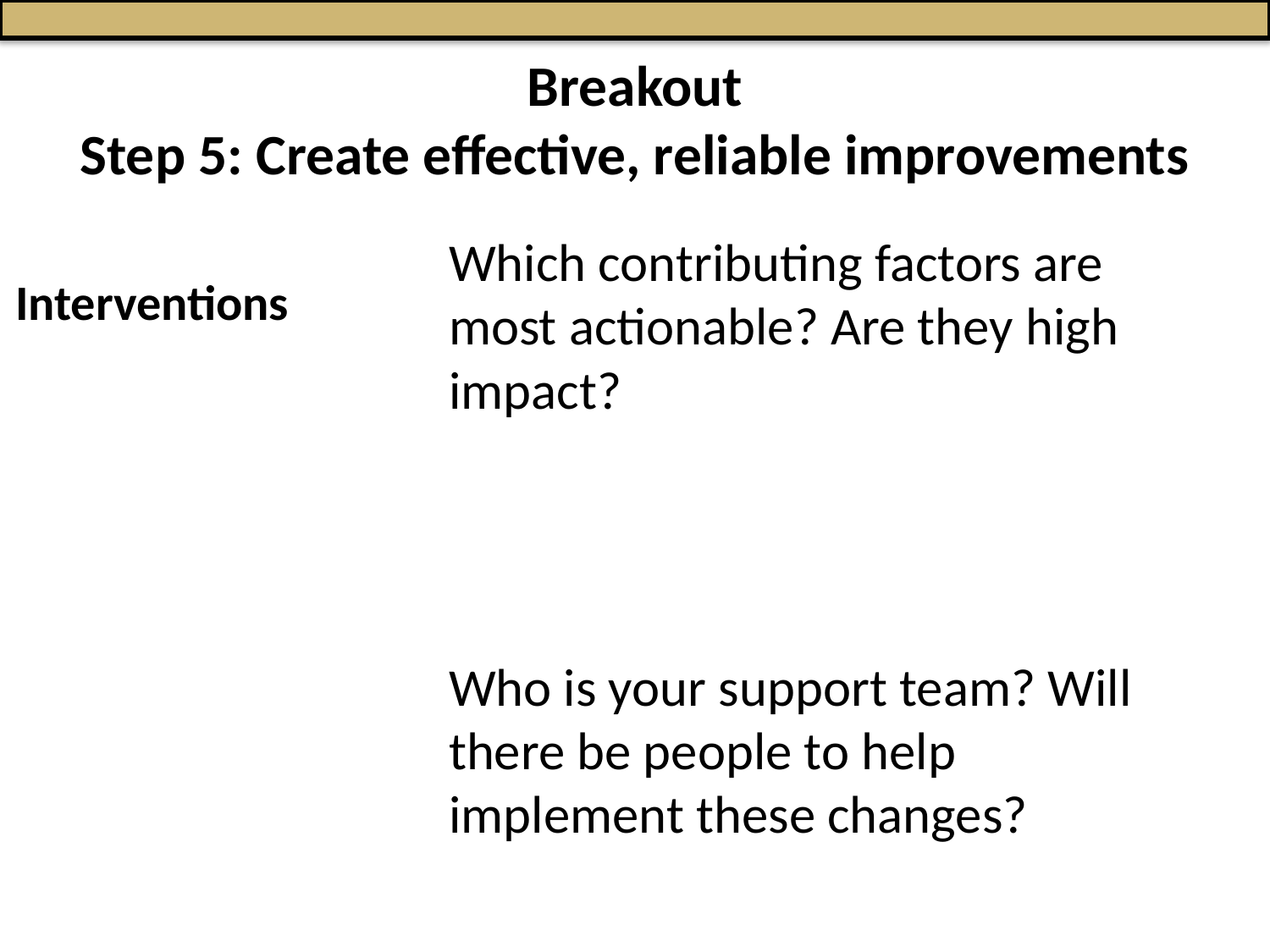

# Breakout Step 5: Create effective, reliable improvements
Interventions
Which contributing factors are most actionable? Are they high impact?
Who is your support team? Will there be people to help implement these changes?

## Slide 53
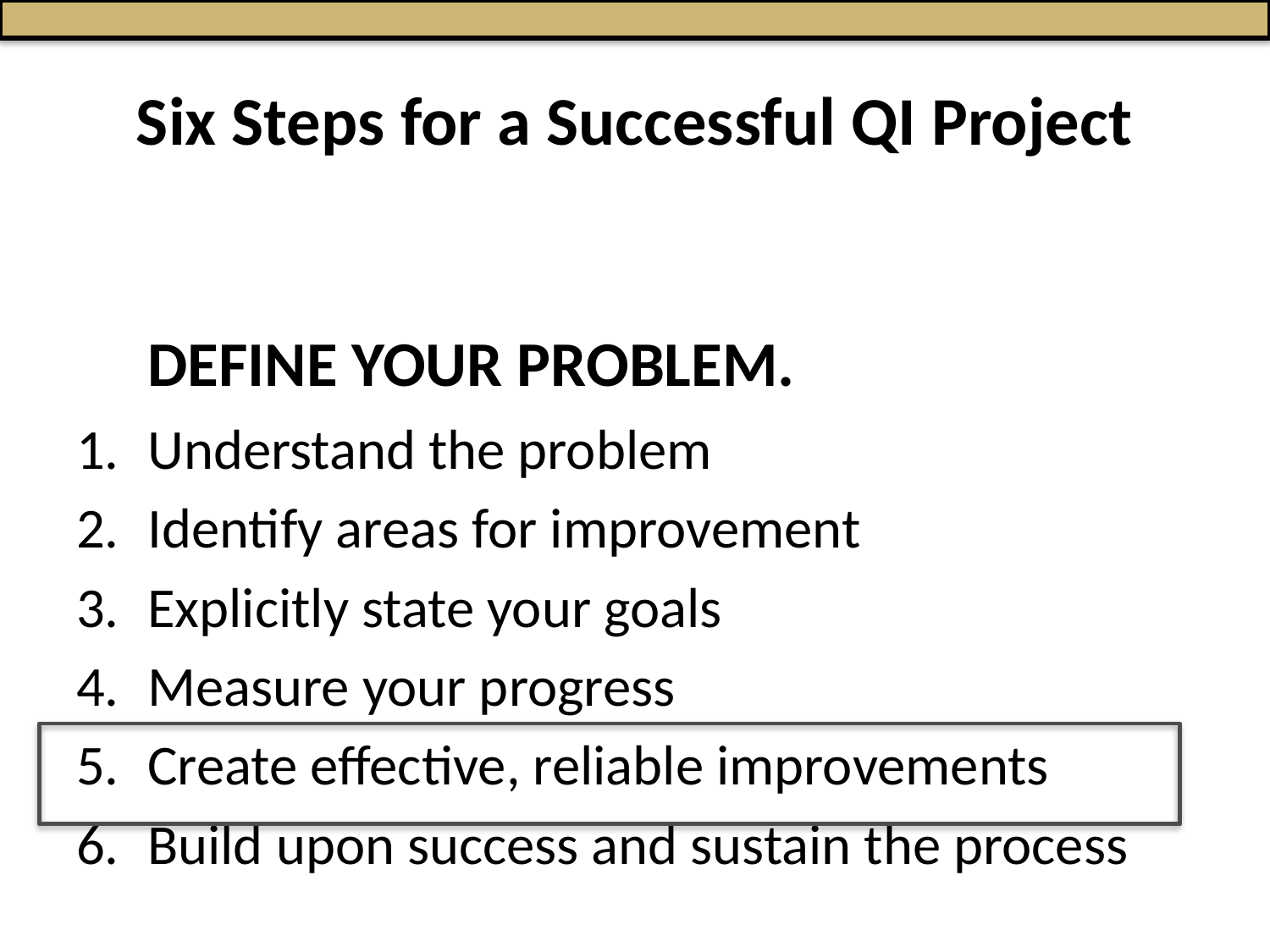

# Six Steps for a Successful QI Project
DEFINE YOUR PROBLEM.
Understand the problem
Identify areas for improvement
Explicitly state your goals
Measure your progress
Create effective, reliable improvements
Build upon success and sustain the process

## Slide 54
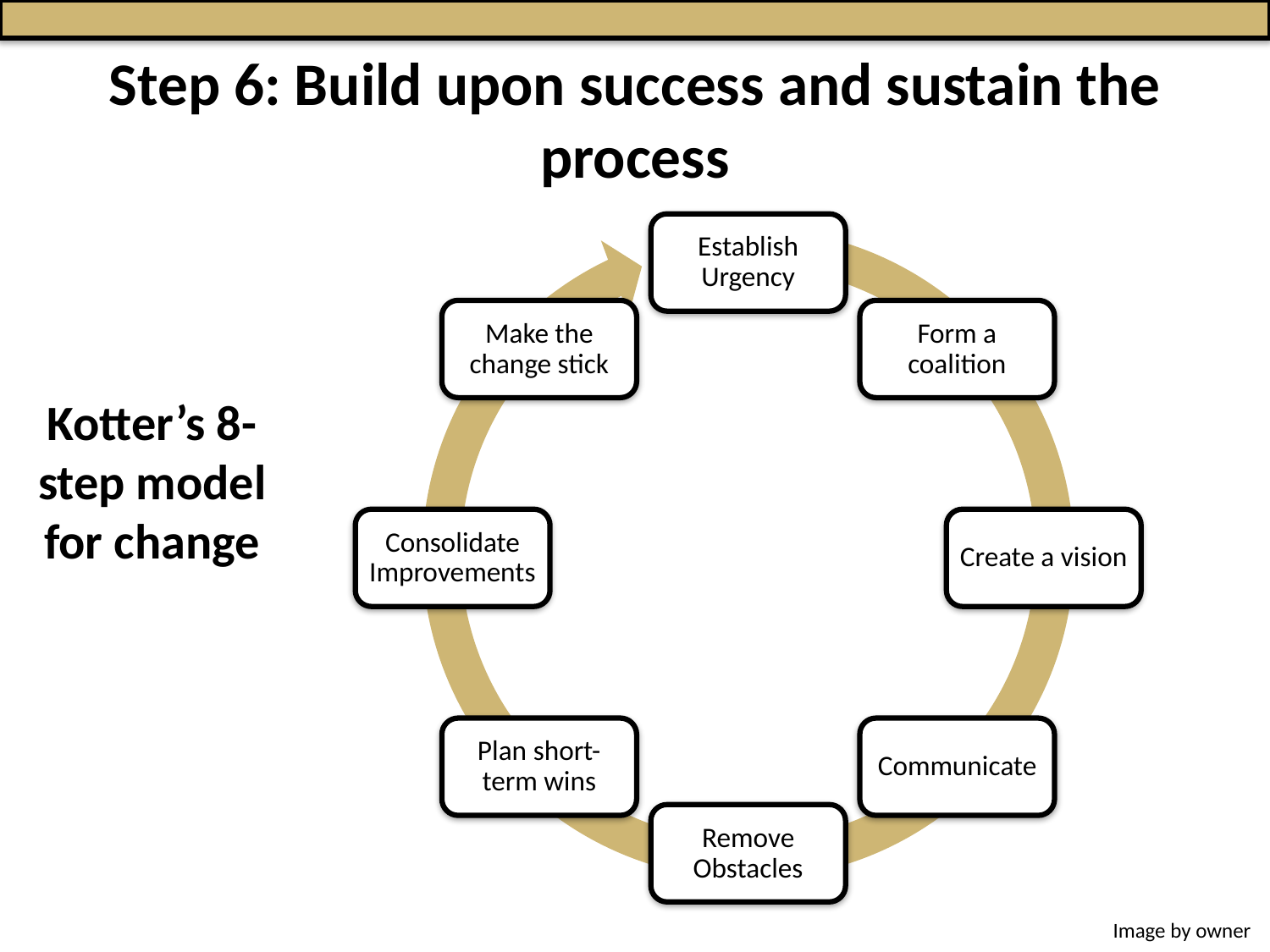

# Step 6: Build upon success and sustain the process
Establish Urgency
Make the change stick
Form a coalition
Consolidate Improvements
Create a vision
Plan short-term wins
Communicate
Remove Obstacles
Kotter’s 8-step model for change
Image by owner

## Slide 55
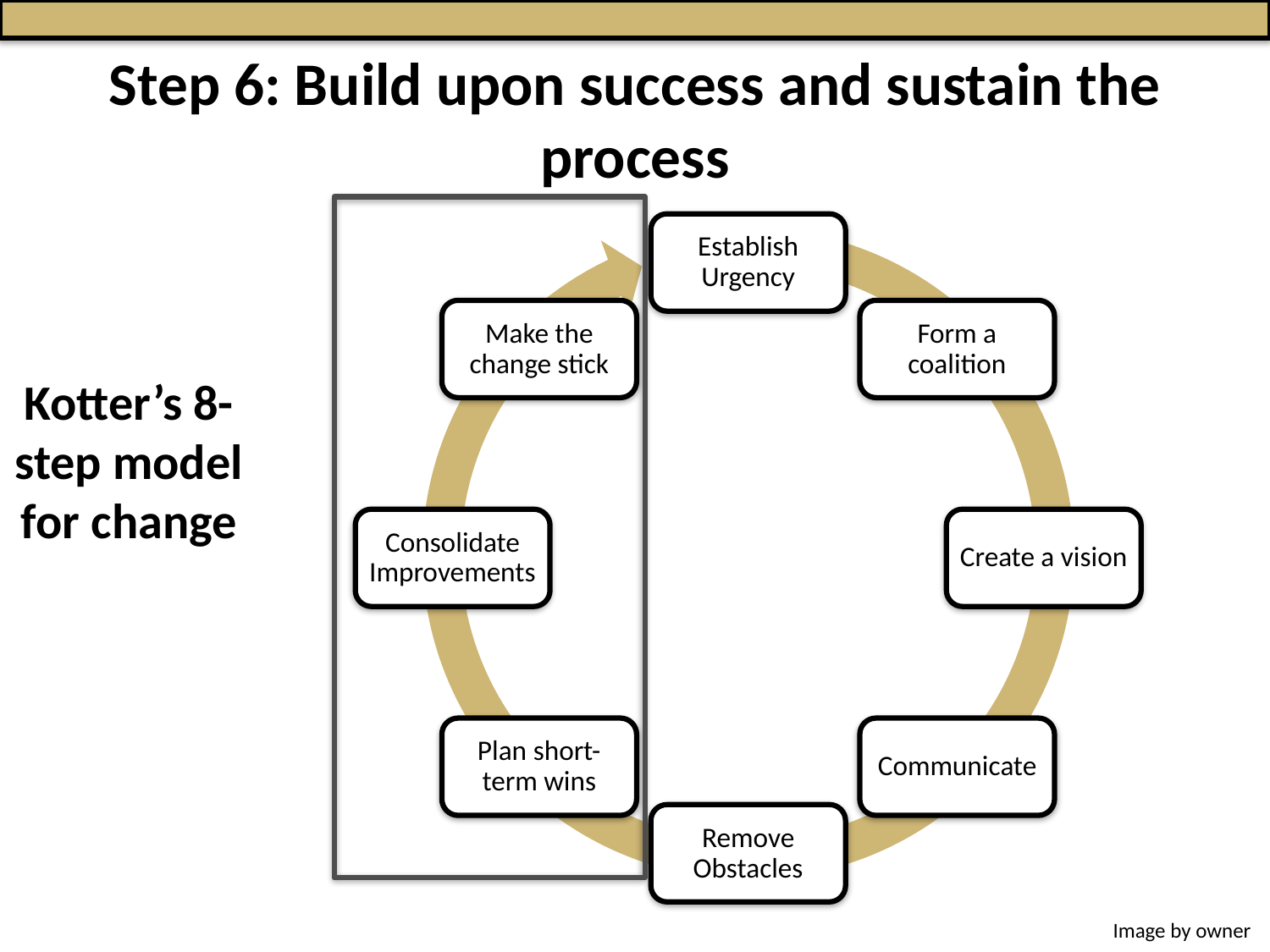

# Step 6: Build upon success and sustain the process
Establish Urgency
Make the change stick
Form a coalition
Consolidate Improvements
Create a vision
Plan short-term wins
Communicate
Remove Obstacles
Kotter’s 8-step model for change
Image by owner

## Slide 56
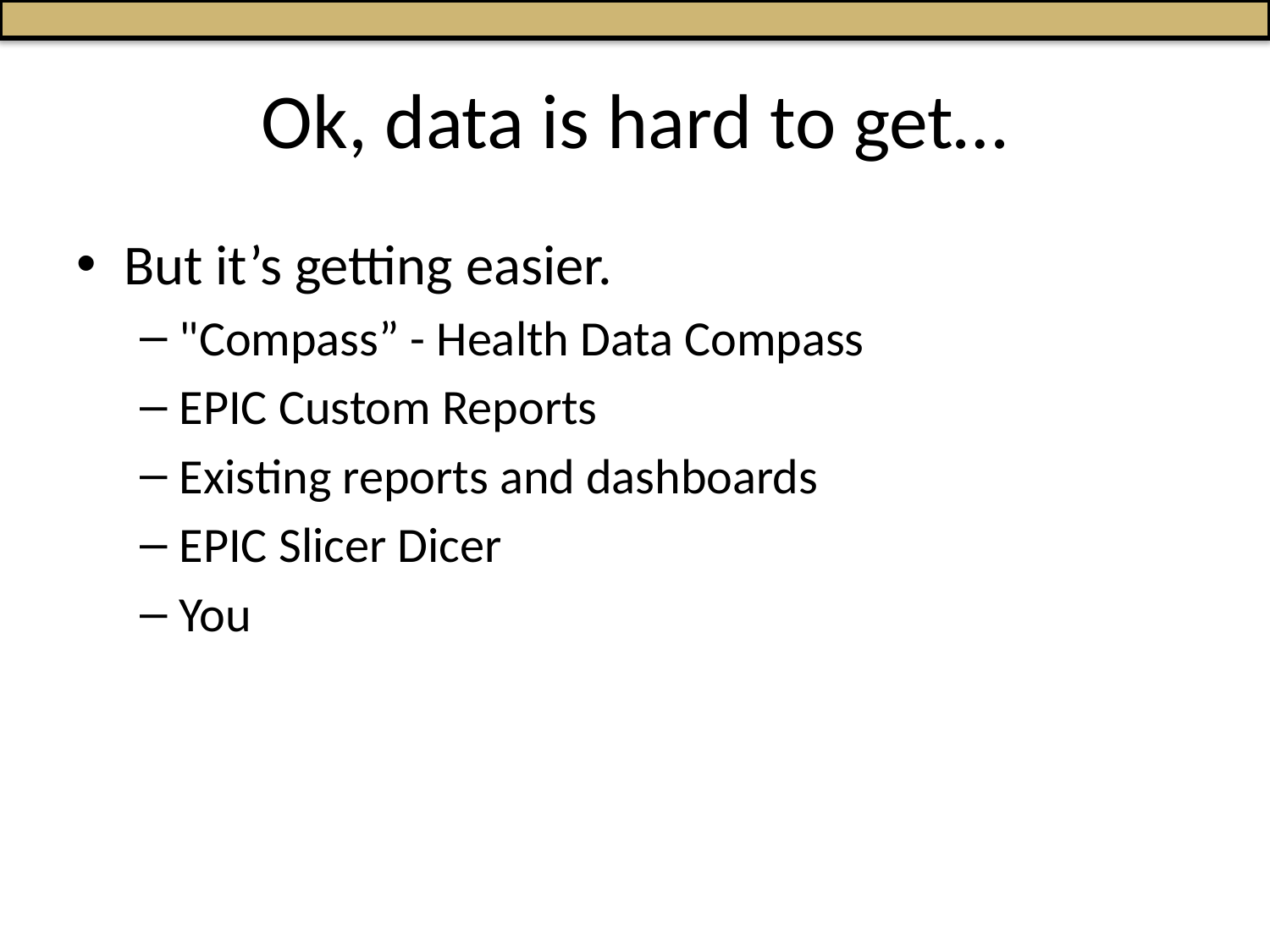

# Ok, data is hard to get…
But it’s getting easier.
"Compass” - Health Data Compass
EPIC Custom Reports
Existing reports and dashboards
EPIC Slicer Dicer
You

## Slide 57
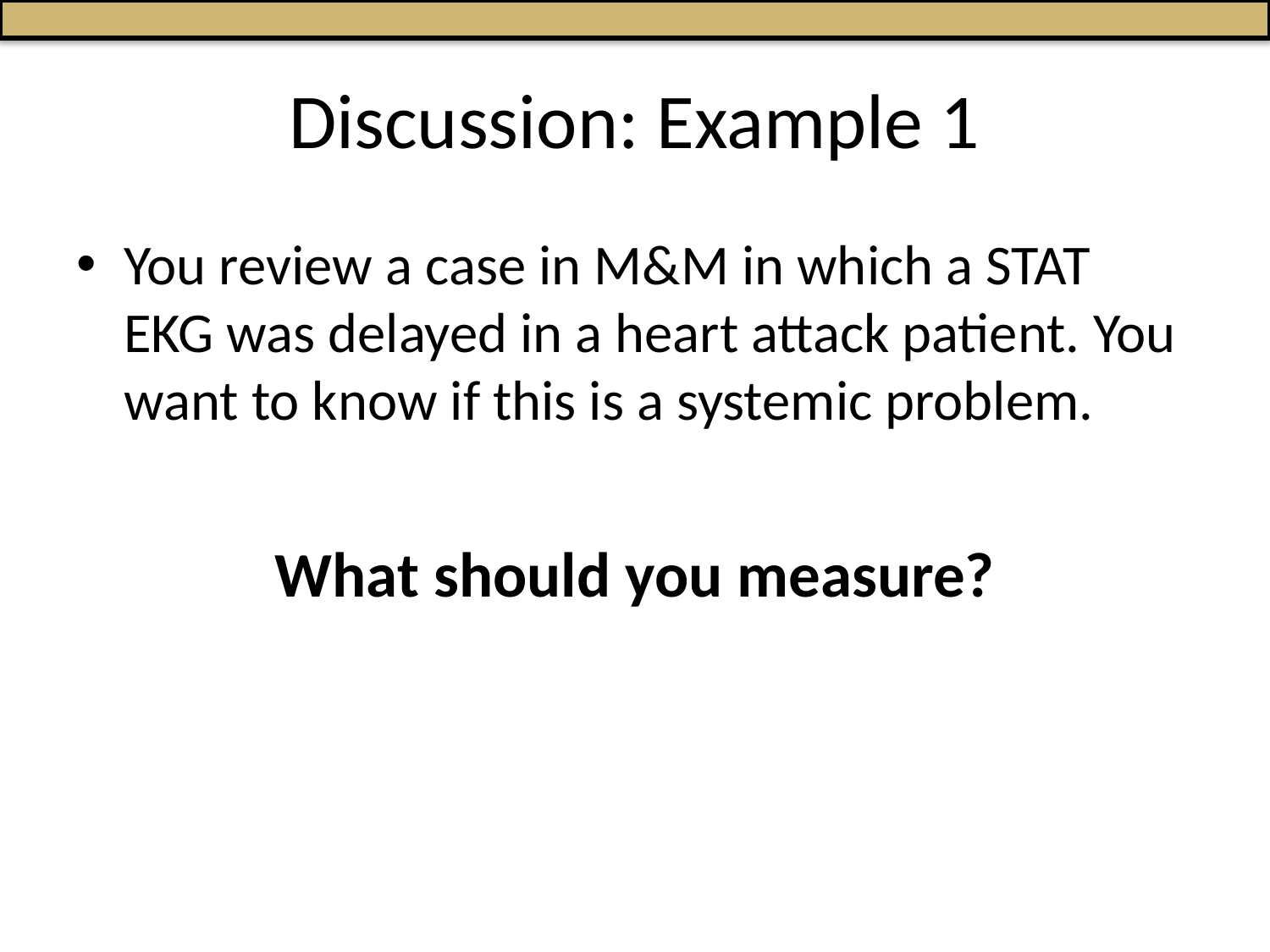

# Discussion: Example 1
You review a case in M&M in which a STAT EKG was delayed in a heart attack patient. You want to know if this is a systemic problem.
What should you measure?

## Slide 58
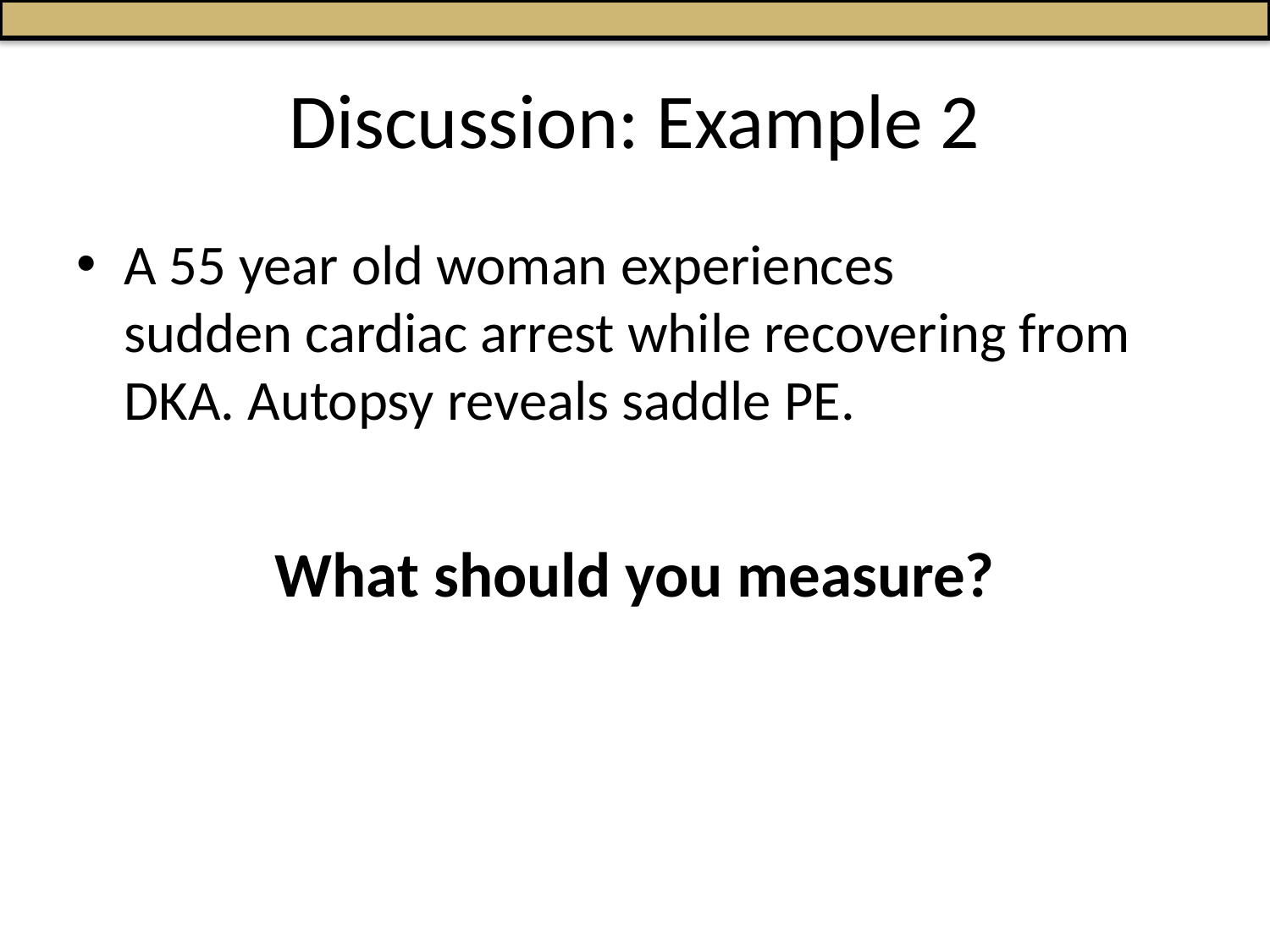

# Discussion: Example 2
A 55 year old woman experiences sudden cardiac arrest while recovering from DKA. Autopsy reveals saddle PE.
What should you measure?

## Slide 59
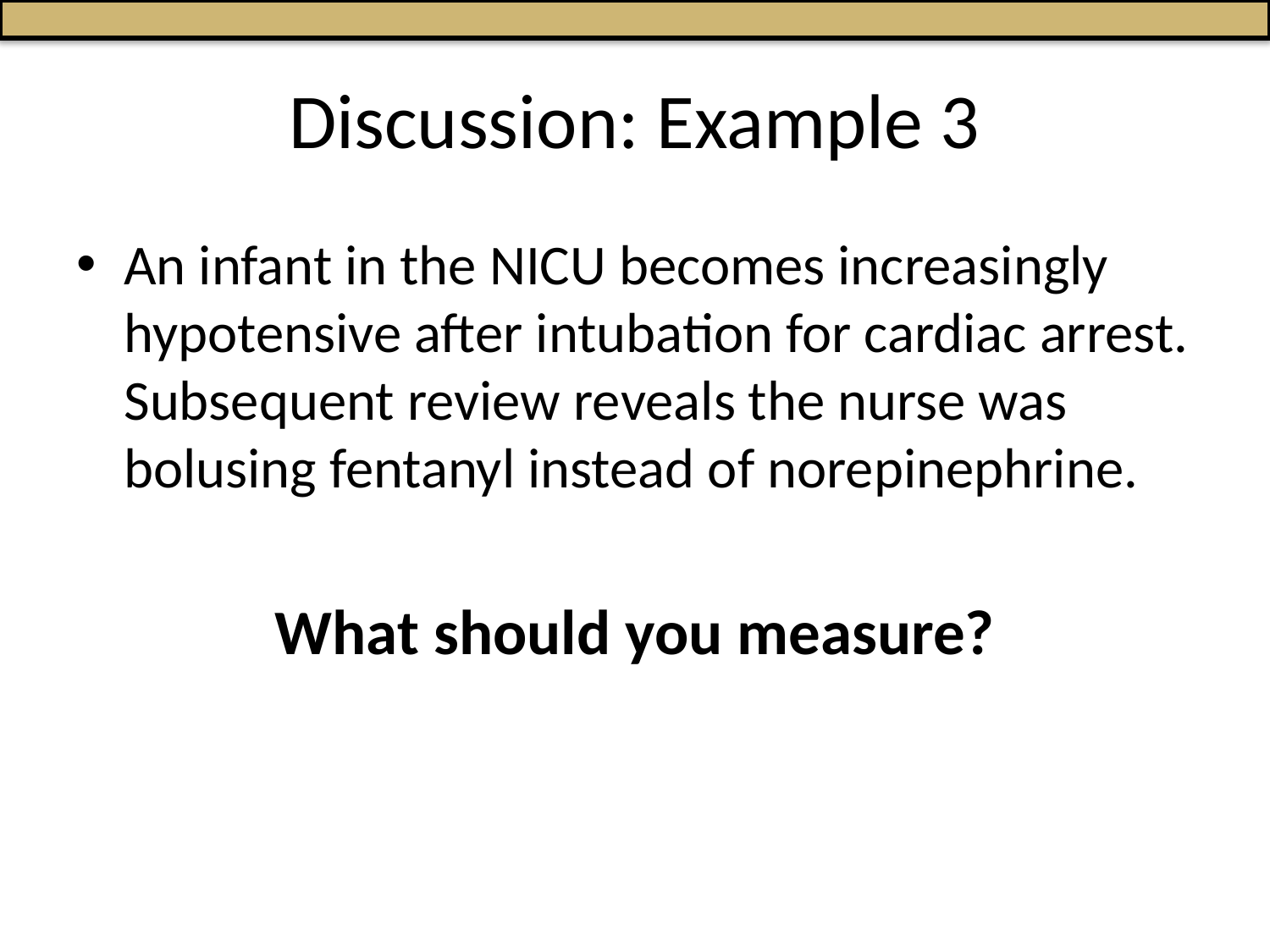

# Discussion: Example 3
An infant in the NICU becomes increasingly hypotensive after intubation for cardiac arrest. Subsequent review reveals the nurse was bolusing fentanyl instead of norepinephrine.
What should you measure?

## Slide 60
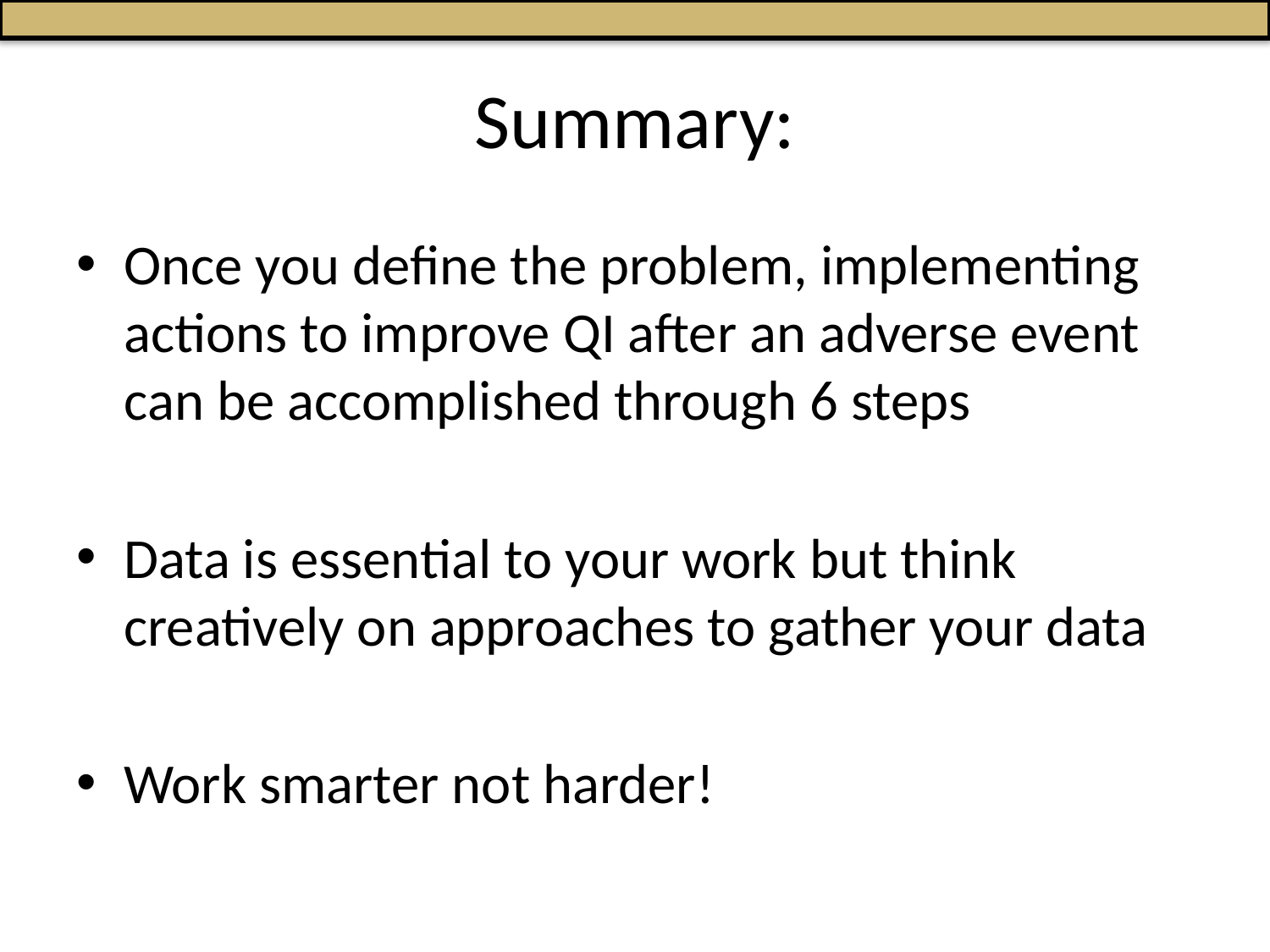

# Summary:
Once you define the problem, implementing actions to improve QI after an adverse event can be accomplished through 6 steps
Data is essential to your work but think creatively on approaches to gather your data
Work smarter not harder!

## Slide 61
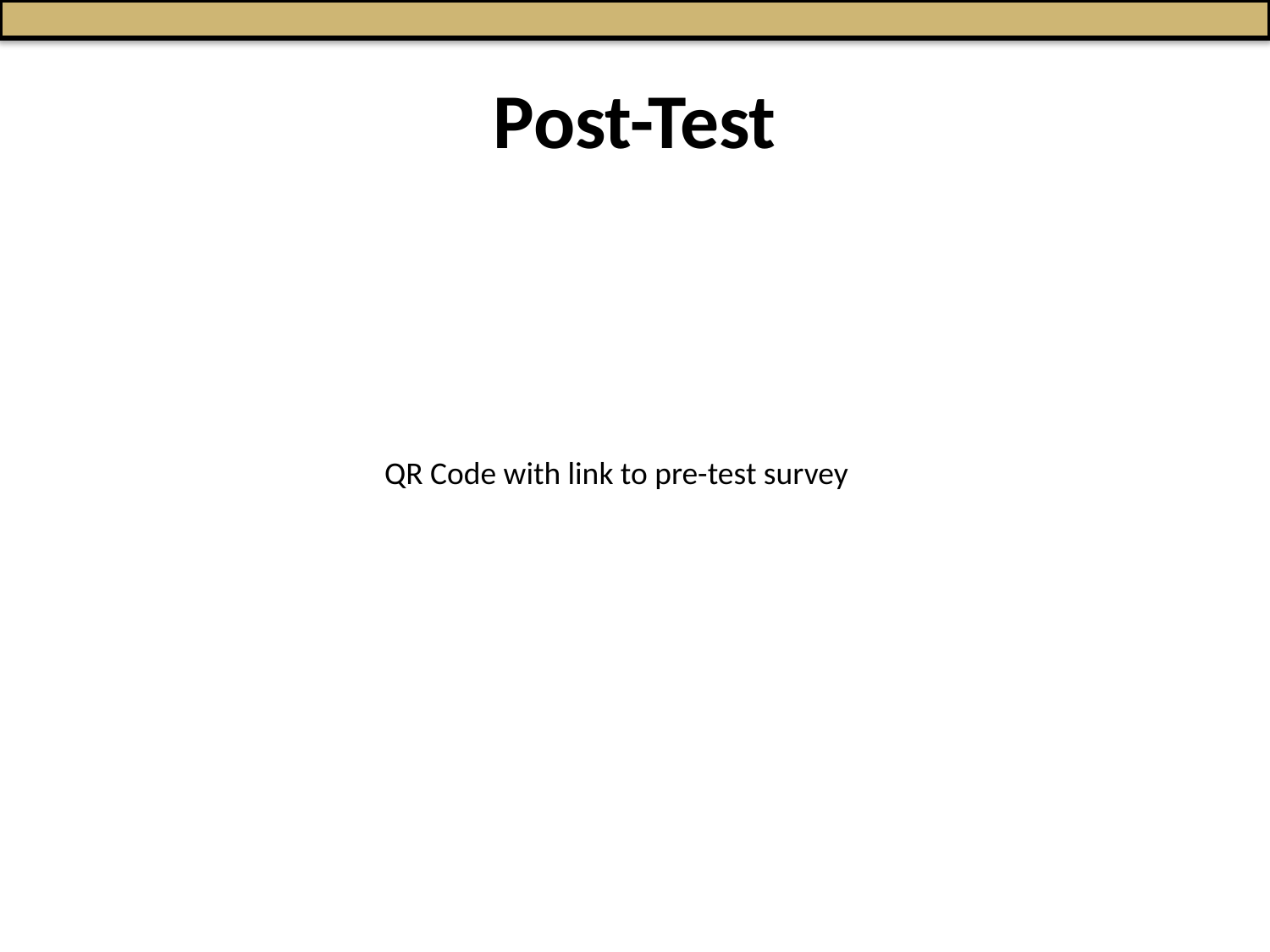

# Post-Test
QR Code with link to pre-test survey
