# Supplementary material for: Expanding Training in Quality Improvement and Patient Safety Through a Multispecialty Graduate Medical Education Curriculum Designed for Fellows
Source: MedEdPORTAL. 2020 Dec 30;16:11064. doi: 10.15766/mep_2374-8265.11064 (PMC7780740; doi:10.15766/mep_2374-8265.11064)
Supplement: Supplementary file 1 — Foundations in Patient Safety Teaching Slides.pptxFoundations in Patient Safety Playbook and Small-Group Activities.docxAdverse Events Into QI Teaching Slides.pptxAdverse Events Into QI Playbook and Small-Group Activities.docxQuality in Academics Teaching Slides.pptxQuality in Academics Playbook and Small-Group Activities.docxFoundations in Patient Safety Assessment Survey.docxAdverse Events Into QI Assessment Survey.docxQuality in Academics Assessment Survey.docx [file mep_2374-8265.11064-s001.zip › D. Adverse Events Into QI Playbook and Small-Group Activities.docx]

# Quality & Safety Academy

# QI Tools, Tips, and Tricks Playbook

## Step 0: Define the Problem

**Please provide a one-liner of the problem you identified in your clinical environment.**

How do you know this is a problem?

*Who is impacted?*

*What is the scale?*

*What other data do you have to support this?*

## Step 1: Understand the Problem

Create a Process Map

*If unable with current knowledge of the problem, create what you THINK is the current process. BE AS SPECFIC AS YOU CAN!*

Create a Cause & Effect Analysis (Fishbone Diagram) THEN – Use “5-why’s” to further understand one contributing factor.

**
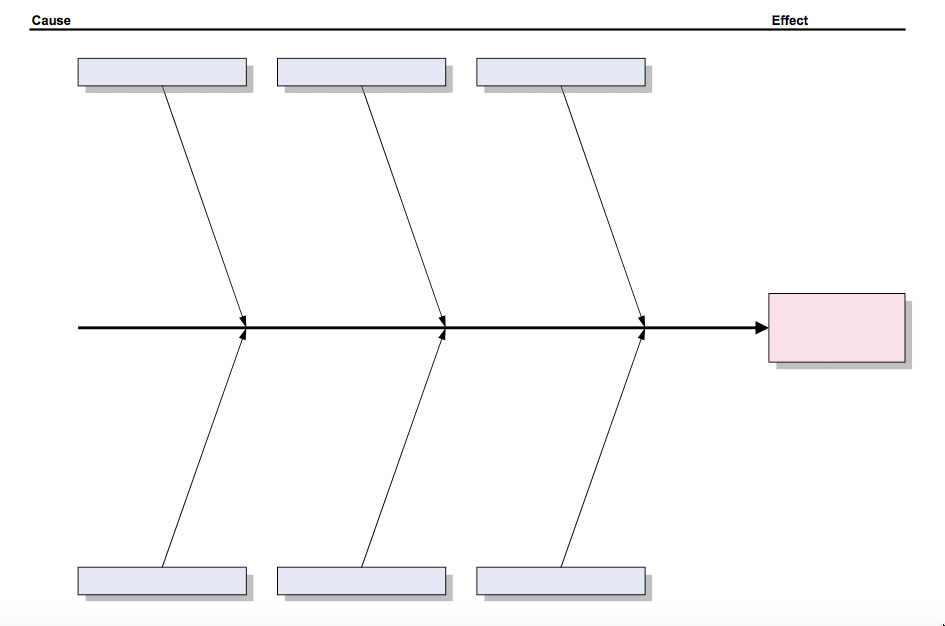
**

## Step 2: Identify Areas for Improvement

**Quick Wins**

**Major Projects**

**Thankless Tasks**

**Fill-in Jobs**

**Impact**

**Effort**

## Step 3: State your Goals

What is your AIM statement? Make it SMART (“How much, of what, by when?”).

***S****pecific*

***M****easurable*

***A****chievable*

***R****elevant*

***T****imely*

## Step 4: Measure your Progress

What are you measuring? List potential measures below in each category.

***Process measures*** *(the steps of doing the work—e.g., what % of patients get a lactate drawn when we think they have sepsis):*

***Outcome measures*** *(the impact on the patient/population of interest—e.g., how many patients die each year from sepsis?):*

***Balancing measures*** *(negative side effects we hope not to cause, and thus should watch for—e.g., % patients who get aggressive fluids for presumed sepsis, and then develop pulmonary edema and end up on the ventilator):*

What are your data sources?

## Step 5: Create Impactful, Sustainable Solutions

Which contributing factors are most actionable? Are they high impact?

Who is your support team? Will there be people to help implement these changes?

## Step 6: Build Upon Success & Sustain

Plan for quick wins:

*What will you celebrate?*

*How will you celebrate?*

*How will you show that your project makes lives better? What’s the return-on-investment?*

*How will you ensure leaders are aware of your success, and can take (some) credit for it?*
