# Supplementary material for: Expanding Training in Quality Improvement and Patient Safety Through a Multispecialty Graduate Medical Education Curriculum Designed for Fellows
Source: MedEdPORTAL. 2020 Dec 30;16:11064. doi: 10.15766/mep_2374-8265.11064 (PMC7780740; doi:10.15766/mep_2374-8265.11064)
Supplement: Supplementary file 1 — Foundations in Patient Safety Teaching Slides.pptxFoundations in Patient Safety Playbook and Small-Group Activities.docxAdverse Events Into QI Teaching Slides.pptxAdverse Events Into QI Playbook and Small-Group Activities.docxQuality in Academics Teaching Slides.pptxQuality in Academics Playbook and Small-Group Activities.docxFoundations in Patient Safety Assessment Survey.docxAdverse Events Into QI Assessment Survey.docxQuality in Academics Assessment Survey.docx [file mep_2374-8265.11064-s001.zip › E. Quality in Academics Teaching Slides.pptx]

## Slide 1
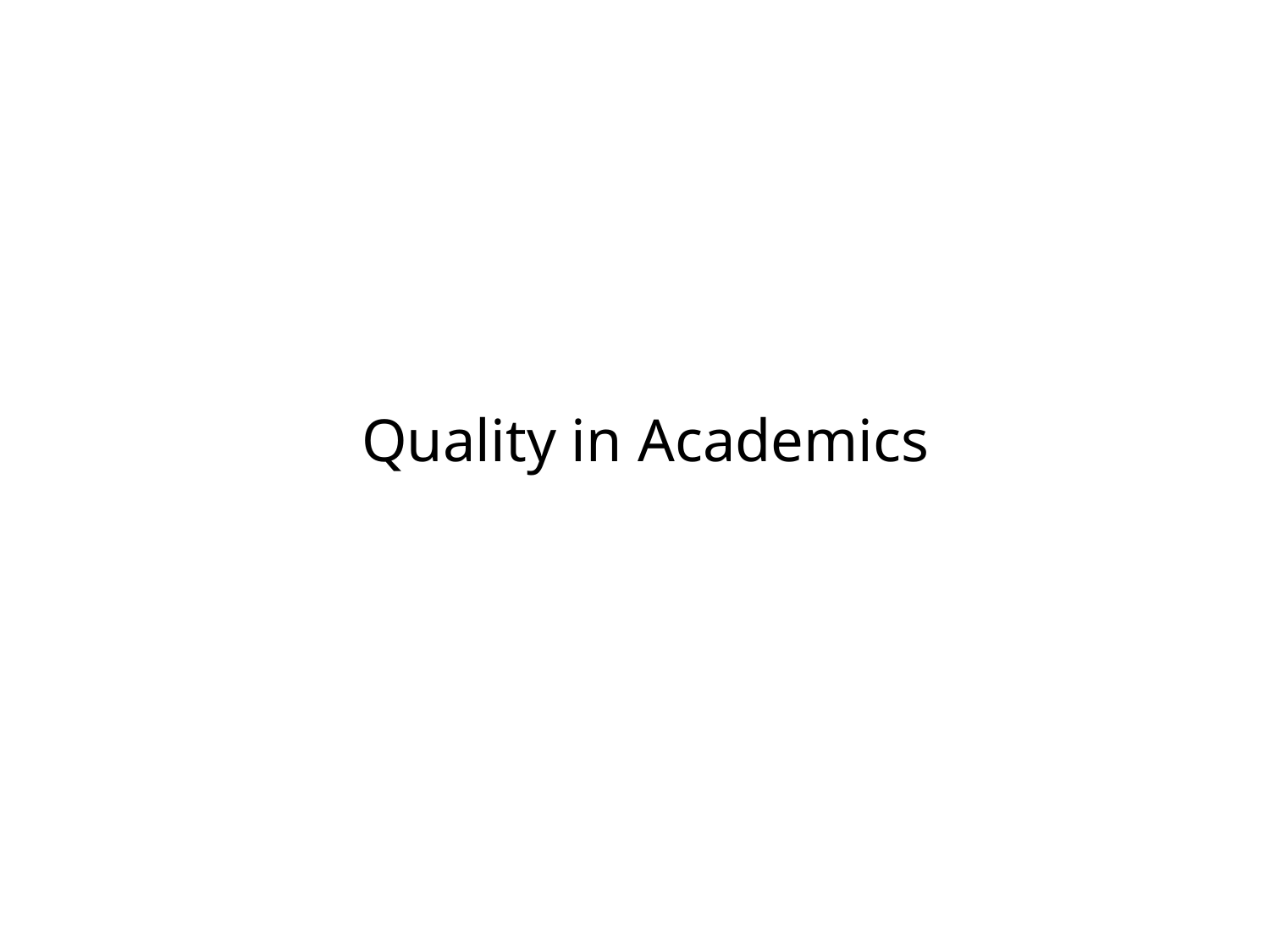

Quality in Academics

## Slide 2
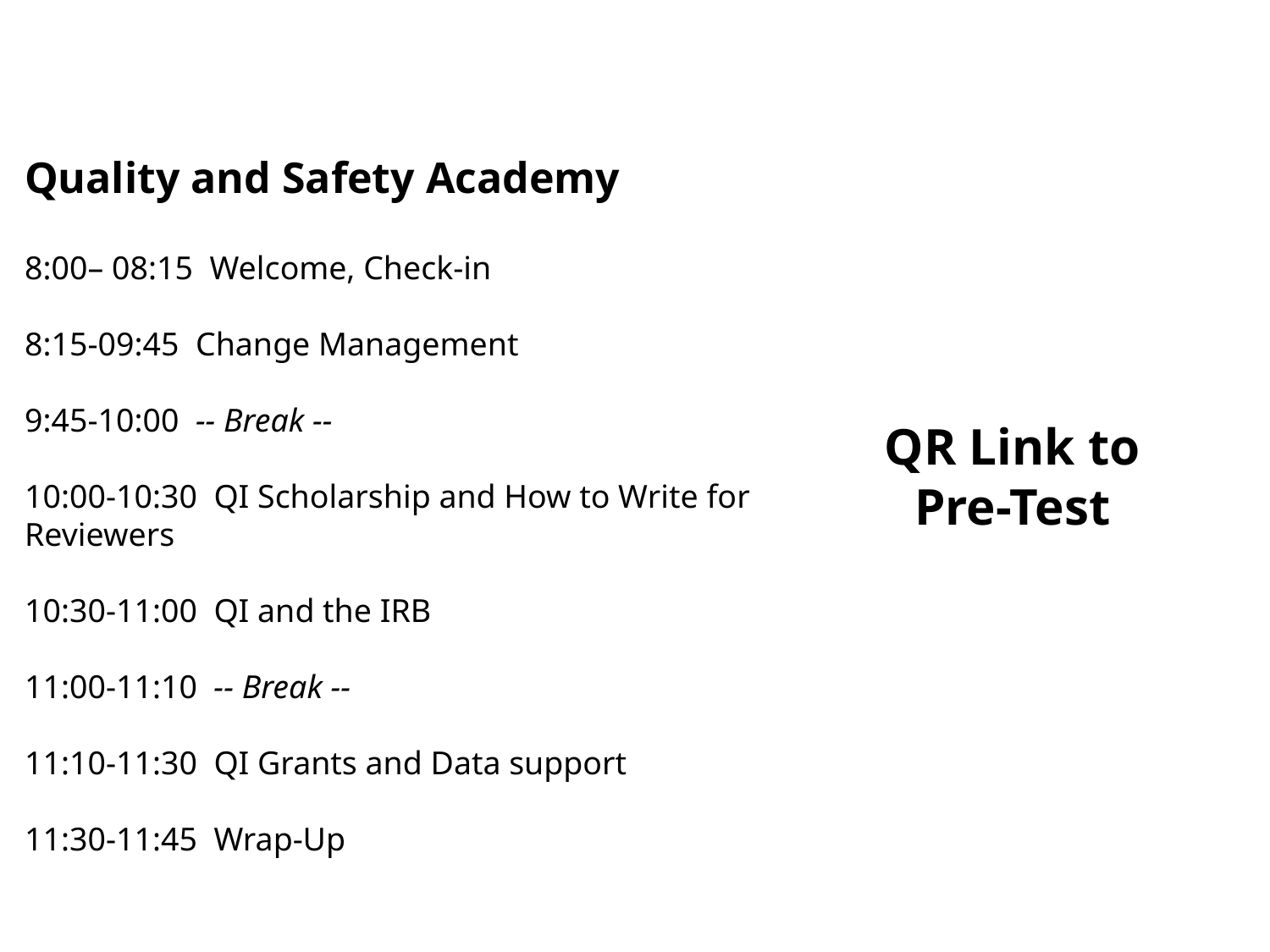

Quality and Safety Academy
8:00– 08:15  Welcome, Check-in
8:15-09:45  Change Management
9:45-10:00  -- Break --
10:00-10:30  QI Scholarship and How to Write for Reviewers
10:30-11:00  QI and the IRB
11:00-11:10  -- Break --
11:10-11:30  QI Grants and Data support
11:30-11:45 Wrap-Up
QR Link to Pre-Test

## Slide 3
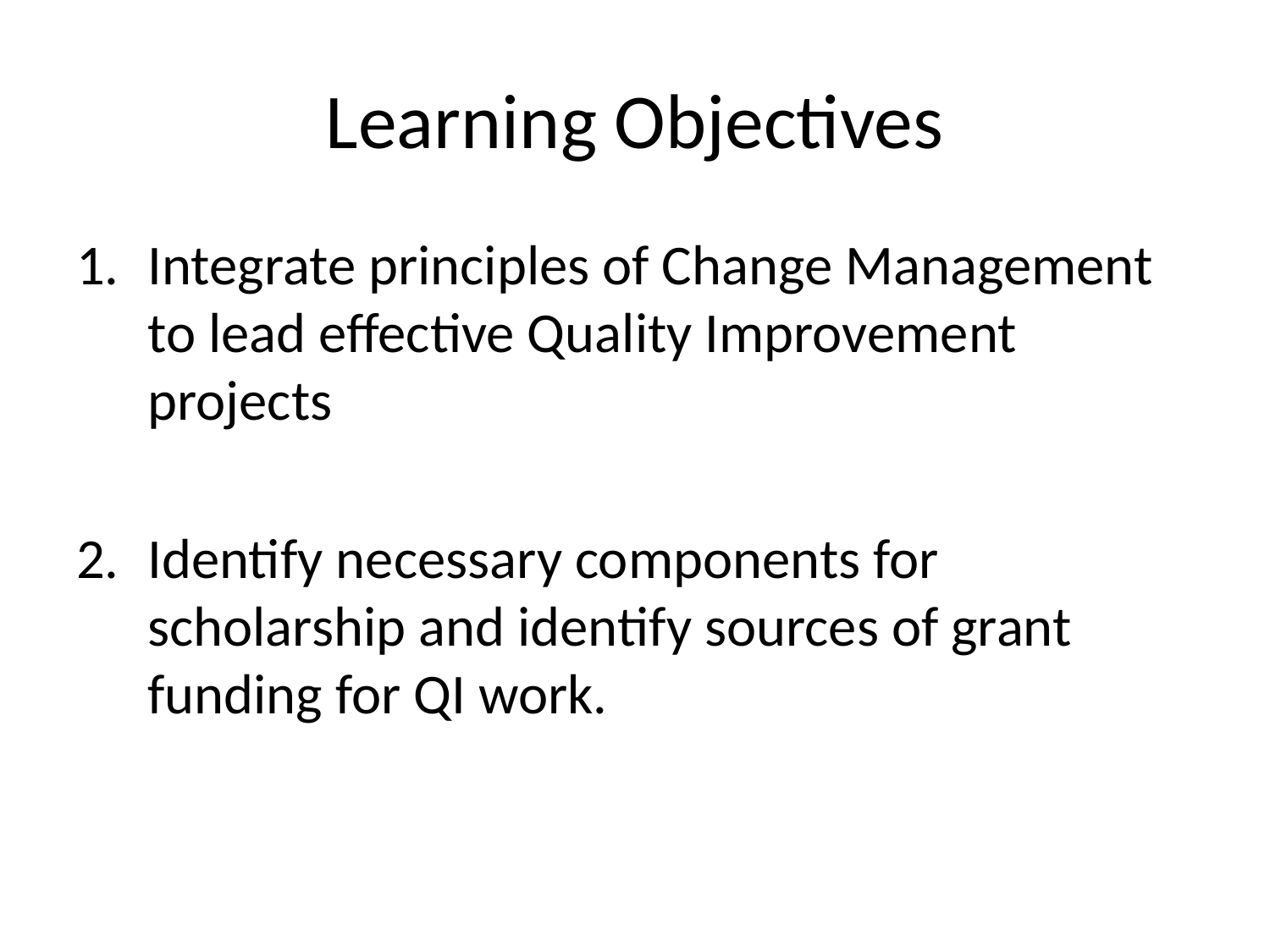

# Learning Objectives
Integrate principles of Change Management to lead effective Quality Improvement projects
Identify necessary components for scholarship and identify sources of grant funding for QI work.

## Slide 4
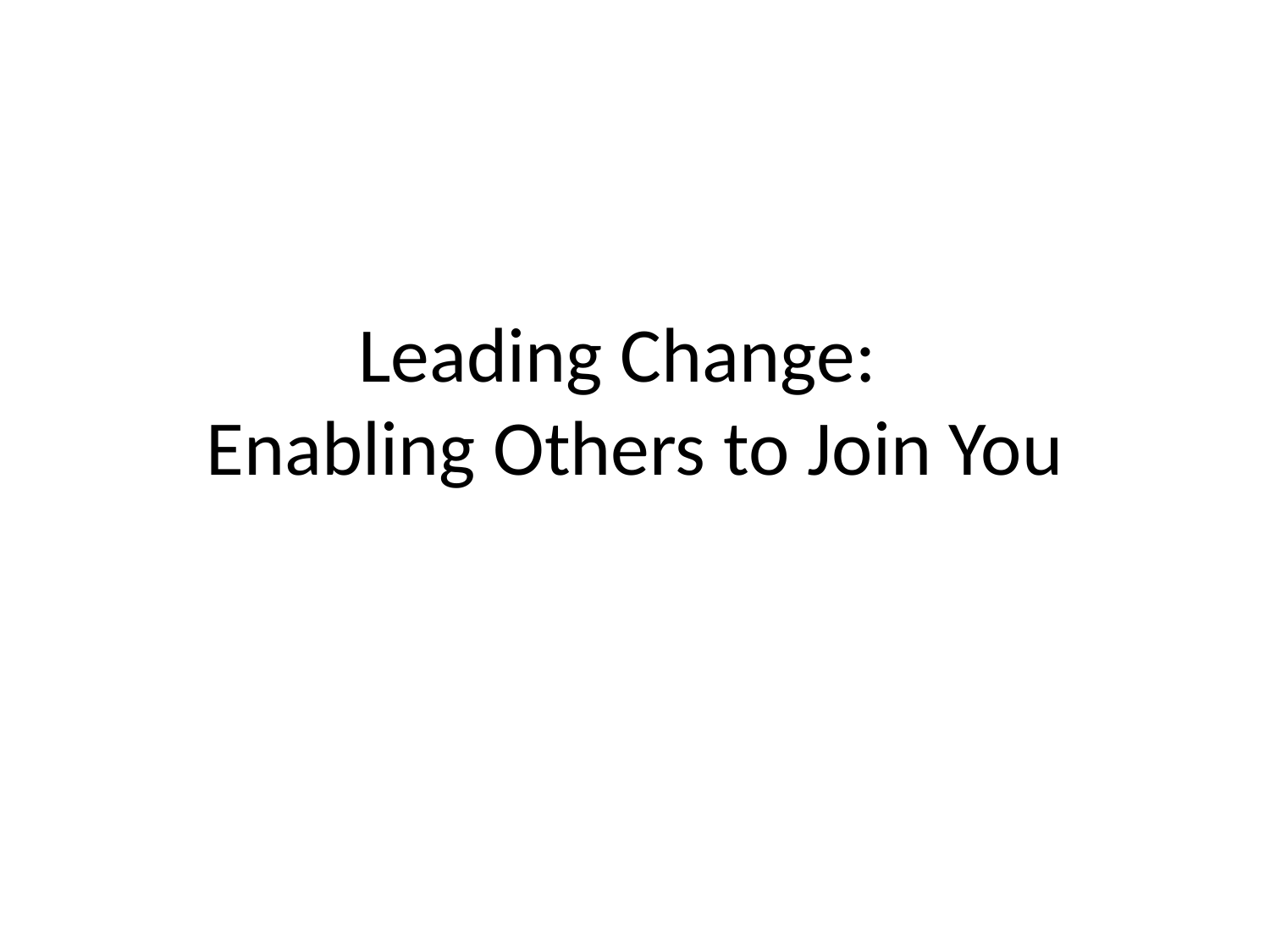

# Leading Change: Enabling Others to Join You

## Slide 5
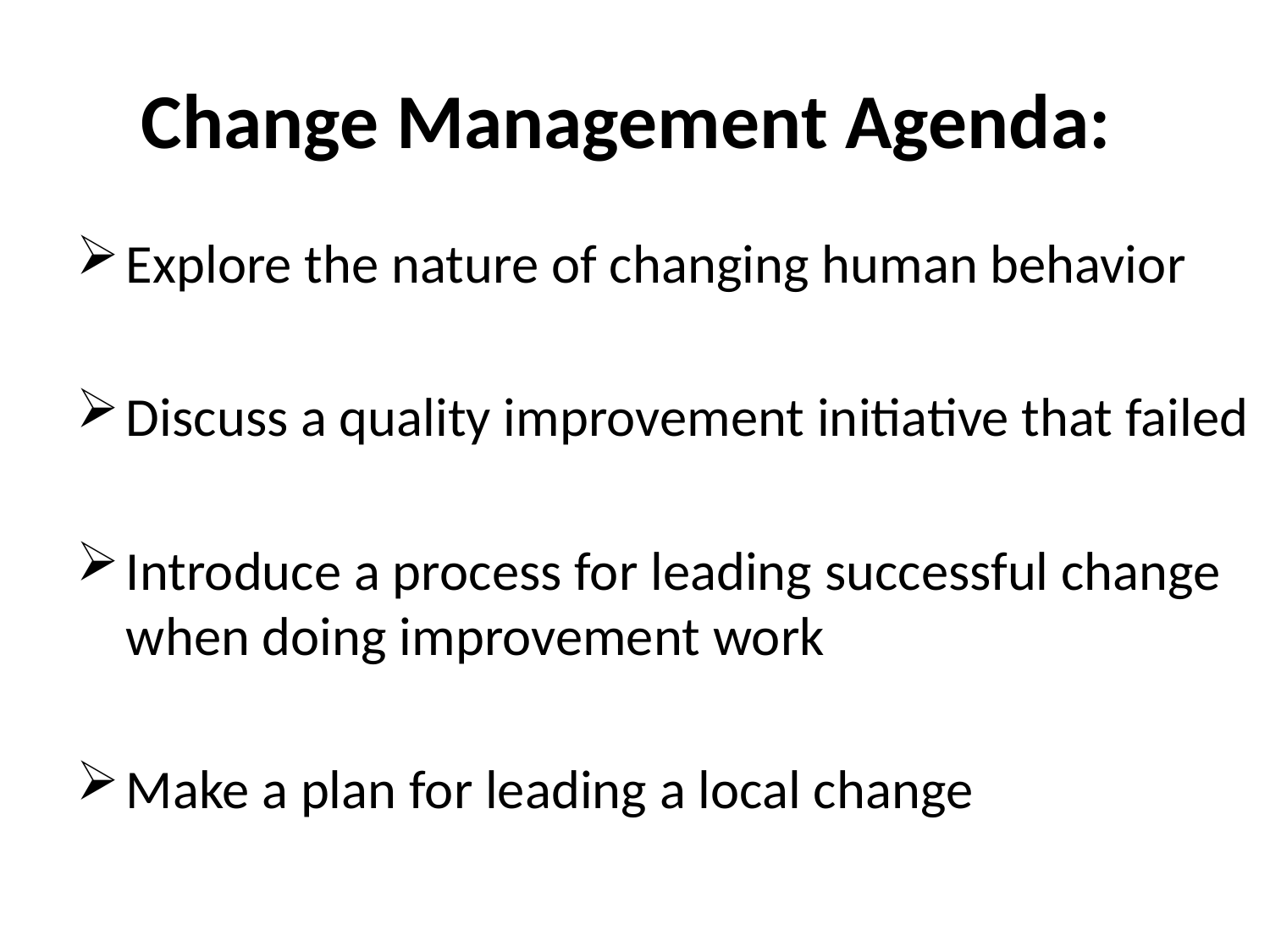

# Change Management Agenda:
Explore the nature of changing human behavior
Discuss a quality improvement initiative that failed
Introduce a process for leading successful change when doing improvement work
Make a plan for leading a local change

## Slide 6
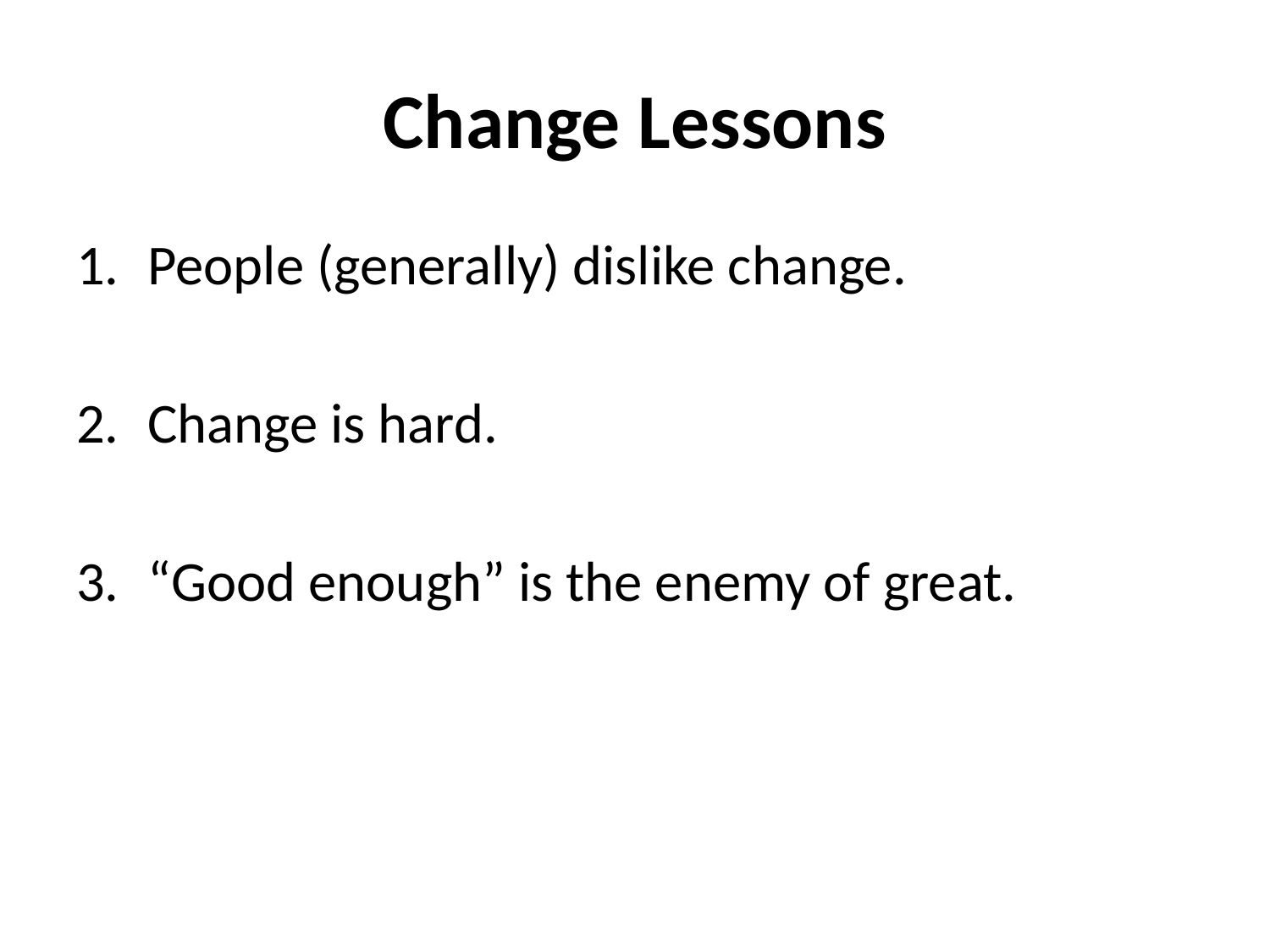

# Change Lessons
People (generally) dislike change.
Change is hard.
“Good enough” is the enemy of great.

## Slide 7
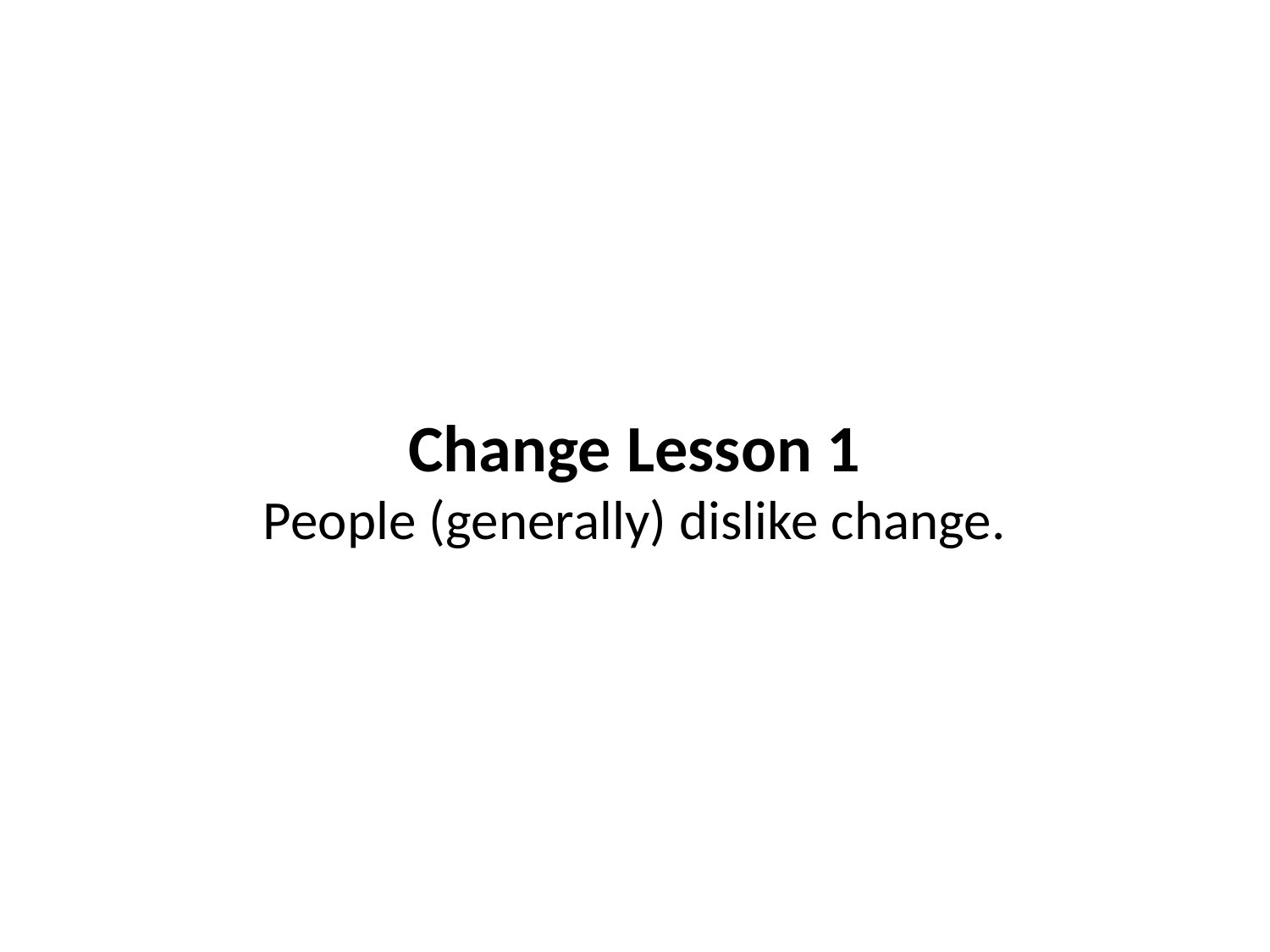

# Change Lesson 1 People (generally) dislike change.

## Slide 8
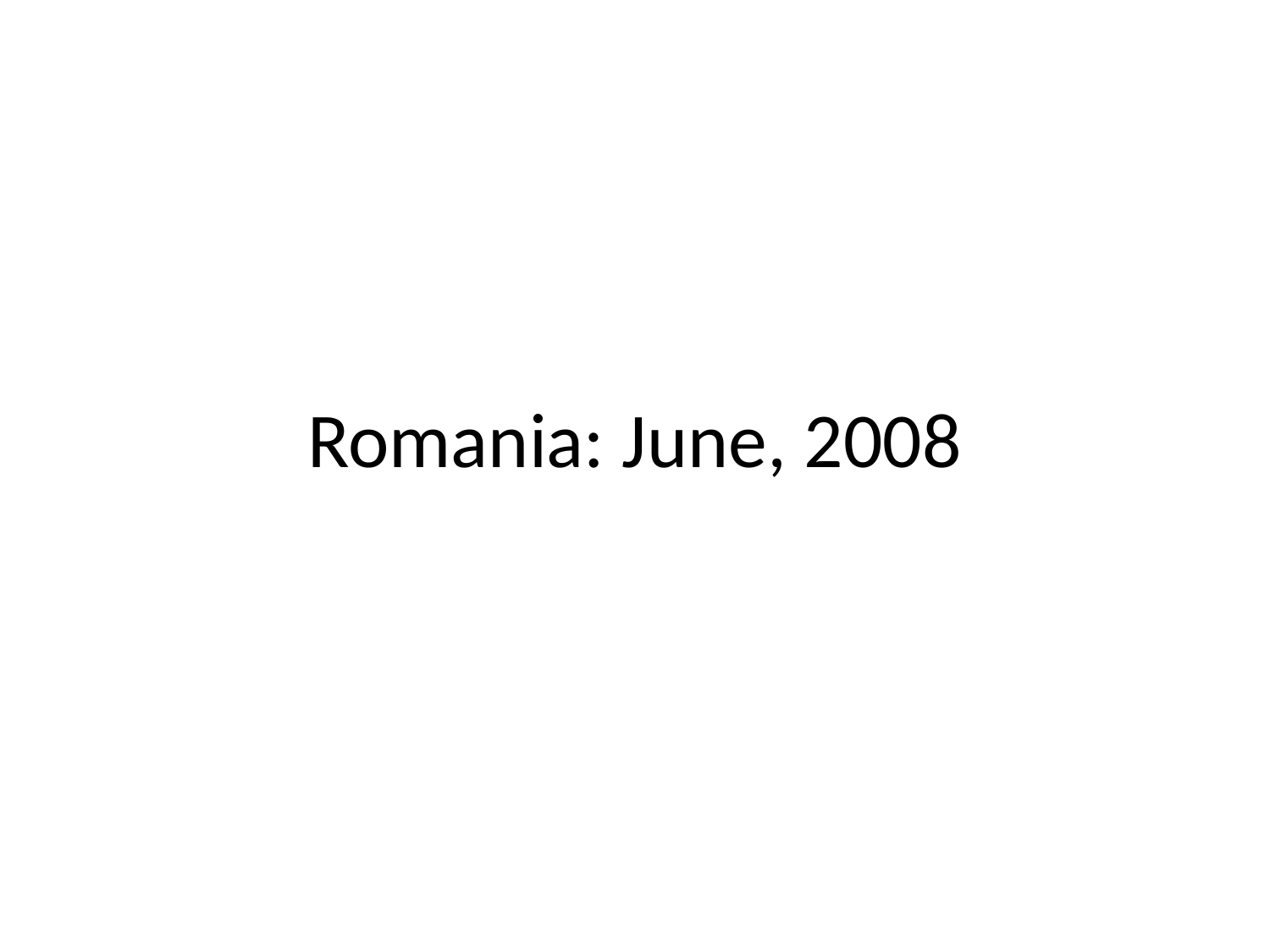

# Romania: June, 2008

## Slide 9
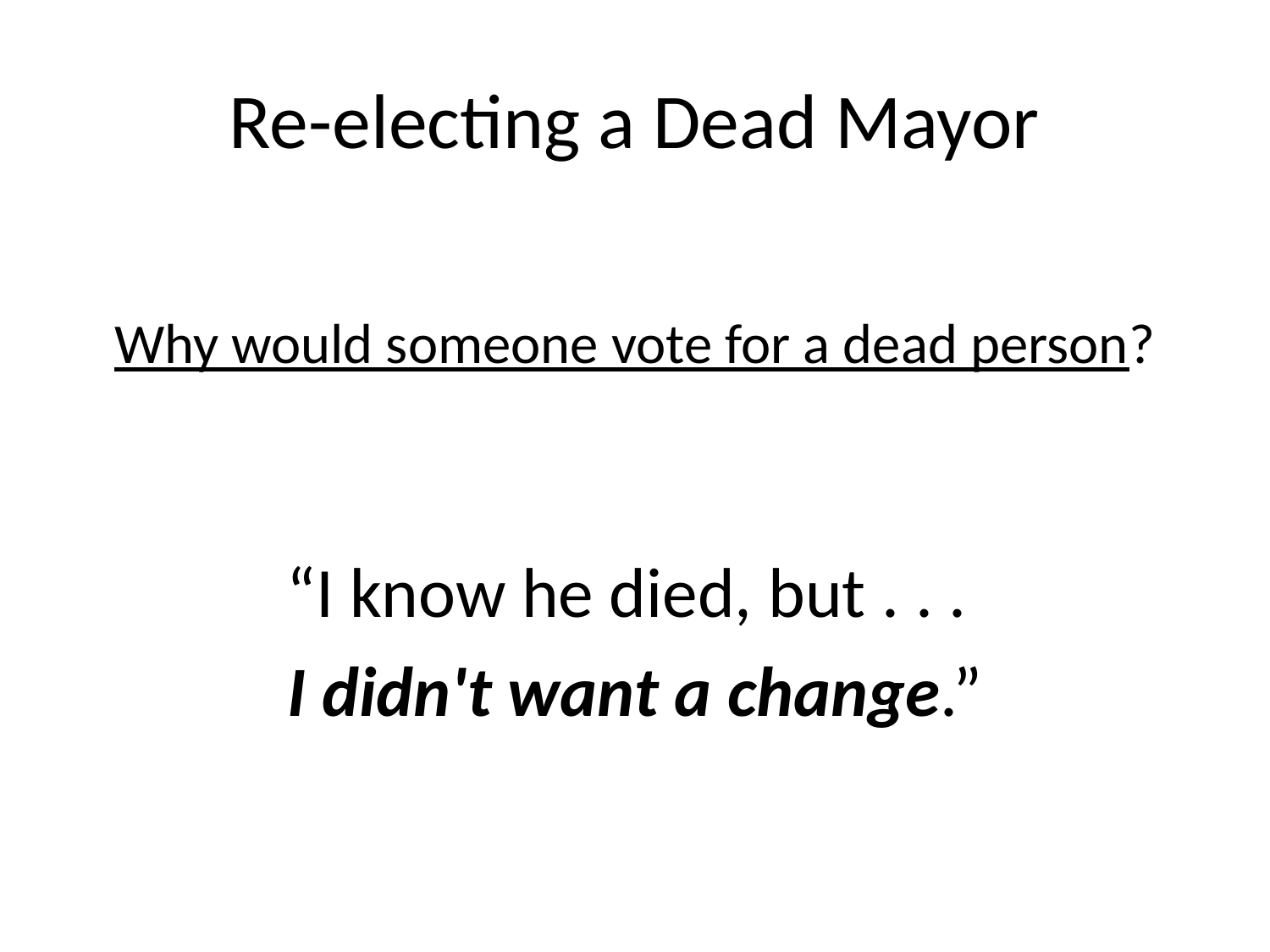

# Re-electing a Dead Mayor
Why would someone vote for a dead person?
“I know he died, but . . .
I didn't want a change.”

## Slide 10
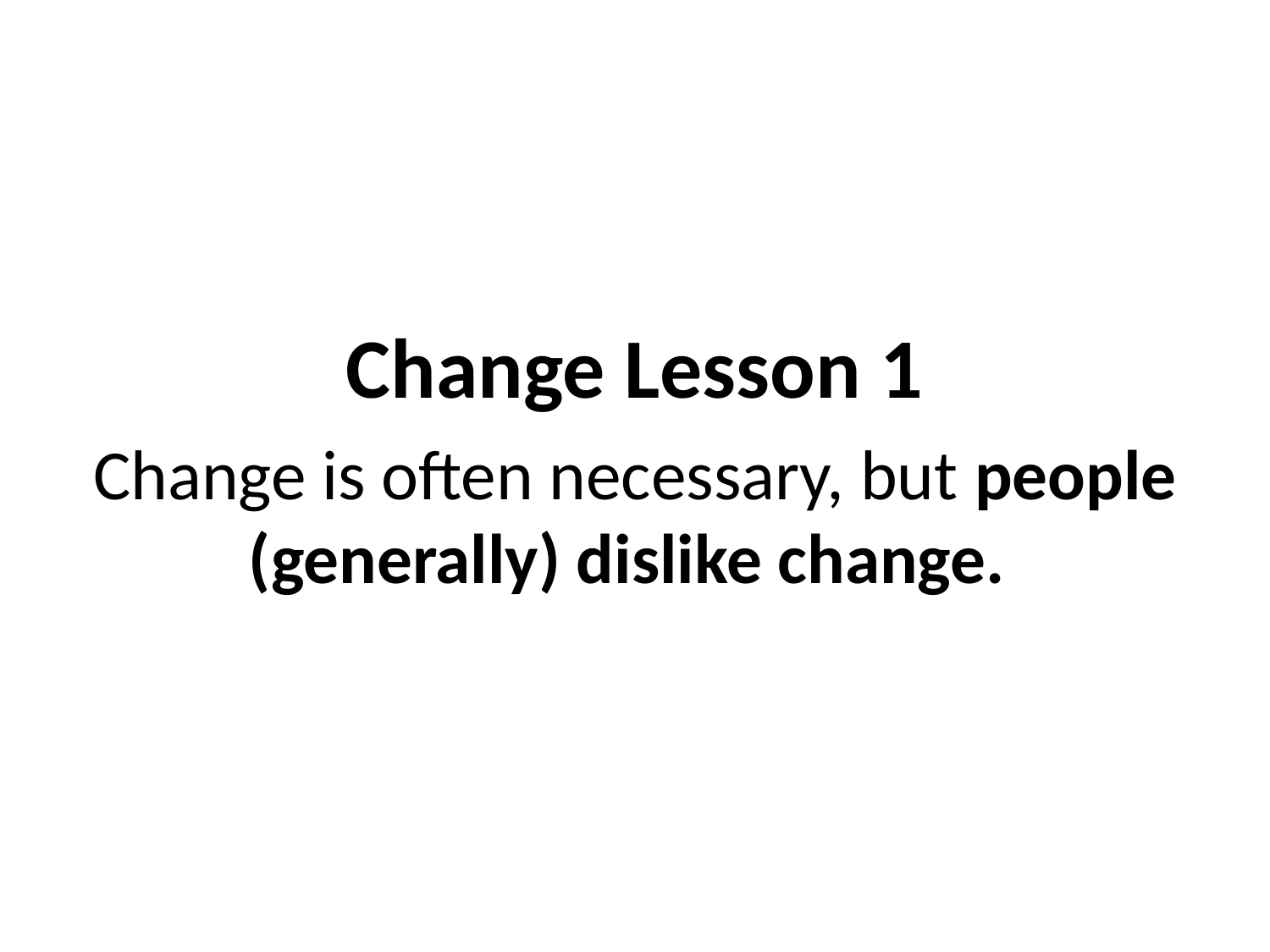

Change Lesson 1
Change is often necessary, but people (generally) dislike change.

## Slide 11
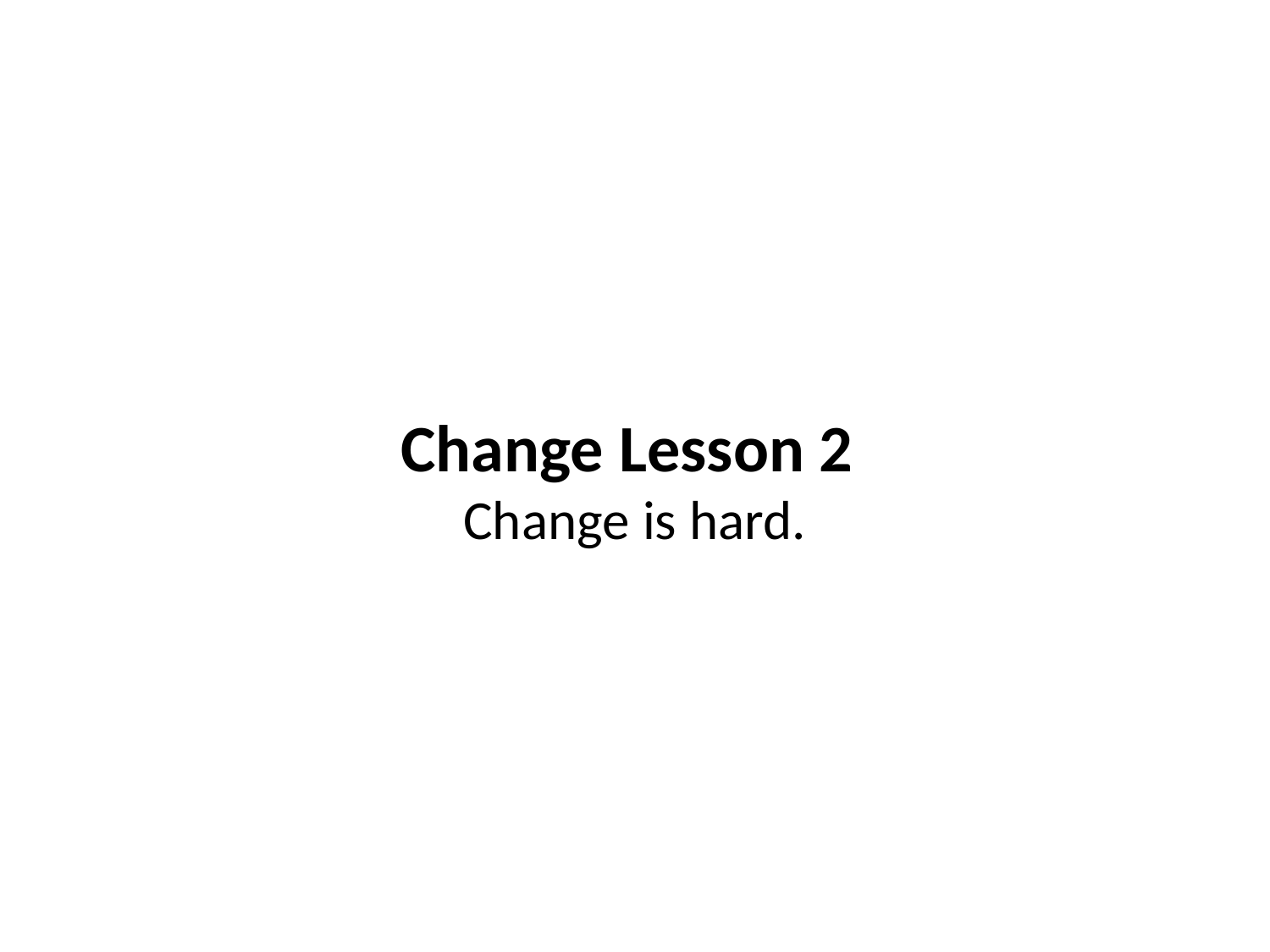

# Change Lesson 2 Change is hard.

## Slide 12
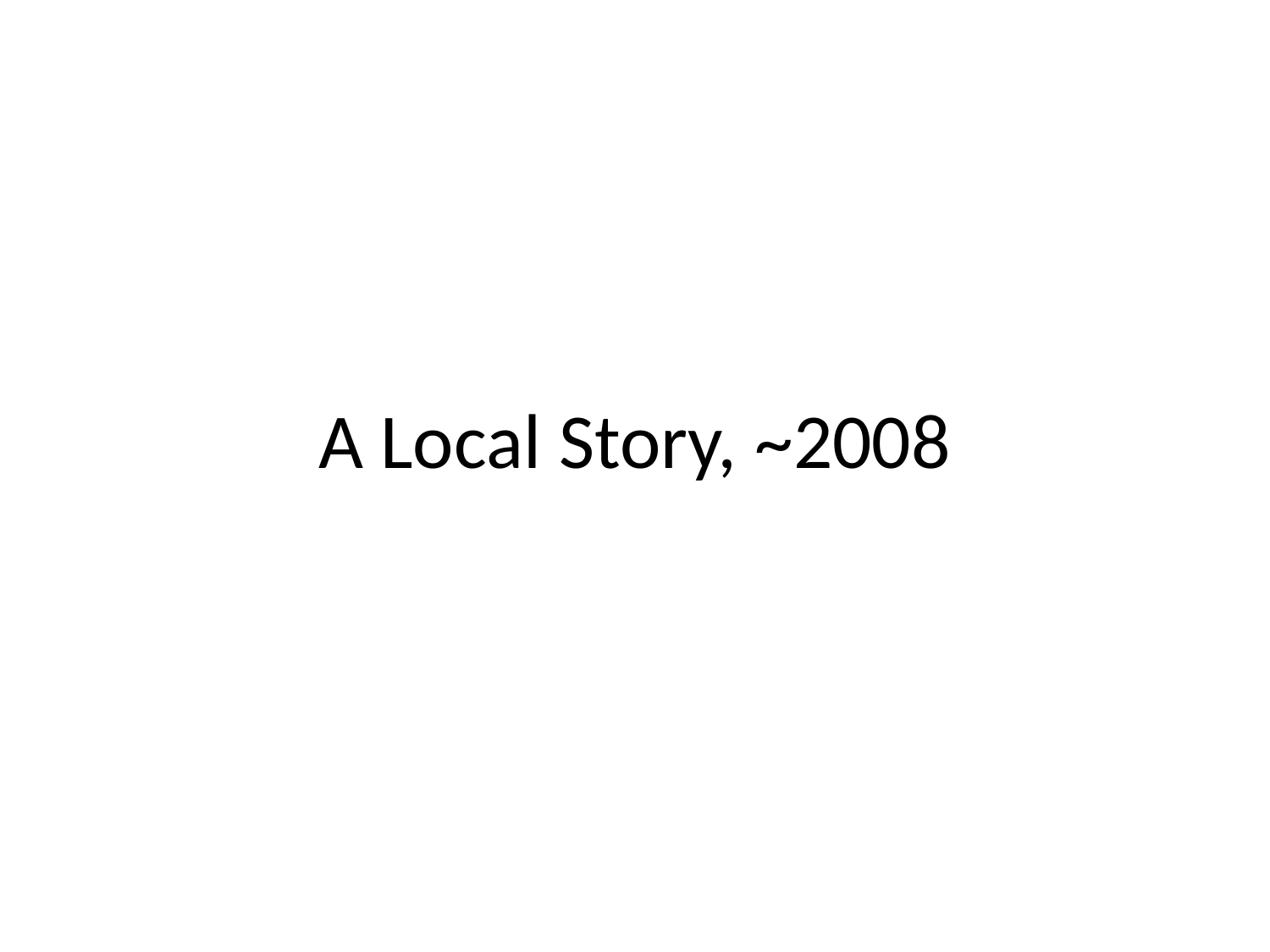

# A Local Story, ~2008

## Slide 13
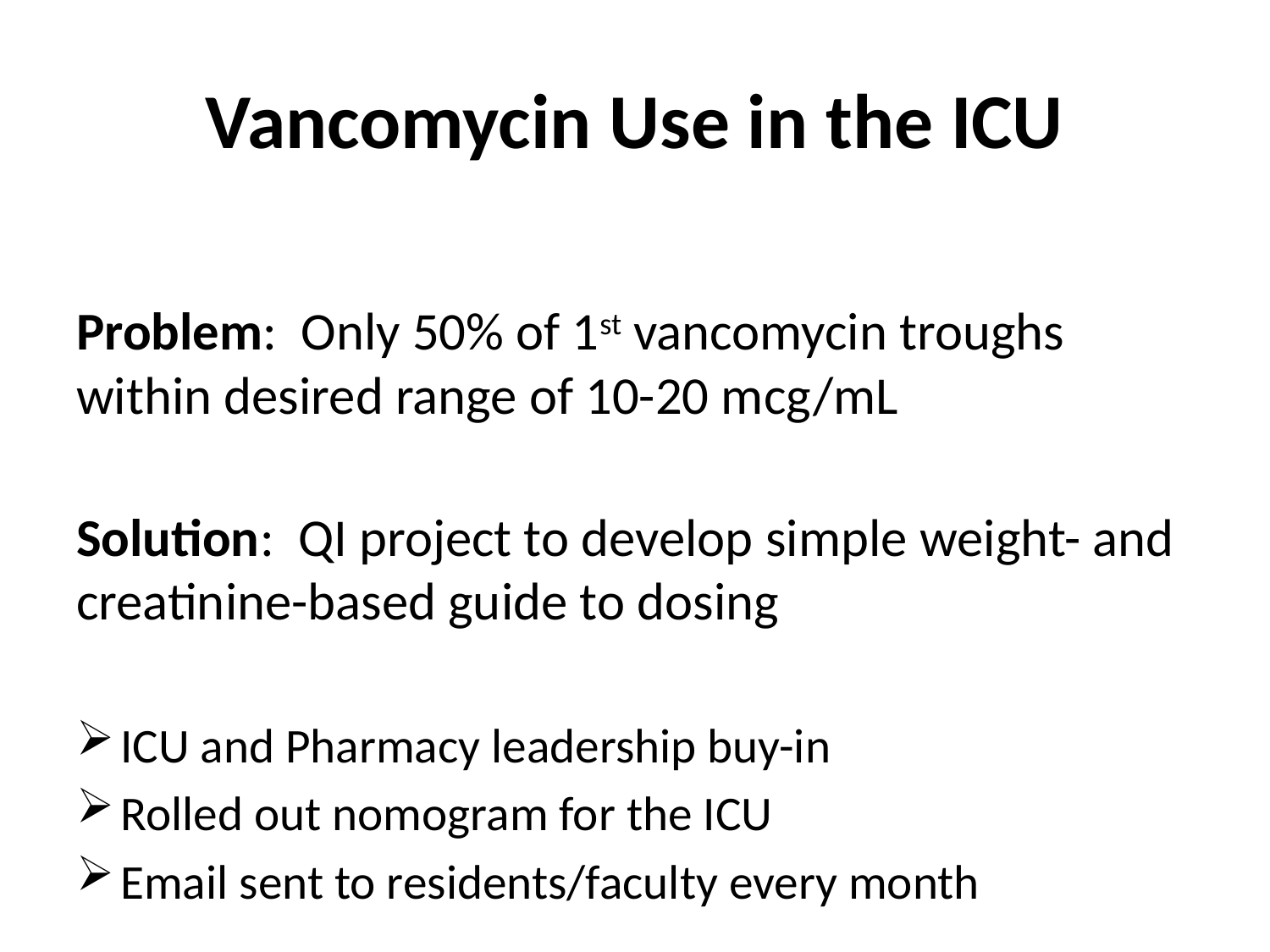

# Vancomycin Use in the ICU
Problem: Only 50% of 1st vancomycin troughs within desired range of 10-20 mcg/mL
Solution: QI project to develop simple weight- and creatinine-based guide to dosing
ICU and Pharmacy leadership buy-in
Rolled out nomogram for the ICU
Email sent to residents/faculty every month

## Slide 14
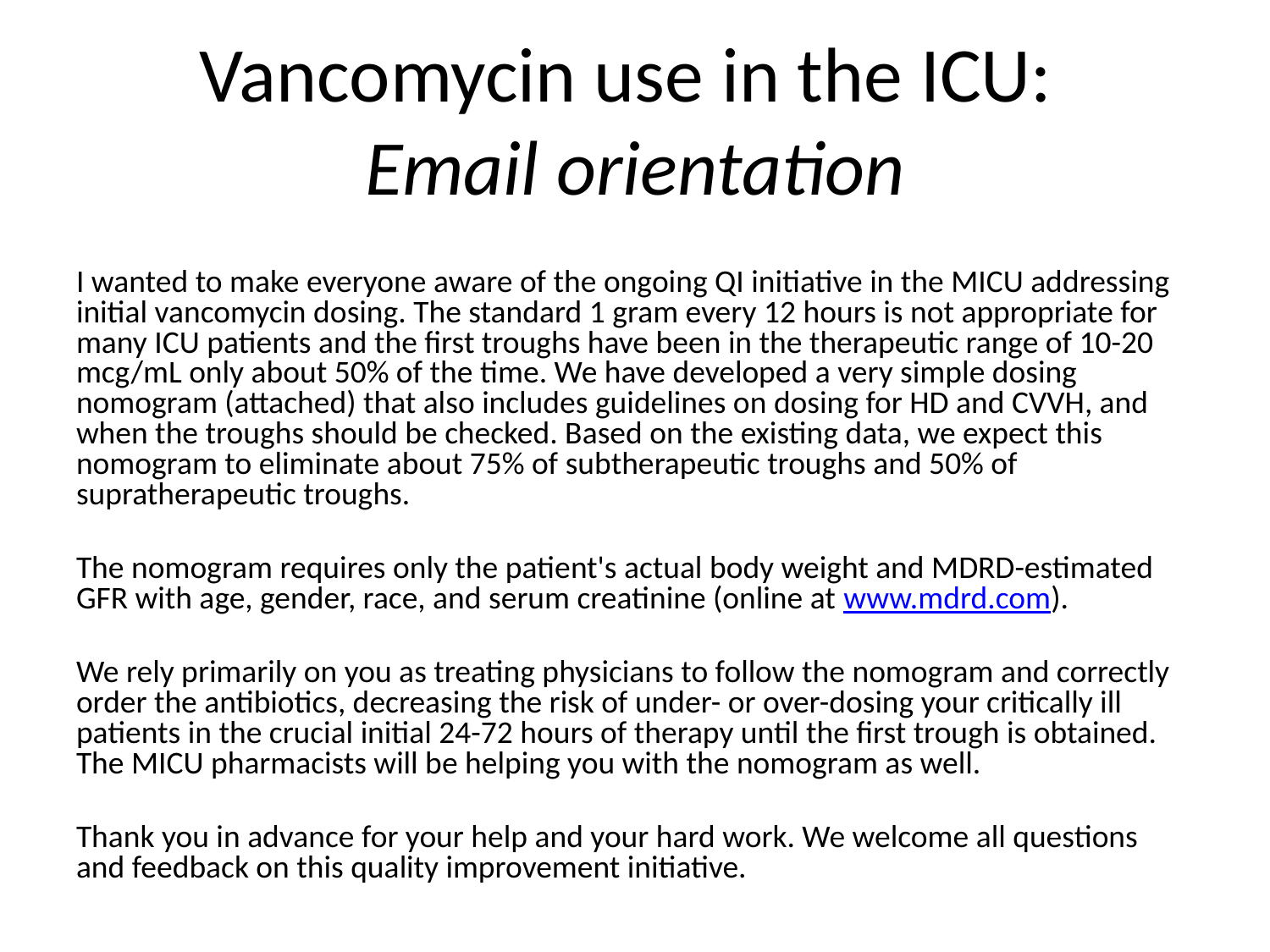

# Vancomycin use in the ICU: Email orientation
I wanted to make everyone aware of the ongoing QI initiative in the MICU addressing initial vancomycin dosing. The standard 1 gram every 12 hours is not appropriate for many ICU patients and the first troughs have been in the therapeutic range of 10-20 mcg/mL only about 50% of the time. We have developed a very simple dosing nomogram (attached) that also includes guidelines on dosing for HD and CVVH, and when the troughs should be checked. Based on the existing data, we expect this nomogram to eliminate about 75% of subtherapeutic troughs and 50% of supratherapeutic troughs.
The nomogram requires only the patient's actual body weight and MDRD-estimated GFR with age, gender, race, and serum creatinine (online at www.mdrd.com).
We rely primarily on you as treating physicians to follow the nomogram and correctly order the antibiotics, decreasing the risk of under- or over-dosing your critically ill patients in the crucial initial 24-72 hours of therapy until the first trough is obtained. The MICU pharmacists will be helping you with the nomogram as well.
Thank you in advance for your help and your hard work. We welcome all questions and feedback on this quality improvement initiative.

## Slide 15
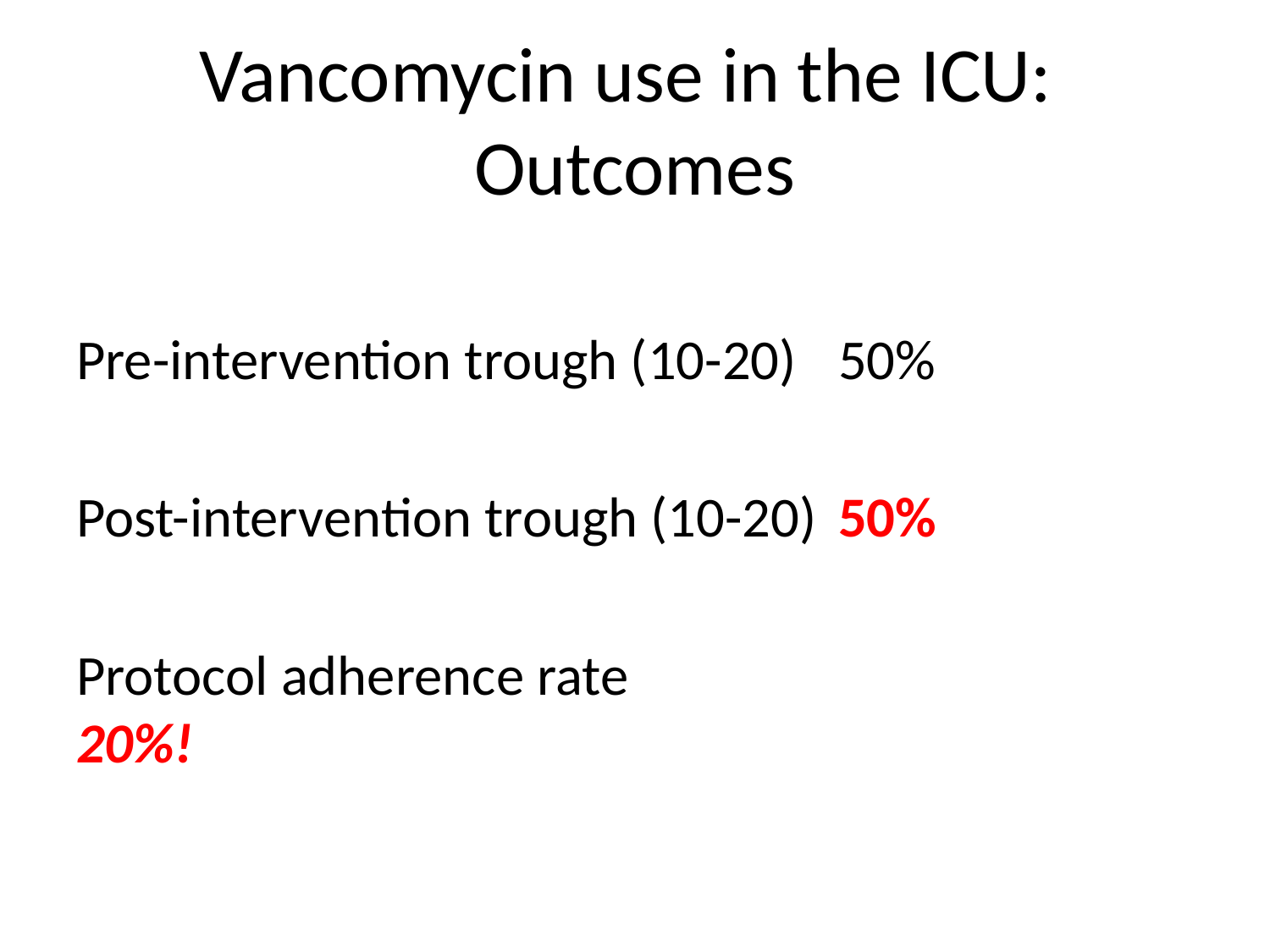

# Vancomycin use in the ICU: Outcomes
Pre-intervention trough (10-20) 	50%
Post-intervention trough (10-20)	50%
Protocol adherence rate				20%!

## Slide 16
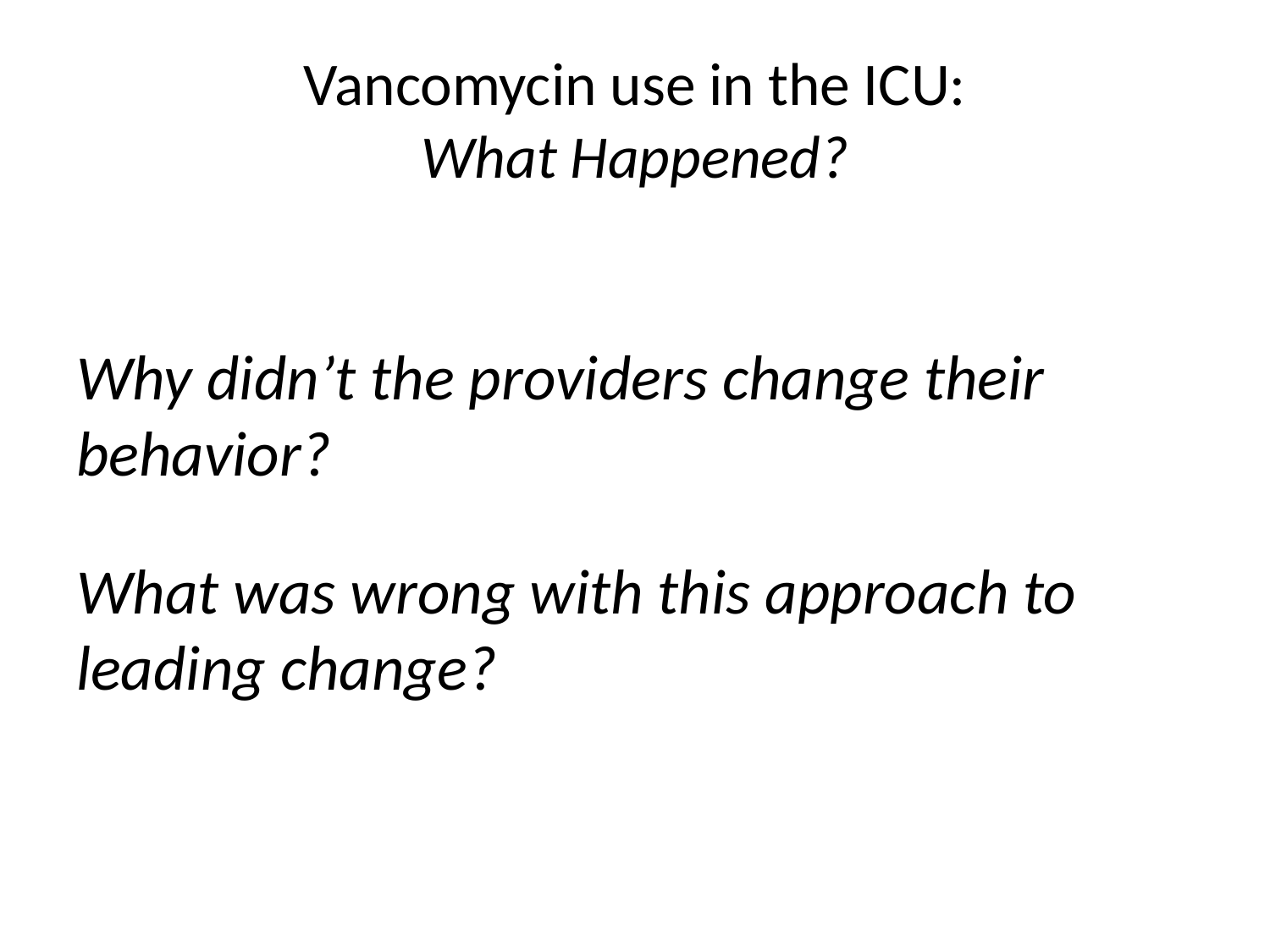

# Vancomycin use in the ICU: What Happened?
Why didn’t the providers change their behavior?
What was wrong with this approach to leading change?

## Slide 17
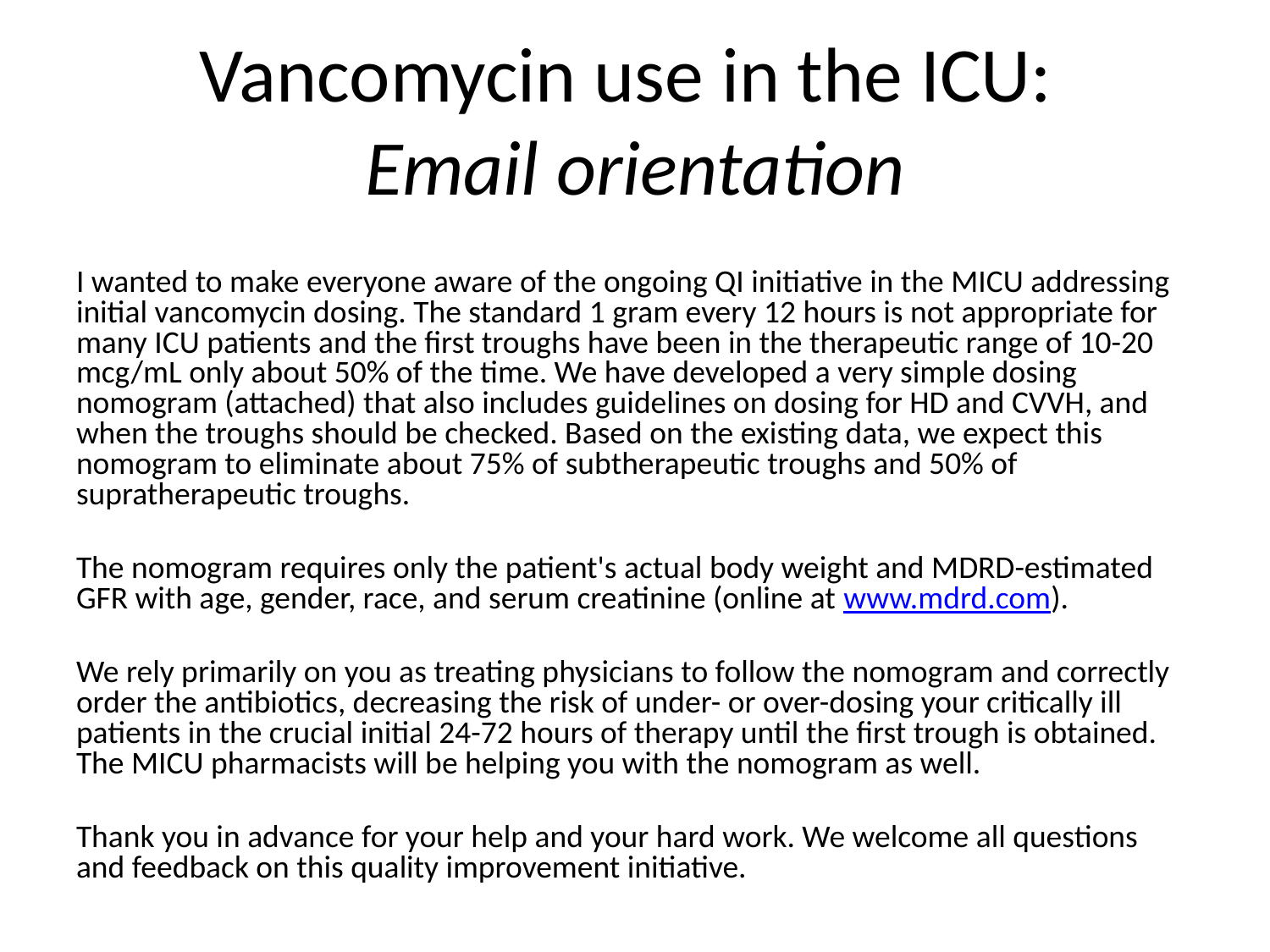

# Vancomycin use in the ICU: Email orientation
I wanted to make everyone aware of the ongoing QI initiative in the MICU addressing initial vancomycin dosing. The standard 1 gram every 12 hours is not appropriate for many ICU patients and the first troughs have been in the therapeutic range of 10-20 mcg/mL only about 50% of the time. We have developed a very simple dosing nomogram (attached) that also includes guidelines on dosing for HD and CVVH, and when the troughs should be checked. Based on the existing data, we expect this nomogram to eliminate about 75% of subtherapeutic troughs and 50% of supratherapeutic troughs.
The nomogram requires only the patient's actual body weight and MDRD-estimated GFR with age, gender, race, and serum creatinine (online at www.mdrd.com).
We rely primarily on you as treating physicians to follow the nomogram and correctly order the antibiotics, decreasing the risk of under- or over-dosing your critically ill patients in the crucial initial 24-72 hours of therapy until the first trough is obtained. The MICU pharmacists will be helping you with the nomogram as well.
Thank you in advance for your help and your hard work. We welcome all questions and feedback on this quality improvement initiative.

## Slide 18
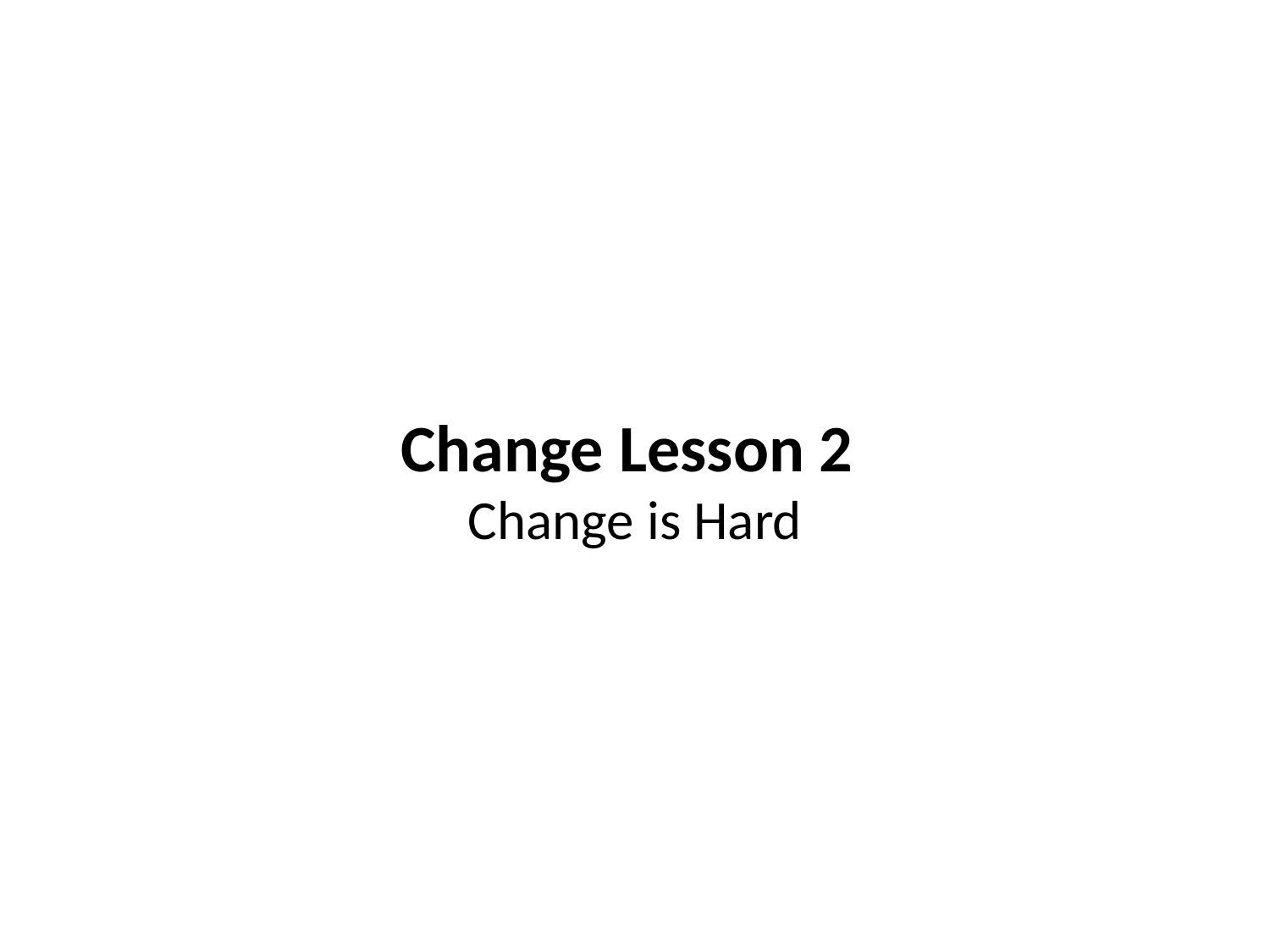

# Change Lesson 2 Change is Hard

## Slide 19
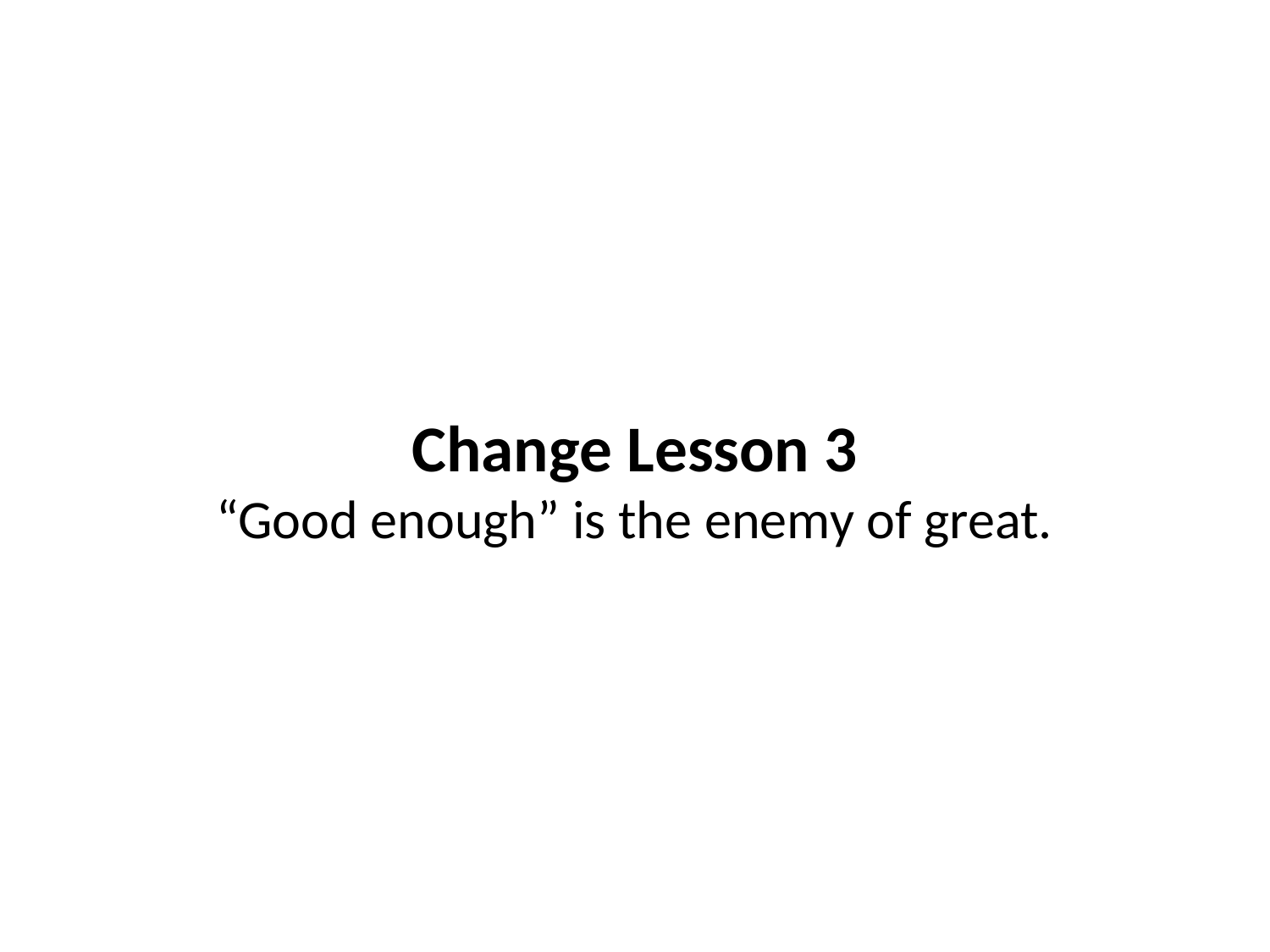

# Change Lesson 3 “Good enough” is the enemy of great.

## Slide 20
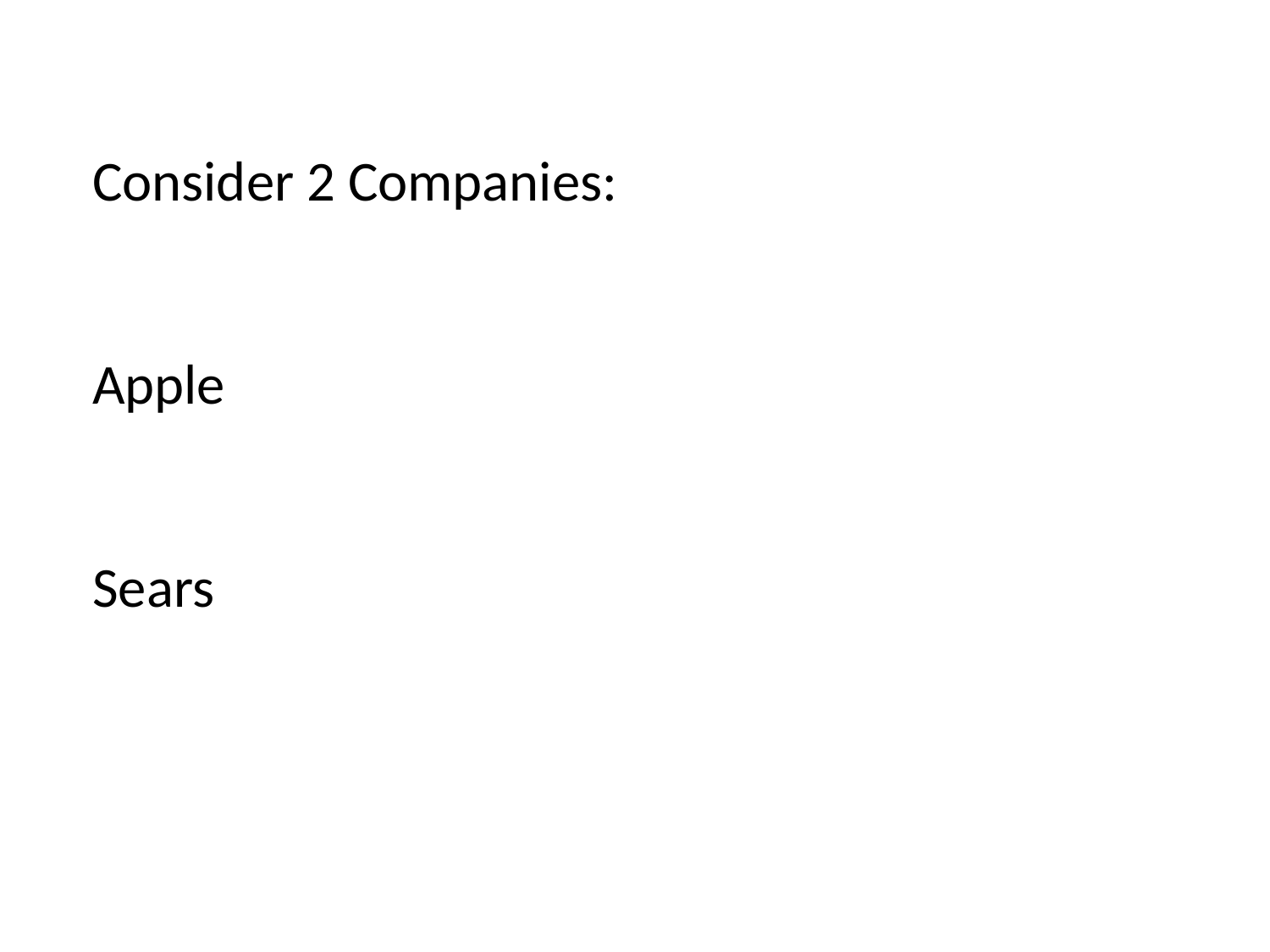

Consider 2 Companies:
Apple
Sears

## Slide 21
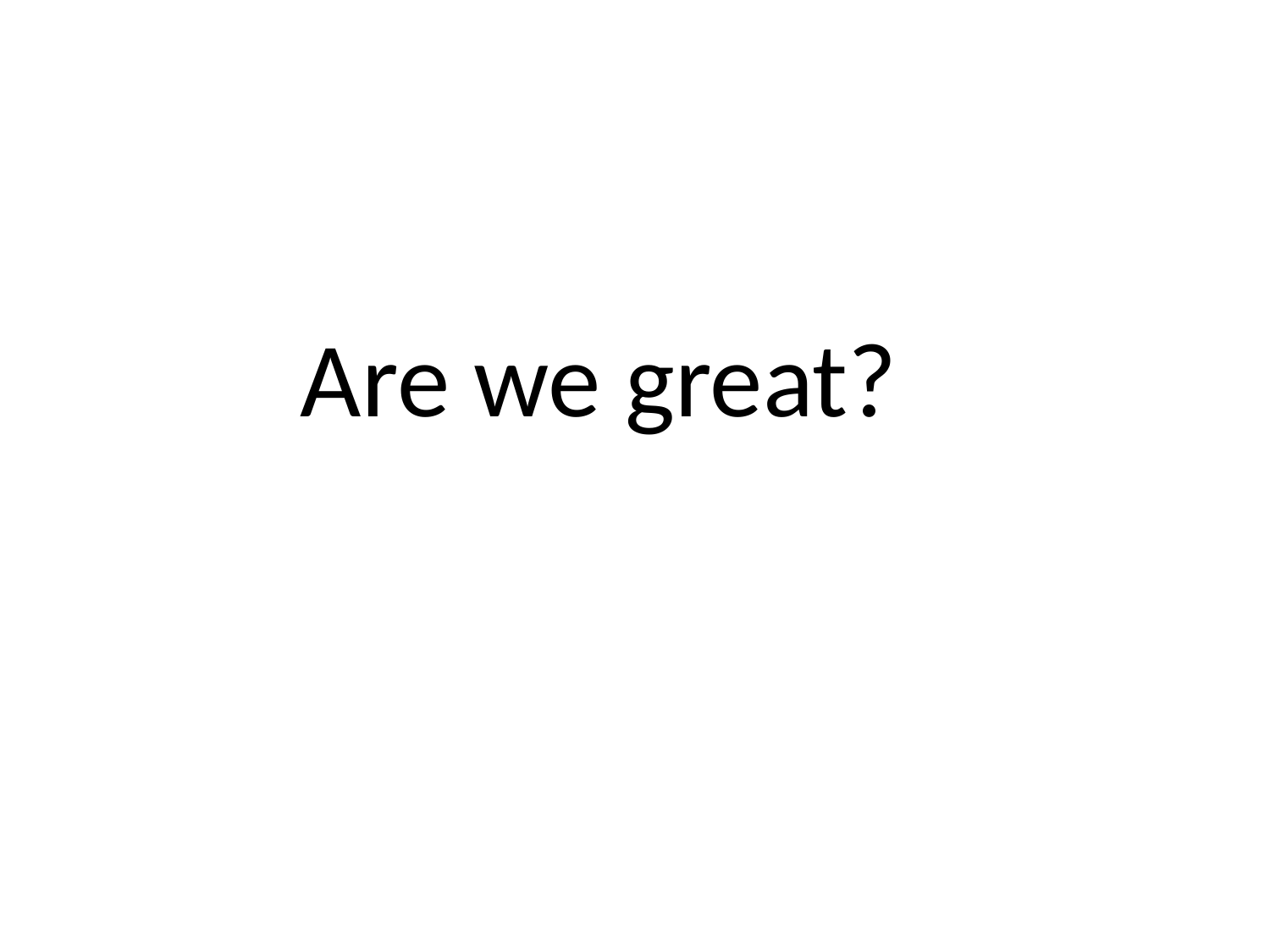

Are we great?

## Slide 22
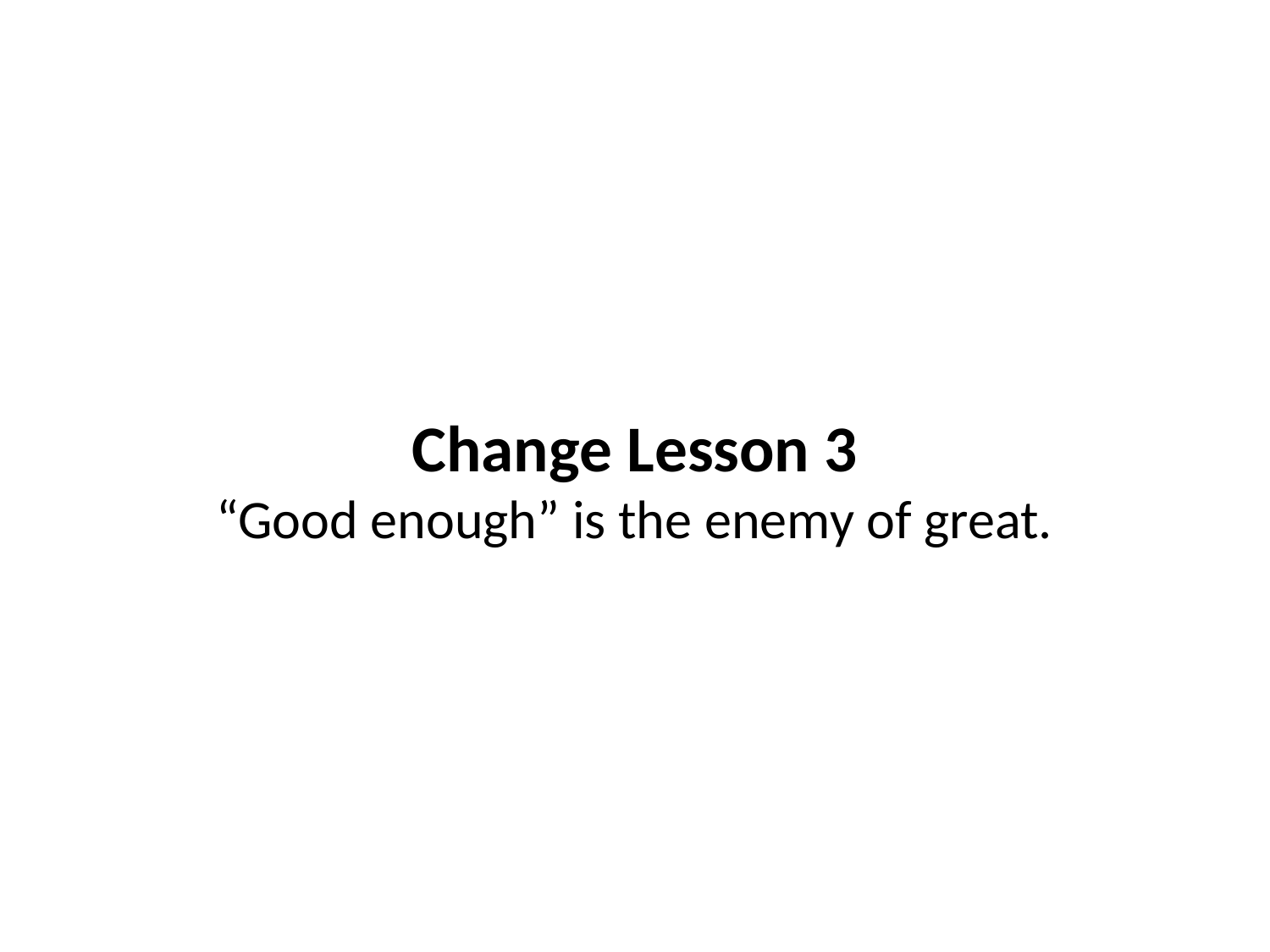

# Change Lesson 3 “Good enough” is the enemy of great.

## Slide 23
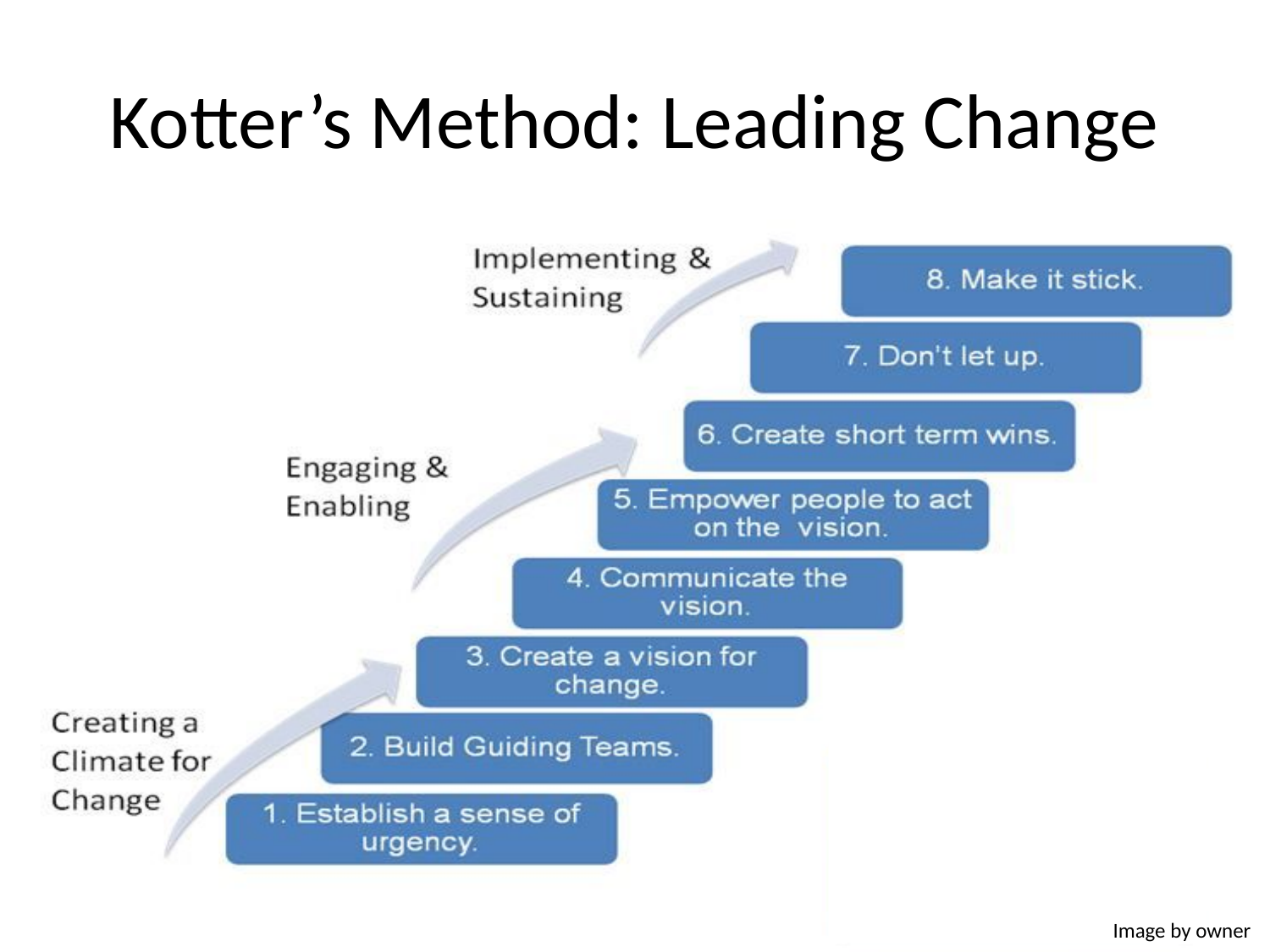

# Kotter’s Method: Leading Change
Image by owner

## Slide 24
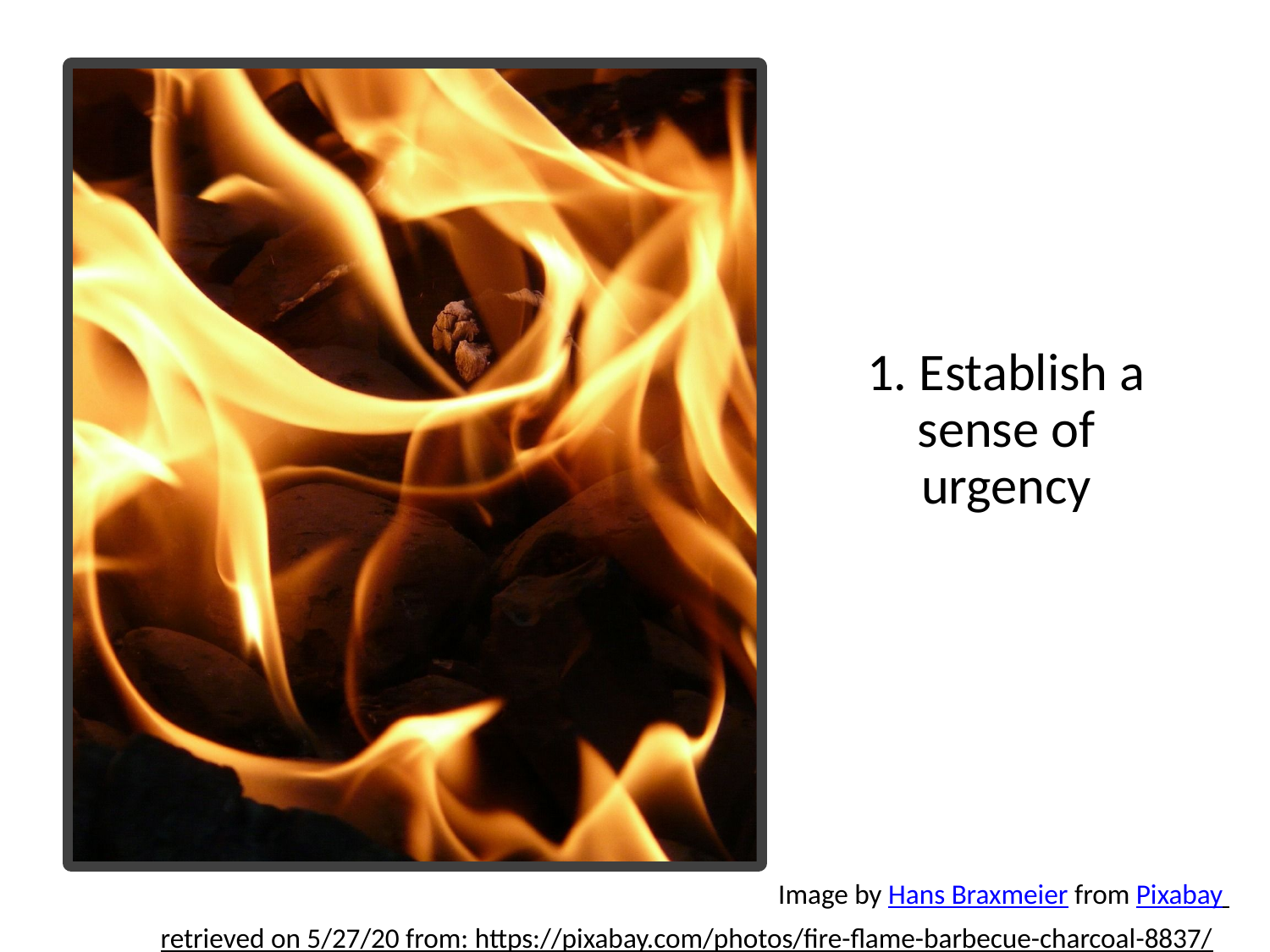

# 1. Establish a sense of urgency
Image by Hans Braxmeier from Pixabay
retrieved on 5/27/20 from: https://pixabay.com/photos/fire-flame-barbecue-charcoal-8837/ /

## Slide 25
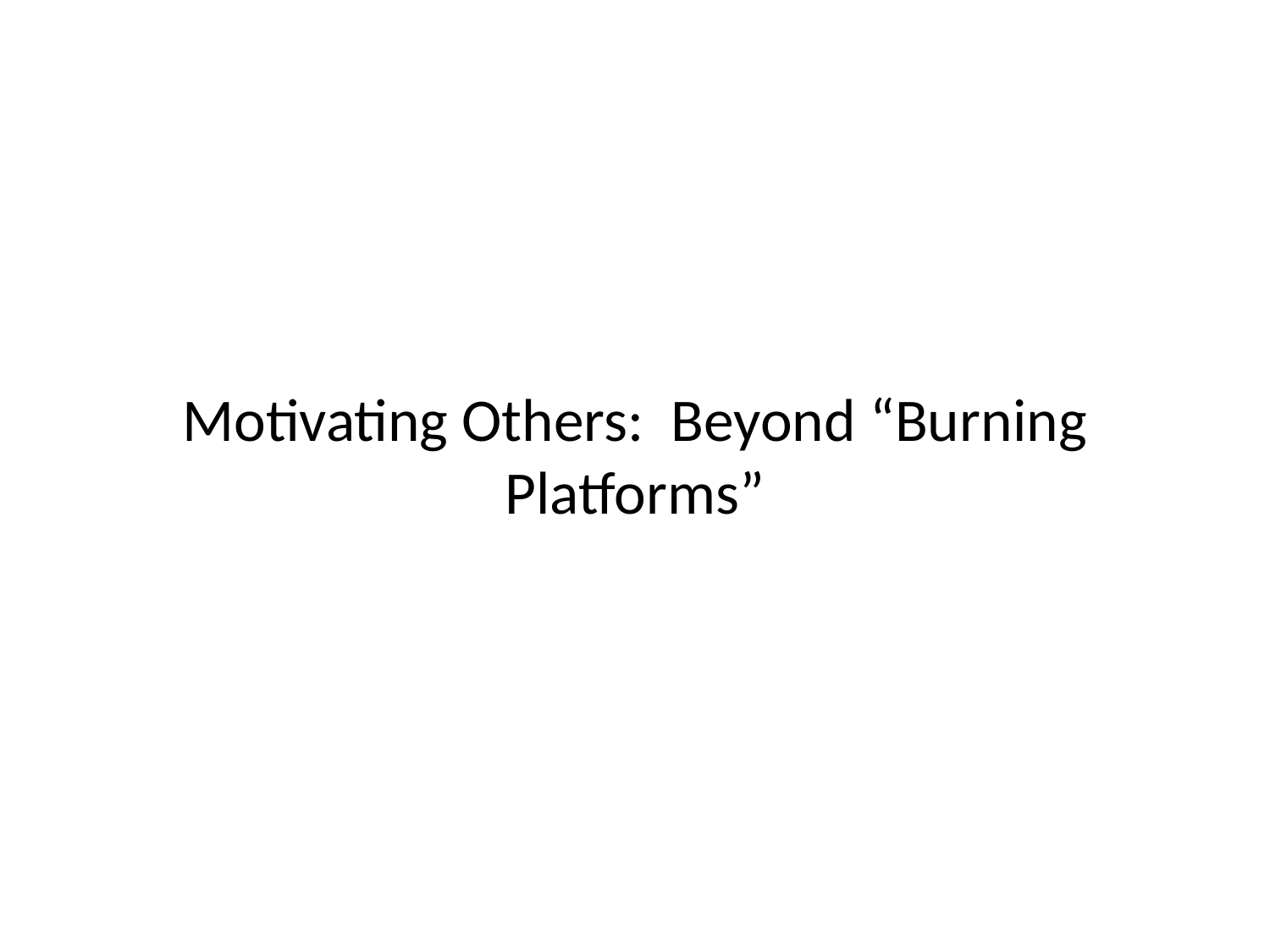

# Motivating Others: Beyond “Burning Platforms”

## Slide 26
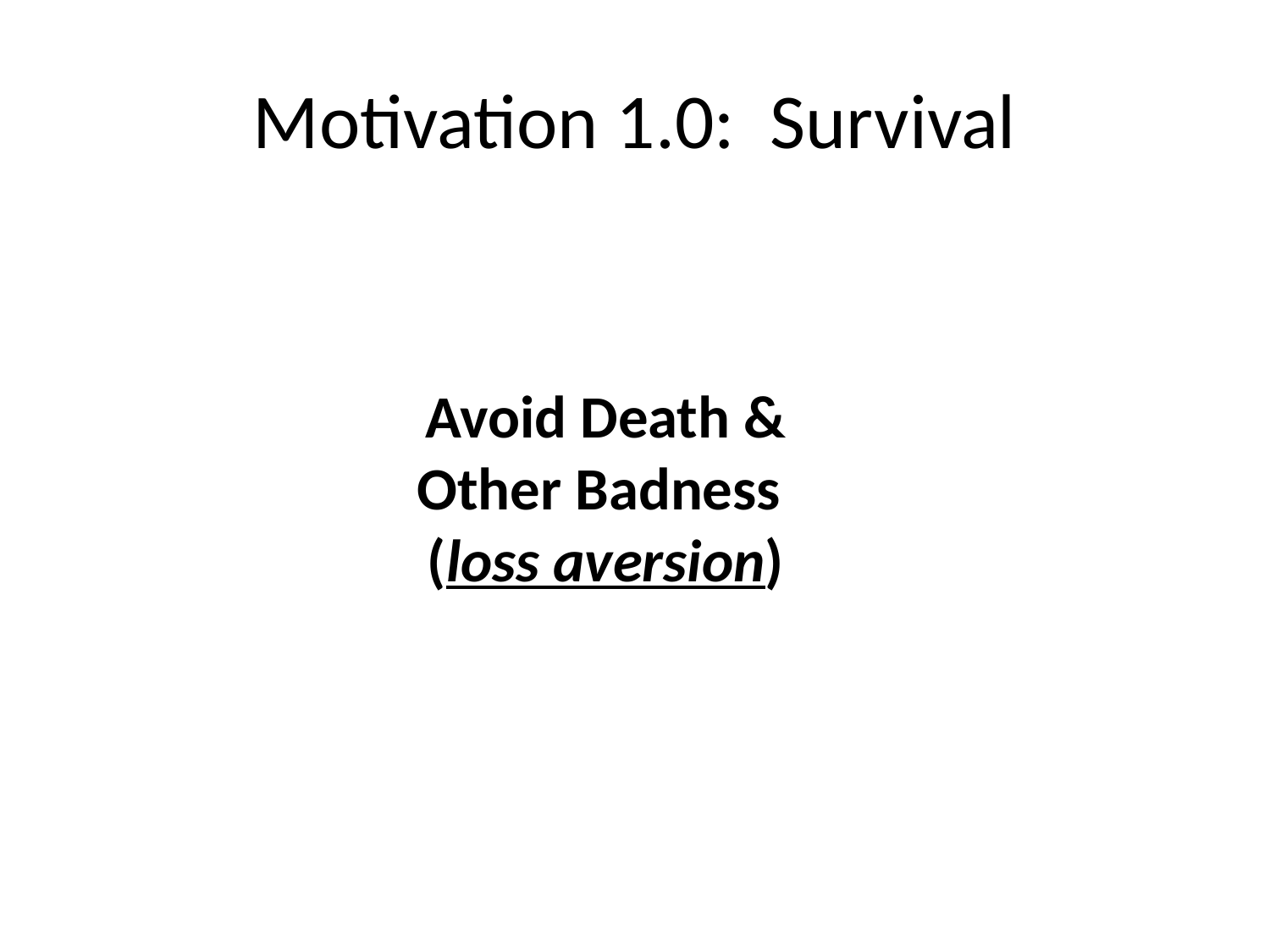

# Motivation 1.0: Survival
Avoid Death & Other Badness
(loss aversion)

## Slide 27
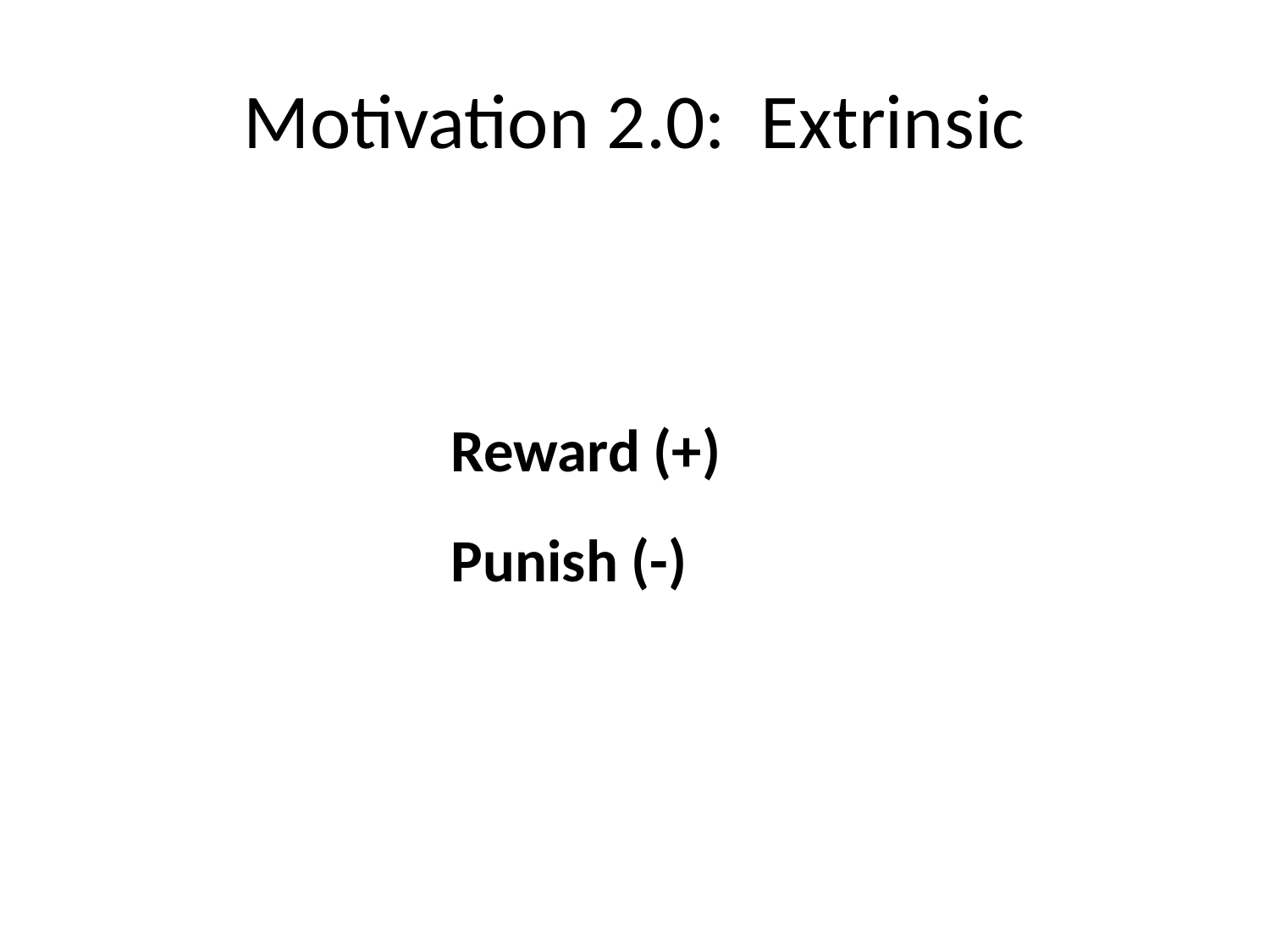

# Motivation 2.0: Extrinsic
Reward (+)
Punish (-)

## Slide 28
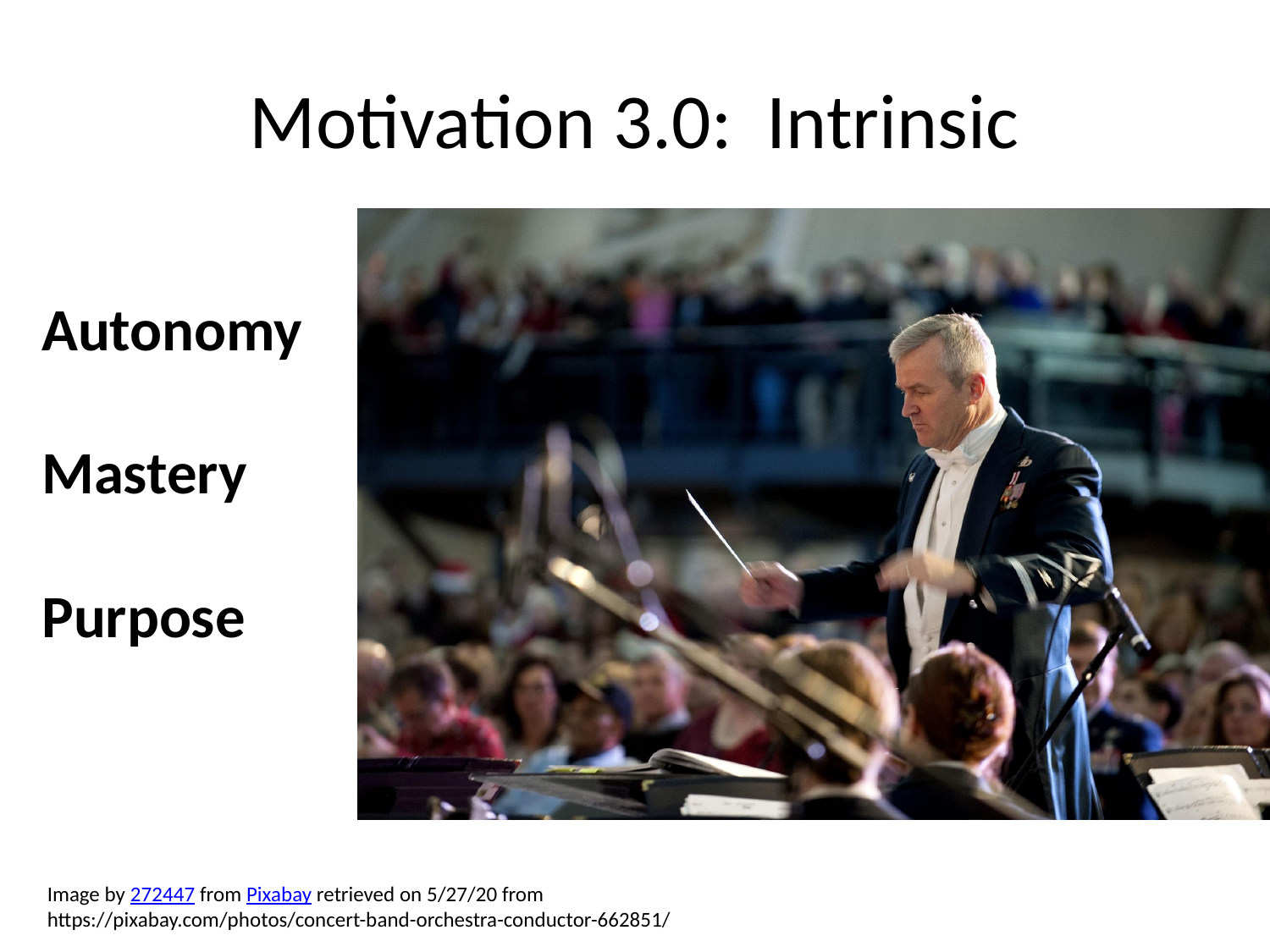

# Motivation 3.0: Intrinsic
Autonomy
Mastery
Purpose
Image by 272447 from Pixabay retrieved on 5/27/20 from
https://pixabay.com/photos/concert-band-orchestra-conductor-662851/

## Slide 29
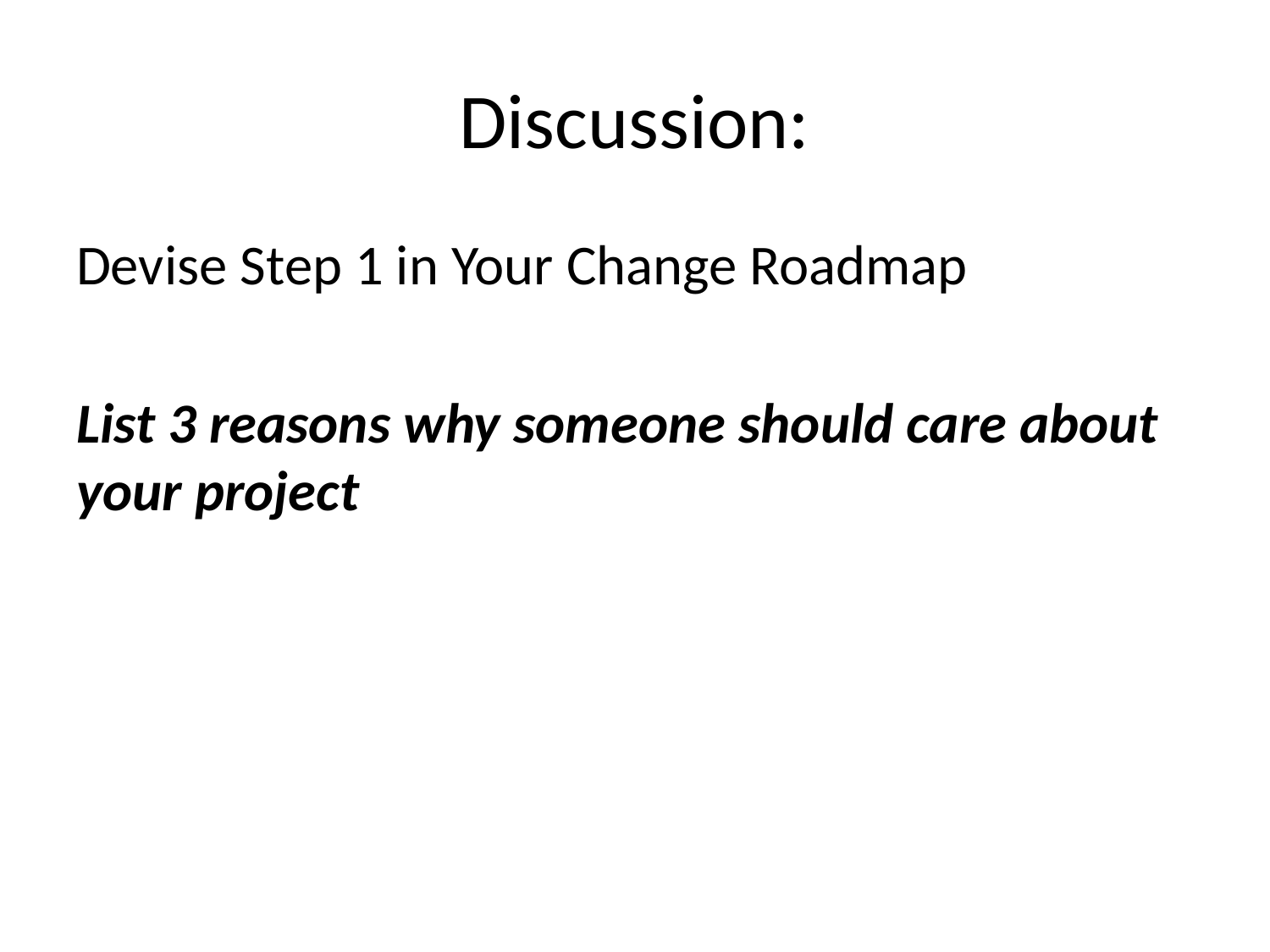

# Discussion:
Devise Step 1 in Your Change Roadmap
List 3 reasons why someone should care about your project

## Slide 30
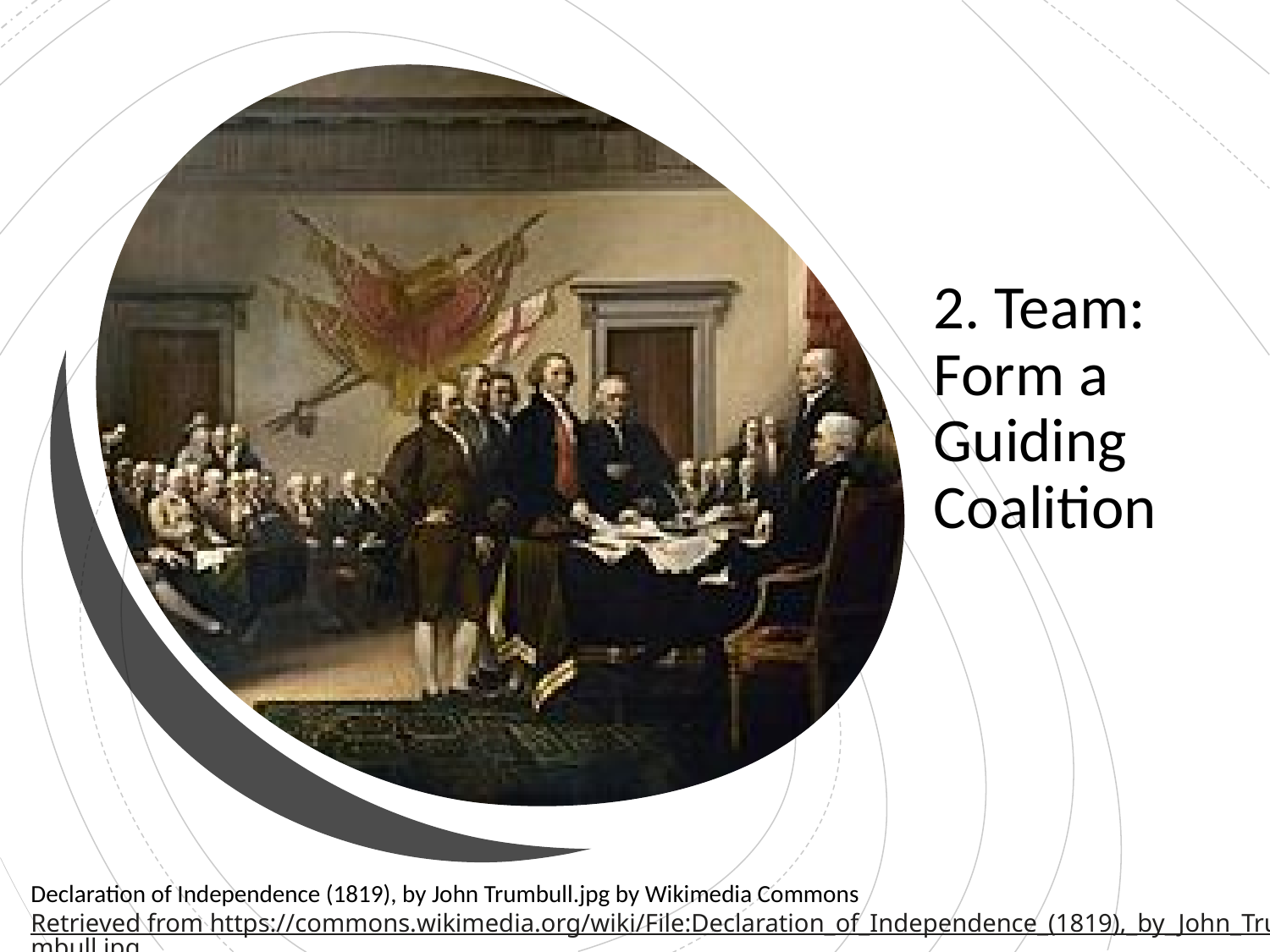

# 2. Team: Form a Guiding Coalition
Declaration of Independence (1819), by John Trumbull.jpg by Wikimedia Commons
Retrieved from https://commons.wikimedia.org/wiki/File:Declaration_of_Independence_(1819),_by_John_Trumbull.jpg).

## Slide 31
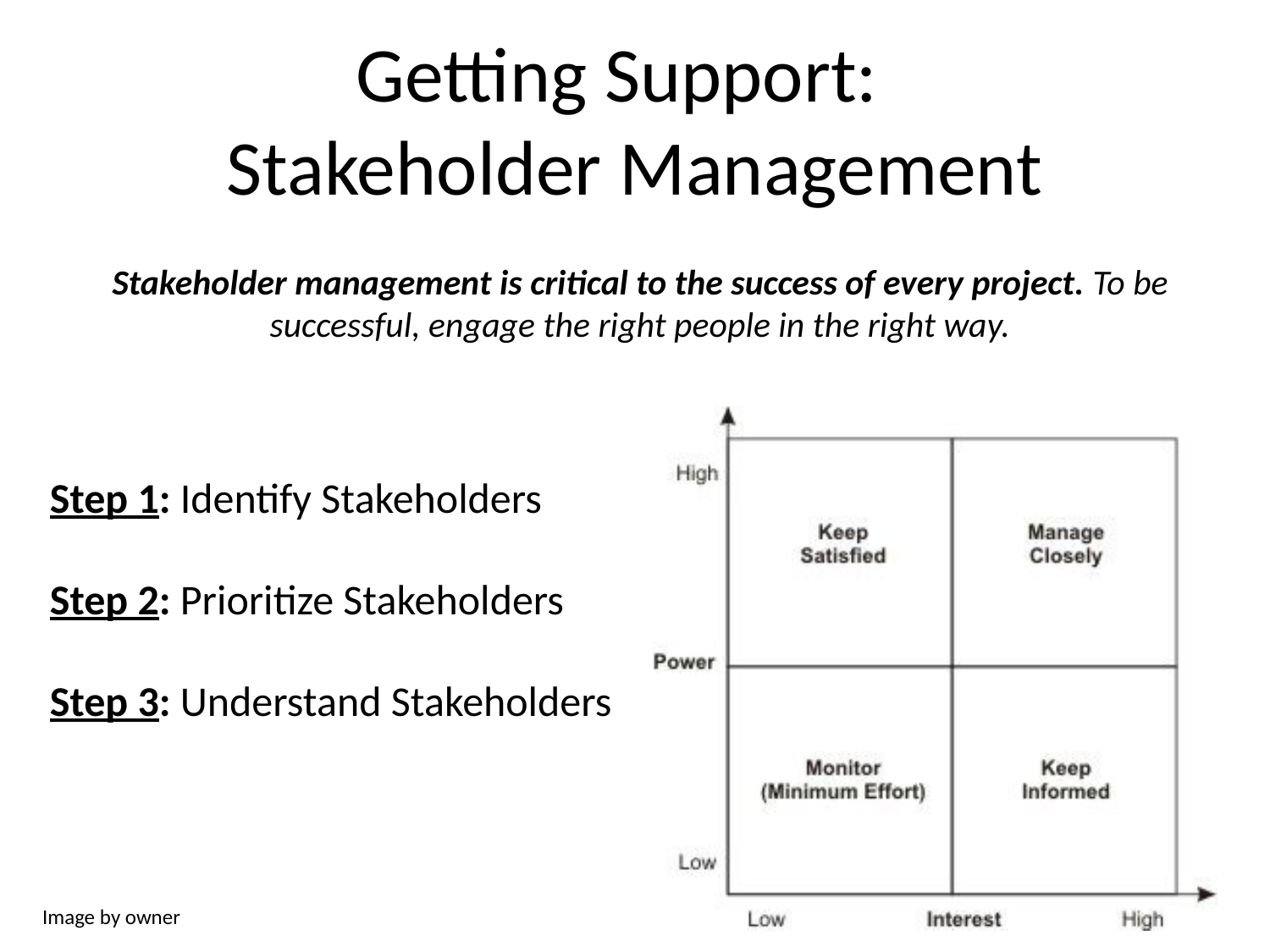

# Getting Support: Stakeholder Management
Stakeholder management is critical to the success of every project. To be successful, engage the right people in the right way.
Step 1: Identify Stakeholders
Step 2: Prioritize Stakeholders
Step 3: Understand Stakeholders
Image by owner

## Slide 32
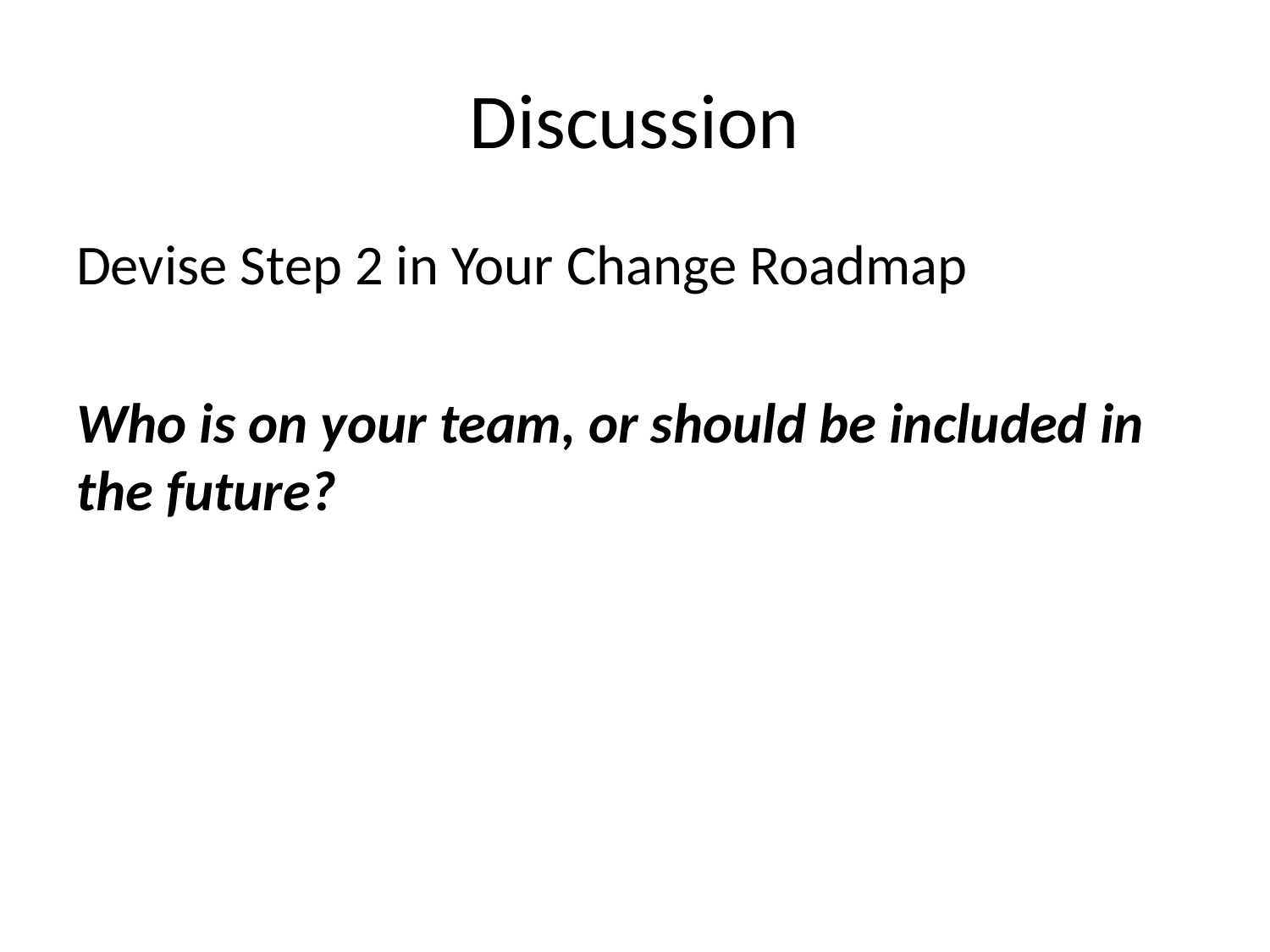

# Discussion
Devise Step 2 in Your Change Roadmap
Who is on your team, or should be included in the future?

## Slide 33
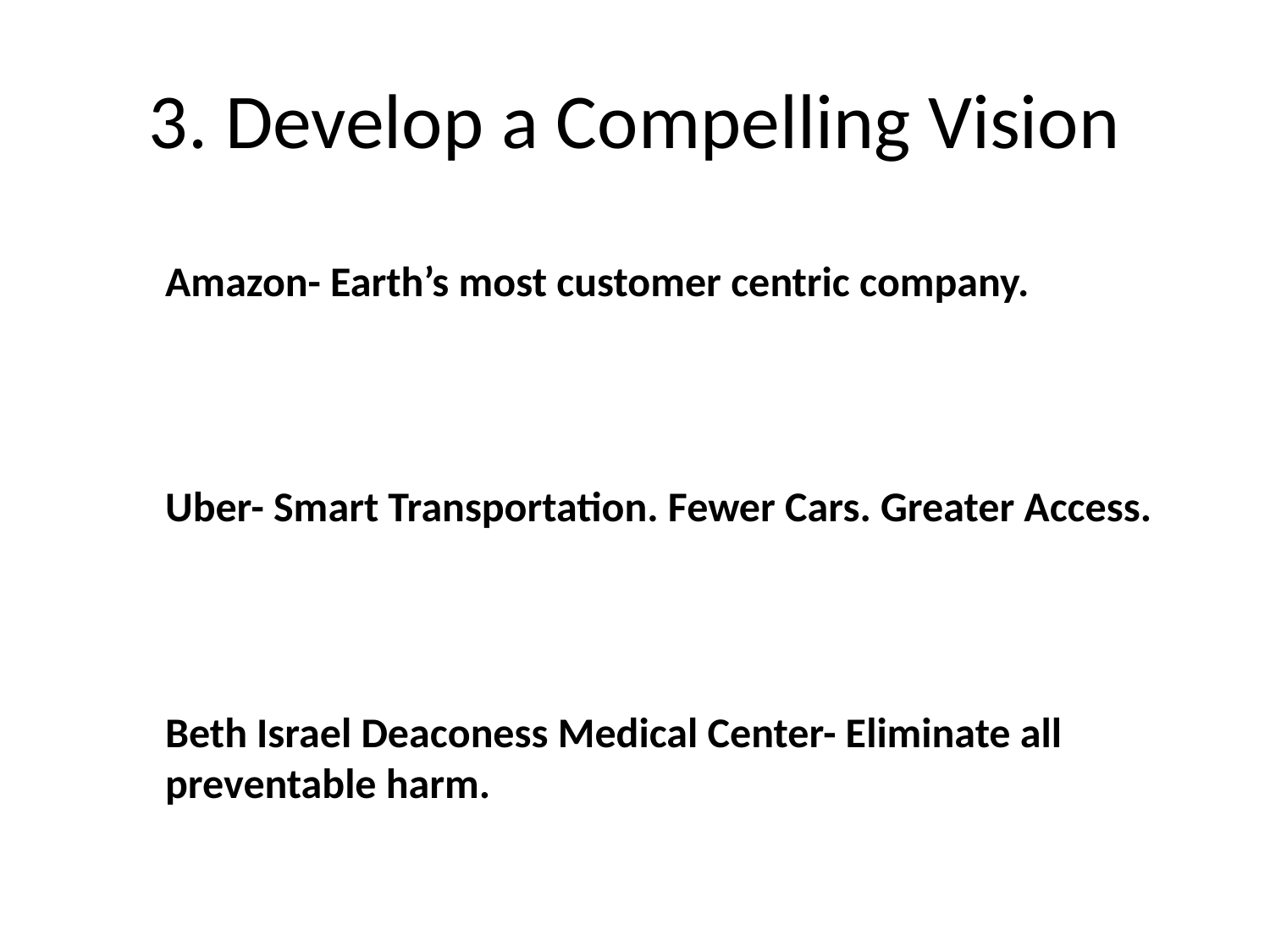

# 3. Develop a Compelling Vision
Amazon- Earth’s most customer centric company.
Uber- Smart Transportation. Fewer Cars. Greater Access.
Beth Israel Deaconess Medical Center- Eliminate all preventable harm.

## Slide 34
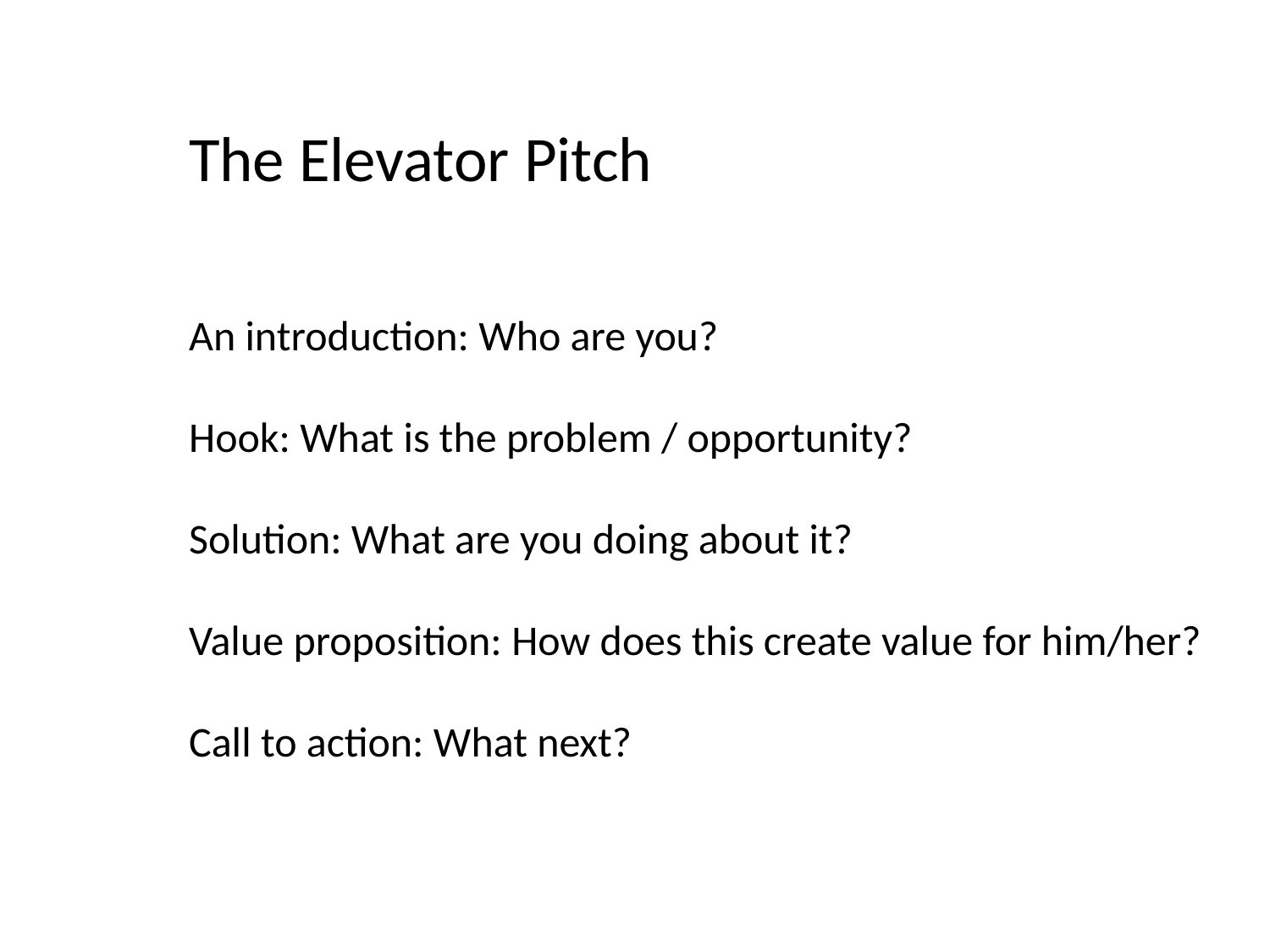

The Elevator Pitch
An introduction: Who are you?
Hook: What is the problem / opportunity?
Solution: What are you doing about it?
Value proposition: How does this create value for him/her?
Call to action: What next?

## Slide 35
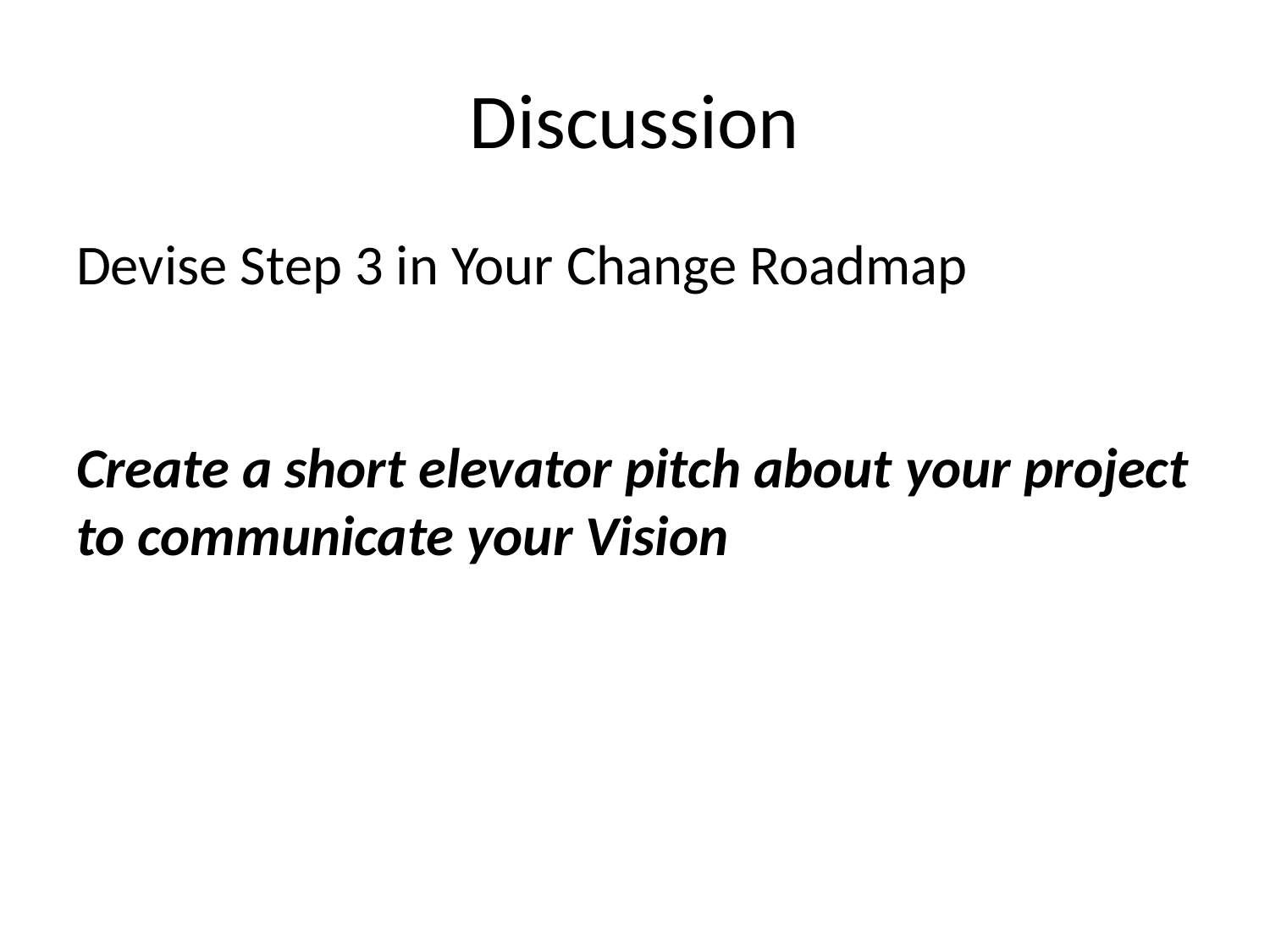

# Discussion
Devise Step 3 in Your Change Roadmap
Create a short elevator pitch about your project to communicate your Vision

## Slide 36
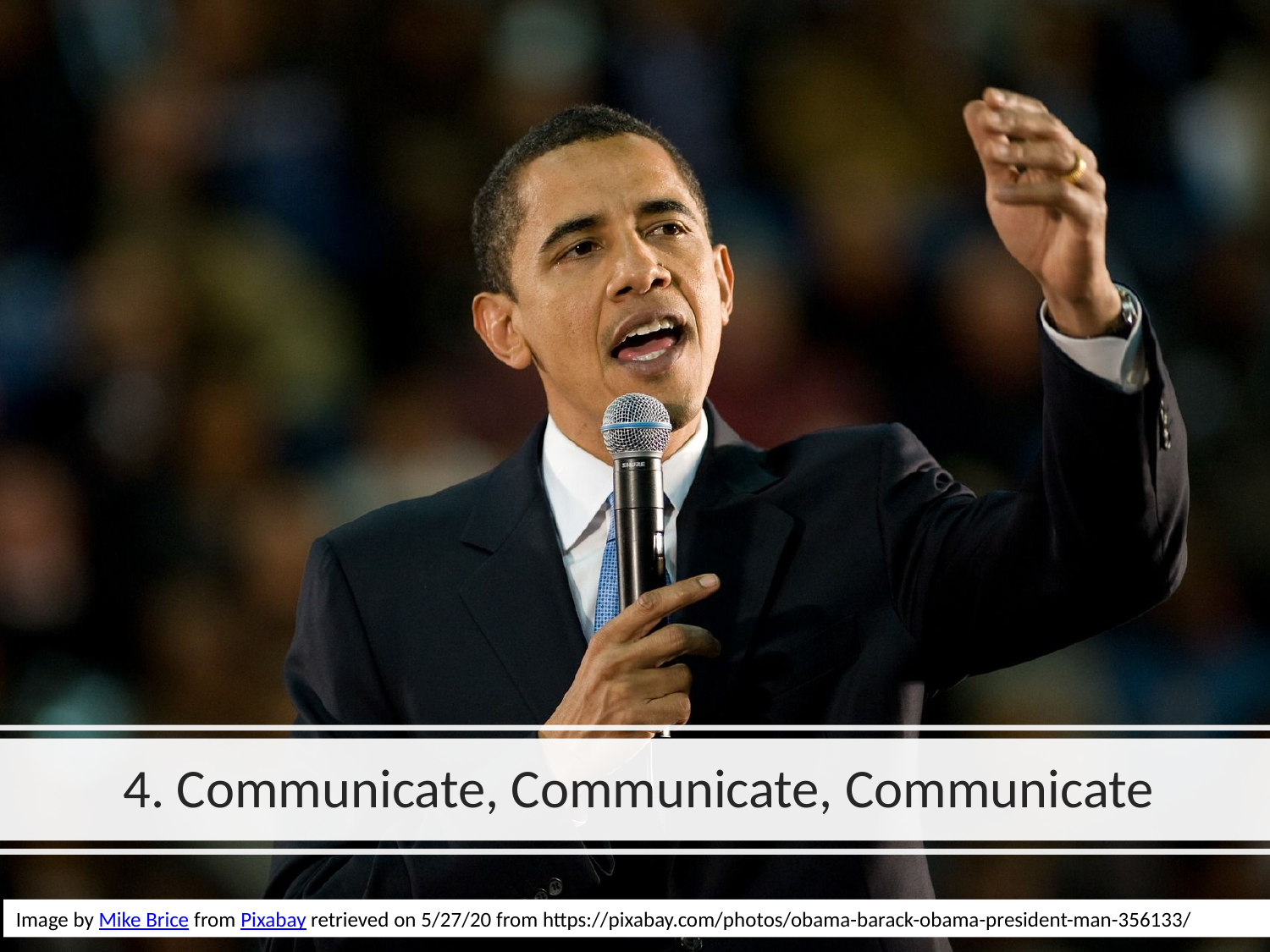

# 4. Communicate, Communicate, Communicate
Image by Mike Brice from Pixabay retrieved on 5/27/20 from https://pixabay.com/photos/obama-barack-obama-president-man-356133/

## Slide 37
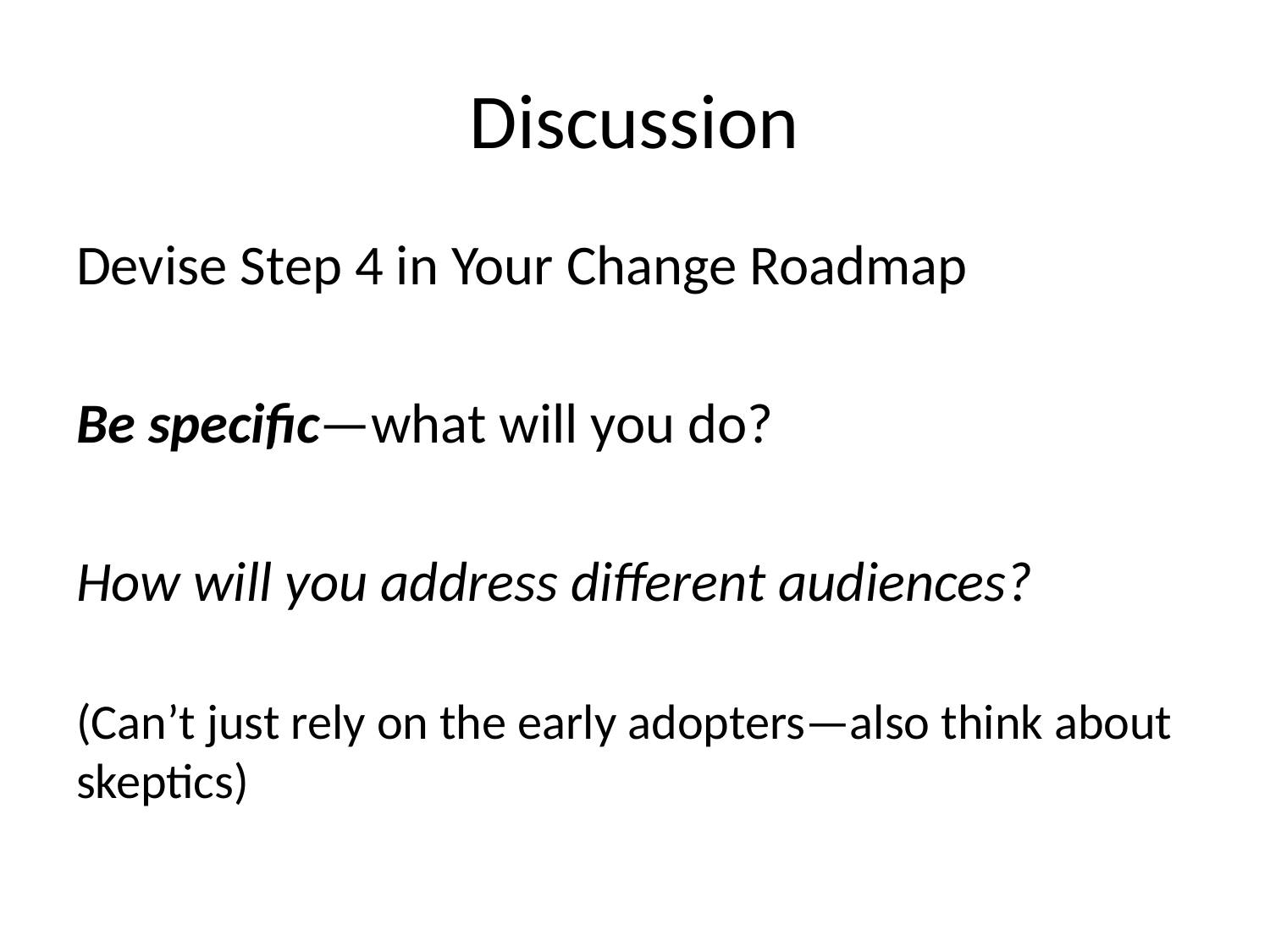

# Discussion
Devise Step 4 in Your Change Roadmap
Be specific—what will you do?
How will you address different audiences?
(Can’t just rely on the early adopters—also think about skeptics)

## Slide 38
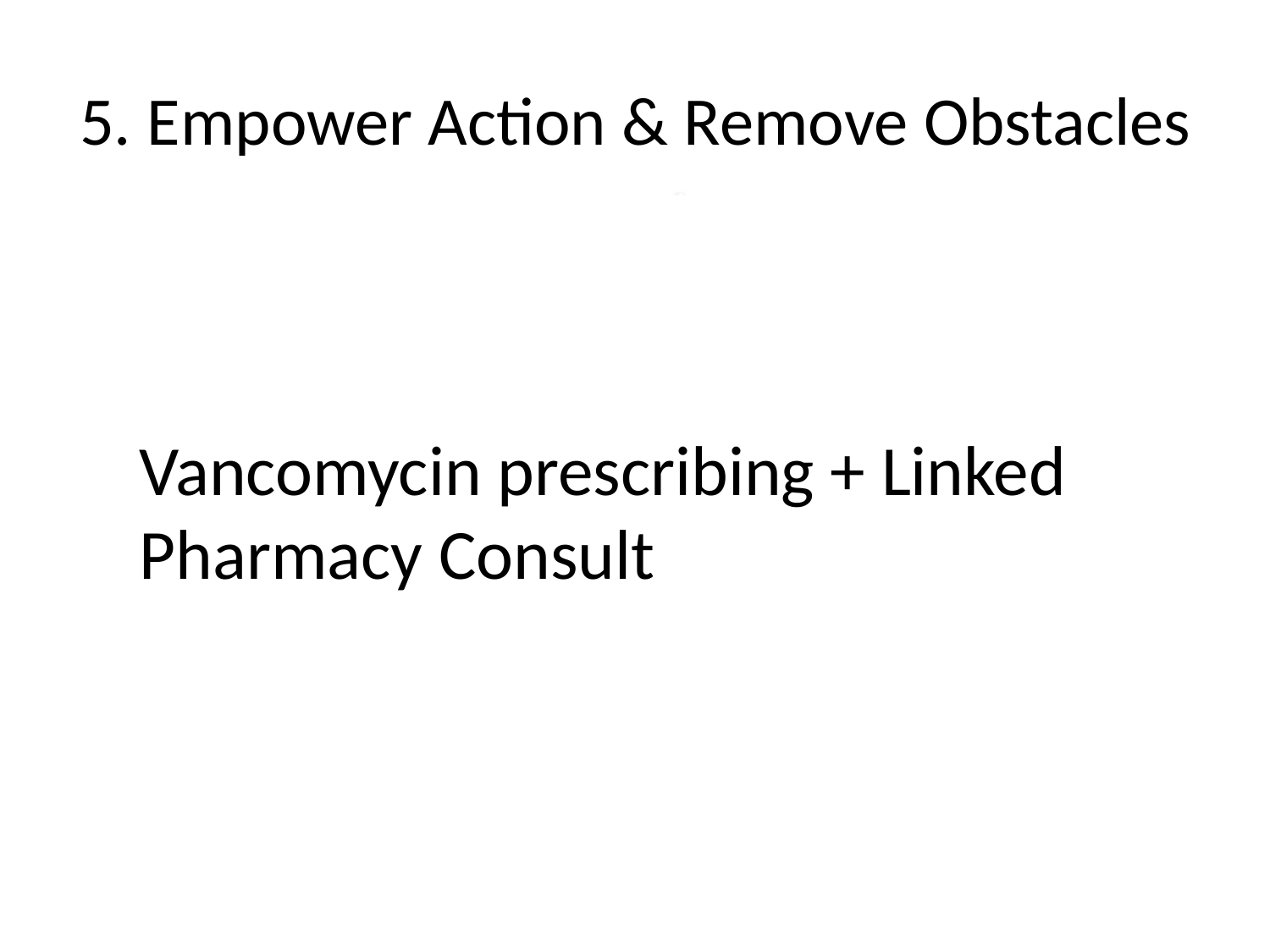

# 5. Empower Action & Remove Obstacles
Vancomycin prescribing + Linked Pharmacy Consult

## Slide 39
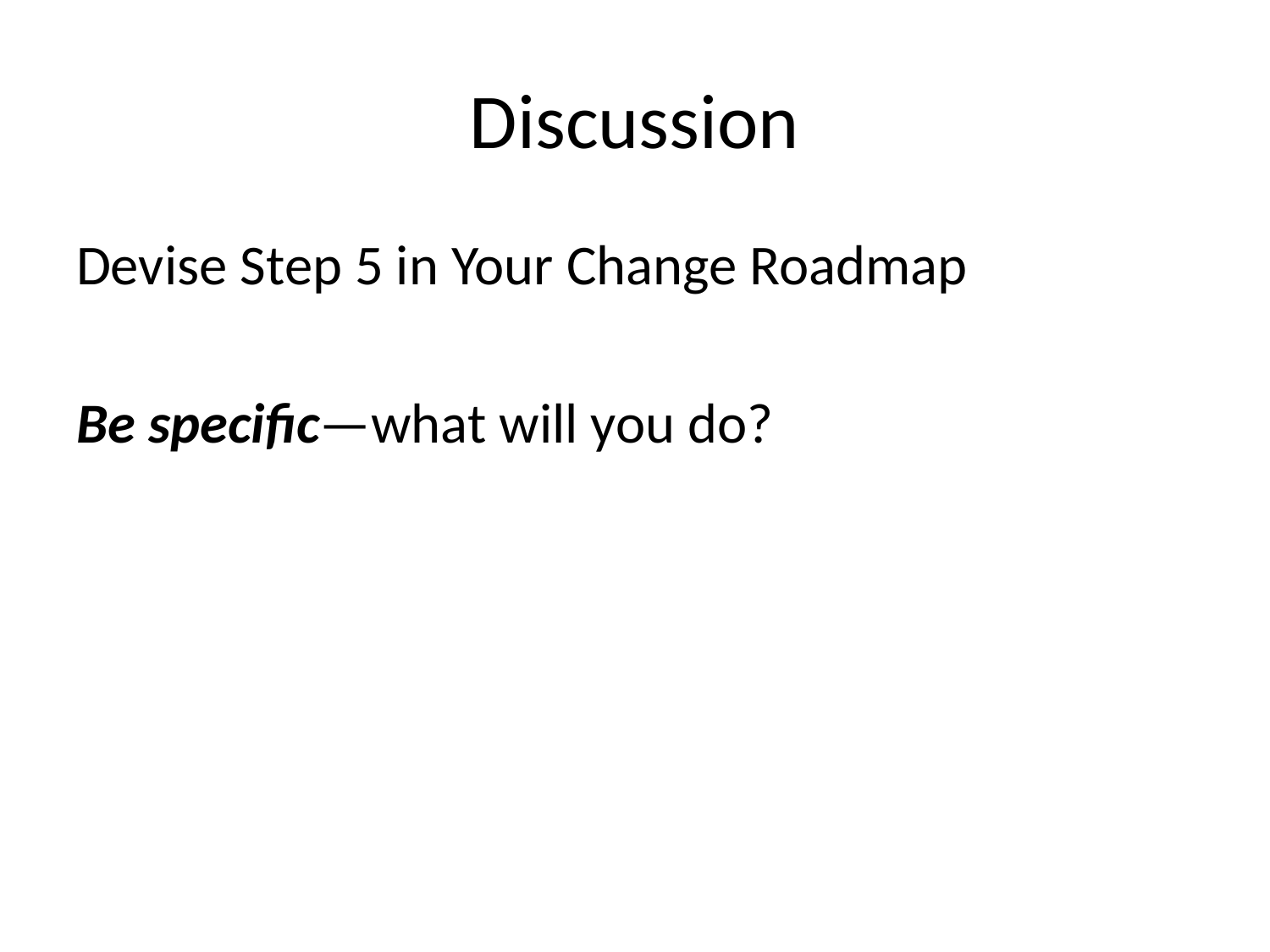

# Discussion
Devise Step 5 in Your Change Roadmap
Be specific—what will you do?

## Slide 40
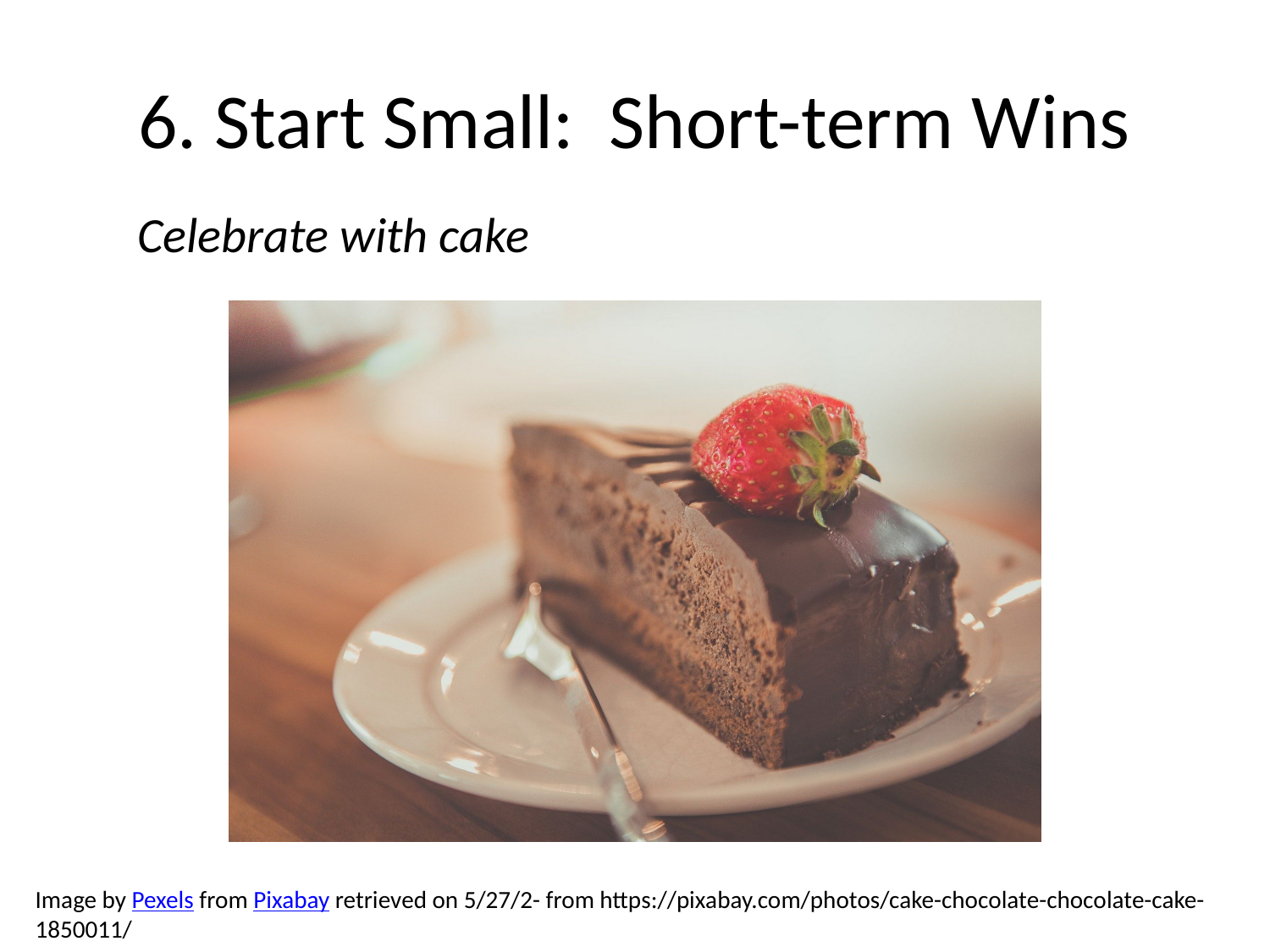

# 6. Start Small: Short-term Wins
Celebrate with cake
Image by Pexels from Pixabay retrieved on 5/27/2- from https://pixabay.com/photos/cake-chocolate-chocolate-cake-1850011/

## Slide 41
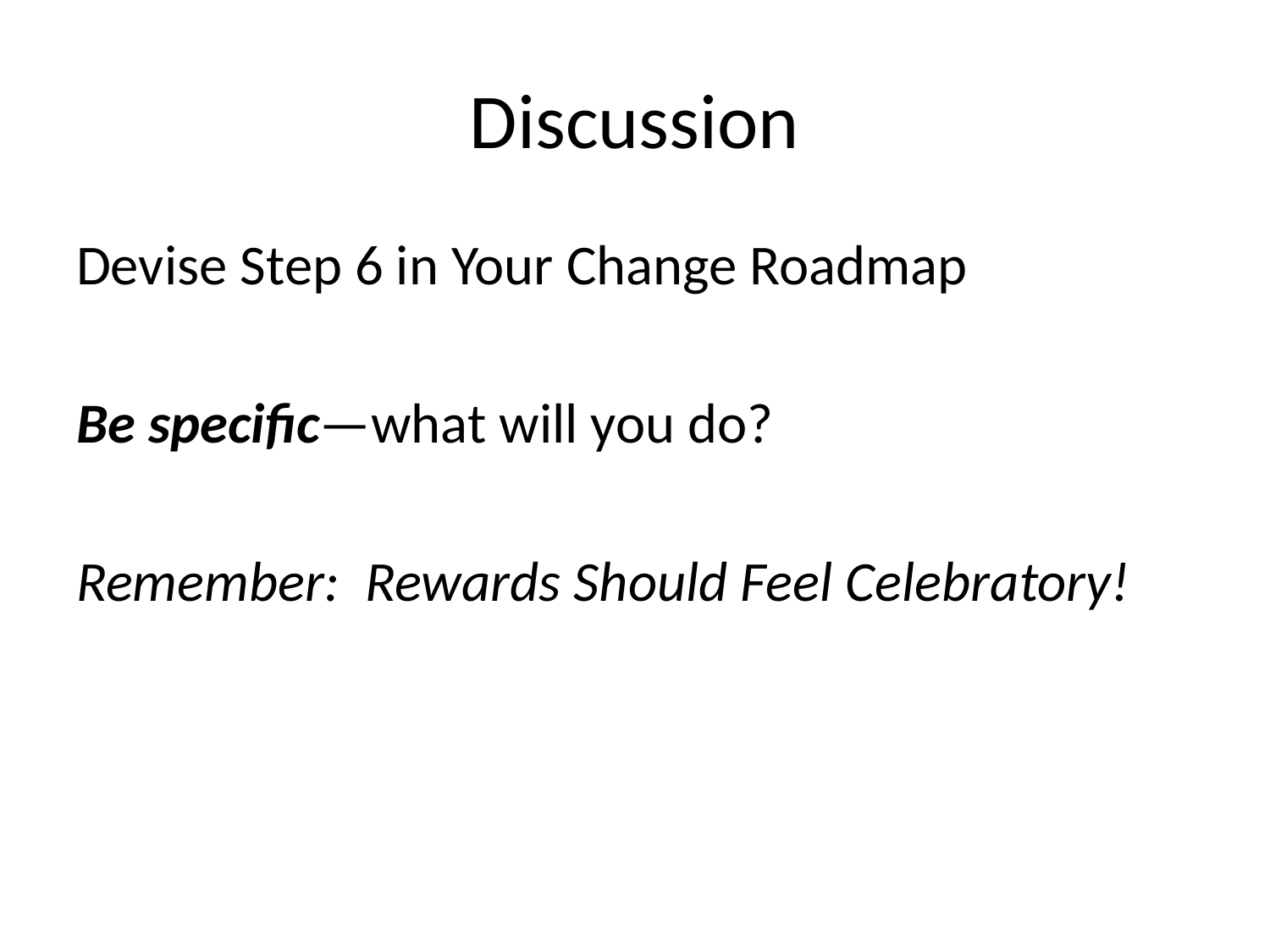

# Discussion
Devise Step 6 in Your Change Roadmap
Be specific—what will you do?
Remember: Rewards Should Feel Celebratory!

## Slide 42
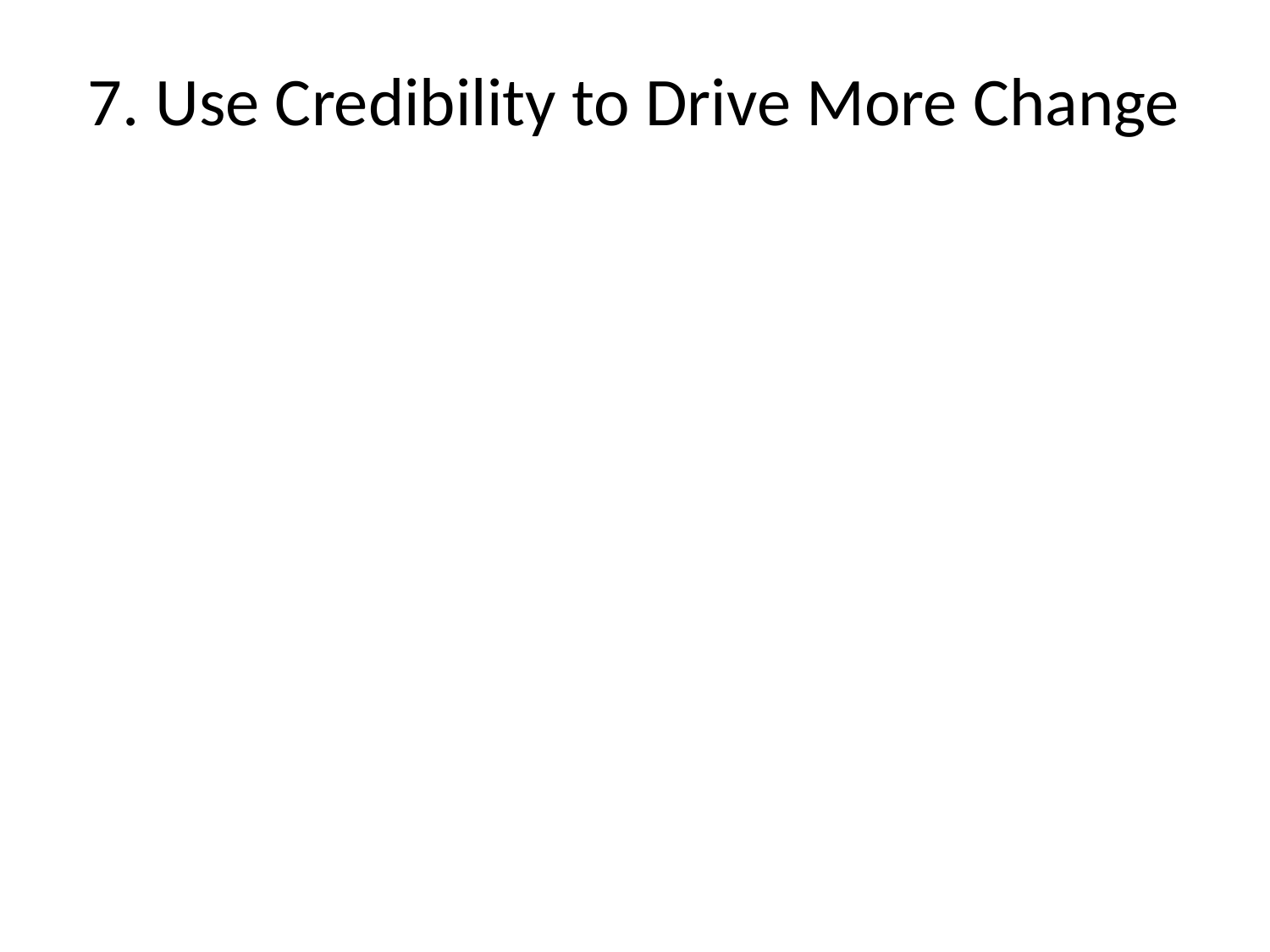

# 7. Use Credibility to Drive More Change

## Slide 43
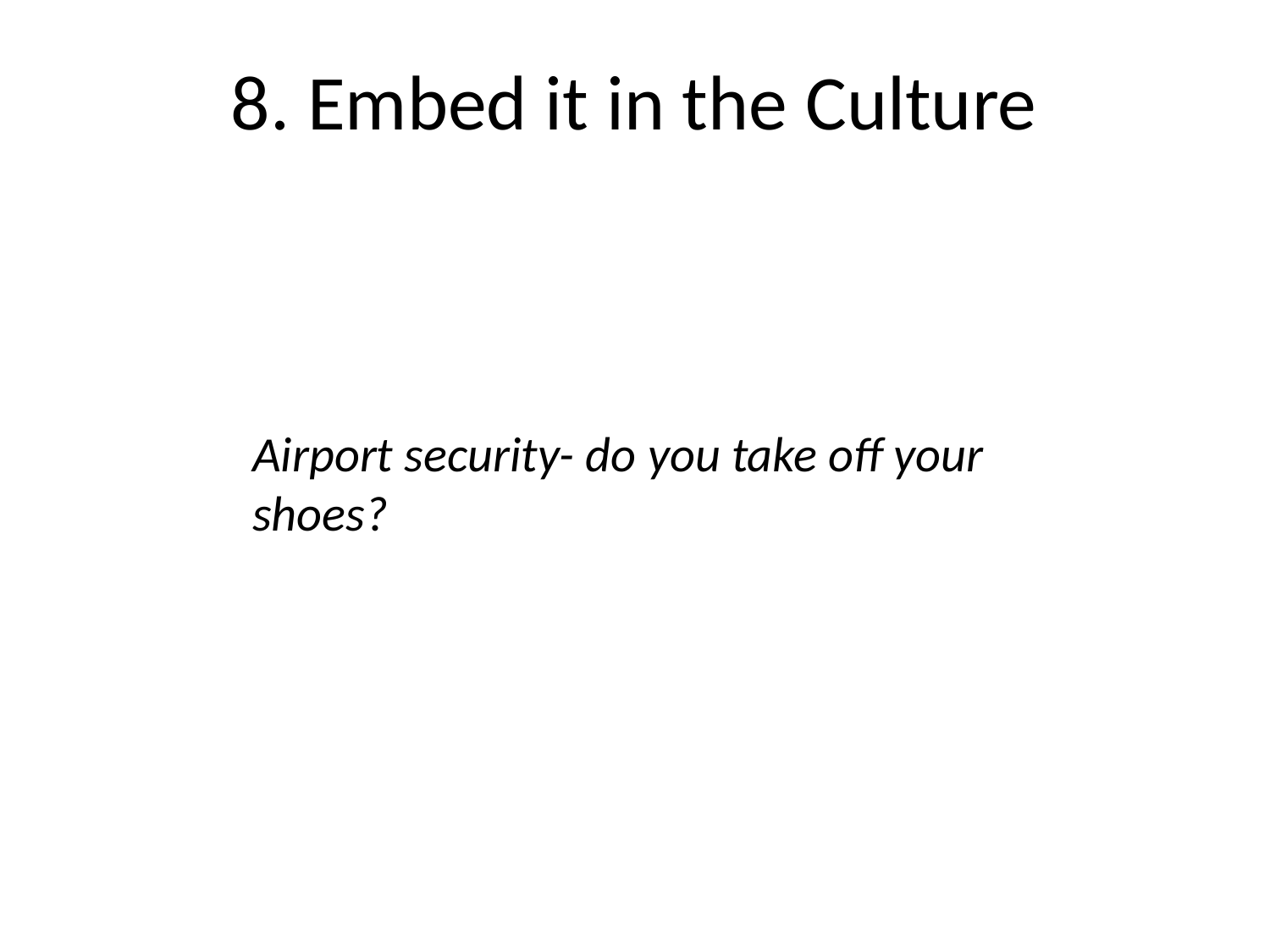

# 8. Embed it in the Culture
Airport security- do you take off your shoes?

## Slide 44
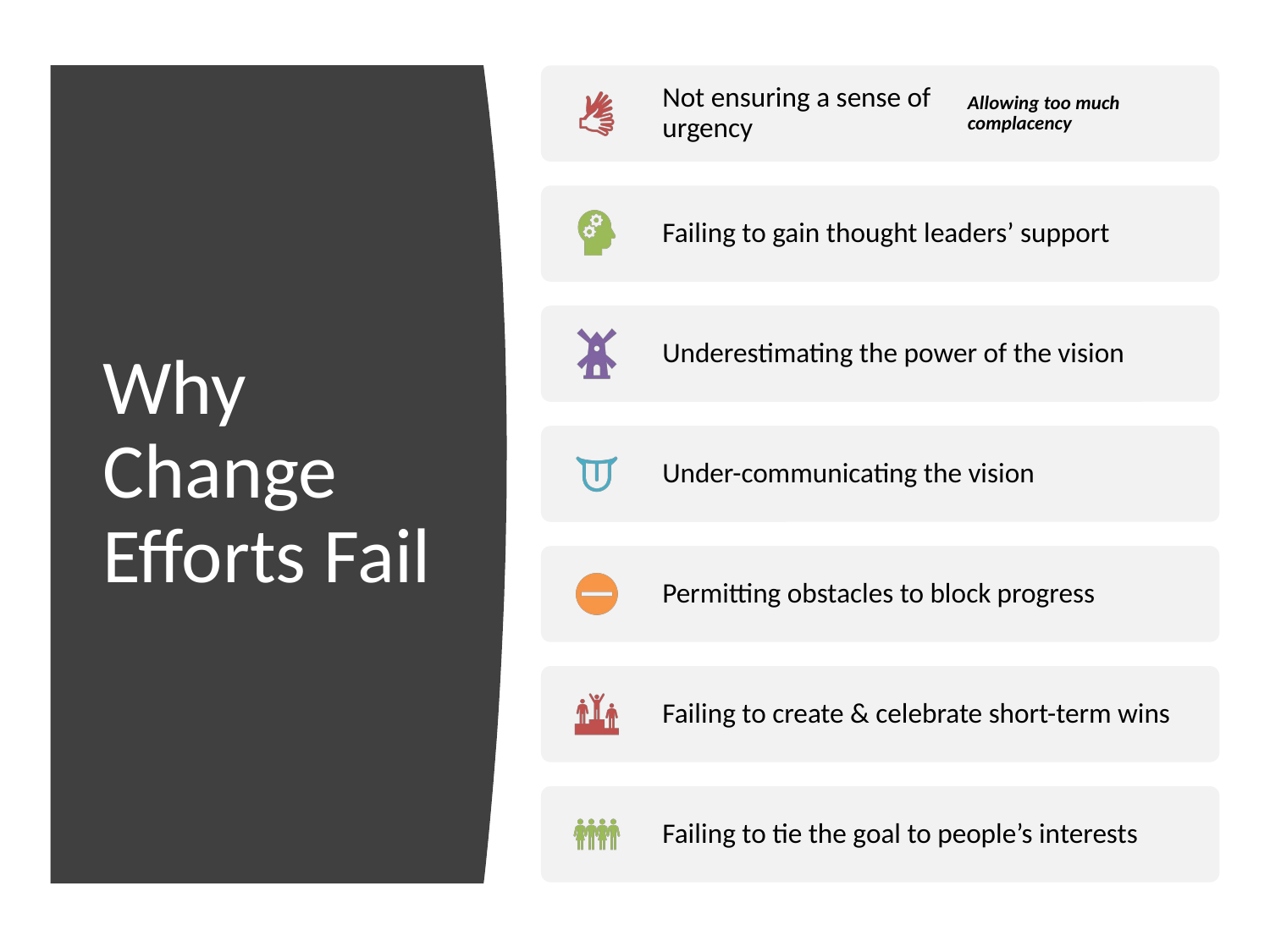

Why Change Efforts Fail

## Slide 45
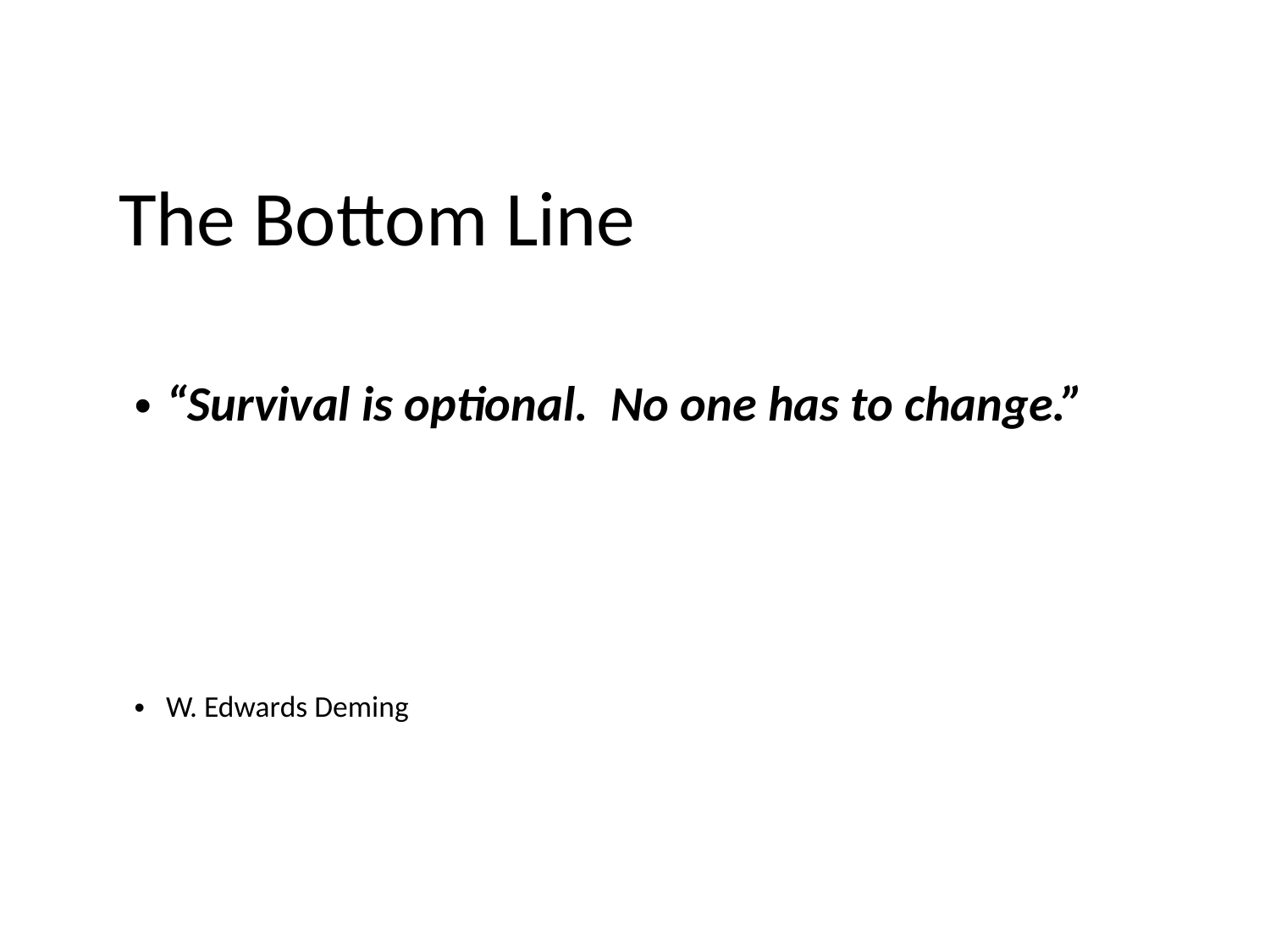

The Bottom Line
“Survival is optional.  No one has to change.”
W. Edwards Deming

## Slide 46
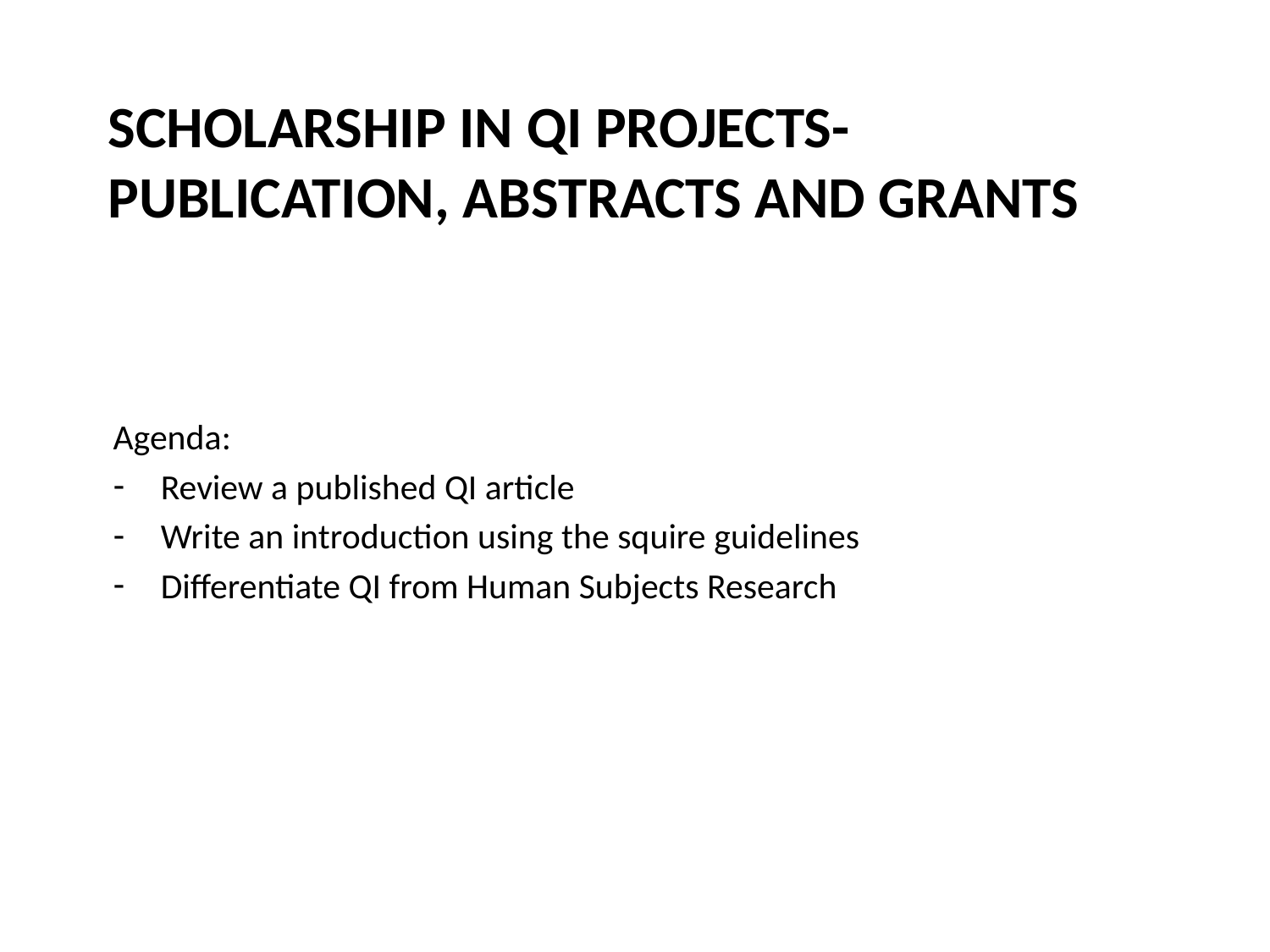

# Scholarship in QI PROJECTS- PUBLICATION, ABSTRACTs and GRANTs
Agenda:
Review a published QI article
Write an introduction using the squire guidelines
Differentiate QI from Human Subjects Research

## Slide 47
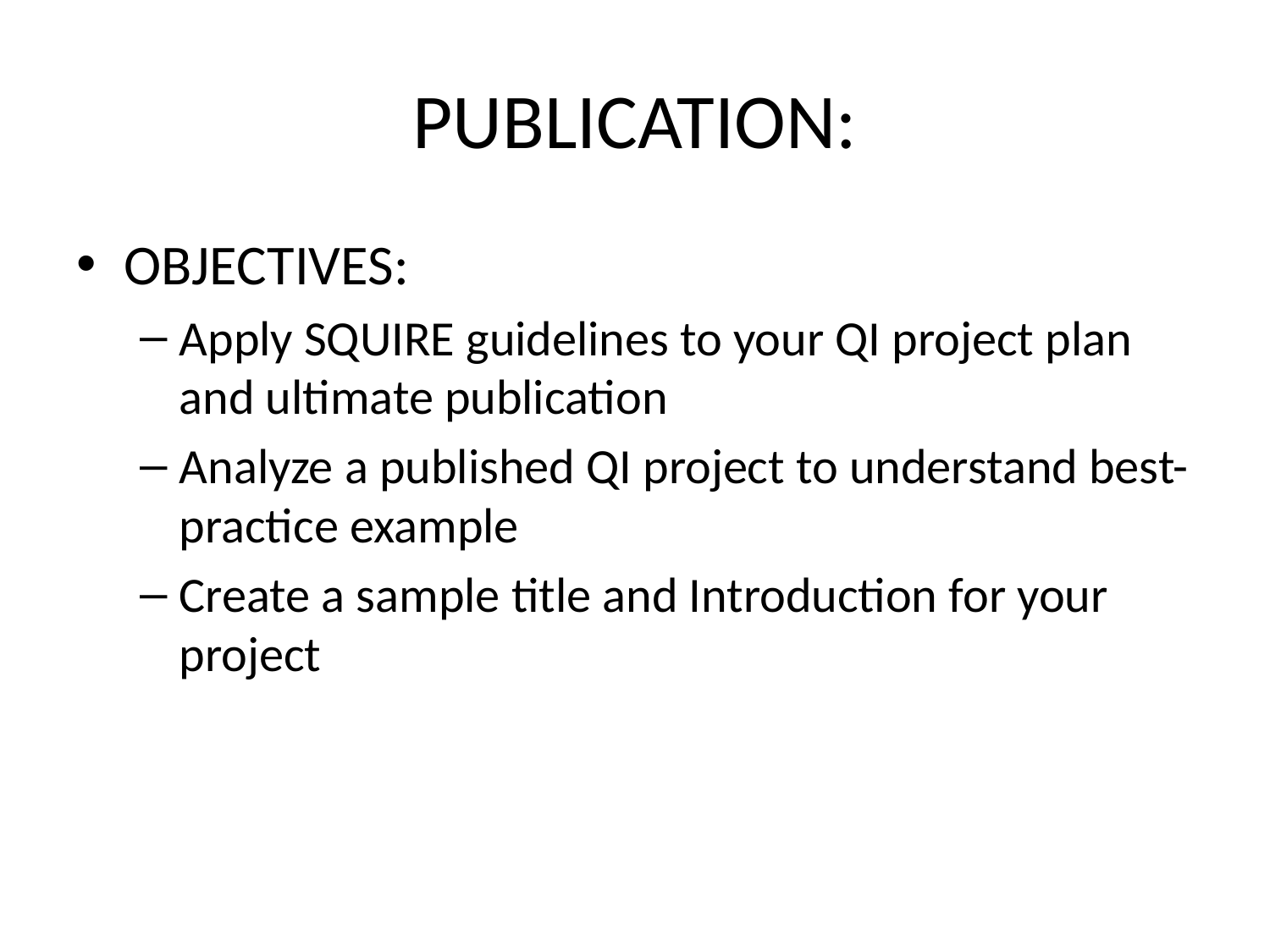

# PUBLICATION:
OBJECTIVES:
Apply SQUIRE guidelines to your QI project plan and ultimate publication
Analyze a published QI project to understand best-practice example
Create a sample title and Introduction for your project

## Slide 48
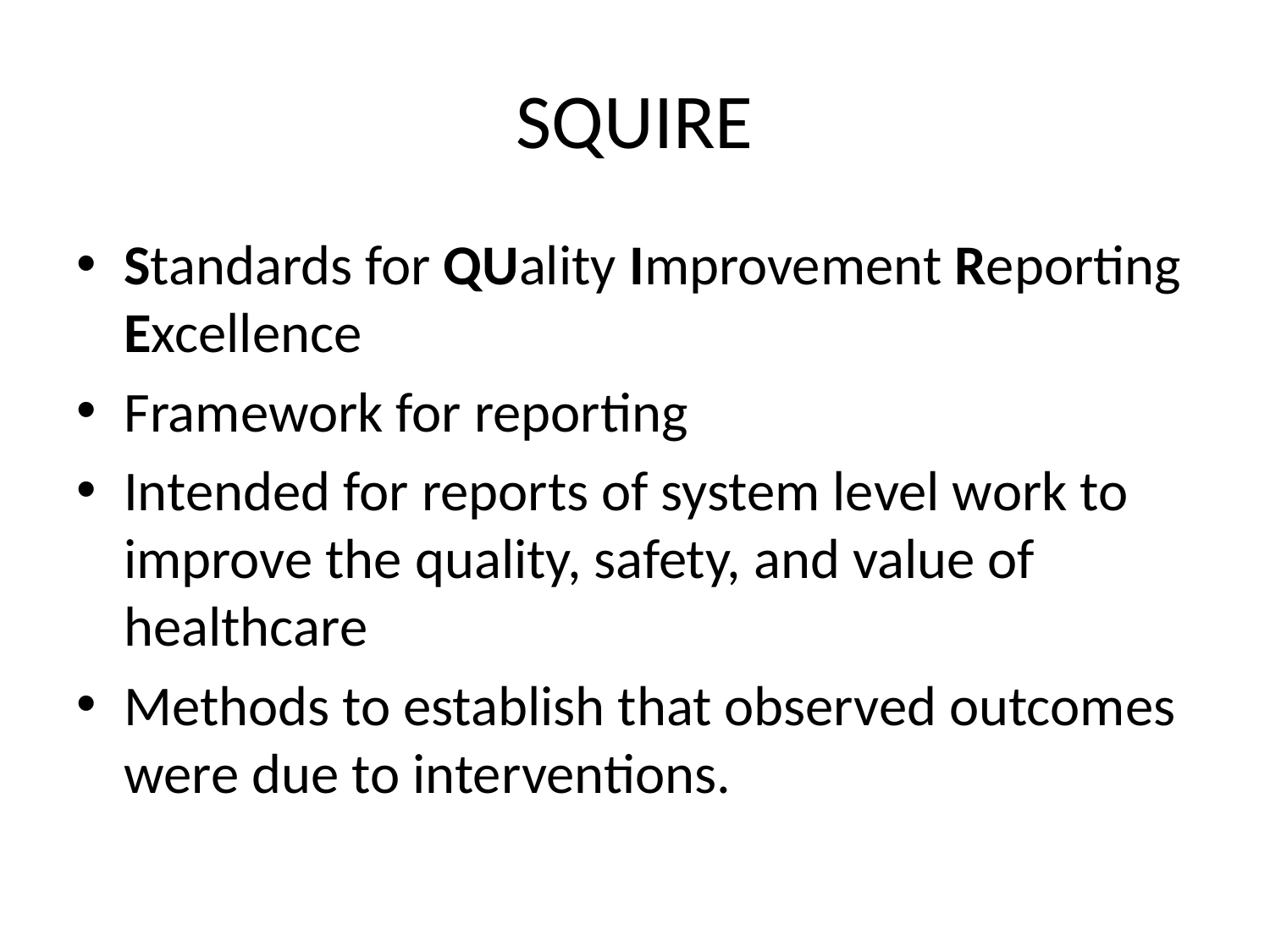

# SQUIRE
Standards for QUality Improvement Reporting Excellence
Framework for reporting
Intended for reports of system level work to improve the quality, safety, and value of healthcare
Methods to establish that observed outcomes were due to interventions.

## Slide 49
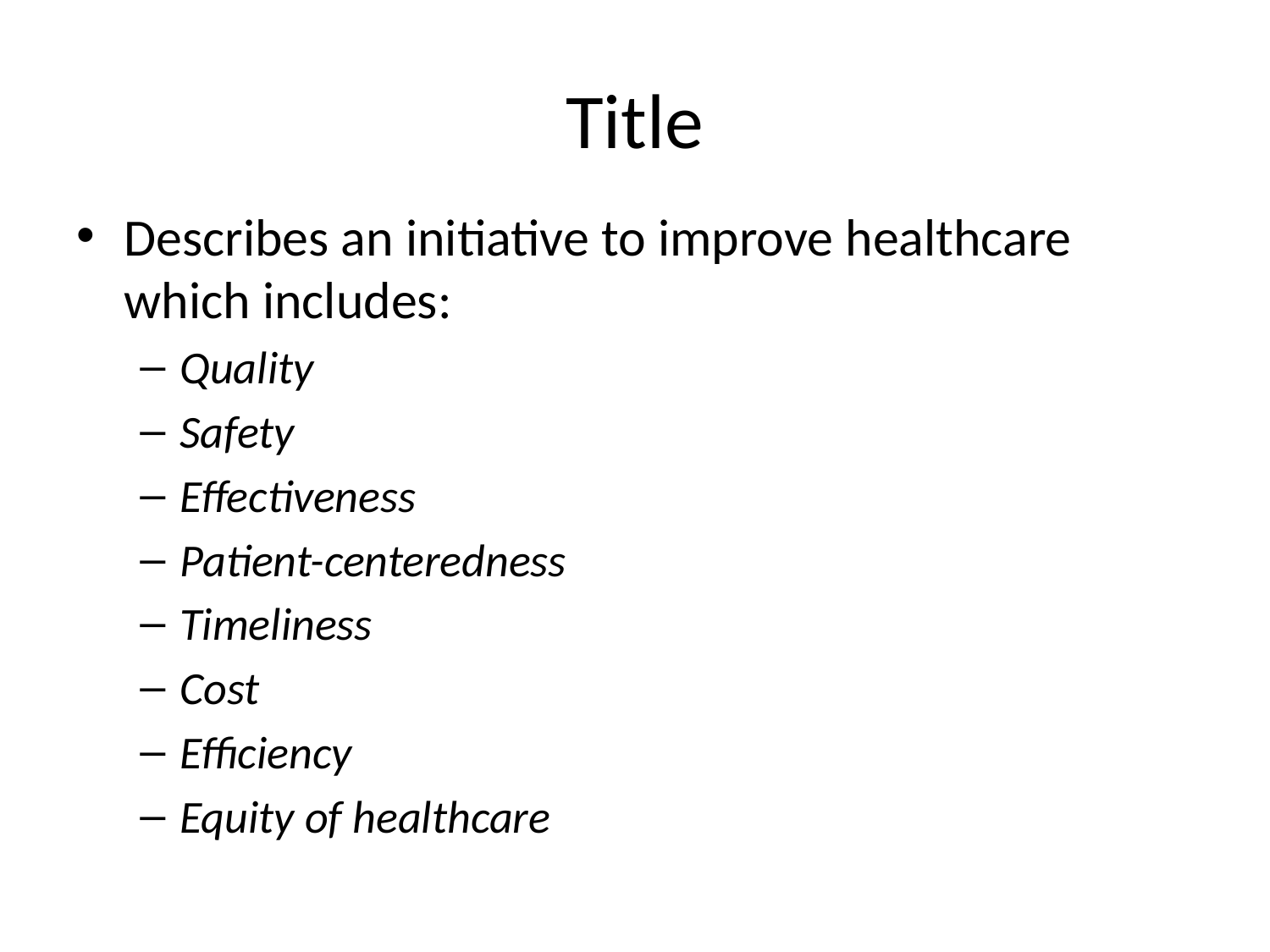

# Title
Describes an initiative to improve healthcare which includes:
Quality
Safety
Effectiveness
Patient-centeredness
Timeliness
Cost
Efficiency
Equity of healthcare

## Slide 50
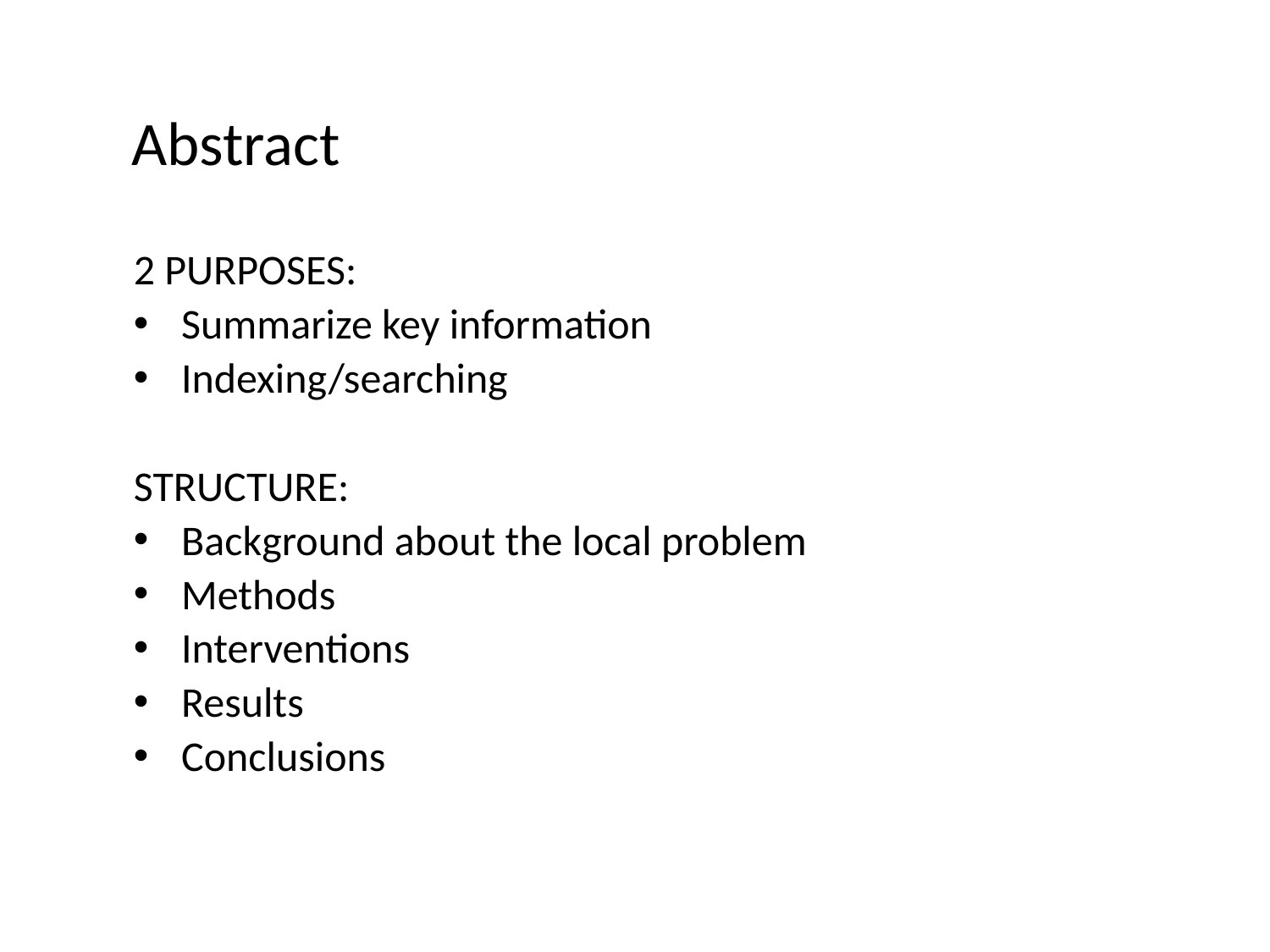

# Abstract
2 PURPOSES:
Summarize key information
Indexing/searching
STRUCTURE:
Background about the local problem
Methods
Interventions
Results
Conclusions

## Slide 51
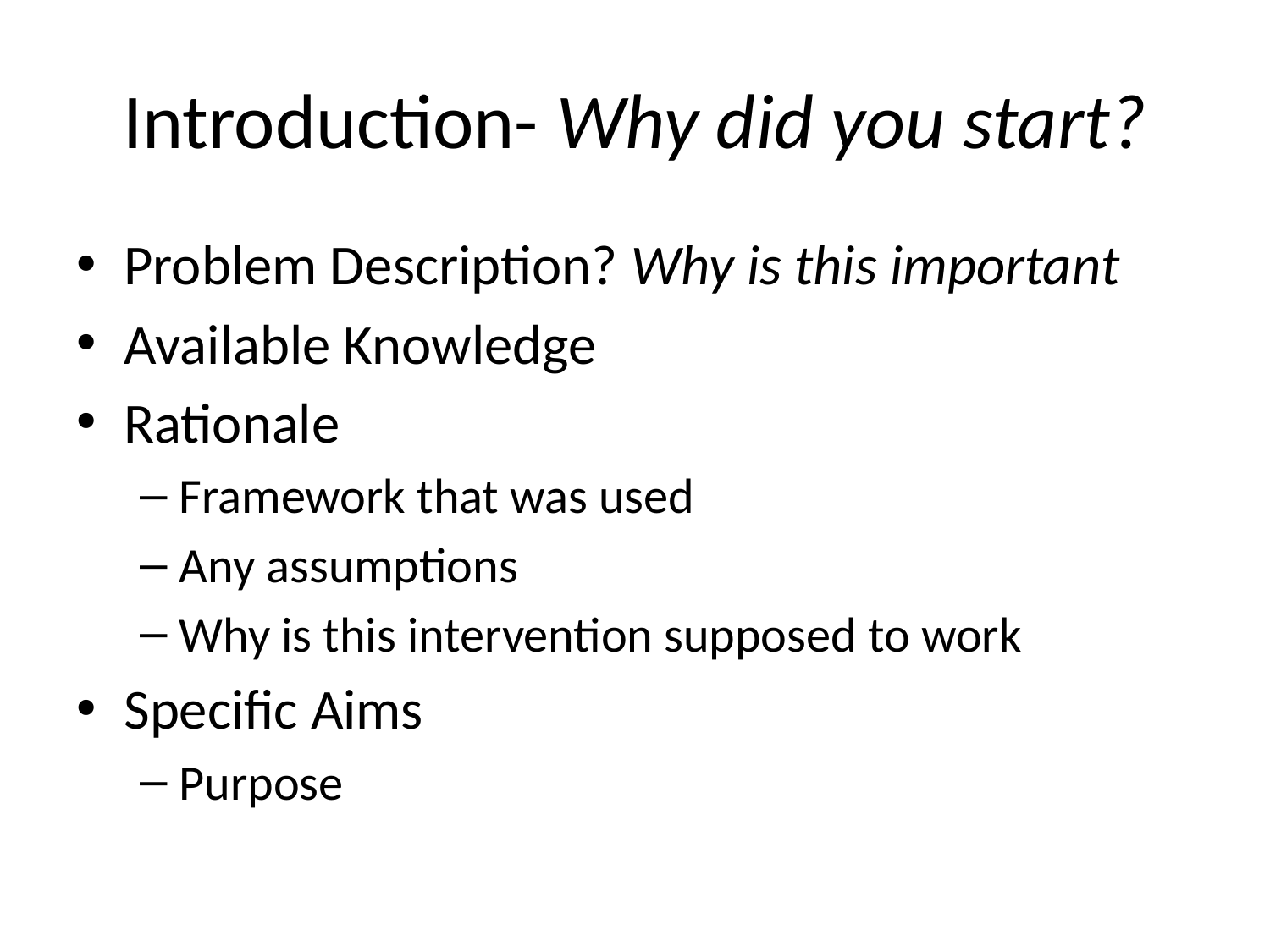

# Introduction- Why did you start?
Problem Description? Why is this important
Available Knowledge
Rationale
Framework that was used
Any assumptions
Why is this intervention supposed to work
Specific Aims
Purpose

## Slide 52
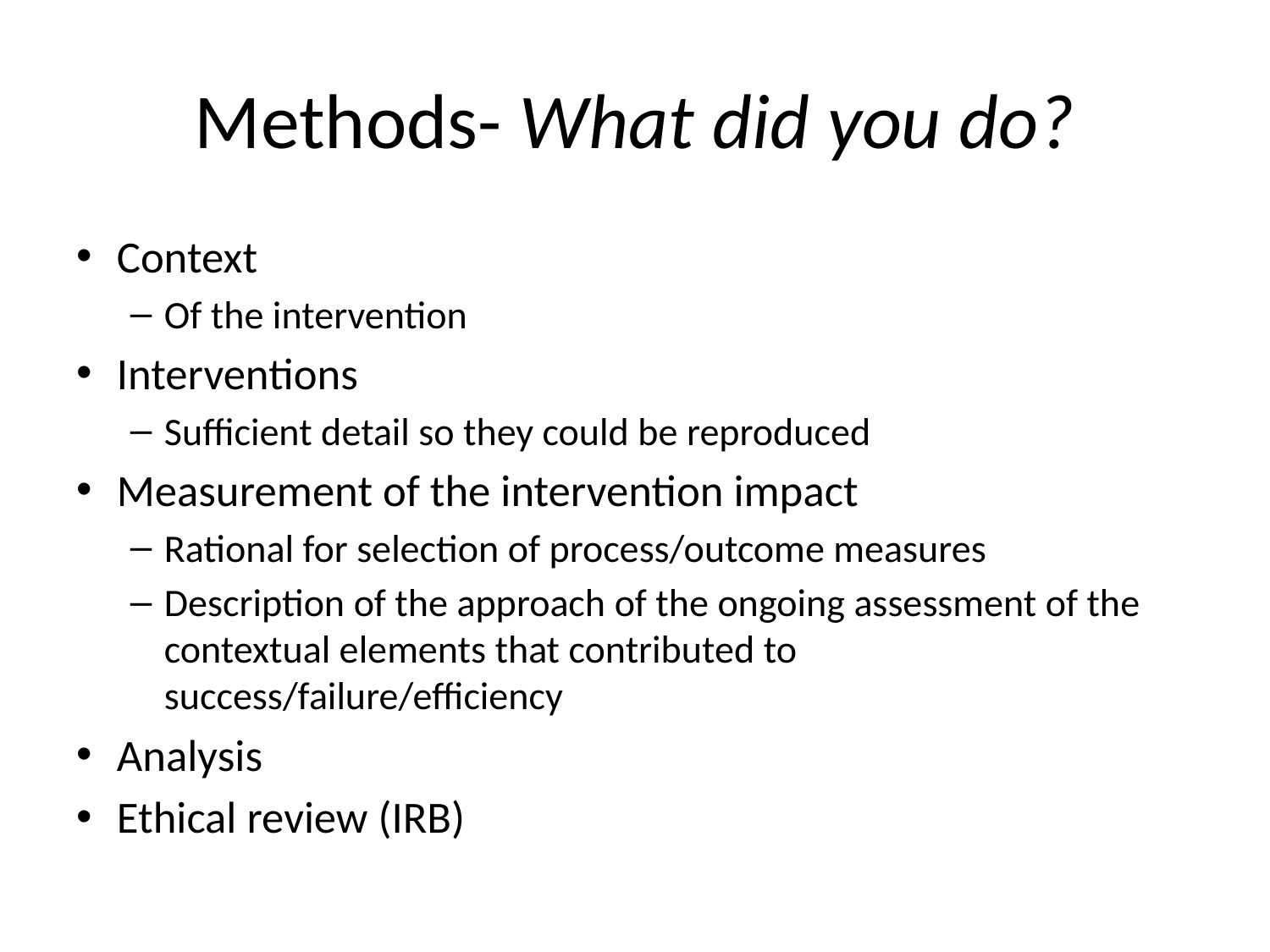

# Methods- What did you do?
Context
Of the intervention
Interventions
Sufficient detail so they could be reproduced
Measurement of the intervention impact
Rational for selection of process/outcome measures
Description of the approach of the ongoing assessment of the contextual elements that contributed to success/failure/efficiency
Analysis
Ethical review (IRB)

## Slide 53
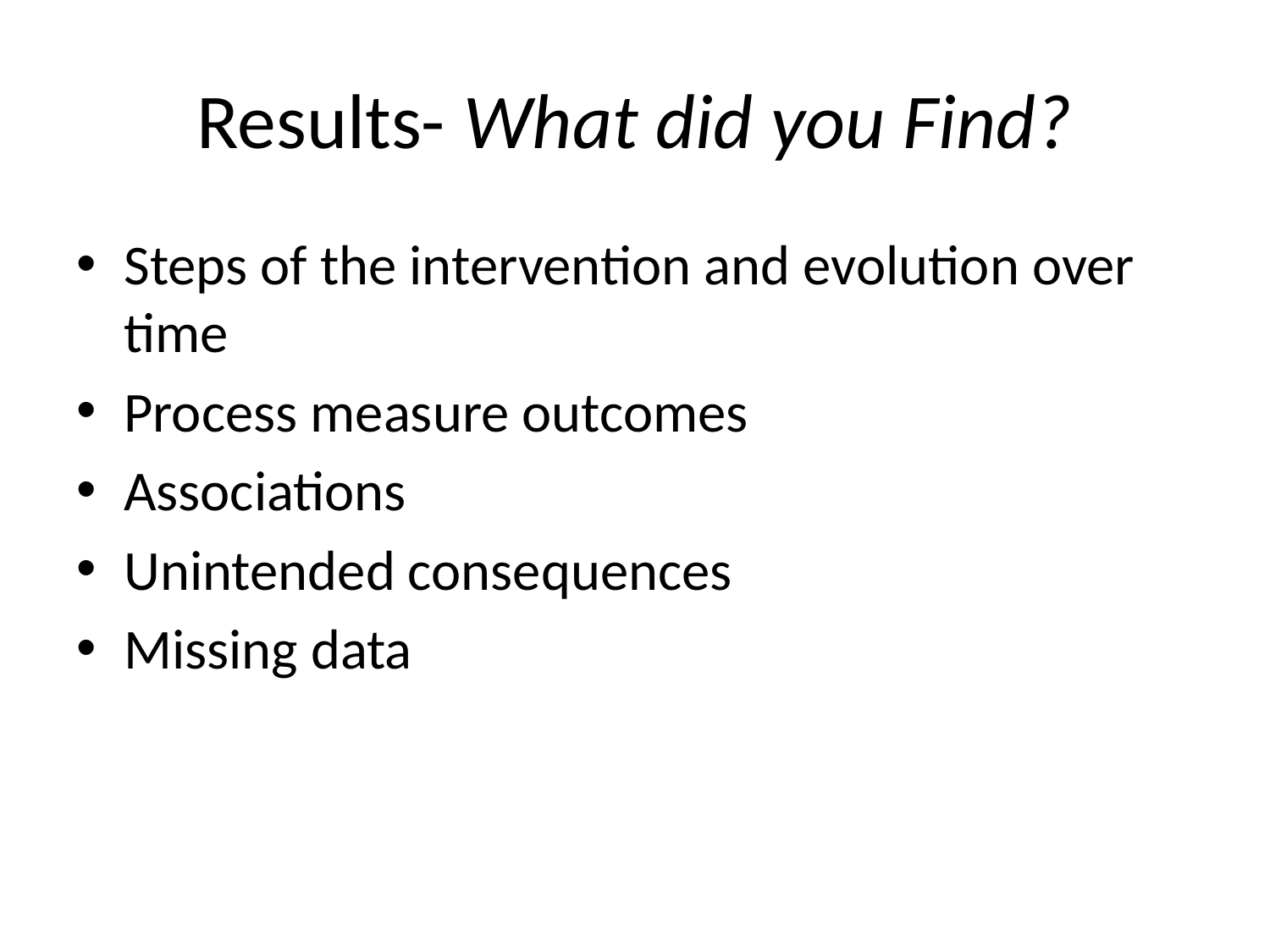

# Results- What did you Find?
Steps of the intervention and evolution over time
Process measure outcomes
Associations
Unintended consequences
Missing data

## Slide 54
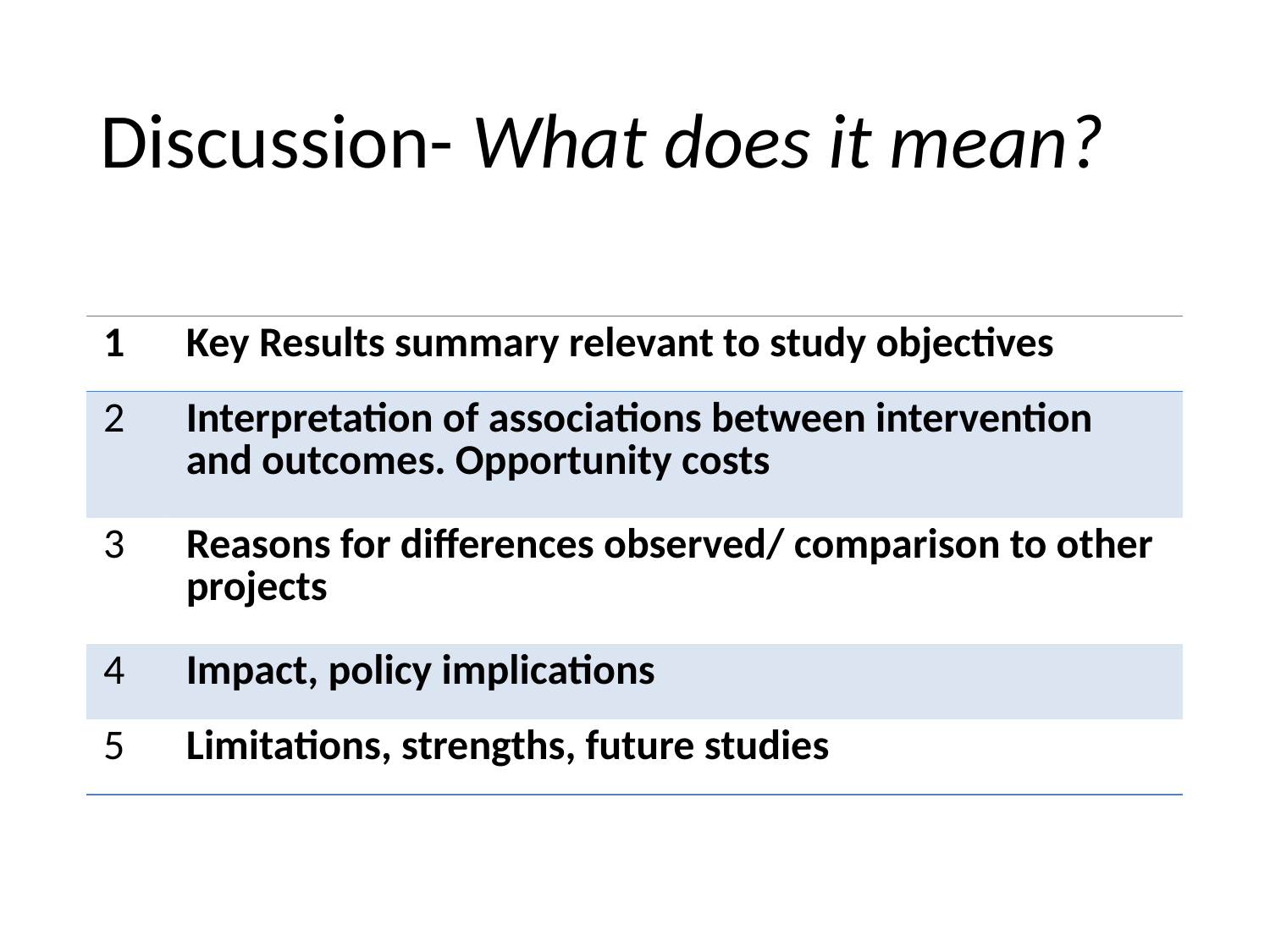

# Discussion- What does it mean?
| 1 | Key Results summary relevant to study objectives |
| --- | --- |
| 2 | Interpretation of associations between intervention and outcomes. Opportunity costs |
| 3 | Reasons for differences observed/ comparison to other projects |
| 4 | Impact, policy implications |
| 5 | Limitations, strengths, future studies |

## Slide 55
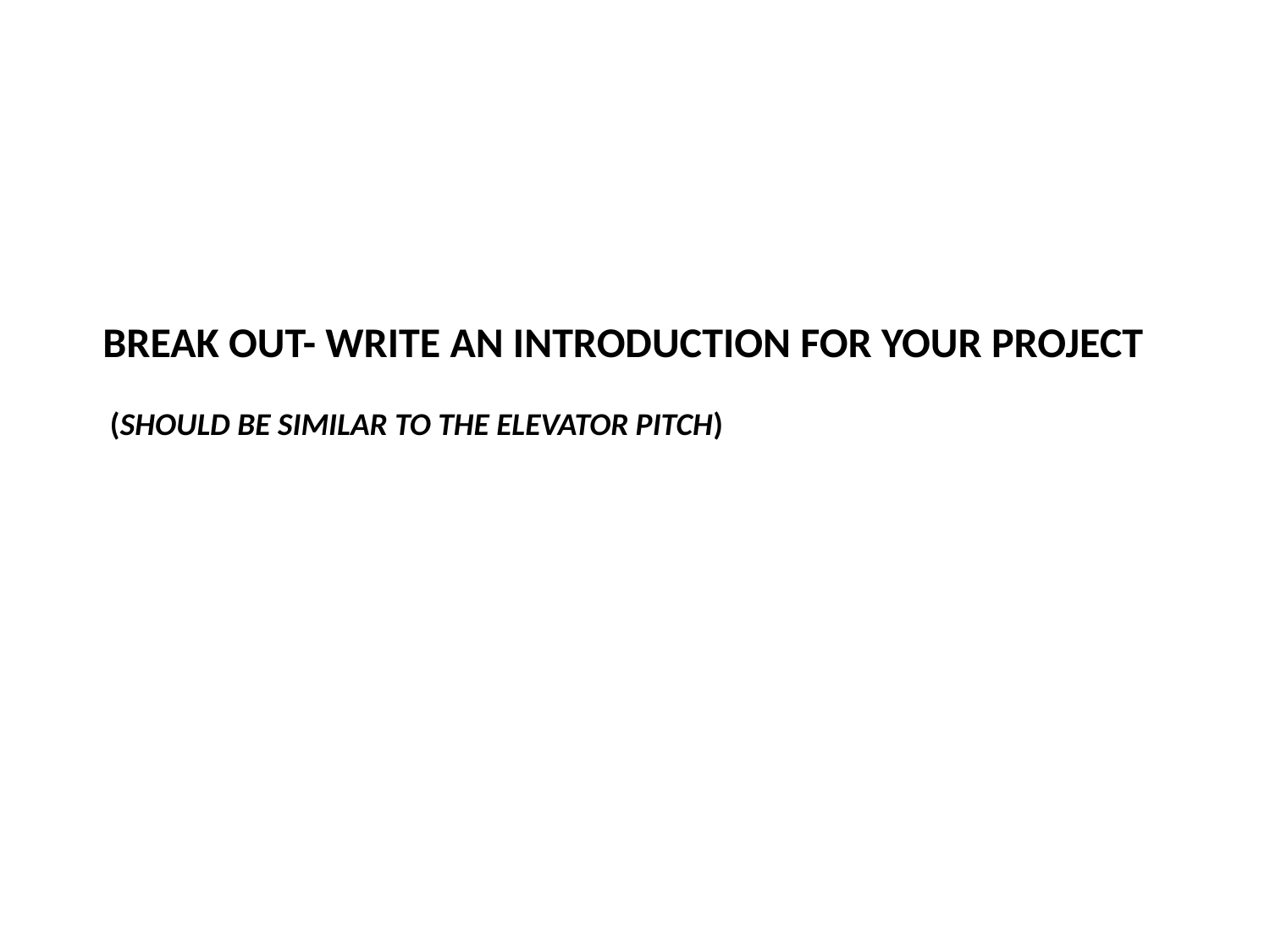

# Break Out- Write an Introduction for your project (should be similar to the elevator pitch)

## Slide 56
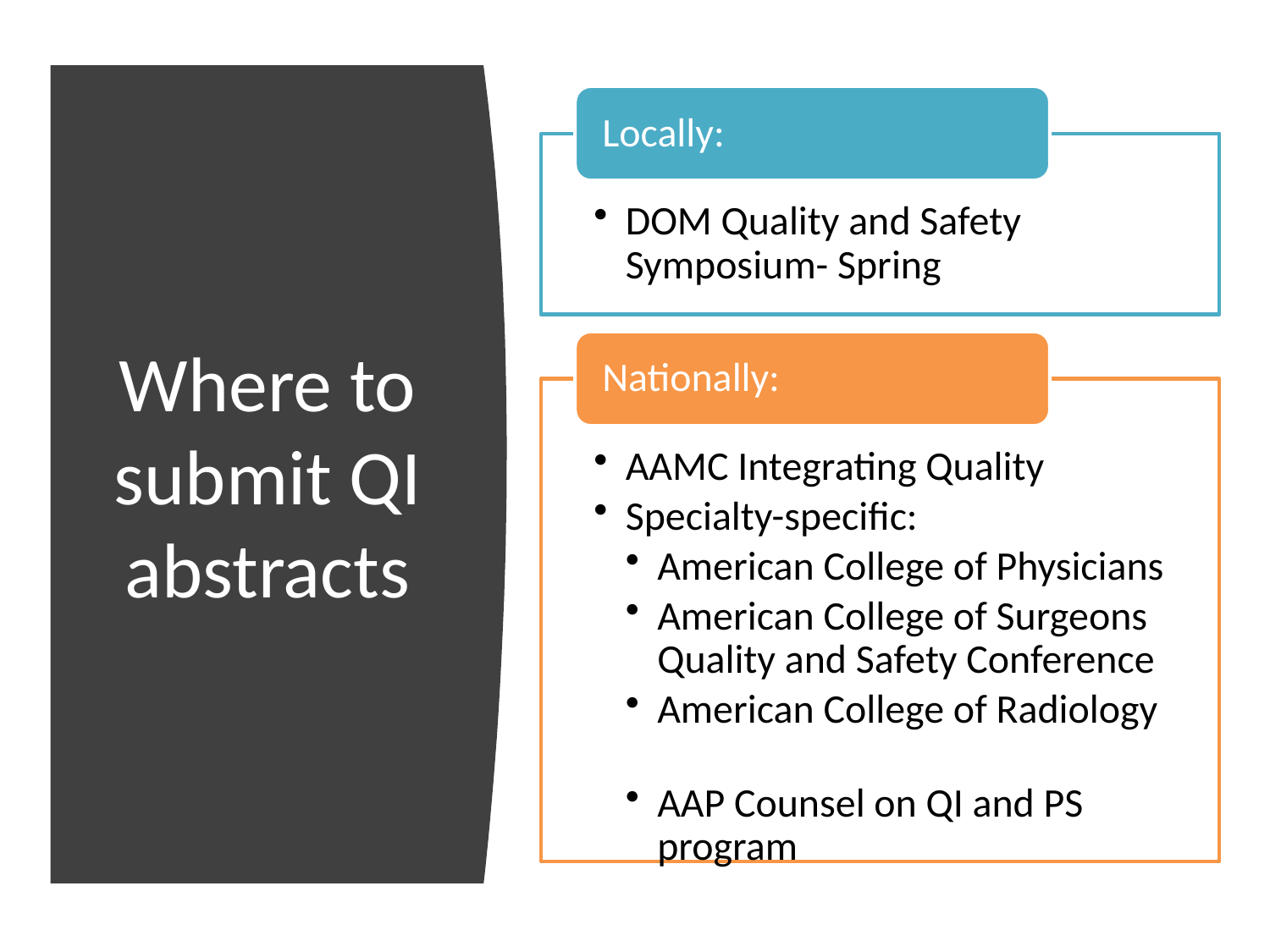

# Where to submit QI abstracts

## Slide 57
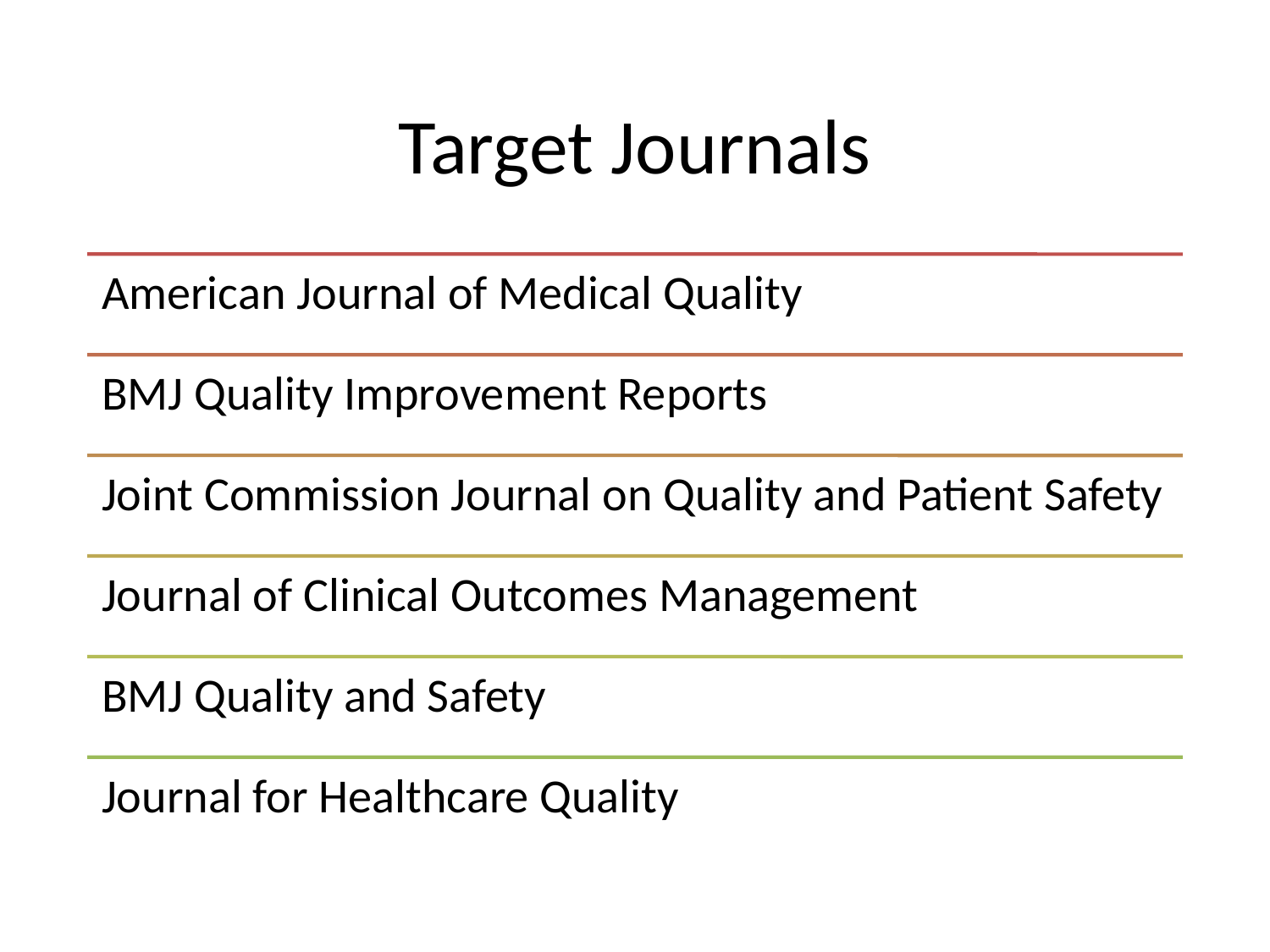

# Target Journals

## Slide 58
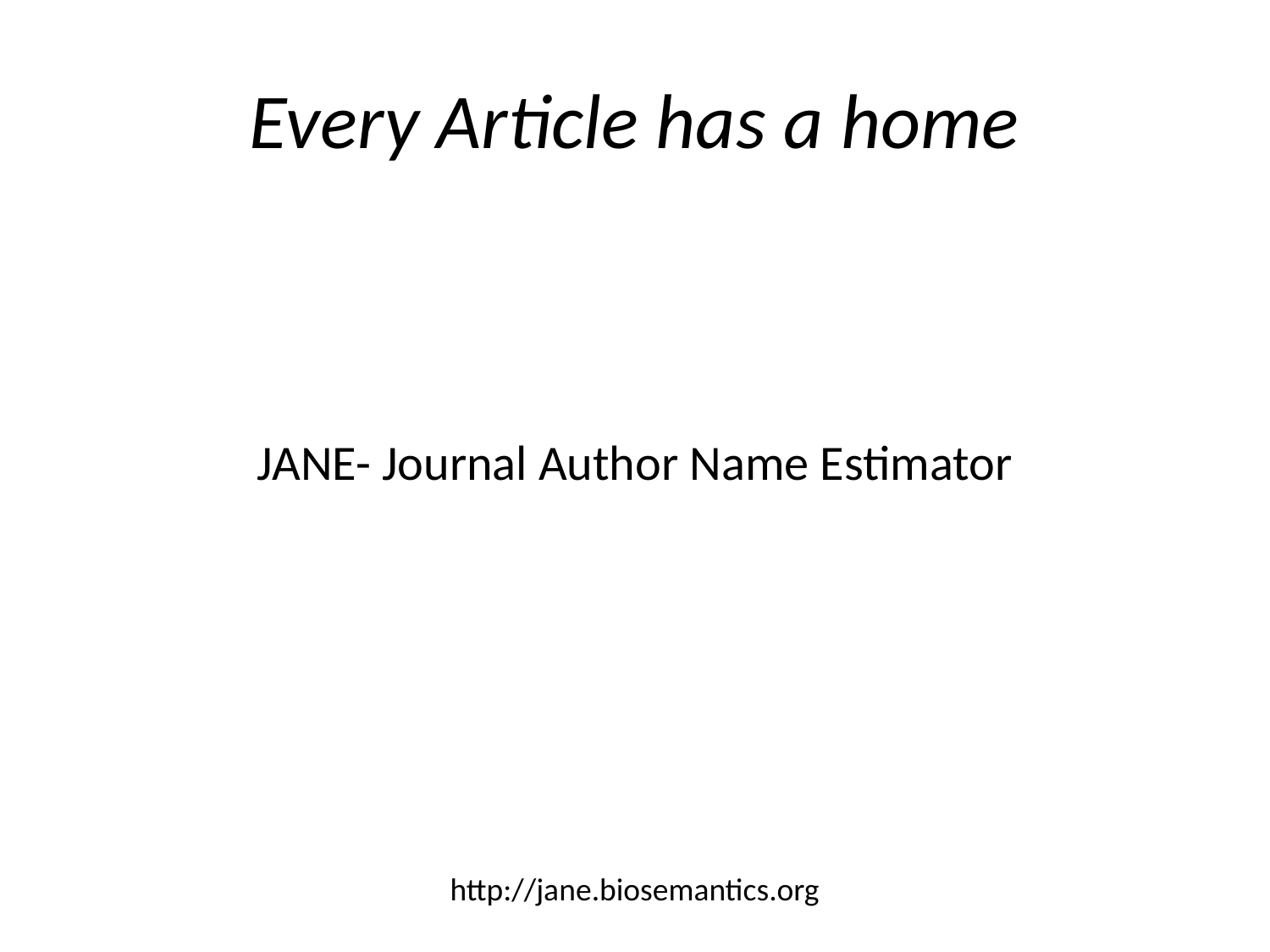

# Every Article has a home
JANE- Journal Author Name Estimator
http://jane.biosemantics.org

## Slide 59
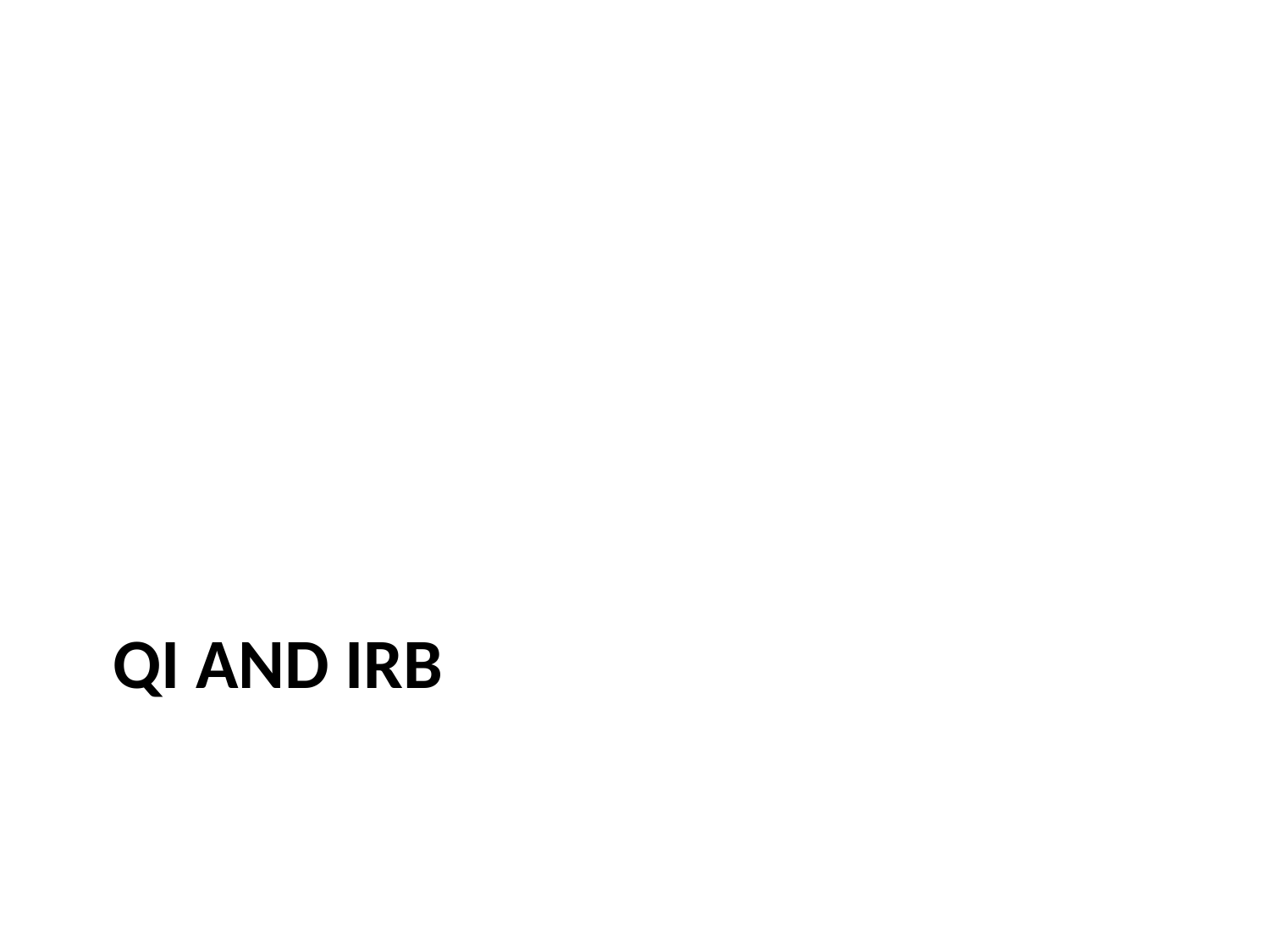

# QI and IRB

## Slide 60
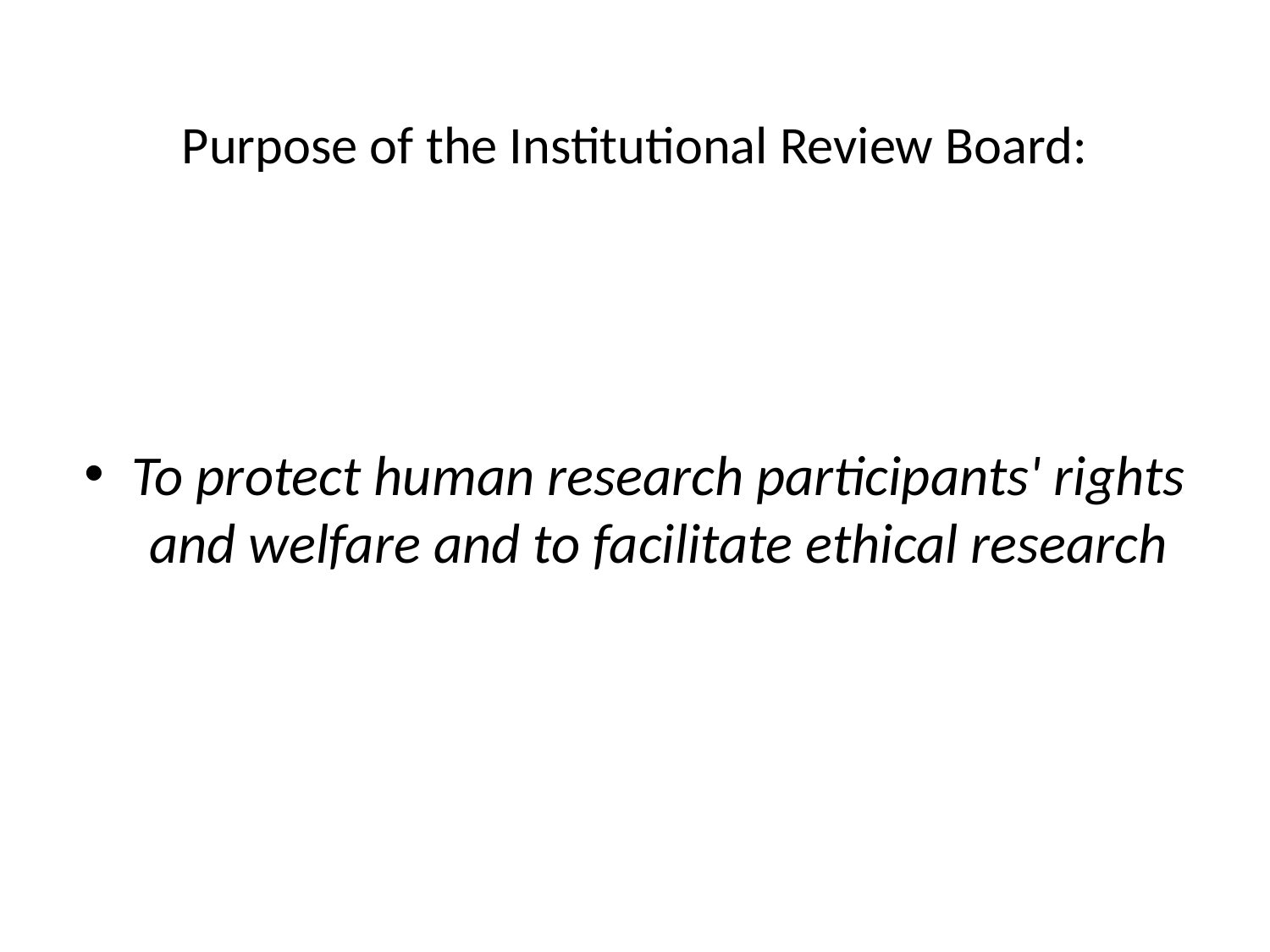

# Purpose of the Institutional Review Board:
To protect human research participants' rights and welfare and to facilitate ethical research

## Slide 61
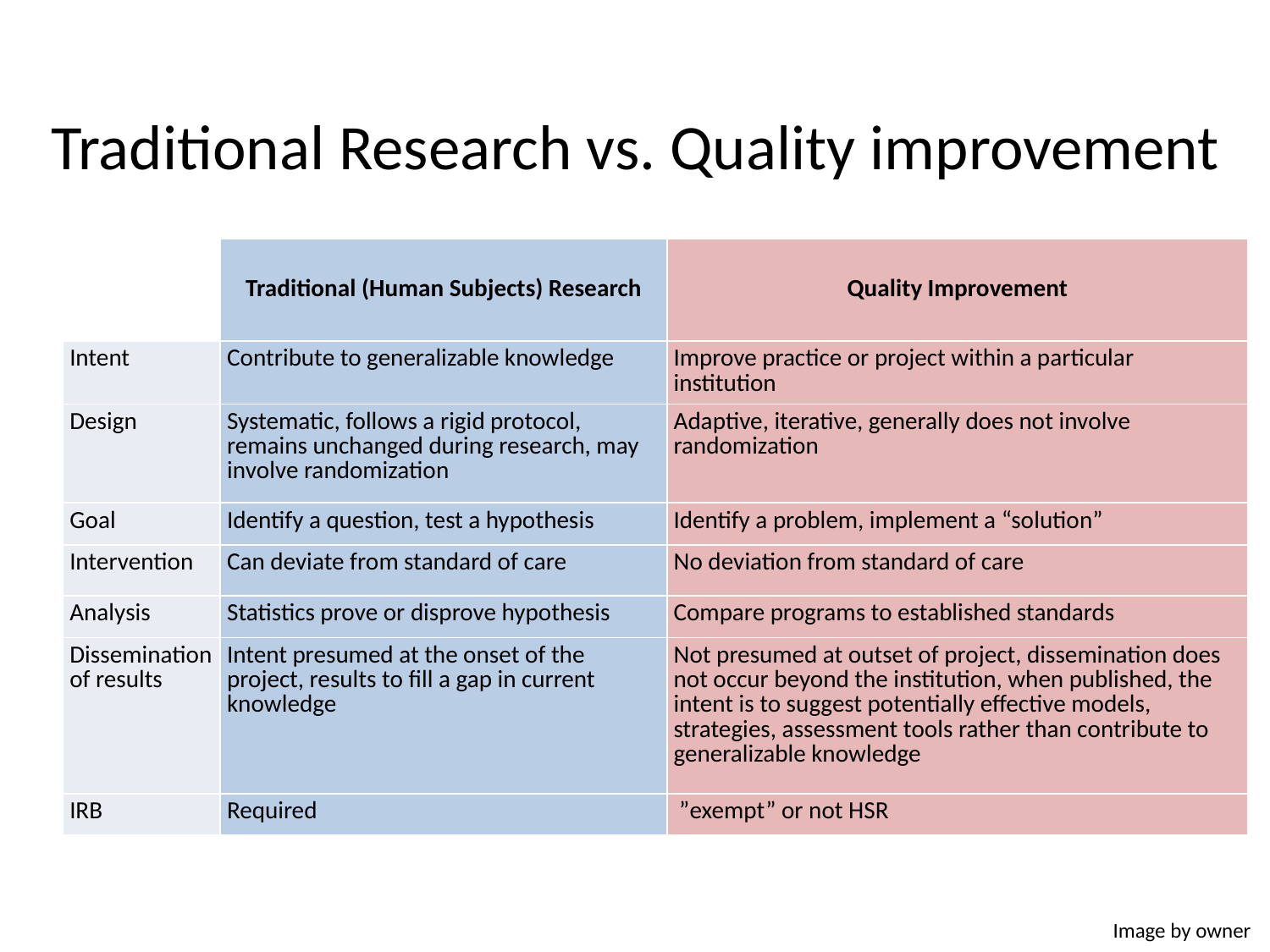

# Traditional Research vs. Quality improvement
| | Traditional (Human Subjects) Research | Quality Improvement |
| --- | --- | --- |
| Intent | Contribute to generalizable knowledge | Improve practice or project within a particular institution |
| Design | Systematic, follows a rigid protocol, remains unchanged during research, may involve randomization | Adaptive, iterative, generally does not involve randomization |
| Goal | Identify a question, test a hypothesis | Identify a problem, implement a “solution” |
| Intervention | Can deviate from standard of care | No deviation from standard of care |
| Analysis | Statistics prove or disprove hypothesis | Compare programs to established standards |
| Dissemination of results | Intent presumed at the onset of the project, results to fill a gap in current knowledge | Not presumed at outset of project, dissemination does not occur beyond the institution, when published, the intent is to suggest potentially effective models, strategies, assessment tools rather than contribute to generalizable knowledge |
| IRB | Required | ”exempt” or not HSR |
Image by owner

## Slide 62
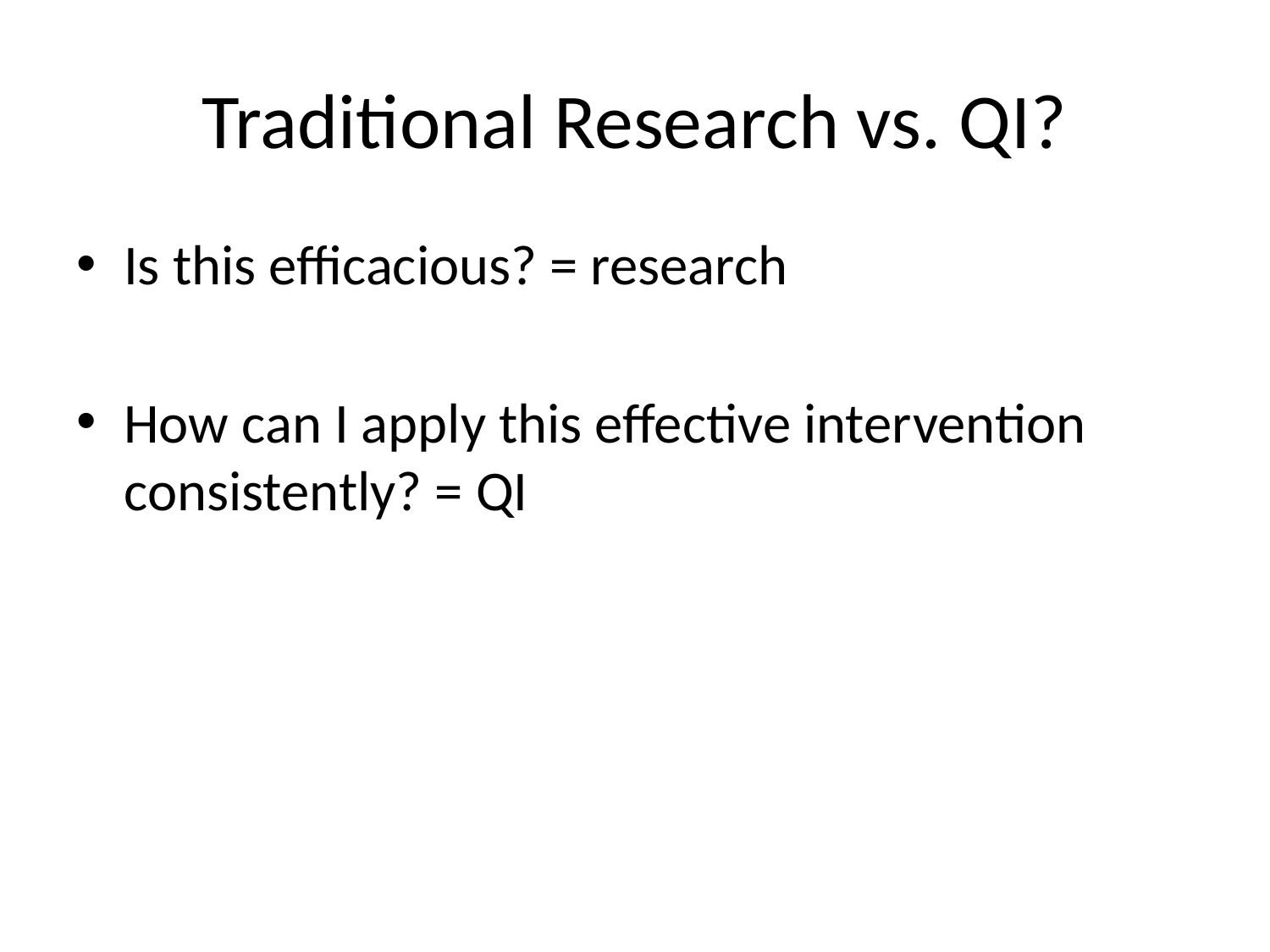

# Traditional Research vs. QI?
Is this efficacious? = research
How can I apply this effective intervention consistently? = QI

## Slide 63
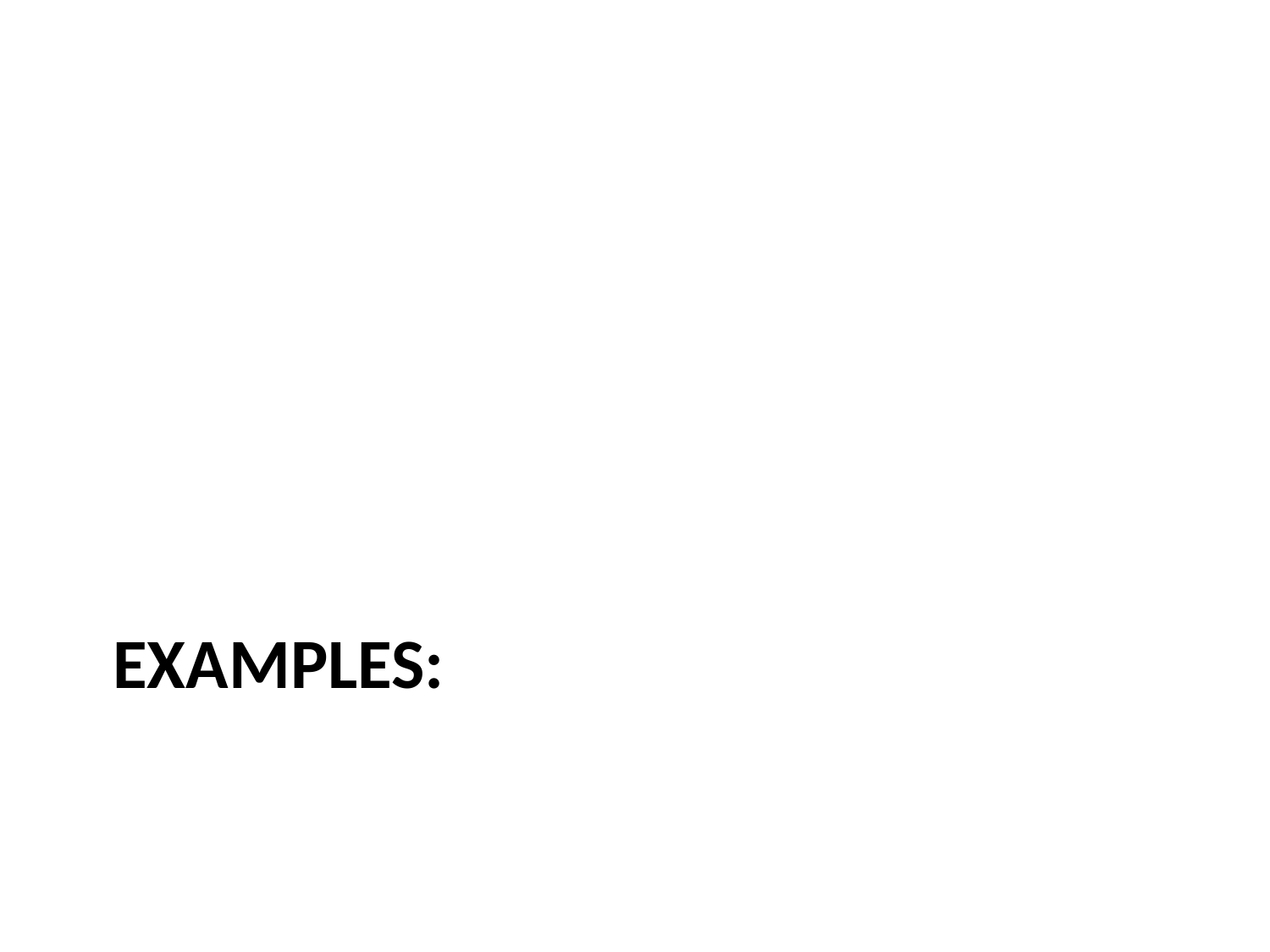

# Examples:

## Slide 64
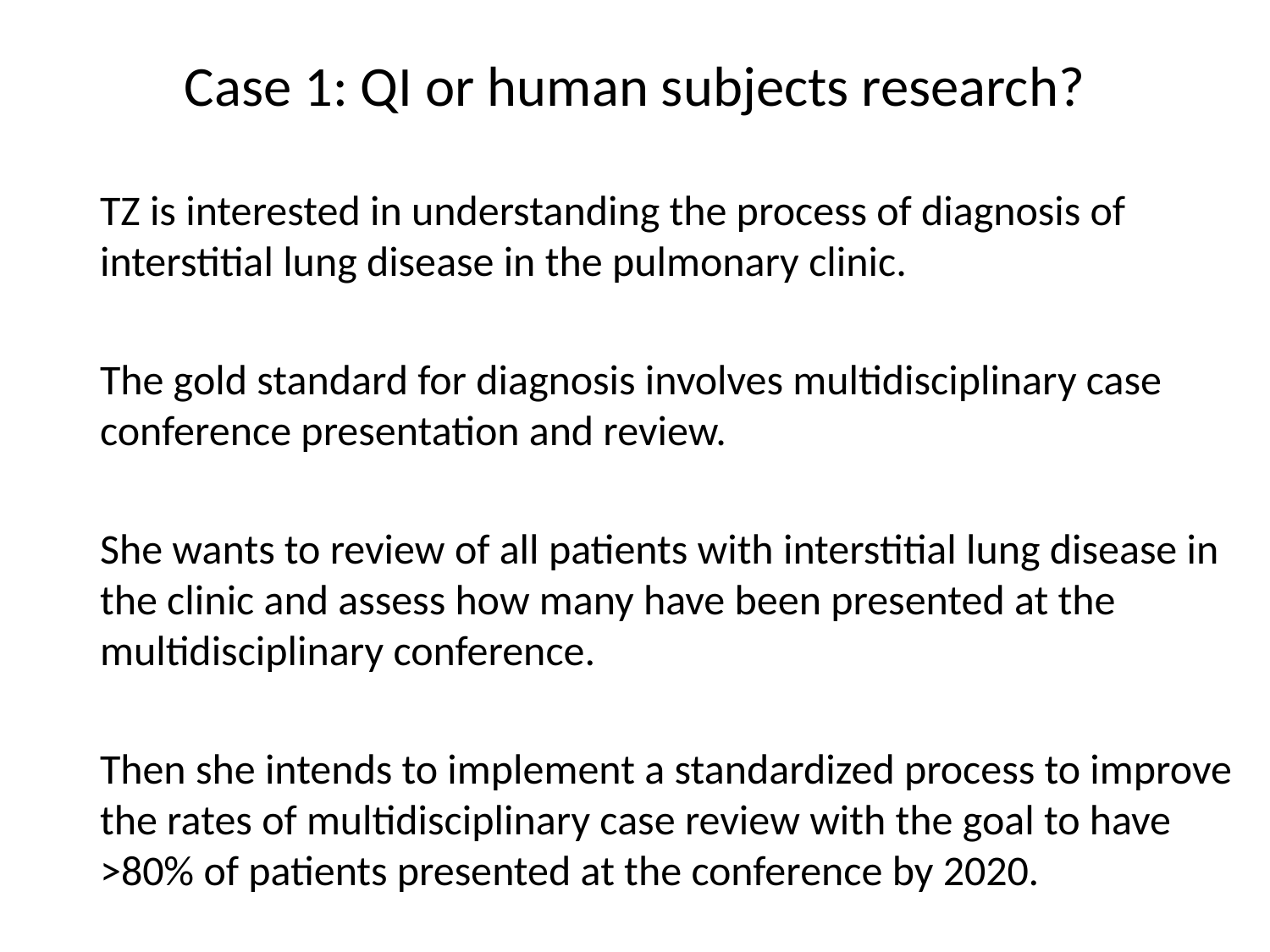

# Case 1: QI or human subjects research?
TZ is interested in understanding the process of diagnosis of interstitial lung disease in the pulmonary clinic.
The gold standard for diagnosis involves multidisciplinary case conference presentation and review.
She wants to review of all patients with interstitial lung disease in the clinic and assess how many have been presented at the multidisciplinary conference.
Then she intends to implement a standardized process to improve the rates of multidisciplinary case review with the goal to have >80% of patients presented at the conference by 2020.

## Slide 65
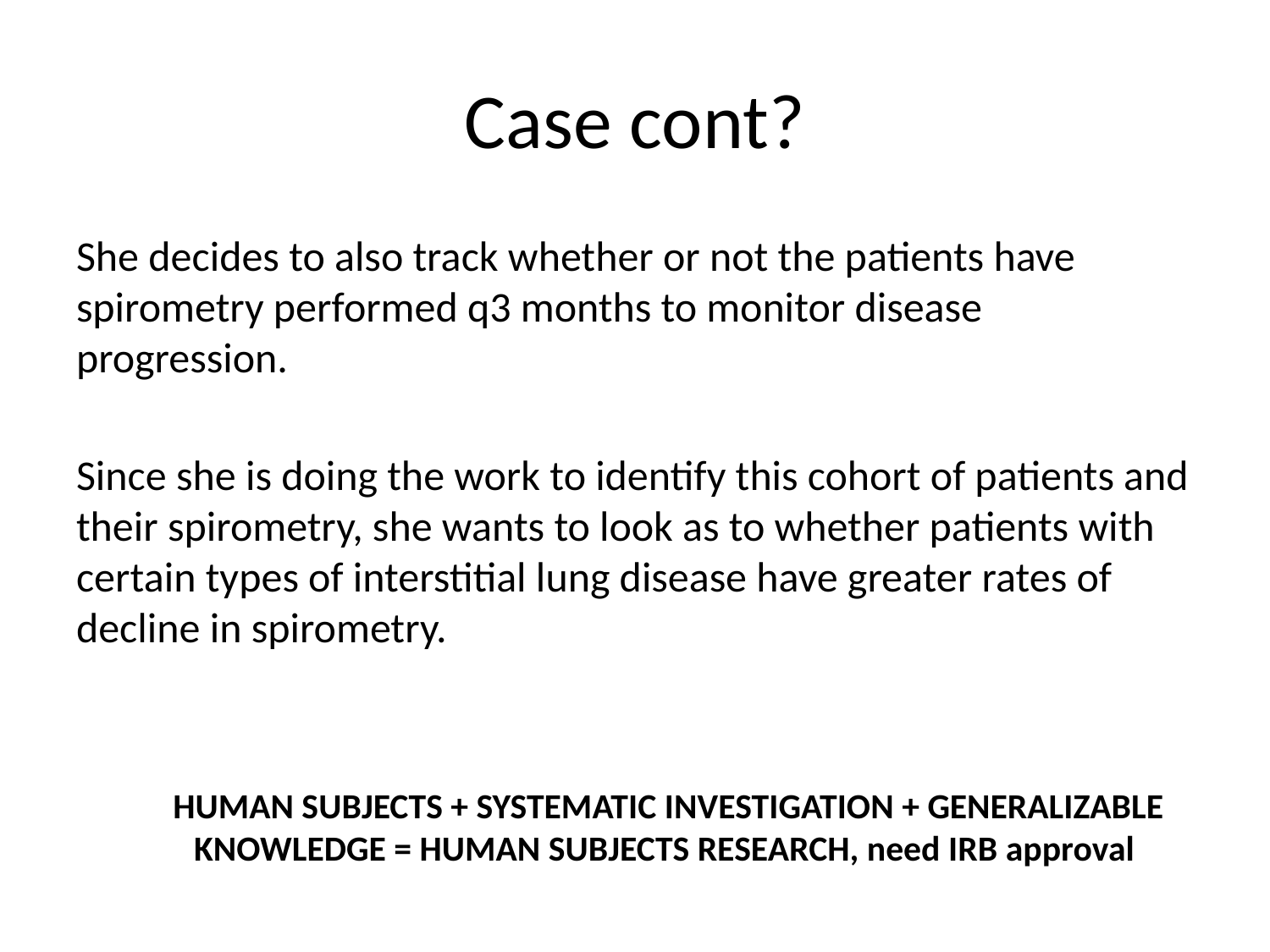

# Case cont?
She decides to also track whether or not the patients have spirometry performed q3 months to monitor disease progression.
Since she is doing the work to identify this cohort of patients and their spirometry, she wants to look as to whether patients with certain types of interstitial lung disease have greater rates of decline in spirometry.
HUMAN SUBJECTS + SYSTEMATIC INVESTIGATION + GENERALIZABLE KNOWLEDGE = HUMAN SUBJECTS RESEARCH, need IRB approval

## Slide 66
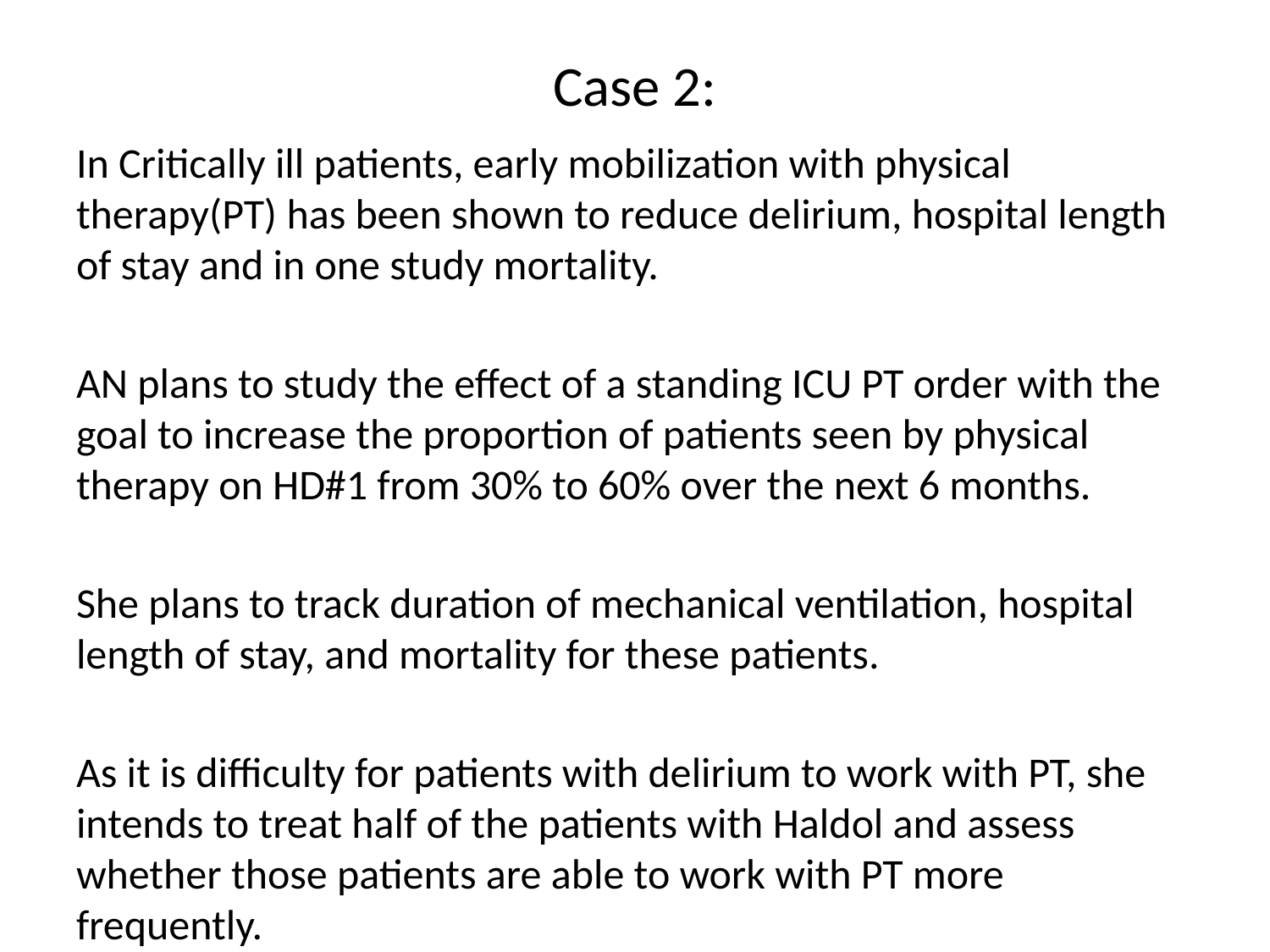

# Case 2:
In Critically ill patients, early mobilization with physical therapy(PT) has been shown to reduce delirium, hospital length of stay and in one study mortality.
AN plans to study the effect of a standing ICU PT order with the goal to increase the proportion of patients seen by physical therapy on HD#1 from 30% to 60% over the next 6 months.
She plans to track duration of mechanical ventilation, hospital length of stay, and mortality for these patients.
As it is difficulty for patients with delirium to work with PT, she intends to treat half of the patients with Haldol and assess whether those patients are able to work with PT more frequently.

## Slide 67
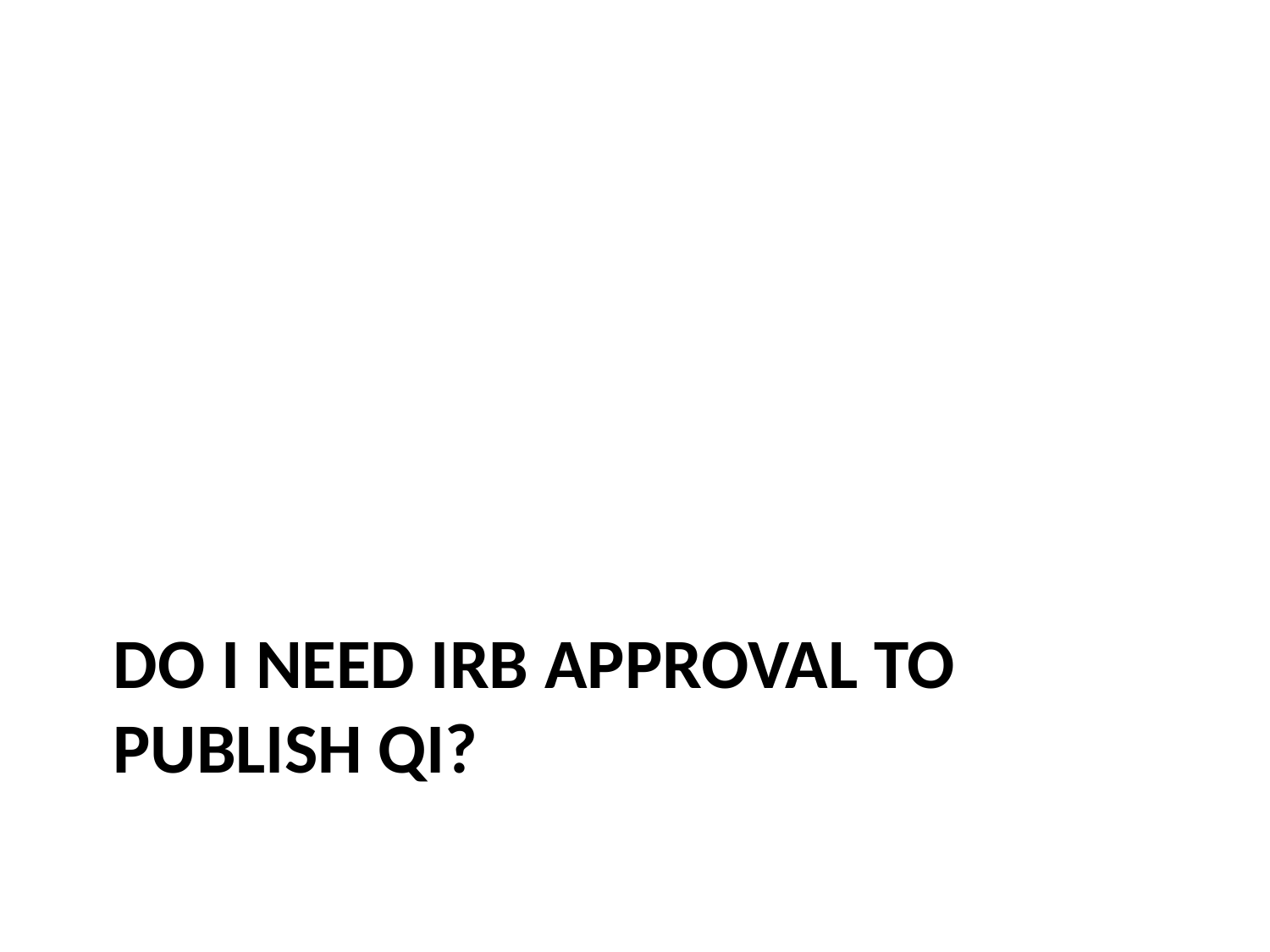

# Do I need IRB approval to publish QI?

## Slide 68
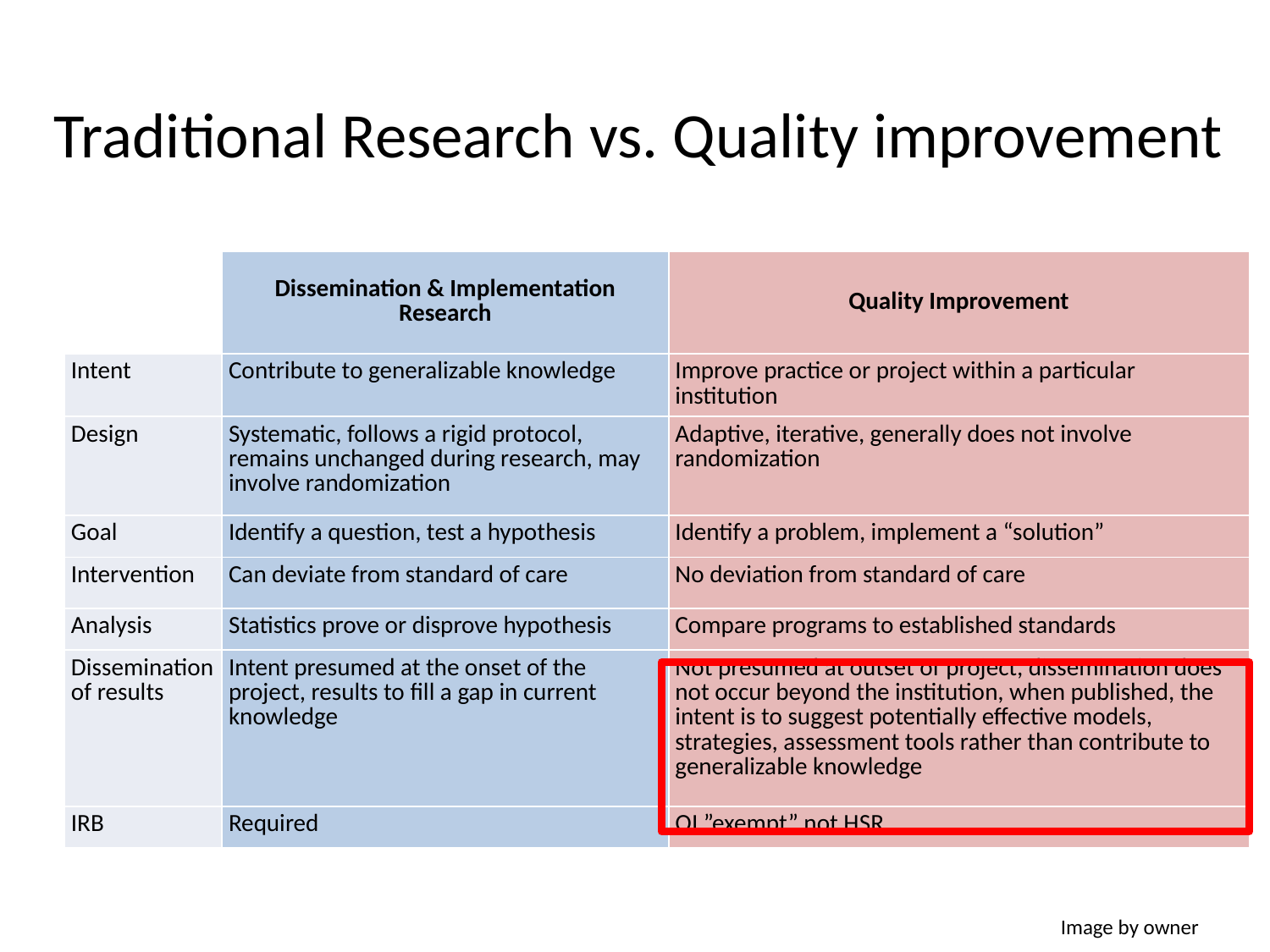

# Traditional Research vs. Quality improvement
| | Dissemination & Implementation Research | Quality Improvement |
| --- | --- | --- |
| Intent | Contribute to generalizable knowledge | Improve practice or project within a particular institution |
| Design | Systematic, follows a rigid protocol, remains unchanged during research, may involve randomization | Adaptive, iterative, generally does not involve randomization |
| Goal | Identify a question, test a hypothesis | Identify a problem, implement a “solution” |
| Intervention | Can deviate from standard of care | No deviation from standard of care |
| Analysis | Statistics prove or disprove hypothesis | Compare programs to established standards |
| Dissemination of results | Intent presumed at the onset of the project, results to fill a gap in current knowledge | Not presumed at outset of project, dissemination does not occur beyond the institution, when published, the intent is to suggest potentially effective models, strategies, assessment tools rather than contribute to generalizable knowledge |
| IRB | Required | QI ”exempt” not HSR |
Image by owner

## Slide 69
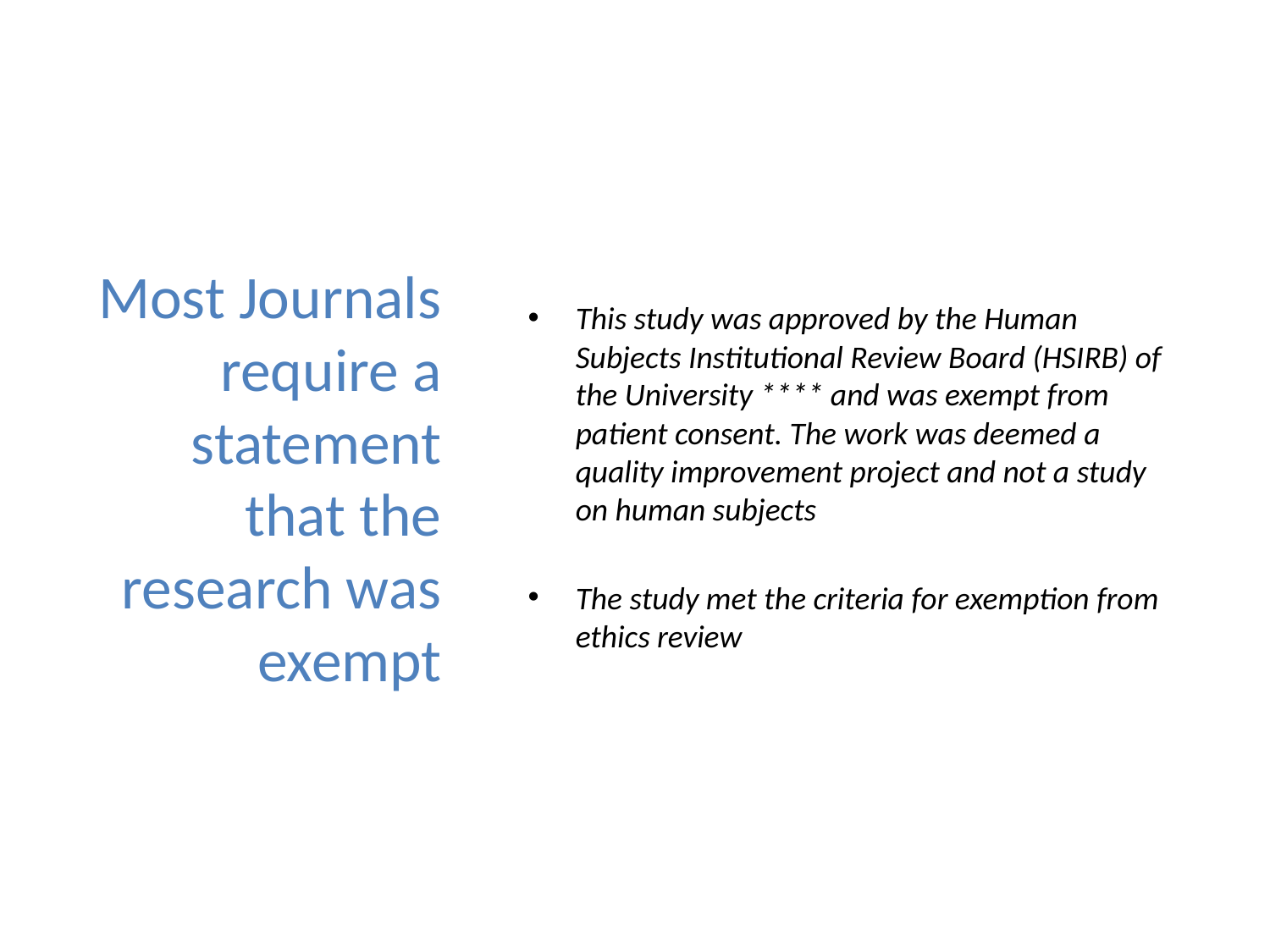

# Most Journals require a statement that the research was exempt
This study was approved by the Human Subjects Institutional Review Board (HSIRB) of the University **** and was exempt from patient consent. The work was deemed a quality improvement project and not a study on human subjects
The study met the criteria for exemption from ethics review

## Slide 70
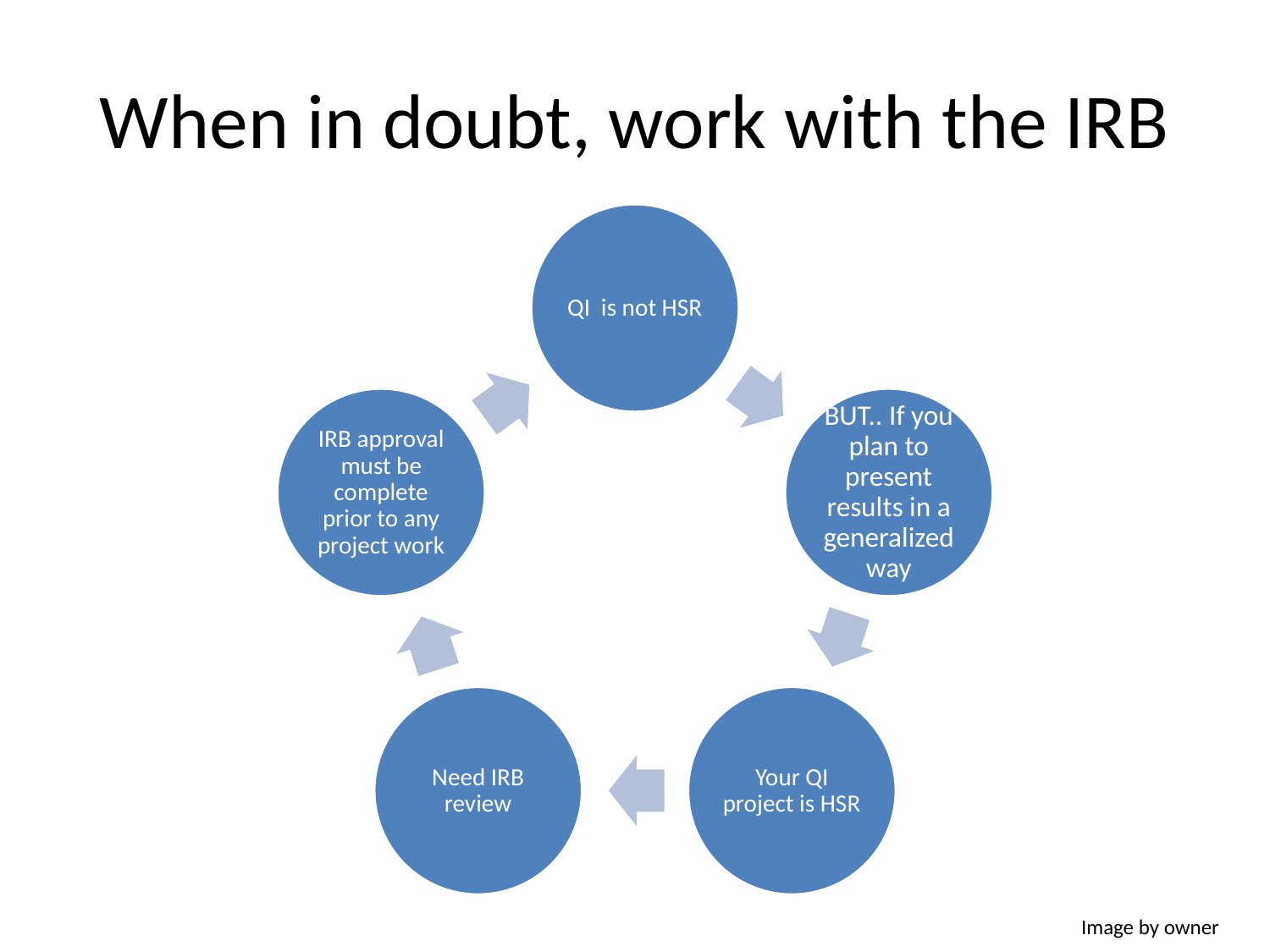

# When in doubt, work with the IRB
Image by owner

## Slide 71
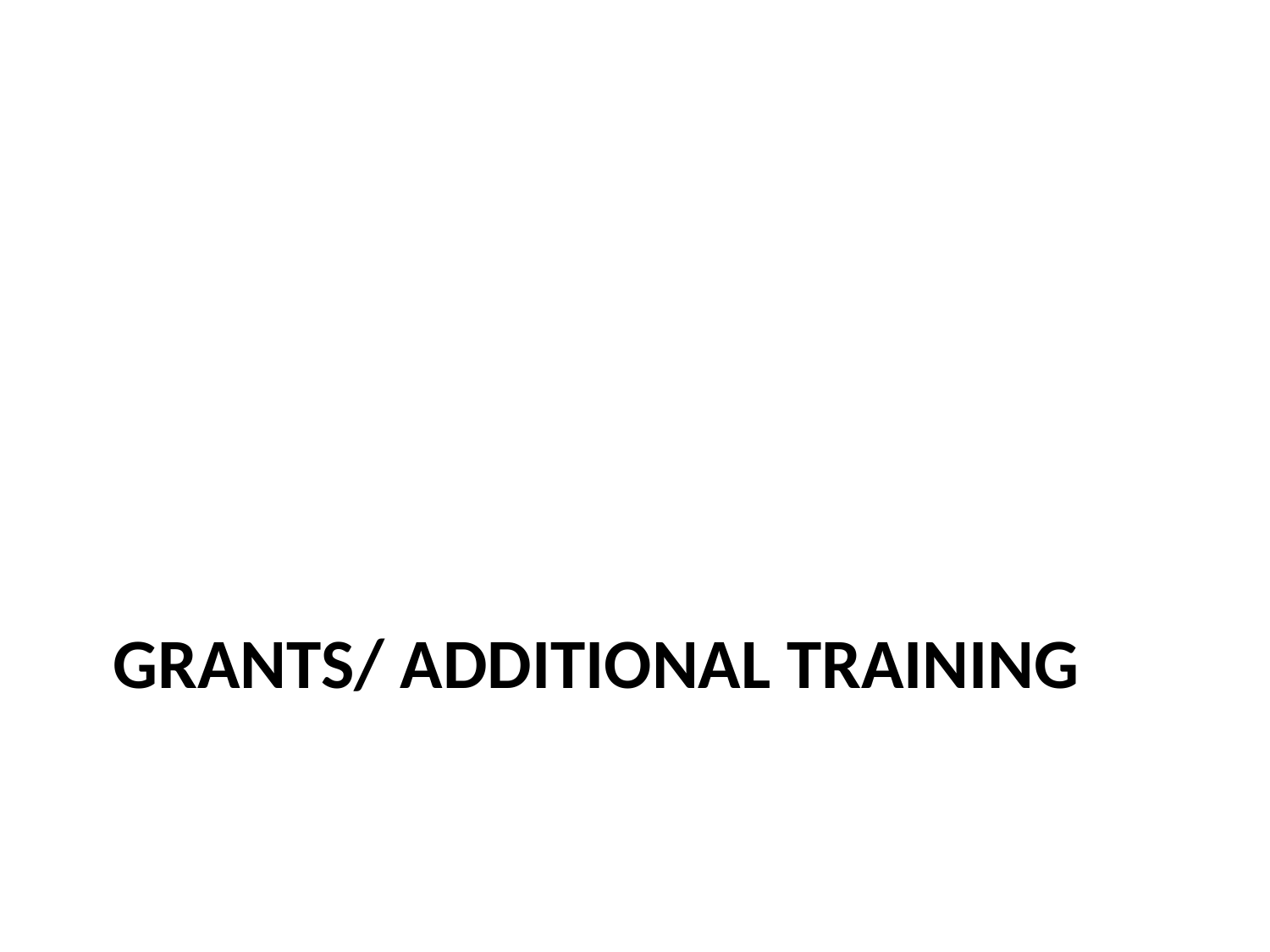

# GRANTS/ ADDITIONAL TRAINING

## Slide 72
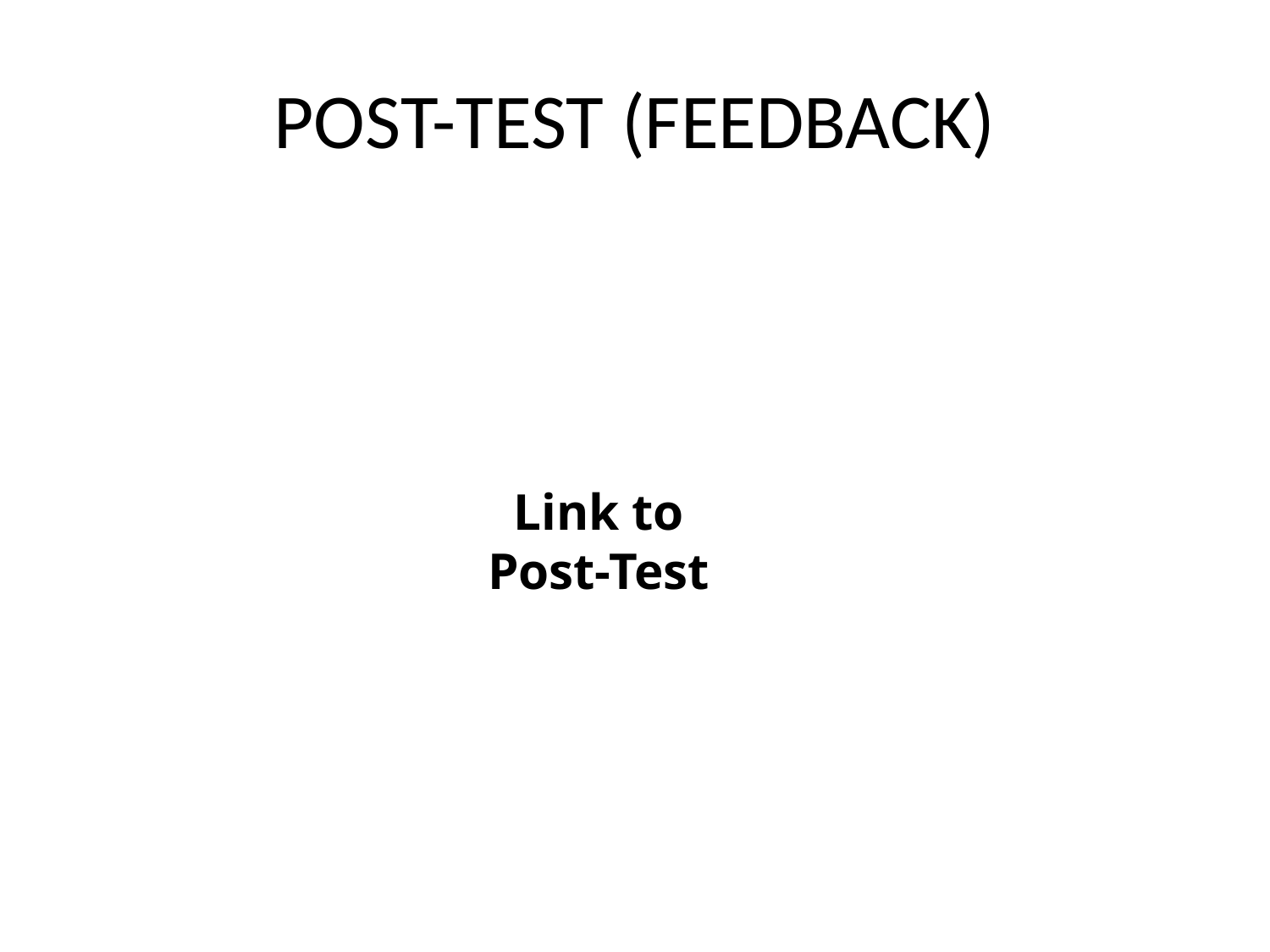

# POST-TEST (FEEDBACK)
Link to Post-Test
