# Supplementary material for: Expanding Training in Quality Improvement and Patient Safety Through a Multispecialty Graduate Medical Education Curriculum Designed for Fellows
Source: MedEdPORTAL. 2020 Dec 30;16:11064. doi: 10.15766/mep_2374-8265.11064 (PMC7780740; doi:10.15766/mep_2374-8265.11064)
Supplement: Supplementary file 1 — Foundations in Patient Safety Teaching Slides.pptxFoundations in Patient Safety Playbook and Small-Group Activities.docxAdverse Events Into QI Teaching Slides.pptxAdverse Events Into QI Playbook and Small-Group Activities.docxQuality in Academics Teaching Slides.pptxQuality in Academics Playbook and Small-Group Activities.docxFoundations in Patient Safety Assessment Survey.docxAdverse Events Into QI Assessment Survey.docxQuality in Academics Assessment Survey.docx [file mep_2374-8265.11064-s001.zip › F. Quality in Academics Playbook and Small-Group Activities.docx]

**Quality in Academics Learning Activities**

**CHANGE ROADMAP:**

What problem(s) are you trying to solve with your program/project?

List some reasons why your planned change may fail? Include people!

1. **Establish a sense of urgency**—people must genuinely believe that the status quo will not suffice, and that the program/project must begin now

- List 3 reasons why anyone should care about your program/project.

1. **Creating a guiding coalition**—generating buy-in is key to success

- List leaders, colleagues, and others who can help you start your program/project.
- Now, put those names on a stakeholder map. Mapping your stakeholders will influence your communication strategy (step 4).


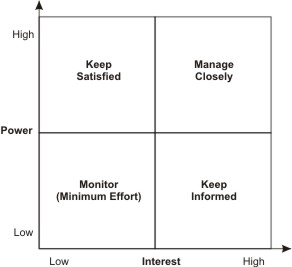


1. **Develop a vision and strategy**—people must be inspired to join you
   - What is the vision for your program/project (describe, succinctly what you will achieve)?
   - Name 3 strategies that will help you achieve your vision.
2. **Communicate the project vision**—people should start to associate you with the vision *automatically*

- List at least 4 places or avenues you can use to communicate your vision (include forums, mtgs, list serves, publications, etc.).
- For the next 6 months, create a “communication timeline”, listing rough dates and target audiences or forums for planned communication efforts. Keep your stakeholder map in mind. Plan to communicate multiple times monthly—and more often for some stakeholders than others.

| **Month 1** | **Month 2** | **Month 3** | **Month 4** | **Month 5** | **Month 6** |
| --- | --- | --- | --- | --- | --- |
| e.g., 5 min overview at monthly ACE mtg |  |  |  |  |  |
|  |  |  |  |  |  |
|  |  |  |  |  |  |
|  |  |  |  |  |  |

1. **Empower broad-based action**—make it easy to support the program/project (eliminate barriers)
2. List at least 3 major barriers to the success of your program/project?
3. List ideas for overcoming these barriers.
4. List ways in which you could make it EASIER to support the program/project.
5. **Generate short-term wins**—convert skeptics and reward supporters through frequent, clear demonstrations that your agenda carries benefits over the status quo
6. List 3 short-term wins you can achieve in the next few months.
7. List 3 rewards that you can afford to give in response to success (don’t forget appreciation—it’s often free!).
8. **Build on the change** – use your credibility to drive more change.
9. **Embed it in the culture** – design your interventions so they are permanent and systems-focused.

**Publication in QI**

**Applying the SQUIRE guidelines:**

Draft the:

**Title of your project:**

**Sample Introduction** (*hint… this may be very similar to your Elevator Pitch*)

Problem Description:

Available Knowledge:

Rationale:

Specific Aim:
