# Supplementary material for: Expanding Training in Quality Improvement and Patient Safety Through a Multispecialty Graduate Medical Education Curriculum Designed for Fellows
Source: MedEdPORTAL. 2020 Dec 30;16:11064. doi: 10.15766/mep_2374-8265.11064 (PMC7780740; doi:10.15766/mep_2374-8265.11064)
Supplement: Supplementary file 1 — Foundations in Patient Safety Teaching Slides.pptxFoundations in Patient Safety Playbook and Small-Group Activities.docxAdverse Events Into QI Teaching Slides.pptxAdverse Events Into QI Playbook and Small-Group Activities.docxQuality in Academics Teaching Slides.pptxQuality in Academics Playbook and Small-Group Activities.docxFoundations in Patient Safety Assessment Survey.docxAdverse Events Into QI Assessment Survey.docxQuality in Academics Assessment Survey.docx [file mep_2374-8265.11064-s001.zip › G. Foundations in Patient Safety Assessment Survey.docx]

**FOUNDATIONS OF PATIENT SAFETY- PRE-TEST**

1. So that we can compare pre and post survey answers, while maintaining your anonymity, please list the two digits for your birth month followed by your last two digits of your social security number.
   For example, if you were born in January and your social ends in 56 - enter 0156.

____________________________________________________________________

1. What is your level of training (PGY year)?

____________________________________________________________________

1. What is your training program?

____________________________________________________________________

1. Please rate your current interest in Patient safety on a level of 1-5 (1= week, 5 =strong)

____________________________________________________________________

1. Describe the case you will review?

____________________________________________________________________

1. In your case, what was the preventable adverse event?

____________________________________________________________________

1. What formal training have you had in patient safety/quality improvement? (select all that apply:
   1. None
   2. Curriculum during medical school
   3. Curriculum during residency
   4. Curriculum during fellowship
2. What is your highest level of experiential training in patient safety/quality improvement? (select only one)
   1. Attendance at a departmental/division M&M
   2. Involvement in a case review
   3. Group QI project in which you had a small role
   4. Group QI project in which you had a large role
   5. Initiation of a QI project
   6. Initiation and completion of a QI project
3. Have you ever submitted a patient safety report?
   1. Yes
   2. No
4. Please indicate your level of agreement with the following statements (disagree =1, somewhat disagree=2, somewhat agree=3, agree=4)
   1. It is part of my role as a physician to engage in quality improvement initiatives
      1. Disagree
      2. Somewhat disagree
      3. Somewhat agree
      4. agree
   2. I know how to file a patient safety report within my current practice setting.
      1. Disagree
      2. Somewhat disagree
      3. Somewhat agree
      4. agree
   3. I know what happens when I submit a patient safety report.
      1. Disagree
      2. Somewhat disagree
      3. Somewhat agree
      4. agree
   4. I can conduct a root-cause analysis to deepen understanding of a problem.
      1. Disagree
      2. Somewhat disagree
      3. Somewhat agree
      4. agree
   5. I feel comfortable designing a systems-focused M&M.
      1. Disagree
      2. Somewhat disagree
      3. Somewhat agree
      4. agree
5. A 21-year-old college student with a documented penicillin allergy is given doxycycline for yet another episode of chlamydia. He develops a rash from the medication. This incident is best described as:
   1. A potential adverse drug event
   2. A preventable adverse drug event
   3. A non-preventable adverse drug event
   4. A latent error
6. You were just promoted to be Clinical Chief of your division. Congratulations! In your first meeting with your new boss, he asks you to address the recent uptick in patient safety events. Later that week, your quality administrator sends you a spreadsheet in which she has cleverly categorized events according to themes (e.g. Procedural sedation, Hospital-acquired infection, Medication withheld, etc.). There are over 200 events and 22 themes. What patient safety tool can help you identify which themes to focus on in terms of Quality initiatives?
   1. Stakeholder Map
   2. Action Priority Matrix
   3. Pareto Chart
   4. Fishbone diagram

Additional Knowledge Assessment Multiple Choice Questions were obtained from MKSAP with permission for use obtained from ACP.

**FOUNDATIONS OF PATIENT SAFETY- POST-TEST**

1. So that we can compare pre and post survey answers, while maintaining your anonymity, please list the two digits for your birth month followed by your last two digits of your social security number.
   For example, if you were born in January and your social ends in 56 - enter 0156.

____________________________________________________________________

1. Please rate your current interest in Patient safety on a level of 1-5 (1= week, 5 =strong)

____________________________________________________________________

1. Having now reviewed your case, based on what you learned, describe the adverse event:

____________________________________________________________________

1. Please indicate your level of agreement with the following statements (disagree =1, somewhat disagree=2, somewhat agree=3, agree=4)
   1. It is part of my role as a physician to engage in quality improvement initiatives
      1. Disagree
      2. Somewhat disagree
      3. Somewhat agree
      4. agree
   2. I know how to file a patient safety report within my current practice setting.
      1. Disagree
      2. Somewhat disagree
      3. Somewhat agree
      4. agree
   3. I know what happens when I submit a patient safety report.
      1. Disagree
      2. Somewhat disagree
      3. Somewhat agree
      4. agree
   4. I can conduct a root-cause analysis to deepen understanding of a problem.
      1. Disagree
      2. Somewhat disagree
      3. Somewhat agree
      4. agree
   5. I feel comfortable designing a systems-focused M&M.
      1. Disagree
      2. Somewhat disagree
      3. Somewhat agree
      4. Agree
2. A 21-year-old college student with a documented penicillin allergy is given doxycycline for yet another episode of chlamydia. He develops a rash from the medication. This incident is best described as:
   1. A potential adverse drug event
   2. A preventable adverse drug event
   3. A non-preventable adverse drug event
   4. A latent error
3. You were just promoted to be Clinical Chief of your division. Congratulations! In your first meeting with your new boss, he asks you to address the recent uptick in patient safety events. Later that week, your quality administrator sends you a spreadsheet in which she has cleverly categorized events according to themes (e.g. Procedural sedation, Hospital-acquired infection, Medication withheld, etc.). There are over 200 events and 22 themes. What patient safety tool can help you identify which themes to focus on in terms of Quality initiatives?
   1. Stakeholder Map
   2. Action Priority Matrix
   3. Pareto Chart
   4. Fishbone diagram
4. This program met the learning objectives:
   1. Disagree
   2. Somewhat disagree
   3. Somewhat agree
   4. Agree
5. As the next step in patient safety/QI education, I would like to learn about (can select more than one)
   1. Nothing
   2. How to obtain data for a QI project
   3. How to analyze data for a QI project
   4. How to execute a plan for a QI project
   5. How to find mentors for a QI project
6. How satisfied are you with the session.
   1. Dissatisfied
   2. Somewhat dissatisfied
   3. Somewhat satisfied
   4. Satisfied
7. What did you like about the session?

____________________________________________________________________

1. How can the session be improved?

____________________________________________________________________

Additional Knowledge Assessment Multiple Choice Questions were obtained from MKSAP with permission for use obtained from ACP.
