# Supplementary material for: Expanding Training in Quality Improvement and Patient Safety Through a Multispecialty Graduate Medical Education Curriculum Designed for Fellows
Source: MedEdPORTAL. 2020 Dec 30;16:11064. doi: 10.15766/mep_2374-8265.11064 (PMC7780740; doi:10.15766/mep_2374-8265.11064)
Supplement: Supplementary file 1 — Foundations in Patient Safety Teaching Slides.pptxFoundations in Patient Safety Playbook and Small-Group Activities.docxAdverse Events Into QI Teaching Slides.pptxAdverse Events Into QI Playbook and Small-Group Activities.docxQuality in Academics Teaching Slides.pptxQuality in Academics Playbook and Small-Group Activities.docxFoundations in Patient Safety Assessment Survey.docxAdverse Events Into QI Assessment Survey.docxQuality in Academics Assessment Survey.docx [file mep_2374-8265.11064-s001.zip › H. Adverse Events Into QI Assessment Survey.docx]

**Adverse Events into QI pre and post survey assessment**

**PRE-ASSESSMENT:**

1. So that we can compare pre and post survey answers, while maintaining your anonymity, please list the two digits for your birth month followed by your last two digits of your social security number.
   For example, if you were born in January and your social ends in 56 - enter 0156.

____________________________________________________________________

1. What is your level of training (PGY year)?

____________________________________________________________________

1. What is your training program?

____________________________________________________________________

1. Please rate your current interest in Patient safety on a level of 1-5 (1= week, 5 =strong)

____________________________________________________________________

1. Please describe the problem you are working to improve.

____________________________________________________________________

1. Which prior Quality and Safety Academy Seminars have you attended? (select all that apply)
   1. Foundation in Patient Safety
   2. Quality in Academics
2. Please indicate your level of agreement with the following statements (disagree =1, somewhat disagree=2, somewhat agree=3, agree=4)
   1. It is part of my role as a physician to engage in quality improvement initiatives.
      1. Disagree
      2. Somewhat disagree
      3. Somewhat agree
      4. agree
   2. QI is important for improving care.
      1. Disagree
      2. Somewhat disagree
      3. Somewhat agree
      4. agree
   3. I understand the importance of defining a problem before implementing solutions.
      1. Disagree
      2. Somewhat disagree
      3. Somewhat agree
      4. agree
   4. I can use some of the QI tools (ishikawa, process map, 5-why's) to investigate a problem.
      1. Disagree
      2. Somewhat disagree
      3. Somewhat agree
      4. agree
   5. I am familiar with how to obtain data (for example using the tools to extract from the EMR).
      1. Disagree
      2. Somewhat disagree
      3. Somewhat agree
      4. agree
   6. I can describe the data tools analysis methods used in QI
      1. Disagree
      2. Somewhat disagree
      3. Somewhat agree
      4. agree
3. You were just promoted to be Clinical Chief of your division. Congratulations! In your first meeting with your new boss, he asks you to address the recent uptick in patient safety events. Later that week, your quality administrator sends you a spreadsheet in which she has cleverly categorized events according to themes (e.g. Procedural sedation, Hospital-acquired infection, Medication withheld, etc.). There are over 200 events and 22 themes. What patient safety tool can help you identify which themes to focus on in terms of Quality initiatives?
   1. Stakeholder Map
   2. Action Priority Matrix
   3. Pareto Chart
   4. Fishbone diagram
4. Which of these would NOT qualify as a QI project?
   1. Reducing readmission rates on the medicine floor
   2. Improving patient satisfaction in a dialysis center
   3. Testing the best drug to reduce hypertension
   4. Preventing polypharmacy in the geriatric population
5. What action is most consistent with designing a QI project?
   1. Selecting a large enough sample size
   2. Blinding investigators to interventions and outcomes
   3. Collecting retrospective information for a database
   4. Frequently re-evaluating the success of an intervention

All participants were then administered the QIKAT-R- Anesthesia case

Singh MK, Ogrinc G, Cox KR, et al. The Quality Improvement Knowledge Application Tool Revised (QIKAT-R). *Acad Med*. 2014;89(10):1386-1391. doi:10.1097/ACM.0000000000000456

**POST-ASSESSMENT:**

1. So that we can compare pre and post survey answers, while maintaining your anonymity, please list the two digits for your birth month followed by your last two digits of your social security number. For example, if you were born in January and your social ends in 56 - enter 0156.

____________________________________________________________________

1. This program met the learning objectives:
   1. Disagree
   2. Somewhat disagree
   3. Somewhat agree
   4. Agree
2. Please rate your current interest in Patient safety on a level of 1-5 (1= week, 5 =strong)

___________________________________________________________________

1. Please indicate your level of agreement with the following statements (disagree =1, somewhat disagree=2, somewhat agree=3, agree=4)
   1. It is part of my role as a physician to engage in quality improvement initiatives.
      1. Disagree
      2. Somewhat disagree
      3. Somewhat agree
      4. agree
   2. QI is important for improving care.
      1. Disagree
      2. Somewhat disagree
      3. Somewhat agree
      4. agree
   3. I understand the importance of defining a problem before implementing solutions.
      1. Disagree
      2. Somewhat disagree
      3. Somewhat agree
      4. agree
   4. I can use some of the QI tools (ishikawa, process map, 5-why's) to investigate a problem.
      1. Disagree
      2. Somewhat disagree
      3. Somewhat agree
      4. agree
   5. I am familiar with how to obtain data (for example using the tools to extract from the EMR).
      1. Disagree
      2. Somewhat disagree
      3. Somewhat agree
      4. agree
   6. I can describe the data tools analysis methods used in QI
      1. Disagree
      2. Somewhat disagree
      3. Somewhat agree
      4. Agree
2. Which of these would NOT quality as a QI project?
   1. Reducing readmission rates on the medicine floor
   2. Improving patient satisfaction in a dialysis center
   3. Testing the best drug to reduce hypertension
   4. Preventing polypharmacy in the geriatric population
3. What action is most consistent with designing a QI project?
   1. Selecting a large enough sample size
   2. Blinding investigators to interventions and outcomes
   3. Collecting retrospective information for a database
   4. Frequently re-evaluating the success of an intervention

All participants were then administered the QIKAT-R- Radiology case

1. As the next step in patient safety/QI education, I would like to learn about (can select more than one)
   1. Nothing
   2. How to obtain data for a QI project
   3. How to analyze data for a QI project
   4. How to execute a plan for a QI project
   5. How to find mentors for a QI project
2. How satisfied are you with the session?
   1. Dissatisfied
   2. Somewhat dissatisfied
   3. Somewhat satisfied
   4. Satisfied
3. What did you like about the session?

______________________________________________________________________

1. How can the session be improved?

______________________________________________________________________
