# Supplementary material for: Expanding Training in Quality Improvement and Patient Safety Through a Multispecialty Graduate Medical Education Curriculum Designed for Fellows
Source: MedEdPORTAL. 2020 Dec 30;16:11064. doi: 10.15766/mep_2374-8265.11064 (PMC7780740; doi:10.15766/mep_2374-8265.11064)
Supplement: Supplementary file 1 — Foundations in Patient Safety Teaching Slides.pptxFoundations in Patient Safety Playbook and Small-Group Activities.docxAdverse Events Into QI Teaching Slides.pptxAdverse Events Into QI Playbook and Small-Group Activities.docxQuality in Academics Teaching Slides.pptxQuality in Academics Playbook and Small-Group Activities.docxFoundations in Patient Safety Assessment Survey.docxAdverse Events Into QI Assessment Survey.docxQuality in Academics Assessment Survey.docx [file mep_2374-8265.11064-s001.zip › I. Quality in Academics Assessment Survey.docx]

QUALITY IN ACADEMICS

PRE-ASSESSMENT:

So that we can compare pre and post survey answers, while maintaining your anonymity, please list the two digits for your birth month followed by the last two digits of your social security number
For example, if you were born in January and your social ends in 56 - enter 0156.

­­­­­­ ______________________________________________________________________________

1. What is your level of training (PGY year)?

______________________________________________________________________________

1. What is your training program?

______________________________________________________________________________

1. Please rate your current interest in Patient safety on a level of 1-5 (1= week, 5 =strong)

______________________________________________________________________________

1. Please describe the problem you are working to improve.

______________________________________________________________________________

1. Which prior Quality and Safety Academy Seminars have you attended? (select all that apply)
   1. Foundation in Patient Safety
   2. Adverse Events into QI
2. Please indicate your level of agreement with the following statements (disagree =1, somewhat disagree=2, somewhat agree=3, agree=4)
   1. It is part of my role as a physician to engage in quality improvement initiatives.
      1. Disagree
      2. Somewhat disagree
      3. Somewhat agree
      4. Agree
   2. QI is important for improving care.
      1. Disagree
      2. Somewhat disagree
      3. Somewhat agree
      4. Agree
   3. I understand how to use Kotter’s 8-step model for change
      1. Disagree
      2. Somewhat disagree
      3. Somewhat agree
      4. Agree
   4. I know about the SQUIRE guide for publishing QI
      1. Disagree
      2. Somewhat disagree
      3. Somewhat agree
      4. Agree
   5. I understand when to pursue IRB review for QI work
      1. Disagree
      2. Somewhat disagree
      3. Somewhat agree
      4. Agree
3. You outpatient clinic hopes to design a strategy to increase influenza vaccination rates this year. Which of the following projects would be classified as QI instead of human subjects research?
   1. Assessing whether the use of phone or text message reminders to increase the proportion of patients who receive influenza vaccination.
   2. Initiating an immunization protocol in clinic to increase the proportion of patients with influenza vaccination by 20%.
   3. Interviewing group of patients to identify barriers to receiving influenza immunization.
   4. Determining the comparative efficacy of forms of influenza vaccination (inhaled vs injected).

POST- ASSESSMENT:

So that we can compare pre and post survey answers, while maintaining your anonymity, please list the two digits for your birth month followed by the last two digits of your social security number
For example, if you were born in January and your social ends in 56 - enter 0156.

______________________________________________________________________________

1. What is your level of training (PGY year)?

______________________________________________________________________________

1. Please rate your current interest in Patient safety on a level of 1-5 (1= week, 5 =strong)

______________________________________________________________________________

1. Please indicate your level of agreement with the following statements (disagree =1, somewhat disagree=2, somewhat agree=3, agree=4)
   1. It is part of my role as a physician to engage in quality improvement initiatives.
      1. Disagree
      2. Somewhat disagree
      3. Somewhat agree
      4. Agree
   2. QI is important for improving care.
      1. Disagree
      2. Somewhat disagree
      3. Somewhat agree
      4. Agree
   3. I understand how to use Kotter’s 8-step model for change
      1. Disagree
      2. Somewhat disagree
      3. Somewhat agree
      4. Agree
   4. I know about the SQUIRE guide for publishing QI
      1. Disagree
      2. Somewhat disagree
      3. Somewhat agree
      4. Agree
   5. I understand when to pursue IRB review for QI work
      1. Disagree
      2. Somewhat disagree
      3. Somewhat agree
      4. Agree
2. You outpatient clinic hopes to design a strategy to increase influenza vaccination rates this year. Which of the following projects would be classified as QI instead of human subjects research?
   1. Assessing whether the use of phone or text message reminders to increase the proportion of patients who receive influenza vaccination.
   2. Initiating an immunization protocol in clinic to increase the proportion of patients with influenza vaccination by 20%.
   3. Interviewing group of patients to identify barriers to receiving influenza immunization.
   4. Determining the comparative efficacy of forms of influenza vaccination (inhaled vs injected).
3. How satisfied are you with the session?
   1. Dissatisfied
   2. Somewhat dissatisfied
   3. Somewhat satisfied
   4. Satisfied
4. What did you like about the session?

______________________________________________________________________________

1. How can the session be improved?

‘

______________________________________________________________________________
