# Supplementary material for: Expanding Training in Quality Improvement and Patient Safety Through a Multispecialty Graduate Medical Education Curriculum Designed for Fellows
Source: MedEdPORTAL. 2020 Dec 30;16:11064. doi: 10.15766/mep_2374-8265.11064 (PMC7780740; doi:10.15766/mep_2374-8265.11064)
Supplement: Supplementary file 1 — Foundations in Patient Safety Teaching Slides.pptxFoundations in Patient Safety Playbook and Small-Group Activities.docxAdverse Events Into QI Teaching Slides.pptxAdverse Events Into QI Playbook and Small-Group Activities.docxQuality in Academics Teaching Slides.pptxQuality in Academics Playbook and Small-Group Activities.docxFoundations in Patient Safety Assessment Survey.docxAdverse Events Into QI Assessment Survey.docxQuality in Academics Assessment Survey.docx [file mep_2374-8265.11064-s001.zip › _Educational_Summary_Report_11064.pdf]

MedEdPORTAL® The AAMC Journal of Teaching and Learning Resources

Original Publication Open Access

Expanding Training in Quality Improvement and Patient Safety Through a Multispecialty Graduate Medical Education Curriculum Designed for Fellows

Anna Neumeier, MD*, Andrew E. Levy, MD, Emily Gottenborg, MD, Tyler Anstett, DO, Read G. Pierce, MD, Darlene Tad-y, MD

*Corresponding author: anna.neumeier@cuanschutz.edu

Abstract

Introduction: Although the Accreditation Council for Graduate Medical Education requires quality improvement and patient safety (QIPS) training for fellow-level trainees, this experience is often insufficient due to lack of faculty time and expertise within fellowship training programs. We developed a centralized GME curriculum targeted to an integrated, multispecialty audience of fellow-level trainees with the goal of promoting leadership and scholarship in QIPS. **Methods:** The University of Colorado implemented the Fellows' Quality and Safety Academy, a three-seminar curriculum in patient safety and health systems improvement. As most participants had prior training in QIPS during medical school or residency, educational strategies emphasized application of QIPS concepts through focused didactic content review paired with small-group case-based exercises and coaching of experiential project work to promote content mastery as well as practice of leadership and scholarship strategies. **Results:** Since the curriculum's inception in 2017, there have been 106 participants in the Foundations in Patient Safety seminar, 49 participants in the Adverse Events Into Quality Improvement seminar, and 48 participants in the Quality in Academics seminar. These participants represented 44 separate fellowship disciplines from both adult and pediatric subspecialties. Learners reported improved attitudes and confidence and demonstrated objective knowledge acquisition across QIPS content domains. **Discussion:** Our pedagogical approach of centralizing QIPS training and harnessing faculty expertise to teach fellow-level trainees across specialties through interdisciplinary collaboration and interactive project-based work is an effective strategy to promote development of QIPS competencies during fellowship training.

Keywords:
Quality Improvement, Patient Safety, Fellow, Leadership, Scholarship, Quality Improvement/Patient Safety, Case-Based Learning

Educational Objectives

By the end of this activity, learners will be able to:

1. Define types of patient safety adverse events and assess level of harm associated with these events.
2. Conduct an adverse event analysis using quality and safety tools to identify systematic errors and sources of cognitive bias.
3. Design and deliver a morbidity and mortality conference that promotes principles of just culture.
4. Identify actionable patient safety events and understand the current organizational infrastructure at the training institution used to implement necessary change.
5. Apply tools of quality improvement and data interpretation.
6. Integrate principles of change management to lead effective quality improvement projects.
7. Identify necessary components for scholarship and sources of grant funding for quality improvement work.

Citation:
Neumeier A, Levy AE, Gottenborg E, Anstett T, Pierce RG, Tad-y D. Expanding training in quality improvement and patient safety through a multispecialty graduate medical education curriculum designed for fellows. *MedEdPORTAL*. 2020;16:11064.
https://doi.org/10.15766/mep_2374-8265.11064

Introduction

As an Accreditation Council for Graduate Medical Education (ACGME) competency requirement, postgraduate medical trainees are expected to engage in patient safety (PS) practice, systematically analyze practice using quality improvement (QI) methods, and implement changes with the goal of practice improvement.[1] Despite increased efforts to improve education and training in QI and PS (QIPS) at the subspecialty fellowship level, the optimal delivery strategy remains unclear, particularly given the ongoing severe shortage of faculty in all specialties who are highly experienced in both teaching and doing QIPS. Currently, most educational literature and published curricula describe educational programs focused on medical students

Copyright © 2020 Neumeier et al. This is an open-access publication distributed under the terms of the Creative Commons Attribution license.

or residents, emphasizing the need for fellow-level curricula.[2] Previously identified implementation challenges of QIPS education within subspecialty fellowship training programs include lack of faculty expertise, time constraints, and competing priorities amongst trainees and their mentors.[3,4] Furthermore, as QIPS education occurs across the continuum of physician development, educational efforts must be coordinated to reinforce content and advance skills in order to optimize learner engagement and avoid unnecessary duplication of learning activities. To promote this continuum of professional advancement for fellow-level learners in QIPS, practice and development of QIPS skills through the framework of leadership and scholarship—two competencies emphasized in fellowship training programs—are needed.

Through a general and targeted needs assessment, reviewing available literature and surveying both program directors and trainees, we identified an educational landscape of QIPS for fellows at the University of Colorado School of Medicine similar to that described nationally. Fellow participation in mandatory ACGME QIPS activities was infrequent. Only half of fellows (50%) reported systematically analyzing their practice, and even fewer (38%) reported receiving performance data on quality measures. The majority of fellows (90%) agreed on the importance of engaging in QI initiatives. The majority of program directors (75%) were interested in creation of a formal QIPS curriculum, but comparably few programs (25%) could identify faculty who could contribute to quality and safety education for fellows. To meet this curricular need, we designed and implemented the Fellows' Quality and Safety Academy, a multispecialty training program centralized with the Graduate Medical Education Office in the School of Medicine, to promote postgraduate fellow practice in QIPS, targeting the specific needs of fellows and providing a more advanced training course building on prior curricula offered in medical school and residency.

To date, although *MedEdPORTAL* has many QI educational resources,[5-19] none focus on the learner at the fellowship level. Our resource builds upon the framework and content of existing curricula to expand and advance QIPS practice across the continuum of professional development, with a goal of developing the knowledge, skills, and attitudes necessary among fellows-in-training to enhance leadership and scholarship in PS, QI data acquisition, interpretation, and project design and implementation.

Methods

In designing the Fellows' Quality and Safety Academy, we sought to provide a comprehensive curriculum encompassing ACGME-based competencies promoting knowledge and skills development that built upon learners' prior experience and training. The needs assessment revealed that the majority of fellow-level trainees had received prior education in QIPS during either residency or medical school. Therefore, applying spiral learning theory,[20] learners reviewed common concepts in QIPS through active learning strategies promoting application of QIPS concepts as well as more advanced leadership strategies.

The curriculum was instituted in the fall of 2017 and focused on PS practice for fellows within subspecialties of internal medicine. Due to popularity and demand, we expanded the curriculum in 2018 to a 12-hour seminar-based active learning curriculum targeting fellows across all specialties. Topics from the Institute for Healthcare Improvement and our institution's Institute for Healthcare Quality, Safety, and Efficiency (IHQSE), comprising local faculty experts, guided the curricular content. Facilitators from both medical and surgical departments with mastery of QIPS in academic settings led the seminars. We obtained institutional sponsorship from both the University of Colorado Graduate Medical Education Office and the IHQSE, a multidisciplinary collaboration promoting skills acquisition and practice of QI, safety, and systems redesign leadership.

Through the Fellows' Quality and Safety Academy, fellows enrolled in three seminars that repeated biannually (spring and fall): (1) Foundations in Patient Safety: Performing a Case Review, (2) Quality Improvement: Turning Adverse Events Into Local Change, and (3) Quality in Academics: Strategies to Drive Sustainability and Dissemination. All seminars were held in central conference areas with audiovisual capabilities (projector and computer). Additional educational materials included activity handouts provided on paper and electronically.

Seminar 1: Foundations in Patient Safety

For the first seminar (Appendix A; see also Figure 1), learners were instructed to bring a case of an adverse event in which they had been personally involved and were prepared to analyze. Through small-group and paired breakout activities (Appendix B), learners analyzed their case using tools to conduct a root cause analysis, identified actionable safety issues, prepared their case to present as a morbidity and mortality (M&M) conference, and practiced facilitation techniques necessary to lead the discussion at the M&M conference.

Seminar 2: Adverse Events Into Quality Improvement

In the second seminar (Appendix C; see also Figure 2), learners returned to work on the action items they had identified during the first seminar or arrived with another potential project for

Copyright © 2020 Neumeier et al. This is an open-access publication distributed under the terms of the Creative Commons Attribution license.

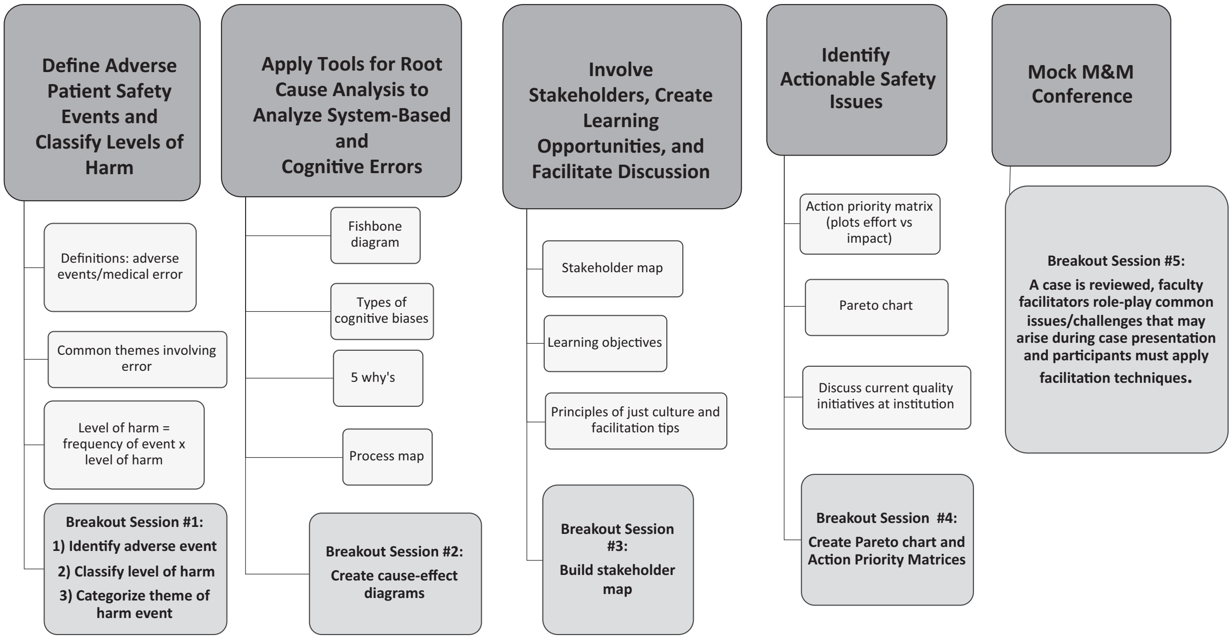

Figure 1. Foundations in Patient Safety curricular map. Abbreviation: M&M, morbidity and mortality.

QI. Through small-group activities (Appendix D), they planned the steps of their QI project, applied tools of problem analysis, created an aim statement, identified potential performance measures, learned how to obtain appropriate data, and selected improvement interventions.

Seminar 3: Quality in Academics

During the third seminar (Appendix E; see also Figure 3), through small-group breakout sessions (Appendix F), learners applied change management strategies to their project, reviewed local institutional review board policies, and learned dissemination

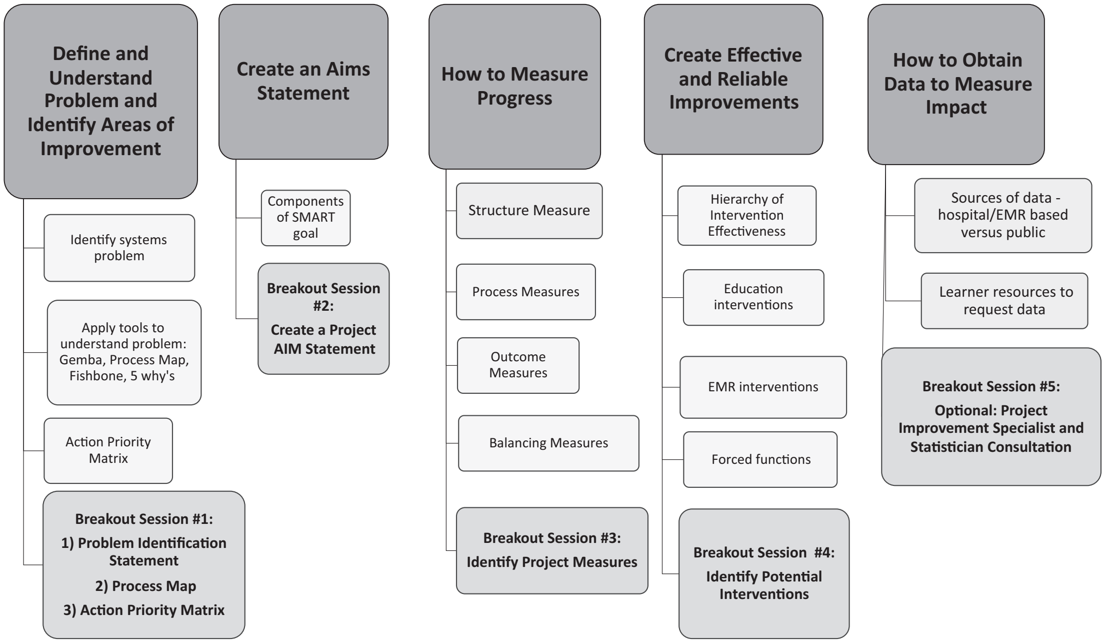

Figure 2. Adverse Events Into Quality Improvement curricular map. Abbreviation: EMR, electronic medical record.

Copyright © 2020 Neumeier et al. This is an open-access publication distributed under the terms of the Creative Commons Attribution license.

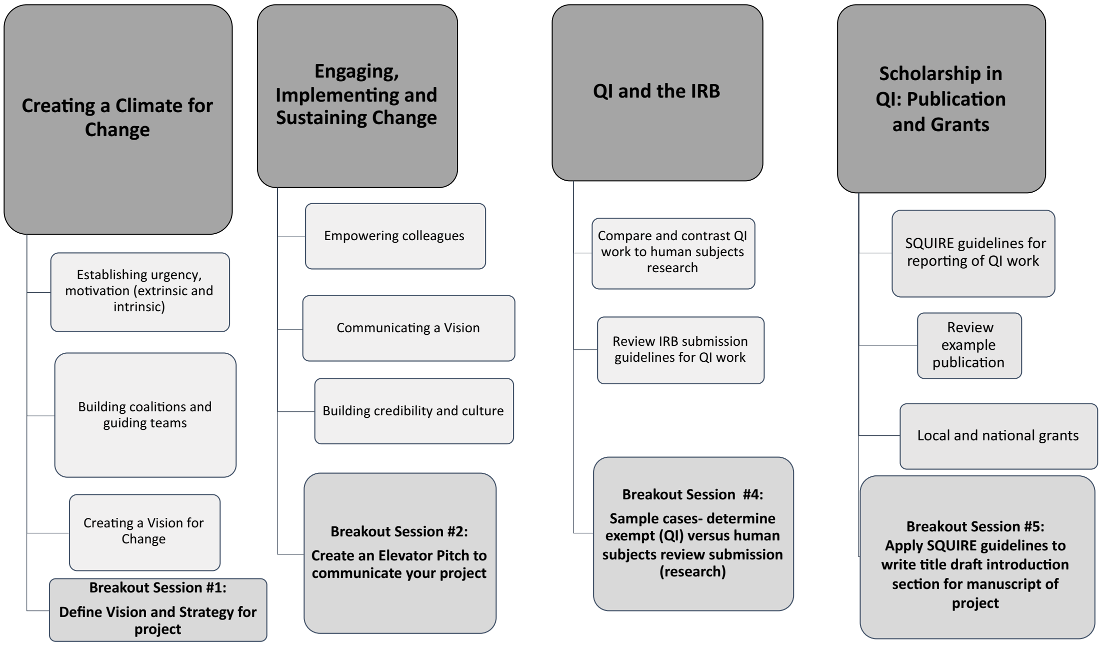

Figure 3. Quality in Academics curricular map. Abbreviations: IRB, institutional review board; QI, quality improvement.

and publication strategies for QI work that emphasized how to turn QIPS efforts into scholarship that could support academic promotion.

Assessment and Program Evaluation

The program evaluation was submitted to the University of Colorado Institutional Review Board and deemed exempt from formal review. Included in the evaluation were presurvey and postsurvey assessment question for each seminar (Appendices G-I). Learners completed self-reported knowledge and skills assessment as well as objective knowledge assessment questions. In developing the knowledge assessment questions, we used previously developed multiple-choice questions from prior QIPS curricula,[19] MKSAP version 17 questions for which permission was obtained,[21] the Quality Improvement Knowledge Application Tool Revised (QIKAT-R),[22] and open-ended application questions. For statistical analysis, to account for the paired nature of the data, we used linear mixed models to compare between questions on Likert scales and linear mixed models and generalized binomial mixed models to compare correct/incorrect questions.

Results

Between 2017 and 2019, there were 106 participants in the Foundations in Patient Safety seminar, 49 participants in the Adverse Events Into Quality Improvement seminar and 48 participants in the Quality in Academics seminar. As of 2018, when all three seminars were offered, 24 of 57 participants (42%) completed all three seminars in the series. Fellows from across 44 separate disciplines in both adult and pediatric subspecialties attended. Eighty-three of 106 participants (78%) in the Foundations in Patient Safety seminar, 28 of 49 participants (57%) in the Adverse Events Into Quality Improvement seminar, and 46 of 47 participants (98%) in the Quality in Academics seminar completed evaluations. Ninety percent of respondents reported the highest level of satisfaction with the courses.

Fellow trainees reported improvement in self-assessed knowledge, skills, and attitudes across PS and QI content domains (for details, see the top half of the Table). Evaluations revealed significant differences showing improvement between pretest and posttest in self-reported knowledge and skills in filing a PS report, performance of a root cause analysis, design of a systems-focused M&M, use of QI tools, application of the Kotter model for change management,[23] use of Standards for Quality Improvement Reporting Excellence. (SQUIRE) guidelines,[24] and navigating interactions with the institutional review board.

The objective knowledge assessment, using multiple-choice questions as well as the QIKAT-R score, demonstrated overall improvement in knowledge in most domains, with statistically significant difference in knowledge surrounding identification of the second victim and selection of the appropriate PS tool (for details, see the bottom half of the Table). Qualitative

Copyright © 2020 Neumeier et al. This is an open-access publication distributed under the terms of the Creative Commons Attribution license.

Table. Change in Self-Rated Knowledge, Skills, and Attitudes and in Objective Knowledge

Item	Presurvey Likert Score *M* (*SD*)	Postsurvey Likert Score *M* (*SD*)	*p*
Self-reported change in knowledge, skills, and attitudes[a]			
It is part of my role as a physician to engage in QI.	3.7 (0.48)	3.8 (0.40)	.081
I know how to file a patient safety report.	2.7 (1.11)	3.6 (0.67)	<.001
I know what happens when I submit a patient safety report.	2.3 (1.00)	3.2 (0.66)	<.001
I can conduct a root cause analysis.	2.8 (0.80)	3.4 (0.62)	<.001
I feel comfortable designing a system M&M.	2.7 (0.90)	3.4 (0.61)	<.001
It is part of my role as a physician to engage in QI.	3.7 (0.46)	3.9 (0.24)	.035
QI is important for improving care.	3.9 (0.36)	3.9 (0.24)	.396
I understand the importance of defining a problem.	3.8 (0.44)	3.8 (0.39)	.387
I can use QI tools to investigate a problem.	2.4 (1.02)	3.6 (0.51)	<.001
I am familiar with the Slicer Dicer tool.	2.1 (1.13)	3.5 (0.62)	<.001
I can describe the tools used in QI.	2.0 (0.72)	3.4 (0.70)	<.001
It is part of my role as a physician to engage in QI.	3.7 (0.54)	3.9 (0.33)	.043
QI is important for improving care.	3.8 (0.48)	3.9 (0.24)	1
I understand how to use the Kotter model.	2.1 (0.91)	3.6 (0.60)	<.001
I know about the SQUIRE guide for publishing QI.	1.5 (0.83)	3.5 (0.62)	<.001
I understand when to pursue IRB review for QI work.	2.2 (1.07)	3.9 (0.33)	<.001
Change in objective knowledge as assessed by multiple-choice questions and QIKAT-R[b]	**Presurvey % Correct**	**Postsurvey % Correct**	
Multiple-choice question content			
Identify type of cognitive error	48	70	.906
Definition of second victim	40	83	<.001
Identify type of system error	34	69	.117
Define type of adverse event	34	54	.088
Define latent error	60	60	.961
Application of just culture	34	43	.317
Identify appropriate patient safety tool	11	75	<.001
Definition of a QI project	76	76	.97
Identify QI tool	91	83	1
	**Presurvey Total Score (*SD*)**	**Postsurvey Total Score (*SD*)**	
QIKAT-R score	4.87 (2.47)	5.17 (2.06)	.656

Abbreviations: IRB, institutional review board; M&M, morbidity and mortality conference; QI, quality improvement; QIKAT-R, Quality Improvement Knowledge Application Tool Revised.
[a]Results of linear mixed models comparing pre/post answers rated on a 4-point Likert scale (1 = *strongly disagree*, 2 = *disagree*, 3 = *agree*, 4 = *strongly agree*). Results are interpreted as differences in scores from pre to post.
[b]Results of binomial mixed models comparing pre/post answers. Results are interpreted as odds ratios of being correct from pre to post.

review of course evaluations revealed additional domains of curricular impact. Learners described value in their opportunity to engage with peers across specialty, the tangible application of the seminar content, and the emphasis on professional skills advancement. Example statements from learners arranged by them are shown below.

- Community and collaboration amongst professional peers:
  - "Interactive, engaging conversation in my group, learned from colleagues outside my specialty."
- Applicability of learning to plan project work:
  - "Helped me workshop my QI project. I have actionable next steps now."
  - "There was a combination of real-world experiences through faculty and workshop aspect."
  - "I valued the discussions on IRB, stats analysis, and components of writing up a QI project."
- Professional skills training for career development:
  - "Loved the opportunity to explicitly plan elevator speech, get feedback, vision a catchy title for my project."
  - "Really emphasized how to make Qi a bigger part of your career."

Discussion

Our pedagogical approach of centralizing QIPS training and harnessing faculty experts from a few select specialties to teach fellow-level trainees across a breadth of specialty disciplines is a novel and effective strategy to promote competencies in QIPS. This approach overcomes two frequently cited challenges: insufficient faculty expertise to teach QIPS skills to fellows and redundancy of QIPS skills development at the fellow level. Through interactive project-based work, fellow-level trainees improved attitudes, knowledge, and self-perceived skills in multiple domains of PS and QI practice. Additional values of the

Copyright © 2020 Neumeier et al. This is an open-access publication distributed under the terms of the Creative Commons Attribution license.

curriculum include interdisciplinary collaboration and learning in a multispecialty setting. Importantly, to promote QIPS education across the training continuum, the AAMC recently defined QIPS competencies specific to learner training level that guide curricular and professional development as well as performance assessment.[25] Our curriculum objectives and educational strategies align well with these competencies, building on trainees' prior experience with QIPS and reintroducing the concepts with a focus on leadership and scholarship.

Our experience designing and teaching the Fellows' Quality and Safety Academy revealed valuable lessons. Implementing the curriculum reinforced the need for fellow-specific QIPS education at our institution. Although we initially designed the course for trainees within the subspecialties of the Department of Medicine, significant interest from fellowship programs outside of the department in both adult and pediatric subspecialties became apparent. In response, we expanded the program after the first year to include trainees from all fellowships. This expansion increased the need to understand the practice of quality and safety across both adult and pediatric hospital systems and required recruitment of additional expert faculty from pediatric and surgical specialty divisions. A more diverse learner audience also required us to include relevant cases for discussion that crossed clinical practice and specialties. Through increasing the size and breadth of the program, we learned that the most robust dialogue occurred in small groups with participants from different disciplines. In order to promote this collaborative problem-solving, we intentionally created diverse groups by encouraging fellows from the same specialty to disperse; this required additional foresight and planning. The program structure also helped us identify specific needs for faculty development in health systems improvement. Specifically, faculty requested to attend certain fellows' seminars to increase their own knowledge and inform their mentorship efforts, and fellows used the seminars to name gaps in faculty mentorship related to their selected QI projects. This led to the development of a faculty-specific seminar series focused on coaching and teaching QIPS. Over time, this focused faculty development will expand the pool of available mentors across the institution.

There are several limitations to our curriculum. First, because our posttest assessments were administered immediately after the sessions, longitudinal knowledge and skills evaluation was not captured. Interestingly, most participants reported prior training in QIPS, yet they performed poorly on preseminar knowledge assessment questions. Thus, more effective measurement of retention, application, and long-term engagement in QIPS is needed to verify mastery. We intend to collect these data via self-report on our GME exit survey; however, even if improved trainee engagement is identified, it may not be fully attributable to the curriculum as the impact of institutional culture change cannot be measured easily. Additionally, although we used previously validated multiple-choice questions and the QIKAT-R, these assessment strategies may not adequately measure performance of the leadership skills taught within these seminars. Challenges to generalizability include faculty resourcing and time to deliver this model, faculty expertise in all domains covered by our curriculum, and need for GME administrative support to coordinate course enrollment.

Future directions include helping to implement and study the curriculum within other training institutions. Within our own institution, we hope to study the effects of this curriculum over time, as we refine both our curricular focus and teaching strategies. A critical next step for this work is to develop performance-assessment strategies to measure whether our curriculum promotes behavior change and learner competencies in QIPS practice. Ultimately, identifying the optimal pedagogical factors that promote sustainable practice in health systems improvement is necessary to guide future curriculum design and implementation.

Appendices

A. Foundations in Patient Safety Teaching Slides.pptx
B. Foundations in Patient Safety Playbook and Small-Group Activities.docx
C. Adverse Events Into QI Teaching Slides.pptx
D. Adverse Events Into QI Playbook and Small-Group Activities.docx
E. Quality in Academics Teaching Slides.pptx
F. Quality in Academics Playbook and Small-Group Activities.docx
G. Foundations in Patient Safety Assessment Survey.docx
H. Adverse Events Into QI Assessment Survey.docx
I. Quality in Academics Assessment Survey.docx

All appendices are peer reviewed as integral parts of the Original Publication.

Anna Neumeier, MD: Assistant Professor, Division of Pulmonary Sciences and Critical Care Medicine, Department of Medicine, University of Colorado School of Medicine

Andrew E. Levy, MD: Assistant Professor, Division of Cardiology, Department of Medicine, University of Colorado School of Medicine

Emily Gottenborg, MD: Assistant Professor, Division of Hospital Medicine, Department of Medicine, University of Colorado School of Medicine

Copyright © 2020 Neumeier et al. This is an open-access publication distributed under the terms of the Creative Commons Attribution license.

Tyler Anstett, DO: Assistant Professor, Division of Hospital Medicine, Department of Medicine, University of Colorado School of Medicine

Read G. Pierce, MD: Associate Professor, Division of Hospital Medicine, Department of Internal Medicine, University of Texas at Austin Dell Medical School

Darlene Tad-y, MD: Associate Professor, Division of Hospital Medicine, Department of Medicine, University of Colorado School of Medicine

Disclosures
None to report.

Funding/Support
None to report.

Prior Presentations
Neumeier A, Gottenborg E. Train the Trainer: QI and PS program for fellows across departments and subspecialties. Presented at: Integrating Quality Conference: Getting on Track to Achieve Health Equity, AAMC; June 6, 2019; Minneapolis, MN.

Ethical Approval
The University of Colorado Institutional Review Board approved this study.

References

1. *ACGME Program Requirements for Graduate Medical Education in Pulmonary Disease and Critical Care Medicine*. Accreditation Council for Graduate Medical Education; 2020. https://www.acgme.org/Portals/0/PFAssets/ProgramRequirements/156_PCCM_2020.pdf?ver=2020-06-29-162350-787
2. Zhou W. Exploring opportunities in quality improvement during fellowship. *J Am Coll Cardiol*. 2016;67(15):1859-1862. https://doi.org/10.1016/j.jacc.2016.03.001
3. Kahn JM, Feemster LC, Fruci CM, et al. Attitudes of pulmonary and critical care training program directors toward quality improvement education. *Ann Am Thorac Soc*. 2015;12(4): 587-590. https://doi.org/10.1513/AnnalsATS.201501-061BC
4. Wong BM, Etchells EE, Kuper A, Levinson W, Shojania KG. Teaching quality improvement and patient safety to trainees: a systematic review. *Acad Med*. 2010;85(9):1425-1439. https://doi.org/10.1097/acm.0b013e3181e2d0c6
5. Bartlett CS, Huerta SA. Creating change: an experiential quality improvement and patient safety curriculum for medical students. *MedEdPORTAL*. 2018;14:10660. https://doi.org/10.15766/mep_2374-8265.10660
6. Djuricich A. A continuous quality improvement (CQI) curriculum for residents. *MedEdPORTAL*. 2007;3:468. https://doi.org/10.15766/mep_2374-8265.468
7. Dumenco L, Monteiro K, George P, McNicoll L, Warrier S, Dollase R. An interactive quality improvement and patient safety workshop for first-year medical students. *MedEdPORTAL*. 2018; 14:10734. https://doi.org/10.15766/mep_2374-8265.10734
8. Garcia C, Goolsarran N. Learning from errors: curriculum guide for the morbidity and mortality conference with a focus on patient safety concepts. *MedEdPORTAL*. 2016;12:10462. https://doi.org/10.15766/mep_2374-8265.10462
9. Keefer P, Orringer K, Vredeveld J, Warrier K, Burrows H. Developing a quality improvement and patient safety toolbox: the curriculum. *MedEdPORTAL*. 2016;12:10385. https://doi.org/10.15766/mep_2374-8265.10385
10. Kiger ME, Bertagnoli T. A project-based, resident-led quality improvement curriculum within a pediatric continuity clinic. *MedEdPORTAL*. 2018;14:10738. https://doi.org/10.15766/mep_2374-8265.10738
11. Reardon CL, Hafer R, Langheim FJP, et al. A quality improvement curriculum for psychiatry residents. *MedEdPORTAL*. 2020;16: 10870. https://doi.org/10.15766/mep_2374-8265.10870
12. Stewart D, Lye C, Lopez M, Mothner B, Camp E, Vachani J. Engaging learners through modules in quality improvement and patient safety. *MedEdPORTAL*. 2016;12:10482. https://doi.org/10.15766/mep_2374-8265.10482
13. Swamy L, Worsham C, Bialas MJ, et al. The 60-minute root cause analysis: a workshop to engage interdisciplinary clinicians in quality improvement. *MedEdPORTAL*. 2018;14:10685. https://doi.org/10.15766/mep_2374-8265.10685
14. Tad-y D, Price L, Cumbler E, Levin D, Wald H, Glasheen J. An experiential quality improvement curriculum for the inpatient setting—part 1: design phase of a QI project. *MedEdPORTAL*. 2014;10:9841. https://doi.org/10.15766/mep_2374-8265.9841
15. Tapper E, Sullivan A, Tess A. Teaching quality improvement on the wards: how we do it. *MedEdPORTAL*. 2015;11:10211. https://doi.org/10.15766/mep_2374-8265.10211
16. Vinas EK, White AB, Rogers RG, Ridgeway JJ, Young AE. Developing best clinical practices through outcomes improvement: an ongoing quality improvement curriculum for faculty and residents. *MedEdPORTAL*. 2018;14:10676. https://doi.org/10.15766/mep_2374-8265.10676
17. Walker M, Rubio D, Horstman M, Trautner B, Stewart D. Stop the blame game: restructuring morbidity and mortality conferences to teach patient safety and quality improvement to residents. *MedEdPORTAL*. 2016;12:10475. https://doi.org/10.15766/mep_2374-8265.10475
18. Werner JA. An integrated, multimodal resident curriculum in patient safety and quality improvement. *MedEdPORTAL*. 2017; 13:10641. https://doi.org/10.15766/mep_2374-8265.10641
19. Worsham C, Swamy L, Gilad A, Abbott J. Quality improvement virtual practicum: the QI simulator. *MedEdPORTAL*. 2018;14: 10670. https://doi.org/10.15766/mep_2374-8265.10670
20. Johnston H. *The Spiral Curriculum: Research Into Practice*. Education Partnerships, Inc; 2012. Accessed March 2, 2020. https://eric.ed.gov/?id=ED538282

Copyright © 2020 Neumeier et al. This is an open-access publication distributed under the terms of the Creative Commons Attribution license.

21. Medical Knowledge Self-Assessment Program, 17th edition (MKSAP 17). *American College of Physicians*; 2015.

22. Singh MK, Ogrinc G, Cox KR, et al. The Quality Improvement Knowledge Application Tool Revised (QIKAT-R). *Acad Med*. 2014; 89(10):1386-1391. https://doi.org/10.1097/ACM.0000000000000456

23. Kotter JP. *Leading Change*. Harvard Business Review Press; 2017.

24. Ogrinc G, Davies L, Goodman D, Batalden P, Davidoff F, Stevens D. SQUIRE 2.0 (Standards for QUality Improvement Reporting Excellence): revised publication guidelines from a detailed consensus process. *BMJ Qual Safety*. 2016;25(12):986-992. https://doi.org/10.1136/bmjqs-2015-004411

25. Association of American Medical Colleges. *Quality Improvement and Patient Safety Competencies Across the Learning Continuum*. Association of American Medical Colleges; 2019. Accessed November 22, 2019. https://store.aamc.org/quality-improvement-and-patient-safety-competencies-across-the-learning-continuum.html

Received: April 28, 2020
Accepted: September 17, 2020
Published: December 30, 2020

Copyright © 2020 Neumeier et al. This is an open-access publication distributed under the terms of the Creative Commons Attribution license.
